# Supplementary material for: Genomewide landscape of gene–metabolome associations in Escherichia coli
Source: Mol Syst Biol. 2017 Jan 16;13(1):907. doi: 10.15252/msb.20167150 (PMC5293155; doi:10.15252/msb.20167150)
Supplement: Supplementary file 1 — Expanded View Figures PDF [file MSB-13-907-s001.pdf]

Expanded View Figures

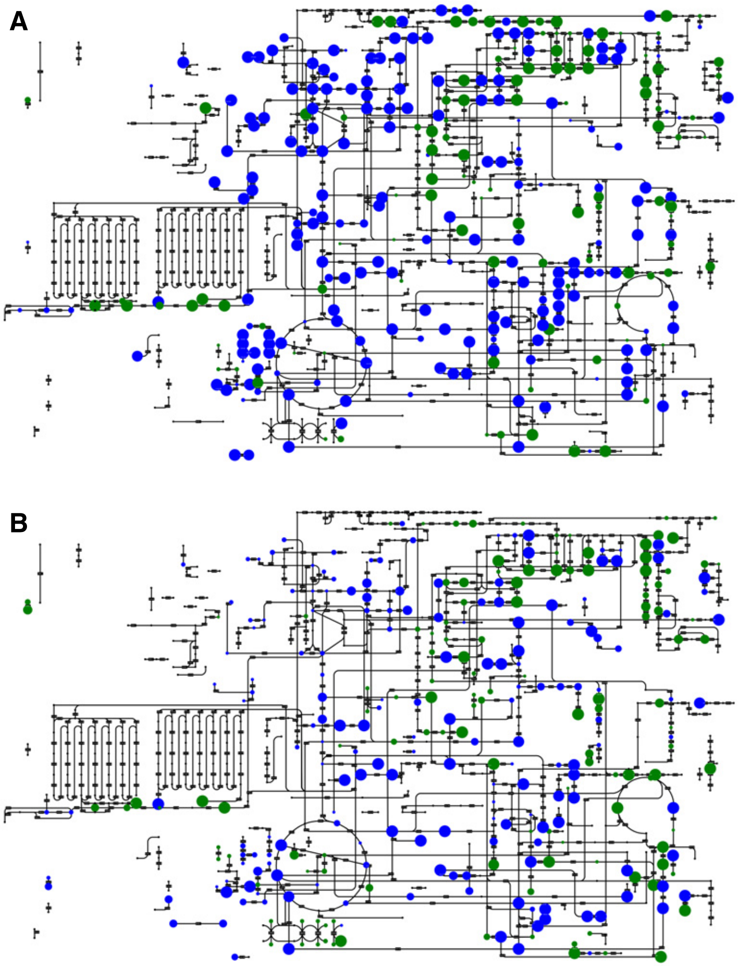

Figure EV1. Annotation coverage.

A, B Metabolites putatively detected in central metabolism for (A) negative mode and (B) positive mode ionization. Green circles represent compounds specifically associated with a single detected ion within 3 mDa tolerance. Blue circles represent compounds that are ambiguously associated with an ion, that is, compounds whose mass was found to match a detected ion but has not a unique molecular weight in the metabolic model used for annotation. The dot size is proportional to the annotation confidence. Additional compounds not depicted on the amended network were also putatively identified.

© 2017 The Authors

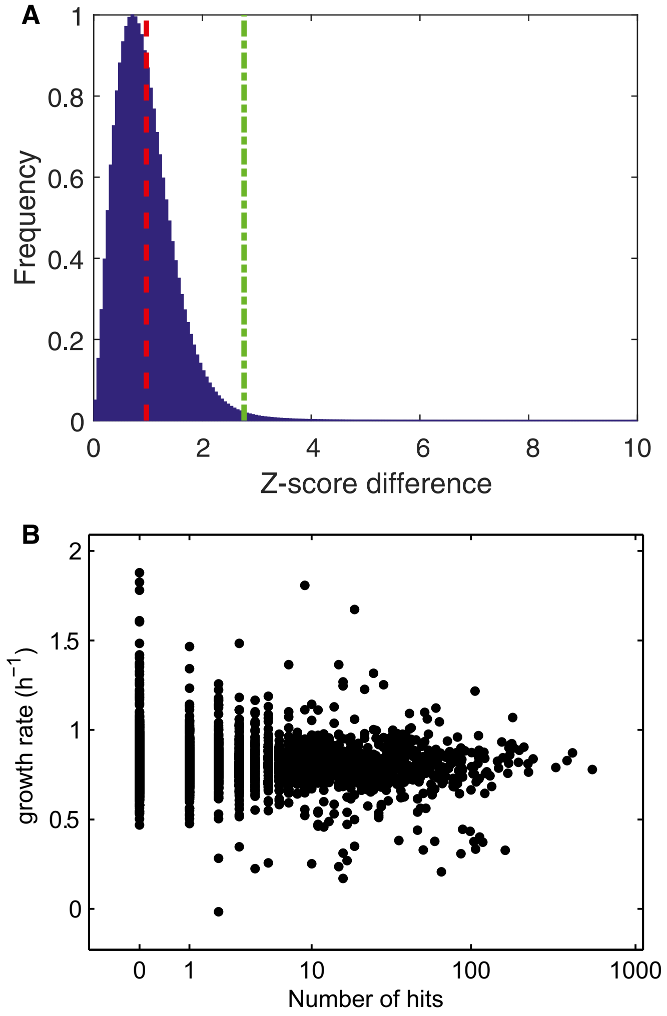

Figure EV2. Biological reproducibility of *Z*-scores.

A For each ion and deletion mutant, the absolute difference between *Z*-scores of two biological replicates separately processed on different days was calculated. The overall distribution is shown in the histogram. Mean value (μ, red dashed line) and 99.9 confidence interval (μ + 3σ, green dashed line).

B Growth-rate dependence of metabolic changes. For each deletion mutant, the number of hits (largest 0.1% of metabolic changes) is plotted against the respective growth rate.

 © 2017 The Authors

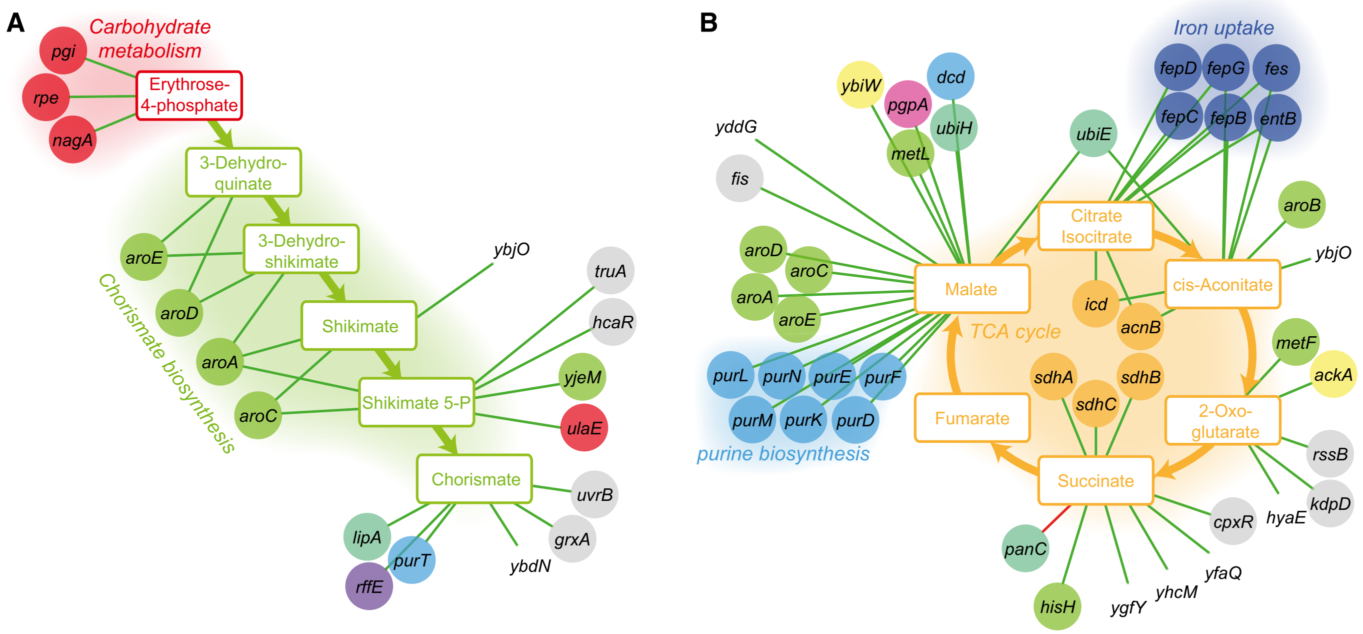

Figure EV3. Genes associated with the detectable intermediates of chorismate metabolism and the TCA cycle.

A, B Genes associated with the detectable intermediates of chorismate metabolism (A) and the TCA cycle (B). To illustrate only high-confidence links, we report only the edges associating a gene to deprotonated metabolites. Color code of metabolites and genes is as in Fig 2. Green and red edges represent increase and decrease in metabolite levels, respectively.

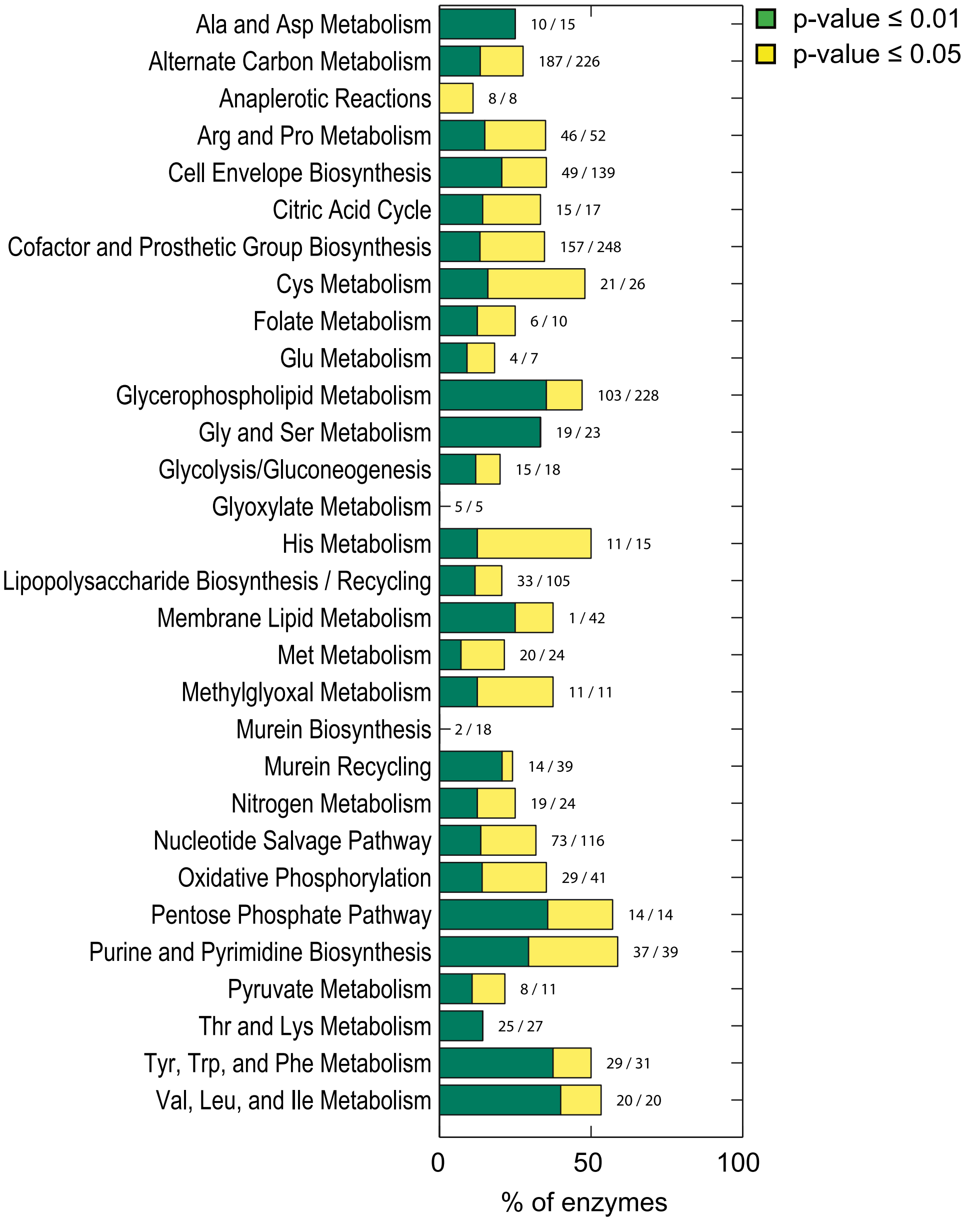

Figure EV4. Enzymes with significant changes of metabolites in the immediate vicinity.
Enzymes with significant changes of metabolites in the immediate vicinity (e.g., distance 1 and different *P*-value cutoff of 0.01 and 0.05, calculated from a permutation test) grouped by metabolic pathways according to the *Escherichia coli* metabolic model. For each pathway, the fraction of detected genes yielding largest changes in the metabolites on the immediate vicinity is indicated on the side of the bar chart plot.

© 2017 The Authors

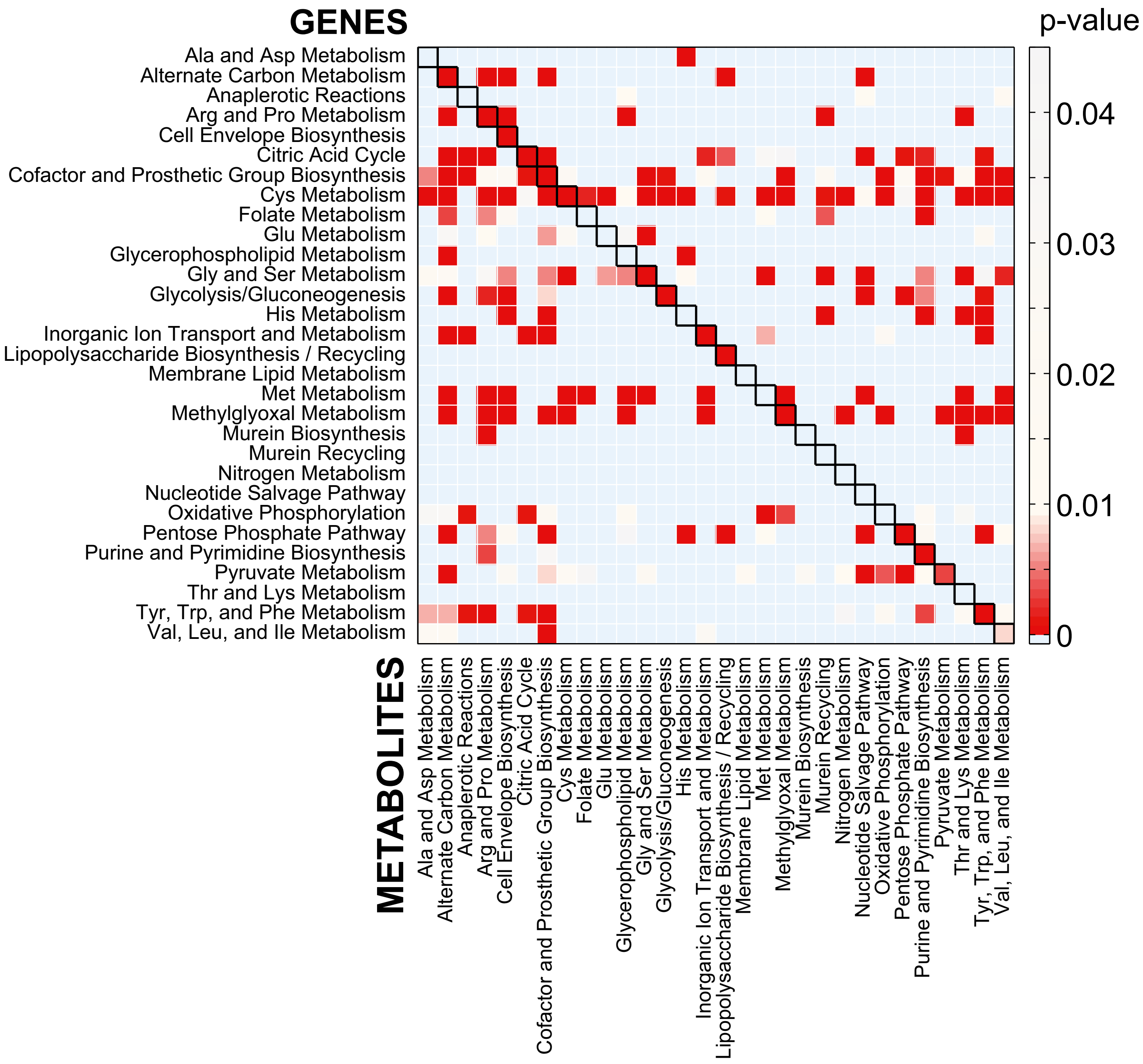

Figure EV5. Pathway enrichment analysis.

Statistical significance of the association between gene deletions (rows) and metabolic changes grouped by metabolic pathways (columns). Metabolic pathways in which gene deletions exhibit a significant (*P*-value < 0.05, calculated from a permutation test) overrepresentation of strong altered metabolites are represented as a squared non-symmetric matrix.

© 2017 The Authors

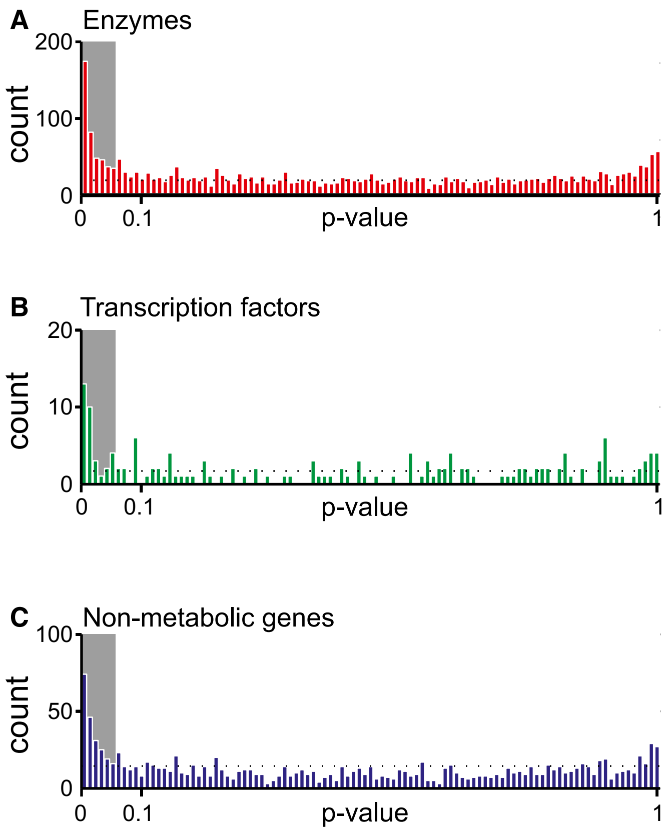

Figure EV6. Locality analysis for enzymes, transcription factors and non-metabolic proteins.

A–C Locality analysis for genes encoding either enzymes, transcription factors, or protein of non-metabolic function [e.g., ribosomes (Andres Leon *et al*, 2009)]. The distribution of the significance (i.e., *P*-value, calculated from a permutation test) of the locality test for each of the tested genes is reported. Genes are grouped in three major classes: enzymes, transcription factors, and genes encoding for proteins that establish a physical interaction with at least one annotated enzyme (i.e., non-metabolic genes). The gray region in the histogram highlights those gene deletions for which a significant local effect can be extrapolated (P-value < 0.05). The overrepresentation of genes within this region supports a tendency for several knockouts to elicit local metabolic effects.

© 2017 The Authors

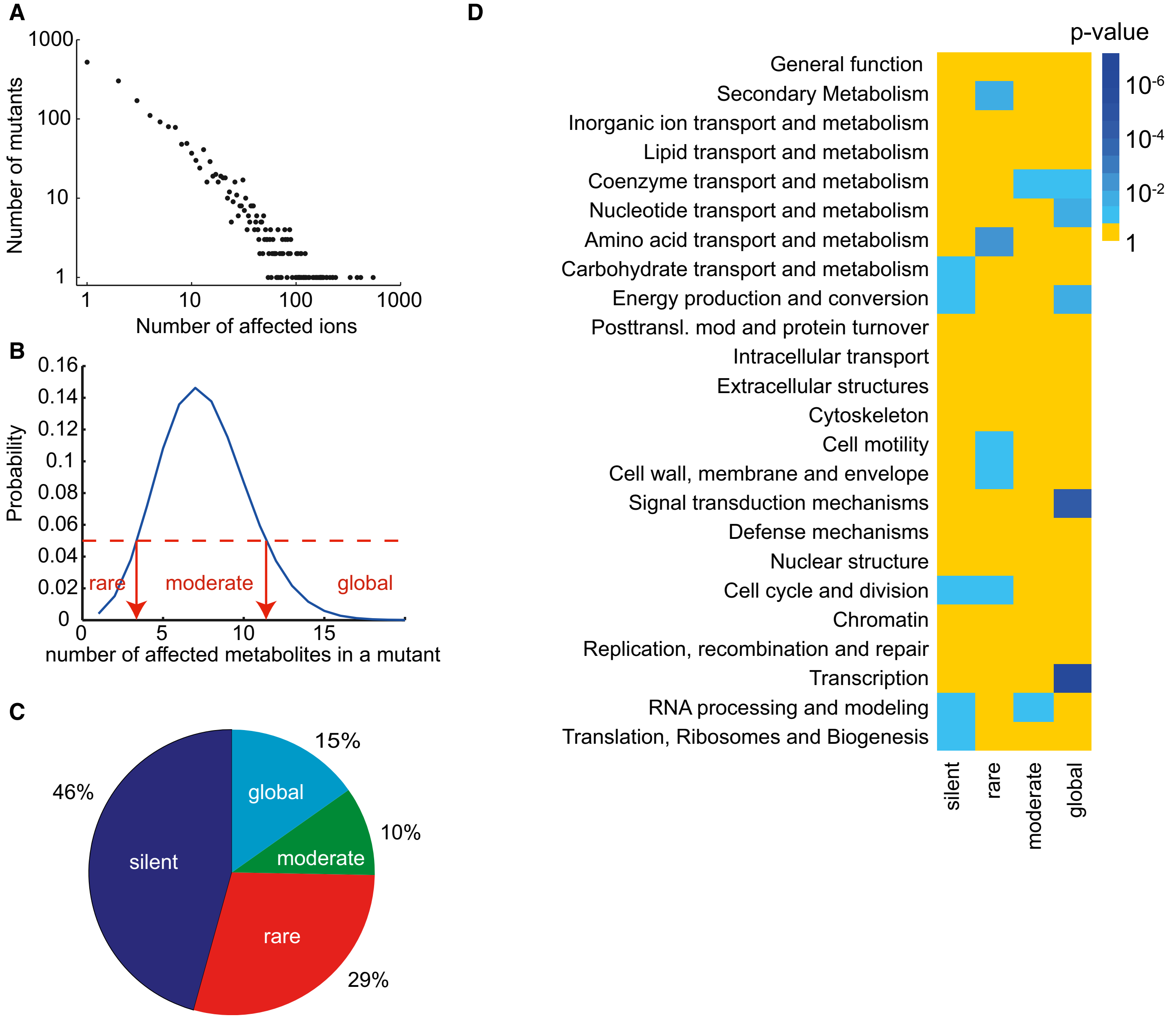

Figure EV7. Distribution of metabolite changes.

A Frequency of metabolite changes in gene knockout mutants in the set of 0.1% most significant changes, seemingly following a power-law distribution.
B Definition of boundaries for the classification of mutants according to the number of differential ions present in the top 0.1% percentile.
C Classification of mutants based on number of detectable changes in metabolome.
D Cellular function enrichment analysis. Enrichment significance (*P*-value) was derived by hypergeometric probability density function. Only significant enrichments with a *P*-value < 0.1 are highlighted.

© 2017 The Authors

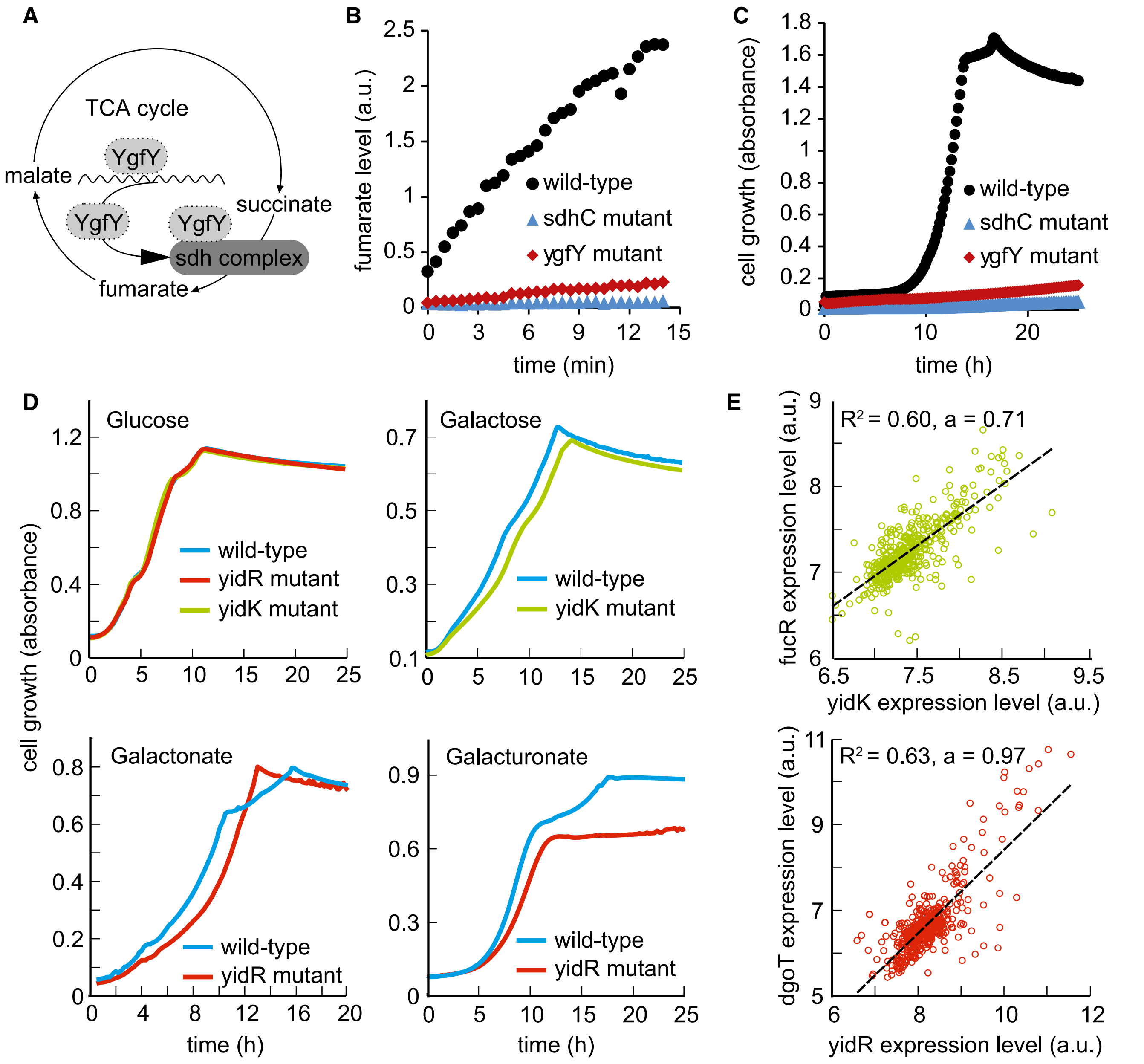

Figure EV8. Potential metabolic functions of YgfY, YidR and YidK.

A Potential functions of YgfY: transcriptional, post-translational, or complex-related modulator of succinate dehydrogenase activity.

B Succinate dehydrogenase activity assay in cell lysates of wild-type strain, sdhC, and ygfY mutants. Fumarate formation from supplied succinate was followed over time by mass spectrometry. Data are shown as mean and standard deviation of three replicates.

C Growth defect of ygfY mutant on succinate minimal medium in comparison with wild-type strain and sdhC mutant. Data are shown as mean and standard deviation of two replicates.

D Growth defect of yidR and yidK mutants on mineral salt medium with the indicated carbon source in comparison with wild-type strain. Solid line indicates mean from at least two replicates.

E Correlation of expression levels over all 907 experiments in the M3D database. R^2 indicates goodness of a linear fit to the data and the strength of the correlation. FucR is a transcriptional activator of operons involved in fucose metabolism, and DgoT is a putative galactonate transporter.

 © 2017 The Authors

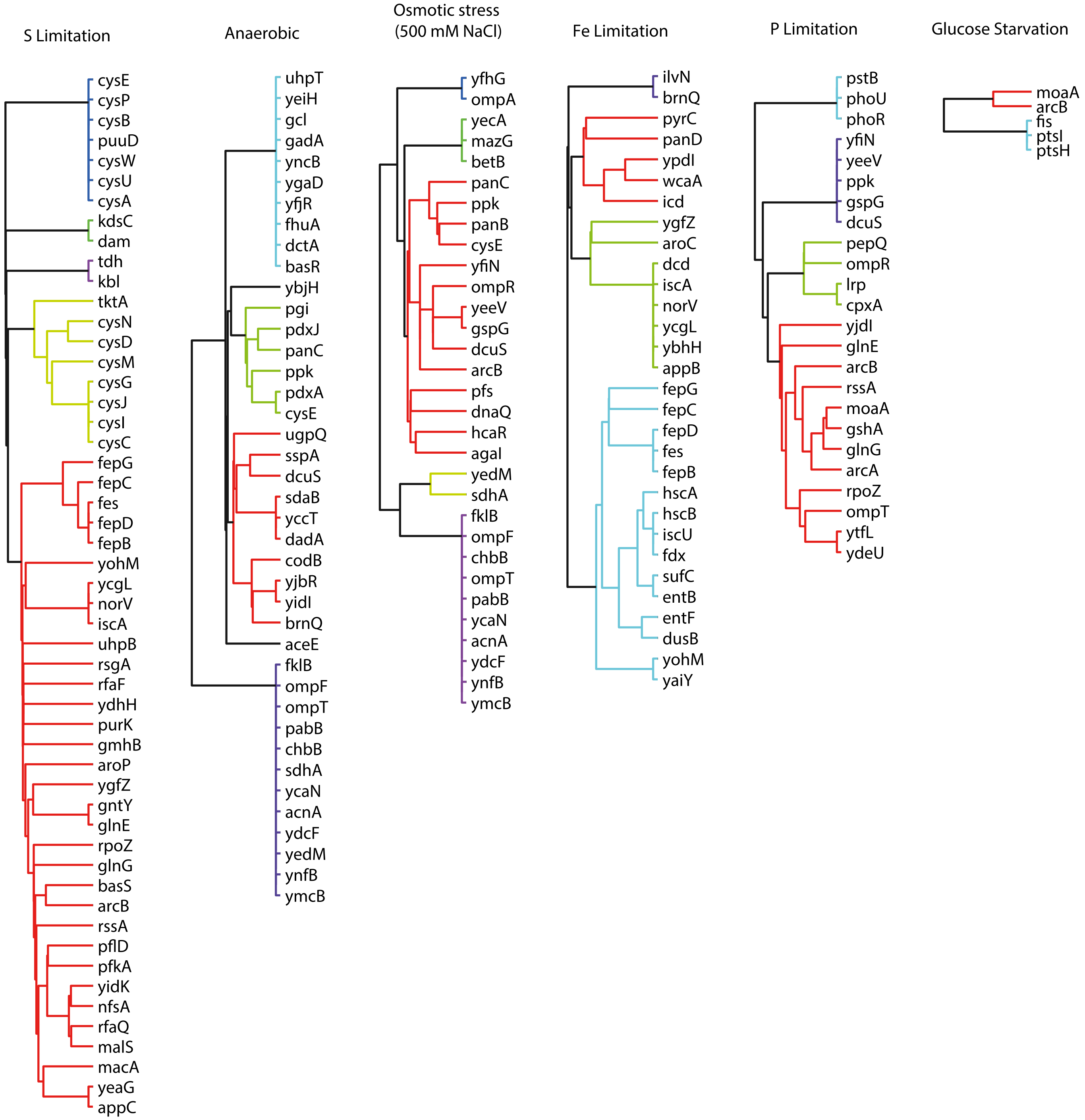

Figure EV9. Predicted genes mediating cellular response to environmental stimuli.
Dendrograms represent genes with significant overlap of differential metabolites between the respective knockouts and environmental perturbations. Genes are grouped on the basis of their topological distance by means of the minimum number of connecting reactions on the metabolic network.

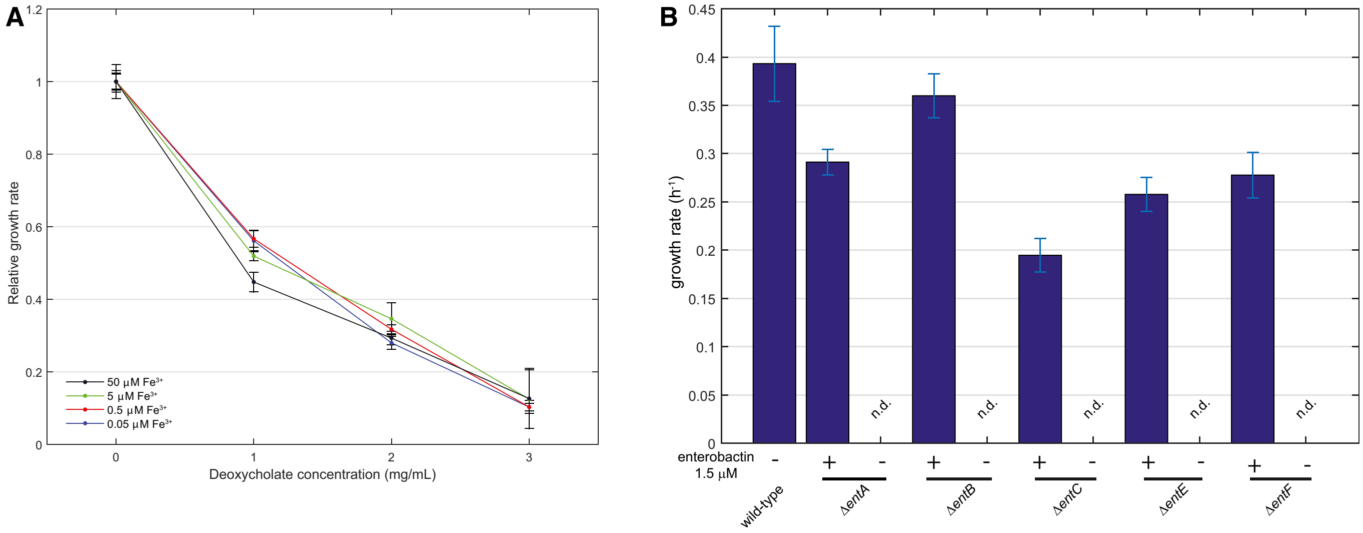

Figure EV10. Growth rates of *Escherichia coli* wild-type strain grown under varying iron, enterobactin, and deoxycholate concentrations.

A *Escherichia coli* wild-type strain was grown on minimal medium casein hydrolysate with iron concentrations ranging from 0.05 up to 50 µM in the presence of 0, 1, 2, or 3 mg/ml deoxycholate. Relative growth rates were calculated during exponential growth and normalized to the 50 µM iron condition. Error bars represent standard deviations from three biological replicates.

B *Escherichia coli* wild-type strain and deletion mutants in enterobactin biosynthesis were grown on minimal medium without amino acids and with 1.5 µM (indicated with +) or without (indicated with −) enterobactin in the absence of deoxycholate. Maximum average growth rates with standard deviations during exponential growth phase were calculated from triplicate cultivations.

 © 2017 The Authors
