# Supplementary material for: Genomewide landscape of gene–metabolome associations in Escherichia coli
Source: Mol Syst Biol. 2017 Jan 16;13(1):907. doi: 10.15252/msb.20167150 (PMC5293155; doi:10.15252/msb.20167150)
Supplement: Supplementary file 4 — Table EV3 [file MSB-13-907-s004.zip › index.html]

 
 
 orphan_merged_110914_pval0011_alpha4 
 
 
 orphan_merged_110914_pval0011_alpha4 
    gene links CLR DIFF IONS KEGG PATHWAYS COG MET Prediction 
  by CLR 
  hits top hit top score Prediction-score hits top hit top score hits top hit top score hits top hit top score hits top hit top score overlap
   yidK   ecocyc   kegg   phen   254   rmuC   ecocyc   kegg  15.0  21.1   119  UDPgalactose  C00052  7.731
  18  Bacterial secretion system 0  11  RNA polymerase 0.000  26  S-Ribosyl-L-homocysteine 0.000 1.000
   ybjO   ecocyc   kegg   phen   72   ubiE   ecocyc   kegg  16.5  14.6   182  N-Carbamoyl-L-aspartate  C00438  14.833
  15  Microbial metabolism in diverse environments 1e-16  14  Phenylalanine, tyrosine and tryptophan biosynthesis 0.000  13  3-Dehydroshikimate 0.000 1.000
   yhhK   ecocyc   kegg   phen   162   panE   ecocyc   kegg  22.8  14.4   205  (R)-Pantoate  C00522  15.881
  30  Microbial metabolism in diverse environments 8e-10  17  Chlorocyclohexane and chlorobenzene degradation 0.000  38  (R)-Pantoate 0.000 1.000
   yjeK   ecocyc   kegg   phen   1128   yjfJ   ecocyc   kegg  19.0  11.9   215  D-Allose 6-phosphate  C02962  4.488
  28  Fructose and mannose metabolism 1e-10  9  Chlorocyclohexane and chlorobenzene degradation 0.000  39  D-Mannose 0.000 1.000
   yahJ   ecocyc   kegg   phen   307   emrE   ecocyc   kegg  17.1  10.7   62  N-Acetyl-L-glutamate  C00624  6.805
  23  Arginine and proline metabolism 1e-08  4  Ribosome 0.000  33  Deoxyuridine 0.011 1.000
   yjbJ   ecocyc   kegg   phen   340   yjhG   ecocyc   kegg  10.3  9.9   42  D-Glycero-D-manno-heptose 7-phosphate  C07836  4.570
  17  Benzoate degradation 9e-07  8  Chlorocyclohexane and chlorobenzene degradation 0.000  28  (S)-Dihydroorotate 0.000 1.000
   ygfY   ecocyc   kegg   phen   105   yghT   ecocyc   kegg  10.0  8.6   62  D-Lactaldehyde  C00937  11.039
  17  Purine metabolism 2e-05  18  Valine, leucine and isoleucine degradation 0.000  7  Succinate 0.011 1.000
   yhbT   ecocyc   kegg   phen   405   rpmG   ecocyc   kegg  34.1  8.0   118  sulfur dioxide  C09306  10.798
  20  Limonene and pinene degradation 2e-10  9  Bacterial chemotaxis 0.000  39  Acetoacetate 0.000 1.000
   yacL   ecocyc   kegg   phen   327   ybjR   ecocyc   kegg  18.2  7.8   184  Dethiobiotin  C01909  8.805
  22  Lipoic acid metabolism 0  13  Ethylbenzene degradation 0.001  40  D-Mannose 6-phosphate 0.009 1.000
   ycfM   ecocyc   kegg   phen   35   stfE   ecocyc   kegg  24.5  7.7   23  tetradecenoate (n-C14:1)  tetradecenoate (n-C14:1)  7.883
  8  Limonene and pinene degradation 3e-15  11  Terpenoid backbone biosynthesis 0.000  7  tetradecenoate (n-C14:1) 0.000 1.000
   yahO   ecocyc   kegg   phen   115   ybhP   ecocyc   kegg  9.7  7.4   22  L-Tyrosine  C00082  7.449
  11  C5-Branched dibasic acid metabolism 8e-12  11  Chlorocyclohexane and chlorobenzene degradation 0.000  12  L-Tyrosine 0.001 1.000
   yeiH   ecocyc   kegg   phen   130   yccT   ecocyc   kegg  22.6  6.6   35  Dodecanoate (n-C12:0)  C02679  15.712
  14  Limonene and pinene degradation 3e-12  2  Caprolactam degradation 0.007  11  3-Phospho-D-glycerate 0.000 1.000
   yfjG   ecocyc   kegg   phen   133   appB   ecocyc   kegg  18.6  6.6   89  3-Dehydroshikimate  C02637  9.808
  19  D-Alanine metabolism 2e-08  9  Phenylalanine, tyrosine and tryptophan biosynthesis 0.000  12  3-Dehydroshikimate 0.000 1.000
   yrbA   ecocyc   kegg   phen   182   slyD   ecocyc   kegg  18.2  6.3   47  N-Acetyl-L-glutamate  C00624  9.697
  9  D-Glutamine and D-glutamate metabolism 2e-10  2  Ribosome 0.000  20  Deoxyuridine 0.002 1.000
   ybeU   ecocyc   kegg   phen   143   yqcD   ecocyc   kegg  15.7  6.2   86  N1-Acetylspermidine  C00612  9.696
  21  Tyrosine metabolism 4e-08  18  Pyruvate metabolism 0.000  17  (R)-Pantoate 0.000 1.000
   ytfM   ecocyc   kegg   phen   131   hisG   ecocyc   kegg  14.6  5.6   55  5,6-Dimethylbenzimidazole  C03114  5.165
  9  Naphthalene degradation 2e-07  17  Porphyrin and chlorophyll metabolism 0.000  13  Cytosine 0.001 1.000
   yagU   ecocyc   kegg   phen   106   yneK   ecocyc   kegg  15.7  5.3   33  L-Tyrosine  C00082  7.835
  3  Nicotinate and nicotinamide metabolism 0.0001  5  Pantothenate and CoA biosynthesis 0.000  4  L-Tyrosine 0.000 1.000
   yfiN   ecocyc   kegg   phen   168   ypdI   ecocyc   kegg  15.9  5.3   88  S-Adenosylmethioninamine  C01137  8.839
  18  ABC transporters 5e-08  5  RNA polymerase 0.000  11  (S)-Dihydroorotate 0.000 1.000
   ydhP   ecocyc   kegg   phen   202   mdtJ   ecocyc   kegg  18.7  5.2   35  2-Oxo-3-hydroxy-4-phosphobutanoate  C06054  5.896
  4  Naphthalene degradation 5e-07  7  Porphyrin and chlorophyll metabolism 0.001  22  (S)-Dihydroorotate 0.000 1.000
   ykfA   ecocyc   kegg   phen   213   ldhA   ecocyc   kegg  19.3  4.8   146  tetradecanoate (n-C14:0)  C06424  10.383
  22  Fructose and mannose metabolism 1e-11  16  Chlorocyclohexane and chlorobenzene degradation 0.000  8  Sedoheptulose 7-phosphate 0.001 1.000
   ydfI   ecocyc   kegg   phen   173   ydjF   ecocyc   kegg  15.2  4.8   64  N-Acetyl-L-glutamate  C00624  6.411
  9  Lysine biosynthesis 6e-09  16  Nucleotide excision repair 0.000  23  N-Acetyl-L-glutamate 0.000 1.000
   yqcE   ecocyc   kegg   phen   54   panE   ecocyc   kegg  17.1  4.6   25  (R)-Pantoate  C00522  6.419
  23  Microbial metabolism in diverse environments 2e-07  10  Pantothenate and CoA biosynthesis 0.000  6  (R)-Pantoate 0.000 1.000
   yhcM   ecocyc   kegg   phen   113   ygfY   ecocyc   kegg  9.4  4.4   21  D-Lactaldehyde  C00937  6.183
  19  Chlorocyclohexane and chlorobenzene degradation 2e-07  4  Toluene degradation 0.000  3  Succinate 0.000 1.000
   ybhH   ecocyc   kegg   phen   114   appB   ecocyc   kegg  15.9  4.4   50  Succinate  C00042  9.792
  9  Lysine degradation 2e-09  14  Ubiquinone and other terpenoid-quinone biosynthesis 0.000  12  3-Dehydroshikimate 0.000 1.000
   ybaZ   ecocyc   kegg   phen   66   cysD   ecocyc   kegg  19.4  4.1   26  2-Oxopent-4-enoate  C00596  9.192
  24  Glycine, serine and threonine metabolism 1e-06  16  Sulfur metabolism 0.000  19  O-Acetyl-L-serine 0.000 1.000
   ycgL   ecocyc   kegg   phen   155   yaiY   ecocyc   kegg  10.6  3.8   64  2-C-methyl-D-erythritol 2,4-cyclodiphosphate  C11453  6.824
  12  Valine, leucine and isoleucine biosynthesis 1e-15  8  Biosynthesis of siderophore group nonribosomal peptides 0.000  14  Iminoaspartate 0.000 1.000
   ydiU   ecocyc   kegg   phen   155   yaaH   ecocyc   kegg  14.9  3.8   23  Dodecanoate (n-C12:0)  C02679  6.740
  21  Alanine, aspartate and glutamate metabolism 2e-07  3  Lysine degradation 0.007  8  (S)-Dihydroorotate 0.000 1.000
   ydiN   ecocyc   kegg   phen   62   ydiB   ecocyc   kegg  17.8  3.7   15  2-Dehydro-3-deoxy-D-galactonate  C01216  4.399
  31  Microbial metabolism in diverse environments 9e-10  14  Lipoic acid metabolism 0.000  10  (R)-Pantoate 0.000 1.000
   ybfF   ecocyc   kegg   phen   157   macB   ecocyc   kegg  8.9  3.7   5  3-Dehydroshikimate  C02637  4.649
  3  Microbial metabolism in diverse environments 0  3  Bacterial secretion system 0.001  6  3-Dehydroshikimate 0.000 1.000
   ydjO   ecocyc   kegg   phen   101   yceO   ecocyc   kegg  15.2  3.7   22  trans-Aconitate  C02341  5.089
  7  C5-Branched dibasic acid metabolism 0  13  Biosynthesis of siderophore group nonribosomal peptides 0.000  19  Iminoaspartate 0.000 1.000
   yneK   ecocyc   kegg   phen   187   yagU   ecocyc   kegg  15.7  3.6   86  N-Acetyl-L-glutamate  C00624  9.280
  21  Bacterial secretion system 0  5  RNA polymerase 0.000  7  L-Tyrosine 0.002 1.000
   yajG   ecocyc   kegg   phen   78   yehW   ecocyc   kegg  8.4  3.4   21  5'-deoxyribose  5'-deoxyribose  7.966
  25  Inositol phosphate metabolism 5e-07  12  Phosphotransferase system (PTS) 0.000  21  (R)-Pantoate 0.000 1.000
   ydeQ   ecocyc   kegg   phen   198   yeeU   ecocyc   kegg  13.2  3.3   56  E-3-carboxy-2-pentenedioate 6-methyl ester  C11514  5.487
  17  Lysine degradation 4e-09  7  Ubiquinone and other terpenoid-quinone biosynthesis 0.000  10  L-Lactate 0.002 1.000
   yraI   ecocyc   kegg   phen   82   sodB   ecocyc   kegg  10.7  3.3   3  3-Methyl-2-oxobutanoate  C00141  4.065
  12  Microbial metabolism in diverse environments 0  4  Valine, leucine and isoleucine biosynthesis 0.000  6  3-Methyl-2-oxobutanoate 0.000 1.000
   yddG   ecocyc   kegg   phen   138   yneK   ecocyc   kegg  8.6  3.2   224  5'-deoxyribose  5'-deoxyribose  5.787
  24  Fructose and mannose metabolism 4e-10  3  Lipoic acid metabolism 0.004  13  D-Tagatose 1,6-biphosphate 0.000 1.000
   yjfZ   ecocyc   kegg   phen   173   yidG   ecocyc   kegg  9.2  3.2   45  L-Histidinol  C00860  7.336
  11  Bacterial secretion system 0  5  Homologous recombination 0.000  19  3'-Phosphoadenylyl sulfate 0.004 1.000
   yjfJ   ecocyc   kegg   phen   450   yjeK   ecocyc   kegg  19.0  3.1   72  D-Allose 6-phosphate  C02962  3.804
  7  Lipopolysaccharide biosynthesis 5e-08  6  Chlorocyclohexane and chlorobenzene degradation 0.000  32  D-Fructose 1-phosphate 0.010 1.000
   ygiS   ecocyc   kegg   phen   110   pyrB   ecocyc   kegg  14.3  3.1   22  gamma-hydroxybutyrate  C00989  5.688
  9  Propanoate metabolism 8e-05  22  Ethylbenzene degradation 0.000  36  Dihydroxyacetone 0.007 1.000
   ydaL   ecocyc   kegg   phen   163   ybjR   ecocyc   kegg  15.6  3.1   30  7-aminomethyl-7-deazaguanine  C01449  4.128
  13  D-Glutamine and D-glutamate metabolism 6e-11  4  Two-component system 0.003  11  Nitrous oxide 0.000 1.000
   ylbF   ecocyc   kegg   phen   89   yfdF   ecocyc   kegg  29.5  3.1   76  4-Phospho-L-aspartate  C03082  5.776
  11  D-Glutamine and D-glutamate metabolism 2e-10  10  Taurine and hypotaurine metabolism 0.000  8  Hexanoate (n-C6:0) 0.000 1.000
   yicN   ecocyc   kegg   phen   183   yibQ   ecocyc   kegg  8.8  3.0   2  dehydroglycine  C15809  4.017
 &nbsp &nbsp &nbsp  5  Protein export 0.003  9  dehydroglycine 0.000 1.000
   ynfM   ecocyc   kegg   phen   306   sbmC   ecocyc   kegg  14.3  3.0   20  N-Carbamoyl-L-aspartate  C00438  4.352
  5  Caprolactam degradation 1e-05  8  Chlorocyclohexane and chlorobenzene degradation 0.000  15  (S)-Dihydroorotate 0.000 1.000
   ydjZ   ecocyc   kegg   phen   90   ybeU   ecocyc   kegg  12.0  3.0   10  5'-deoxyribose  5'-deoxyribose  5.295
  35  Xylene degradation 2e-08  7  Lipoic acid metabolism 0.000  12  (R)-Pantoate 0.000 1.000
   yfcV   ecocyc   kegg   phen   314   cysH   ecocyc   kegg  11.2  2.9   44  3-Methyl-2-oxobutanoate  C00141  3.603
  19  Lysine degradation 1e-07  14  Chlorocyclohexane and chlorobenzene degradation 0.000  27  3-Methyl-2-oxobutanoate 0.000 1.000
   yqhH   ecocyc   kegg   phen   286   fkpA   ecocyc   kegg  13.2  2.8   51  3-Hydroxypropanoate  C01013  5.361
  9  Benzoate degradation 3e-07  14  Flagellar assembly 0.000  44  Bicarbonate 0.002 1.000
   yjjK   ecocyc   kegg   phen   214   priC   ecocyc   kegg  18.8  2.8   48  N1-Acetylspermidine  C00612  5.484
  16  D-Glutamine and D-glutamate metabolism 4e-05  10  Alanine, aspartate and glutamate metabolism 0.000  22  Guanine 0.009 1.000
   yehI   ecocyc   kegg   phen   145   crp   ecocyc   kegg  11.3  2.7   17  4-Aminobenzoate  C00568  4.836
  14  Novobiocin biosynthesis 8e-06  3  Pantothenate and CoA biosynthesis 0.003  3  2-Oxobutanoate 0.001 1.000
   yiaY   ecocyc   kegg   phen   135   yggP   ecocyc   kegg  14.8  2.7   19  cis-Aconitate  C00417  4.929
  1  alpha-Linolenic acid metabolism 0  5  Ubiquinone and other terpenoid-quinone biosynthesis 0.000  16  3-Dehydroshikimate 0.000 1.000
   yhbS   ecocyc   kegg   phen   405   yjeK   ecocyc   kegg  13.8  2.7   8  Isopentenyl diphosphate  C00129  3.591
  9  Nitrogen metabolism 3e-05  13  Chlorocyclohexane and chlorobenzene degradation 0.000  38  ITP 0.001 1.000
   yjhI   ecocyc   kegg   phen   142   yjhG   ecocyc   kegg  10.2  2.7   8  (S)-3-Methyl-2-oxopentanoate  C00671  3.572
 &nbsp &nbsp &nbsp  5  Chlorocyclohexane and chlorobenzene degradation 0.000  14  2,3-Dihydro-2,3-dihydroxybenzoate 0.000 1.000
   ycjY   ecocyc   kegg   phen   94   yccJ   ecocyc   kegg  10.3  2.7   6  Propanal  C00479  4.322
  1  Histidine metabolism 7e-05  3  Vitamin B6 metabolism 0.000  7  L-alanine-D-glutamate 0.000 1.000
   ygcW   ecocyc   kegg   phen   519   gpp   ecocyc   kegg  13.0  2.7   27  Hydroquinone  C00530  4.122
  7  Pyrimidine metabolism 1e-09  4  Chlorocyclohexane and chlorobenzene degradation 0.000  23  cyclic pyranopterin monophosphate 0.000 1.000
   yabI   ecocyc   kegg   phen   119   torI   ecocyc   kegg  7.9  2.7   12  D-Lactaldehyde  C00937  3.592
  1  Taurine and hypotaurine metabolism 0  3  Bacterial secretion system 0.000  6  Propionate (n-C3:0) 0.000 1.000
   yhdL   ecocyc   kegg   phen   482   msrA   ecocyc   kegg  26.3  2.6   115  Betaine aldehyde  C00576  8.084
  18  Limonene and pinene degradation 2e-16  4  Ribosome 0.000  44  Dimethyl sulfide 0.001 1.000
   yfaA   ecocyc   kegg   phen   106   ptrB   ecocyc   kegg  9.1  2.6   22  O-Phospho-L-serine  C01005  3.836
  13  Pyruvate metabolism 4e-06  4  Oxidative phosphorylation 0.000  9  Succinate 0.007 1.000
   yigF   ecocyc   kegg   phen   200   yjeK   ecocyc   kegg  7.9  2.6   6  Acetoacetate  C00164  3.788
  1  Glycerophospholipid metabolism 0  11  Chlorocyclohexane and chlorobenzene degradation 0.000  12  2-Oxobutanoate 0.007 1.000
   yebG   ecocyc   kegg   phen   246   yobD   ecocyc   kegg  19.0  2.5   44  cyclopropane phosphatidylethanolamine (dihexadec-9,10-cyclo-anoyl, n-C16:0 cyclo)  cyclopropane phosphatidylethanolamine (dihexadec-9,10-cyclo-anoyl, n-C16:0 cyclo)  4.878
  7  Arachidonic acid metabolism 0  2  Two-component system 0.001  20  Deoxyuridine 0.005 1.000
   ydfH   ecocyc   kegg   phen   165   ydhP   ecocyc   kegg  14.0  2.5   27  Quinate  C00296  4.956
  10  Caprolactam degradation 2e-06  2  Fatty acid biosynthesis 0.002  9  (S)-Dihydroorotate 0.000 1.000
   yhfU   ecocyc   kegg   phen   244   yjeI   ecocyc   kegg  17.9  2.5   15  L-seryl-AMP  C05820  4.492
  4  Chloroalkane and chloroalkene degradation 4e-07  5  Ribosome 0.000  21  Dimethyl sulfide 0.000 1.000
   yebQ   ecocyc   kegg   phen   177   putP   ecocyc   kegg  9.8  2.5   79  4-Hydroxy-L-threonine  C06056  6.659
  24  Microbial metabolism in diverse environments 4e-10  1  Sphingolipid metabolism 0.002  5  Propionate (n-C3:0) 0.000 1.000
   yidR   ecocyc   kegg   phen   547   dgoT   ecocyc   kegg  12.7  2.4   8  5,6,7,8-tetrahydropteridine  C05650  3.861
 &nbsp &nbsp &nbsp  8  Chlorocyclohexane and chlorobenzene degradation 0.000  49  Bicarbonate 0.004 1.000
   yjcZ   ecocyc   kegg   phen   119   eptA   ecocyc   kegg  12.4  2.4   5  Thiamin monophosphate  C01081  3.631
  2  Toluene degradation 0  11  Chlorocyclohexane and chlorobenzene degradation 0.000  9  Hydroxypyruvate 0.000 1.000
   yheS   ecocyc   kegg   phen   103   feoB   ecocyc   kegg  14.6  2.4   9  octadecanoate (n-C18:0)  C01530  4.337
  10  Lysine degradation 7e-10  3  ABC transporters 0.000  18  Sulfate 0.003 1.000
   ybhM   ecocyc   kegg   phen   147   sbmC   ecocyc   kegg  10.5  2.4   18  D-Tagatose 1,6-biphosphate  C03785  3.588
  7  Inositol phosphate metabolism 2e-07  11  Ubiquinone and other terpenoid-quinone biosynthesis 0.000  8  5-O-(1-Carboxyvinyl)-3-phosphoshikimate 0.000 1.000
   yjfK   ecocyc   kegg   phen   425   yjeK   ecocyc   kegg  15.6  2.4   51  Hypoxanthine  C00262  3.566
  10  Lipopolysaccharide biosynthesis 5e-08  8  Pantothenate and CoA biosynthesis 0.000  34  Bicarbonate 0.004 1.000
   ybcK   ecocyc   kegg   phen   127   ymfD   ecocyc   kegg  11.7  2.3   19  trans-Aconitate  C02341  3.895
  6  C5-Branched dibasic acid metabolism 0  6  Biosynthesis of siderophore group nonribosomal peptides 0.000  10  2,3-Dihydroxybenzoate 0.000 1.000
   yfjK   ecocyc   kegg   phen   190   yahL   ecocyc   kegg  15.0  2.3   80  D-Glucosamine 1-phosphate  C06156  3.996
  21  alpha-Linolenic acid metabolism 4e-12  3  Oxidative phosphorylation 0.000  11  N-Acetyl-D-glucosamine 6-phosphate 0.009 1.000
   yqgA   ecocyc   kegg   phen   113   ypfI   ecocyc   kegg  16.4  2.3   5  2-Acyl-sn-glycero-3-phosphoethanolamine (n-C16:1)  C05973  5.749
  6  Biosynthesis of secondary metabolites 5e-05  1  Pyrimidine metabolism 0.004  18  Inosine 0.001 1.000
   ybjH   ecocyc   kegg   phen   103   ybdN   ecocyc   kegg  17.3  2.2   38  o-Succinylbenzoate  C02730  6.207
  8  Ascorbate and aldarate metabolism 3e-05  8  Lipoic acid metabolism 0.000  18  dGDP 0.002 1.000
   ypdI   ecocyc   kegg   phen   276   yfjV   ecocyc   kegg  22.8  2.2   151  (S)-Dihydroorotate  C00337  6.248
  12  Arachidonic acid metabolism 0  7  RNA polymerase 0.000  11  D-Lactate 0.001 1.000
   ygiQ   ecocyc   kegg   phen   224   yigE   ecocyc   kegg  11.3  2.2   22  Thiosulfate  C00320  4.022
  6  Phenylalanine metabolism 1e-08  15  Chlorocyclohexane and chlorobenzene degradation 0.000  23  Phenylpyruvate 0.001 1.000
   ybaA   ecocyc   kegg   phen   158   emrE   ecocyc   kegg  9.6  2.1   11  4-Aminobutanoate  C00334  6.332
  7  Nicotinate and nicotinamide metabolism 2e-05  4  Aminoacyl-tRNA biosynthesis 0.000  15  alpha-D-Ribose 5-phosphate 0.003 1.000
   yaaA   ecocyc   kegg   phen   111  yadC  ecocyc   kegg  5.8 0.0 &nbsp &nbsp &nbsp
 &nbsp &nbsp &nbsp  6  Valine, leucine and isoleucine biosynthesis 0.002 &nbsp &nbsp &nbsp
   yaaH   ecocyc   kegg   phen   100  ydiU  ecocyc   kegg  14.9 0.0  14  Deoxyadenosine  C00559  6.638
  10  C5-Branched dibasic acid metabolism 2e-11  10  Glycolysis / Gluconeogenesis 0.000  5  (-)-Ureidoglycolate 0.000 0.000
   yaaI   ecocyc   kegg   phen   161  yfiQ  ecocyc   kegg  16.6 0.0  2  crotonobetaine  C04114  3.579
  2  Amino sugar and nucleotide sugar metabolism 0.0007  2  Pyrimidine metabolism 0.001  14  D-Allose 0.000 0.000
   yaaJ   ecocyc   kegg   phen   69  yaiX  ecocyc   kegg  16.8 0.0 &nbsp &nbsp &nbsp
  1  Biosynthesis of secondary metabolites 0  1  Nitrogen metabolism 0.003 &nbsp &nbsp &nbsp
   yaaU   ecocyc   kegg   phen   230  yjeK  ecocyc   kegg  11.3 0.0  6  N-Acetyl-L-glutamate  C00624  4.514
  3  Tryptophan metabolism 7e-09  6  Pantothenate and CoA biosynthesis 0.000  28  dTDP-4-amino-4,6-dideoxy-D-galactose 0.000 0.000
   yaaW   ecocyc   kegg   phen   96  ilvM  ecocyc   kegg  7.9 0.0 &nbsp &nbsp &nbsp
 &nbsp &nbsp &nbsp  5  Chlorocyclohexane and chlorobenzene degradation 0.000 &nbsp &nbsp &nbsp
   yaaX   ecocyc   kegg   phen   119  ycgR  ecocyc   kegg  7.8 0.0  5  Xanthosine 5'-phosphate  C00655  3.862
  3  Ethylbenzene degradation 4e-05  7  Geraniol degradation 0.001  20  3-Oxodecanoyl-CoA 0.000 0.000
   yaaY   ecocyc   kegg   phen   249  yrhA  ecocyc   kegg  9.4 0.0  1  &nbsp &nbsp
 &nbsp &nbsp &nbsp  1  Dioxin degradation 0.005  7  bis-molybdenum cofactor 0.000 0.000
   yabP   ecocyc   kegg   phen   160  ygcG  ecocyc   kegg  8.1 0.0  2  Biotin  C00120  -4.598
  3  Biotin metabolism 7e-06  2  Mismatch repair 0.003  4  D-Ribulose 5-phosphate 0.000 0.000
   yacC   ecocyc   kegg   phen   103  yaaI  ecocyc   kegg  14.0 0.0 &nbsp &nbsp &nbsp
 &nbsp &nbsp &nbsp  3  Pyrimidine metabolism 0.000 &nbsp &nbsp &nbsp
   yacF   ecocyc   kegg   phen   66  ydjM  ecocyc   kegg  8.4 0.0  4  gamma-hydroxybutyrate  C00989  3.555
  5  Propanoate metabolism 2e-05  6  Histidine metabolism 0.000  4  Formate 0.001 0.000
   yacG   ecocyc   kegg   phen   119  yjhC  ecocyc   kegg  8.4 0.0  8  sulfur dioxide  C09306  4.097
 &nbsp &nbsp &nbsp  3  Valine, leucine and isoleucine biosynthesis 0.000  3  L-Xylulose 0.000 0.000
   yacH   ecocyc   kegg   phen   149  envZ  ecocyc   kegg  10.6 0.0 &nbsp &nbsp &nbsp
  1  Biosynthesis of secondary metabolites 0  5  Pantothenate and CoA biosynthesis 0.000 &nbsp &nbsp &nbsp
   yadC   ecocyc   kegg   phen   86  yaaA  ecocyc   kegg  5.8 0.0 &nbsp &nbsp &nbsp
 &nbsp &nbsp &nbsp  3  Phenylalanine, tyrosine and tryptophan biosynthesis 0.001 &nbsp &nbsp &nbsp
   yadD   ecocyc   kegg   phen   49  ykfF  ecocyc   kegg  6.0 0.0 &nbsp &nbsp &nbsp
 &nbsp &nbsp &nbsp  3  Galactose metabolism 0.000 &nbsp &nbsp &nbsp
   yadE   ecocyc   kegg   phen   105  yohL  ecocyc   kegg  13.5 0.0  8  2-Acyl-sn-glycero-3-phosphoethanolamine (n-C18:0)  C05973  4.135
  5  Lysine degradation 0  6  Sphingolipid metabolism 0.001  2  3-Aminoacrylate 0.000 0.000
   yadI   ecocyc   kegg   phen   117  aaeA  ecocyc   kegg  8.5 0.0 &nbsp &nbsp &nbsp
 &nbsp &nbsp &nbsp  3  D-Glutamine and D-glutamate metabolism 0.001 &nbsp &nbsp &nbsp
   yadK   ecocyc   kegg   phen   110  uhpB  ecocyc   kegg  13.2 0.0  7  Adenosine  C00212  4.594
  4  Arachidonic acid metabolism 0  6  Lysine degradation 0.002  2  CTP 0.001 0.000
   yadL   ecocyc   kegg   phen   229  yfeX  ecocyc   kegg  16.1 0.0  7  octadecenoate (n-C18:1)  octadecenoate (n-C18:1)  4.002
  3  Arachidonic acid metabolism 0  2  Sphingolipid metabolism 0.004  12  3-Oxodecanoyl-CoA 0.001 0.000
   yadM   ecocyc   kegg   phen   157  yehD  ecocyc   kegg  8.5 0.0  2  2-hydroxy-6-ketononatrienedioate  C12624  3.481
 &nbsp &nbsp &nbsp  1  Riboflavin metabolism 0.010  4  UDP-D-galacto-1,4-furanose 0.000 0.000
   yadN   ecocyc   kegg   phen   114  ygeR  ecocyc   kegg  8.8 0.0  1  1,2-didodecanoyl-sn-glycerol 3-phosphate  C00416  3.525
 &nbsp &nbsp &nbsp  4  Pantothenate and CoA biosynthesis 0.000  6  Phenylpropanoate 0.000 0.000
   yadS   ecocyc   kegg   phen   91  fkpA  ecocyc   kegg  12.1 0.0  6  2-Oxo-3-hydroxy-4-phosphobutanoate  C06054  3.726
  2  Tryptophan metabolism 0  4  Bacterial secretion system 0.000  4  Bicarbonate 0.000 0.000
   yaeB   ecocyc   kegg   phen   101  yfiQ  ecocyc   kegg  10.5 0.0 &nbsp &nbsp &nbsp
 &nbsp &nbsp &nbsp  3  Amino sugar and nucleotide sugar metabolism 0.001 &nbsp &nbsp &nbsp
   yaeF   ecocyc   kegg   phen   211  yhiK  ecocyc   kegg  12.9 0.0  1  5-Methylthio-D-ribose  C03089  3.861
  1  Arginine and proline metabolism 0  6  Protein export 0.000  13  2-Dehydro-3-deoxy-D-gluconate 0.000 0.000
   yaeH   ecocyc   kegg   phen   47  yeiR  ecocyc   kegg  18.3 0.0  7  Oxalureate  C00802  4.436
  6  Porphyrin and chlorophyll metabolism 9e-06  3  Folate biosynthesis 0.000  1  octanoate (n-C8:0) 0.000 0.000
   yaeI   ecocyc   kegg   phen   170  yaeF  ecocyc   kegg  9.2 0.0  13  1,4-Dihydroxy-2-naphthoate  C03657  3.524
 &nbsp &nbsp &nbsp  2  Lysine biosynthesis 0.001  4  D-Sorbitol 6-phosphate 0.000 1.000
   yaeJ   ecocyc   kegg   phen   79  yaiX  ecocyc   kegg  13.8 0.0  1  Choline  C00114  -4.113
  1  Biosynthesis of secondary metabolites 0  6  Pyrimidine metabolism 0.001  4  5-Phospho-beta-D-ribosylamine 0.000 0.000
   yaeP   ecocyc   kegg   phen   107  yqcC  ecocyc   kegg  9.7 0.0  1  trans-Cinnamate  C00423  3.768
  5  Phenylalanine metabolism 2e-07  1  Biotin metabolism 0.007  8  dTTP 0.000 0.000
   yaeQ   ecocyc   kegg   phen   70  yaeR  ecocyc   kegg  7.0 0.0  1  Guanine  C00242  4.495
  1  Phosphonate and phosphinate metabolism 0  1  Biosynthesis of siderophore group nonribosomal peptides 0.005  1  Uracil 0.001 0.000
   yaeR   ecocyc   kegg   phen   132  gspH  ecocyc   kegg  9.2 0.0  1  Guanine  C00242  3.606
  1  Phosphonate and phosphinate metabolism 0  1  Caprolactam degradation 0.007  9  Crotonoyl-CoA 0.000 0.000
   yafC   ecocyc   kegg   phen   120  zntR  ecocyc   kegg  16.4 0.0  55  cyclopropane phosphatidylethanolamine (dihexadec-9,10-cyclo-anoyl, n-C16:0 cyclo)  cyclopropane phosphatidylethanolamine (dihexadec-9,10-cyclo-anoyl, n-C16:0 cyclo)  4.380
  14  Pyrimidine metabolism 1e-07  5  Oxidative phosphorylation 0.000  7  L-Malate 0.000 0.000
   yafD   ecocyc   kegg   phen   117  ykgL  ecocyc   kegg  8.5 0.0  6  Adenosine  C00212  5.683
  2  Phosphonate and phosphinate metabolism 2e-05  6  Aminoacyl-tRNA biosynthesis 0.001  9  dehydroglycine 0.000 0.000
   yafE   ecocyc   kegg   phen   116  garP  ecocyc   kegg  7.0 0.0  1  2-Acyl-sn-glycero-3-phosphoglycerol (n-C16:0)  2-Acyl-sn-glycero-3-phosphoglycerol (n-C16:0)  3.566
  4  Biosynthesis of secondary metabolites 0.0001  1  Oxidative phosphorylation 0.002  8  D-Glucose 6-phosphate 0.000 0.000
   yafJ   ecocyc   kegg   phen   87  mazG  ecocyc   kegg  11.3 0.0 &nbsp &nbsp &nbsp
 &nbsp &nbsp &nbsp  1  D-Glutamine and D-glutamate metabolism 0.001 &nbsp &nbsp &nbsp
   yafK   ecocyc   kegg   phen   86  ychH  ecocyc   kegg  5.6 0.0  5  2-Dehydro-3-deoxy-D-galactonate  C01216  3.465
 &nbsp &nbsp &nbsp  1  Ribosome 0.000 &nbsp &nbsp &nbsp
   yafL   ecocyc   kegg   phen   70  yafW  ecocyc   kegg  7.0 0.0  2  Hexadecenoate (n-C16:1)  C08362  3.531
 &nbsp &nbsp &nbsp  3  Bisphenol degradation 0.000  1  UDP 0.007 0.000
   yafM   ecocyc   kegg   phen   72  ybgF  ecocyc   kegg  18.6 0.0  6  N1-Acetylspermidine  C00612  3.500
  3  Fatty acid biosynthesis 2e-05  13  Inositol phosphate metabolism 0.000  4  (-)-Ureidoglycolate 0.000 0.000
   yafN   ecocyc   kegg   phen   92  yjiX  ecocyc   kegg  6.7 0.0  6  5-O-(1-Carboxyvinyl)-3-phosphoshikimate  C01269  3.580
 &nbsp &nbsp &nbsp  16  Chlorocyclohexane and chlorobenzene degradation 0.000  7  L-Cystathionine 0.000 0.000
   yafO   ecocyc   kegg   phen   204  ygiL  ecocyc   kegg  12.6 0.0  19  Isopentenyl diphosphate  C00129  4.152
  1  Purine metabolism 3e-06  1  Terpenoid backbone biosynthesis 0.008  30  2-dodecanoyl-sn-glycerol 3-phosphate 0.000 0.000
   yafP   ecocyc   kegg   phen   150  allA  ecocyc   kegg  19.6 0.0  42  Formaldehyde  C00067  8.915
  13  Nicotinate and nicotinamide metabolism 0.0004  1  D-Glutamine and D-glutamate metabolism 0.002  7  O-Acetyl-L-serine 0.000 0.000
   yafS   ecocyc   kegg   phen   87  ycaK  ecocyc   kegg  14.7 0.0  8  Formaldehyde  C00067  4.465
  11  Arginine and proline metabolism 0.0004  1  D-Glutamine and D-glutamate metabolism 0.001  3  O-Acetyl-L-serine 0.000 0.000
   yafT   ecocyc   kegg   phen   177  ypfI  ecocyc   kegg  14.1 0.0  2  octadecanoate (n-C18:0)  C01530  4.359
  2  Arachidonic acid metabolism 0  2  Toluene degradation 0.007  11  dCTP 0.000 0.000
   yafU   ecocyc   kegg   phen   163  holD  ecocyc   kegg  11.5 0.0  5  Hexadecenoate (n-C16:1)  C08362  3.846
 &nbsp &nbsp &nbsp  15  Chlorocyclohexane and chlorobenzene degradation 0.000  32  5-amino-1-(5-phospho-D-ribosyl)imidazole-4-carboxylate 0.000 0.000
   yafV   ecocyc   kegg   phen   148  fadE  ecocyc   kegg  11.8 0.0 &nbsp &nbsp &nbsp
  1  Arachidonic acid metabolism 0  9  Inositol phosphate metabolism 0.002 &nbsp &nbsp &nbsp
   yafX   ecocyc   kegg   phen   148  fadE  ecocyc   kegg  13.0 0.0  3  Adenosine  C00212  4.130
  3  Limonene and pinene degradation 2e-10  7  Bacterial secretion system 0.001  5  3-keto-L-gulonate-6-phosphate 0.000 0.000
   yafY   ecocyc   kegg   phen   142  yjgD  ecocyc   kegg  16.6 0.0  208  O-Phospho-L-serine  C01005  37.505
  24  One carbon pool by folate 0  5  Aminoacyl-tRNA biosynthesis 0.002  13  L-Tryptophan 0.004 1.000
   yafZ   ecocyc   kegg   phen   201  yagB  ecocyc   kegg  15.2 0.0  12  Guanine  C00242  5.709
  3  Phosphonate and phosphinate metabolism 5e-05  7  Pantothenate and CoA biosynthesis 0.000  17  2-Oxobutanoate 0.000 0.000
   yagA   ecocyc   kegg   phen   114  yejH  ecocyc   kegg  8.1 0.0 &nbsp &nbsp &nbsp
  1  Phenylalanine metabolism 0  4  Purine metabolism 0.001 &nbsp &nbsp &nbsp
   yagB   ecocyc   kegg   phen   143  yafZ  ecocyc   kegg  15.2 0.0  12  Formaldehyde  C00067  3.467
  2  Phosphonate and phosphinate metabolism 2e-05  5  Pantothenate and CoA biosynthesis 0.000  10  4-Amino-2-methyl-5-phosphomethylpyrimidine 0.000 0.000
   yagE   ecocyc   kegg   phen   233  yegJ  ecocyc   kegg  7.6 0.0  2  Guanosine  C00387  3.923
 &nbsp &nbsp &nbsp &nbsp &nbsp &nbsp  9  glucosyl-O-acetyl-rhamanosyl-N-acetylglucosamyl-undecaprenyl diphosphate 0.000 0.000
   yagF   ecocyc   kegg   phen   172  ydfJ  ecocyc   kegg  7.6 0.0  16  Pyridoxamine  C00534  3.731
  5  Lipoic acid metabolism 0  1  Mismatch repair 0.004  4  glucosyl-O-acetyl-rhamanosyl-N-acetylglucosamyl-undecaprenyl diphosphate 0.000 0.000
   yagH   ecocyc   kegg   phen   159  fixA  ecocyc   kegg  6.7 0.0 &nbsp &nbsp &nbsp
  1  Biosynthesis of secondary metabolites 0  3  Valine, leucine and isoleucine degradation 0.009 &nbsp &nbsp &nbsp
   yagI   ecocyc   kegg   phen   219  yibF  ecocyc   kegg  9.0 0.0 &nbsp &nbsp &nbsp
 &nbsp &nbsp &nbsp &nbsp &nbsp &nbsp &nbsp &nbsp &nbsp
   yagJ   ecocyc   kegg   phen   75  yaiI  ecocyc   kegg  7.0 0.0  9  Lipoate  C00725  4.114
  4  Limonene and pinene degradation 8e-11  7  Pentose phosphate pathway 0.000  3  D-Glucose 6-phosphate 0.000 0.000
   yagK   ecocyc   kegg   phen   28  yeaO  ecocyc   kegg  32.8 0.0  30  N-Acetylneuraminate  C00270  24.495
  5  alpha-Linolenic acid metabolism 1e-08  2  Inositol phosphate metabolism 0.000  2  Glyceraldehyde 3-phosphate 0.000 0.000
   yagM   ecocyc   kegg   phen   125  rmuC  ecocyc   kegg  17.1 0.0  35  N-Acetyl-L-glutamate  C00624  4.727
  13  Arginine and proline metabolism 5e-07  12  Sulfur metabolism 0.000  8  Adenosine 5'-phosphosulfate 0.000 0.000
   yagN   ecocyc   kegg   phen   70  yjeK  ecocyc   kegg  6.0 0.0 &nbsp &nbsp &nbsp
 &nbsp &nbsp &nbsp  7  Lipopolysaccharide biosynthesis 0.000 &nbsp &nbsp &nbsp
   yagP   ecocyc   kegg   phen   150  yeaB  ecocyc   kegg  14.7 0.0  10  Guanine  C00242  7.431
  7  Cyanoamino acid metabolism 0.0002  6  Chlorocyclohexane and chlorobenzene degradation 0.000  2  D-Glucose 6-phosphate 0.003 0.000
   yagV   ecocyc   kegg   phen   127  yafX  ecocyc   kegg  10.1 0.0  4  p-Cresol  C01468  3.670
 &nbsp &nbsp &nbsp  3  Novobiocin biosynthesis 0.006  7  fructoselysine 0.000 0.000
   yagW   ecocyc   kegg   phen   106  cheY  ecocyc   kegg  9.1 0.0  2  Guanine  C00242  3.619
  1  Phosphonate and phosphinate metabolism 0  5  Toluene degradation 0.000  9  2-Demethylmenaquinone 8 0.000 0.000
   yagX   ecocyc   kegg   phen   237  nadR  ecocyc   kegg  10.6 0.0  7  D-Glucosamine 1-phosphate  C06156  6.869
  2  Caprolactam degradation 0.0002  1  Aminoacyl-tRNA biosynthesis 0.007  9  [4Fe-4S] iron-sulfur cluster 0.001 0.000
   yahB   ecocyc   kegg   phen   93  yjhG  ecocyc   kegg  6.0 0.0  3  N-(5-Phospho-D-ribosyl)anthranilate  C04302  3.648
  2  Xylene degradation 0  4  Chlorocyclohexane and chlorobenzene degradation 0.000  3  Phenylpyruvate 0.000 0.000
   yahC   ecocyc   kegg   phen   432  yjeK  ecocyc   kegg  14.0 0.0  10  Glycerophosphoserine  Glycerophosphoserine  3.903
  1  Nicotinate and nicotinamide metabolism 0  13  Chlorocyclohexane and chlorobenzene degradation 0.000  38  2-Octaprenyl-6-methoxyphenol 0.000 0.000
   yahE   ecocyc   kegg   phen   98  clpA  ecocyc   kegg  14.2 0.0  6  Hexadecenoate (n-C16:1)  C08362  4.390
 &nbsp &nbsp &nbsp  1  Two-component system 0.001  3  Putrescine 0.001 0.000
   yahF   ecocyc   kegg   phen   105  ydbA  ecocyc   kegg  7.4 0.0 &nbsp &nbsp &nbsp
  1  Biosynthesis of secondary metabolites 0  8  Fatty acid biosynthesis 0.001 &nbsp &nbsp &nbsp
   yahG   ecocyc   kegg   phen   113  yafZ  ecocyc   kegg  11.7 0.0  21  Farnesyl diphosphate  C00448  4.775
  7  C5-Branched dibasic acid metabolism 5e-09  3  Glycolysis / Gluconeogenesis 0.000  3  L-Tryptophan 0.001 0.000
   yahH   ecocyc   kegg   phen   126  ymdF  ecocyc   kegg  7.5 0.0 &nbsp &nbsp &nbsp
 &nbsp &nbsp &nbsp  3  Phosphonate and phosphinate metabolism 0.001 &nbsp &nbsp &nbsp
   yahI   ecocyc   kegg   phen   131  gabD  ecocyc   kegg  22.7 0.0  50  7-cyano-7-carbaguanine  C15996  4.324
  7  D-Glutamine and D-glutamate metabolism 4e-10  9  Taurine and hypotaurine metabolism 0.001  6  2,3-dehydroadipyl-CoA 0.000 0.000
   yahK   ecocyc   kegg   phen   350  yjeK  ecocyc   kegg  9.8 0.0  28  N(omega)-(L-Arginino)succinate  C03406  5.628
  10  Fructose and mannose metabolism 5e-06  16  Chlorocyclohexane and chlorobenzene degradation 0.000  21  5-amino-1-(5-phospho-D-ribosyl)imidazole-4-carboxylate 0.000 0.000
   yahL   ecocyc   kegg   phen   178  rnt  ecocyc   kegg  17.6 0.0  51  phosphatidylethanolamine (ditetradecanoyl, n-C14:0)  C00350  4.630
  17  Arachidonic acid metabolism 0  4  Two-component system 0.000  16  silver 0.000 0.000
   yahM   ecocyc   kegg   phen   155  nmpC  ecocyc   kegg  9.0 0.0 &nbsp &nbsp &nbsp
 &nbsp &nbsp &nbsp  2  Bisphenol degradation 0.002 &nbsp &nbsp &nbsp
   yaiA   ecocyc   kegg   phen   110  hslV  ecocyc   kegg  8.3 0.0  2  Propanal  C00479  4.342
  2  Ubiquinone and other terpenoid-quinone biosynthesis 0  4  Two-component system 0.000  9  D-Glycerate 2-phosphate 0.000 0.000
   yaiE   ecocyc   kegg   phen   87  ybdD  ecocyc   kegg  11.5 0.0  5  1,2-dihexadecanoyl-sn-glycerol 3-phosphate  C00416  4.216
 &nbsp &nbsp &nbsp  10  Oxidative phosphorylation 0.000  6  2-Demethylmenaquinone 8 0.000 0.000
   yaiF   ecocyc   kegg   phen   189  ysgA  ecocyc   kegg  6.7 0.0 &nbsp &nbsp &nbsp
 &nbsp &nbsp &nbsp  5  Chlorocyclohexane and chlorobenzene degradation 0.000 &nbsp &nbsp &nbsp
   yaiI   ecocyc   kegg   phen   320  ygjV  ecocyc   kegg  16.0 0.0  49  cAMP  C00575  5.159
  7  Purine metabolism 2e-12 &nbsp &nbsp &nbsp  15  Thiamin monophosphate 0.000 0.000
   yaiO   ecocyc   kegg   phen   83  yddL  ecocyc   kegg  9.8 0.0  6  L-Glutamate 5-semialdehyde  C01165  5.455
  7  Arginine and proline metabolism 7e-06  9  Arachidonic acid metabolism 0.000  3  2-Oxobutanoate 0.000 0.000
   yaiP   ecocyc   kegg   phen   121  ybhC  ecocyc   kegg  14.9 0.0  12  Formaldehyde  C00067  5.644
 &nbsp &nbsp &nbsp  8  Pyruvate metabolism 0.001  5  (-)-Ureidoglycolate 0.000 0.000
   yaiS   ecocyc   kegg   phen   126  yegE  ecocyc   kegg  14.2 0.0  21  Dihydropteroate  C00921  6.333
  3  Arachidonic acid metabolism 0 &nbsp &nbsp &nbsp  3  Sulfite 0.002 0.000
   yaiT   ecocyc   kegg   phen   153  ydeH  ecocyc   kegg  7.6 0.0  1  1,2-dihexadecanoyl-sn-glycerol 3-phosphate  C00416  4.327
 &nbsp &nbsp &nbsp &nbsp &nbsp &nbsp  4  glucosyl-O-acetyl-rhamanosyl-N-acetylglucosamyl-undecaprenyl diphosphate 0.000 0.000
   yaiU   ecocyc   kegg   phen   186  ymdF  ecocyc   kegg  8.6 0.0 &nbsp &nbsp &nbsp
 &nbsp &nbsp &nbsp  1  Phosphonate and phosphinate metabolism 0.002 &nbsp &nbsp &nbsp
   yaiV   ecocyc   kegg   phen   62  rdgC  ecocyc   kegg  5.7 0.0 &nbsp &nbsp &nbsp
 &nbsp &nbsp &nbsp  1  Vitamin B6 metabolism 0.007 &nbsp &nbsp &nbsp
   yaiW   ecocyc   kegg   phen   50  cusF  ecocyc   kegg  27.4 0.0  21  Bicarbonate  C00288  6.886
  9  Tryptophan metabolism 9e-05  10  Chloroalkane and chloroalkene degradation 0.001  1  L-Malate 0.004 0.000
   yaiY   ecocyc   kegg   phen   81  yohM  ecocyc   kegg  14.5 0.0  52  2-C-methyl-D-erythritol 2,4-cyclodiphosphate  C11453  8.393
  8  C5-Branched dibasic acid metabolism 7e-14  4  Biosynthesis of siderophore group nonribosomal peptides 0.000  10  2,3-Dihydro-2,3-dihydroxybenzoate 0.000 0.000
   yaiZ   ecocyc   kegg   phen   102  ydaU  ecocyc   kegg  14.7 0.0  8  Hypoxanthine  C00262  7.754
  17  Caprolactam degradation 5e-06  1  Biotin metabolism 0.007  6  O-Acetyl-L-serine 0.000 0.000
   yajD   ecocyc   kegg   phen   131  yagY  ecocyc   kegg  15.9 0.0  58  2-Acyl-sn-glycero-3-phosphoethanolamine (n-C18:0)  C05973  4.541
  17  Lipoic acid metabolism 0  9  Oxidative phosphorylation 0.000  2  2-Demethylmenaquinol 8 0.000 0.000
   yajI   ecocyc   kegg   phen   113  ynfP  ecocyc   kegg  8.5 0.0 &nbsp &nbsp &nbsp
 &nbsp &nbsp &nbsp  3  Fructose and mannose metabolism 0.003 &nbsp &nbsp &nbsp
   yajL   ecocyc   kegg   phen   192  sfmH  ecocyc   kegg  10.2 0.0 &nbsp &nbsp &nbsp
 &nbsp &nbsp &nbsp  1  Riboflavin metabolism 0.000 &nbsp &nbsp &nbsp
   yajO   ecocyc   kegg   phen   326  ypdG  ecocyc   kegg  8.2 0.0  12  cyclic pyranopterin monophosphate  C18239  3.688
  6  Arachidonic acid metabolism 6e-07  10  Chlorocyclohexane and chlorobenzene degradation 0.000  17  (S)-3-Methyl-2-oxopentanoate 0.000 0.000
   yajQ   ecocyc   kegg   phen   111  yajR  ecocyc   kegg  8.7 0.0 &nbsp &nbsp &nbsp
 &nbsp &nbsp &nbsp  4  Phosphonate and phosphinate metabolism 0.001 &nbsp &nbsp &nbsp
   yajR   ecocyc   kegg   phen   153  yajQ  ecocyc   kegg  8.7 0.0 &nbsp &nbsp &nbsp
 &nbsp &nbsp &nbsp  2  Phosphonate and phosphinate metabolism 0.002 &nbsp &nbsp &nbsp
   ybaE   ecocyc   kegg   phen   80  ybeX  ecocyc   kegg  11.0 0.0 &nbsp &nbsp &nbsp
 &nbsp &nbsp &nbsp  3  Cyanoamino acid metabolism 0.006 &nbsp &nbsp &nbsp
   ybaL   ecocyc   kegg   phen   50  ybeX  ecocyc   kegg  22.8 0.0  1  cis-3-(3-carboxyethyl)-3,5-cyclohexadiene-1,2-diol  cis-3-(3-carboxyethyl)-3,5-cyclohexadiene-1,2-diol  4.569
  4  Ethylbenzene degradation 0.0003  1  Protein export 0.003 &nbsp &nbsp &nbsp
   ybaM   ecocyc   kegg   phen   90  dsbC  ecocyc   kegg  8.3 0.0  3  2-Oxo-3-hydroxy-4-phosphobutanoate  C06054  3.490
 &nbsp &nbsp &nbsp  2  Two-component system 0.003 &nbsp &nbsp &nbsp
   ybaN   ecocyc   kegg   phen   91  cheY  ecocyc   kegg  9.0 0.0  2  XDP  C01337  3.707
 &nbsp &nbsp &nbsp  5  Two-component system 0.000  4  2-Demethylmenaquinone 8 0.000 0.000
   ybaO   ecocyc   kegg   phen   90  yrfC  ecocyc   kegg  7.1 0.0  4  2-Dehydro-3-deoxy-D-galactonate 6-phosphate  C01286  3.616
 &nbsp &nbsp &nbsp  6  Valine, leucine and isoleucine biosynthesis 0.001  10  L-Methionine Sulfoxide 0.000 0.000
   ybaP   ecocyc   kegg   phen   169  ybjR  ecocyc   kegg  15.5 0.0  31  trans-Aconitate  C02341  3.927
  8  C5-Branched dibasic acid metabolism 2e-15  7  Two-component system 0.000  16  Citrate 0.006 1.000
   ybaQ   ecocyc   kegg   phen   116  hslJ  ecocyc   kegg  14.7 0.0  19  (R)-Pantothenate  C00864  6.787
  18  D-Alanine metabolism 1e-06  15  Citrate cycle (TCA cycle) 0.000  10  Propionate (n-C3:0) 0.000 0.000
   ybaT   ecocyc   kegg   phen   93  leuD  ecocyc   kegg  6.9 0.0  2  Thiosulfate  C00320  6.643
 &nbsp &nbsp &nbsp  10  Bisphenol degradation 0.001  4  Anthranilate 0.000 0.000
   ybaV   ecocyc   kegg   phen   126  flgK  ecocyc   kegg  7.8 0.0  5  L-Glutamate 5-phosphate  C03287  4.250
  3  Lysine biosynthesis 0.004  8  Valine, leucine and isoleucine biosynthesis 0.000  18  dGTP 0.000 0.000
   ybaY   ecocyc   kegg   phen   64  mppA  ecocyc   kegg  11.8 0.0  1  S-Adenosyl-L-methionine  C00019  3.543
 &nbsp &nbsp &nbsp  5  D-Glutamine and D-glutamate metabolism 0.000  6  L-Histidine 0.000 0.000
   ybbC   ecocyc   kegg   phen   212  mviM  ecocyc   kegg  8.0 0.0  5  Trehalose  C01083  5.749
  10  C5-Branched dibasic acid metabolism 1e-09 &nbsp &nbsp &nbsp  5  glucosyl-O-acetyl-rhamanosyl-N-acetylglucosamyl-undecaprenyl diphosphate 0.000 0.000
   ybbD   ecocyc   kegg   phen   81  ybcQ  ecocyc   kegg  9.3 0.0  1  UDP-4-keto-pyranose  C16155  4.159
 &nbsp &nbsp &nbsp  3  Two-component system 0.000  2  Undecaprenyl phosphate 0.000 0.000
   ybbL   ecocyc   kegg   phen   55  ygcB  ecocyc   kegg  7.0 0.0  7  Thiosulfate  C00320  6.159
  2  Arachidonic acid metabolism 4e-06  4  Alanine, aspartate and glutamate metabolism 0.000  7  3-Carboxy-3-hydroxy-4-methylpentanoate 0.000 0.000
   ybbM   ecocyc   kegg   phen   190  ybiN  ecocyc   kegg  10.8 0.0 &nbsp &nbsp &nbsp
 &nbsp &nbsp &nbsp  2  Glycerolipid metabolism 0.002 &nbsp &nbsp &nbsp
   ybbN   ecocyc   kegg   phen   447  kdpB  ecocyc   kegg  11.4 0.0  8  d-biotin d-sulfoxide  d-biotin d-sulfoxide  3.478
  1  Lysine degradation 9e-05  5  Chlorocyclohexane and chlorobenzene degradation 0.000  29  (S)-3-Methyl-2-oxopentanoate 0.000 0.000
   ybbO   ecocyc   kegg   phen   159  ynhG  ecocyc   kegg  17.5 0.0  16  2-Acyl-sn-glycero-3-phosphoethanolamine (n-C16:1)  C05973  4.741
  2  Biosynthesis of unsaturated fatty acids 4e-05  2  Lysine degradation 0.007  5  L-Leucine 0.002 0.000
   ybbV   ecocyc   kegg   phen   83  fhuE  ecocyc   kegg  17.4 0.0  22  L-tartrate  C00898  9.596
  12  Arginine and proline metabolism 0.0006  1  D-Glutamine and D-glutamate metabolism 0.000  2  O-Acetyl-L-serine 0.000 0.000
   ybbW   ecocyc   kegg   phen   115  uvrC  ecocyc   kegg  12.8 0.0 &nbsp &nbsp &nbsp
  3  Limonene and pinene degradation 2e-10  4  ABC transporters 0.000 &nbsp &nbsp &nbsp
   ybbY   ecocyc   kegg   phen   154  yafQ  ecocyc   kegg  12.2 0.0  1  1,2-Diacyl-sn-glycerol (dioctadecanoyl, n-C18:0)  C00641  4.642
  3  Biosynthesis of secondary metabolites 0  8  Geraniol degradation 0.001  15  Acetoacetyl-CoA 0.000 0.000
   ybcC   ecocyc   kegg   phen   158  frmB  ecocyc   kegg  11.7 0.0  47  myo-Inositol  C00137  4.162
  20  Alanine, aspartate and glutamate metabolism 9e-05  3  Aminoacyl-tRNA biosynthesis 0.000  7  L-Lysine-tRNA (Lys) 0.000 0.000
   ybcF   ecocyc   kegg   phen   76  gloA  ecocyc   kegg  9.1 0.0  4  (R)-S-Lactoylglutathione  C03451  4.737
  1  Pyruvate metabolism 4e-05  16  Arachidonic acid metabolism 0.000  2  UDPglucose 0.001 0.000
   ybcH   ecocyc   kegg   phen   89  yliI  ecocyc   kegg  12.7 0.0  22  crotonobetaine  C04114  5.034
  9  Biosynthesis of secondary metabolites 7e-08  17  Glycolysis / Gluconeogenesis 0.000  5  [2Fe-1S] desulfurated iron-sulfur cluster 0.000 0.000
   ybcI   ecocyc   kegg   phen   96  pps  ecocyc   kegg  10.1 0.0  1  gamma-butyrobetaine  C01181  5.324
 &nbsp &nbsp &nbsp  3  Two-component system 0.004  1  Oxaloacetate 0.001 0.000
   ybcJ   ecocyc   kegg   phen   101  ybbM  ecocyc   kegg  7.2 0.0 &nbsp &nbsp &nbsp
 &nbsp &nbsp &nbsp  2  Phosphonate and phosphinate metabolism 0.001 &nbsp &nbsp &nbsp
   ybcL   ecocyc   kegg   phen   191  rem  ecocyc   kegg  9.8 0.0  35  Pyridoxamine  C00534  5.051
  14  Lipoic acid metabolism 0  1  Mismatch repair 0.006  11  CMP 0.005 1.000
   ybcM   ecocyc   kegg   phen   88  ybgL  ecocyc   kegg  13.5 0.0  5  3-(3-hydroxy-phenyl)propionate  C11457  3.594
  10  Phenylalanine, tyrosine and tryptophan biosynthesis 5e-06  11  Toluene degradation 0.001  3  Succinate 0.000 0.000
   ybcN   ecocyc   kegg   phen   194  gspO  ecocyc   kegg  11.4 0.0  7  5'-Deoxyadenosine  C05198  3.905
  5  Ubiquinone and other terpenoid-quinone biosynthesis 0.001  11  Chlorocyclohexane and chlorobenzene degradation 0.000  12  2-Oxobutanoate 0.000 0.000
   ybcO   ecocyc   kegg   phen   99  atoS  ecocyc   kegg  8.1 0.0 &nbsp &nbsp &nbsp
  2  Caprolactam degradation 3e-05  2  Biotin metabolism 0.006 &nbsp &nbsp &nbsp
   ybcV   ecocyc   kegg   phen   107  ydaU  ecocyc   kegg  15.1 0.0  33  Hypoxanthine  C00262  13.157
  18  Microbial metabolism in diverse environments 3e-08  1  Pyruvate metabolism 0.004  7  L-Cysteine 0.001 1.000
   ybcW   ecocyc   kegg   phen   76  ydfI  ecocyc   kegg  9.3 0.0  13  UDP-D-galacto-1,4-furanose  C03733  4.129
  10  Lysine degradation 9e-10  4  Other glycan degradation 0.000  2  ADP-L-glycero-D-manno-heptose 0.000 0.000
   ybcY   ecocyc   kegg   phen   70  ymgB  ecocyc   kegg  7.0 0.0 &nbsp &nbsp &nbsp
 &nbsp &nbsp &nbsp  2  Pyrimidine metabolism 0.004 &nbsp &nbsp &nbsp
   ybdD   ecocyc   kegg   phen   171  radA  ecocyc   kegg  13.5 0.0  31  2-Acyl-sn-glycero-3-phosphoethanolamine (n-C18:0)  C05973  5.643
  8  Phosphotransferase system (PTS) 2e-07  15  Glycolysis / Gluconeogenesis 0.000  9  D-Glycerate 2-phosphate 0.000 0.000
   ybdF   ecocyc   kegg   phen   146  ycbF  ecocyc   kegg  8.9 0.0 &nbsp &nbsp &nbsp
 &nbsp &nbsp &nbsp  1  Glycerolipid metabolism 0.001 &nbsp &nbsp &nbsp
   ybdH   ecocyc   kegg   phen   164  yedJ  ecocyc   kegg  11.5 0.0  47  D-Glycerate 2-phosphate  C00631  4.899
  17  Oxidative phosphorylation 6e-06  3  Inositol phosphate metabolism 0.002  2  D-Gluconate 0.001 0.000
   ybdJ   ecocyc   kegg   phen   84  yiaU  ecocyc   kegg  11.7 0.0  7  L-Lysine  C00047  -3.521
  1  Thiamine metabolism 0  5  Bacterial secretion system 0.001  1  Oxaloacetate 0.001 0.000
   ybdM   ecocyc   kegg   phen   100  poxB  ecocyc   kegg  12.7 0.0  15  Urate  C00366  3.787
 &nbsp &nbsp &nbsp  4  Oxidative phosphorylation 0.000  4  D-Glycerate 2-phosphate 0.000 0.000
   ybdN   ecocyc   kegg   phen   71  grxA  ecocyc   kegg  19.4 0.0  78  o-Succinylbenzoate  C02730  11.966
  14  Microbial metabolism in diverse environments 4e-05  6  Sphingolipid metabolism 0.000  11  UDP 0.000 0.000
   ybdO   ecocyc   kegg   phen   151  cyoD  ecocyc   kegg  17.4 0.0  86  8-Amino-7-oxononanoate  C01092  5.586
  18  Purine metabolism 3e-08  6  Oxidative phosphorylation 0.000  4  Succinate 0.000 1.000
   ybdR   ecocyc   kegg   phen   226  holD  ecocyc   kegg  13.2 0.0  1  Thiocyanate  C01755  3.499
  1  Cysteine and methionine metabolism 0  12  Chlorocyclohexane and chlorobenzene degradation 0.000  23  GDP-4-dehydro-6-deoxy-D-mannose 0.000 0.000
   ybdZ   ecocyc   kegg   phen   105  sufA  ecocyc   kegg  13.4 0.0  33  D-Glucosamine 1-phosphate  C06156  4.441
  17  C5-Branched dibasic acid metabolism 3e-08  6  Chlorocyclohexane and chlorobenzene degradation 0.000  3  Sulfite 0.004 0.000
   ybeB   ecocyc   kegg   phen   130  ymdC  ecocyc   kegg  8.5 0.0 &nbsp &nbsp &nbsp
 &nbsp &nbsp &nbsp &nbsp &nbsp &nbsp &nbsp &nbsp &nbsp
   ybeD   ecocyc   kegg   phen   88  ybeX  ecocyc   kegg  12.8 0.0  5  N1-Acetylspermidine  C00612  10.748
  2  Lysine degradation 0.0006  6  Fatty acid biosynthesis 0.001  4  [2Fe-1S] desulfurated iron-sulfur cluster 0.000 0.000
   ybeF   ecocyc   kegg   phen   79  cyoB  ecocyc   kegg  10.7 0.0  25  1,2-dihexadecanoyl-sn-glycerol 3-phosphate  C00416  4.147
  9  Butanoate metabolism 7e-08  3  Oxidative phosphorylation 0.000  3  Succinyl-CoA 0.000 0.000
   ybeH   ecocyc   kegg   phen   210  rem  ecocyc   kegg  8.0 0.0  6  L-Phenylalanine  C00079  3.601
  4  Riboflavin metabolism 4e-05  2  Base excision repair 0.004  13  glucosyl-O-acetyl-rhamanosyl-N-acetylglucosamyl-undecaprenyl diphosphate 0.000 0.000
   ybeL   ecocyc   kegg   phen   87  yfeD  ecocyc   kegg  9.9 0.0 &nbsp &nbsp &nbsp
  3  Limonene and pinene degradation 4e-13  10  Glycolysis / Gluconeogenesis 0.001 &nbsp &nbsp &nbsp
   ybeM   ecocyc   kegg   phen   118  yahA  ecocyc   kegg  11.3 0.0 &nbsp &nbsp &nbsp
  4  Toluene degradation 0 &nbsp &nbsp &nbsp &nbsp &nbsp &nbsp
   ybeQ   ecocyc   kegg   phen   170  holD  ecocyc   kegg  11.2 0.0  4  Pyridoxamine  C00534  3.818
 &nbsp &nbsp &nbsp  7  C5-Branched dibasic acid metabolism 0.000  8  ITP 0.000 0.000
   ybeR   ecocyc   kegg   phen   67  ybfQ  ecocyc   kegg  10.2 0.0  11  1,2-Diacyl-sn-glycerol (dioctadec-11-enoyl, n-C18:1)  C00641  4.691
  6  Terpenoid backbone biosynthesis 0.001  9  Microbial metabolism in diverse environments 0.000  9  Isocitrate 0.000 0.000
   ybeT   ecocyc   kegg   phen   246  bioA  ecocyc   kegg  16.3 0.0  65  cyclopropane phosphatidylglycerol (dihexadec-9,10-cyclo-anoyl, n-C16:0 cyclo)  cyclopropane phosphatidylglycerol (dihexadec-9,10-cyclo-anoyl, n-C16:0 cyclo)  6.328
  7  Fatty acid biosynthesis 1e-07  8  RNA polymerase 0.000  12  7,8-Diaminononanoate 0.000 0.000
   ybeX   ecocyc   kegg   phen   61  lomR  ecocyc   kegg  55.9 0.0  61  p-Cresol  C01468  27.035
  11  Arachidonic acid metabolism 0  1  Protein export 0.005  2  D-Glucose 6-phosphate 0.001 0.000
   ybeZ   ecocyc   kegg   phen   135  hokA  ecocyc   kegg  9.4 0.0  3  Superoxide anion  C00704  3.626
  2  Terpenoid backbone biosynthesis 0.0003 &nbsp &nbsp &nbsp  10  L-Citrulline 0.000 0.000
   ybfA   ecocyc   kegg   phen   166  ydfW  ecocyc   kegg  10.4 0.0  2  L-Glutamine  C00064  3.996
  10  D-Glutamine and D-glutamate metabolism 2e-08  4  Riboflavin metabolism 0.000  7  Orotidine 5'-phosphate 0.000 0.000
   ybfB   ecocyc   kegg   phen   133  cusF  ecocyc   kegg  21.8 0.0  38  2-Acyl-sn-glycero-3-phosphoethanolamine (n-C16:1)  C05973  6.129
  12  C5-Branched dibasic acid metabolism 6e-10  6  Chlorocyclohexane and chlorobenzene degradation 0.000  4  D-Glycerate 2-phosphate 0.000 0.000
   ybfC   ecocyc   kegg   phen   99  pflA  ecocyc   kegg  9.1 0.0  6  Pyridoxal 5'-phosphate  C00018  3.498
 &nbsp &nbsp &nbsp  2  Bacterial secretion system 0.002  3  Propanoyl-CoA 0.000 0.000
   ybfD   ecocyc   kegg   phen   238  nadR  ecocyc   kegg  9.2 0.0  1  crotonobetaine  C04114  4.446
  2  Caprolactam degradation 0.0002 &nbsp &nbsp &nbsp  3  L-fuculose 0.000 0.000
   ybfG   ecocyc   kegg   phen   106  ymdF  ecocyc   kegg  8.4 0.0 &nbsp &nbsp &nbsp
 &nbsp &nbsp &nbsp  1  Other glycan degradation 0.002 &nbsp &nbsp &nbsp
   ybfH   ecocyc   kegg   phen   205  ybiO  ecocyc   kegg  9.2 0.0 &nbsp &nbsp &nbsp
 &nbsp &nbsp &nbsp  1  Glycerolipid metabolism 0.000 &nbsp &nbsp &nbsp
   ybfO   ecocyc   kegg   phen   149  yqeJ  ecocyc   kegg  15.9 0.0  22  Formaldehyde  C00067  4.718
  10  beta-Alanine metabolism 0.0003  2  Aminoacyl-tRNA biosynthesis 0.002  2  D-Fructose 1,6-bisphosphate 0.000 0.000
   ybfP   ecocyc   kegg   phen   95  yegE  ecocyc   kegg  10.4 0.0  2  Hydroxypyruvate  C00168  3.737
 &nbsp &nbsp &nbsp  1  Novobiocin biosynthesis 0.004  1  Uracil 0.000 0.000
   ybfQ   ecocyc   kegg   phen   111  ymjA  ecocyc   kegg  10.7 0.0  15  2-Octaprenyl-6-hydroxyphenol  C05811  4.008
  13  Microbial metabolism in diverse environments 5e-09  6  Pyruvate metabolism 0.004  3  D-Glycerate 2-phosphate 0.000 0.000
   ybgA   ecocyc   kegg   phen   250  yahC  ecocyc   kegg  10.5 0.0  1  lipoyl-AMP  C16238  -3.544
 &nbsp &nbsp &nbsp  18  Chlorocyclohexane and chlorobenzene degradation 0.000  19  Dephospho-CoA 0.000 0.000
   ybgD   ecocyc   kegg   phen   191  yidK  ecocyc   kegg  13.7 0.0  39  UDPgalactose  C00052  4.520
  15  Lysine degradation 9e-07  13  Methane metabolism 0.000  16  myo-Inositol 0.000 0.000
   ybgE   ecocyc   kegg   phen   148  tktB  ecocyc   kegg  8.7 0.0  14  E-3-carboxy-2-pentenedioate 6-methyl ester  C11514  4.320
 &nbsp &nbsp &nbsp  5  Two-component system 0.000  7  D-Glycerate 2-phosphate 0.000 0.000
   ybgK   ecocyc   kegg   phen   60  lomR  ecocyc   kegg  22.1 0.0 &nbsp &nbsp &nbsp
  3  Limonene and pinene degradation 2e-06  3  Polyketide sugar unit biosynthesis 0.004 &nbsp &nbsp &nbsp
   ybgL   ecocyc   kegg   phen   184  ycbC  ecocyc   kegg  15.5 0.0  14  4-Hydroxy-L-threonine  C06056  -3.526
  2  Purine metabolism 3e-06  12  RNA polymerase 0.000  6  7,8-Diaminononanoate 0.000 0.000
   ybgO   ecocyc   kegg   phen   156  ymdF  ecocyc   kegg  8.4 0.0 &nbsp &nbsp &nbsp
 &nbsp &nbsp &nbsp &nbsp &nbsp &nbsp &nbsp &nbsp &nbsp
   ybgP   ecocyc   kegg   phen   96  yagY  ecocyc   kegg  8.9 0.0  6  N-Acetyl-L-glutamate  C00624  4.682
  3  Terpenoid backbone biosynthesis 0.001  5  Phosphonate and phosphinate metabolism 0.001  6  Guanosine 3'-diphosphate 5'-triphosphate 0.000 0.000
   ybgQ   ecocyc   kegg   phen   104  yceP  ecocyc   kegg  8.6 0.0 &nbsp &nbsp &nbsp
 &nbsp &nbsp &nbsp &nbsp &nbsp &nbsp &nbsp &nbsp &nbsp
   ybgS   ecocyc   kegg   phen   117  yaiS  ecocyc   kegg  9.6 0.0  8  cyclopropane phosphatidylethanolamine (dihexadec-9,10-cyclo-anoyl, n-C16:0 cyclo)  cyclopropane phosphatidylethanolamine (dihexadec-9,10-cyclo-anoyl, n-C16:0 cyclo)  4.003
 &nbsp &nbsp &nbsp  5  Lysine degradation 0.003  2  Cu+ 0.000 0.000
   ybgT   ecocyc   kegg   phen   130  iscA  ecocyc   kegg  17.4 0.0  30  N1-Acetylspermidine  C00612  5.214
  20  Aminoacyl-tRNA biosynthesis 3e-07  21  Butanoate metabolism 0.000  22  L-Proline 0.007 1.000
   ybhB   ecocyc   kegg   phen   127  ychJ  ecocyc   kegg  16.7 0.0  31  2-C-methyl-D-erythritol 2,4-cyclodiphosphate  C11453  4.035
  5  Fatty acid biosynthesis 2e-07  9  Chlorocyclohexane and chlorobenzene degradation 0.000  5  MoaD Protein with carboxylate 0.000 0.000
   ybhC   ecocyc   kegg   phen   111  yaiP  ecocyc   kegg  14.9 0.0  25  Formaldehyde  C00067  4.954
  10  Arginine and proline metabolism 1e-05  11  Pyruvate metabolism 0.001  10  Sedoheptulose 7-phosphate 0.000 0.000
   ybhD   ecocyc   kegg   phen   108  yciV  ecocyc   kegg  11.6 0.0  26  Hydroxypyruvate  C00168  4.233
  9  Glycerolipid metabolism 8e-06  12  Nitrotoluene degradation 0.000  10  Decanoyl-ACP (n-C10:0ACP) 0.000 0.000
   ybhG   ecocyc   kegg   phen   52  yaiX  ecocyc   kegg  15.1 0.0  1  p-Cresol  C01468  -4.588
  1  Toluene degradation 8e-05 &nbsp &nbsp &nbsp &nbsp &nbsp &nbsp
   ybhI   ecocyc   kegg   phen   100  ycaK  ecocyc   kegg  11.3 0.0  3  Formaldehyde  C00067  3.670
 &nbsp &nbsp &nbsp  1  D-Glutamine and D-glutamate metabolism 0.001  2  alpha-D-Ribose 5-phosphate 0.000 0.000
   ybhJ   ecocyc   kegg   phen   42  yciQ  ecocyc   kegg  28.9 0.0  26  tetradecenoate (n-C14:1)  tetradecenoate (n-C14:1)  15.343
  12  Limonene and pinene degradation 0  7  Terpenoid backbone biosynthesis 0.000 &nbsp &nbsp &nbsp
   ybhK   ecocyc   kegg   phen   77  ygeA  ecocyc   kegg  13.3 0.0  33  trans-Aconitate  C02341  4.417
  3  Arachidonic acid metabolism 0  1  Protein export 0.008  2  Arbutin 6-phosphate 0.000 0.000
   ybhL   ecocyc   kegg   phen   90  btuB  ecocyc   kegg  11.3 0.0  10  Thiosulfate  C00320  4.739
  1  Histidine metabolism 0  6  Arachidonic acid metabolism 0.001  7  [4Fe-4S] iron-sulfur cluster 0.001 0.000
   ybhP   ecocyc   kegg   phen   34  folM  ecocyc   kegg  40.2 0.0  166  7-aminomethyl-7-deazaguanine  C01449  46.308
  11  Lysine degradation 9e-05  10  Bacterial chemotaxis 0.001  3  CTP 0.000 0.000
   ybhQ   ecocyc   kegg   phen   211  yfjW  ecocyc   kegg  9.7 0.0  5  5,10-Methenyltetrahydrofolate  C00445  5.196
  1  Lysine biosynthesis 0.0009  1  Mismatch repair 0.008  9  glucosyl-O-acetyl-rhamanosyl-N-acetylglucosamyl-undecaprenyl diphosphate 0.000 0.000
   ybhT   ecocyc   kegg   phen   103  rsgA  ecocyc   kegg  9.8 0.0 &nbsp &nbsp &nbsp
 &nbsp &nbsp &nbsp  16  Chlorocyclohexane and chlorobenzene degradation 0.000 &nbsp &nbsp &nbsp
   ybiA   ecocyc   kegg   phen   143  yajD  ecocyc   kegg  13.5 0.0  30  2-C-methyl-D-erythritol 2,4-cyclodiphosphate  C11453  5.475
  8  Arginine and proline metabolism 7e-05  6  D-Glutamine and D-glutamate metabolism 0.001  3  (-)-Ureidoglycolate 0.000 0.000
   ybiC   ecocyc   kegg   phen   126  allD  ecocyc   kegg  11.9 0.0  6  3-Methyl-2-oxobutanoate  C00141  6.152
  13  Toluene degradation 4e-05  6  Ascorbate and aldarate metabolism 0.000  13  ADP-L-glycero-D-manno-heptose 0.000 0.000
   ybiH   ecocyc   kegg   phen   69  ugpB  ecocyc   kegg  22.8 0.0  41  Thiosulfate  C00320  7.777
  9  Butanoate metabolism 0.0002  3  ABC transporters 0.000  12  D-Glucose 6-phosphate 0.005 1.000
   ybiI   ecocyc   kegg   phen   76  ydhF  ecocyc   kegg  9.5 0.0  6  (R)-Pantothenate  C00864  4.505
  8  beta-Alanine metabolism 5e-06  14  Chloroalkane and chloroalkene degradation 0.001  1  5-Amino-1-(5-Phospho-D-ribosyl)imidazole-4-carboxamide 0.000 0.000
   ybiJ   ecocyc   kegg   phen   93  fimE  ecocyc   kegg  9.7 0.0  4  1,2-Diacyl-sn-glycerol (dioctadec-11-enoyl, n-C18:1)  C00641  4.697
  5  Lysine degradation 5e-10  13  Toluene degradation 0.000  8  cis-tetradec-7-enoyl-[acyl-carrier protein] (n-C14:1) 0.000 0.000
   ybiO   ecocyc   kegg   phen   246  puuA  ecocyc   kegg  10.1 0.0  3  Hexanoate (n-C6:0)  C01585  3.741
  9  Caprolactam degradation 3e-07  5  Limonene and pinene degradation 0.002  15  Dihydroxyacetone 0.000 0.000
   ybiP   ecocyc   kegg   phen   57  ydjJ  ecocyc   kegg  17.5 0.0  4  octadecanoate (n-C18:0)  C01530  3.613
  1  Biosynthesis of secondary metabolites 0  1  Riboflavin metabolism 0.001 &nbsp &nbsp &nbsp
   ybiR   ecocyc   kegg   phen   203  ynhG  ecocyc   kegg  11.7 0.0  22  O-Acetyl-L-serine  C00979  5.556
  15  D-Glutamine and D-glutamate metabolism 2e-05  1  Phosphonate and phosphinate metabolism 0.003  11  glucosyl-O-acetyl-rhamanosyl-N-acetylglucosamyl-undecaprenyl diphosphate 0.000 0.000
   ybiS   ecocyc   kegg   phen   152  ygjH  ecocyc   kegg  9.7 0.0  2  octadecanoate (n-C18:0)  C01530  -4.251
  3  alpha-Linolenic acid metabolism 4e-07  8  alpha-Linolenic acid metabolism 0.000  4  D-Glucose 0.003 0.000
   ybiT   ecocyc   kegg   phen   173  malG  ecocyc   kegg  17.7 0.0  24  Deoxyadenosine  C00559  5.826
  10  Arachidonic acid metabolism 4e-15  8  Pyrimidine metabolism 0.000  20  Adenosine 0.004 1.000
   ybiU   ecocyc   kegg   phen   82  yfcP  ecocyc   kegg  7.6 0.0 &nbsp &nbsp &nbsp
 &nbsp &nbsp &nbsp  6  RNA polymerase 0.000 &nbsp &nbsp &nbsp
   ybiW   ecocyc   kegg   phen   198  hcaR  ecocyc   kegg  11.7 0.0  146  cis-Aconitate  C00417  9.797
  15  Microbial metabolism in diverse environments 8e-10  4  Bacterial secretion system 0.000  4  2-Oxobutanoate 0.000 0.000
   ybiX   ecocyc   kegg   phen   63  ycaI  ecocyc   kegg  15.8 0.0  10  3-Methyl-2-oxobutanoate  C00141  5.713
  13  Microbial metabolism in diverse environments 6e-09 &nbsp &nbsp &nbsp  2  Dihydroxyacetone 0.000 0.000
   ybiY   ecocyc   kegg   phen   113  rfaY  ecocyc   kegg  9.4 0.0  1  (R)-S-Lactoylglutathione  C03451  4.146
 &nbsp &nbsp &nbsp  9  Amino sugar and nucleotide sugar metabolism 0.000  10  UDPglucose 0.000 0.000
   ybjC   ecocyc   kegg   phen   101  nuoN  ecocyc   kegg  13.8 0.0  11  5-Methylthioadenosine  C00170  3.470
  9  Amino sugar and nucleotide sugar metabolism 5e-06  4  Two-component system 0.000  9  D-Glycerate 2-phosphate 0.000 0.000
   ybjD   ecocyc   kegg   phen   101  glmM  ecocyc   kegg  11.3 0.0  26  Dihydropteroate  C00921  4.461
  13  Glycine, serine and threonine metabolism 3e-08  16  Chlorocyclohexane and chlorobenzene degradation 0.000  5  Pyridoxine 5'-phosphate 0.000 0.000
   ybjE   ecocyc   kegg   phen   94  mraW  ecocyc   kegg  8.8 0.0  4  (R)-S-Lactoylglutathione  C03451  3.548
  1  Pyruvate metabolism 4e-05  8  Benzoate degradation 0.003  6  2,3-dehydroadipyl-CoA 0.000 0.000
   ybjI   ecocyc   kegg   phen   121  ybiN  ecocyc   kegg  7.9 0.0 &nbsp &nbsp &nbsp
 &nbsp &nbsp &nbsp  3  Phosphonate and phosphinate metabolism 0.001 &nbsp &nbsp &nbsp
   ybjK   ecocyc   kegg   phen   149  ygiU  ecocyc   kegg  10.2 0.0  2  1-dodecanoyl-sn-glycerol 3-phosphate  C00681  4.326
 &nbsp &nbsp &nbsp &nbsp &nbsp &nbsp  1  Phenylacetic acid 0.000 0.000
   ybjL   ecocyc   kegg   phen   110  ybbB  ecocyc   kegg  15.2 0.0  4  3-Phospho-D-glycerate  C00197  3.730
  12  C5-Branched dibasic acid metabolism 4e-13  2  Inositol phosphate metabolism 0.001  5  3-Aminoacrylate 0.000 0.000
   ybjM   ecocyc   kegg   phen   169  ybjR  ecocyc   kegg  10.2 0.0  1  Choline  C00114  -3.867
 &nbsp &nbsp &nbsp  17  Ethylbenzene degradation 0.000  27  Choline 0.009 1.000
   ybjN   ecocyc   kegg   phen   135  yciS  ecocyc   kegg  10.8 0.0  44  8-Amino-7-oxononanoate  C01092  6.353
  15  alpha-Linolenic acid metabolism 2e-08  10  Chlorocyclohexane and chlorobenzene degradation 0.000  1  Choline 0.001 0.000
   ybjP   ecocyc   kegg   phen   106  grxC  ecocyc   kegg  8.2 0.0 &nbsp &nbsp &nbsp
 &nbsp &nbsp &nbsp  3  D-Alanine metabolism 0.002 &nbsp &nbsp &nbsp
   ybjQ   ecocyc   kegg   phen   126  yffH  ecocyc   kegg  12.8 0.0  11  trans-Aconitate  C02341  4.082
  7  C5-Branched dibasic acid metabolism 0  7  Biosynthesis of siderophore group nonribosomal peptides 0.000  10  2,3-Dihydro-2,3-dihydroxybenzoate 0.000 0.000
   ybjS   ecocyc   kegg   phen   348  ygcS  ecocyc   kegg  11.2 0.0 &nbsp &nbsp &nbsp
 &nbsp &nbsp &nbsp  8  Chlorocyclohexane and chlorobenzene degradation 0.000 &nbsp &nbsp &nbsp
   ybjT   ecocyc   kegg   phen   142  yccX  ecocyc   kegg  16.2 0.0  9  Hypoxanthine  C00262  6.520
  9  Caprolactam degradation 4e-07  2  Phosphonate and phosphinate metabolism 0.001  12  Dodecanoly-phosphate (n-C12:0) 0.000 0.000
   ybjX   ecocyc   kegg   phen   142  bipA  ecocyc   kegg  16.0 0.0  6  1,5-Diaminopentane  C01672  4.173
  3  Fatty acid biosynthesis 8e-06  4  Bacterial secretion system 0.000  13  bis-molybdopterin guanine dinucleotide 0.000 0.000
   ycaD   ecocyc   kegg   phen   82  ygcB  ecocyc   kegg  15.3 0.0  14  Glycine betaine  C00719  3.882
  5  Sulfur metabolism 0.002  2  Biotin metabolism 0.004 &nbsp &nbsp &nbsp
   ycaI   ecocyc   kegg   phen   90  ybiX  ecocyc   kegg  15.8 0.0  7  Hexanoate (n-C6:0)  C01585  5.817
  13  Microbial metabolism in diverse environments 6e-09  1  Flagellar assembly 0.009  5  O-Acetyl-L-serine 0.000 0.000
   ycaK   ecocyc   kegg   phen   108  ygeG  ecocyc   kegg  18.3 0.0  15  Formaldehyde  C00067  6.922
  4  Arginine and proline metabolism 0  4  D-Glutamine and D-glutamate metabolism 0.001  6  O-Acetyl-L-serine 0.000 0.000
   ycaL   ecocyc   kegg   phen   118  ydcW  ecocyc   kegg  7.1 0.0 &nbsp &nbsp &nbsp
 &nbsp &nbsp &nbsp  2  Phosphotransferase system (PTS) 0.002 &nbsp &nbsp &nbsp
   ycaM   ecocyc   kegg   phen   127  ynjC  ecocyc   kegg  7.6 0.0 &nbsp &nbsp &nbsp
 &nbsp &nbsp &nbsp &nbsp &nbsp &nbsp &nbsp &nbsp &nbsp
   ycaN   ecocyc   kegg   phen   78  xapB  ecocyc   kegg  10.3 0.0  3  1,2-Diacyl-sn-glycerol (dioctadec-11-enoyl, n-C18:1)  C00641  11.043
  6  Arginine and proline metabolism 8e-06  6  Arachidonic acid metabolism 0.000  5  L-Proline 0.003 1.000
   ycaO   ecocyc   kegg   phen   136  yphF  ecocyc   kegg  9.2 0.0 &nbsp &nbsp &nbsp
 &nbsp &nbsp &nbsp  1  Ubiquinone and other terpenoid-quinone biosynthesis 0.010 &nbsp &nbsp &nbsp
   ycaP   ecocyc   kegg   phen   116  ycbK  ecocyc   kegg  17.3 0.0  18  cyclopropane phosphatidylethanolamine (dihexadec-9,10-cyclo-anoyl, n-C16:0 cyclo)  cyclopropane phosphatidylethanolamine (dihexadec-9,10-cyclo-anoyl, n-C16:0 cyclo)  5.324
  8  Starch and sucrose metabolism 8e-08  4  D-Glutamine and D-glutamate metabolism 0.001  7  dAMP 0.000 0.000
   ycaQ   ecocyc   kegg   phen   201  phnH  ecocyc   kegg  7.5 0.0 &nbsp &nbsp &nbsp
 &nbsp &nbsp &nbsp  1  Histidine metabolism 0.010 &nbsp &nbsp &nbsp
   ycaR   ecocyc   kegg   phen   111  yohL  ecocyc   kegg  10.2 0.0  3  Guanosine  C00387  3.838
 &nbsp &nbsp &nbsp  4  Protein export 0.001  5  L-Arabinose 0.000 0.000
   ycbB   ecocyc   kegg   phen   72  yohL  ecocyc   kegg  8.6 0.0 &nbsp &nbsp &nbsp
 &nbsp &nbsp &nbsp  6  Protein export 0.000 &nbsp &nbsp &nbsp
   ycbC   ecocyc   kegg   phen   154  ybgL  ecocyc   kegg  15.5 0.0  22  phosphatidylethanolamine (dihexadec-9enoyl, n-C16:1)  C00350  5.832
  3  Fatty acid biosynthesis 0  8  RNA polymerase 0.000  6  Decanoyl-ACP (n-C10:0ACP) 0.000 0.000
   ycbF   ecocyc   kegg   phen   177  ymdF  ecocyc   kegg  9.6 0.0  2  5-Phospho-alpha-D-ribose 1-diphosphate  C00119  4.883
 &nbsp &nbsp &nbsp  1  Phosphonate and phosphinate metabolism 0.002  7  Dihydroxyacetone 0.000 0.000
   ycbJ   ecocyc   kegg   phen   79  thiI  ecocyc   kegg  20.7 0.0  60  1-tetradec-7-enoyl-sn-glycerol 3-phosphate  C00681  31.618
  27  Starch and sucrose metabolism 1e-09  11  RNA polymerase 0.000  7  Decanoyl-ACP (n-C10:0ACP) 0.000 0.000
   ycbK   ecocyc   kegg   phen   87  yfaE  ecocyc   kegg  20.8 0.0  22  Dodecanoate (n-C12:0)  C02679  9.265
  12  Pyruvate metabolism 2e-06  5  Phenylalanine metabolism 0.001  8  trans-Cinnamate 0.000 0.000
   ycbL   ecocyc   kegg   phen   114  lsrF  ecocyc   kegg  8.2 0.0  1  &nbsp &nbsp
 &nbsp &nbsp &nbsp  5  Two-component system 0.000  15  D-Glycerate 2-phosphate 0.000 0.000
   ycbU   ecocyc   kegg   phen   178  yiaU  ecocyc   kegg  13.1 0.0  1  2,3-diaminopropionate  C06393  3.584
 &nbsp &nbsp &nbsp  3  Pyruvate metabolism 0.001  9  D-Tagatose 1,6-biphosphate 0.000 0.000
   ycbV   ecocyc   kegg   phen   98  ybiX  ecocyc   kegg  13.3 0.0  9  3-Methyl-2-oxobutanoate  C00141  5.076
  13  Microbial metabolism in diverse environments 6e-09  5  Fructose and mannose metabolism 0.002  5  Dihydroxyacetone 0.000 0.000
   ycbW   ecocyc   kegg   phen   278  ynfC  ecocyc   kegg  8.3 0.0 &nbsp &nbsp &nbsp
 &nbsp &nbsp &nbsp  3  Chlorocyclohexane and chlorobenzene degradation 0.000 &nbsp &nbsp &nbsp
   ycbX   ecocyc   kegg   phen   37  stfE  ecocyc   kegg  20.0 0.0  9  octadecanoate (n-C18:0)  C01530  4.807
  5  Lysine degradation 8e-09  9  Terpenoid backbone biosynthesis 0.000 &nbsp &nbsp &nbsp
   ycbZ   ecocyc   kegg   phen   118  ygaX  ecocyc   kegg  8.6 0.0  1  2-Succinyl-6-hydroxy-2,4-cyclohexadiene-1-carboxylate  C05817  -3.640
 &nbsp &nbsp &nbsp  4  D-Glutamine and D-glutamate metabolism 0.001  4  o-Succinylbenzoate 0.000 0.000
   yccE   ecocyc   kegg   phen   61  hisG  ecocyc   kegg  16.1 0.0  12  Pyridoxamine  C00534  4.027
  9  Lipoic acid metabolism 0  6  Nicotinate and nicotinamide metabolism 0.000  2  Iminoaspartate 0.000 0.000
   yccF   ecocyc   kegg   phen   74  yegU  ecocyc   kegg  16.0 0.0  37  1,6-anhydrous-N-Acetylmuramate  1,6-anhydrous-N-Acetylmuramate  38.552
  7  Purine metabolism 4e-07  1  Biosynthesis of unsaturated fatty acids 0.004  5  [4Fe-4S] iron-sulfur cluster 0.000 0.000
   yccJ   ecocyc   kegg   phen   120  yecF  ecocyc   kegg  12.8 0.0 &nbsp &nbsp &nbsp
  1  Arachidonic acid metabolism 0  2  Taurine and hypotaurine metabolism 0.001 &nbsp &nbsp &nbsp
   yccM   ecocyc   kegg   phen   156  ydiB  ecocyc   kegg  11.7 0.0  15  5'-deoxyribose  5'-deoxyribose  3.893
  15  C5-Branched dibasic acid metabolism 2e-12  8  Pentose and glucuronate interconversions 0.002  26  5-Phospho-beta-D-ribosylamine 0.000 0.000
   yccS   ecocyc   kegg   phen   144  lar  ecocyc   kegg  10.8 0.0  1  ferroxamine minus Fe(3)  ferroxamine minus Fe(3)  3.774
 &nbsp &nbsp &nbsp  1  Glycerolipid metabolism 0.007  10  Dodecanoly-phosphate (n-C12:0) 0.000 0.000
   yccT   ecocyc   kegg   phen   59  yfaE  ecocyc   kegg  25.6 0.0  15  Dodecanoate (n-C12:0)  C02679  15.957
  8  Fatty acid biosynthesis 5e-08  5  Cysteine and methionine metabolism 0.001  3  L-Serine 0.000 0.000
   yccU   ecocyc   kegg   phen   158  ymdF  ecocyc   kegg  7.5 0.0 &nbsp &nbsp &nbsp
 &nbsp &nbsp &nbsp  3  Glycerolipid metabolism 0.001 &nbsp &nbsp &nbsp
   yccX   ecocyc   kegg   phen   132  ybjT  ecocyc   kegg  16.2 0.0  7  L-tartrate  C00898  3.871
  9  Caprolactam degradation 3e-07  2  Phosphonate and phosphinate metabolism 0.001  6  O-Acetyl-L-serine 0.000 0.000
   ycdT   ecocyc   kegg   phen   294  pnp  ecocyc   kegg  12.7 0.0 &nbsp &nbsp &nbsp
 &nbsp &nbsp &nbsp  5  Chlorocyclohexane and chlorobenzene degradation 0.000 &nbsp &nbsp &nbsp
   ycdU   ecocyc   kegg   phen   344  bioH  ecocyc   kegg  8.6 0.0  4  D-Glycero-D-manno-heptose 1,7-bisphosphate  C11472  -3.825
 &nbsp &nbsp &nbsp  8  Chlorocyclohexane and chlorobenzene degradation 0.000  13  D-Tagatose 1,6-biphosphate 0.000 0.000
   ycdX   ecocyc   kegg   phen   141  ybgH  ecocyc   kegg  16.4 0.0  6  1,6-anhydrous-N-Acetylmuramyl-tripeptide  1,6-anhydrous-N-Acetylmuramyl-tripeptide  3.767
  5  Thiamine metabolism 7e-05  4  Inositol phosphate metabolism 0.001  4  3-Hydroxypropanoate 0.000 0.000
   ycdY   ecocyc   kegg   phen   78  abgR  ecocyc   kegg  12.5 0.0  11  D-4'-Phosphopantothenate  C03492  4.242
  9  Fatty acid biosynthesis 2e-09  1  Flagellar assembly 0.006 &nbsp &nbsp &nbsp
   ycdZ   ecocyc   kegg   phen   170  ycbF  ecocyc   kegg  8.4 0.0 &nbsp &nbsp &nbsp
 &nbsp &nbsp &nbsp  2  Bisphenol degradation 0.002 &nbsp &nbsp &nbsp
   yceA   ecocyc   kegg   phen   145  ychJ  ecocyc   kegg  23.0 0.0  26  2-octadec-11-enoyl-sn-glycerol 3-phosphate  C03974  5.655
  9  Fatty acid biosynthesis 6e-07  12  RNA polymerase 0.000  9  Decanoyl-ACP (n-C10:0ACP) 0.000 0.000
   yceB   ecocyc   kegg   phen   79  yecR  ecocyc   kegg  8.0 0.0  2  Malonate semialdehyde  C00222  4.294
 &nbsp &nbsp &nbsp  2  Other glycan degradation 0.001  1  Succinate 0.003 0.000
   yceD   ecocyc   kegg   phen   122  rnb  ecocyc   kegg  11.0 0.0  20  glycogen  C00182  3.996
  10  Fatty acid biosynthesis 1e-05  8  Chlorocyclohexane and chlorobenzene degradation 0.000  8  Pyridoxine 5'-phosphate 0.000 0.000
   yceF   ecocyc   kegg   phen   176  ydfO  ecocyc   kegg  9.3 0.0 &nbsp &nbsp &nbsp
 &nbsp &nbsp &nbsp  1  Phosphonate and phosphinate metabolism 0.002 &nbsp &nbsp &nbsp
   yceG   ecocyc   kegg   phen   249  yhjC  ecocyc   kegg  8.9 0.0  1  2-C-methyl-D-erythritol 2,4-cyclodiphosphate  C11453  -3.579
  1  Glycerophospholipid metabolism 0  4  Chlorocyclohexane and chlorobenzene degradation 0.000  9  Guanosine 3'-diphosphate 5'-triphosphate 0.000 0.000
   yceH   ecocyc   kegg   phen   110  yfiQ  ecocyc   kegg  9.0 0.0  6  4-Phospho-L-aspartate  C03082  3.955
  19  Methane metabolism 6e-07  3  Oxidative phosphorylation 0.002  7  D-Glycerate 2-phosphate 0.000 0.000
   yceI   ecocyc   kegg   phen   114  fimE  ecocyc   kegg  11.8 0.0  37  o-Succinylbenzoate  C02730  4.454
  9  Phenylalanine metabolism 2e-05  3  Two-component system 0.000 &nbsp &nbsp &nbsp
   yceJ   ecocyc   kegg   phen   89  nuoM  ecocyc   kegg  17.8 0.0  30  Formaldehyde  C00067  8.881
  14  Microbial metabolism in diverse environments 3e-05  1  D-Glutamine and D-glutamate metabolism 0.001  1  potassium 0.001 0.000
   yceK   ecocyc   kegg   phen   85  tnaA  ecocyc   kegg  8.2 0.0  1  Indole  C00463  -3.970
  3  Phenylalanine, tyrosine and tryptophan biosynthesis 3e-05  1  Fructose and mannose metabolism 0.007  7  undecaprenyl phosphate-4-amino-4-deoxy-L-arabinose 0.000 0.000
   yceO   ecocyc   kegg   phen   80  hscB  ecocyc   kegg  16.6 0.0  36  trans-Aconitate  C02341  4.723
  10  C5-Branched dibasic acid metabolism 2e-15  4  Biosynthesis of siderophore group nonribosomal peptides 0.000  18  2,3-Dihydro-2,3-dihydroxybenzoate 0.000 0.000
   ycfD   ecocyc   kegg   phen   87  ybdN  ecocyc   kegg  11.5 0.0  41  Maltose 6'-phosphate  C02995  23.175
  8  Inositol phosphate metabolism 4e-07  3  Arachidonic acid metabolism 0.001  3  L-Idonate 0.000 0.000
   ycfH   ecocyc   kegg   phen   105  yjeK  ecocyc   kegg  7.6 0.0 &nbsp &nbsp &nbsp
 &nbsp &nbsp &nbsp  5  Chlorocyclohexane and chlorobenzene degradation 0.000 &nbsp &nbsp &nbsp
   ycfJ   ecocyc   kegg   phen   328  hinT  ecocyc   kegg  11.0 0.0  15  2-Acyl-sn-glycero-3-phosphoethanolamine (n-C16:0)  C05973  4.670
  7  Galactose metabolism 6e-10 &nbsp &nbsp &nbsp  20  O-acetyl-rhamanosyl-N-acetylglucosamyl-undecaprenyl diphosphate 0.000 0.000
   ycfL   ecocyc   kegg   phen   55  basR  ecocyc   kegg  16.2 0.0  23  3-Dehydroshikimate  C02637  4.000
  10  Fatty acid biosynthesis 8e-06  6  Chloroalkane and chloroalkene degradation 0.001  8  O-Acetyl-L-serine 0.000 0.000
   ycfP   ecocyc   kegg   phen   79  ybfG  ecocyc   kegg  7.8 0.0  2  Succinate  C00042  -3.556
  14  Chlorocyclohexane and chlorobenzene degradation 2e-08 &nbsp &nbsp &nbsp  4  NMN 0.000 0.000
   ycfQ   ecocyc   kegg   phen   207  ymfA  ecocyc   kegg  11.4 0.0  3  7-cyano-7-carbaguanine  C15996  3.456
 &nbsp &nbsp &nbsp  1  Glycerolipid metabolism 0.002  8  Dihydroxyacetone 0.000 0.000
   ycfS   ecocyc   kegg   phen   129  yrhA  ecocyc   kegg  8.1 0.0  1  &nbsp &nbsp
  2  Terpenoid backbone biosynthesis 0  5  Chlorocyclohexane and chlorobenzene degradation 0.000  2  Acetol 0.000 0.000
   ycfT   ecocyc   kegg   phen   105  yadL  ecocyc   kegg  8.6 0.0  3  Phosphatidylglycerophosphate (didodecanoyl, n-C12:0)  C03892  3.838
  1  Porphyrin and chlorophyll metabolism 0  2  Lysine biosynthesis 0.002  3  L-Valine 0.001 0.000
   ycgB   ecocyc   kegg   phen   12  pepP  ecocyc   kegg  33.0 0.0  22  1-dodecanoyl-sn-glycerol 3-phosphate  C00681  5.933
  16  Biosynthesis of secondary metabolites 1e-07  2  Glutathione metabolism 0.000 &nbsp &nbsp &nbsp
   ycgE   ecocyc   kegg   phen   88  mcrA  ecocyc   kegg  9.9 0.0  15  4-Hydroxy-L-threonine  C06056  3.561
  9  Pantothenate and CoA biosynthesis 0.0002  8  Fatty acid biosynthesis 0.001  7  malonyl-CoA methyl ester 0.000 0.000
   ycgF   ecocyc   kegg   phen   126  yejF  ecocyc   kegg  8.9 0.0  1  Succinate  C00042  3.559
 &nbsp &nbsp &nbsp  3  Toluene degradation 0.003  9  Dihydroxyacetone 0.000 0.000
   ycgG   ecocyc   kegg   phen   156  ylaC  ecocyc   kegg  9.8 0.0  5  1-dodecanoyl-sn-glycerol 3-phosphate  C00681  4.296
  5  Taurine and hypotaurine metabolism 0.0002  2  Phosphonate and phosphinate metabolism 0.002  6  Dihydroxyacetone 0.000 0.000
   ycgI   ecocyc   kegg   phen   93  yceO  ecocyc   kegg  14.5 0.0  27  2-C-methyl-D-erythritol 2,4-cyclodiphosphate  C11453  4.981
  8  C5-Branched dibasic acid metabolism 0  5  Biosynthesis of siderophore group nonribosomal peptides 0.000  12  2,3-Dihydro-2,3-dihydroxybenzoate 0.000 0.000
   ycgJ   ecocyc   kegg   phen   475  etp  ecocyc   kegg  10.1 0.0 &nbsp &nbsp &nbsp
 &nbsp &nbsp &nbsp  6  Chlorocyclohexane and chlorobenzene degradation 0.000 &nbsp &nbsp &nbsp
   ycgM   ecocyc   kegg   phen   85  arcA  ecocyc   kegg  9.0 0.0  7  N1-Acetylspermidine  C00612  4.236
  6  Pantothenate and CoA biosynthesis 3e-07  6  D-Glutamine and D-glutamate metabolism 0.000  3  D-Glucosamine 6-phosphate 0.001 0.000
   ycgN   ecocyc   kegg   phen   129  ymdF  ecocyc   kegg  8.4 0.0 &nbsp &nbsp &nbsp
 &nbsp &nbsp &nbsp  1  Glycerolipid metabolism 0.005 &nbsp &nbsp &nbsp
   ycgV   ecocyc   kegg   phen   85  ygeH  ecocyc   kegg  13.5 0.0  27  2-C-methyl-D-erythritol 2,4-cyclodiphosphate  C11453  3.946
  8  Citrate cycle (TCA cycle) 7e-05  13  Ubiquinone and other terpenoid-quinone biosynthesis 0.000  16  2-Octaprenyl-6-methoxy-1,4-benzoquinol 0.000 0.000
   ycgX   ecocyc   kegg   phen   121  sodC  ecocyc   kegg  16.7 0.0  44  Hypoxanthine  C00262  7.687
  13  C5-Branched dibasic acid metabolism 8e-07  12  Ubiquinone and other terpenoid-quinone biosynthesis 0.000  13  2,3-Dihydro-2,3-dihydroxybenzoate 0.000 0.000
   ycgY   ecocyc   kegg   phen   95  yaiI  ecocyc   kegg  7.0 0.0 &nbsp &nbsp &nbsp
 &nbsp &nbsp &nbsp  7  Chlorocyclohexane and chlorobenzene degradation 0.000 &nbsp &nbsp &nbsp
   ycgZ   ecocyc   kegg   phen   191  modE  ecocyc   kegg  10.6 0.0  1  N2-Succinyl-L-arginine  C03296  3.712
  1  Arginine and proline metabolism 0  1  Dioxin degradation 0.002  9  crotonobetainyl-CoA 0.000 0.000
   ychA   ecocyc   kegg   phen   89  yaiI  ecocyc   kegg  8.9 0.0  4  (R)-Glycerate  C00258  4.252
 &nbsp &nbsp &nbsp  2  Ribosome 0.004 &nbsp &nbsp &nbsp
   ychE   ecocyc   kegg   phen   253  gabT  ecocyc   kegg  7.4 0.0  3  Nicotinate  C00253  3.465
 &nbsp &nbsp &nbsp  1  Base excision repair 0.008  10  glucosyl-O-acetyl-rhamanosyl-N-acetylglucosamyl-undecaprenyl diphosphate 0.000 0.000
   ychF   ecocyc   kegg   phen   164  cysH  ecocyc   kegg  9.1 0.0  109  (R)-Pantothenate  C00864  6.713
  19  Fructose and mannose metabolism 4e-13  13  Chlorocyclohexane and chlorobenzene degradation 0.000  20  Adenosine 5'-phosphosulfate 0.000 0.000
   ychH   ecocyc   kegg   phen   61  ycgR  ecocyc   kegg  8.3 0.0  3  D-Alanyl-D-alanine  C00993  -4.262
  4  Arginine and proline metabolism 0 &nbsp &nbsp &nbsp &nbsp &nbsp &nbsp
   ychJ   ecocyc   kegg   phen   148  yceA  ecocyc   kegg  23.0 0.0  29  Hexadecanoate (n-C16:0)  C00249  6.441
  9  Valine, leucine and isoleucine biosynthesis 1e-07  11  RNA polymerase 0.000  8  Dodecanoyl-ACP (n-C12:0ACP) 0.000 0.000
   ychM   ecocyc   kegg   phen   229  yeeU  ecocyc   kegg  10.0 0.0 &nbsp &nbsp &nbsp
 &nbsp &nbsp &nbsp  2  Biosynthesis of siderophore group nonribosomal peptides 0.004 &nbsp &nbsp &nbsp
   ychN   ecocyc   kegg   phen   105  ynbA  ecocyc   kegg  10.0 0.0 &nbsp &nbsp &nbsp
 &nbsp &nbsp &nbsp  4  Taurine and hypotaurine metabolism 0.000 &nbsp &nbsp &nbsp
   ychP   ecocyc   kegg   phen   85  fiu  ecocyc   kegg  8.8 0.0 &nbsp &nbsp &nbsp
 &nbsp &nbsp &nbsp  2  Two-component system 0.007 &nbsp &nbsp &nbsp
   ychQ   ecocyc   kegg   phen   99  ycfL  ecocyc   kegg  10.9 0.0  5  tetradecanoate (n-C14:0)  C06424  4.635
  1  Arachidonic acid metabolism 0.0002  5  Ribosome 0.000  1  2-Oxobutanoate 0.001 0.000
   yciA   ecocyc   kegg   phen   68  ybaW  ecocyc   kegg  6.2 0.0 &nbsp &nbsp &nbsp
 &nbsp &nbsp &nbsp  1  Other glycan degradation 0.001 &nbsp &nbsp &nbsp
   yciB   ecocyc   kegg   phen   190  rpsT  ecocyc   kegg  9.9 0.0  8  2-C-methyl-D-erythritol 2,4-cyclodiphosphate  C11453  3.471
  5  Lysine degradation 0  6  Chlorocyclohexane and chlorobenzene degradation 0.000  3  Acetol 0.000 0.000
   yciC   ecocyc   kegg   phen   76  yfaL  ecocyc   kegg  5.8 0.0  6  5-Dehydro-4-deoxy-D-glucarate  C00679  3.559
  1  Phenylalanine metabolism 0 &nbsp &nbsp &nbsp &nbsp &nbsp &nbsp
   yciE   ecocyc   kegg   phen   130  yebV  ecocyc   kegg  8.0 0.0  18  2-Octaprenyl-6-methoxyphenol  C05812  4.842
  2  Ethylbenzene degradation 0  1  Other glycan degradation 0.003  6  L-Malate 0.000 0.000
   yciG   ecocyc   kegg   phen   83  yejL  ecocyc   kegg  10.9 0.0  7  1,2-didodecanoyl-sn-glycerol 3-phosphate  C00416  3.615
  6  Glycolysis / Gluconeogenesis 0  1  Other glycan degradation 0.001  4  Carbamoyl phosphate 0.000 0.000
   yciH   ecocyc   kegg   phen   159  yjeK  ecocyc   kegg  9.3 0.0  4  L-Histidinol phosphate  C01100  4.597
  2  Histidine metabolism 7e-05  6  Pantothenate and CoA biosynthesis 0.001  5  4-Phospho-L-aspartate 0.000 0.000
   yciI   ecocyc   kegg   phen   210  fkpA  ecocyc   kegg  10.4 0.0  52  2-hydroxy-6-ketononatrienedioate  C12624  6.006
  19  D-Alanine metabolism 5e-08  6  Flagellar assembly 0.000  9  L-Proline 0.008 1.000
   yciK   ecocyc   kegg   phen   99  frwD  ecocyc   kegg  8.5 0.0  1  Dethiobiotin  C01909  3.836
 &nbsp &nbsp &nbsp  8  Glycerophospholipid metabolism 0.002  4  Zinc 0.001 0.000
   yciM   ecocyc   kegg   phen   29  yeaO  ecocyc   kegg  31.1 0.0  29  N-Acetylneuraminate  C00270  10.862
  10  alpha-Linolenic acid metabolism 2e-09  3  Inositol phosphate metabolism 0.000  2  Dihydroxyacetone phosphate 0.000 0.000
   yciN   ecocyc   kegg   phen   110  yccJ  ecocyc   kegg  8.7 0.0  18  N-Acetylanthranilate  C06332  5.277
  8  Butanoate metabolism 2e-07  1  Arachidonic acid metabolism 0.001  4  N-Acetyl-D-glucosamine 6-phosphate 0.000 0.000
   yciO   ecocyc   kegg   phen   70  ybhJ  ecocyc   kegg  26.5 0.0  40  tetradecenoate (n-C14:1)  tetradecenoate (n-C14:1)  13.698
  9  Limonene and pinene degradation 1e-15  5  Vitamin B6 metabolism 0.000  3  Pyridoxine 5'-phosphate 0.000 0.000
   yciQ   ecocyc   kegg   phen   42  ybhJ  ecocyc   kegg  28.9 0.0  17  tetradecenoate (n-C14:1)  tetradecenoate (n-C14:1)  11.704
  7  Limonene and pinene degradation 1e-16  8  Terpenoid backbone biosynthesis 0.000 &nbsp &nbsp &nbsp
   yciS   ecocyc   kegg   phen   148  fdhE  ecocyc   kegg  16.3 0.0  108  cyclopropane phosphatidylethanolamine (dihexadec-9,10-cyclo-anoyl, n-C16:0 cyclo)  cyclopropane phosphatidylethanolamine (dihexadec-9,10-cyclo-anoyl, n-C16:0 cyclo)  8.450
  15  alpha-Linolenic acid metabolism 0  16  Butanoate metabolism 0.000  19  Decanoyl-ACP (n-C10:0ACP) 0.000 0.000
   yciU   ecocyc   kegg   phen   158  ypfE  ecocyc   kegg  10.6 0.0  28  cyclopropane phosphatidylethanolamine (dihexadec-9,10-cyclo-anoyl, n-C16:0 cyclo)  cyclopropane phosphatidylethanolamine (dihexadec-9,10-cyclo-anoyl, n-C16:0 cyclo)  4.545
  8  C5-Branched dibasic acid metabolism 0  14  RNA polymerase 0.000  26  Crotonoyl-CoA 0.000 0.000
   yciV   ecocyc   kegg   phen   135  aceE  ecocyc   kegg  14.5 0.0  30  L-methionine-R-sulfoxide  C15998  11.083
  6  Fatty acid biosynthesis 3e-08  9  Nitrotoluene degradation 0.000  5  Glycerol 0.000 0.000
   yciW   ecocyc   kegg   phen   115  ynjB  ecocyc   kegg  7.9 0.0 &nbsp &nbsp &nbsp
 &nbsp &nbsp &nbsp  2  Amino sugar and nucleotide sugar metabolism 0.007 &nbsp &nbsp &nbsp
   ycjD   ecocyc   kegg   phen   241  nadR  ecocyc   kegg  7.6 0.0  2  1,2-Diacyl-sn-glycerol (dihexadec-9-enoyl, n-C16:1)  C00641  3.496
  4  Riboflavin metabolism 4e-05 &nbsp &nbsp &nbsp  13  glucosyl-O-acetyl-rhamanosyl-N-acetylglucosamyl-undecaprenyl diphosphate 0.000 0.000
   ycjF   ecocyc   kegg   phen   81  ypdB  ecocyc   kegg  10.3 0.0  5  N(omega)-(L-Arginino)succinate  C03406  3.789
 &nbsp &nbsp &nbsp  4  Other glycan degradation 0.001  5  Undecaprenyl phosphate 0.000 0.000
   ycjM   ecocyc   kegg   phen   128  yegX  ecocyc   kegg  8.3 0.0  6  N1-Acetylspermidine  C00612  3.766
  4  Riboflavin metabolism 7e-05 &nbsp &nbsp &nbsp  4  L-Serine 0.000 0.000
   ycjQ   ecocyc   kegg   phen   35  ygjI  ecocyc   kegg  22.3 0.0  8  2-Acyl-sn-glycero-3-phosphoethanolamine (n-C14:1)  C05973  8.064
  1  Arachidonic acid metabolism 8e-10  3  Protein export 0.002  1  L-Arginine 0.001 0.000
   ycjR   ecocyc   kegg   phen   114  yfjW  ecocyc   kegg  9.5 0.0  1  Hexadecanoyl-phosphate (n-C16:0)  Hexadecanoyl-phosphate (n-C16:0)  4.476
 &nbsp &nbsp &nbsp  2  Mismatch repair 0.001  2  Dihydroxyacetone 0.000 0.000
   ycjS   ecocyc   kegg   phen   100  yebE  ecocyc   kegg  12.5 0.0  16  Butanal  C01412  5.660
  21  Alanine, aspartate and glutamate metabolism 5e-05  10  RNA polymerase 0.000  3  Succinate 0.000 0.000
   ycjT   ecocyc   kegg   phen   98  ycdX  ecocyc   kegg  10.9 0.0 &nbsp &nbsp &nbsp
 &nbsp &nbsp &nbsp  1  Taurine and hypotaurine metabolism 0.009 &nbsp &nbsp &nbsp
   ycjU   ecocyc   kegg   phen   68  ushA  ecocyc   kegg  9.0 0.0  10  phosphatidylethanolamine (dihexadec-9enoyl, n-C16:1)  C00350  3.523
  8  Glycine, serine and threonine metabolism 1e-06  8  Bacterial secretion system 0.000  10  GDP-4-dehydro-6-deoxy-D-mannose 0.000 0.000
   ycjV   ecocyc   kegg   phen   115  chaA  ecocyc   kegg  10.8 0.0 &nbsp &nbsp &nbsp
 &nbsp &nbsp &nbsp  6  ABC transporters 0.000 &nbsp &nbsp &nbsp
   ycjW   ecocyc   kegg   phen   103  yjeK  ecocyc   kegg  8.7 0.0  4  3-Phosphohydroxypyruvate  C03232  3.608
  1  Biosynthesis of secondary metabolites 0  16  Pantothenate and CoA biosynthesis 0.000  8  4-Phospho-L-aspartate 0.000 0.000
   ycjX   ecocyc   kegg   phen   68  yfhJ  ecocyc   kegg  8.7 0.0  6  methylisocitrate  C04593  3.487
  3  Arachidonic acid metabolism 2e-08  2  ABC transporters 0.003  7  D-Alanyl-D-alanine 0.000 0.000
   ydaC   ecocyc   kegg   phen   48  thiI  ecocyc   kegg  23.6 0.0  25  1-tetradec-7-enoyl-sn-glycerol 3-phosphate  C00681  23.197
  8  Biosynthesis of secondary metabolites 1e-05  6  Novobiocin biosynthesis 0.001  1  2-Oxoglutarate 0.006 0.000
   ydaE   ecocyc   kegg   phen   95  yaaI  ecocyc   kegg  12.1 0.0  3  2-hexadec-9-enoyl-sn-glycerol 3-phosphate  C03974  3.667
 &nbsp &nbsp &nbsp &nbsp &nbsp &nbsp  3  Uracil 0.000 0.000
   ydaF   ecocyc   kegg   phen   98  yiaU  ecocyc   kegg  11.0 0.0  13  (S)-3-Methyl-2-oxopentanoate  C00671  3.757
  21  Methane metabolism 3e-06  2  Glycolysis / Gluconeogenesis 0.008  3  2-Oxobutanoate 0.000 0.000
   ydaG   ecocyc   kegg   phen   154  yfjW  ecocyc   kegg  13.6 0.0  2  1-dodecanoyl-sn-glycerol 3-phosphate  C00681  5.688
  1  Lysine biosynthesis 0.0009 &nbsp &nbsp &nbsp  6  L-Malate 0.000 0.000
   ydaQ   ecocyc   kegg   phen   115  yajR  ecocyc   kegg  8.0 0.0 &nbsp &nbsp &nbsp
 &nbsp &nbsp &nbsp  2  Phosphonate and phosphinate metabolism 0.001 &nbsp &nbsp &nbsp
   ydaS   ecocyc   kegg   phen   123  holD  ecocyc   kegg  11.7 0.0  7  N1-Acetylspermidine  C00612  3.779
 &nbsp &nbsp &nbsp  12  Sulfur metabolism 0.000  7  4-Phospho-L-aspartate 0.000 0.000
   ydaT   ecocyc   kegg   phen   105  ygeD  ecocyc   kegg  8.0 0.0  14  L-Isoleucine  C00407  3.476
  10  Tryptophan metabolism 5e-06  6  Amino sugar and nucleotide sugar metabolism 0.001  5  Protoheme 0.000 0.000
   ydaU   ecocyc   kegg   phen   73  nrdI  ecocyc   kegg  17.2 0.0  54  Hypoxanthine  C00262  33.416
  16  Microbial metabolism in diverse environments 5e-10  3  Terpenoid backbone biosynthesis 0.001  4  L-Cysteine 0.001 1.000
   ydaV   ecocyc   kegg   phen   121  yjgA  ecocyc   kegg  13.7 0.0  11  D-Glycero-D-manno-heptose 7-phosphate  C07836  4.582
  1  Glycerophospholipid metabolism 1e-05  6  Homologous recombination 0.000  4  Taurine 0.000 0.000
   ydaW   ecocyc   kegg   phen   38  yciO  ecocyc   kegg  24.1 0.0  12  tetradecenoate (n-C14:1)  tetradecenoate (n-C14:1)  6.923
  6  Limonene and pinene degradation 0  12  Terpenoid backbone biosynthesis 0.000 &nbsp &nbsp &nbsp
   ydaY   ecocyc   kegg   phen   87  ycjT  ecocyc   kegg  7.7 0.0 &nbsp &nbsp &nbsp
 &nbsp &nbsp &nbsp  2  Other glycan degradation 0.002 &nbsp &nbsp &nbsp
   ydbC   ecocyc   kegg   phen   109  ydfI  ecocyc   kegg  11.5 0.0  4  (R)-S-Lactoylglutathione  C03451  4.367
  4  Biosynthesis of secondary metabolites 3e-05  1  Lipopolysaccharide biosynthesis 0.007  13  ADP-L-glycero-D-manno-heptose 0.000 0.000
   ydbD   ecocyc   kegg   phen   159  ydfO  ecocyc   kegg  8.2 0.0 &nbsp &nbsp &nbsp
 &nbsp &nbsp &nbsp  3  Phosphonate and phosphinate metabolism 0.002 &nbsp &nbsp &nbsp
   ydbH   ecocyc   kegg   phen   136  yaiI  ecocyc   kegg  15.7 0.0  49  Dihydroneopterin monophosphate  C05925  5.284
  6  Purine metabolism 4e-08  3  Lipoic acid metabolism 0.004  2  GTP 0.001 1.000
   ydbJ   ecocyc   kegg   phen   359  yieP  ecocyc   kegg  9.8 0.0 &nbsp &nbsp &nbsp
 &nbsp &nbsp &nbsp  6  Chlorocyclohexane and chlorobenzene degradation 0.000 &nbsp &nbsp &nbsp
   ydbK   ecocyc   kegg   phen   99  sufC  ecocyc   kegg  10.4 0.0 &nbsp &nbsp &nbsp
  2  Aminobenzoate degradation 0  10  Biosynthesis of siderophore group nonribosomal peptides 0.000 &nbsp &nbsp &nbsp
   ydbL   ecocyc   kegg   phen   159  lar  ecocyc   kegg  8.0 0.0 &nbsp &nbsp &nbsp
 &nbsp &nbsp &nbsp  2  Phosphonate and phosphinate metabolism 0.002 &nbsp &nbsp &nbsp
   ydcC   ecocyc   kegg   phen   109  torZ  ecocyc   kegg  11.1 0.0 &nbsp &nbsp &nbsp
  5  C5-Branched dibasic acid metabolism 8e-12  5  Chlorocyclohexane and chlorobenzene degradation 0.000 &nbsp &nbsp &nbsp
   ydcD   ecocyc   kegg   phen   78  yccT  ecocyc   kegg  19.5 0.0  14  Dodecanoate (n-C12:0)  C02679  11.040
  6  Fatty acid biosynthesis 1e-12  6  Cysteine and methionine metabolism 0.000  5  O-Succinyl-L-homoserine 0.000 0.000
   ydcF   ecocyc   kegg   phen   87  pgpA  ecocyc   kegg  8.4 0.0  13  1,2-Diacyl-sn-glycerol (dioctadec-11-enoyl, n-C18:1)  C00641  10.375
  6  Arginine and proline metabolism 8e-06  11  Pantothenate and CoA biosynthesis 0.000  6  2-Octaprenyl-3-methyl-5-hydroxy-6-methoxy-1,4-benzoquinol 0.000 0.000
   ydcH   ecocyc   kegg   phen   366  yegR  ecocyc   kegg  12.2 0.0  1  Phenylacetic acid  C07086  3.842
 &nbsp &nbsp &nbsp  8  Chlorocyclohexane and chlorobenzene degradation 0.000  14  Adenosine 5'-phosphosulfate 0.000 0.000
   ydcI   ecocyc   kegg   phen   92  ycgG  ecocyc   kegg  9.3 0.0  3  5,10-Methenyltetrahydrofolate  C00445  4.057
  1  Lysine biosynthesis 0.0009  3  Bisphenol degradation 0.001  1  Dihydroxyacetone phosphate 0.001 0.000
   ydcJ   ecocyc   kegg   phen   112  yejK  ecocyc   kegg  8.2 0.0  5  4-Phospho-L-aspartate  C03082  4.403
  4  Vitamin B6 metabolism 3e-06  7  RNA polymerase 0.000  13  5-Methyltetrahydrofolate 0.000 0.000
   ydcK   ecocyc   kegg   phen   76  atoS  ecocyc   kegg  8.9 0.0  8  2-succinyl-5-enolpyruvyl-6-hydroxy-3-cyclohexene-1-carboxylate  C16519  3.994
  3  Bisphenol degradation 0.0001  6  Glycolysis / Gluconeogenesis 0.000 &nbsp &nbsp &nbsp
   ydcL   ecocyc   kegg   phen   101  hscA  ecocyc   kegg  9.6 0.0  8  phosphatidylethanolamine (dioctadec-11-enoyl, n-C18:1)  C00350  5.753
  5  Biotin metabolism 6e-05  11  Novobiocin biosynthesis 0.000  9  3-(4-Hydroxyphenyl)pyruvate 0.000 0.000
   ydcN   ecocyc   kegg   phen   110  ynbB  ecocyc   kegg  11.8 0.0  6  L-Lactate  C00186  4.284
 &nbsp &nbsp &nbsp  18  Fatty acid metabolism 0.000  7  2-Octaprenyl-3-methyl-5-hydroxy-6-methoxy-1,4-benzoquinol 0.000 0.000
   ydcO   ecocyc   kegg   phen   150  hcr  ecocyc   kegg  11.9 0.0  4  L-tartrate  C00898  5.194
  2  Porphyrin and chlorophyll metabolism 3e-05  3  Phosphonate and phosphinate metabolism 0.002  4  Dihydroxyacetone 0.000 0.000
   ydcP   ecocyc   kegg   phen   98  slp  ecocyc   kegg  12.4 0.0  49  8-Amino-7-oxononanoate  C01092  10.745
  12  Lysine degradation 9e-14  18  Two-component system 0.000  8  D-Glycerate 2-phosphate 0.000 0.000
   ydcR   ecocyc   kegg   phen   123  ycgB  ecocyc   kegg  25.4 0.0  7  1-tetradecanoyl-sn-glycerol 3-phosphate  C00681  4.546
  1  Biosynthesis of secondary metabolites 0  4  Glutathione metabolism 0.002  7  Nitrous oxide 0.000 0.000
   ydcX   ecocyc   kegg   phen   133  ybiN  ecocyc   kegg  10.5 0.0 &nbsp &nbsp &nbsp
 &nbsp &nbsp &nbsp  4  Phosphonate and phosphinate metabolism 0.001 &nbsp &nbsp &nbsp
   ydcY   ecocyc   kegg   phen   155  yedE  ecocyc   kegg  12.1 0.0  10  4-Aminobenzoate  C00568  3.751
  12  Aminobenzoate degradation 5e-05  7  RNA polymerase 0.000  6  7,8-Diaminononanoate 0.000 0.000
   ydcZ   ecocyc   kegg   phen   119  yaaI  ecocyc   kegg  11.1 0.0 &nbsp &nbsp &nbsp
  2  Taurine and hypotaurine metabolism 8e-06  1  D-Glutamine and D-glutamate metabolism 0.001 &nbsp &nbsp &nbsp
   yddB   ecocyc   kegg   phen   80  chaA  ecocyc   kegg  15.4 0.0  1  Thiamin  C00378  4.680
 &nbsp &nbsp &nbsp  6  ABC transporters 0.000  11  Xanthosine 0.000 0.000
   yddE   ecocyc   kegg   phen   98  ybjQ  ecocyc   kegg  10.7 0.0 &nbsp &nbsp &nbsp
 &nbsp &nbsp &nbsp  15  Other glycan degradation 0.000 &nbsp &nbsp &nbsp
   yddH   ecocyc   kegg   phen   96  rarA  ecocyc   kegg  9.2 0.0 &nbsp &nbsp &nbsp
 &nbsp &nbsp &nbsp  13  Chlorocyclohexane and chlorobenzene degradation 0.000 &nbsp &nbsp &nbsp
   yddJ   ecocyc   kegg   phen   121  yeaG  ecocyc   kegg  13.7 0.0  26  Hypoxanthine  C00262  5.572
  8  C5-Branched dibasic acid metabolism 8e-15  9  Biosynthesis of siderophore group nonribosomal peptides 0.000  6  2,3-Dihydro-2,3-dihydroxybenzoate 0.000 0.000
   yddK   ecocyc   kegg   phen   77  ybbV  ecocyc   kegg  10.3 0.0  4  L-tartrate  C00898  4.126
 &nbsp &nbsp &nbsp  5  Terpenoid backbone biosynthesis 0.001  6  L-Methionine Sulfoxide 0.000 0.000
   yddL   ecocyc   kegg   phen   236  yneG  ecocyc   kegg  10.9 0.0  15  Anthranilate  C00108  4.208
  3  Fatty acid biosynthesis 2e-07  10  Chlorocyclohexane and chlorobenzene degradation 0.000  18  L-Homoserine 0.002 1.000
   yddM   ecocyc   kegg   phen   394  ygcS  ecocyc   kegg  11.4 0.0 &nbsp &nbsp &nbsp
 &nbsp &nbsp &nbsp  4  Chlorocyclohexane and chlorobenzene degradation 0.000 &nbsp &nbsp &nbsp
   yddW   ecocyc   kegg   phen   195  ygeD  ecocyc   kegg  12.0 0.0  2  1,4-Dihydroxy-2-naphthoate  C03657  3.764
 &nbsp &nbsp &nbsp  9  Lysine degradation 0.001  16  Nitrous oxide 0.000 0.000
   ydeA   ecocyc   kegg   phen   110  lepA  ecocyc   kegg  10.8 0.0  9  2-Succinyl-6-hydroxy-2,4-cyclohexadiene-1-carboxylate  C05817  7.031
  2  Ubiquinone and other terpenoid-quinone biosynthesis 0  5  ABC transporters 0.000  12  Spermidine 0.000 0.000
   ydeE   ecocyc   kegg   phen   75  yqiK  ecocyc   kegg  7.8 0.0 &nbsp &nbsp &nbsp
 &nbsp &nbsp &nbsp  5  D-Glutamine and D-glutamate metabolism 0.000 &nbsp &nbsp &nbsp
   ydeI   ecocyc   kegg   phen   119  ydhW  ecocyc   kegg  7.7 0.0  9  Shikimate 5-phosphate  C03175  4.512
  7  Toluene degradation 2e-05  5  Oxidative phosphorylation 0.000  9  D-Glycerate 2-phosphate 0.000 0.000
   ydeJ   ecocyc   kegg   phen   94  cusR  ecocyc   kegg  13.5 0.0  14  2-Acyl-sn-glycero-3-phosphoethanolamine (n-C18:1)  C05973  5.612
  2  Cysteine and methionine metabolism 0.0003  10  Citrate cycle (TCA cycle) 0.000  3  Succinate 0.000 0.000
   ydeK   ecocyc   kegg   phen   78  ymfH  ecocyc   kegg  6.7 0.0 &nbsp &nbsp &nbsp
 &nbsp &nbsp &nbsp  3  Phenylalanine, tyrosine and tryptophan biosynthesis 0.001 &nbsp &nbsp &nbsp
   ydeM   ecocyc   kegg   phen   122  yfcS  ecocyc   kegg  6.9 0.0  4  4-Aminobenzoate  C00568  3.984
 &nbsp &nbsp &nbsp  1  ABC transporters 0.000  7  L-Leucine 0.000 0.000
   ydeN   ecocyc   kegg   phen   382  ydfO  ecocyc   kegg  8.8 0.0  1  &nbsp &nbsp
  4  Riboflavin metabolism 4e-05  3  Mismatch repair 0.002  21  glucosyl-O-acetyl-rhamanosyl-N-acetylglucosamyl-undecaprenyl diphosphate 0.000 0.000
   ydeO   ecocyc   kegg   phen   318  hinT  ecocyc   kegg  9.7 0.0 &nbsp &nbsp &nbsp
  1  Biosynthesis of secondary metabolites 0 &nbsp &nbsp &nbsp &nbsp &nbsp &nbsp
   ydeP   ecocyc   kegg   phen   234  hinT  ecocyc   kegg  8.8 0.0 &nbsp &nbsp &nbsp
  1  Porphyrin and chlorophyll metabolism 0.0006 &nbsp &nbsp &nbsp &nbsp &nbsp &nbsp
   ydeR   ecocyc   kegg   phen   241  yedY  ecocyc   kegg  7.1 0.0 &nbsp &nbsp &nbsp
 &nbsp &nbsp &nbsp  1  Lipopolysaccharide biosynthesis 0.009 &nbsp &nbsp &nbsp
   ydeS   ecocyc   kegg   phen   124  yjhG  ecocyc   kegg  11.2 0.0 &nbsp &nbsp &nbsp
 &nbsp &nbsp &nbsp  8  Porphyrin and chlorophyll metabolism 0.001 &nbsp &nbsp &nbsp
   ydeT   ecocyc   kegg   phen   102  cirA  ecocyc   kegg  9.3 0.0  18  lipoyl-AMP  C16238  3.626
  5  Thiamine metabolism 0.0002  9  Toluene degradation 0.000  5  Fumarate 0.000 0.000
   ydfA   ecocyc   kegg   phen   133  uhpB  ecocyc   kegg  14.6 0.0  21  Hypoxanthine  C00262  6.855
  13  alpha-Linolenic acid metabolism 5e-09  3  Two-component system 0.000  1  Succinate 0.006 0.000
   ydfC   ecocyc   kegg   phen   114  ynfB  ecocyc   kegg  10.4 0.0 &nbsp &nbsp &nbsp
  1  Biosynthesis of secondary metabolites 0  7  RNA polymerase 0.000 &nbsp &nbsp &nbsp
   ydfD   ecocyc   kegg   phen   150  norW  ecocyc   kegg  12.4 0.0  32  cyclopropane phosphatidylethanolamine (dihexadec-9,10-cyclo-anoyl, n-C16:0 cyclo)  cyclopropane phosphatidylethanolamine (dihexadec-9,10-cyclo-anoyl, n-C16:0 cyclo)  4.443
  19  D-Alanine metabolism 5e-08  5  Toluene degradation 0.000  5  (-)-Ureidoglycolate 0.000 0.000
   ydfE   ecocyc   kegg   phen   177  cspE  ecocyc   kegg  9.8 0.0  1  Butanal  C01412  3.552
  1  Biosynthesis of secondary metabolites 0  3  Riboflavin metabolism 0.000  5  2-Amino-4-hydroxy-6-(erythro-1,2,3-trihydroxypropyl)dihydropteridine triphosphate 0.000 0.000
   ydfJ   ecocyc   kegg   phen   184  xerC  ecocyc   kegg  8.3 0.0  9  L-Histidinol phosphate  C01100  3.790
  6  Nitrotoluene degradation 0.0007 &nbsp &nbsp &nbsp  6  glucosyl-O-acetyl-rhamanosyl-N-acetylglucosamyl-undecaprenyl diphosphate 0.000 0.000
   ydfO   ecocyc   kegg   phen   755  yebW  ecocyc   kegg  11.4 0.0 &nbsp &nbsp &nbsp
 &nbsp &nbsp &nbsp  2  Mismatch repair 0.004 &nbsp &nbsp &nbsp
   ydfR   ecocyc   kegg   phen   88  ygaH  ecocyc   kegg  8.9 0.0  4  cyclopropane phosphatidylethanolamine (dihexadec-9,10-cyclo-anoyl, n-C16:0 cyclo)  cyclopropane phosphatidylethanolamine (dihexadec-9,10-cyclo-anoyl, n-C16:0 cyclo)  4.566
 &nbsp &nbsp &nbsp  1  Two-component system 0.001  3  L-Cysteine 0.000 0.000
   ydfU   ecocyc   kegg   phen   100  qor  ecocyc   kegg  11.3 0.0  22  N-Acetyl-L-glutamate  C00624  6.595
  6  Lysine degradation 2e-07  6  RNA polymerase 0.000  5  D-Fructose 6-phosphate 0.002 0.000
   ydfV   ecocyc   kegg   phen   88  sgbH  ecocyc   kegg  8.6 0.0  4  Pyridoxamine  C00534  3.923
  5  Lipoic acid metabolism 0  8  Butanoate metabolism 0.001  4  L-Arabinose 0.000 0.000
   ydfW   ecocyc   kegg   phen   204  ybbP  ecocyc   kegg  12.6 0.0  4  Sedoheptulose 1,7-bisphosphate  C00447  4.385
 &nbsp &nbsp &nbsp  4  RNA degradation 0.004  9  bis-molybdenum cofactor 0.000 0.000
   ydfX   ecocyc   kegg   phen   140  yecE  ecocyc   kegg  12.0 0.0  47  D-Glucosamine 1-phosphate  C06156  7.409
  14  Lysine degradation 2e-12  7  Two-component system 0.000  15  D-Glycerate 2-phosphate 0.000 0.000
   ydfZ   ecocyc   kegg   phen   146  yiaU  ecocyc   kegg  11.3 0.0  8  gamma-glutamyl-gamma aminobutyric acid  C15767  3.614
  6  Lysine degradation 2e-05  4  Pantothenate and CoA biosynthesis 0.003  5  2-Oxobutanoate 0.000 0.000
   ydgA   ecocyc   kegg   phen   119  atoS  ecocyc   kegg  12.4 0.0 &nbsp &nbsp &nbsp
 &nbsp &nbsp &nbsp  8  RNA polymerase 0.000 &nbsp &nbsp &nbsp
   ydgC   ecocyc   kegg   phen   159  fiu  ecocyc   kegg  13.7 0.0  3  Ethanol  C00469  4.000
  6  Butanoate metabolism 1e-05  2  Two-component system 0.001  7  D-Glycerate 2-phosphate 0.000 0.000
   ydgD   ecocyc   kegg   phen   160  yfdK  ecocyc   kegg  16.1 0.0  50  8-Amino-7-oxononanoate  C01092  6.059
  8  Arachidonic acid metabolism 0 &nbsp &nbsp &nbsp  5  Undecaprenyl phosphate 0.000 0.000
   ydgH   ecocyc   kegg   phen   102  yebE  ecocyc   kegg  10.0 0.0  6  2-Oxo-3-hydroxy-4-phosphobutanoate  C06054  4.440
  2  Selenoamino acid metabolism 2e-06  3  Two-component system 0.000  5  1-deoxy-D-xylulose 5-phosphate 0.000 0.000
   ydgI   ecocyc   kegg   phen   123  ddpC  ecocyc   kegg  16.5 0.0  40  gamma-butyrobetaine  C01181  5.620
  16  Lysine degradation 6e-07  3  ABC transporters 0.000  11  L-Arabinose 0.000 0.000
   ydgJ   ecocyc   kegg   phen   339  ydhV  ecocyc   kegg  14.2 0.0  18  Glycolaldehyde  C00266  5.632
  21  Valine, leucine and isoleucine degradation 1e-06  3  Aminoacyl-tRNA biosynthesis 0.002  17  2-Octaprenyl-6-methoxyphenol 0.000 0.000
   ydgK   ecocyc   kegg   phen   144  yiaU  ecocyc   kegg  10.9 0.0  12  Cobinamide  C05774  7.405
  2  Arachidonic acid metabolism 0  2  Protein export 0.002 &nbsp &nbsp &nbsp
   ydhB   ecocyc   kegg   phen   481  pbl  ecocyc   kegg  11.6 0.0  14  D-Glucosamine 1-phosphate  C06156  6.418
  5  C5-Branched dibasic acid metabolism 5e-10  1  Mismatch repair 0.007  18  glucosyl-O-acetyl-rhamanosyl-N-acetylglucosamyl-undecaprenyl diphosphate 0.000 0.000
   ydhC   ecocyc   kegg   phen   107  ybeU  ecocyc   kegg  13.4 0.0  9  4-Hydroxy-L-threonine  C06056  3.567
  11  ABC transporters 0.0009  8  Chlorocyclohexane and chlorobenzene degradation 0.000  5  L-ascorbate-6-phosphate 0.000 0.000
   ydhF   ecocyc   kegg   phen   101  rsgA  ecocyc   kegg  11.9 0.0  35  gamma-hydroxybutyrate  C00989  3.976
  12  Benzoate degradation 9e-07  12  Chlorocyclohexane and chlorobenzene degradation 0.000  10  Adenosine 5'-phosphosulfate 0.000 0.000
   ydhI   ecocyc   kegg   phen   90  proB  ecocyc   kegg  16.5 0.0  13  L-tartrate  C00898  7.277
 &nbsp &nbsp &nbsp  5  D-Glutamine and D-glutamate metabolism 0.001  3  O-Acetyl-L-serine 0.000 0.000
   ydhK   ecocyc   kegg   phen   101  ycaK  ecocyc   kegg  15.4 0.0  33  Formaldehyde  C00067  6.543
  12  Glyoxylate and dicarboxylate metabolism 0.0002  2  D-Glutamine and D-glutamate metabolism 0.001  2  potassium 0.003 0.000
   ydhL   ecocyc   kegg   phen   557  yegR  ecocyc   kegg  12.0 0.0  5  3-(3-hydroxy-phenyl)propionate  C11457  3.525
  7  Cyanoamino acid metabolism 4e-05  9  Chlorocyclohexane and chlorobenzene degradation 0.000  46  2,3-Dihydro-2,3-dihydroxybenzoate 0.000 0.000
   ydhO   ecocyc   kegg   phen   335  yegX  ecocyc   kegg  9.8 0.0 &nbsp &nbsp &nbsp
 &nbsp &nbsp &nbsp  2  Bisphenol degradation 0.008 &nbsp &nbsp &nbsp
   ydhS   ecocyc   kegg   phen   113  ypjF  ecocyc   kegg  14.0 0.0  22  Lipoate  C00725  4.396
  7  Oxidative phosphorylation 7e-05  6  Two-component system 0.000  4  D-Lactate 0.000 0.000
   ydhT   ecocyc   kegg   phen   133  yebE  ecocyc   kegg  15.1 0.0  31  cyclopropane phosphatidylglycerol (dihexadec-9,10-cyclo-anoyl, n-C16:0 cyclo)  cyclopropane phosphatidylglycerol (dihexadec-9,10-cyclo-anoyl, n-C16:0 cyclo)  4.386
  7  Arginine and proline metabolism 8e-05  8  Nitrotoluene degradation 0.000  4  S-Adenosyl-L-methionine 0.005 0.000
   ydhU   ecocyc   kegg   phen   105  flhA  ecocyc   kegg  10.2 0.0  2  Thymine  C00178  3.628
  1  Riboflavin metabolism 0  9  Bacterial secretion system 0.000  1  2-Oxobutanoate 0.003 0.000
   ydhV   ecocyc   kegg   phen   172  ydgJ  ecocyc   kegg  14.2 0.0  4  Biotin  C00120  -5.467
  3  Biotin metabolism 1e-05  3  Sphingolipid metabolism 0.002  7  (S)-2-Aceto-2-hydroxybutanoate 0.000 0.000
   ydhW   ecocyc   kegg   phen   173  yeiE  ecocyc   kegg  12.6 0.0  21  cyclopropane phosphatidylethanolamine (dihexadec-9,10-cyclo-anoyl, n-C16:0 cyclo)  cyclopropane phosphatidylethanolamine (dihexadec-9,10-cyclo-anoyl, n-C16:0 cyclo)  3.615
  6  Biosynthesis of unsaturated fatty acids 7e-08  5  Two-component system 0.000  12  silver 0.000 0.000
   ydhX   ecocyc   kegg   phen   210  yhiK  ecocyc   kegg  13.7 0.0  17  2-Acyl-sn-glycero-3-phosphoethanolamine (n-C18:0)  C05973  5.414
  16  Peptidoglycan biosynthesis 5e-05  4  Glycerolipid metabolism 0.002  22  Glycerol 2-phosphate 0.000 0.000
   ydhY   ecocyc   kegg   phen   91  ybjH  ecocyc   kegg  7.3 0.0 &nbsp &nbsp &nbsp
 &nbsp &nbsp &nbsp  6  Pantothenate and CoA biosynthesis 0.001 &nbsp &nbsp &nbsp
   ydhZ   ecocyc   kegg   phen   256  ydiI  ecocyc   kegg  7.9 0.0  4  2-hexadec-9-enoyl-sn-glycerol 3-phosphate  C03974  3.478
  5  Glutathione metabolism 0.0003 &nbsp &nbsp &nbsp  10  glucosyl-O-acetyl-rhamanosyl-N-acetylglucosamyl-undecaprenyl diphosphate 0.000 0.000
   ydiE   ecocyc   kegg   phen   106  atoS  ecocyc   kegg  9.5 0.0 &nbsp &nbsp &nbsp
  8  Nitrotoluene degradation 2e-06  3  Glycolysis / Gluconeogenesis 0.001 &nbsp &nbsp &nbsp
   ydiF   ecocyc   kegg   phen   156  yehW  ecocyc   kegg  10.5 0.0  1  L-Asparagine  C00152  -4.069
  8  Nitrogen metabolism 1e-05  2  Pentose and glucuronate interconversions 0.002  7  uridine 5''-diphospho-{beta}-4-deoxy-4-formamido-L-arabinose 0.000 0.000
   ydiH   ecocyc   kegg   phen   110  ydhW  ecocyc   kegg  10.1 0.0  43  Hydroquinone  C00530  6.021
  20  Pyrimidine metabolism 3e-06  4  Two-component system 0.000  5  D-Glycerate 2-phosphate 0.000 0.000
   ydiI   ecocyc   kegg   phen   168  yeiA  ecocyc   kegg  13.2 0.0  11  Formaldehyde  C00067  4.404
  11  Histidine metabolism 0.0001  2  Base excision repair 0.002  12  [4Fe-4S] iron-sulfur cluster 0.000 0.000
   ydiJ   ecocyc   kegg   phen   121  sufC  ecocyc   kegg  10.4 0.0  39  trans-Aconitate  C02341  7.941
  10  C5-Branched dibasic acid metabolism 6e-14  2  Biosynthesis of siderophore group nonribosomal peptides 0.000  14  2,3-Dihydro-2,3-dihydroxybenzoate 0.000 0.000
   ydiK   ecocyc   kegg   phen   221  hinT  ecocyc   kegg  8.5 0.0  3  Butyrate (n-C4:0)  C00246  3.546
  2  Butanoate metabolism 0 &nbsp &nbsp &nbsp  10  glucosyl-O-acetyl-rhamanosyl-N-acetylglucosamyl-undecaprenyl diphosphate 0.000 0.000
   ydiM   ecocyc   kegg   phen   114  yddW  ecocyc   kegg  10.5 0.0 &nbsp &nbsp &nbsp
  1  Fatty acid metabolism 2e-06  2  Terpenoid backbone biosynthesis 0.003 &nbsp &nbsp &nbsp
   ydiP   ecocyc   kegg   phen   137  ydjA  ecocyc   kegg  12.9 0.0  2  L-Isoleucine  C00407  4.501
  6  Valine, leucine and isoleucine degradation 1e-08  4  Phosphonate and phosphinate metabolism 0.001  6  tungstate 0.000 0.000
   ydiQ   ecocyc   kegg   phen   407  ynjB  ecocyc   kegg  12.2 0.0  4  N-Carbamoyl-L-aspartate  C00438  3.881
  11  Inositol phosphate metabolism 5e-06  1  Glycerolipid metabolism 0.004  12  2-Octaprenyl-6-methoxyphenol 0.000 0.000
   ydiR   ecocyc   kegg   phen   123  ydhP  ecocyc   kegg  13.6 0.0  3  2-Oxo-3-hydroxy-4-phosphobutanoate  C06054  4.090
 &nbsp &nbsp &nbsp  8  Fatty acid biosynthesis 0.001  12  o-Succinylbenzoate 0.000 0.000
   ydiS   ecocyc   kegg   phen   100  yegE  ecocyc   kegg  9.2 0.0 &nbsp &nbsp &nbsp
 &nbsp &nbsp &nbsp &nbsp &nbsp &nbsp &nbsp &nbsp &nbsp
   ydiT   ecocyc   kegg   phen   131  malG  ecocyc   kegg  12.7 0.0 &nbsp &nbsp &nbsp
 &nbsp &nbsp &nbsp  5  Protein export 0.001 &nbsp &nbsp &nbsp
   ydiV   ecocyc   kegg   phen   115  ygcH  ecocyc   kegg  9.2 0.0  2  2-Octaprenyl-6-methoxyphenol  C05812  3.824
 &nbsp &nbsp &nbsp  2  Pyruvate metabolism 0.005  6  undecaprenyl phosphate-4-amino-4-deoxy-L-arabinose 0.000 0.000
   ydiY   ecocyc   kegg   phen   139  yiaU  ecocyc   kegg  9.7 0.0  12  2-octadec-11-enoyl-sn-glycerol 3-phosphate  C03974  4.246
  2  Sphingolipid metabolism 3e-05  6  Flagellar assembly 0.001  6  2-Oxobutanoate 0.000 0.000
   ydiZ   ecocyc   kegg   phen   248  holD  ecocyc   kegg  13.6 0.0  1  L-Tryptophan  C00078  3.475
  2  Dioxin degradation 1e-05  19  Chlorocyclohexane and chlorobenzene degradation 0.000  34  5-amino-1-(5-phospho-D-ribosyl)imidazole-4-carboxylate 0.000 0.000
   ydjE   ecocyc   kegg   phen   109  ftn  ecocyc   kegg  7.6 0.0  2  Cobinamide  C05774  3.702
  1  Porphyrin and chlorophyll metabolism 0  3  ABC transporters 0.000  17  Maltoheptaose 0.000 0.000
   ydjF   ecocyc   kegg   phen   81  ydfI  ecocyc   kegg  15.2 0.0  4  UDPgalactose  C00052  3.520
  1  Biosynthesis of secondary metabolites 0  7  Nitrotoluene degradation 0.000  6  D-Tagaturonate 0.000 0.000
   ydjG   ecocyc   kegg   phen   212  sbmC  ecocyc   kegg  12.2 0.0  10  glycogen  C00182  4.100
  5  Benzoate degradation 1e-07  9  Chlorocyclohexane and chlorobenzene degradation 0.000  15  Adenosine 5'-phosphosulfate 0.000 0.000
   ydjH   ecocyc   kegg   phen   230  yehP  ecocyc   kegg  12.3 0.0  3  CDP-1,2-ditetradec-7-enoylglycerol  C00269  3.856
  3  Biotin metabolism 7e-06  2  Bisphenol degradation 0.004  4  L-Threonine 0.000 0.000
   ydjI   ecocyc   kegg   phen   70  ogt  ecocyc   kegg  22.0 0.0  61  N-Acetylneuraminate  C00270  9.019
  5  Lysine degradation 1e-09  10  Inositol phosphate metabolism 0.000  2  2-Octaprenyl-3-methyl-6-methoxy- 1,4-benzoquinol 0.000 0.000
   ydjJ   ecocyc   kegg   phen   74  rffD  ecocyc   kegg  17.7 0.0  11  1-dodecanoyl-sn-glycerol 3-phosphate  C00681  4.938
  19  D-Alanine metabolism 2e-07  3  Riboflavin metabolism 0.002  7  D-Galactarate 0.000 0.000
   ydjK   ecocyc   kegg   phen   114  dkgA  ecocyc   kegg  10.1 0.0  3  gamma-butyrobetaine  C01181  7.096
 &nbsp &nbsp &nbsp  5  Bisphenol degradation 0.001  9  ethanesulfonate 0.000 0.000
   ydjL   ecocyc   kegg   phen   126  yddW  ecocyc   kegg  10.5 0.0  3  gamma-glutamyl-putrescine  C15699  3.666
  3  Riboflavin metabolism 4e-05  6  beta-Alanine metabolism 0.000  6  Arbutin 6-phosphate 0.000 0.000
   ydjM   ecocyc   kegg   phen   168  yhaI  ecocyc   kegg  9.3 0.0  14  4-Phospho-L-aspartate  C03082  5.025
  12  Valine, leucine and isoleucine degradation 5e-06  13  Benzoate degradation 0.000  24  D-Glucosamine 6-phosphate 0.007 1.000
   ydjN   ecocyc   kegg   phen   94  nrdD  ecocyc   kegg  7.8 0.0  3  N-Succinyl-2-L-amino-6-oxoheptanedioate  C04462  3.941
 &nbsp &nbsp &nbsp  10  ABC transporters 0.000  24  D-Alanyl-D-alanine 0.000 0.000
   ydjX   ecocyc   kegg   phen   137  tdk  ecocyc   kegg  14.9 0.0  86  N-Acetyl-L-glutamate  C00624  9.075
  12  Aminoacyl-tRNA biosynthesis 2e-06  2  Phosphonate and phosphinate metabolism 0.001  8  tungstate 0.000 0.000
   ydjY   ecocyc   kegg   phen   366  yebU  ecocyc   kegg  10.2 0.0  1  &nbsp &nbsp
 &nbsp &nbsp &nbsp  3  Mismatch repair 0.002  17  glucosyl-O-acetyl-rhamanosyl-N-acetylglucosamyl-undecaprenyl diphosphate 0.000 0.000
   yeaC   ecocyc   kegg   phen   106  lldD  ecocyc   kegg  12.3 0.0  12  N-Acetyl-L-glutamate  C00624  4.818
  4  Tryptophan metabolism 5e-08  2  Two-component system 0.000  6  silver 0.000 0.000
   yeaD   ecocyc   kegg   phen   306  yjhS  ecocyc   kegg  8.1 0.0  2  crotonobetaine  C04114  3.576
  4  Porphyrin and chlorophyll metabolism 5e-05  1  Phosphonate and phosphinate metabolism 0.006  19  O-acetyl-rhamanosyl-N-acetylglucosamyl-undecaprenyl diphosphate 0.000 0.000
   yeaG   ecocyc   kegg   phen   116  yddJ  ecocyc   kegg  13.7 0.0  45  Hypoxanthine  C00262  16.274
  11  Microbial metabolism in diverse environments 6e-08  5  Mismatch repair 0.001  10  5-Methyltetrahydrofolate 0.000 0.000
   yeaH   ecocyc   kegg   phen   276  wbbK  ecocyc   kegg  8.6 0.0  15  D-Glucosamine 1-phosphate  C06156  4.458
  6  Naphthalene degradation 2e-07 &nbsp &nbsp &nbsp  10  glucosyl-O-acetyl-rhamanosyl-N-acetylglucosamyl-undecaprenyl diphosphate 0.000 0.000
   yeaI   ecocyc   kegg   phen   82  yahI  ecocyc   kegg  9.7 0.0  3  cyclopropane phosphatidylethanolamine (dihexadec-9,10-cyclo-anoyl, n-C16:0 cyclo)  cyclopropane phosphatidylethanolamine (dihexadec-9,10-cyclo-anoyl, n-C16:0 cyclo)  3.736
 &nbsp &nbsp &nbsp  7  Taurine and hypotaurine metabolism 0.000  7  Oxaloacetate 0.000 0.000
   yeaJ   ecocyc   kegg   phen   204  yegX  ecocyc   kegg  11.0 0.0  14  Oxidized glutathione  C00127  5.158
  16  Peptidoglycan biosynthesis 6e-05  1  Phosphonate and phosphinate metabolism 0.003  6  D-Fructose 1,6-bisphosphate 0.001 0.000
   yeaK   ecocyc   kegg   phen   152  yohK  ecocyc   kegg  14.8 0.0  12  tetradecanoate (n-C14:0)  C06424  10.964
  2  Arachidonic acid metabolism 0  5  Two-component system 0.004  3  potassium 0.003 0.000
   yeaL   ecocyc   kegg   phen   254  ybjR  ecocyc   kegg  11.5 0.0 &nbsp &nbsp &nbsp
  3  Novobiocin biosynthesis 7e-05  12  Lysine degradation 0.000 &nbsp &nbsp &nbsp
   yeaM   ecocyc   kegg   phen   88  malY  ecocyc   kegg  7.4 0.0 &nbsp &nbsp &nbsp
 &nbsp &nbsp &nbsp  4  Lipopolysaccharide biosynthesis 0.004 &nbsp &nbsp &nbsp
   yeaN   ecocyc   kegg   phen   372  yphB  ecocyc   kegg  14.5 0.0  9  Butanal  C01412  3.838
  14  Microbial metabolism in diverse environments 5e-10 &nbsp &nbsp &nbsp  13  glucosyl-O-acetyl-rhamanosyl-N-acetylglucosamyl-undecaprenyl diphosphate 0.000 0.000
   yeaO   ecocyc   kegg   phen   20  yagK  ecocyc   kegg  32.8 0.0  22  N-Acetylneuraminate  C00270  15.359
  3  alpha-Linolenic acid metabolism 2e-09  3  Inositol phosphate metabolism 0.000  2  Glyceraldehyde 3-phosphate 0.000 0.000
   yeaP   ecocyc   kegg   phen   323  ydiQ  ecocyc   kegg  10.0 0.0  2  p-Cresol  C01468  4.468
  8  Microbial metabolism in diverse environments 4e-15  3  Mismatch repair 0.006  12  glucosyl-O-acetyl-rhamanosyl-N-acetylglucosamyl-undecaprenyl diphosphate 0.000 0.000
   yeaQ   ecocyc   kegg   phen   95  yddV  ecocyc   kegg  13.4 0.0  11  Lactose  C00243  34.288
  3  Phosphonate and phosphinate metabolism 2e-05 &nbsp &nbsp &nbsp  3  glucosyl-O-acetyl-rhamanosyl-N-acetylglucosamyl-undecaprenyl diphosphate 0.000 0.000
   yeaR   ecocyc   kegg   phen   100  chbG  ecocyc   kegg  7.0 0.0  5  Adenosine  C00212  3.627
  7  Microbial metabolism in diverse environments 7e-09  6  Glycolysis / Gluconeogenesis 0.000  2  Arbutin 6-phosphate 0.000 0.000
   yeaV   ecocyc   kegg   phen   146  nmpC  ecocyc   kegg  9.9 0.0  19  Pyridoxal  C00250  3.890
  2  Porphyrin and chlorophyll metabolism 0.0006  5  Limonene and pinene degradation 0.001  6  Citrate 0.000 0.000
   yeaX   ecocyc   kegg   phen   127  wcaF  ecocyc   kegg  10.3 0.0  3  meso-2,6-Diaminoheptanedioate  C00680  3.595
 &nbsp &nbsp &nbsp  6  Bacterial secretion system 0.001  20  Acetoacetate 0.000 0.000
   yeaY   ecocyc   kegg   phen   125  mltB  ecocyc   kegg  12.6 0.0  13  Hydroxypyruvate  C00168  4.607
  9  Phenylalanine metabolism 6e-10  5  Toluene degradation 0.003  2  Succinate 0.003 0.000
   yebA   ecocyc   kegg   phen   214  yjjM  ecocyc   kegg  9.6 0.0 &nbsp &nbsp &nbsp
 &nbsp &nbsp &nbsp  4  Chlorocyclohexane and chlorobenzene degradation 0.000 &nbsp &nbsp &nbsp
   yebB   ecocyc   kegg   phen   213  yoaB  ecocyc   kegg  9.5 0.0  3  Glycerophosphoserine  Glycerophosphoserine  -3.706
 &nbsp &nbsp &nbsp  3  Bisphenol degradation 0.003  7  L-fuculose 0.000 0.000
   yebC   ecocyc   kegg   phen   76  yafQ  ecocyc   kegg  14.7 0.0  4  (S)-Dihydroorotate  C00337  3.526
  4  Limonene and pinene degradation 0.0005  6  Chlorocyclohexane and chlorobenzene degradation 0.000 &nbsp &nbsp &nbsp
   yebE   ecocyc   kegg   phen   216  atoS  ecocyc   kegg  18.1 0.0  37  cyclopropane phosphatidylglycerol (dihexadec-9,10-cyclo-anoyl, n-C16:0 cyclo)  cyclopropane phosphatidylglycerol (dihexadec-9,10-cyclo-anoyl, n-C16:0 cyclo)  5.675
  8  Purine metabolism 6e-08  11  RNA polymerase 0.000  10  Succinate 0.000 0.000
   yebF   ecocyc   kegg   phen   79  yhbQ  ecocyc   kegg  8.3 0.0  5  (R)-Pantothenate  C00864  4.211
  6  Pantothenate and CoA biosynthesis 3e-07  14  Chlorocyclohexane and chlorobenzene degradation 0.000  3  ADP-L-glycero-D-manno-heptose 0.000 0.000
   yebK   ecocyc   kegg   phen   97  yaiX  ecocyc   kegg  10.4 0.0  2  psicoselysine  psicoselysine  -4.941
  1  Biosynthesis of secondary metabolites 0  3  Tryptophan metabolism 0.002  12  bis-molybdopterin guanine dinucleotide 0.000 0.000
   yebN   ecocyc   kegg   phen   220  yddM  ecocyc   kegg  9.4 0.0 &nbsp &nbsp &nbsp
  1  Glycerophospholipid metabolism 0  4  Chlorocyclohexane and chlorobenzene degradation 0.000 &nbsp &nbsp &nbsp
   yebO   ecocyc   kegg   phen   148  yobD  ecocyc   kegg  15.1 0.0  34  N-Acetylneuraminate  C00270  4.904
  14  Arachidonic acid metabolism 9e-16  5  Citrate cycle (TCA cycle) 0.000  6  potassium 0.000 0.000
   yebS   ecocyc   kegg   phen   193  glyS  ecocyc   kegg  14.5 0.0  10  Uridine  C00299  4.189
  9  Naphthalene degradation 4e-07 &nbsp &nbsp &nbsp  7  5-Dehydro-4-deoxy-D-glucarate 0.000 0.000
   yebT   ecocyc   kegg   phen   104  ybaO  ecocyc   kegg  6.8 0.0  8  Sedoheptulose 1,7-bisphosphate  C00447  3.850
  5  Microbial metabolism in diverse environments 0  9  Chlorocyclohexane and chlorobenzene degradation 0.000  13  L-Homocysteine 0.000 0.000
   yebV   ecocyc   kegg   phen   437  yegX  ecocyc   kegg  9.8 0.0 &nbsp &nbsp &nbsp
 &nbsp &nbsp &nbsp  1  Glycerolipid metabolism 0.005 &nbsp &nbsp &nbsp
   yebW   ecocyc   kegg   phen   363  ydfO  ecocyc   kegg  11.4 0.0 &nbsp &nbsp &nbsp
 &nbsp &nbsp &nbsp  5  Mismatch repair 0.002 &nbsp &nbsp &nbsp
   yebY   ecocyc   kegg   phen   174  cusR  ecocyc   kegg  17.5 0.0  40  cyclopropane phosphatidylethanolamine (dihexadec-9,10-cyclo-anoyl, n-C16:0 cyclo)  cyclopropane phosphatidylethanolamine (dihexadec-9,10-cyclo-anoyl, n-C16:0 cyclo)  6.426
  7  Arachidonic acid metabolism 0  8  RNA polymerase 0.000  10  Decanoyl-ACP (n-C10:0ACP) 0.000 0.000
   yebZ   ecocyc   kegg   phen   188  yfeX  ecocyc   kegg  14.1 0.0  9  2-Acyl-sn-glycero-3-phosphoglycerol (n-C18:1)  2-Acyl-sn-glycero-3-phosphoglycerol (n-C18:1)  5.217
  1  Arachidonic acid metabolism 0  3  Toluene degradation 0.001  9  Deoxyuridine 0.004 0.000
   yecA   ecocyc   kegg   phen   65  yedF  ecocyc   kegg  21.6 0.0  17  D-Lactaldehyde  C00937  16.869
  10  Toluene degradation 5e-15  6  Pantothenate and CoA biosynthesis 0.000  1  Sulfite 0.004 0.000
   yecC   ecocyc   kegg   phen   107  blr  ecocyc   kegg  7.7 0.0  4  Trehalose  C01083  4.172
  5  Galactose metabolism 7e-12  5  Chlorocyclohexane and chlorobenzene degradation 0.000  6  L-fuculose 0.000 0.000
   yecD   ecocyc   kegg   phen   249  yebU  ecocyc   kegg  9.1 0.0  1  Deoxyuridine  C00526  4.307
 &nbsp &nbsp &nbsp &nbsp &nbsp &nbsp  7  L-Threonine 0.000 0.000
   yecE   ecocyc   kegg   phen   131  ydfX  ecocyc   kegg  12.0 0.0  68  N-Acetyl-L-glutamate  C00624  5.545
  16  Lipoic acid metabolism 0  4  Two-component system 0.000  6  D-Glucose 6-phosphate 0.003 1.000
   yecF   ecocyc   kegg   phen   177  yccJ  ecocyc   kegg  12.8 0.0  7  8-Amino-7-oxononanoate  C01092  3.989
  3  Arachidonic acid metabolism 0  4  Two-component system 0.000  6  D-Glycerate 2-phosphate 0.000 0.000
   yecH   ecocyc   kegg   phen   188  atoS  ecocyc   kegg  19.7 0.0  71  cyclopropane phosphatidylglycerol (dihexadec-9,10-cyclo-anoyl, n-C16:0 cyclo)  cyclopropane phosphatidylglycerol (dihexadec-9,10-cyclo-anoyl, n-C16:0 cyclo)  10.724
  11  Fatty acid biosynthesis 8e-06  9  RNA polymerase 0.000  9  Decanoyl-ACP (n-C10:0ACP) 0.000 0.000
   yecJ   ecocyc   kegg   phen   117  pgpA  ecocyc   kegg  9.5 0.0  23  4-Amino-5-hydroxymethyl-2-methylpyrimidine  C01279  5.000
  8  Novobiocin biosynthesis 0.0004  8  Nucleotide excision repair 0.001  2  ADP-L-glycero-D-manno-heptose 0.000 0.000
   yecM   ecocyc   kegg   phen   240  yodB  ecocyc   kegg  10.5 0.0  9  dehydroglycine  C15809  4.228
  4  Biotin metabolism 1e-05  2  Phosphonate and phosphinate metabolism 0.004  12  tungstate 0.000 0.000
   yecN   ecocyc   kegg   phen   213  yehT  ecocyc   kegg  9.1 0.0  1  crotonobetaine  C04114  4.824
  2  Caprolactam degradation 0  3  Mismatch repair 0.001  3  D-Fructose 1,6-bisphosphate 0.000 0.000
   yecR   ecocyc   kegg   phen   188  yebE  ecocyc   kegg  15.1 0.0  15  1,2-Diacyl-sn-glycerol (dioctadec-11-enoyl, n-C18:1)  C00641  4.996
  4  Butanoate metabolism 3e-08  5  Citrate cycle (TCA cycle) 0.000  7  Succinate 0.000 0.000
   yecS   ecocyc   kegg   phen   128  ccmB  ecocyc   kegg  8.7 0.0 &nbsp &nbsp &nbsp
  1  Biosynthesis of secondary metabolites 0  9  Amino sugar and nucleotide sugar metabolism 0.002 &nbsp &nbsp &nbsp
   yecT   ecocyc   kegg   phen   470  yebU  ecocyc   kegg  10.3 0.0  4  Uridine  C00299  -3.596
  3  Biotin metabolism 7e-06  2  Mismatch repair 0.001  12  glucosyl-O-acetyl-rhamanosyl-N-acetylglucosamyl-undecaprenyl diphosphate 0.000 0.000
   yedA   ecocyc   kegg   phen   134  yeaA  ecocyc   kegg  12.4 0.0  20  Dodecanoate (n-C12:0)  C02679  6.407
  13  Fatty acid biosynthesis 4e-05  6  Cysteine and methionine metabolism 0.002  4  L-Serine 0.000 1.000
   yedD   ecocyc   kegg   phen   175  yceJ  ecocyc   kegg  16.8 0.0  36  Formaldehyde  C00067  7.886
  12  Microbial metabolism in diverse environments 1e-07  6  RNA polymerase 0.000  13  Decanoyl-ACP (n-C10:0ACP) 0.000 0.000
   yedE   ecocyc   kegg   phen   202  yebY  ecocyc   kegg  15.4 0.0  52  cyclopropane phosphatidylethanolamine (dihexadec-9,10-cyclo-anoyl, n-C16:0 cyclo)  cyclopropane phosphatidylethanolamine (dihexadec-9,10-cyclo-anoyl, n-C16:0 cyclo)  5.257
  9  Pyrimidine metabolism 6e-08  10  RNA polymerase 0.000  7  Succinate 0.000 0.000
   yedF   ecocyc   kegg   phen   25  mazG  ecocyc   kegg  22.2 0.0  49  D-Lactaldehyde  C00937  41.643
  17  Microbial metabolism in diverse environments 5e-10  2  Ubiquinone and other terpenoid-quinone biosynthesis 0.003 &nbsp &nbsp &nbsp
   yedI   ecocyc   kegg   phen   59  yfhJ  ecocyc   kegg  6.5 0.0  5  4-Phospho-L-aspartate  C03082  4.077
  8  Valine, leucine and isoleucine degradation 0.0004 &nbsp &nbsp &nbsp &nbsp &nbsp &nbsp
   yedJ   ecocyc   kegg   phen   116  ybdH  ecocyc   kegg  11.5 0.0  59  3',5'-Cyclic GMP  C00942  5.542
  13  Microbial metabolism in diverse environments 2e-11  4  Lipoic acid metabolism 0.003 &nbsp &nbsp &nbsp
   yedK   ecocyc   kegg   phen   205  yehA  ecocyc   kegg  11.0 0.0  2  UDP-D-glucuronate  C00167  3.597
  3  Biosynthesis of secondary metabolites 0  7  Pantothenate and CoA biosynthesis 0.000  16  4-Phospho-L-aspartate 0.000 0.000
   yedL   ecocyc   kegg   phen   85  emrD  ecocyc   kegg  12.1 0.0  4  Butanal  C01412  4.104
  1  Biosynthesis of secondary metabolites 0  1  Biosynthesis of unsaturated fatty acids 0.005  3  D-Mannose 6-phosphate 0.000 0.000
   yedM   ecocyc   kegg   phen   117  barA  ecocyc   kegg  10.6 0.0  25  1,2-Diacyl-sn-glycerol (dioctadec-11-enoyl, n-C18:1)  C00641  14.709
  5  Nitrogen metabolism 3e-05  3  Ribosome 0.001  1  D-Gluconate 0.000 0.000
   yedP   ecocyc   kegg   phen   146  yjeK  ecocyc   kegg  10.9 0.0  3  3-(3-hydroxy-phenyl)propionate  C11457  3.573
  5  Glycerophospholipid metabolism 9e-05  16  Chlorocyclohexane and chlorobenzene degradation 0.000  8  4-Phospho-L-aspartate 0.000 0.000
   yedQ   ecocyc   kegg   phen   186  yqhC  ecocyc   kegg  9.5 0.0 &nbsp &nbsp &nbsp
 &nbsp &nbsp &nbsp  5  Chlorocyclohexane and chlorobenzene degradation 0.000 &nbsp &nbsp &nbsp
   yedR   ecocyc   kegg   phen   146  ybgL  ecocyc   kegg  13.5 0.0  8  2-Oxo-3-hydroxy-4-phosphobutanoate  C06054  4.254
  3  Fatty acid biosynthesis 2e-05  6  Toluene degradation 0.000  6  molybdopterin 0.000 0.000
   yedV   ecocyc   kegg   phen   124  pflB  ecocyc   kegg  14.7 0.0  134  2-Acyl-sn-glycero-3-phosphoethanolamine (n-C18:0)  C05973  8.356
  26  Aminoacyl-tRNA biosynthesis 2e-07  17  Two-component system 0.000  17  Glyceraldehyde 3-phosphate 0.000 1.000
   yedW   ecocyc   kegg   phen   223  yrhA  ecocyc   kegg  8.2 0.0 &nbsp &nbsp &nbsp
 &nbsp &nbsp &nbsp  6  Chlorocyclohexane and chlorobenzene degradation 0.000 &nbsp &nbsp &nbsp
   yedY   ecocyc   kegg   phen   201  relE  ecocyc   kegg  7.1 0.0 &nbsp &nbsp &nbsp
  3  Novobiocin biosynthesis 4e-05 &nbsp &nbsp &nbsp &nbsp &nbsp &nbsp
   yedZ   ecocyc   kegg   phen   221  ybgT  ecocyc   kegg  13.0 0.0  23  Oxidized glutathione  C00127  6.040
  25  Arginine and proline metabolism 4e-07 &nbsp &nbsp &nbsp  11  5-[(5-phospho-1-deoxyribulos-1-ylamino)methylideneamino]-1-(5-phosphoribosyl)imidazole-4-carboxamide 0.000 0.000
   yeeA   ecocyc   kegg   phen   153  barA  ecocyc   kegg  10.3 0.0  23  D-Allose 6-phosphate  C02962  3.670
  18  D-Glutamine and D-glutamate metabolism 7e-06  1  Ribosome 0.002  1  D-Gluconate 0.000 0.000
   yeeD   ecocyc   kegg   phen   115  yejF  ecocyc   kegg  6.7 0.0 &nbsp &nbsp &nbsp
 &nbsp &nbsp &nbsp  1  Biosynthesis of unsaturated fatty acids 0.009 &nbsp &nbsp &nbsp
   yeeE   ecocyc   kegg   phen   185  sapD  ecocyc   kegg  15.1 0.0  44  Formaldehyde  C00067  6.600
  14  Glycine, serine and threonine metabolism 7e-06  7  D-Glutamine and D-glutamate metabolism 0.002  10  5-Phospho-beta-D-ribosylamine 0.000 0.000
   yeeF   ecocyc   kegg   phen   143  yoeF  ecocyc   kegg  13.6 0.0  4  3-Methyl-2-oxobutanoate  C00141  -4.156
  7  Valine, leucine and isoleucine degradation 0.0005  4  Bacterial secretion system 0.001  5  D-Carnitine 0.000 0.000
   yeeN   ecocyc   kegg   phen   38  ogt  ecocyc   kegg  24.0 0.0  7  N-Acetylneuraminate  C00270  5.970
  1  alpha-Linolenic acid metabolism 2e-10  3  Inositol phosphate metabolism 0.000  5  Dihydroxyacetone phosphate 0.000 0.000
   yeeO   ecocyc   kegg   phen   254  ydfO  ecocyc   kegg  9.5 0.0  4  Fumarate  C00122  4.569
  1  Biosynthesis of secondary metabolites 0  1  Base excision repair 0.009  5  glucosyl-O-acetyl-rhamanosyl-N-acetylglucosamyl-undecaprenyl diphosphate 0.000 0.000
   yeeR   ecocyc   kegg   phen   164  ynfM  ecocyc   kegg  13.4 0.0  11  Dodecanoly-phosphate (n-C12:0)  Dodecanoly-phosphate (n-C12:0)  3.890
  3  Starch and sucrose metabolism 1e-10  8  Homologous recombination 0.000  7  o-Succinylbenzoate 0.000 0.000
   yeeS   ecocyc   kegg   phen   120  ybeT  ecocyc   kegg  15.3 0.0  22  D-Mannitol 1-phosphate  C00644  4.305
  8  Fatty acid biosynthesis 5e-06  7  Nitrotoluene degradation 0.000  16  Decanoyl-ACP (n-C10:0ACP) 0.000 0.000
   yeeT   ecocyc   kegg   phen   230  ycdW  ecocyc   kegg  8.3 0.0  1  crotonobetaine  C04114  -4.163
  2  Caprolactam degradation 0 &nbsp &nbsp &nbsp  4  glucosyl-O-acetyl-rhamanosyl-N-acetylglucosamyl-undecaprenyl diphosphate 0.000 0.000
   yeeW   ecocyc   kegg   phen   148  yehA  ecocyc   kegg  12.9 0.0  40  phosphatidylethanolamine (dioctadec-11-enoyl, n-C18:1)  C00350  5.279
  6  Biotin metabolism 6e-05  10  Ascorbate and aldarate metabolism 0.000  9  L-Xylulose 5-phosphate 0.000 1.000
   yeeX   ecocyc   kegg   phen   81  yehW  ecocyc   kegg  8.2 0.0  6  3-Phospho-D-glycerate  C00197  6.122
  12  C5-Branched dibasic acid metabolism 2e-11  1  Polyketide sugar unit biosynthesis 0.006  1  Uracil 0.001 0.000
   yeeY   ecocyc   kegg   phen   277  cld  ecocyc   kegg  10.7 0.0 &nbsp &nbsp &nbsp
 &nbsp &nbsp &nbsp  4  Chlorocyclohexane and chlorobenzene degradation 0.000 &nbsp &nbsp &nbsp
   yeeZ   ecocyc   kegg   phen   149  yfbO  ecocyc   kegg  11.5 0.0  6  (S)-Propane-1,2-diol  C02917  4.066
  4  Naphthalene degradation 1e-05  5  Chlorocyclohexane and chlorobenzene degradation 0.000  1  L-Homoserine 0.000 0.000
   yegD   ecocyc   kegg   phen   165  yedZ  ecocyc   kegg  9.0 0.0  10  Glycine betaine  C00719  4.028
  6  Galactose metabolism 2e-11  1  Mismatch repair 0.003  14  glucosyl-O-acetyl-rhamanosyl-N-acetylglucosamyl-undecaprenyl diphosphate 0.000 0.000
   yegE   ecocyc   kegg   phen   141  ydgD  ecocyc   kegg  14.3 0.0  5  1,2-didodecanoyl-sn-glycerol 3-phosphate  C00416  3.758
  4  Arachidonic acid metabolism 0  3  Taurine and hypotaurine metabolism 0.001  11  UDP 0.000 0.000
   yegH   ecocyc   kegg   phen   345  ydfO  ecocyc   kegg  9.7 0.0  3  Biotin  C00120  -4.055
  4  Biotin metabolism 1e-05  4  Bisphenol degradation 0.008  10  L-Threonine 0.000 0.000
   yegI   ecocyc   kegg   phen   148  narH  ecocyc   kegg  6.9 0.0 &nbsp &nbsp &nbsp
  1  Biosynthesis of secondary metabolites 0  1  Lysine biosynthesis 0.006 &nbsp &nbsp &nbsp
   yegJ   ecocyc   kegg   phen   273  crcA  ecocyc   kegg  8.3 0.0 &nbsp &nbsp &nbsp
  1  Biosynthesis of secondary metabolites 0 &nbsp &nbsp &nbsp &nbsp &nbsp &nbsp
   yegK   ecocyc   kegg   phen   146  yfcQ  ecocyc   kegg  8.0 0.0  8  2-Acyl-sn-glycero-3-phosphoglycerol (n-C18:1)  2-Acyl-sn-glycero-3-phosphoglycerol (n-C18:1)  3.779
  2  Tyrosine metabolism 0.0002 &nbsp &nbsp &nbsp  3  O-acetyl-rhamanosyl-N-acetylglucosamyl-undecaprenyl diphosphate 0.000 0.000
   yegL   ecocyc   kegg   phen   162  ybjR  ecocyc   kegg  11.7 0.0  15  7-aminomethyl-7-deazaguanine  C01449  3.621
  7  Lysine degradation 9e-08  3  D-Alanine metabolism 0.005  14  L-Tryptophan 0.000 1.000
   yegP   ecocyc   kegg   phen   198  yebU  ecocyc   kegg  8.7 0.0  1  &nbsp &nbsp
  4  Riboflavin metabolism 4e-05  1  Sphingolipid metabolism 0.003  7  L-Threonine 0.000 0.000
   yegQ   ecocyc   kegg   phen   134  hycI  ecocyc   kegg  18.7 0.0  37  1,5-Diaminopentane  C01672  6.490
  10  Lysine degradation 1e-08  3  RNA polymerase 0.000  2  Succinate 0.001 0.000
   yegR   ecocyc   kegg   phen   361  ydcH  ecocyc   kegg  12.2 0.0  2  D-Malate  C00497  3.507
  1  Biosynthesis of secondary metabolites 0  8  Chlorocyclohexane and chlorobenzene degradation 0.000  21  3-Carboxy-2-hydroxy-4-methylpentanoate 0.000 0.000
   yegS   ecocyc   kegg   phen   85  alkA  ecocyc   kegg  8.6 0.0  2  (R)-Propane-1,2-diol  C02912  -3.472
  3  Glycerolipid metabolism 1e-07  18  Novobiocin biosynthesis 0.000  2  2-Succinyl-6-hydroxy-2,4-cyclohexadiene-1-carboxylate 0.000 0.000
   yegT   ecocyc   kegg   phen   189  ptsG  ecocyc   kegg  12.8 0.0  3  XDP  C01337  3.776
 &nbsp &nbsp &nbsp  16  ABC transporters 0.000  28  alpha-D-Galactose 1-phosphate 0.000 0.000
   yegU   ecocyc   kegg   phen   82  yccF  ecocyc   kegg  16.0 0.0  32  1,6-anhydrous-N-Acetylmuramate  1,6-anhydrous-N-Acetylmuramate  34.310
  9  Arginine and proline metabolism 2e-05  3  Bacterial secretion system 0.000  3  Propanoyl-CoA 0.000 0.000
   yegV   ecocyc   kegg   phen   82  yaeR  ecocyc   kegg  6.0 0.0  3  N-Acetyl-D-mannosamine  C00645  4.016
 &nbsp &nbsp &nbsp  3  Bacterial secretion system 0.000  1  L-Arginine 0.002 0.000
   yegW   ecocyc   kegg   phen   141  emrE  ecocyc   kegg  11.6 0.0  11  phosphatidylethanolamine (ditetradecanoyl, n-C14:0)  C00350  4.375
  19  Methane metabolism 3e-06  2  Aminoacyl-tRNA biosynthesis 0.002  15  (R)-Pantothenate 0.000 0.000
   yegX   ecocyc   kegg   phen   741  gnsB  ecocyc   kegg  16.4 0.0  25  N-Acetylneuraminate  C00270  4.526
  14  Purine metabolism 4e-08  5  Chlorocyclohexane and chlorobenzene degradation 0.000  20  D-Glycerate 2-phosphate 0.000 0.000
   yehA   ecocyc   kegg   phen   189  yeeW  ecocyc   kegg  12.9 0.0  31  fructoselysine  C16488  4.539
  14  C5-Branched dibasic acid metabolism 5e-05  9  Chlorocyclohexane and chlorobenzene degradation 0.000  8  Pyridoxine 5'-phosphate 0.000 0.000
   yehB   ecocyc   kegg   phen   125  creB  ecocyc   kegg  18.9 0.0  45  N1-Acetylspermidine  C00612  15.849
  16  Limonene and pinene degradation 1e-16  13  Pyruvate metabolism 0.000  7  2-Demethylmenaquinone 8 0.005 1.000
   yehC   ecocyc   kegg   phen   103  ykgN  ecocyc   kegg  14.7 0.0  2  Hydrogen sulfide  C00283  3.546
 &nbsp &nbsp &nbsp  16  Vitamin B6 metabolism 0.000  18  2,3-Dihydro-2,3-dihydroxybenzoate 0.000 0.000
   yehD   ecocyc   kegg   phen   134  yadM  ecocyc   kegg  8.5 0.0  8  N(omega)-(L-Arginino)succinate  C03406  4.228
  5  Galactose metabolism 7e-12  1  Riboflavin metabolism 0.007  10  O-acetyl-rhamanosyl-N-acetylglucosamyl-undecaprenyl diphosphate 0.000 0.000
   yehE   ecocyc   kegg   phen   116  atoS  ecocyc   kegg  12.9 0.0  3  Ornithine  C01602  3.502
  6  Phenylalanine metabolism 4e-07  2  RNA polymerase 0.000 &nbsp &nbsp &nbsp
   yehK   ecocyc   kegg   phen   110  yebZ  ecocyc   kegg  9.3 0.0  5  N1-(5-Phospho-D-ribosyl)glycinamide  C03838  3.721
  5  Fructose and mannose metabolism 3e-05  3  RNA polymerase 0.000  4  Undecaprenyl phosphate 0.000 0.000
   yehL   ecocyc   kegg   phen   324  yfbJ  ecocyc   kegg  10.9 0.0  2  Deoxyuridine  C00526  3.732
  7  Aminobenzoate degradation 1e-07  2  Glycerolipid metabolism 0.001  12  L-Threonine 0.000 0.000
   yehM   ecocyc   kegg   phen   100  clpA  ecocyc   kegg  8.8 0.0 &nbsp &nbsp &nbsp
  1  Biosynthesis of secondary metabolites 0  2  Glycolysis / Gluconeogenesis 0.008 &nbsp &nbsp &nbsp
   yehP   ecocyc   kegg   phen   74  ddpA  ecocyc   kegg  15.3 0.0  3  CDP-1,2-ditetradec-7-enoylglycerol  C00269  49.343
  3  Streptomycin biosynthesis 0.0002  1  Fructose and mannose metabolism 0.005  1  D-Fructose 1,6-bisphosphate 0.000 0.000
   yehQ   ecocyc   kegg   phen   67  clpX  ecocyc   kegg  11.9 0.0  27  4,5-dihydroxy-2,3-pentanedione  C11838  3.552
  22  D-Alanine metabolism 1e-08  2  Biosynthesis of unsaturated fatty acids 0.003  4  L-Aspartate 0.005 1.000
   yehR   ecocyc   kegg   phen   235  dacD  ecocyc   kegg  9.7 0.0 &nbsp &nbsp &nbsp
 &nbsp &nbsp &nbsp &nbsp &nbsp &nbsp &nbsp &nbsp &nbsp
   yehS   ecocyc   kegg   phen   88  yehW  ecocyc   kegg  11.8 0.0  9  4-Hydroxy-L-threonine  C06056  3.634
  2  Nicotinate and nicotinamide metabolism 0.002  5  Inositol phosphate metabolism 0.001  3  Glyceraldehyde 3-phosphate 0.006 0.000
   yehT   ecocyc   kegg   phen   295  yecN  ecocyc   kegg  9.1 0.0 &nbsp &nbsp &nbsp
 &nbsp &nbsp &nbsp &nbsp &nbsp &nbsp &nbsp &nbsp &nbsp
   yehU   ecocyc   kegg   phen   331  yebU  ecocyc   kegg  8.9 0.0  1  all-trans-Octaprenyl diphosphate  C04146  4.134
  3  Ubiquinone and other terpenoid-quinone biosynthesis 0  2  Bisphenol degradation 0.008  9  glucosyl-O-acetyl-rhamanosyl-N-acetylglucosamyl-undecaprenyl diphosphate 0.000 0.000
   yeiB   ecocyc   kegg   phen   89  hscA  ecocyc   kegg  13.5 0.0  49  Iminoaspartate  C05840  6.262
  15  C5-Branched dibasic acid metabolism 5e-13  7  Biosynthesis of siderophore group nonribosomal peptides 0.000  9  2,3-Dihydro-2,3-dihydroxybenzoate 0.000 0.000
   yeiE   ecocyc   kegg   phen   214  hepA  ecocyc   kegg  17.4 0.0  61  Cytidine  C00475  7.150
  7  Arachidonic acid metabolism 0  3  Two-component system 0.000  13  L-Proline 0.006 1.000
   yeiI   ecocyc   kegg   phen   111  yfbS  ecocyc   kegg  6.9 0.0  4  D-Lactaldehyde  C00937  3.602
 &nbsp &nbsp &nbsp  2  Other glycan degradation 0.002  3  (R)-Glycerate 0.001 0.000
   yeiP   ecocyc   kegg   phen   319  yeaP  ecocyc   kegg  9.7 0.0  1  Uridine  C00299  -4.172
 &nbsp &nbsp &nbsp  2  Glycerolipid metabolism 0.001  11  Phenylacetic acid 0.000 0.000
   yeiQ   ecocyc   kegg   phen   78  yjeK  ecocyc   kegg  9.5 0.0  14  2-Dehydro-3-deoxy-D-galactonate 6-phosphate  C01286  4.089
  6  Pentose phosphate pathway 6e-06  16  Phosphotransferase system (PTS) 0.000  9  Dephospho-CoA 0.000 0.000
   yeiR   ecocyc   kegg   phen   72  gmr  ecocyc   kegg  24.0 0.0  35  Oxalureate  C00802  8.123
  13  Lipopolysaccharide biosynthesis 3e-08  3  Folate biosynthesis 0.001  9  octanoate (n-C8:0) 0.000 0.000
   yeiS   ecocyc   kegg   phen   131  frmB  ecocyc   kegg  7.7 0.0 &nbsp &nbsp &nbsp
 &nbsp &nbsp &nbsp  2  Bacterial secretion system 0.005 &nbsp &nbsp &nbsp
   yeiW   ecocyc   kegg   phen   337  yodB  ecocyc   kegg  13.0 0.0  23  N2-Succinyl-L-glutamate 5-semialdehyde  C05932  4.353
  14  C5-Branched dibasic acid metabolism 3e-09 &nbsp &nbsp &nbsp  13  2-Octaprenyl-6-methoxyphenol 0.000 0.000
   yejG   ecocyc   kegg   phen   103  yfaS  ecocyc   kegg  9.7 0.0  12  Propanoyl phosphate  C02876  3.957
  8  Lysine degradation 1e-12  3  Arachidonic acid metabolism 0.001  1  Undecaprenyl diphosphate 0.000 0.000
   yejH   ecocyc   kegg   phen   142  lsrF  ecocyc   kegg  8.8 0.0  3  Phenylacetic acid  C07086  3.738
 &nbsp &nbsp &nbsp &nbsp &nbsp &nbsp  3  L-alanine-D-glutamate 0.000 0.000
   yejL   ecocyc   kegg   phen   74  yciG  ecocyc   kegg  10.9 0.0  3  2-(Formamido)-N1-(5-phospho-D-ribosyl)acetamidine  C04640  3.519
 &nbsp &nbsp &nbsp  7  Other glycan degradation 0.001 &nbsp &nbsp &nbsp
   yejO   ecocyc   kegg   phen   163  cld  ecocyc   kegg  9.6 0.0 &nbsp &nbsp &nbsp
  1  Biosynthesis of secondary metabolites 0  5  Chlorocyclohexane and chlorobenzene degradation 0.000 &nbsp &nbsp &nbsp
   yfaD   ecocyc   kegg   phen   58  iap  ecocyc   kegg  35.1 0.0  66  Dethiobiotin  C01909  32.491
  9  Bisphenol degradation 0.001  11  Aminoacyl-tRNA biosynthesis 0.000  5  Orotidine 5'-phosphate 0.000 0.000
   yfaE   ecocyc   kegg   phen   57  yccT  ecocyc   kegg  25.6 0.0  11  Dodecanoate (n-C12:0)  C02679  11.861
  7  Fatty acid biosynthesis 1e-06  4  Cysteine and methionine metabolism 0.001  1  L-Serine 0.000 0.000
   yfaH   ecocyc   kegg   phen   105  bcsE  ecocyc   kegg  12.2 0.0  3  o-Succinylbenzoate  C02730  3.694
  8  Lysine degradation 1e-08  2  Arachidonic acid metabolism 0.001  1  undecaprenyl phosphate-4-amino-4-deoxy-L-arabinose 0.000 0.000
   yfaP   ecocyc   kegg   phen   112  ypeC  ecocyc   kegg  12.0 0.0  14  gamma-butyrobetaine  C01181  4.055
  2  Thiamine metabolism 1e-05  6  Peptidoglycan biosynthesis 0.000  5  Cys-Gly 0.001 0.000
   yfaQ   ecocyc   kegg   phen   201  yfcP  ecocyc   kegg  20.5 0.0  32  4-Hydroxy-L-threonine  C06056  5.339
  10  Lysine degradation 1e-08  7  Chlorocyclohexane and chlorobenzene degradation 0.000  7  Pyridoxine 5'-phosphate 0.000 0.000
   yfaT   ecocyc   kegg   phen   74  yfcD  ecocyc   kegg  10.0 0.0  1  &nbsp &nbsp
  1  Biosynthesis of secondary metabolites 0 &nbsp &nbsp &nbsp  1  undecaprenyl phosphate-4-amino-4-deoxy-L-arabinose 0.000 0.000
   yfaY   ecocyc   kegg   phen   85  yfbK  ecocyc   kegg  9.7 0.0  1  octadecenoate (n-C18:1)  octadecenoate (n-C18:1)  3.853
  1  Biosynthesis of secondary metabolites 0  5  Phenylalanine metabolism 0.001  5  Undecaprenyl phosphate 0.000 0.000
   yfaZ   ecocyc   kegg   phen   273  dacD  ecocyc   kegg  10.1 0.0  1  crotonobetaine  C04114  -4.740
  2  Caprolactam degradation 0  2  Mismatch repair 0.003  13  L-Threonine 0.001 0.000
   yfbK   ecocyc   kegg   phen   93  yfaY  ecocyc   kegg  9.7 0.0  2  Choline  C00114  3.790
  5  Phenylalanine metabolism 2e-07  2  Pyrimidine metabolism 0.002  4  Uracil 0.000 0.000
   yfbL   ecocyc   kegg   phen   186  lsrF  ecocyc   kegg  11.0 0.0  1  L-Histidinol  C00860  3.754
 &nbsp &nbsp &nbsp  4  Glycerolipid metabolism 0.001  13  D-Mannose 0.000 0.000
   yfbM   ecocyc   kegg   phen   116  yfcP  ecocyc   kegg  21.4 0.0  28  Formate  C00058  8.952
  10  Purine metabolism 4e-09  7  Sulfur relay system 0.000  6  MoaD Protein with carboxylate 0.000 0.000
   yfbN   ecocyc   kegg   phen   89  artI  ecocyc   kegg  8.0 0.0  2  Thiosulfate  C00320  3.761
  4  Arginine and proline metabolism 0  16  Geraniol degradation 0.000  24  3-Oxodecanoyl-CoA 0.000 0.000
   yfbO   ecocyc   kegg   phen   133  yeeZ  ecocyc   kegg  11.5 0.0  7  Thiosulfate  C00320  4.262
  1  Biosynthesis of secondary metabolites 0.005  11  Other glycan degradation 0.000  5  Methylglyoxal 0.000 0.000
   yfbP   ecocyc   kegg   phen   94  hflK  ecocyc   kegg  10.3 0.0  14  8-Amino-7-oxononanoate  C01092  4.035
  6  Tyrosine metabolism 8e-06 &nbsp &nbsp &nbsp &nbsp &nbsp &nbsp
   yfbS   ecocyc   kegg   phen   68  ykfG  ecocyc   kegg  9.7 0.0 &nbsp &nbsp &nbsp
 &nbsp &nbsp &nbsp  2  Other glycan degradation 0.001 &nbsp &nbsp &nbsp
   yfbT   ecocyc   kegg   phen   531  ynjC  ecocyc   kegg  10.2 0.0  6  Lactose  C00243  -4.347
  8  Galactose metabolism 7e-11  1  Mismatch repair 0.000  20  glucosyl-O-acetyl-rhamanosyl-N-acetylglucosamyl-undecaprenyl diphosphate 0.000 0.000
   yfbU   ecocyc   kegg   phen   167  ydeO  ecocyc   kegg  7.6 0.0  3  2-Succinyl-6-hydroxy-2,4-cyclohexadiene-1-carboxylate  C05817  3.691
  3  Ubiquinone and other terpenoid-quinone biosynthesis 0  1  Riboflavin metabolism 0.000  10  O-acetyl-rhamanosyl-N-acetylglucosamyl-undecaprenyl diphosphate 0.000 0.000
   yfbV   ecocyc   kegg   phen   119  lsrF  ecocyc   kegg  9.1 0.0  4  6-Phospho-D-gluconate  C00345  3.464
 &nbsp &nbsp &nbsp  2  Terpenoid backbone biosynthesis 0.003  8  D-Glycerate 2-phosphate 0.000 0.000
   yfcA   ecocyc   kegg   phen   94  adhP  ecocyc   kegg  12.8 0.0  57  D-Glucosamine 1-phosphate  C06156  22.252
  15  C5-Branched dibasic acid metabolism 5e-07  3  Chloroalkane and chloroalkene degradation 0.002  2  NMN 0.000 0.000
   yfcC   ecocyc   kegg   phen   155  ybdB  ecocyc   kegg  9.6 0.0  2  glycogen  C00182  3.493
 &nbsp &nbsp &nbsp  1  Glycerolipid metabolism 0.009  5  Choline 0.001 0.000
   yfcD   ecocyc   kegg   phen   205  yfcP  ecocyc   kegg  12.9 0.0  13  3'-Phosphoadenylyl sulfate  C00053  -3.953
 &nbsp &nbsp &nbsp  7  Chlorocyclohexane and chlorobenzene degradation 0.000  12  Cadmium 0.000 0.000
   yfcH   ecocyc   kegg   phen   81  ycaO  ecocyc   kegg  7.6 0.0 &nbsp &nbsp &nbsp
 &nbsp &nbsp &nbsp  3  Other glycan degradation 0.001 &nbsp &nbsp &nbsp
   yfcI   ecocyc   kegg   phen   106  pepE  ecocyc   kegg  10.8 0.0  18  Methylglyoxal  C00546  -3.533
  36  D-Alanine metabolism 7e-10  3  Streptomycin biosynthesis 0.001  6  Formate 0.000 1.000
   yfcJ   ecocyc   kegg   phen   188  ydjX  ecocyc   kegg  14.6 0.0  24  N-Acetyl-L-glutamate  C00624  5.258
  10  D-Glutamine and D-glutamate metabolism 2e-05  7  Butanoate metabolism 0.001  14  D-Glycerate 2-phosphate 0.000 0.000
   yfcL   ecocyc   kegg   phen   85  dgoK  ecocyc   kegg  7.9 0.0  10  8-Amino-7-oxononanoate  C01092  4.357
  14  Chlorocyclohexane and chlorobenzene degradation 4e-08  7  Oxidative phosphorylation 0.000  8  Succinate 0.001 1.000
   yfcM   ecocyc   kegg   phen   358  yfbT  ecocyc   kegg  9.5 0.0  4  (R)-Propane-1,2-diol  C02912  3.496
  6  Glycerolipid metabolism 2e-06  5  Glycerolipid metabolism 0.002  18  Phenylacetic acid 0.000 0.000
   yfcN   ecocyc   kegg   phen   71  ycaK  ecocyc   kegg  8.3 0.0  2  Cytosine  C00380  4.371
  1  Vitamin B6 metabolism 5e-05  3  D-Glutamine and D-glutamate metabolism 0.000  2  chorismate 0.000 0.000
   yfcO   ecocyc   kegg   phen   89  yjeO  ecocyc   kegg  8.8 0.0  4  1-dodecanoyl-sn-glycerol 3-phosphate  C00681  4.001
  6  Butanoate metabolism 2e-05  6  Chlorocyclohexane and chlorobenzene degradation 0.000  3  ferroxamine 0.000 0.000
   yfcP   ecocyc   kegg   phen   248  yfbM  ecocyc   kegg  21.4 0.0  67  5-Phospho-beta-D-ribosylamine  C03090  4.891
  15  Pyrimidine metabolism 3e-12  4  Peptidoglycan biosynthesis 0.000  9  Thiamin monophosphate 0.000 0.000
   yfcQ   ecocyc   kegg   phen   297  ydeH  ecocyc   kegg  8.2 0.0  9  D-Glucosamine 1-phosphate  C06156  3.520
  1  Biosynthesis of secondary metabolites 7e-10  1  Peptidoglycan biosynthesis 0.008  13  2-Oxoglutarate 0.007 1.000
   yfcR   ecocyc   kegg   phen   97  yfcP  ecocyc   kegg  10.5 0.0  5  d-biotin d-sulfoxide  d-biotin d-sulfoxide  3.535
 &nbsp &nbsp &nbsp  8  Arachidonic acid metabolism 0.001  2  undecaprenyl phosphate-4-amino-4-deoxy-L-arabinose 0.000 0.000
   yfcS   ecocyc   kegg   phen   254  wbbI  ecocyc   kegg  7.6 0.0 &nbsp &nbsp &nbsp
 &nbsp &nbsp &nbsp &nbsp &nbsp &nbsp &nbsp &nbsp &nbsp
   yfcT   ecocyc   kegg   phen   134  yeiE  ecocyc   kegg  14.6 0.0  23  Lactose  C00243  5.583
  5  alpha-Linolenic acid metabolism 3e-08  2  Two-component system 0.000  6  Putrescine 0.004 1.000
   yfcU   ecocyc   kegg   phen   182  wbbK  ecocyc   kegg  7.4 0.0  9  Hexanoate (n-C6:0)  C01585  3.740
  8  Galactose metabolism 5e-09 &nbsp &nbsp &nbsp  7  glucosyl-O-acetyl-rhamanosyl-N-acetylglucosamyl-undecaprenyl diphosphate 0.000 0.000
   yfcZ   ecocyc   kegg   phen   127  yeeS  ecocyc   kegg  13.8 0.0  26  4-Phospho-L-aspartate  C03082  5.330
  12  Pentose and glucuronate interconversions 3e-08  6  Biotin metabolism 0.000  14  MoaD Protein with carboxylate 0.000 0.000
   yfdC   ecocyc   kegg   phen   240  ybiR  ecocyc   kegg  9.9 0.0  5  Superoxide anion  C00704  4.545
  9  Biosynthesis of secondary metabolites 7e-09 &nbsp &nbsp &nbsp  16  L-Asparagine 0.000 1.000
   yfdE   ecocyc   kegg   phen   146  glf  ecocyc   kegg  11.1 0.0 &nbsp &nbsp &nbsp
 &nbsp &nbsp &nbsp  1  Riboflavin metabolism 0.000 &nbsp &nbsp &nbsp
   yfdF   ecocyc   kegg   phen   92  ylbF  ecocyc   kegg  29.5 0.0  157  Lipoate  C00725  8.145
  10  D-Glutamine and D-glutamate metabolism 2e-08  7  Two-component system 0.000  4  Spermidine 0.001 0.000
   yfdK   ecocyc   kegg   phen   175  hslV  ecocyc   kegg  20.1 0.0  22  Deoxyadenosine  C00559  4.706
  4  Arachidonic acid metabolism 0  3  Taurine and hypotaurine metabolism 0.002  3  Undecaprenyl phosphate 0.000 0.000
   yfdL   ecocyc   kegg   phen   283  ydfO  ecocyc   kegg  9.3 0.0  1  Tetradecanoyl-phosphate (n-C14:0)  Tetradecanoyl-phosphate (n-C14:0)  3.613
 &nbsp &nbsp &nbsp  3  Base excision repair 0.002  11  L-Threonine 0.000 0.000
   yfdM   ecocyc   kegg   phen   129  yfeD  ecocyc   kegg  14.7 0.0  10  fructoselysine Phosphate  C16489  3.779
  9  Lysine degradation 1e-12  7  RNA polymerase 0.000  3  2-Octaprenyl-3-methyl-6-methoxy- 1,4-benzoquinol 0.000 0.000
   yfdN   ecocyc   kegg   phen   219  ylbH  ecocyc   kegg  7.8 0.0 &nbsp &nbsp &nbsp
 &nbsp &nbsp &nbsp  4  Chlorocyclohexane and chlorobenzene degradation 0.000 &nbsp &nbsp &nbsp
   yfdO   ecocyc   kegg   phen   158  rpoZ  ecocyc   kegg  14.4 0.0  69  1,5-Diaminopentane  C01672  6.046
  12  Lysine degradation 0  7  RNA polymerase 0.000  6  Cys-Gly 0.000 0.000
   yfdP   ecocyc   kegg   phen   179  hinT  ecocyc   kegg  7.5 0.0  1  2-Acyl-sn-glycero-3-phosphoglycerol (n-C18:0)  2-Acyl-sn-glycero-3-phosphoglycerol (n-C18:0)  -4.867
  1  Biosynthesis of secondary metabolites 0  1  Fructose and mannose metabolism 0.005  12  glucosyl-O-acetyl-rhamanosyl-N-acetylglucosamyl-undecaprenyl diphosphate 0.000 0.000
   yfdQ   ecocyc   kegg   phen   104  yfaP  ecocyc   kegg  11.2 0.0  8  Formate  C00058  4.950
 &nbsp &nbsp &nbsp  7  Arachidonic acid metabolism 0.001  2  2-Demethylmenaquinone 8 0.003 0.000
   yfdR   ecocyc   kegg   phen   105  mepA  ecocyc   kegg  9.5 0.0  6  2-Oxoglutarate  C00026  4.219
  2  Porphyrin and chlorophyll metabolism 2e-05  12  Two-component system 0.000  7  L-Malate 0.000 0.000
   yfdS   ecocyc   kegg   phen   169  ybgI  ecocyc   kegg  7.5 0.0  3  UTP  C00075  -3.487
 &nbsp &nbsp &nbsp &nbsp &nbsp &nbsp  5  glucosyl-O-acetyl-rhamanosyl-N-acetylglucosamyl-undecaprenyl diphosphate 0.000 0.000
   yfdV   ecocyc   kegg   phen   236  rcsB  ecocyc   kegg  20.9 0.0  141  Isopentenyl diphosphate  C00129  8.240
  19  Nicotinate and nicotinamide metabolism 2e-07  12  Two-component system 0.000  19  L-alanine-D-glutamate 0.001 1.000
   yfdX   ecocyc   kegg   phen   114  eutQ  ecocyc   kegg  20.0 0.0  36  2-Hydroxy-6-oxonona-2,4-diene-1,9-dioate  C04479  6.612
  7  Lipoic acid metabolism 0  6  RNA polymerase 0.000  8  Succinate 0.005 1.000
   yfdY   ecocyc   kegg   phen   141  barA  ecocyc   kegg  9.2 0.0  16  2-hydroxy-6-ketononatrienedioate  C12624  5.082
  11  Glycolysis / Gluconeogenesis 5e-06  2  Chlorocyclohexane and chlorobenzene degradation 0.000  2  D-Gluconate 0.000 0.000
   yfeA   ecocyc   kegg   phen   289  ydfO  ecocyc   kegg  9.1 0.0  5  7-cyano-7-carbaguanine  C15996  3.914
  3  Alanine, aspartate and glutamate metabolism 0.0002 &nbsp &nbsp &nbsp  5  Dihydroxyacetone 0.001 0.000
   yfeC   ecocyc   kegg   phen   111  hslU  ecocyc   kegg  15.0 0.0  12  N1-Acetylspermidine  C00612  3.603
  34  Microbial metabolism in diverse environments 0  4  Peptidoglycan biosynthesis 0.000  5  Cys-Gly 0.000 0.000
   yfeD   ecocyc   kegg   phen   143  yfdM  ecocyc   kegg  14.7 0.0  26  8-Amino-7-oxononanoate  C01092  3.934
  11  Fatty acid biosynthesis 1e-07  8  RNA polymerase 0.000  4  Succinate 0.000 1.000
   yfeH   ecocyc   kegg   phen   322  ygcW  ecocyc   kegg  11.9 0.0  6  S-Ribosyl-L-homocysteine  C03539  4.145
  6  Purine metabolism 5e-08  5  Chlorocyclohexane and chlorobenzene degradation 0.000  22  bis-molybdopterin guanine dinucleotide 0.000 0.000
   yfeK   ecocyc   kegg   phen   107  yfbR  ecocyc   kegg  9.9 0.0  13  N1-Acetylspermidine  C00612  3.715
  9  D-Alanine metabolism 4e-05  14  Glycolysis / Gluconeogenesis 0.000  10  Cys-Gly 0.000 0.000
   yfeN   ecocyc   kegg   phen   256  yggS  ecocyc   kegg  9.8 0.0  3  7-cyano-7-carbaguanine  C15996  3.791
  7  Alanine, aspartate and glutamate metabolism 0.0003 &nbsp &nbsp &nbsp  14  O-acetyl-rhamanosyl-N-acetylglucosamyl-undecaprenyl diphosphate 0.000 0.000
   yfeO   ecocyc   kegg   phen   81  osmE  ecocyc   kegg  8.6 0.0  1  Butanal  C01412  3.466
 &nbsp &nbsp &nbsp  2  Flagellar assembly 0.006  1  L-Homoserine 0.000 0.000
   yfeR   ecocyc   kegg   phen   272  nadR  ecocyc   kegg  9.6 0.0 &nbsp &nbsp &nbsp
 &nbsp &nbsp &nbsp &nbsp &nbsp &nbsp &nbsp &nbsp &nbsp
   yfeS   ecocyc   kegg   phen   328  rpsO  ecocyc   kegg  11.7 0.0 &nbsp &nbsp &nbsp
 &nbsp &nbsp &nbsp  3  Chlorocyclohexane and chlorobenzene degradation 0.000 &nbsp &nbsp &nbsp
   yfeW   ecocyc   kegg   phen   332  pfkB  ecocyc   kegg  9.2 0.0  2  1,5-Diaminopentane  C01672  3.567
  5  C5-Branched dibasic acid metabolism 8e-12  5  Mismatch repair 0.001  16  glucosyl-O-acetyl-rhamanosyl-N-acetylglucosamyl-undecaprenyl diphosphate 0.000 0.000
   yfeX   ecocyc   kegg   phen   281  yadL  ecocyc   kegg  16.1 0.0  13  cyclopropane phosphatidylethanolamine (dihexadec-9,10-cyclo-anoyl, n-C16:0 cyclo)  cyclopropane phosphatidylethanolamine (dihexadec-9,10-cyclo-anoyl, n-C16:0 cyclo)  4.418
  4  Arachidonic acid metabolism 0  4  Toluene degradation 0.003  15  3-Oxodecanoyl-CoA 0.001 0.000
   yfeY   ecocyc   kegg   phen   83  yfdM  ecocyc   kegg  10.1 0.0  9  Pyridoxamine  C00534  4.974
  2  Arachidonic acid metabolism 0.0007  9  Arachidonic acid metabolism 0.000 &nbsp &nbsp &nbsp
   yfeZ   ecocyc   kegg   phen   94  ybgL  ecocyc   kegg  8.7 0.0  6  Formate  C00058  5.352
  6  beta-Alanine metabolism 0.0006  3  Sulfur relay system 0.000  8  MoaD Protein with carboxylate 0.000 0.000
   yffB   ecocyc   kegg   phen   52  frmR  ecocyc   kegg  12.1 0.0  6  Deoxycytidine  C00881  5.680
  1  Pantothenate and CoA biosynthesis 0  5  Valine, leucine and isoleucine degradation 0.000 &nbsp &nbsp &nbsp
   yfgC   ecocyc   kegg   phen   93  macA  ecocyc   kegg  7.9 0.0 &nbsp &nbsp &nbsp
 &nbsp &nbsp &nbsp  5  Chlorocyclohexane and chlorobenzene degradation 0.000 &nbsp &nbsp &nbsp
   yfgD   ecocyc   kegg   phen   233  ogrK  ecocyc   kegg  13.4 0.0  5  methylisocitrate  C04593  3.572
  2  Chloroalkane and chloroalkene degradation 0  3  Bisphenol degradation 0.004  29  S-Formylglutathione 0.000 0.000
   yfgF   ecocyc   kegg   phen   114  mdtD  ecocyc   kegg  8.8 0.0  2  4-Phospho-L-aspartate  C03082  3.782
 &nbsp &nbsp &nbsp  4  Histidine metabolism 0.002  2  L-Arginine 0.004 0.000
   yfgG   ecocyc   kegg   phen   136  exo  ecocyc   kegg  7.6 0.0  2  Propanal  C00479  3.799
 &nbsp &nbsp &nbsp  4  Ribosome 0.001  8  Urate 0.000 0.000
   yfgH   ecocyc   kegg   phen   203  ydfO  ecocyc   kegg  10.3 0.0  3  Uridine  C00299  -3.956
  3  Biotin metabolism 7e-06  1  Sphingolipid metabolism 0.003  6  glucosyl-O-acetyl-rhamanosyl-N-acetylglucosamyl-undecaprenyl diphosphate 0.000 0.000
   yfgI   ecocyc   kegg   phen   184  yjhS  ecocyc   kegg  7.5 0.0 &nbsp &nbsp &nbsp
 &nbsp &nbsp &nbsp  2  Chloroalkane and chloroalkene degradation 0.007 &nbsp &nbsp &nbsp
   yfgJ   ecocyc   kegg   phen   289  nudD  ecocyc   kegg  9.9 0.0  7  D-tartrate  C02107  3.719
  4  Glyoxylate and dicarboxylate metabolism 2e-06  3  Mismatch repair 0.001  16  glucosyl-O-acetyl-rhamanosyl-N-acetylglucosamyl-undecaprenyl diphosphate 0.000 0.000
   yfgM   ecocyc   kegg   phen   77  ygcB  ecocyc   kegg  24.1 0.0  100  Glycine betaine  C00719  27.065
  19  D-Alanine metabolism 8e-07  13  Butanoate metabolism 0.000  2  Fumarate 0.005 1.000
   yfgO   ecocyc   kegg   phen   155  melB  ecocyc   kegg  8.4 0.0 &nbsp &nbsp &nbsp
 &nbsp &nbsp &nbsp  4  Other glycan degradation 0.000 &nbsp &nbsp &nbsp
   yfhG   ecocyc   kegg   phen   167  ymcB  ecocyc   kegg  11.6 0.0  51  (R)-S-Lactoylglutathione  C03451  6.873
  9  Arachidonic acid metabolism 0  2  Two-component system 0.000  6  5-Phospho-beta-D-ribosylamine 0.000 0.000
   yfhH   ecocyc   kegg   phen   96  ydiF  ecocyc   kegg  7.4 0.0 &nbsp &nbsp &nbsp
 &nbsp &nbsp &nbsp &nbsp &nbsp &nbsp &nbsp &nbsp &nbsp
   yfhL   ecocyc   kegg   phen   151  ypjL  ecocyc   kegg  9.3 0.0 &nbsp &nbsp &nbsp
 &nbsp &nbsp &nbsp  8  Chlorocyclohexane and chlorobenzene degradation 0.000 &nbsp &nbsp &nbsp
   yfhM   ecocyc   kegg   phen   125  ydfZ  ecocyc   kegg  8.4 0.0  101  2-Hydroxy-3-oxopropanoate  C01146  5.595
  14  Arachidonic acid metabolism 0  4  Chlorocyclohexane and chlorobenzene degradation 0.000  3  S-Adenosyl-L-homocysteine 0.001 0.000
   yfhR   ecocyc   kegg   phen   106  hyfE  ecocyc   kegg  6.5 0.0 &nbsp &nbsp &nbsp
 &nbsp &nbsp &nbsp  5  Ribosome 0.000 &nbsp &nbsp &nbsp
   yfiB   ecocyc   kegg   phen   103  ygcO  ecocyc   kegg  8.0 0.0  2  2-Oxopent-4-enoate  C00596  3.798
  2  Methane metabolism 0  9  Bisphenol degradation 0.001  1  L-Homocysteine 0.000 0.000
   yfiC   ecocyc   kegg   phen   93  yfiQ  ecocyc   kegg  7.4 0.0 &nbsp &nbsp &nbsp
 &nbsp &nbsp &nbsp  3  Ethylbenzene degradation 0.002 &nbsp &nbsp &nbsp
   yfiE   ecocyc   kegg   phen   138  yffI  ecocyc   kegg  12.7 0.0  2  dGMP  C00362  -3.716
  1  Biosynthesis of secondary metabolites 0  6  Chlorocyclohexane and chlorobenzene degradation 0.000  7  2-Octaprenyl-6-methoxyphenol 0.000 0.000
   yfiF   ecocyc   kegg   phen   82  ybaO  ecocyc   kegg  5.5 0.0 &nbsp &nbsp &nbsp
 &nbsp &nbsp &nbsp  7  Novobiocin biosynthesis 0.000 &nbsp &nbsp &nbsp
   yfiH   ecocyc   kegg   phen   118  fimE  ecocyc   kegg  9.8 0.0 &nbsp &nbsp &nbsp
 &nbsp &nbsp &nbsp  3  Pyrimidine metabolism 0.001 &nbsp &nbsp &nbsp
   yfiL   ecocyc   kegg   phen   268  yfbE  ecocyc   kegg  8.6 0.0  6  Uridine  C00299  4.466
  6  Glycerolipid metabolism 2e-06  4  Mismatch repair 0.003  13  glucosyl-O-acetyl-rhamanosyl-N-acetylglucosamyl-undecaprenyl diphosphate 0.000 0.000
   yfiM   ecocyc   kegg   phen   173  hcaT  ecocyc   kegg  14.6 0.0  17  4-Phospho-L-aspartate  C03082  4.327
  8  Phenylalanine metabolism 3e-06  6  Pantothenate and CoA biosynthesis 0.000  4  2-Oxobutanoate 0.000 0.000
   yfiP   ecocyc   kegg   phen   326  yegX  ecocyc   kegg  10.4 0.0  8  D-Glucosamine 1-phosphate  C06156  5.209
  4  Riboflavin metabolism 4e-05  1  Mismatch repair 0.007  18  glucosyl-O-acetyl-rhamanosyl-N-acetylglucosamyl-undecaprenyl diphosphate 0.000 0.000
   yfiQ   ecocyc   kegg   phen   142  yaaI  ecocyc   kegg  16.6 0.0 &nbsp &nbsp &nbsp
  7  Vitamin B6 metabolism 0.0001  3  Pyrimidine metabolism 0.000 &nbsp &nbsp &nbsp
   yfiR   ecocyc   kegg   phen   124  hcaT  ecocyc   kegg  10.0 0.0  17  L-Lyxose  C01508  3.698
  9  Pentose and glucuronate interconversions 2e-16  4  Chlorocyclohexane and chlorobenzene degradation 0.000  1  alpha,alpha'-Trehalose 6-phosphate 0.000 0.000
   yfjD   ecocyc   kegg   phen   294  ydfO  ecocyc   kegg  9.9 0.0  9  D-Glucosamine 1-phosphate  C06156  3.640
  14  Aminobenzoate degradation 3e-06  4  Mismatch repair 0.004  13  glucosyl-O-acetyl-rhamanosyl-N-acetylglucosamyl-undecaprenyl diphosphate 0.000 0.000
   yfjF   ecocyc   kegg   phen   136  minC  ecocyc   kegg  13.2 0.0  14  trans-Aconitate  C02341  3.491
  9  C5-Branched dibasic acid metabolism 1e-07  13  Phenylalanine, tyrosine and tryptophan biosynthesis 0.000  16  2,3-Dihydro-2,3-dihydroxybenzoate 0.000 0.000
   yfjH   ecocyc   kegg   phen   101  yebZ  ecocyc   kegg  8.4 0.0  26  3-Deoxy-D-manno-2-octulosonate  C01187  4.228
  11  Nicotinate and nicotinamide metabolism 2e-07  2  Two-component system 0.002  1  UDP 0.000 0.000
   yfjI   ecocyc   kegg   phen   90  hcaT  ecocyc   kegg  6.3 0.0  24  N-(5-Phospho-D-ribosyl)anthranilate  C04302  4.377
  4  Ascorbate and aldarate metabolism 9e-06  5  Chlorocyclohexane and chlorobenzene degradation 0.000  7  D-Tagatose 1,6-biphosphate 0.000 0.000
   yfjJ   ecocyc   kegg   phen   213  yfcX  ecocyc   kegg  10.4 0.0 &nbsp &nbsp &nbsp
 &nbsp &nbsp &nbsp  5  Caprolactam degradation 0.001 &nbsp &nbsp &nbsp
   yfjL   ecocyc   kegg   phen   133  ygeL  ecocyc   kegg  10.1 0.0  5  L-ascorbate-6-phosphate  C16186  4.023
 &nbsp &nbsp &nbsp  1  Histidine metabolism 0.003  5  silver 0.000 0.000
   yfjM   ecocyc   kegg   phen   85  yfjG  ecocyc   kegg  6.2 0.0  2  2-hydroxy-6-ketononatrienedioate  C12624  3.709
  3  Cyanoamino acid metabolism 0.0002  4  Selenoamino acid metabolism 0.000  4  5-Methyltetrahydrofolate 0.000 0.000
   yfjP   ecocyc   kegg   phen   123  narY  ecocyc   kegg  14.8 0.0  4  O-Acetyl-L-serine  C00979  5.190
  19  D-Glutamine and D-glutamate metabolism 9e-07  5  Bacterial secretion system 0.004 &nbsp &nbsp &nbsp
   yfjQ   ecocyc   kegg   phen   154  cusA  ecocyc   kegg  17.6 0.0  66  cyclopropane phosphatidylethanolamine (dihexadec-9,10-cyclo-anoyl, n-C16:0 cyclo)  cyclopropane phosphatidylethanolamine (dihexadec-9,10-cyclo-anoyl, n-C16:0 cyclo)  3.977
  17  Lipoic acid metabolism 0  10  Two-component system 0.000  9  D-Glycerate 2-phosphate 0.000 0.000
   yfjR   ecocyc   kegg   phen   123  uhpT  ecocyc   kegg  11.0 0.0  10  N-Acetyl-D-glucosamine  C00140  9.980
  7  Biosynthesis of unsaturated fatty acids 5e-06  9  Chlorocyclohexane and chlorobenzene degradation 0.000  8  Pyridoxine 5'-phosphate 0.000 0.000
   yfjS   ecocyc   kegg   phen   198  yfcQ  ecocyc   kegg  7.4 0.0 &nbsp &nbsp &nbsp
 &nbsp &nbsp &nbsp &nbsp &nbsp &nbsp &nbsp &nbsp &nbsp
   yfjT   ecocyc   kegg   phen   126  sbmC  ecocyc   kegg  12.8 0.0  6  2,3-Dihydro-2,3-dihydroxybenzoate  C04171  3.477
  1  Inositol phosphate metabolism 0  6  Fatty acid biosynthesis 0.001  9  o-Succinylbenzoate 0.000 0.000
   yfjU   ecocyc   kegg   phen   83  rcsD  ecocyc   kegg  6.1 0.0 &nbsp &nbsp &nbsp
 &nbsp &nbsp &nbsp  5  alpha-Linolenic acid metabolism 0.001 &nbsp &nbsp &nbsp
   yfjV   ecocyc   kegg   phen   143  ypdI  ecocyc   kegg  22.8 0.0  86  Lactose  C00243  9.335
  15  Arachidonic acid metabolism 0  5  Two-component system 0.000  1  D-Lactate 0.000 0.000
   yfjX   ecocyc   kegg   phen   97  ybjQ  ecocyc   kegg  11.5 0.0  20  trans-Aconitate  C02341  5.281
  7  C5-Branched dibasic acid metabolism 0  3  Biosynthesis of siderophore group nonribosomal peptides 0.000  9  2,3-Dihydro-2,3-dihydroxybenzoate 0.000 0.000
   yfjY   ecocyc   kegg   phen   82  ygcB  ecocyc   kegg  23.9 0.0  26  Glycine betaine  C00719  8.823
  6  Sulfur metabolism 0.002  7  Pyrimidine metabolism 0.001 &nbsp &nbsp &nbsp
   ygaC   ecocyc   kegg   phen   137  uhpB  ecocyc   kegg  12.4 0.0  27  N2-Succinyl-L-ornithine  C03415  5.324
  14  Lysine degradation 3e-09  8  RNA polymerase 0.000  2  Putrescine 0.002 0.000
   ygaD   ecocyc   kegg   phen   100  yccT  ecocyc   kegg  21.9 0.0  31  5,6-dihydrouracil  C00429  15.433
  15  Fatty acid biosynthesis 4e-07  6  Chlorocyclohexane and chlorobenzene degradation 0.000  15  Reduced riboflavin 0.000 0.000
   ygaM   ecocyc   kegg   phen   142  yfjW  ecocyc   kegg  17.2 0.0  59  5,10-Methenyltetrahydrofolate  C00445  7.595
  10  Phosphotransferase system (PTS) 8e-05  7  Oxidative phosphorylation 0.000  7  D-Glycerate 2-phosphate 0.000 0.000
   ygaP   ecocyc   kegg   phen   73  yoaE  ecocyc   kegg  10.4 0.0  1  &nbsp &nbsp
 &nbsp &nbsp &nbsp  11  Two-component system 0.001  2  2-Demethylmenaquinol 8 0.001 0.000
   ygaQ   ecocyc   kegg   phen   215  cspB  ecocyc   kegg  11.1 0.0  7  gamma-glutamyl-gamma-butyraldehyde  C15700  3.765
  17  Nitrogen metabolism 7e-05  2  Limonene and pinene degradation 0.002  13  D-Glycerate 2-phosphate 0.000 0.000
   ygaR   ecocyc   kegg   phen   228  eutT  ecocyc   kegg  14.9 0.0  37  Agmatine  C00179  5.440
  8  Lysine degradation 2e-07  11  Two-component system 0.000  14  6-phospho-D-glucono-1,5-lactone 0.000 0.000
   ygaU   ecocyc   kegg   phen   83  ygeX  ecocyc   kegg  6.6 0.0  1  7-cyano-7-carbaguanine  C15996  3.566
  3  Alanine, aspartate and glutamate metabolism 0.0002  15  Phosphotransferase system (PTS) 0.000  6  L-Cystathionine 0.000 0.000
   ygaV   ecocyc   kegg   phen   58  ygaF  ecocyc   kegg  7.6 0.0  13  cyclopropane phosphatidylethanolamine (dihexadec-9,10-cyclo-anoyl, n-C16:0 cyclo)  cyclopropane phosphatidylethanolamine (dihexadec-9,10-cyclo-anoyl, n-C16:0 cyclo)  3.522
  18  Propanoate metabolism 1e-08  5  RNA polymerase 0.000 &nbsp &nbsp &nbsp
   ygaW   ecocyc   kegg   phen   184  yhiI  ecocyc   kegg  11.7 0.0  37  1,2-Diacyl-sn-glycerol (dioctadecanoyl, n-C18:0)  C00641  5.401
  9  Arachidonic acid metabolism 0  6  RNA polymerase 0.000  4  Succinate 0.001 0.000
   ygaX   ecocyc   kegg   phen   119  ycbZ  ecocyc   kegg  8.6 0.0 &nbsp &nbsp &nbsp
 &nbsp &nbsp &nbsp  11  Fatty acid metabolism 0.001 &nbsp &nbsp &nbsp
   ygbA   ecocyc   kegg   phen   101  ydhK  ecocyc   kegg  11.2 0.0  1  cyclopropane phosphatidylethanolamine (dihexadec-9,10-cyclo-anoyl, n-C16:0 cyclo)  cyclopropane phosphatidylethanolamine (dihexadec-9,10-cyclo-anoyl, n-C16:0 cyclo)  -3.673
  2  beta-Alanine metabolism 0  1  Biosynthesis of unsaturated fatty acids 0.007  7  octadecanoate (n-C18:0) 0.001 0.000
   ygbE   ecocyc   kegg   phen   132  yoaF  ecocyc   kegg  9.1 0.0  4  O-Phospho-L-homoserine  C01102  3.671
  2  Glutathione metabolism 3e-05  2  Lysine biosynthesis 0.004  2  Undecaprenyl phosphate 0.000 0.000
   ygbF   ecocyc   kegg   phen   215  thrL  ecocyc   kegg  13.2 0.0  4  Glycolaldehyde  C00266  3.944
  8  Aminobenzoate degradation 5e-08  1  Mismatch repair 0.008  11  glucosyl-O-acetyl-rhamanosyl-N-acetylglucosamyl-undecaprenyl diphosphate 0.000 0.000
   ygbI   ecocyc   kegg   phen   76  ypdE  ecocyc   kegg  8.7 0.0  6  N-Acetyl-D-glucosamine  C00140  4.344
  4  Amino sugar and nucleotide sugar metabolism 1e-06  8  D-Glutamine and D-glutamate metabolism 0.000  3  Melibiose 0.000 0.000
   ygbJ   ecocyc   kegg   phen   111  tap  ecocyc   kegg  12.9 0.0  9  N1-Acetylspermidine  C00612  3.534
  2  Pantothenate and CoA biosynthesis 4e-05  13  Aminoacyl-tRNA biosynthesis 0.001  26  Formyl-CoA 0.000 0.000
   ygbK   ecocyc   kegg   phen   69  yfcN  ecocyc   kegg  8.1 0.0  7  (S)-Dihydroorotate  C00337  3.511
  7  Alanine, aspartate and glutamate metabolism 0.0003  2  Valine, leucine and isoleucine biosynthesis 0.004 &nbsp &nbsp &nbsp
   ygbL   ecocyc   kegg   phen   109  yohL  ecocyc   kegg  15.7 0.0  3  Hexadecenoate (n-C16:1)  C08362  3.586
 &nbsp &nbsp &nbsp  7  Bacterial secretion system 0.000  8  o-Succinylbenzoate 0.000 0.000
   ygbM   ecocyc   kegg   phen   111  ybdB  ecocyc   kegg  7.4 0.0 &nbsp &nbsp &nbsp
 &nbsp &nbsp &nbsp  5  Nitrotoluene degradation 0.001 &nbsp &nbsp &nbsp
   ygbN   ecocyc   kegg   phen   150  srlB  ecocyc   kegg  8.1 0.0 &nbsp &nbsp &nbsp
 &nbsp &nbsp &nbsp  10  Valine, leucine and isoleucine biosynthesis 0.001 &nbsp &nbsp &nbsp
   ygcB   ecocyc   kegg   phen   39  yfgM  ecocyc   kegg  24.1 0.0  26  4-Hydroxy-L-threonine  C06056  5.158
  6  Sulfur metabolism 0.002  14  D-Glutamine and D-glutamate metabolism 0.000 &nbsp &nbsp &nbsp
   ygcE   ecocyc   kegg   phen   175  ygdB  ecocyc   kegg  8.2 0.0  5  Glycine betaine  C00719  3.723
 &nbsp &nbsp &nbsp &nbsp &nbsp &nbsp  2  D-Ribulose 5-phosphate 0.000 0.000
   ygcF   ecocyc   kegg   phen   61  glxR  ecocyc   kegg  12.9 0.0  3  N1-Acetylspermidine  C00612  3.916
  2  beta-Alanine metabolism 4e-05  3  Bacterial secretion system 0.000 &nbsp &nbsp &nbsp
   ygcG   ecocyc   kegg   phen   176  yabP  ecocyc   kegg  8.1 0.0 &nbsp &nbsp &nbsp
 &nbsp &nbsp &nbsp  1  Fructose and mannose metabolism 0.005 &nbsp &nbsp &nbsp
   ygcN   ecocyc   kegg   phen   388  yjeK  ecocyc   kegg  13.5 0.0  2  N2-Acetyl-L-ornithine  C00437  -3.627
 &nbsp &nbsp &nbsp  14  Chlorocyclohexane and chlorobenzene degradation 0.000  28  5-amino-1-(5-phospho-D-ribosyl)imidazole-4-carboxylate 0.000 0.000
   ygcO   ecocyc   kegg   phen   58  yfiB  ecocyc   kegg  8.0 0.0 &nbsp &nbsp &nbsp
  1  Biosynthesis of secondary metabolites 0  2  Polyketide sugar unit biosynthesis 0.003 &nbsp &nbsp &nbsp
   ygcP   ecocyc   kegg   phen   127  yccJ  ecocyc   kegg  9.6 0.0  2  2-Demethylmenaquinone 8  C05818  3.547
  2  Arginine and proline metabolism 0.001 &nbsp &nbsp &nbsp  5  5-Phospho-beta-D-ribosylamine 0.000 0.000
   ygcQ   ecocyc   kegg   phen   210  nadR  ecocyc   kegg  8.8 0.0  3  Cytidine  C00475  3.829
  5  Biotin metabolism 5e-05 &nbsp &nbsp &nbsp  7  glucosyl-O-acetyl-rhamanosyl-N-acetylglucosamyl-undecaprenyl diphosphate 0.000 0.000
   ygcR   ecocyc   kegg   phen   321  thrL  ecocyc   kegg  13.7 0.0  2  Fe(III)hydroxamate  C06227  3.650
  10  Aminobenzoate degradation 3e-06  1  Mismatch repair 0.006  11  glucosyl-O-acetyl-rhamanosyl-N-acetylglucosamyl-undecaprenyl diphosphate 0.000 0.000
   ygcS   ecocyc   kegg   phen   345  yddM  ecocyc   kegg  11.4 0.0 &nbsp &nbsp &nbsp
 &nbsp &nbsp &nbsp  9  Chlorocyclohexane and chlorobenzene degradation 0.000 &nbsp &nbsp &nbsp
   ygcU   ecocyc   kegg   phen   210  yqaC  ecocyc   kegg  9.7 0.0  5  D-Glucosamine 1-phosphate  C06156  4.100
  2  Limonene and pinene degradation 0 &nbsp &nbsp &nbsp  14  O-acetyl-rhamanosyl-N-acetylglucosamyl-undecaprenyl diphosphate 0.000 0.000
   ygdB   ecocyc   kegg   phen   173  ubiH  ecocyc   kegg  12.2 0.0  7  Trehalose  C01083  3.687
  9  C5-Branched dibasic acid metabolism 4e-10  2  Sulfur relay system 0.008  8  Ornithine 0.000 0.000
   ygdD   ecocyc   kegg   phen   200  iscA  ecocyc   kegg  13.2 0.0  14  1,2-Diacyl-sn-glycerol (ditetradecanoyl, n-C14:0)  C00641  4.293
  10  Pentose and glucuronate interconversions 9e-15  4  Pentose and glucuronate interconversions 0.001  9  D-Glycerate 2-phosphate 0.000 0.000
   ygdH   ecocyc   kegg   phen   79  elbA  ecocyc   kegg  7.1 0.0  3  N-Acetyl-L-glutamate  C00624  3.471
  3  Lysine biosynthesis 0.002  9  Caprolactam degradation 0.002  5  2,3-dihydroxicinnamic acid 0.000 0.000
   ygdI   ecocyc   kegg   phen   182  bolA  ecocyc   kegg  9.2 0.0 &nbsp &nbsp &nbsp
 &nbsp &nbsp &nbsp  3  Chlorocyclohexane and chlorobenzene degradation 0.000 &nbsp &nbsp &nbsp
   ygdQ   ecocyc   kegg   phen   105  bipA  ecocyc   kegg  11.6 0.0  16  3-Hydroxypropanoate  C01013  3.523
  29  D-Alanine metabolism 3e-07  1  Bacterial secretion system 0.002  2  Propanoyl-CoA 0.001 0.000
   ygdR   ecocyc   kegg   phen   105  deoA  ecocyc   kegg  11.1 0.0  1  2-Acyl-sn-glycero-3-phosphoethanolamine (n-C18:1)  C05973  3.684
  1  Biosynthesis of secondary metabolites 0  14  Pyrimidine metabolism 0.000  23  2,3-dehydroadipyl-CoA 0.000 0.000
   ygeA   ecocyc   kegg   phen   80  ybhK  ecocyc   kegg  13.3 0.0  7  ferroxamine minus Fe(3)  ferroxamine minus Fe(3)  3.795
  7  Inositol phosphate metabolism 4e-05  8  Chlorocyclohexane and chlorobenzene degradation 0.000  6  Pyridoxine 5'-phosphate 0.000 0.000
   ygeF   ecocyc   kegg   phen   144  zraS  ecocyc   kegg  10.3 0.0  43  8-Amino-7-oxononanoate  C01092  5.339
  24  Propanoate metabolism 6e-09  9  Oxidative phosphorylation 0.000  10  Fumarate 0.000 1.000
   ygeG   ecocyc   kegg   phen   95  ycaK  ecocyc   kegg  18.3 0.0  12  L-tartrate  C00898  7.022
 &nbsp &nbsp &nbsp  4  D-Glutamine and D-glutamate metabolism 0.001  5  O-Acetyl-L-serine 0.000 0.000
   ygeH   ecocyc   kegg   phen   84  sodC  ecocyc   kegg  17.4 0.0  20  Succinate  C00042  6.736
  5  Arginine and proline metabolism 8e-06  13  Phenylalanine, tyrosine and tryptophan biosynthesis 0.000  16  2-Octaprenyl-3-methyl-5-hydroxy-6-methoxy-1,4-benzoquinol 0.000 0.000
   ygeI   ecocyc   kegg   phen   195  glyS  ecocyc   kegg  9.9 0.0  4  D-Glucosamine 1-phosphate  C06156  4.278
 &nbsp &nbsp &nbsp  4  Peptidoglycan biosynthesis 0.002  9  Ornithine 0.000 0.000
   ygeK   ecocyc   kegg   phen   211  yqaC  ecocyc   kegg  9.4 0.0  13  Glycolaldehyde  C00266  5.790
  19  Peptidoglycan biosynthesis 0.0001  1  Mismatch repair 0.008  5  5-Dehydro-4-deoxy-D-glucarate 0.000 0.000
   ygeL   ecocyc   kegg   phen   205  htpX  ecocyc   kegg  11.5 0.0  9  Oxalate  C00209  4.068
  3  Arachidonic acid metabolism 0  3  Pentose phosphate pathway 0.002  7  silver 0.000 0.000
   ygeM   ecocyc   kegg   phen   134  thiF  ecocyc   kegg  7.7 0.0 &nbsp &nbsp &nbsp
 &nbsp &nbsp &nbsp  4  Bisphenol degradation 0.001 &nbsp &nbsp &nbsp
   ygeO   ecocyc   kegg   phen   139  yjeM  ecocyc   kegg  18.6 0.0  19  N-Acetylneuraminate  C00270  7.280
  15  alpha-Linolenic acid metabolism 3e-08  5  Sulfur relay system 0.000  4  Bicarbonate 0.000 0.000
   ygeP   ecocyc   kegg   phen   157  ycgZ  ecocyc   kegg  7.0 0.0  1  Hydrogen sulfide  C00283  4.035
  2  Terpenoid backbone biosynthesis 0  3  Propanoate metabolism 0.004  10  glucosyl-O-acetyl-rhamanosyl-N-acetylglucosamyl-undecaprenyl diphosphate 0.000 0.000
   ygeQ   ecocyc   kegg   phen   293  nadR  ecocyc   kegg  10.4 0.0 &nbsp &nbsp &nbsp
 &nbsp &nbsp &nbsp &nbsp &nbsp &nbsp &nbsp &nbsp &nbsp
   ygeV   ecocyc   kegg   phen   113  rihB  ecocyc   kegg  9.3 0.0  1  Cytosine  C00380  -3.665
 &nbsp &nbsp &nbsp  12  Purine metabolism 0.001  9  (3S)-3-Hydroxyadipyl-CoA 0.000 0.000
   ygeW   ecocyc   kegg   phen   144  yicR  ecocyc   kegg  9.8 0.0  1  Thiosulfate  C00320  4.134
 &nbsp &nbsp &nbsp  1  Starch and sucrose metabolism 0.006  4  Fe3+ 0.000 0.000
   ygeY   ecocyc   kegg   phen   95  dacB  ecocyc   kegg  12.8 0.0  11  2-Hydroxy-6-oxonona-2,4-diene-1,9-dioate  C04479  3.694
  2  Porphyrin and chlorophyll metabolism 2e-05  5  Peptidoglycan biosynthesis 0.000  7  undecaprenyl phosphate-4-amino-4-deoxy-L-arabinose 0.000 0.000
   ygfA   ecocyc   kegg   phen   73  yhbC  ecocyc   kegg  6.6 0.0  2  7,8-Diaminononanoate  C01037  3.770
  2  Caprolactam degradation 0  6  Cyanoamino acid metabolism 0.000  5  L-Allo-threonine 0.000 0.000
   ygfB   ecocyc   kegg   phen   159  yhiK  ecocyc   kegg  9.3 0.0  1  Propanal  C00479  5.902
 &nbsp &nbsp &nbsp &nbsp &nbsp &nbsp  11  Glycerol 2-phosphate 0.000 0.000
   ygfF   ecocyc   kegg   phen   31  yiiQ  ecocyc   kegg  24.1 0.0  10  N-Acetylneuraminate  C00270  10.574
  3  alpha-Linolenic acid metabolism 7e-11  11  Inositol phosphate metabolism 0.000  2  Glyceraldehyde 3-phosphate 0.000 0.000
   ygfI   ecocyc   kegg   phen   188  ygaY  ecocyc   kegg  7.8 0.0  12  D-Glucosamine 1-phosphate  C06156  3.567
  10  Microbial metabolism in diverse environments 9e-13 &nbsp &nbsp &nbsp  7  5-Dehydro-4-deoxy-D-glucarate 0.000 0.000
   ygfQ   ecocyc   kegg   phen   112  bcsF  ecocyc   kegg  6.7 0.0 &nbsp &nbsp &nbsp
 &nbsp &nbsp &nbsp  2  Bisphenol degradation 0.001 &nbsp &nbsp &nbsp
   ygfS   ecocyc   kegg   phen   167  yidL  ecocyc   kegg  6.8 0.0  1  Thiosulfate  C00320  3.604
 &nbsp &nbsp &nbsp  2  Propanoate metabolism 0.005  9  glucosyl-O-acetyl-rhamanosyl-N-acetylglucosamyl-undecaprenyl diphosphate 0.000 0.000
   ygfT   ecocyc   kegg   phen   133  ygfU  ecocyc   kegg  8.5 0.0 &nbsp &nbsp &nbsp
 &nbsp &nbsp &nbsp  2  Bisphenol degradation 0.001 &nbsp &nbsp &nbsp
   ygfU   ecocyc   kegg   phen   110  gabD  ecocyc   kegg  10.4 0.0 &nbsp &nbsp &nbsp
 &nbsp &nbsp &nbsp  6  Phosphonate and phosphinate metabolism 0.001 &nbsp &nbsp &nbsp
   ygfX   ecocyc   kegg   phen   64  yggW  ecocyc   kegg  9.6 0.0  4  2-Oxoglutarate  C00026  4.242
  14  Chlorocyclohexane and chlorobenzene degradation 2e-08  9  Caprolactam degradation 0.002  1  Malonate semialdehyde 0.000 0.000
   ygfZ   ecocyc   kegg   phen   86  fdx  ecocyc   kegg  12.7 0.0  77  5'-deoxyribose  5'-deoxyribose  11.134
  13  Valine, leucine and isoleucine biosynthesis 2e-09  13  Biosynthesis of siderophore group nonribosomal peptides 0.000  6  2,3-Dihydro-2,3-dihydroxybenzoate 0.000 0.000
   yggC   ecocyc   kegg   phen   72  dcm  ecocyc   kegg  9.1 0.0  1  Formate  C00058  3.808
 &nbsp &nbsp &nbsp  3  Sulfur relay system 0.000  8  MoaD Protein with carboxylate 0.000 0.000
   yggD   ecocyc   kegg   phen   70  ydjL  ecocyc   kegg  10.0 0.0  3  N-Acetyl-L-glutamate  C00624  4.319
  4  Limonene and pinene degradation 1e-11  3  Glycine, serine and threonine metabolism 0.000  1  L-Threonine 0.001 0.000
   yggE   ecocyc   kegg   phen   83  yheU  ecocyc   kegg  8.0 0.0 &nbsp &nbsp &nbsp
 &nbsp &nbsp &nbsp  9  Bisphenol degradation 0.000 &nbsp &nbsp &nbsp
   yggG   ecocyc   kegg   phen   105  ypdF  ecocyc   kegg  11.2 0.0  3  6-Acetyl-D-glucose  C02655  3.815
 &nbsp &nbsp &nbsp  2  Porphyrin and chlorophyll metabolism 0.009  5  undecaprenyl phosphate-4-amino-4-formyl-L-arabinose 0.000 0.000
   yggL   ecocyc   kegg   phen   235  prmA  ecocyc   kegg  15.8 0.0  39  Sulfate  C00059  7.532
  13  Nitrotoluene degradation 3e-06  3  Ribosome 0.000  31  Deoxyuridine 0.005 1.000
   yggM   ecocyc   kegg   phen   71  yqjA  ecocyc   kegg  8.3 0.0  6  Hydroxypyruvate  C00168  5.553
  5  Fructose and mannose metabolism 3e-05  2  Lipoic acid metabolism 0.001  2  2-Demethylmenaquinone 8 0.003 0.000
   yggN   ecocyc   kegg   phen   92  sgcQ  ecocyc   kegg  34.0 0.0  45  1-(2-Carboxyphenylamino)-1-deoxy-D-ribulose 5-phosphate  C01302  44.705
  11  One carbon pool by folate 0  1  Fructose and mannose metabolism 0.010  1  D-Fructose 1,6-bisphosphate 0.000 0.000
   yggP   ecocyc   kegg   phen   82  yiaY  ecocyc   kegg  14.8 0.0  22  L-Lactate  C00186  5.776
  2  Chloroalkane and chloroalkene degradation 1e-05  14  Ubiquinone and other terpenoid-quinone biosynthesis 0.000  8  2-Octaprenyl-6-methoxy-1,4-benzoquinol 0.000 0.000
   yggR   ecocyc   kegg   phen   120  lysS  ecocyc   kegg  13.7 0.0  3  2-Acyl-sn-glycero-3-phosphoethanolamine (n-C14:1)  C05973  3.674
  13  Purine metabolism 0  5  Aminoacyl-tRNA biosynthesis 0.001  27  5-Phospho-beta-D-ribosylamine 0.000 0.000
   yggS   ecocyc   kegg   phen   283  pbl  ecocyc   kegg  9.8 0.0  8  D-Glucosamine 1-phosphate  C06156  3.819
  4  Lysine biosynthesis 0.0002 &nbsp &nbsp &nbsp  9  5-Dehydro-4-deoxy-D-glucarate 0.000 0.000
   yggT   ecocyc   kegg   phen   76  yggM  ecocyc   kegg  7.0 0.0  1  L-Tyrosine  C00082  3.645
  1  Tryptophan metabolism 0  3  Arachidonic acid metabolism 0.000  3  5-Phospho-alpha-D-ribose 1-diphosphate 0.001 0.000
   yggU   ecocyc   kegg   phen   102  ampE  ecocyc   kegg  11.1 0.0  2  gamma-butyrobetaine  C01181  3.814
  1  Lysine biosynthesis 0.0009  4  Phosphonate and phosphinate metabolism 0.001  6  Dimethyl sulfide 0.000 0.000
   yggW   ecocyc   kegg   phen   102  ygfX  ecocyc   kegg  9.6 0.0  7  3-Phosphohydroxypyruvate  C03232  3.751
  13  Chlorocyclohexane and chlorobenzene degradation 8e-08  3  Novobiocin biosynthesis 0.004  7  D-tartrate 0.000 0.000
   yghA   ecocyc   kegg   phen   231  fkpA  ecocyc   kegg  10.8 0.0  1  methanesulfonate  C11145  -3.580
 &nbsp &nbsp &nbsp  6  Flagellar assembly 0.000  29  bis-molybdopterin guanine dinucleotide 0.000 0.000
   yghB   ecocyc   kegg   phen   139  yegX  ecocyc   kegg  8.4 0.0  2  Dodecanoly-phosphate (n-C12:0)  Dodecanoly-phosphate (n-C12:0)  3.602
  3  Biosynthesis of secondary metabolites 3e-08  1  Glycerolipid metabolism 0.006  5  Cys-Gly 0.001 0.000
   yghD   ecocyc   kegg   phen   103  actP  ecocyc   kegg  7.0 0.0  4  Hydroxypyruvate  C00168  4.000
  3  Glycerolipid metabolism 1e-07  14  Geraniol degradation 0.000  17  Acetoacetyl-CoA 0.000 0.000
   yghF   ecocyc   kegg   phen   263  yghX  ecocyc   kegg  21.3 0.0  128  6-phospho-D-glucono-1,5-lactone  C01236  6.317
  18  Purine metabolism 1e-07  6  Chlorocyclohexane and chlorobenzene degradation 0.000  30  Galactitol 1-phosphate 0.005 1.000
   yghG   ecocyc   kegg   phen   59  spr  ecocyc   kegg  5.3 0.0  9  Hexanoate (n-C6:0)  C01585  4.542
  6  Toluene degradation 3e-09  9  Novobiocin biosynthesis 0.000  1  2-Oxoglutarate 0.004 0.000
   yghJ   ecocyc   kegg   phen   100  srlE  ecocyc   kegg  7.0 0.0  2  octanoate (n-C8:0)  C06423  4.959
 &nbsp &nbsp &nbsp &nbsp &nbsp &nbsp  1  D-Sorbitol 6-phosphate 0.000 0.000
   yghO   ecocyc   kegg   phen   299  yohG  ecocyc   kegg  10.8 0.0  3  2,3-dihydroxicinnamic acid  C12623  -4.068
 &nbsp &nbsp &nbsp  2  Chlorocyclohexane and chlorobenzene degradation 0.000  12  3-Carboxy-2-hydroxy-4-methylpentanoate 0.000 0.000
   yghQ   ecocyc   kegg   phen   167  hslR  ecocyc   kegg  9.5 0.0  2  Bicarbonate  C00288  3.506
  1  Arginine and proline metabolism 0  5  Protein export 0.000  10  Glycerol 2-phosphate 0.000 0.000
   yghR   ecocyc   kegg   phen   124  rfaQ  ecocyc   kegg  9.5 0.0  5  N-Acetyl-L-glutamate  C00624  4.266
  4  Lysine degradation 2e-08  3  Arginine and proline metabolism 0.002  9  5,10-Methylenetetrahydrofolate 0.001 0.000
   yghS   ecocyc   kegg   phen   169  rbbA  ecocyc   kegg  11.0 0.0  1  dehydroglycine  C15809  -4.085
 &nbsp &nbsp &nbsp  6  Ribosome 0.000  8  N-Acetyl-D-glucosamine(anhydrous)N-Acetylmuramic acid 0.000 0.000
   yghT   ecocyc   kegg   phen   67  nrdI  ecocyc   kegg  17.3 0.0  6  Hypoxanthine  C00262  9.236
  16  Caprolactam degradation 2e-06  5  D-Glutamine and D-glutamate metabolism 0.000  6  O-Acetyl-L-serine 0.000 0.000
   yghW   ecocyc   kegg   phen   101  yjeK  ecocyc   kegg  9.1 0.0 &nbsp &nbsp &nbsp
 &nbsp &nbsp &nbsp  14  Chlorocyclohexane and chlorobenzene degradation 0.000 &nbsp &nbsp &nbsp
   ygiB   ecocyc   kegg   phen   197  yqeC  ecocyc   kegg  8.4 0.0  1  Sulfate  C00059  -3.809
 &nbsp &nbsp &nbsp  1  Ribosome 0.000  17  N-Acetyl-D-glucosamine(anhydrous)N-Acetylmuramic acid 0.000 0.000
   ygiC   ecocyc   kegg   phen   95  yjeK  ecocyc   kegg  9.0 0.0 &nbsp &nbsp &nbsp
 &nbsp &nbsp &nbsp  8  Chlorocyclohexane and chlorobenzene degradation 0.000 &nbsp &nbsp &nbsp
   ygiF   ecocyc   kegg   phen   95  htrG  ecocyc   kegg  9.7 0.0 &nbsp &nbsp &nbsp
  1  Biosynthesis of secondary metabolites 0  13  Geraniol degradation 0.000 &nbsp &nbsp &nbsp
   ygiL   ecocyc   kegg   phen   164  yafO  ecocyc   kegg  12.6 0.0  1  N-Acetyl-L-glutamyl 5-phosphate  C04133  3.850
 &nbsp &nbsp &nbsp  9  Lysine degradation 0.001  8  D-Galactarate 0.000 0.000
   ygiV   ecocyc   kegg   phen   116  yqgA  ecocyc   kegg  16.0 0.0  3  Hexadecenoate (n-C16:1)  C08362  3.551
  7  Limonene and pinene degradation 0.0002  10  Geraniol degradation 0.001  16  Acetoacetyl-CoA 0.000 0.000
   ygiW   ecocyc   kegg   phen   101  ushA  ecocyc   kegg  7.3 0.0  9  Dihydroxyacetone  C00184  3.655
 &nbsp &nbsp &nbsp  9  Novobiocin biosynthesis 0.000  9  bis-molybdenum cofactor 0.000 0.000
   ygiZ   ecocyc   kegg   phen   80  ycaK  ecocyc   kegg  14.4 0.0  7  L-tartrate  C00898  6.834
 &nbsp &nbsp &nbsp  4  D-Glutamine and D-glutamate metabolism 0.000  7  O-Acetyl-L-serine 0.000 0.000
   ygjH   ecocyc   kegg   phen   81  ybiS  ecocyc   kegg  9.7 0.0  13  L-Lyxose  C01508  4.015
 &nbsp &nbsp &nbsp  5  Aminobenzoate degradation 0.001  12  Acetoacetate 0.000 0.000
   ygjI   ecocyc   kegg   phen   29  yqjE  ecocyc   kegg  30.3 0.0  10  2-Acyl-sn-glycero-3-phosphoethanolamine (n-C16:1)  C05973  19.707
  1  Biosynthesis of secondary metabolites 0  5  Two-component system 0.002  1  L-Serine 0.000 1.000
   ygjJ   ecocyc   kegg   phen   90  ptsG  ecocyc   kegg  7.6 0.0  4  L-Histidinol phosphate  C01100  3.550
 &nbsp &nbsp &nbsp  6  Phosphotransferase system (PTS) 0.000  2  Arbutin 6-phosphate 0.000 0.000
   ygjK   ecocyc   kegg   phen   49  mhpB  ecocyc   kegg  21.6 0.0  6  Sarcosine  C00213  3.592
  11  Arachidonic acid metabolism 0  2  Glycolysis / Gluconeogenesis 0.007  1  D-Glucose 6-phosphate 0.001 0.000
   ygjP   ecocyc   kegg   phen   161  zapA  ecocyc   kegg  7.6 0.0  3  Glycolaldehyde  C00266  -3.642
 &nbsp &nbsp &nbsp  3  Ribosome 0.000  8  Citrate 0.000 0.000
   ygjQ   ecocyc   kegg   phen   75  ygjI  ecocyc   kegg  12.7 0.0  4  cAMP  C00575  3.731
  1  Lysine degradation 9e-05  7  Chlorocyclohexane and chlorobenzene degradation 0.000  1  dATP 0.000 0.000
   ygjR   ecocyc   kegg   phen   135  yjeK  ecocyc   kegg  12.2 0.0 &nbsp &nbsp &nbsp
  2  Biosynthesis of unsaturated fatty acids 1e-07  15  Chlorocyclohexane and chlorobenzene degradation 0.000 &nbsp &nbsp &nbsp
   ygjV   ecocyc   kegg   phen   138  yaiI  ecocyc   kegg  16.0 0.0  19  N1-Acetylspermidine  C00612  3.608
  7  Glycerolipid metabolism 2e-05  3  Lipoic acid metabolism 0.004  3  GTP 0.001 0.000
   yhaB   ecocyc   kegg   phen   126  ygcM  ecocyc   kegg  11.4 0.0  18  Cytosine  C00380  4.699
  9  Porphyrin and chlorophyll metabolism 1e-05  4  Ribosome 0.001  11  L-Lysine-tRNA (Lys) 0.000 0.000
   yhaC   ecocyc   kegg   phen   126  ydgJ  ecocyc   kegg  8.7 0.0  16  N2-Acetyl-L-ornithine  C00437  -4.112
  9  Lipoic acid metabolism 0  2  Mismatch repair 0.000  2  Uroporphyrinogen III 0.000 0.000
   yhaH   ecocyc   kegg   phen   125  yafQ  ecocyc   kegg  9.6 0.0  9  5-Phospho-beta-D-ribosylamine  C03090  3.618
 &nbsp &nbsp &nbsp  6  Phosphonate and phosphinate metabolism 0.001  6  Molybdate 0.000 0.000
   yhaI   ecocyc   kegg   phen   128  ybgT  ecocyc   kegg  14.3 0.0  12  (R)-Pantothenate  C00864  5.150
  6  Valine, leucine and isoleucine degradation 6e-08  6  Phosphonate and phosphinate metabolism 0.001  16  Hexadecenoyl-CoA (n-C16:1CoA) 0.000 0.000
   yhaJ   ecocyc   kegg   phen   88  uidB  ecocyc   kegg  8.1 0.0  1  Glycerol  C00116  3.992
 &nbsp &nbsp &nbsp  16  Novobiocin biosynthesis 0.000  2  Melibiose 0.000 0.000
   yhaK   ecocyc   kegg   phen   127  fbp  ecocyc   kegg  10.1 0.0  53  N-Acetyl-L-glutamate  C00624  6.360
  20  Lysine degradation 8e-08  13  RNA polymerase 0.000  15  L-Malate 0.000 0.000
   yhaL   ecocyc   kegg   phen   118  tsr  ecocyc   kegg  6.7 0.0 &nbsp &nbsp &nbsp
 &nbsp &nbsp &nbsp  7  Propanoate metabolism 0.001 &nbsp &nbsp &nbsp
   yhaM   ecocyc   kegg   phen   142  carB  ecocyc   kegg  7.3 0.0  8  octanoate (n-C8:0)  C06423  5.272
  3  Vitamin B6 metabolism 0.0001  10  Sulfur metabolism 0.001  8  Hydrogen sulfide 0.000 0.000
   yhaO   ecocyc   kegg   phen   107  thiG  ecocyc   kegg  9.4 0.0  4  dehydroglycine  C15809  4.373
 &nbsp &nbsp &nbsp  9  Valine, leucine and isoleucine biosynthesis 0.000  4  2-Dehydro-3-deoxy-D-gluconate 6-phosphate 0.000 0.000
   yhbE   ecocyc   kegg   phen   324  yjeK  ecocyc   kegg  10.5 0.0  9  Pyridoxamine  C00534  4.573
  2  Toluene degradation 0  7  Chlorocyclohexane and chlorobenzene degradation 0.000  32  4-Phospho-L-aspartate 0.001 0.000
   yhbJ   ecocyc   kegg   phen   79  nagA  ecocyc   kegg  16.4 0.0  16  UDP-N-acetyl-D-mannosamine  C01170  23.723
  5  Amino sugar and nucleotide sugar metabolism 6e-08  12  Chlorocyclohexane and chlorobenzene degradation 0.000  10  5-Methyltetrahydrofolate 0.000 0.000
   yhbO   ecocyc   kegg   phen   252  rtcB  ecocyc   kegg  8.5 0.0  5  tetradecanoate (n-C14:0)  C06424  3.634
  3  Biosynthesis of secondary metabolites 1e-06  2  Peptidoglycan biosynthesis 0.004  7  Succinic semialdehyde 0.000 0.000
   yhbP   ecocyc   kegg   phen   128  nrfF  ecocyc   kegg  11.3 0.0  3  dGMP  C00362  3.821
  1  Tyrosine metabolism 0  6  Nucleotide excision repair 0.000  6  ADP-L-glycero-D-manno-heptose 0.000 0.000
   yhbQ   ecocyc   kegg   phen   191  wcaE  ecocyc   kegg  8.6 0.0  4  Glycolaldehyde  C00266  4.847
 &nbsp &nbsp &nbsp  7  Flagellar assembly 0.000  10  bis-molybdenum cofactor 0.000 0.000
   yhbU   ecocyc   kegg   phen   78  frvX  ecocyc   kegg  10.7 0.0  1  1,4-Dihydroxy-2-naphthoate  C03657  4.097
  6  Nitrotoluene degradation 7e-06  7  Phosphonate and phosphinate metabolism 0.000  5  D-erythro-1-(Imidazol-4-yl)glycerol 3-phosphate 0.000 0.000
   yhbW   ecocyc   kegg   phen   122  flgK  ecocyc   kegg  9.6 0.0  5  3-Methyl-2-oxobutanoate  C00141  5.104
  12  Microbial metabolism in diverse environments 0  7  Ascorbate and aldarate metabolism 0.000  10  3-Dehydro-L-gulonate 0.000 0.000
   yhbX   ecocyc   kegg   phen   146  yghU  ecocyc   kegg  9.1 0.0  1  Nitrite  C00088  3.855
 &nbsp &nbsp &nbsp  2  Ribosome 0.000  14  arsenite 0.000 0.000
   yhbY   ecocyc   kegg   phen   105  narY  ecocyc   kegg  12.5 0.0  5  2-Oxopent-4-enoate  C00596  8.914
  14  Glycerolipid metabolism 1e-06  4  Riboflavin metabolism 0.000  1  L-Xylulose 5-phosphate 0.000 0.000
   yhcA   ecocyc   kegg   phen   165  gadX  ecocyc   kegg  7.4 0.0  7  1,4-Dihydroxy-2-naphthoate  C03657  3.480
 &nbsp &nbsp &nbsp  10  Chlorocyclohexane and chlorobenzene degradation 0.000  10  (S)-3-Methyl-2-oxopentanoate 0.000 0.000
   yhcB   ecocyc   kegg   phen   122  pphA  ecocyc   kegg  11.2 0.0  3  N1-Acetylspermidine  C00612  4.108
  3  Microbial metabolism in diverse environments 0  22  Sulfur relay system 0.000  16  ADP-D-glycero-D-manno-heptose 0.000 0.000
   yhcC   ecocyc   kegg   phen   133  dinG  ecocyc   kegg  8.3 0.0 &nbsp &nbsp &nbsp
 &nbsp &nbsp &nbsp &nbsp &nbsp &nbsp &nbsp &nbsp &nbsp
   yhcD   ecocyc   kegg   phen   120  priC  ecocyc   kegg  15.9 0.0  7  (S)-Dihydroorotate  C00337  3.538
  13  Microbial metabolism in diverse environments 3e-06  21  Microbial metabolism in diverse environments 0.000  9  Malonate semialdehyde 0.000 0.000
   yhcF   ecocyc   kegg   phen   135  rnk  ecocyc   kegg  10.1 0.0  1  Phosphatidylglycerol (ditetradecanoyl, n-C14:0)  C00344  4.109
 &nbsp &nbsp &nbsp  8  Benzoate degradation 0.001  16  alpha-D-Galactose 1-phosphate 0.000 0.000
   yhcG   ecocyc   kegg   phen   109  yhfS  ecocyc   kegg  8.2 0.0 &nbsp &nbsp &nbsp
 &nbsp &nbsp &nbsp  2  alpha-Linolenic acid metabolism 0.002 &nbsp &nbsp &nbsp
   yhcH   ecocyc   kegg   phen   95  yhcG  ecocyc   kegg  6.8 0.0 &nbsp &nbsp &nbsp
 &nbsp &nbsp &nbsp  1  Ribosome 0.004 &nbsp &nbsp &nbsp
   yhcN   ecocyc   kegg   phen   101  yacG  ecocyc   kegg  6.6 0.0 &nbsp &nbsp &nbsp
 &nbsp &nbsp &nbsp  5  Valine, leucine and isoleucine biosynthesis 0.000 &nbsp &nbsp &nbsp
   yhcO   ecocyc   kegg   phen   150  yjeK  ecocyc   kegg  8.4 0.0  2  dUMP  C00365  -3.555
  6  Biosynthesis of secondary metabolites 0.0001  7  Pantothenate and CoA biosynthesis 0.000  14  4-Phospho-L-aspartate 0.000 0.000
   yhdE   ecocyc   kegg   phen   73  yiaJ  ecocyc   kegg  4.7 0.0 &nbsp &nbsp &nbsp
  3  Biosynthesis of secondary metabolites 0  6  Chlorocyclohexane and chlorobenzene degradation 0.000 &nbsp &nbsp &nbsp
   yhdH   ecocyc   kegg   phen   69  bax  ecocyc   kegg  7.5 0.0  11  D-Glucosamine 1-phosphate  C06156  7.890
  6  Amino sugar and nucleotide sugar metabolism 3e-07  3  Polyketide sugar unit biosynthesis 0.005 &nbsp &nbsp &nbsp
   yhdN   ecocyc   kegg   phen   174  betT  ecocyc   kegg  8.4 0.0  2  Hydroxypyruvate  C00168  3.717
 &nbsp &nbsp &nbsp  7  Chlorocyclohexane and chlorobenzene degradation 0.000  13  Aerobactin 0.000 0.000
   yhdP   ecocyc   kegg   phen   166  glyS  ecocyc   kegg  12.8 0.0  8  Glycolaldehyde  C00266  6.069
  15  C5-Branched dibasic acid metabolism 8e-11 &nbsp &nbsp &nbsp  1  D-Ribulose 5-phosphate 0.000 0.000
   yhdT   ecocyc   kegg   phen   110  yhgE  ecocyc   kegg  7.8 0.0  9  gamma-glutamyl-gamma aminobutyric acid  C15767  3.494
  4  Glycerolipid metabolism 4e-07  1  Other glycan degradation 0.002  1  L-Xylulose 0.000 0.000
   yhdU   ecocyc   kegg   phen   112  yiaD  ecocyc   kegg  7.1 0.0  2  Inosine  C00294  4.830
 &nbsp &nbsp &nbsp &nbsp &nbsp &nbsp  2  Formate 0.001 0.000
   yhdV   ecocyc   kegg   phen   146  yjeK  ecocyc   kegg  8.0 0.0 &nbsp &nbsp &nbsp
 &nbsp &nbsp &nbsp  16  Chlorocyclohexane and chlorobenzene degradation 0.000 &nbsp &nbsp &nbsp
   yheO   ecocyc   kegg   phen   225  bipA  ecocyc   kegg  14.7 0.0  5  Dihydroxyacetone phosphate  C00111  -3.514
  2  Dioxin degradation 0  6  Protein export 0.000  10  Dihydroxyacetone phosphate 0.010 1.000
   yheT   ecocyc   kegg   phen   125  ypdB  ecocyc   kegg  6.8 0.0  3  Glycerophosphoserine  Glycerophosphoserine  4.551
 &nbsp &nbsp &nbsp  3  Novobiocin biosynthesis 0.000  13  N2-Succinyl-L-arginine 0.000 0.000
   yheU   ecocyc   kegg   phen   88  yhjA  ecocyc   kegg  10.9 0.0  3  (R)-Propane-1,2-diol  C02912  -4.586
  3  Glycerolipid metabolism 1e-07 &nbsp &nbsp &nbsp  6  Maltotriose 0.000 0.000
   yheV   ecocyc   kegg   phen   61  ppiA  ecocyc   kegg  6.7 0.0  8  1,6-anhydrous-N-Acetylmuramyl-tripeptide  1,6-anhydrous-N-Acetylmuramyl-tripeptide  44.082
 &nbsp &nbsp &nbsp &nbsp &nbsp &nbsp  2  Fe3+ 0.000 0.000
   yhfA   ecocyc   kegg   phen   66  relA  ecocyc   kegg  6.3 0.0  12  2-Dehydro-3-deoxy-D-galactonate 6-phosphate  C01286  8.542
  5  Pentose phosphate pathway 2e-06  3  C5-Branched dibasic acid metabolism 0.000 &nbsp &nbsp &nbsp
   yhfG   ecocyc   kegg   phen   78  fdx  ecocyc   kegg  14.5 0.0  40  2-C-methyl-D-erythritol 2,4-cyclodiphosphate  C11453  5.579
  8  C5-Branched dibasic acid metabolism 3e-15  6  Biosynthesis of siderophore group nonribosomal peptides 0.000  10  2,3-Dihydro-2,3-dihydroxybenzoate 0.000 0.000
   yhfK   ecocyc   kegg   phen   166  yjeI  ecocyc   kegg  17.4 0.0  14  crotonobetaine  C04114  4.755
  4  Microbial metabolism in diverse environments 0  7  Chlorocyclohexane and chlorobenzene degradation 0.000  12  ADP-D-glycero-D-manno-heptose 0.000 0.000
   yhfL   ecocyc   kegg   phen   112  yiaJ  ecocyc   kegg  8.7 0.0  7  1,2-Diacyl-sn-glycerol (dioctadec-11-enoyl, n-C18:1)  C00641  4.010
  2  Ubiquinone and other terpenoid-quinone biosynthesis 0  1  Ribosome 0.001  5  Deoxyinosine 0.000 0.000
   yhfS   ecocyc   kegg   phen   82  yrbE  ecocyc   kegg  21.9 0.0  11  Sarcosine  C00213  4.243
  21  Valine, leucine and isoleucine degradation 4e-07  11  Glycolysis / Gluconeogenesis 0.000  4  [4Fe-4S] iron-sulfur cluster 0.000 0.000
   yhfT   ecocyc   kegg   phen   233  ygfJ  ecocyc   kegg  7.1 0.0 &nbsp &nbsp &nbsp
 &nbsp &nbsp &nbsp &nbsp &nbsp &nbsp &nbsp &nbsp &nbsp
   yhfX   ecocyc   kegg   phen   87  rnk  ecocyc   kegg  5.8 0.0  2  Inosine  C00294  3.847
 &nbsp &nbsp &nbsp &nbsp &nbsp &nbsp  1  L-Arginine 0.000 0.000
   yhfY   ecocyc   kegg   phen   166  frlR  ecocyc   kegg  9.4 0.0  2  Pyridoxine  C00314  3.762
  2  Lysine biosynthesis 6e-05  4  Chlorocyclohexane and chlorobenzene degradation 0.000  2  L-Aspartate 0.000 0.000
   yhfZ   ecocyc   kegg   phen   175  glyS  ecocyc   kegg  8.9 0.0 &nbsp &nbsp &nbsp
 &nbsp &nbsp &nbsp &nbsp &nbsp &nbsp &nbsp &nbsp &nbsp
   yhgA   ecocyc   kegg   phen   126  yjeK  ecocyc   kegg  10.5 0.0 &nbsp &nbsp &nbsp
 &nbsp &nbsp &nbsp  4  C5-Branched dibasic acid metabolism 0.000 &nbsp &nbsp &nbsp
   yhgE   ecocyc   kegg   phen   138  yibH  ecocyc   kegg  9.6 0.0  10  Sarcosine  C00213  3.472
  3  Glycerolipid metabolism 1e-07  1  Other glycan degradation 0.004  10  ADPribose 0.000 0.000
   yhgF   ecocyc   kegg   phen   153  trkD  ecocyc   kegg  8.8 0.0  27  D-Methionine  C00855  4.521
  8  Pentose and glucuronate interconversions 1e-10  4  Phosphotransferase system (PTS) 0.001  23  Deoxycytidine 0.001 0.000
   yhgN   ecocyc   kegg   phen   106  yiaC  ecocyc   kegg  8.7 0.0  3  Inosine  C00294  5.068
 &nbsp &nbsp &nbsp  1  Phosphotransferase system (PTS) 0.006  7  L-tartrate 0.000 0.000
   yhhA   ecocyc   kegg   phen   139  deoA  ecocyc   kegg  9.1 0.0  4  D-Glucosamine 1-phosphate  C06156  3.640
 &nbsp &nbsp &nbsp  5  alpha-Linolenic acid metabolism 0.004  10  L-alanine-D-glutamate 0.001 0.000
   yhhH   ecocyc   kegg   phen   147  ydgJ  ecocyc   kegg  9.3 0.0  25  N(omega)-(L-Arginino)succinate  C03406  5.226
  12  C5-Branched dibasic acid metabolism 1e-11  2  Valine, leucine and isoleucine biosynthesis 0.004  10  Dihydroxyacetone phosphate 0.002 1.000
   yhhI   ecocyc   kegg   phen   301  thrL  ecocyc   kegg  14.0 0.0  8  D-Glucosamine 6-phosphate  C00352  -3.787
  10  C5-Branched dibasic acid metabolism 6e-09 &nbsp &nbsp &nbsp  15  glucosyl-O-acetyl-rhamanosyl-N-acetylglucosamyl-undecaprenyl diphosphate 0.000 0.000
   yhhJ   ecocyc   kegg   phen   110  frlR  ecocyc   kegg  8.6 0.0 &nbsp &nbsp &nbsp
 &nbsp &nbsp &nbsp  7  Chlorocyclohexane and chlorobenzene degradation 0.000 &nbsp &nbsp &nbsp
   yhhL   ecocyc   kegg   phen   170  slyD  ecocyc   kegg  10.1 0.0 &nbsp &nbsp &nbsp
 &nbsp &nbsp &nbsp  6  Selenoamino acid metabolism 0.000 &nbsp &nbsp &nbsp
   yhhM   ecocyc   kegg   phen   168  yjeK  ecocyc   kegg  12.8 0.0  1  Phenylacetic acid  C07086  4.062
 &nbsp &nbsp &nbsp  15  Chlorocyclohexane and chlorobenzene degradation 0.000  28  Dephospho-CoA 0.000 0.000
   yhhN   ecocyc   kegg   phen   64  yiaC  ecocyc   kegg  5.5 0.0  5  D-Glucosamine 1-phosphate  C06156  8.048
  6  Amino sugar and nucleotide sugar metabolism 1e-08  5  Ribosome 0.001  3  L-Tryptophan 0.000 0.000
   yhhS   ecocyc   kegg   phen   126  tatB  ecocyc   kegg  7.7 0.0 &nbsp &nbsp &nbsp
 &nbsp &nbsp &nbsp  8  Chlorocyclohexane and chlorobenzene degradation 0.000 &nbsp &nbsp &nbsp
   yhhT   ecocyc   kegg   phen   126  eptA  ecocyc   kegg  9.2 0.0 &nbsp &nbsp &nbsp
  1  Biosynthesis of secondary metabolites 0  3  Chlorocyclohexane and chlorobenzene degradation 0.000 &nbsp &nbsp &nbsp
   yhhW   ecocyc   kegg   phen   194  ilvB  ecocyc   kegg  9.7 0.0  7  5-O-(1-Carboxyvinyl)-3-phosphoshikimate  C01269  3.633
  1  Histidine metabolism 0  14  Pantothenate and CoA biosynthesis 0.000  22  D-Tagatose 1,6-biphosphate 0.002 1.000
   yhhX   ecocyc   kegg   phen   28  tas  ecocyc   kegg  12.3 0.0  2  Deoxycytidine  C00881  10.889
  1  Pantothenate and CoA biosynthesis 0  9  alpha-Linolenic acid metabolism 0.000 &nbsp &nbsp &nbsp
   yhhY   ecocyc   kegg   phen   125  yhgN  ecocyc   kegg  7.0 0.0  2  N1-(5-Phospho-D-ribosyl)glycinamide  C03838  4.231
 &nbsp &nbsp &nbsp &nbsp &nbsp &nbsp  2  L-Xylulose 0.000 0.000
   yhhZ   ecocyc   kegg   phen   123  aaeB  ecocyc   kegg  6.5 0.0  3  5,6,7,8-tetrahydropteridine  C05650  3.491
 &nbsp &nbsp &nbsp  4  Novobiocin biosynthesis 0.000  3  Prephenate 0.000 0.000
   yhiD   ecocyc   kegg   phen   92  nirC  ecocyc   kegg  7.3 0.0 &nbsp &nbsp &nbsp
 &nbsp &nbsp &nbsp  4  Protein export 0.000 &nbsp &nbsp &nbsp
   yhiI   ecocyc   kegg   phen   117  atoS  ecocyc   kegg  13.2 0.0  63  N-Acetyl-L-glutamate  C00624  12.566
  18  Lysine degradation 3e-16  6  RNA polymerase 0.000  5  Spermidine 0.002 1.000
   yhiJ   ecocyc   kegg   phen   119  yfaY  ecocyc   kegg  7.2 0.0 &nbsp &nbsp &nbsp
 &nbsp &nbsp &nbsp  3  Arginine and proline metabolism 0.002 &nbsp &nbsp &nbsp
   yhiL   ecocyc   kegg   phen   190  chpB  ecocyc   kegg  15.7 0.0  4  crotonobetaine  C04114  5.089
  2  Fatty acid biosynthesis 8e-06 &nbsp &nbsp &nbsp  14  D-Mannose 0.000 0.000
   yhiM   ecocyc   kegg   phen   197  mobB  ecocyc   kegg  8.3 0.0 &nbsp &nbsp &nbsp
 &nbsp &nbsp &nbsp  4  Chlorocyclohexane and chlorobenzene degradation 0.000 &nbsp &nbsp &nbsp
   yhiN   ecocyc   kegg   phen   103  pyrB  ecocyc   kegg  9.7 0.0  4  L-Phenylalanine  C00079  -4.170
 &nbsp &nbsp &nbsp  8  D-Alanine metabolism 0.002  25  Orotidine 5'-phosphate 0.000 0.000
   yhiQ   ecocyc   kegg   phen   171  emrD  ecocyc   kegg  14.1 0.0 &nbsp &nbsp &nbsp
 &nbsp &nbsp &nbsp  2  Aminoacyl-tRNA biosynthesis 0.003 &nbsp &nbsp &nbsp
   yhiR   ecocyc   kegg   phen   93  yjbG  ecocyc   kegg  9.9 0.0  5  Inosine  C00294  9.097
  6  Biosynthesis of secondary metabolites 0.0001  2  Nicotinate and nicotinamide metabolism 0.003  3  Adenine 0.001 0.000
   yhiS   ecocyc   kegg   phen   127  ydgI  ecocyc   kegg  9.7 0.0  15  N2-Succinyl-L-ornithine  C03415  4.497
  7  Starch and sucrose metabolism 1e-07  1  Porphyrin and chlorophyll metabolism 0.002  15  Uridine 0.003 1.000
   yhjA   ecocyc   kegg   phen   100  yheU  ecocyc   kegg  10.9 0.0  10  Bicarbonate  C00288  3.630
  8  Glycerolipid metabolism 5e-06  3  Sulfur metabolism 0.004  5  Fe3+ 0.000 0.000
   yhjB   ecocyc   kegg   phen   111  gadX  ecocyc   kegg  9.6 0.0  4  D-Glycero-D-manno-heptose 7-phosphate  C07836  4.499
  1  Biosynthesis of secondary metabolites 0  3  Valine, leucine and isoleucine biosynthesis 0.000  9  Adenosine 0.000 0.000
   yhjC   ecocyc   kegg   phen   591  trmA  ecocyc   kegg  13.1 0.0  18  Sedoheptulose 7-phosphate  C05382  3.518
  1  Pyrimidine metabolism 0  4  Chlorocyclohexane and chlorobenzene degradation 0.000  30  (S)-3-Methyl-2-oxopentanoate 0.000 0.000
   yhjD   ecocyc   kegg   phen   86  yhdU  ecocyc   kegg  5.4 0.0  21  Thiamin monophosphate  C01081  5.282
 &nbsp &nbsp &nbsp  2  Porphyrin and chlorophyll metabolism 0.000 &nbsp &nbsp &nbsp
   yhjE   ecocyc   kegg   phen   43  yiaV  ecocyc   kegg  5.7 0.0  8  N1-(5-Phospho-D-ribosyl)glycinamide  C03838  3.815
  6  Biosynthesis of secondary metabolites 0.0001 &nbsp &nbsp &nbsp &nbsp &nbsp &nbsp
   yhjG   ecocyc   kegg   phen   150  yfeN  ecocyc   kegg  7.0 0.0 &nbsp &nbsp &nbsp
 &nbsp &nbsp &nbsp &nbsp &nbsp &nbsp &nbsp &nbsp &nbsp
   yhjJ   ecocyc   kegg   phen   79  agaR  ecocyc   kegg  11.0 0.0  27  N-Methyltryptophan  C02983  4.869
  9  Microbial metabolism in diverse environments 3e-06  2  Peptidoglycan biosynthesis 0.001  2  undecaprenyl phosphate-4-amino-4-deoxy-L-arabinose 0.000 0.000
   yhjK   ecocyc   kegg   phen   163  kdgK  ecocyc   kegg  13.6 0.0 &nbsp &nbsp &nbsp
 &nbsp &nbsp &nbsp  5  Galactose metabolism 0.000 &nbsp &nbsp &nbsp
   yhjR   ecocyc   kegg   phen   121  yjeK  ecocyc   kegg  7.1 0.0  1  Deoxyuridine  C00526  3.489
 &nbsp &nbsp &nbsp  12  Chlorocyclohexane and chlorobenzene degradation 0.000  4  Arbutin 6-phosphate 0.000 0.000
   yhjV   ecocyc   kegg   phen   118  bcsG  ecocyc   kegg  8.2 0.0  4  Putrescine  C00134  -3.686
 &nbsp &nbsp &nbsp  4  Novobiocin biosynthesis 0.000  10  Prephenate 0.000 0.000
   yhjX   ecocyc   kegg   phen   353  ivbL  ecocyc   kegg  11.7 0.0 &nbsp &nbsp &nbsp
 &nbsp &nbsp &nbsp  4  Chlorocyclohexane and chlorobenzene degradation 0.000 &nbsp &nbsp &nbsp
   yhjY   ecocyc   kegg   phen   159  ogrK  ecocyc   kegg  7.8 0.0 &nbsp &nbsp &nbsp
 &nbsp &nbsp &nbsp  1  Thiamine metabolism 0.001 &nbsp &nbsp &nbsp
   yiaA   ecocyc   kegg   phen   162  yieN  ecocyc   kegg  12.4 0.0  4  Hydroquinone  C00530  3.814
  1  Pyrimidine metabolism 0  3  Glycine, serine and threonine metabolism 0.001  6  Deoxyguanosine 0.001 0.000
   yiaB   ecocyc   kegg   phen   138  yiaY  ecocyc   kegg  12.8 0.0  2  cis-Aconitate  C00417  3.746
 &nbsp &nbsp &nbsp  6  Ubiquinone and other terpenoid-quinone biosynthesis 0.000  14  2-Octaprenyl-3-methyl-5-hydroxy-6-methoxy-1,4-benzoquinol 0.000 0.000
   yiaC   ecocyc   kegg   phen   96  yjcF  ecocyc   kegg  10.8 0.0  9  Inosine  C00294  7.105
  9  Pentose and glucuronate interconversions 1e-05  1  Porphyrin and chlorophyll metabolism 0.007  1  L-Methionine 0.000 0.000
   yiaD   ecocyc   kegg   phen   114  yjbI  ecocyc   kegg  8.6 0.0  7  Inosine  C00294  6.967
  6  Biosynthesis of secondary metabolites 0.0001 &nbsp &nbsp &nbsp  2  L-Arginine 0.000 0.000
   yiaF   ecocyc   kegg   phen   120  dipZ  ecocyc   kegg  8.6 0.0 &nbsp &nbsp &nbsp
 &nbsp &nbsp &nbsp  5  Bacterial secretion system 0.000 &nbsp &nbsp &nbsp
   yiaG   ecocyc   kegg   phen   122  yjeK  ecocyc   kegg  7.9 0.0  1  Hexadecanoyl-phosphate (n-C16:1)  Hexadecanoyl-phosphate (n-C16:1)  -3.621
 &nbsp &nbsp &nbsp  5  Pantothenate and CoA biosynthesis 0.000  8  Aerobactin 0.000 0.000
   yiaI   ecocyc   kegg   phen   130  lldR  ecocyc   kegg  17.0 0.0  9  phosphatidylethanolamine (dihexadec-9enoyl, n-C16:1)  C00350  4.764
  1  Fatty acid biosynthesis 0  9  Biotin metabolism 0.000  6  7,8-Diaminononanoate 0.000 0.000
   yiaJ   ecocyc   kegg   phen   140  yhfL  ecocyc   kegg  8.7 0.0  2  2-C-methyl-D-erythritol 2,4-cyclodiphosphate  C11453  3.550
 &nbsp &nbsp &nbsp  3  Other glycan degradation 0.004  10  2,3-dehydroadipyl-CoA 0.000 0.000
   yiaL   ecocyc   kegg   phen   119  yicM  ecocyc   kegg  7.7 0.0  6  Dodecanoly-phosphate (n-C12:0)  Dodecanoly-phosphate (n-C12:0)  4.769
  7  Alanine, aspartate and glutamate metabolism 0.0003  2  Propanoate metabolism 0.008  4  molybdenum cofactor 0.000 0.000
   yiaT   ecocyc   kegg   phen   126  yebG  ecocyc   kegg  11.9 0.0  13  Oxalate  C00209  4.094
  6  alpha-Linolenic acid metabolism 1e-06 &nbsp &nbsp &nbsp &nbsp &nbsp &nbsp
   yiaU   ecocyc   kegg   phen   324  yeaT  ecocyc   kegg  25.8 0.0  45  2-Acyl-sn-glycero-3-phosphoglycerol (n-C16:1)  2-Acyl-sn-glycero-3-phosphoglycerol (n-C16:1)  6.680
  12  Purine metabolism 4e-06  3  RNA polymerase 0.000  25  bis-molybdopterin guanine dinucleotide 0.000 0.000
   yiaW   ecocyc   kegg   phen   150  yibA  ecocyc   kegg  9.7 0.0  3  N2-Succinyl-L-ornithine  C03415  -5.098
 &nbsp &nbsp &nbsp  6  Cysteine and methionine metabolism 0.001  6  5-Methyltetrahydrofolate 0.000 0.000
   yibD   ecocyc   kegg   phen   89  chpB  ecocyc   kegg  7.5 0.0  7  L-Phenylalanine  C00079  3.471
  13  Purine metabolism 0  6  C5-Branched dibasic acid metabolism 0.001  9  5-Phospho-beta-D-ribosylamine 0.000 0.000
   yibF   ecocyc   kegg   phen   289  yagI  ecocyc   kegg  9.0 0.0  7  1,5-Diaminopentane  C01672  3.786
  1  Biosynthesis of secondary metabolites 0 &nbsp &nbsp &nbsp  10  O-acetyl-rhamanosyl-N-acetylglucosamyl-undecaprenyl diphosphate 0.000 0.000
   yibG   ecocyc   kegg   phen   103  csgB  ecocyc   kegg  14.5 0.0  5  Oxalureate  C00802  5.008
 &nbsp &nbsp &nbsp  5  Lipoic acid metabolism 0.002  2  octanoate (n-C8:0) 0.000 0.000
   yibH   ecocyc   kegg   phen   113  yhjA  ecocyc   kegg  10.5 0.0  13  Hydroxypyruvate  C00168  3.991
  8  Glycerolipid metabolism 5e-06  4  alpha-Linolenic acid metabolism 0.003  1  L-Aspartate 0.009 0.000
   yibI   ecocyc   kegg   phen   214  tsr  ecocyc   kegg  9.5 0.0  7  Glycolaldehyde  C00266  -4.090
 &nbsp &nbsp &nbsp  1  Dioxin degradation 0.003  13  crotonobetainyl-CoA 0.000 0.000
   yibL   ecocyc   kegg   phen   161  yjeK  ecocyc   kegg  9.3 0.0 &nbsp &nbsp &nbsp
 &nbsp &nbsp &nbsp  12  Chlorocyclohexane and chlorobenzene degradation 0.000 &nbsp &nbsp &nbsp
   yibN   ecocyc   kegg   phen   110  tsgA  ecocyc   kegg  7.6 0.0  3  N2-Succinyl-L-ornithine  C03415  -3.638
 &nbsp &nbsp &nbsp &nbsp &nbsp &nbsp  3  L-Histidine 0.000 0.000
   yibQ   ecocyc   kegg   phen   124  yicR  ecocyc   kegg  9.1 0.0  6  L-Glutamate 5-semialdehyde  C01165  3.849
  7  Arginine and proline metabolism 5e-06  4  Sphingolipid metabolism 0.001  4  D-Xylulose 5-phosphate 0.000 0.000
   yibT   ecocyc   kegg   phen   106  yibH  ecocyc   kegg  6.8 0.0 &nbsp &nbsp &nbsp
 &nbsp &nbsp &nbsp &nbsp &nbsp &nbsp &nbsp &nbsp &nbsp
   yicC   ecocyc   kegg   phen   148  ilvB  ecocyc   kegg  9.5 0.0  5  Hydroxypyruvate  C00168  3.650
 &nbsp &nbsp &nbsp  8  Pantothenate and CoA biosynthesis 0.000  14  4-Phospho-L-aspartate 0.000 0.000
   yicG   ecocyc   kegg   phen   184  eco  ecocyc   kegg  7.6 0.0  2  Methylglyoxal  C00546  4.156
 &nbsp &nbsp &nbsp  1  Ribosome 0.000  4  arsenite 0.000 0.000
   yicH   ecocyc   kegg   phen   187  yibA  ecocyc   kegg  16.9 0.0  25  3-Hydroxypropanoate  C01013  3.550
  29  D-Alanine metabolism 4e-07  2  D-Alanine metabolism 0.007  3  molybdopterin 0.000 0.000
   yicJ   ecocyc   kegg   phen   334  yidR  ecocyc   kegg  12.2 0.0 &nbsp &nbsp &nbsp
 &nbsp &nbsp &nbsp  8  Caprolactam degradation 0.003 &nbsp &nbsp &nbsp
   yicL   ecocyc   kegg   phen   112  fixA  ecocyc   kegg  6.5 0.0  5  Trehalose  C01083  3.613
  5  Galactose metabolism 7e-12  6  Riboflavin metabolism 0.000  2  nickel 0.000 0.000
   yicO   ecocyc   kegg   phen   79  rarD  ecocyc   kegg  7.0 0.0  2  Succinate  C00042  7.164
  2  Ascorbate and aldarate metabolism 0.0001  6  Chloroalkane and chloroalkene degradation 0.001  1  L-Aspartate 0.006 0.000
   yicS   ecocyc   kegg   phen   213  gadX  ecocyc   kegg  7.9 0.0  17  3-Deoxy-D-manno-2-octulosonate  C01187  3.816
  6  Tyrosine metabolism 2e-07  5  Chlorocyclohexane and chlorobenzene degradation 0.000  16  bis-molybdopterin guanine dinucleotide 0.000 0.000
   yidB   ecocyc   kegg   phen   143  yieN  ecocyc   kegg  11.2 0.0  11  L-Rhamnose  C00507  4.717
 &nbsp &nbsp &nbsp  3  Phosphotransferase system (PTS) 0.005  8  arsenite 0.000 0.000
   yidE   ecocyc   kegg   phen   127  eco  ecocyc   kegg  8.7 0.0  11  4,5-dihydroxy-2,3-pentanedione  C11838  4.034
 &nbsp &nbsp &nbsp  4  Ribosome 0.000  10  arsenite 0.000 0.000
   yidF   ecocyc   kegg   phen   300  thrL  ecocyc   kegg  11.6 0.0 &nbsp &nbsp &nbsp
  6  Microbial metabolism in diverse environments 5e-08 &nbsp &nbsp &nbsp &nbsp &nbsp &nbsp
   yidG   ecocyc   kegg   phen   150  yjdL  ecocyc   kegg  14.6 0.0  41  O-Phospho-L-serine  C01005  4.384
  12  Lysine degradation 3e-06  15  Taurine and hypotaurine metabolism 0.001  5  Adenosine 3',5'-bisphosphate 0.000 0.000
   yidH   ecocyc   kegg   phen   118  dcuA  ecocyc   kegg  10.5 0.0  21  2,3-Dihydro-2,3-dihydroxybenzoate  C04171  4.679
  12  Toluene degradation 3e-07  3  Bacterial secretion system 0.000  17  bis-molybdopterin guanine dinucleotide 0.000 0.000
   yidI   ecocyc   kegg   phen   146  hemX  ecocyc   kegg  6.7 0.0  15  Thiamin monophosphate  C01081  4.357
  5  Purine metabolism 0.0001  6  Bacterial secretion system 0.000  9  Aerobactin 0.000 0.000
   yidJ   ecocyc   kegg   phen   235  yidK  ecocyc   kegg  8.1 0.0  26  3-Methyl-2-oxobutanoate  C00141  6.080
  18  Lysine degradation 8e-08  2  Thiamine metabolism 0.005  20  bis-molybdopterin guanine dinucleotide 0.000 0.000
   yidL   ecocyc   kegg   phen   169  yqeK  ecocyc   kegg  7.4 0.0  1  crotonobetaine  C04114  -4.300
  2  Caprolactam degradation 0  2  Propanoate metabolism 0.005  8  glucosyl-O-acetyl-rhamanosyl-N-acetylglucosamyl-undecaprenyl diphosphate 0.000 0.000
   yidP   ecocyc   kegg   phen   83  pdxK  ecocyc   kegg  6.7 0.0 &nbsp &nbsp &nbsp
 &nbsp &nbsp &nbsp  14  Homologous recombination 0.001 &nbsp &nbsp &nbsp
   yidQ   ecocyc   kegg   phen   137  yhiK  ecocyc   kegg  10.3 0.0 &nbsp &nbsp &nbsp
 &nbsp &nbsp &nbsp  2  Bisphenol degradation 0.001 &nbsp &nbsp &nbsp
   yidX   ecocyc   kegg   phen   165  hchA  ecocyc   kegg  8.9 0.0  4  1,2-Diacyl-sn-glycerol (ditetradecanoyl, n-C14:0)  C00641  4.521
  5  Histidine metabolism 0.002  1  Dioxin degradation 0.001  4  D-Gluconate 0.000 0.000
   yidZ   ecocyc   kegg   phen   296  tap  ecocyc   kegg  18.6 0.0  122  cyclopropane phosphatidylethanolamine (dihexadec-9,10-cyclo-anoyl, n-C16:0 cyclo)  cyclopropane phosphatidylethanolamine (dihexadec-9,10-cyclo-anoyl, n-C16:0 cyclo)  6.645
  15  Arachidonic acid metabolism 0  5  Oxidative phosphorylation 0.000  25  dUTP 0.004 1.000
   yieE   ecocyc   kegg   phen   113  ulaR  ecocyc   kegg  7.2 0.0 &nbsp &nbsp &nbsp
 &nbsp &nbsp &nbsp  3  Nucleotide excision repair 0.001 &nbsp &nbsp &nbsp
   yieH   ecocyc   kegg   phen   131  yieI  ecocyc   kegg  9.6 0.0  28  Inosine  C00294  8.922
  14  Microbial metabolism in diverse environments 2e-05  4  Chlorocyclohexane and chlorobenzene degradation 0.000  4  dATP 0.000 0.000
   yieK   ecocyc   kegg   phen   255  bipA  ecocyc   kegg  14.3 0.0 &nbsp &nbsp &nbsp
 &nbsp &nbsp &nbsp  8  Bacterial secretion system 0.000 &nbsp &nbsp &nbsp
   yieL   ecocyc   kegg   phen   181  bipA  ecocyc   kegg  15.9 0.0  16  Glycine betaine  C00719  6.966
  6  Sulfur metabolism 0.001  12  Nitrogen metabolism 0.001  12  2-Oxobutanoate 0.000 0.000
   yieP   ecocyc   kegg   phen   430  yiiF  ecocyc   kegg  13.0 0.0  5  5-Methylthio-D-ribose  C03089  3.642
 &nbsp &nbsp &nbsp  8  Flagellar assembly 0.001  44  bis-molybdopterin guanine dinucleotide 0.000 0.000
   yifB   ecocyc   kegg   phen   121  pyrL  ecocyc   kegg  15.7 0.0  25  gamma-glutamyl-gamma aminobutyric acid  C15767  10.287
  7  Lipopolysaccharide biosynthesis 2e-09  6  Homologous recombination 0.000  5  Taurine 0.000 0.000
   yifE   ecocyc   kegg   phen   122  yihN  ecocyc   kegg  9.8 0.0 &nbsp &nbsp &nbsp
  5  Aminobenzoate degradation 8e-08  3  Chlorocyclohexane and chlorobenzene degradation 0.000 &nbsp &nbsp &nbsp
   yifK   ecocyc   kegg   phen   191  coaE  ecocyc   kegg  13.5 0.0  2  Ethanol  C00469  3.764
  2  Biosynthesis of secondary metabolites 0  13  Chlorocyclohexane and chlorobenzene degradation 0.000  8  D-Fructose 1,6-bisphosphate 0.000 0.000
   yifL   ecocyc   kegg   phen   120  phnH  ecocyc   kegg  5.7 0.0  3  Cytosine  C00380  3.611
  2  Caprolactam degradation 0.0002 &nbsp &nbsp &nbsp  5  glucosyl-O-acetyl-rhamanosyl-N-acetylglucosamyl-undecaprenyl diphosphate 0.000 0.000
   yifO   ecocyc   kegg   phen   66  gatR  ecocyc   kegg  6.3 0.0 &nbsp &nbsp &nbsp
 &nbsp &nbsp &nbsp  1  C5-Branched dibasic acid metabolism 0.010 &nbsp &nbsp &nbsp
   yigA   ecocyc   kegg   phen   124  yiiU  ecocyc   kegg  14.0 0.0  9  N-Acetyl-L-glutamate  C00624  4.803
  4  Tryptophan metabolism 7e-05  1  Phosphonate and phosphinate metabolism 0.001  2  Succinic semialdehyde 0.000 0.000
   yigB   ecocyc   kegg   phen   136  tauB  ecocyc   kegg  9.3 0.0  3  Inosine  C00294  3.541
  3  Fructose and mannose metabolism 9e-05  1  alpha-Linolenic acid metabolism 0.004  12  Aerobactin 0.000 0.000
   yigE   ecocyc   kegg   phen   146  ygiQ  ecocyc   kegg  11.3 0.0  1  L-seryl-AMP  C05820  -3.773
 &nbsp &nbsp &nbsp  10  Phosphotransferase system (PTS) 0.000  9  (S)-3-Methyl-2-oxopentanoate 0.000 0.000
   yigG   ecocyc   kegg   phen   228  rarD  ecocyc   kegg  14.4 0.0 &nbsp &nbsp &nbsp
 &nbsp &nbsp &nbsp  8  Protein export 0.000 &nbsp &nbsp &nbsp
   yigI   ecocyc   kegg   phen   359  tatB  ecocyc   kegg  20.6 0.0  9  Butanal  C01412  3.864
  1  Butanoate metabolism 0  5  Bacterial secretion system 0.002  27  Aerobactin 0.000 0.000
   yigM   ecocyc   kegg   phen   57  eamA  ecocyc   kegg  5.6 0.0 &nbsp &nbsp &nbsp
 &nbsp &nbsp &nbsp &nbsp &nbsp &nbsp &nbsp &nbsp &nbsp
   yigZ   ecocyc   kegg   phen   156  torZ  ecocyc   kegg  11.9 0.0  22  D-Glucosamine 1-phosphate  C06156  16.753
  3  Biosynthesis of secondary metabolites 2e-10  4  Chloroalkane and chloroalkene degradation 0.005  6  glucosyl-O-acetyl-rhamanosyl-N-acetylglucosamyl-undecaprenyl diphosphate 0.000 0.000
   yihD   ecocyc   kegg   phen   121  yifB  ecocyc   kegg  12.0 0.0  24  Thiamin monophosphate  C01081  9.663
  8  Valine, leucine and isoleucine degradation 1e-07  6  Chlorocyclohexane and chlorobenzene degradation 0.000  8  Aerobactin 0.000 0.000
   yihF   ecocyc   kegg   phen   208  yigI  ecocyc   kegg  20.3 0.0  9  Ethanol  C00469  4.100
  2  Biosynthesis of secondary metabolites 0.0002  10  Flagellar assembly 0.000  15  3-Dehydro-L-gulonate 0.000 0.000
   yihG   ecocyc   kegg   phen   76  yihN  ecocyc   kegg  8.7 0.0  29  Glycine betaine  C00719  10.877
  8  Pentose and glucuronate interconversions 1e-10  1  Homologous recombination 0.006  1  Fe2+ 0.001 0.000
   yihL   ecocyc   kegg   phen   152  yiiU  ecocyc   kegg  20.0 0.0  34  Inosine  C00294  7.324
  11  Starch and sucrose metabolism 8e-06  3  Phosphonate and phosphinate metabolism 0.002  7  Hypoxanthine 0.001 1.000
   yihM   ecocyc   kegg   phen   116  yifB  ecocyc   kegg  9.3 0.0  7  gamma-glutamyl-gamma aminobutyric acid  C15767  6.574
  4  Phosphotransferase system (PTS) 2e-06  2  Chlorocyclohexane and chlorobenzene degradation 0.000  6  Aerobactin 0.000 0.000
   yihN   ecocyc   kegg   phen   250  yifE  ecocyc   kegg  9.8 0.0  6  N-Acetyl-L-glutamate  C00624  4.470
  5  Novobiocin biosynthesis 0.002  3  Chlorocyclohexane and chlorobenzene degradation 0.000  8  Agmatine 0.000 0.000
   yihP   ecocyc   kegg   phen   112  tdcC  ecocyc   kegg  7.3 0.0  3  L-Arginine  C00062  -3.500
  2  Caprolactam degradation 0  2  Fructose and mannose metabolism 0.003  3  D-Fructose 1,6-bisphosphate 0.000 0.000
   yihR   ecocyc   kegg   phen   155  yidG  ecocyc   kegg  13.5 0.0  19  Thiamin monophosphate  C01081  4.750
  7  Biosynthesis of secondary metabolites 0.0001  4  Homologous recombination 0.001  2  Adenine 0.001 0.000
   yihT   ecocyc   kegg   phen   108  yiiG  ecocyc   kegg  9.8 0.0  10  5-Formamido-1-(5-phospho-D-ribosyl)imidazole-4-carboxamide  C04734  4.770
  3  Purine metabolism 3e-06  1  Homologous recombination 0.002  3  D-Galactose 0.000 0.000
   yihV   ecocyc   kegg   phen   150  yhiK  ecocyc   kegg  11.8 0.0  7  Thiosulfate  C00320  3.776
  1  Biosynthesis of secondary metabolites 0  4  Bisphenol degradation 0.002  4  Fe3+ 0.000 0.000
   yihW   ecocyc   kegg   phen   160  tufB  ecocyc   kegg  6.7 0.0  1  Glycolate  C00160  4.033
 &nbsp &nbsp &nbsp  4  Glycerolipid metabolism 0.001  17  Dodecanoly-phosphate (n-C12:0) 0.000 0.000
   yiiD   ecocyc   kegg   phen   64  narI  ecocyc   kegg  17.1 0.0  1  Propanal  C00479  5.403
 &nbsp &nbsp &nbsp  8  Chlorocyclohexane and chlorobenzene degradation 0.000  1  Fe2+ 0.006 0.000
   yiiE   ecocyc   kegg   phen   108  menD  ecocyc   kegg  6.5 0.0  1  Thiosulfate  C00320  5.036
 &nbsp &nbsp &nbsp  4  Ascorbate and aldarate metabolism 0.006  6  4-Aminobutanal 0.000 0.000
   yiiF   ecocyc   kegg   phen   506  mobB  ecocyc   kegg  14.2 0.0 &nbsp &nbsp &nbsp
 &nbsp &nbsp &nbsp  4  Flagellar assembly 0.004 &nbsp &nbsp &nbsp
   yiiG   ecocyc   kegg   phen   144  yihL  ecocyc   kegg  15.6 0.0  20  gamma-glutamyl-gamma aminobutyric acid  C15767  7.538
  7  Biosynthesis of secondary metabolites 1e-06  1  Phosphonate and phosphinate metabolism 0.001  5  Adenine 0.000 0.000
   yiiM   ecocyc   kegg   phen   396  mobB  ecocyc   kegg  12.6 0.0 &nbsp &nbsp &nbsp
 &nbsp &nbsp &nbsp  6  Ribosome 0.002 &nbsp &nbsp &nbsp
   yiiQ   ecocyc   kegg   phen   22  yeaO  ecocyc   kegg  29.7 0.0  16  N-Acetylneuraminate  C00270  10.224
  5  alpha-Linolenic acid metabolism 2e-08  4  Inositol phosphate metabolism 0.000  2  Glyceraldehyde 3-phosphate 0.000 0.000
   yiiR   ecocyc   kegg   phen   99  yiaV  ecocyc   kegg  5.9 0.0  2  D-Glycerate 2-phosphate  C00631  4.270
 &nbsp &nbsp &nbsp  4  Fructose and mannose metabolism 0.000  7  L-Xylulose 0.000 0.000
   yiiS   ecocyc   kegg   phen   142  yiiG  ecocyc   kegg  15.0 0.0  71  Thiamin monophosphate  C01081  7.438
  16  Ascorbate and aldarate metabolism 9e-08  2  Homologous recombination 0.001  3  Hypoxanthine 0.002 1.000
   yiiX   ecocyc   kegg   phen   144  yjbC  ecocyc   kegg  12.0 0.0  7  Inosine  C00294  5.045
  5  Biosynthesis of secondary metabolites 1e-06  3  alpha-Linolenic acid metabolism 0.004 &nbsp &nbsp &nbsp
   yijD   ecocyc   kegg   phen   104  yiiG  ecocyc   kegg  8.4 0.0  2  Inosine  C00294  3.675
  3  Fructose and mannose metabolism 0.0006 &nbsp &nbsp &nbsp  5  L-alanine-D-glutamate 0.000 0.000
   yijE   ecocyc   kegg   phen   109  cspG  ecocyc   kegg  11.2 0.0 &nbsp &nbsp &nbsp
 &nbsp &nbsp &nbsp  11  Galactose metabolism 0.000 &nbsp &nbsp &nbsp
   yijF   ecocyc   kegg   phen   73  yjhG  ecocyc   kegg  11.2 0.0  8  D-Glucosamine 1-phosphate  C06156  3.979
 &nbsp &nbsp &nbsp  4  Chlorocyclohexane and chlorobenzene degradation 0.000  1  Spermidine 0.000 0.000
   yijO   ecocyc   kegg   phen   86  creA  ecocyc   kegg  7.5 0.0 &nbsp &nbsp &nbsp
 &nbsp &nbsp &nbsp  8  Other glycan degradation 0.000 &nbsp &nbsp &nbsp
   yijP   ecocyc   kegg   phen   125  yiiU  ecocyc   kegg  12.3 0.0  25  N-Acetyl-L-glutamate  C00624  9.243
  6  Tryptophan metabolism 0.0009  6  Homologous recombination 0.000  5  Taurine 0.000 0.000
   yjaA   ecocyc   kegg   phen   176  ygaY  ecocyc   kegg  6.9 0.0 &nbsp &nbsp &nbsp
 &nbsp &nbsp &nbsp  2  Propanoate metabolism 0.006 &nbsp &nbsp &nbsp
   yjaB   ecocyc   kegg   phen   185  yjcH  ecocyc   kegg  13.9 0.0  17  Thiamin monophosphate  C01081  4.131
  5  Purine metabolism 3e-05  3  Chlorocyclohexane and chlorobenzene degradation 0.000  9  o-Succinylbenzoate 0.000 0.000
   yjaG   ecocyc   kegg   phen   144  yidG  ecocyc   kegg  10.6 0.0  33  Inosine  C00294  10.235
  7  Lysine degradation 5e-06  1  Lipoic acid metabolism 0.004  3  Putrescine 0.007 1.000
   yjaH   ecocyc   kegg   phen   86  yfjW  ecocyc   kegg  13.8 0.0  13  D-Glycero-D-manno-heptose 7-phosphate  C07836  4.365
  4  Biosynthesis of secondary metabolites 1e-08  7  Phosphonate and phosphinate metabolism 0.001  1  Succinic semialdehyde 0.000 0.000
   yjbB   ecocyc   kegg   phen   230  pioO  ecocyc   kegg  7.1 0.0  1  1,2-Diacyl-sn-glycerol (dihexadec-9-enoyl, n-C16:1)  C00641  -4.367
 &nbsp &nbsp &nbsp &nbsp &nbsp &nbsp  16  glucosyl-O-acetyl-rhamanosyl-N-acetylglucosamyl-undecaprenyl diphosphate 0.000 0.000
   yjbD   ecocyc   kegg   phen   137  yjbE  ecocyc   kegg  12.9 0.0  1  Inosine  C00294  -3.788
 &nbsp &nbsp &nbsp  1  Homologous recombination 0.004  2  ferroxamine 0.000 0.000
   yjbE   ecocyc   kegg   phen   138  yjbD  ecocyc   kegg  12.9 0.0  8  gamma-glutamyl-gamma aminobutyric acid  C15767  3.523
  3  Fructose and mannose metabolism 0.0006  2  Homologous recombination 0.000  4  Taurine 0.000 0.000
   yjbF   ecocyc   kegg   phen   145  yrhC  ecocyc   kegg  9.2 0.0  7  d-biotin d-sulfoxide  d-biotin d-sulfoxide  3.793
  15  Peptidoglycan biosynthesis 2e-05  4  Tyrosine metabolism 0.003  2  Fe3+ 0.001 0.000
   yjbG   ecocyc   kegg   phen   255  yjcF  ecocyc   kegg  17.0 0.0  22  E-3-carboxy-2-pentenedioate 6-methyl ester  C11514  4.152
  14  Biosynthesis of secondary metabolites 4e-06  3  Nicotinate and nicotinamide metabolism 0.001  34  N1-(5-Phospho-alpha-D-ribosyl)-5,6-dimethylbenzimidazole 0.000 0.000
   yjbH   ecocyc   kegg   phen   119  yjeK  ecocyc   kegg  9.1 0.0 &nbsp &nbsp &nbsp
 &nbsp &nbsp &nbsp  6  Chlorocyclohexane and chlorobenzene degradation 0.000 &nbsp &nbsp &nbsp
   yjbI   ecocyc   kegg   phen   108  yjbM  ecocyc   kegg  9.1 0.0  1  Inosine  C00294  4.774
 &nbsp &nbsp &nbsp  2  Novobiocin biosynthesis 0.000  4  3-(4-Hydroxyphenyl)pyruvate 0.000 0.000
   yjbL   ecocyc   kegg   phen   101  yjcF  ecocyc   kegg  17.2 0.0  14  D-Glycero-D-manno-heptose 7-phosphate  C07836  4.891
  6  Biosynthesis of secondary metabolites 9e-07 &nbsp &nbsp &nbsp  3  Taurine 0.000 0.000
   yjbM   ecocyc   kegg   phen   136  yjcF  ecocyc   kegg  14.1 0.0  22  D-Glycero-D-manno-heptose 7-phosphate  C07836  4.605
  7  Biosynthesis of secondary metabolites 0.0002  1  Base excision repair 0.010  3  Taurine 0.000 0.000
   yjbQ   ecocyc   kegg   phen   119  yjbL  ecocyc   kegg  10.9 0.0  10  Inosine  C00294  7.307
  2  Folate biosynthesis 1e-05  14  Geraniol degradation 0.000  28  3-Oxodecanoyl-CoA 0.000 0.000
   yjbR   ecocyc   kegg   phen   78  yjcC  ecocyc   kegg  6.6 0.0  5  D-Glycero-D-manno-heptose 7-phosphate  C07836  6.147
  2  Glycerophospholipid metabolism 4e-05  5  Mismatch repair 0.000  1  Choline 0.000 0.000
   yjcB   ecocyc   kegg   phen   165  rluA  ecocyc   kegg  9.8 0.0  4  dehydroglycine  C15809  5.102
  2  Taurine and hypotaurine metabolism 8e-06  3  Ribosome 0.000  9  Glycerol 2-phosphate 0.000 0.000
   yjcC   ecocyc   kegg   phen   107  glpD  ecocyc   kegg  8.8 0.0  31  D-Glycero-D-manno-heptose 7-phosphate  C07836  4.718
  12  Pentose and glucuronate interconversions 5e-14  8  Nitrotoluene degradation 0.000  6  Taurine 0.000 0.000
   yjcD   ecocyc   kegg   phen   125  yjcP  ecocyc   kegg  7.3 0.0  3  N2-Succinyl-L-ornithine  C03415  -3.476
 &nbsp &nbsp &nbsp  2  Novobiocin biosynthesis 0.006  8  Cadmium 0.000 0.000
   yjcE   ecocyc   kegg   phen   100  phnA  ecocyc   kegg  8.7 0.0 &nbsp &nbsp &nbsp
 &nbsp &nbsp &nbsp  2  Phosphonate and phosphinate metabolism 0.001 &nbsp &nbsp &nbsp
   yjcF   ecocyc   kegg   phen   136  yjbL  ecocyc   kegg  17.2 0.0  11  sn-Glycero-3-phosphoethanolamine  C01233  -4.625
  7  Biosynthesis of secondary metabolites 3e-05 &nbsp &nbsp &nbsp  1  GTP 0.002 0.000
   yjcH   ecocyc   kegg   phen   195  yjaB  ecocyc   kegg  13.9 0.0 &nbsp &nbsp &nbsp
 &nbsp &nbsp &nbsp  10  Chlorocyclohexane and chlorobenzene degradation 0.000 &nbsp &nbsp &nbsp
   yjcO   ecocyc   kegg   phen   210  yjcH  ecocyc   kegg  11.7 0.0  1  S-Methyl-L-methionine  C03172  3.549
 &nbsp &nbsp &nbsp  8  Chlorocyclohexane and chlorobenzene degradation 0.000  9  3-Carboxy-3-hydroxy-4-methylpentanoate 0.000 0.000
   yjcS   ecocyc   kegg   phen   200  frlR  ecocyc   kegg  12.4 0.0  5  Isopentenyl diphosphate  C00129  3.709
  1  Biosynthesis of secondary metabolites 0  12  Chlorocyclohexane and chlorobenzene degradation 0.000  21  L-Valine 0.009 1.000
   yjdA   ecocyc   kegg   phen   116  yjeN  ecocyc   kegg  10.1 0.0  7  sn-Glycero-3-phosphoethanolamine  C01233  -3.523
  2  Purine metabolism 0  3  Homologous recombination 0.002  6  Cadmium 0.000 0.000
   yjdC   ecocyc   kegg   phen   168  yphC  ecocyc   kegg  6.9 0.0 &nbsp &nbsp &nbsp
 &nbsp &nbsp &nbsp  3  Chlorocyclohexane and chlorobenzene degradation 0.000 &nbsp &nbsp &nbsp
   yjdF   ecocyc   kegg   phen   518  yjeK  ecocyc   kegg  17.8 0.0  10  (S)-Propane-1,2-diol  C02917  4.004
 &nbsp &nbsp &nbsp  8  Chlorocyclohexane and chlorobenzene degradation 0.000  37  (S)-3-Methyl-2-oxopentanoate 0.000 0.000
   yjdI   ecocyc   kegg   phen   139  yidA  ecocyc   kegg  8.8 0.0  16  D-Malate  C00497  6.080
  1  Biosynthesis of secondary metabolites 0  4  Sphingolipid metabolism 0.001  1  L-Aspartate 0.010 0.000
   yjdJ   ecocyc   kegg   phen   81  yjbG  ecocyc   kegg  8.9 0.0  1  2-Oxopent-4-enoate  C00596  3.948
 &nbsp &nbsp &nbsp  2  Biotin metabolism 0.004  4  2,3-dihydroxicinnamic acid 0.000 0.000
   yjdK   ecocyc   kegg   phen   119  yjeP  ecocyc   kegg  11.2 0.0  20  2-Dehydro-3-deoxy-D-gluconate 6-phosphate  C04442  3.845
  3  Histidine metabolism 0.0002  10  Geraniol degradation 0.001  17  3-Oxodecanoyl-CoA 0.000 0.000
   yjdL   ecocyc   kegg   phen   248  yidG  ecocyc   kegg  14.6 0.0  26  N-Acetyl-L-glutamate  C00624  4.069
  5  Lysine degradation 2e-08  10  Chlorocyclohexane and chlorobenzene degradation 0.000  19  Adenosine 3',5'-bisphosphate 0.000 0.000
   yjdO   ecocyc   kegg   phen   132  dipZ  ecocyc   kegg  11.1 0.0 &nbsp &nbsp &nbsp
  1  Arginine and proline metabolism 0  2  Bacterial secretion system 0.005 &nbsp &nbsp &nbsp
   yjeH   ecocyc   kegg   phen   299  yjeK  ecocyc   kegg  14.8 0.0  26  Isopentenyl diphosphate  C00129  3.714
  3  Purine metabolism 3e-05  7  Chlorocyclohexane and chlorobenzene degradation 0.000  23  (S)-3-Methyl-2-oxopentanoate 0.000 0.000
   yjeI   ecocyc   kegg   phen   229  yhiK  ecocyc   kegg  21.8 0.0  3  glycogen  C00182  4.217
  3  Microbial metabolism in diverse environments 0  4  Ribosome 0.001  15  2,5-diketo-D-gluconate 0.000 0.000
   yjeJ   ecocyc   kegg   phen   164  tauB  ecocyc   kegg  14.5 0.0  30  L-Lyxose  C01508  4.029
  5  Pyrimidine metabolism 3e-05  4  Phosphonate and phosphinate metabolism 0.002  3  cyclic pyranopterin monophosphate 0.000 0.000
   yjeM   ecocyc   kegg   phen   100  yeaO  ecocyc   kegg  20.8 0.0  32  N-Acetylneuraminate  C00270  22.504
  4  alpha-Linolenic acid metabolism 3e-09  4  Inositol phosphate metabolism 0.001  2  Glyceraldehyde 3-phosphate 0.000 0.000
   yjeN   ecocyc   kegg   phen   126  yjdA  ecocyc   kegg  10.1 0.0  16  Hexadecanoyl-phosphate (n-C16:1)  Hexadecanoyl-phosphate (n-C16:1)  6.499
  5  Glycerophospholipid metabolism 0.0001  4  Homologous recombination 0.000  3  Adenine 0.000 0.000
   yjeO   ecocyc   kegg   phen   466  yjeK  ecocyc   kegg  16.3 0.0  18  1,4-Dihydroxy-2-naphthoate  C03657  4.303
  2  Purine metabolism 3e-05  9  Chlorocyclohexane and chlorobenzene degradation 0.000  40  (S)-3-Methyl-2-oxopentanoate 0.000 0.000
   yjeP   ecocyc   kegg   phen   289  yjeK  ecocyc   kegg  12.8 0.0  8  dGMP  C00362  3.486
  2  Purine metabolism 0  10  Chlorocyclohexane and chlorobenzene degradation 0.000  28  5-amino-1-(5-phospho-D-ribosyl)imidazole-4-carboxylate 0.000 0.000
   yjeT   ecocyc   kegg   phen   136  yjfC  ecocyc   kegg  14.2 0.0  30  Hydroxypyruvate  C00168  3.793
  3  Purine metabolism 7e-07  5  Homologous recombination 0.000  6  Taurine 0.000 0.000
   yjfC   ecocyc   kegg   phen   275  yjfL  ecocyc   kegg  15.4 0.0  41  Guanosine  C00387  -3.636
  7  Lipopolysaccharide biosynthesis 2e-08  10  Ascorbate and aldarate metabolism 0.000  29  3-Dehydro-L-gulonate 0.000 0.000
   yjfI   ecocyc   kegg   phen   210  yjfK  ecocyc   kegg  12.2 0.0  8  Inosine  C00294  -3.689
  3  Glycerophospholipid metabolism 4e-05  10  Pantothenate and CoA biosynthesis 0.002  18  Aerobactin 0.000 0.000
   yjfL   ecocyc   kegg   phen   445  yjfC  ecocyc   kegg  15.4 0.0  39  Isopentenyl diphosphate  C00129  3.727
  7  Lipopolysaccharide biosynthesis 1e-08  7  Chlorocyclohexane and chlorobenzene degradation 0.000  32  3-Dehydro-L-gulonate 0.000 0.000
   yjfM   ecocyc   kegg   phen   178  yjfJ  ecocyc   kegg  13.1 0.0  42  Inosine  C00294  10.230
  7  Starch and sucrose metabolism 1e-05  5  Pantothenate and CoA biosynthesis 0.000  13  2-Oxobutanoate 0.003 1.000
   yjfN   ecocyc   kegg   phen   239  yjjM  ecocyc   kegg  10.1 0.0  1  &nbsp &nbsp
 &nbsp &nbsp &nbsp  5  Chlorocyclohexane and chlorobenzene degradation 0.000  8  D-Lactate 0.000 0.000
   yjfP   ecocyc   kegg   phen   296  modE  ecocyc   kegg  14.5 0.0  27  N1-(alpha-D-ribosyl)-5,6-dimethylbenzimidazole  C05775  6.207
  4  Oxidative phosphorylation 3e-05  9  Riboflavin metabolism 0.002  44  crotonobetaine 0.000 0.000
   yjfY   ecocyc   kegg   phen   103  yjeK  ecocyc   kegg  10.1 0.0  6  Cytosine  C00380  5.483
  1  Biosynthesis of secondary metabolites 0  14  Pantothenate and CoA biosynthesis 0.000  16  Aerobactin 0.000 0.000
   yjgA   ecocyc   kegg   phen   108  ydaV  ecocyc   kegg  13.7 0.0  5  N1-(5-Phospho-D-ribosyl)glycinamide  C03838  4.831
  6  Biosynthesis of secondary metabolites 0.0001  4  Nucleotide excision repair 0.001 &nbsp &nbsp &nbsp
   yjgB   ecocyc   kegg   phen   269  emtA  ecocyc   kegg  11.2 0.0 &nbsp &nbsp &nbsp
 &nbsp &nbsp &nbsp  3  Chlorocyclohexane and chlorobenzene degradation 0.000 &nbsp &nbsp &nbsp
   yjgH   ecocyc   kegg   phen   133  yjeK  ecocyc   kegg  8.3 0.0  11  Aminoacetone  C01888  4.078
  5  Pyruvate metabolism 0.0004  15  Chlorocyclohexane and chlorobenzene degradation 0.000  24  Adenosine 5'-phosphosulfate 0.000 0.000
   yjgI   ecocyc   kegg   phen   123  yjhQ  ecocyc   kegg  20.5 0.0  14  phosphatidylethanolamine (dihexadec-9enoyl, n-C16:1)  C00350  4.413
  6  Starch and sucrose metabolism 2e-09  9  Homologous recombination 0.000  6  Succinate 0.006 1.000
   yjgJ   ecocyc   kegg   phen   246  emrD  ecocyc   kegg  14.7 0.0  7  methanesulfonate  C11145  -5.184
  9  Nitrotoluene degradation 2e-05  3  Ribosome 0.001  24  Glycerol 2-phosphate 0.000 0.000
   yjgL   ecocyc   kegg   phen   71  yjjZ  ecocyc   kegg  7.5 0.0  25  octanoate (n-C8:0)  C06423  11.187
  2  Caprolactam degradation 0  2  Aminoacyl-tRNA biosynthesis 0.007  1  Dihydroxyacetone phosphate 0.002 1.000
   yjgM   ecocyc   kegg   phen   128  yjgJ  ecocyc   kegg  11.3 0.0  10  octanoate (n-C8:0)  C06423  10.865
  15  Chlorocyclohexane and chlorobenzene degradation 8e-08  1  Aminoacyl-tRNA biosynthesis 0.001  9  Citrate 0.000 0.000
   yjgN   ecocyc   kegg   phen   94  yeiG  ecocyc   kegg  6.7 0.0  1  Decanoate (n-C10:0)  C01571  6.809
 &nbsp &nbsp &nbsp  1  Sulfur relay system 0.009  7  dehydroglycine 0.000 0.000
   yjgR   ecocyc   kegg   phen   146  fldB  ecocyc   kegg  8.3 0.0 &nbsp &nbsp &nbsp
 &nbsp &nbsp &nbsp  5  Chlorocyclohexane and chlorobenzene degradation 0.000 &nbsp &nbsp &nbsp
   yjgW   ecocyc   kegg   phen   228  yddA  ecocyc   kegg  11.5 0.0  11  Thiosulfate  C00320  5.077
  16  Peptidoglycan biosynthesis 3e-05  4  Glycerolipid metabolism 0.003  11  Citrate 0.000 0.000
   yjgZ   ecocyc   kegg   phen   101  yecT  ecocyc   kegg  6.7 0.0  3  Uridine  C00299  3.764
  4  Biotin metabolism 5e-05  6  Sphingolipid metabolism 0.001  3  L-Threonine 0.000 0.000
   yjhB   ecocyc   kegg   phen   57  yjjZ  ecocyc   kegg  12.3 0.0  2  octanoate (n-C8:0)  C06423  8.007
  2  Caprolactam degradation 0  2  Histidine metabolism 0.007  1  L-Histidine 0.000 0.000
   yjhC   ecocyc   kegg   phen   196  yjeK  ecocyc   kegg  9.4 0.0  2  dIMP  C06196  -4.129
 &nbsp &nbsp &nbsp  12  Chlorocyclohexane and chlorobenzene degradation 0.000  16  (S)-3-Methyl-2-oxopentanoate 0.000 0.000
   yjhF   ecocyc   kegg   phen   123  yjiG  ecocyc   kegg  9.1 0.0  1  Bicarbonate  C00288  3.611
  1  Nitrogen metabolism 0  8  Chlorocyclohexane and chlorobenzene degradation 0.000  9  Aerobactin 0.000 0.000
   yjhG   ecocyc   kegg   phen   491  sbmC  ecocyc   kegg  19.6 0.0  31  5,6-Dimethylbenzimidazole  C03114  3.997
  7  Benzoate degradation 3e-08  11  Chlorocyclohexane and chlorobenzene degradation 0.000  41  (S)-Dihydroorotate 0.002 1.000
   yjhH   ecocyc   kegg   phen   89  yaeF  ecocyc   kegg  7.0 0.0  2  2-Oxoglutarate  C00026  -3.480
 &nbsp &nbsp &nbsp  2  Xylene degradation 0.005  1  Glycolate 0.001 0.000
   yjhP   ecocyc   kegg   phen   207  betT  ecocyc   kegg  10.5 0.0  6  3-Carboxy-3-hydroxy-4-methylpentanoate  C02504  3.696
  4  Aminobenzoate degradation 1e-06  8  Chlorocyclohexane and chlorobenzene degradation 0.000  17  Adenosine 5'-phosphosulfate 0.000 0.000
   yjhQ   ecocyc   kegg   phen   128  yjgI  ecocyc   kegg  20.5 0.0  26  (R)-Pantothenate  C00864  4.380
  9  Pantothenate and CoA biosynthesis 3e-06  8  Fatty acid biosynthesis 0.000  17  Acetyl-ACP 0.000 0.000
   yjhR   ecocyc   kegg   phen   113  yjhG  ecocyc   kegg  6.9 0.0 &nbsp &nbsp &nbsp
  5  Aminobenzoate degradation 8e-08  2  Chlorocyclohexane and chlorobenzene degradation 0.000 &nbsp &nbsp &nbsp
   yjhU   ecocyc   kegg   phen   162  rarD  ecocyc   kegg  8.4 0.0 &nbsp &nbsp &nbsp
 &nbsp &nbsp &nbsp  5  Glycerolipid metabolism 0.001 &nbsp &nbsp &nbsp
   yjhV   ecocyc   kegg   phen   127  tolB  ecocyc   kegg  6.8 0.0  12  N-Methyltryptophan  C02983  4.262
  8  Oxidative phosphorylation 0.0003  1  Phosphonate and phosphinate metabolism 0.001  10  L-Arabinose 0.000 0.000
   yjhX   ecocyc   kegg   phen   211  fhuC  ecocyc   kegg  12.3 0.0  11  Inosine  C00294  7.589
  12  Microbial metabolism in diverse environments 1e-07  10  Pantothenate and CoA biosynthesis 0.000  18  L-Phenylalanine 0.002 1.000
   yjiA   ecocyc   kegg   phen   252  mcrC  ecocyc   kegg  9.3 0.0 &nbsp &nbsp &nbsp
 &nbsp &nbsp &nbsp  3  Chlorocyclohexane and chlorobenzene degradation 0.000 &nbsp &nbsp &nbsp
   yjiC   ecocyc   kegg   phen   114  recT  ecocyc   kegg  9.8 0.0 &nbsp &nbsp &nbsp
 &nbsp &nbsp &nbsp  10  DNA replication 0.000 &nbsp &nbsp &nbsp
   yjiG   ecocyc   kegg   phen   63  yjjW  ecocyc   kegg  17.9 0.0  1  Hexadecenoate (n-C16:1)  C08362  4.653
  1  Biosynthesis of secondary metabolites 0  8  DNA replication 0.000  2  sn-Glycero-3-phosphoethanolamine 0.008 0.000
   yjiH   ecocyc   kegg   phen   102  yjeK  ecocyc   kegg  7.4 0.0  5  octanoate (n-C8:0)  C06423  9.250
  2  Caprolactam degradation 0  13  Cysteine and methionine metabolism 0.000  13  4-Phospho-L-aspartate 0.000 0.000
   yjiJ   ecocyc   kegg   phen   202  phnL  ecocyc   kegg  7.4 0.0 &nbsp &nbsp &nbsp
 &nbsp &nbsp &nbsp  1  Fructose and mannose metabolism 0.002 &nbsp &nbsp &nbsp
   yjiK   ecocyc   kegg   phen   359  yidR  ecocyc   kegg  9.2 0.0  4  D-Glycerate 2-phosphate  C00631  3.629
  8  Glycolysis / Gluconeogenesis 0  9  Chlorocyclohexane and chlorobenzene degradation 0.000  20  3-Carboxy-2-hydroxy-4-methylpentanoate 0.000 0.000
   yjiL   ecocyc   kegg   phen   89  yjgK  ecocyc   kegg  13.0 0.0  5  octanoate (n-C8:0)  C06423  6.274
  3  Chloroalkane and chloroalkene degradation 4e-08  2  Chloroalkane and chloroalkene degradation 0.002  2  D-Galactose 0.000 0.000
   yjiM   ecocyc   kegg   phen   77  yacG  ecocyc   kegg  5.6 0.0  3  6-phospho-D-glucono-1,5-lactone  C01236  3.616
 &nbsp &nbsp &nbsp  2  D-Glutamine and D-glutamate metabolism 0.000  6  2-Dehydro-3-deoxy-D-gluconate 0.000 0.000
   yjiN   ecocyc   kegg   phen   51  yjjW  ecocyc   kegg  20.4 0.0  3  Hexadecenoate (n-C16:1)  C08362  6.770
  5  Fatty acid metabolism 1e-05  8  D-Alanine metabolism 0.001  4  Choline 0.000 0.000
   yjiQ   ecocyc   kegg   phen   152  paaK  ecocyc   kegg  8.2 0.0  1  Nitrate  C00244  -3.883
  5  Biosynthesis of unsaturated fatty acids 5e-06  3  Bisphenol degradation 0.002  4  Dihydroxyacetone 0.000 0.000
   yjiR   ecocyc   kegg   phen   111  sbmC  ecocyc   kegg  12.7 0.0 &nbsp &nbsp &nbsp
 &nbsp &nbsp &nbsp  5  Chlorocyclohexane and chlorobenzene degradation 0.000 &nbsp &nbsp &nbsp
   yjiS   ecocyc   kegg   phen   140  umuC  ecocyc   kegg  8.5 0.0  7  Thiamin monophosphate  C01081  4.107
  2  Purine metabolism 0  3  Homologous recombination 0.000  10  dADP 0.001 0.000
   yjiT   ecocyc   kegg   phen   237  yrhA  ecocyc   kegg  8.2 0.0 &nbsp &nbsp &nbsp
 &nbsp &nbsp &nbsp  4  Chlorocyclohexane and chlorobenzene degradation 0.000 &nbsp &nbsp &nbsp
   yjiX   ecocyc   kegg   phen   138  ydhL  ecocyc   kegg  9.2 0.0  21  Guanine  C00242  6.587
  7  C5-Branched dibasic acid metabolism 0  7  Biosynthesis of siderophore group nonribosomal peptides 0.000  8  2,3-Dihydro-2,3-dihydroxybenzoate 0.000 0.000
   yjiY   ecocyc   kegg   phen   174  dgoD  ecocyc   kegg  10.5 0.0  2  octanoate (n-C8:0)  C06423  5.019
  2  Caprolactam degradation 0 &nbsp &nbsp &nbsp  14  Glycerol 2-phosphate 0.000 0.000
   yjjA   ecocyc   kegg   phen   193  gntR  ecocyc   kegg  7.6 0.0  15  Oxalureate  C00802  4.298
  14  Benzoate degradation 7e-10  2  Ribosome 0.000  11  Glycerol 2-phosphate 0.000 1.000
   yjjB   ecocyc   kegg   phen   220  clcB  ecocyc   kegg  12.2 0.0  1  Deoxyuridine  C00526  -3.677
 &nbsp &nbsp &nbsp  1  ABC transporters 0.008  10  Acetate 0.000 0.000
   yjjI   ecocyc   kegg   phen   93  yifB  ecocyc   kegg  8.9 0.0  6  Thiamin monophosphate  C01081  4.578
  5  Purine metabolism 3e-05  4  Homologous recombination 0.000  9  Aerobactin 0.000 0.000
   yjjJ   ecocyc   kegg   phen   106  holD  ecocyc   kegg  7.2 0.0 &nbsp &nbsp &nbsp
 &nbsp &nbsp &nbsp  12  Chlorocyclohexane and chlorobenzene degradation 0.000 &nbsp &nbsp &nbsp
   yjjP   ecocyc   kegg   phen   84  wzc  ecocyc   kegg  6.7 0.0 &nbsp &nbsp &nbsp
 &nbsp &nbsp &nbsp  5  Galactose metabolism 0.001 &nbsp &nbsp &nbsp
   yjjQ   ecocyc   kegg   phen   154  nudD  ecocyc   kegg  7.3 0.0 &nbsp &nbsp &nbsp
 &nbsp &nbsp &nbsp  1  Fructose and mannose metabolism 0.003 &nbsp &nbsp &nbsp
   yjjU   ecocyc   kegg   phen   160  dcm  ecocyc   kegg  10.6 0.0  3  Cytosine  C00380  3.785
  3  Fructose and mannose metabolism 9e-05  3  Homologous recombination 0.000  11  Aerobactin 0.000 0.000
   yjjV   ecocyc   kegg   phen   136  aspA  ecocyc   kegg  11.4 0.0 &nbsp &nbsp &nbsp
 &nbsp &nbsp &nbsp  2  Ascorbate and aldarate metabolism 0.001 &nbsp &nbsp &nbsp
   yjjW   ecocyc   kegg   phen   50  yjiN  ecocyc   kegg  20.4 0.0  2  octadecanoate (n-C18:0)  C01530  4.390
  2  Biosynthesis of secondary metabolites 0.0002  6  Homologous recombination 0.000  2  sn-Glycero-3-phosphoethanolamine 0.006 0.000
   yjjY   ecocyc   kegg   phen   155  arcA  ecocyc   kegg  15.4 0.0  55  Thiamin monophosphate  C01081  8.555
  11  Pantothenate and CoA biosynthesis 0.0007  8  Homologous recombination 0.000  13  Aerobactin 0.000 0.000
   yjjZ   ecocyc   kegg   phen   72  yjhB  ecocyc   kegg  12.3 0.0  9  octanoate (n-C8:0)  C06423  7.484
  16  Caprolactam degradation 8e-07  2  Bisphenol degradation 0.000  1  L-Serine 0.002 0.000
   ykfB   ecocyc   kegg   phen   128  yafZ  ecocyc   kegg  13.0 0.0  2  Hexadecenoate (n-C16:1)  C08362  5.031
  1  Phosphonate and phosphinate metabolism 0  3  Pantothenate and CoA biosynthesis 0.002  3  2-Oxobutanoate 0.000 0.000
   ykfC   ecocyc   kegg   phen   205  leuB  ecocyc   kegg  7.1 0.0 &nbsp &nbsp &nbsp
 &nbsp &nbsp &nbsp  2  Chlorocyclohexane and chlorobenzene degradation 0.000 &nbsp &nbsp &nbsp
   ykfF   ecocyc   kegg   phen   149  yjjV  ecocyc   kegg  9.5 0.0  1  Ethanol  C00469  3.666
 &nbsp &nbsp &nbsp  10  Bacterial secretion system 0.000  10  Undecaprenyl-diphospho N-acetylglucosamine-N-acetylmannosaminuronate-N-acetamido-4,6-dideoxy-D-galactose 0.000 0.000
   ykfG   ecocyc   kegg   phen   100  yfbS  ecocyc   kegg  9.7 0.0  2  FMN  C00061  4.178
 &nbsp &nbsp &nbsp  1  Biosynthesis of unsaturated fatty acids 0.006  1  L-Methionine 0.004 0.000
   ykfH   ecocyc   kegg   phen   92  ycjF  ecocyc   kegg  6.0 0.0  2  Cobinamide  C05774  4.252
  1  Porphyrin and chlorophyll metabolism 0  3  Other glycan degradation 0.002  1  Sodium 0.001 0.000
   ykfJ   ecocyc   kegg   phen   86  ycjX  ecocyc   kegg  7.4 0.0  1  Guanine  C00242  3.885
  1  Phosphonate and phosphinate metabolism 0 &nbsp &nbsp &nbsp  2  L-tartrate 0.000 0.000
   ykgA   ecocyc   kegg   phen   148  ymdE  ecocyc   kegg  10.4 0.0  1  sulfur dioxide  C09306  5.287
  1  Glycerophospholipid metabolism 0  3  Ascorbate and aldarate metabolism 0.002  20  2-Dehydro-3-deoxy-D-gluconate 0.000 0.000
   ykgB   ecocyc   kegg   phen   194  bolA  ecocyc   kegg  7.5 0.0 &nbsp &nbsp &nbsp
 &nbsp &nbsp &nbsp  3  Chlorocyclohexane and chlorobenzene degradation 0.000 &nbsp &nbsp &nbsp
   ykgC   ecocyc   kegg   phen   192  yegX  ecocyc   kegg  8.6 0.0  25  6-phospho-D-glucono-1,5-lactone  C01236  4.293
  4  Limonene and pinene degradation 1e-09  1  Phosphonate and phosphinate metabolism 0.003  15  (R)-Pantothenate 0.000 0.000
   ykgD   ecocyc   kegg   phen   99  dinB  ecocyc   kegg  15.0 0.0  4  Adenosine  C00212  4.501
  2  Phosphonate and phosphinate metabolism 5e-05  10  Novobiocin biosynthesis 0.000  6  2,3-dehydroadipyl-CoA 0.000 0.000
   ykgE   ecocyc   kegg   phen   211  yphC  ecocyc   kegg  7.7 0.0 &nbsp &nbsp &nbsp
 &nbsp &nbsp &nbsp  4  Chlorocyclohexane and chlorobenzene degradation 0.000 &nbsp &nbsp &nbsp
   ykgG   ecocyc   kegg   phen   111  fliO  ecocyc   kegg  9.9 0.0 &nbsp &nbsp &nbsp
  8  Microbial metabolism in diverse environments 1e-11  4  Caprolactam degradation 0.005 &nbsp &nbsp &nbsp
   ykgH   ecocyc   kegg   phen   105  frsA  ecocyc   kegg  7.8 0.0  3  Adenosine  C00212  3.691
  1  Biosynthesis of secondary metabolites 0  5  Other glycan degradation 0.002  4  chorismate 0.000 0.000
   ykgI   ecocyc   kegg   phen   127  ushA  ecocyc   kegg  9.8 0.0  1  Tetradecanoyl-phosphate (n-C14:0)  Tetradecanoyl-phosphate (n-C14:0)  3.517
  2  Biosynthesis of secondary metabolites 2e-08  7  Bacterial secretion system 0.000  6  3-keto-L-gulonate-6-phosphate 0.000 0.000
   ykgJ   ecocyc   kegg   phen   96  gnd  ecocyc   kegg  10.8 0.0  7  Dihydropteroate  C00921  5.456
  4  Lysine degradation 3e-08  11  D-Glutamine and D-glutamate metabolism 0.001  9  ADP-L-glycero-D-manno-heptose 0.000 0.000
   ykgL   ecocyc   kegg   phen   158  yhiK  ecocyc   kegg  13.6 0.0  1  Thiosulfate  C00320  3.770
  1  Arginine and proline metabolism 0  1  Folate biosynthesis 0.009  8  2-Dehydro-3-deoxy-D-gluconate 0.000 0.000
   ykgM   ecocyc   kegg   phen   124  yghY  ecocyc   kegg  6.4 0.0 &nbsp &nbsp &nbsp
 &nbsp &nbsp &nbsp  2  Bisphenol degradation 0.001 &nbsp &nbsp &nbsp
   ykgN   ecocyc   kegg   phen   145  yehC  ecocyc   kegg  14.7 0.0  5  D-Glucosamine 6-phosphate  C00352  -3.459
 &nbsp &nbsp &nbsp  2  Aminoacyl-tRNA biosynthesis 0.000  15  L-Lysine 0.000 0.000
   ykiA   ecocyc   kegg   phen   120  mppA  ecocyc   kegg  7.2 0.0 &nbsp &nbsp &nbsp
  4  Glycerolipid metabolism 0.0002  1  Peptidoglycan biosynthesis 0.004 &nbsp &nbsp &nbsp
   ykiB   ecocyc   kegg   phen   79  yfbE  ecocyc   kegg  6.0 0.0 &nbsp &nbsp &nbsp
  1  Glycerophospholipid metabolism 0  1  Phosphonate and phosphinate metabolism 0.000 &nbsp &nbsp &nbsp
   ylaC   ecocyc   kegg   phen   105  ycgG  ecocyc   kegg  9.8 0.0 &nbsp &nbsp &nbsp
 &nbsp &nbsp &nbsp  3  Phosphonate and phosphinate metabolism 0.001 &nbsp &nbsp &nbsp
   ylbH   ecocyc   kegg   phen   440  mobB  ecocyc   kegg  13.0 0.0 &nbsp &nbsp &nbsp
 &nbsp &nbsp &nbsp  5  Valine, leucine and isoleucine biosynthesis 0.003 &nbsp &nbsp &nbsp
   ylcE   ecocyc   kegg   phen   77  yjaB  ecocyc   kegg  7.6 0.0 &nbsp &nbsp &nbsp
 &nbsp &nbsp &nbsp  4  Novobiocin biosynthesis 0.000 &nbsp &nbsp &nbsp
   ylcG   ecocyc   kegg   phen   348  yphB  ecocyc   kegg  17.2 0.0  5  D-tartrate  C02107  4.431
  10  Aminobenzoate degradation 8e-07  1  Aminoacyl-tRNA biosynthesis 0.002  20  glucosyl-O-acetyl-rhamanosyl-N-acetylglucosamyl-undecaprenyl diphosphate 0.000 0.000
   yliE   ecocyc   kegg   phen   106  sdaA  ecocyc   kegg  12.5 0.0  2  gamma-glutamyl-gamma-butyraldehyde  C15700  3.771
  1  Biosynthesis of secondary metabolites 0  1  DNA replication 0.002  2  L-Tyrosine 0.000 0.000
   yliF   ecocyc   kegg   phen   97  pphB  ecocyc   kegg  8.5 0.0  2  N-(5-Phospho-D-ribosyl)anthranilate  C04302  -3.514
 &nbsp &nbsp &nbsp  2  ABC transporters 0.001  8  D-Allose 0.000 0.000
   yliI   ecocyc   kegg   phen   100  ycdI  ecocyc   kegg  12.9 0.0  22  2-Octaprenyl-6-methoxyphenol  C05812  4.307
  12  Arginine and proline metabolism 2e-06  7  Chlorocyclohexane and chlorobenzene degradation 0.000  10  Pyridoxine 5'-phosphate 0.000 0.000
   ymbA   ecocyc   kegg   phen   107  ogt  ecocyc   kegg  10.6 0.0 &nbsp &nbsp &nbsp
  1  Biosynthesis of secondary metabolites 0.004  3  Phosphonate and phosphinate metabolism 0.001 &nbsp &nbsp &nbsp
   ymdC   ecocyc   kegg   phen   137  fepA  ecocyc   kegg  8.8 0.0 &nbsp &nbsp &nbsp
 &nbsp &nbsp &nbsp  2  Bisphenol degradation 0.001 &nbsp &nbsp &nbsp
   ymdF   ecocyc   kegg   phen   366  kdpE  ecocyc   kegg  10.4 0.0  17  Butanal  C01412  3.655
  10  Lipoic acid metabolism 0  2  Bisphenol degradation 0.009  20  glucosyl-O-acetyl-rhamanosyl-N-acetylglucosamyl-undecaprenyl diphosphate 0.000 0.000
   ymfA   ecocyc   kegg   phen   186  ycfQ  ecocyc   kegg  11.4 0.0 &nbsp &nbsp &nbsp
 &nbsp &nbsp &nbsp  4  Aminobenzoate degradation 0.001 &nbsp &nbsp &nbsp
   ymfD   ecocyc   kegg   phen   161  ybcK  ecocyc   kegg  11.7 0.0  20  trans-Aconitate  C02341  4.193
  15  C5-Branched dibasic acid metabolism 2e-15  4  Biosynthesis of siderophore group nonribosomal peptides 0.000  17  2,3-Dihydro-2,3-dihydroxybenzoate 0.000 0.000
   ymfE   ecocyc   kegg   phen   230  yccX  ecocyc   kegg  8.4 0.0  2  N2-Formyl-N1-(5-phospho-D-ribosyl)glycinamide  C04376  3.883
 &nbsp &nbsp &nbsp  2  Phosphonate and phosphinate metabolism 0.004  16  Dihydroxyacetone 0.000 0.000
   ymfH   ecocyc   kegg   phen   63  fecA  ecocyc   kegg  7.8 0.0 &nbsp &nbsp &nbsp
 &nbsp &nbsp &nbsp  3  Phenylalanine, tyrosine and tryptophan biosynthesis 0.005 &nbsp &nbsp &nbsp
   ymfI   ecocyc   kegg   phen   92  ybhJ  ecocyc   kegg  12.3 0.0  2  tetradecenoate (n-C14:1)  tetradecenoate (n-C14:1)  4.401
  3  Ubiquinone and other terpenoid-quinone biosynthesis 0.0003  4  Glycolysis / Gluconeogenesis 0.000  4  D-Fructose 1,6-bisphosphate 0.000 0.000
   ymfJ   ecocyc   kegg   phen   93  fldB  ecocyc   kegg  7.8 0.0  1  7-cyano-7-carbaguanine  C15996  3.891
 &nbsp &nbsp &nbsp  5  Valine, leucine and isoleucine biosynthesis 0.001  2  L-Threonine 0.000 0.000
   ymfL   ecocyc   kegg   phen   111  uhpA  ecocyc   kegg  13.2 0.0  1  phosphatidylethanolamine (dihexadec-9enoyl, n-C16:1)  C00350  3.860
  3  Histidine metabolism 2e-06  3  Two-component system 0.000  8  5-Phospho-beta-D-ribosylamine 0.000 0.000
   ymfM   ecocyc   kegg   phen   79  yegX  ecocyc   kegg  6.6 0.0 &nbsp &nbsp &nbsp
 &nbsp &nbsp &nbsp  1  Nucleotide excision repair 0.009 &nbsp &nbsp &nbsp
   ymfN   ecocyc   kegg   phen   221  yncG  ecocyc   kegg  6.4 0.0 &nbsp &nbsp &nbsp
 &nbsp &nbsp &nbsp &nbsp &nbsp &nbsp &nbsp &nbsp &nbsp
   ymfO   ecocyc   kegg   phen   118  rdgC  ecocyc   kegg  9.9 0.0  50  4-Aminobenzoate  C00568  6.528
  13  Lysine degradation 5e-06 &nbsp &nbsp &nbsp  5  Undecaprenyl phosphate 0.000 0.000
   ymfP   ecocyc   kegg   phen   188  nuoC  ecocyc   kegg  7.2 0.0 &nbsp &nbsp &nbsp
 &nbsp &nbsp &nbsp  6  Chlorocyclohexane and chlorobenzene degradation 0.000 &nbsp &nbsp &nbsp
   ymfQ   ecocyc   kegg   phen   142  ymgC  ecocyc   kegg  12.7 0.0  10  Phosphatidylglycerophosphate (didodecanoyl, n-C12:0)  C03892  3.958
  10  Inositol phosphate metabolism 3e-06  2  Two-component system 0.002  10  D-Glycerate 2-phosphate 0.000 0.000
   ymfR   ecocyc   kegg   phen   103  ymdB  ecocyc   kegg  6.0 0.0  16  D-Tagatose 1,6-biphosphate  C03785  6.168
  11  Toluene degradation 9e-08  4  Glycolysis / Gluconeogenesis 0.000  3  D-Glucose 6-phosphate 0.000 0.000
   ymgA   ecocyc   kegg   phen   90  holD  ecocyc   kegg  9.3 0.0  5  2-Dehydro-3-deoxy-D-gluconate 6-phosphate  C04442  3.770
  7  Inositol phosphate metabolism 2e-07  15  Chlorocyclohexane and chlorobenzene degradation 0.000  9  5-Methyltetrahydrofolate 0.000 0.000
   ymgC   ecocyc   kegg   phen   86  ymfQ  ecocyc   kegg  12.7 0.0  1  Phenylpropanoate  C05629  4.449
  1  Biosynthesis of secondary metabolites 0  4  Oxidative phosphorylation 0.001  7  D-Glycerate 2-phosphate 0.000 0.000
   ymgD   ecocyc   kegg   phen   71  cusS  ecocyc   kegg  16.6 0.0  3  4-Phospho-L-aspartate  C03082  3.953
  4  Biosynthesis of secondary metabolites 0.001  7  Phosphonate and phosphinate metabolism 0.000  3  5-Phospho-alpha-D-ribose 1-diphosphate 0.001 0.000
   ymgE   ecocyc   kegg   phen   72  uxuA  ecocyc   kegg  16.6 0.0  26  Dethiobiotin  C01909  4.723
  12  Purine metabolism 3e-08  15  Biosynthesis of secondary metabolites 0.000  31  2-(Formamido)-N1-(5-phospho-D-ribosyl)acetamidine 0.000 0.000
   ymgF   ecocyc   kegg   phen   49  yddK  ecocyc   kegg  6.1 0.0  3  Urea  C00086  4.231
 &nbsp &nbsp &nbsp  3  Vitamin B6 metabolism 0.005  2  2-Demethylmenaquinone 8 0.006 0.000
   ymgG   ecocyc   kegg   phen   141  nmpC  ecocyc   kegg  7.9 0.0  5  L-Tryptophan  C00078  3.574
  19  Peptidoglycan biosynthesis 3e-05  2  Phosphonate and phosphinate metabolism 0.001  2  Dihydroxyacetone 0.000 0.000
   ymgH   ecocyc   kegg   phen   212  yobA  ecocyc   kegg  9.3 0.0 &nbsp &nbsp &nbsp
 &nbsp &nbsp &nbsp  3  Riboflavin metabolism 0.001 &nbsp &nbsp &nbsp
   ymjA   ecocyc   kegg   phen   194  fiu  ecocyc   kegg  14.1 0.0  48  gamma-glutamyl-putrescine  C15699  5.103
  15  D-Glutamine and D-glutamate metabolism 4e-06  3  Oxidative phosphorylation 0.000  13  alpha-D-Ribose 5-phosphate 0.003 1.000
   ymjB   ecocyc   kegg   phen   81  ydhX  ecocyc   kegg  6.9 0.0  37  d-biotin d-sulfoxide  d-biotin d-sulfoxide  3.947
  9  Limonene and pinene degradation 5e-08  2  Bisphenol degradation 0.000  4  Dimethyl sulfide 0.000 0.000
   ymjC   ecocyc   kegg   phen   112  fiu  ecocyc   kegg  11.6 0.0 &nbsp &nbsp &nbsp
  1  Biosynthesis of secondary metabolites 0  3  Toluene degradation 0.002 &nbsp &nbsp &nbsp
   ynaA   ecocyc   kegg   phen   56  yedF  ecocyc   kegg  14.4 0.0  14  D-Lactaldehyde  C00937  7.585
  39  D-Alanine metabolism 2e-05  1  Protein export 0.004 &nbsp &nbsp &nbsp
   ynaE   ecocyc   kegg   phen   163  ltaE  ecocyc   kegg  9.9 0.0  1  L-Lysine  C00047  3.670
 &nbsp &nbsp &nbsp  21  Biosynthesis of siderophore group nonribosomal peptides 0.000  18  2,3-Dihydro-2,3-dihydroxybenzoate 0.000 0.000
   ynaI   ecocyc   kegg   phen   128  ybjL  ecocyc   kegg  11.1 0.0 &nbsp &nbsp &nbsp
 &nbsp &nbsp &nbsp  4  Cyanoamino acid metabolism 0.001 &nbsp &nbsp &nbsp
   ynaJ   ecocyc   kegg   phen   68  ydcD  ecocyc   kegg  19.4 0.0  16  tetradecanoate (n-C14:0)  C06424  11.750
  3  Fatty acid biosynthesis 5e-10  2  Two-component system 0.000  4  L-Serine 0.001 0.000
   ynaK   ecocyc   kegg   phen   89  ygaR  ecocyc   kegg  8.4 0.0 &nbsp &nbsp &nbsp
 &nbsp &nbsp &nbsp &nbsp &nbsp &nbsp &nbsp &nbsp &nbsp
   ynbA   ecocyc   kegg   phen   193  ytjC  ecocyc   kegg  13.4 0.0 &nbsp &nbsp &nbsp
 &nbsp &nbsp &nbsp  7  Oxidative phosphorylation 0.001 &nbsp &nbsp &nbsp
   ynbB   ecocyc   kegg   phen   142  ybgF  ecocyc   kegg  16.8 0.0  69  N-Methyltryptophan  C02983  6.650
  15  Biosynthesis of secondary metabolites 2e-05  8  Ubiquinone and other terpenoid-quinone biosynthesis 0.001  7  2-Octaprenyl-6-methoxy-1,4-benzoquinol 0.000 0.000
   ynbC   ecocyc   kegg   phen   266  crcA  ecocyc   kegg  7.7 0.0  1  5-Phospho-beta-D-ribosylamine  C03090  3.772
 &nbsp &nbsp &nbsp &nbsp &nbsp &nbsp  18  L-fuculose 0.000 0.000
   ynbD   ecocyc   kegg   phen   77  yedR  ecocyc   kegg  8.4 0.0 &nbsp &nbsp &nbsp
 &nbsp &nbsp &nbsp  1  Novobiocin biosynthesis 0.002 &nbsp &nbsp &nbsp
   ynbE   ecocyc   kegg   phen   243  yiaU  ecocyc   kegg  10.0 0.0  13  N-Methyltryptophan  C02983  3.484
  5  Microbial metabolism in diverse environments 0  6  Pantothenate and CoA biosynthesis 0.000  14  D-Tagatose 1,6-biphosphate 0.000 1.000
   yncA   ecocyc   kegg   phen   121  dhaK  ecocyc   kegg  7.1 0.0 &nbsp &nbsp &nbsp
 &nbsp &nbsp &nbsp  5  Bisphenol degradation 0.001 &nbsp &nbsp &nbsp
   yncD   ecocyc   kegg   phen   85  ycjP  ecocyc   kegg  13.6 0.0  8  5-Methylthioadenosine  C00170  4.015
  5  Novobiocin biosynthesis 4e-05  5  Phosphonate and phosphinate metabolism 0.000  4  Pyridoxal 0.000 0.000
   yncE   ecocyc   kegg   phen   101  ydgD  ecocyc   kegg  9.7 0.0  10  Urea  C00086  3.644
  5  Fructose and mannose metabolism 3e-05  1  Taurine and hypotaurine metabolism 0.010  1  Arbutin 6-phosphate 0.000 0.000
   yncG   ecocyc   kegg   phen   214  yfjW  ecocyc   kegg  8.5 0.0  4  5,10-Methenyltetrahydrofolate  C00445  3.646
  1  Lysine biosynthesis 0.0009  2  Fructose and mannose metabolism 0.003  8  L-fuculose 0.000 0.000
   yncH   ecocyc   kegg   phen   258  ydbJ  ecocyc   kegg  7.9 0.0 &nbsp &nbsp &nbsp
 &nbsp &nbsp &nbsp  3  Chlorocyclohexane and chlorobenzene degradation 0.000 &nbsp &nbsp &nbsp
   yncI   ecocyc   kegg   phen   118  yecH  ecocyc   kegg  16.8 0.0  11  cyclopropane phosphatidylglycerol (dihexadec-9,10-cyclo-anoyl, n-C16:0 cyclo)  cyclopropane phosphatidylglycerol (dihexadec-9,10-cyclo-anoyl, n-C16:0 cyclo)  3.828
  2  Biotin metabolism 2e-06  9  RNA polymerase 0.000  2  Succinate 0.001 0.000
   yncJ   ecocyc   kegg   phen   163  ymcB  ecocyc   kegg  9.2 0.0  14  D-Glucosamine 1-phosphate  C06156  3.499
 &nbsp &nbsp &nbsp  4  Oxidative phosphorylation 0.000  11  D-Glycerate 2-phosphate 0.000 0.000
   yncM   ecocyc   kegg   phen   115  gst  ecocyc   kegg  22.8 0.0  118  1-dodecanoyl-sn-glycerol 3-phosphate  C00681  16.705
  15  Aminoacyl-tRNA biosynthesis 5e-12  6  Alanine, aspartate and glutamate metabolism 0.000  14  L-Glutamine 0.008 1.000
   yneE   ecocyc   kegg   phen   153  yoaB  ecocyc   kegg  8.2 0.0  13  Glycolaldehyde  C00266  4.529
  19  Glycerolipid metabolism 3e-05  2  Sphingolipid metabolism 0.002  3  D-Fructose 1,6-bisphosphate 0.000 0.000
   yneF   ecocyc   kegg   phen   268  cld  ecocyc   kegg  11.7 0.0 &nbsp &nbsp &nbsp
 &nbsp &nbsp &nbsp  4  Chlorocyclohexane and chlorobenzene degradation 0.000 &nbsp &nbsp &nbsp
   yneG   ecocyc   kegg   phen   241  yddL  ecocyc   kegg  10.9 0.0  65  ITP  C00081  4.987
  21  Glycine, serine and threonine metabolism 1e-06  7  Chlorocyclohexane and chlorobenzene degradation 0.000  28  2-Oxobutanoate 0.002 1.000
   yneJ   ecocyc   kegg   phen   169  ushA  ecocyc   kegg  9.8 0.0  14  N-Acetyl-D-glucosamine  C00140  4.947
  5  Lysine degradation 4e-06  11  Lysine biosynthesis 0.001  18  Aerobactin 0.000 0.000
   yneL   ecocyc   kegg   phen   78  tiaE  ecocyc   kegg  21.4 0.0  7  methanesulfonate  C11145  4.067
  2  Histidine metabolism 5e-06  13  Biosynthesis of siderophore group nonribosomal peptides 0.000  9  2,3-Dihydro-2,3-dihydroxybenzoate 0.000 0.000
   ynfA   ecocyc   kegg   phen   73  yffB  ecocyc   kegg  10.1 0.0  2  Urate  C00366  3.525
  10  Microbial metabolism in diverse environments 6e-10  2  Ribosome 0.002  2  Nitrate 0.000 0.000
   ynfB   ecocyc   kegg   phen   65  ydfC  ecocyc   kegg  10.4 0.0  24  1,2-Diacyl-sn-glycerol (dioctadec-11-enoyl, n-C18:1)  C00641  11.735
  7  Starch and sucrose metabolism 4e-08  9  Pyruvate metabolism 0.000  3  Acetyl phosphate 0.000 0.000
   ynfC   ecocyc   kegg   phen   285  rzoD  ecocyc   kegg  9.1 0.0  3  7,8-Diaminononanoate  C01037  4.071
 &nbsp &nbsp &nbsp  2  Chlorocyclohexane and chlorobenzene degradation 0.000  13  L-Galactonate 0.000 0.000
   ynfD   ecocyc   kegg   phen   230  wcaC  ecocyc   kegg  7.3 0.0 &nbsp &nbsp &nbsp
 &nbsp &nbsp &nbsp  4  Chlorocyclohexane and chlorobenzene degradation 0.000 &nbsp &nbsp &nbsp
   ynfK   ecocyc   kegg   phen   135  ygaQ  ecocyc   kegg  7.5 0.0  17  N-Acetyl-L-glutamate  C00624  3.773
  6  Chloroalkane and chloroalkene degradation 9e-07  1  Phosphonate and phosphinate metabolism 0.001  11  D-Glycerate 2-phosphate 0.000 0.000
   ynfL   ecocyc   kegg   phen   202  yeiE  ecocyc   kegg  15.5 0.0  21  Propanal  C00479  3.690
  4  Arachidonic acid metabolism 0  4  Two-component system 0.000  8  Putrescine 0.010 1.000
   ynfN   ecocyc   kegg   phen   147  nmpC  ecocyc   kegg  11.0 0.0  11  Butanal  C01412  4.290
  14  Propanoate metabolism 4e-08  3  Bisphenol degradation 0.002  3  Citrate 0.000 0.000
   ynfO   ecocyc   kegg   phen   101  ogrK  ecocyc   kegg  9.5 0.0  7  tetradecanoate (n-C14:0)  C06424  6.752
  3  Glycerophospholipid metabolism 0.0002  4  Chloroalkane and chloroalkene degradation 0.002  8  Glycerol 2-phosphate 0.000 0.000
   ynhG   ecocyc   kegg   phen   187  yeiJ  ecocyc   kegg  19.0 0.0  20  2-Acyl-sn-glycero-3-phosphoethanolamine (n-C16:1)  C05973  7.869
  5  Arachidonic acid metabolism 0  5  ABC transporters 0.001  13  3-Oxodecanoyl-CoA 0.000 0.000
   yniA   ecocyc   kegg   phen   77  yqcC  ecocyc   kegg  12.1 0.0  4  Deoxycytidine  C00881  4.995
  8  Histidine metabolism 1e-05  1  Nucleotide excision repair 0.008  1  L-Serine 0.001 0.000
   yniB   ecocyc   kegg   phen   159  ydhK  ecocyc   kegg  15.2 0.0  30  Formaldehyde  C00067  7.223
  10  Porphyrin and chlorophyll metabolism 3e-05  6  Oxidative phosphorylation 0.000  7  D-Glycerate 2-phosphate 0.000 0.000
   yniC   ecocyc   kegg   phen   130  treC  ecocyc   kegg  9.3 0.0  1  methanesulfonate  C11145  -3.532
  3  Biosynthesis of secondary metabolites 0  11  Chlorocyclohexane and chlorobenzene degradation 0.000  12  ITP 0.000 0.000
   yniD   ecocyc   kegg   phen   136  yghF  ecocyc   kegg  7.5 0.0  4  sulfur dioxide  C09306  3.592
 &nbsp &nbsp &nbsp  1  Bisphenol degradation 0.001  18  S-Formylglutathione 0.000 0.000
   ynjA   ecocyc   kegg   phen   105  dcuA  ecocyc   kegg  7.2 0.0  27  3-Methyl-2-oxobutanoate  C00141  13.975
  18  Bisphenol degradation 0.0001  9  Valine, leucine and isoleucine biosynthesis 0.000  8  2-Octaprenyl-3-methyl-5-hydroxy-6-methoxy-1,4-benzoquinol 0.000 0.000
   ynjB   ecocyc   kegg   phen   345  ydiQ  ecocyc   kegg  12.2 0.0 &nbsp &nbsp &nbsp
 &nbsp &nbsp &nbsp &nbsp &nbsp &nbsp &nbsp &nbsp &nbsp
   ynjE   ecocyc   kegg   phen   206  yegX  ecocyc   kegg  13.1 0.0  7  D-Glucosamine 1-phosphate  C06156  4.452
  11  Glycine, serine and threonine metabolism 4e-05 &nbsp &nbsp &nbsp  6  Methylglyoxal 0.000 0.000
   ynjF   ecocyc   kegg   phen   111  rcsB  ecocyc   kegg  11.4 0.0  1  5-Phospho-beta-D-ribosylamine  C03090  4.085
  1  ABC transporters 0  4  Terpenoid backbone biosynthesis 0.002  1  L-Tyrosine 0.001 0.000
   ynjH   ecocyc   kegg   phen   113  yaiI  ecocyc   kegg  9.9 0.0  6  Sedoheptulose 7-phosphate  C05382  3.581
 &nbsp &nbsp &nbsp &nbsp &nbsp &nbsp &nbsp &nbsp &nbsp
   ynjI   ecocyc   kegg   phen   206  yeiP  ecocyc   kegg  8.6 0.0  2  L-tartrate  C00898  -3.599
  1  Glyoxylate and dicarboxylate metabolism 0 &nbsp &nbsp &nbsp  5  D-Glucarate 0.000 0.000
   yoaA   ecocyc   kegg   phen   131  yfcP  ecocyc   kegg  11.6 0.0  13  Sarcosine  C00213  3.541
  17  Microbial metabolism in diverse environments 1e-07  5  Peptidoglycan biosynthesis 0.000  2  D-Fructose 1,6-bisphosphate 0.000 0.000
   yoaB   ecocyc   kegg   phen   302  yfbT  ecocyc   kegg  9.6 0.0 &nbsp &nbsp &nbsp
 &nbsp &nbsp &nbsp  3  Glycerolipid metabolism 0.001 &nbsp &nbsp &nbsp
   yoaC   ecocyc   kegg   phen   261  yodB  ecocyc   kegg  10.9 0.0  3  O-Phospho-L-serine  C01005  3.610
  5  C5-Branched dibasic acid metabolism 8e-11  4  Phosphonate and phosphinate metabolism 0.005  5  5-Dehydro-4-deoxy-D-glucarate 0.000 0.000
   yoaE   ecocyc   kegg   phen   168  yecH  ecocyc   kegg  16.8 0.0  12  cyclopropane phosphatidylglycerol (dihexadec-9,10-cyclo-anoyl, n-C16:0 cyclo)  cyclopropane phosphatidylglycerol (dihexadec-9,10-cyclo-anoyl, n-C16:0 cyclo)  5.171
  2  Aminobenzoate degradation 0  7  RNA polymerase 0.000  8  3-Dehydro-L-gulonate 0.000 0.000
   yoaF   ecocyc   kegg   phen   116  fimE  ecocyc   kegg  13.6 0.0  16  Glutathionylspermidine  C05730  5.533
  13  Amino sugar and nucleotide sugar metabolism 2e-07  1  Other glycan degradation 0.003  2  D-Alanyl-D-alanine 0.000 0.000
   yoaG   ecocyc   kegg   phen   116  ydhW  ecocyc   kegg  11.2 0.0 &nbsp &nbsp &nbsp
 &nbsp &nbsp &nbsp  7  Two-component system 0.000 &nbsp &nbsp &nbsp
   yoaH   ecocyc   kegg   phen   115  chbG  ecocyc   kegg  15.0 0.0  6  2-Acyl-sn-glycero-3-phosphoglycerol (n-C18:1)  2-Acyl-sn-glycero-3-phosphoglycerol (n-C18:1)  5.015
  6  Ethylbenzene degradation 0.0003  6  Nitrotoluene degradation 0.001  2  Fumarate 0.003 0.000
   yobA   ecocyc   kegg   phen   336  aidB  ecocyc   kegg  10.2 0.0  7  trans-Aconitate  C02341  3.902
 &nbsp &nbsp &nbsp  5  Chlorocyclohexane and chlorobenzene degradation 0.000  6  Acetol 0.001 0.000
   yobB   ecocyc   kegg   phen   95  yebE  ecocyc   kegg  9.6 0.0  18  1,4-Dihydroxy-2-naphthoate  C03657  4.872
  4  Sphingolipid metabolism 1e-07  8  RNA polymerase 0.000 &nbsp &nbsp &nbsp
   yobD   ecocyc   kegg   phen   198  yebG  ecocyc   kegg  19.0 0.0  39  cyclopropane phosphatidylethanolamine (dihexadec-9,10-cyclo-anoyl, n-C16:0 cyclo)  cyclopropane phosphatidylethanolamine (dihexadec-9,10-cyclo-anoyl, n-C16:0 cyclo)  5.724
  6  Arachidonic acid metabolism 0  4  Two-component system 0.000  5  Putrescine 0.005 1.000
   yobF   ecocyc   kegg   phen   238  hinT  ecocyc   kegg  7.7 0.0 &nbsp &nbsp &nbsp
 &nbsp &nbsp &nbsp &nbsp &nbsp &nbsp &nbsp &nbsp &nbsp
   yobH   ecocyc   kegg   phen   130  ybcC  ecocyc   kegg  8.2 0.0  5  Thiamin monophosphate  C01081  4.229
 &nbsp &nbsp &nbsp  3  Bisphenol degradation 0.001  7  L-Lysine-tRNA (Lys) 0.000 0.000
   yodB   ecocyc   kegg   phen   383  yeiW  ecocyc   kegg  13.0 0.0  4  Superoxide anion  C00704  3.500
  6  C5-Branched dibasic acid metabolism 2e-11  2  Aminoacyl-tRNA biosynthesis 0.003  19  2-Octaprenyl-6-methoxyphenol 0.000 0.000
   yodC   ecocyc   kegg   phen   148  fiu  ecocyc   kegg  9.2 0.0  27  Glutathionylspermidine  C05730  6.039
  5  Limonene and pinene degradation 2e-09  7  RNA polymerase 0.000  12  D-Glycerate 2-phosphate 0.000 0.000
   yodD   ecocyc   kegg   phen   215  yegX  ecocyc   kegg  10.4 0.0  1  Uridine  C00299  -3.802
  4  Ethylbenzene degradation 9e-05 &nbsp &nbsp &nbsp  5  D-Glucarate 0.000 0.000
   yoeE   ecocyc   kegg   phen   62  lomR  ecocyc   kegg  14.5 0.0  5  2-Oxopent-4-enoate  C00596  15.547
  11  Caprolactam degradation 3e-06 &nbsp &nbsp &nbsp  1  Reduced glutathione 0.002 0.000
   yoeF   ecocyc   kegg   phen   179  yeeF  ecocyc   kegg  13.6 0.0 &nbsp &nbsp &nbsp
 &nbsp &nbsp &nbsp  6  Bacterial secretion system 0.000 &nbsp &nbsp &nbsp
   yohC   ecocyc   kegg   phen   204  yohN  ecocyc   kegg  10.8 0.0  5  D-Glucosamine 1-phosphate  C06156  3.800
  2  Glutathione metabolism 8e-05  3  Phosphonate and phosphinate metabolism 0.003  11  (R)-Pantoate 0.000 0.000
   yohD   ecocyc   kegg   phen   80  fkpA  ecocyc   kegg  11.8 0.0  14  5'-deoxyribose  5'-deoxyribose  4.536
  6  Biosynthesis of unsaturated fatty acids 4e-07  7  Bacterial secretion system 0.001 &nbsp &nbsp &nbsp
   yohF   ecocyc   kegg   phen   55  rluC  ecocyc   kegg  7.0 0.0 &nbsp &nbsp &nbsp
 &nbsp &nbsp &nbsp  1  Cyanoamino acid metabolism 0.003 &nbsp &nbsp &nbsp
   yohG   ecocyc   kegg   phen   330  cld  ecocyc   kegg  11.2 0.0 &nbsp &nbsp &nbsp
 &nbsp &nbsp &nbsp  2  Chlorocyclohexane and chlorobenzene degradation 0.000 &nbsp &nbsp &nbsp
   yohH   ecocyc   kegg   phen   230  ydgJ  ecocyc   kegg  11.1 0.0 &nbsp &nbsp &nbsp
 &nbsp &nbsp &nbsp  1  Bisphenol degradation 0.004 &nbsp &nbsp &nbsp
   yohJ   ecocyc   kegg   phen   100  yfiD  ecocyc   kegg  12.0 0.0  21  L-methionine-R-sulfoxide  C15998  10.934
  5  Vitamin B6 metabolism 1e-05  5  Bacterial secretion system 0.000  5  Propanoyl-CoA 0.000 0.000
   yohK   ecocyc   kegg   phen   217  yeiJ  ecocyc   kegg  21.7 0.0  26  cyclopropane phosphatidylethanolamine (dihexadec-9,10-cyclo-anoyl, n-C16:0 cyclo)  cyclopropane phosphatidylethanolamine (dihexadec-9,10-cyclo-anoyl, n-C16:0 cyclo)  4.772
  4  Arachidonic acid metabolism 0  2  Toluene degradation 0.001  16  silver 0.000 0.000
   yohN   ecocyc   kegg   phen   204  yohC  ecocyc   kegg  10.8 0.0  32  Cytosine  C00380  6.574
  8  Pentose and glucuronate interconversions 3e-09  1  Sphingolipid metabolism 0.003  9  (R)-Pantoate 0.000 0.000
   yohO   ecocyc   kegg   phen   221  glcG  ecocyc   kegg  8.8 0.0  1  crotonobetaine  C04114  4.525
  1  Caprolactam degradation 3e-05  1  ABC transporters 0.008  14  L-fuculose 0.000 0.000
   ypdA   ecocyc   kegg   phen   153  yegX  ecocyc   kegg  8.7 0.0  4  (R)-Propane-1,2-diol  C02912  3.554
  7  Glycerolipid metabolism 8e-06  2  Sphingolipid metabolism 0.002  3  D-Ribulose 5-phosphate 0.000 0.000
   ypdB   ecocyc   kegg   phen   122  yejA  ecocyc   kegg  12.1 0.0 &nbsp &nbsp &nbsp
  1  Biosynthesis of secondary metabolites 0  2  Novobiocin biosynthesis 0.006 &nbsp &nbsp &nbsp
   ypdC   ecocyc   kegg   phen   97  ypjD  ecocyc   kegg  8.1 0.0  1  Hydroquinone  C00530  3.486
 &nbsp &nbsp &nbsp  1  Lipoic acid metabolism 0.002  2  Choline 0.000 0.000
   ypdJ   ecocyc   kegg   phen   100  yfiE  ecocyc   kegg  9.2 0.0 &nbsp &nbsp &nbsp
 &nbsp &nbsp &nbsp  6  Chlorocyclohexane and chlorobenzene degradation 0.000 &nbsp &nbsp &nbsp
   ypeA   ecocyc   kegg   phen   118  phnB  ecocyc   kegg  8.6 0.0  8  L-Citrulline  C00327  3.557
  6  Fructose and mannose metabolism 6e-05 &nbsp &nbsp &nbsp &nbsp &nbsp &nbsp
   ypeB   ecocyc   kegg   phen   223  yieP  ecocyc   kegg  6.9 0.0  7  Sucrose 6-phosphate  C02591  3.903
  6  Starch and sucrose metabolism 3e-11  6  Chlorocyclohexane and chlorobenzene degradation 0.000  7  3-Methyl-2-oxobutanoate 0.001 0.000
   ypeC   ecocyc   kegg   phen   120  eutQ  ecocyc   kegg  16.3 0.0  16  1,5-Diaminopentane  C01672  6.237
  3  Lysine degradation 5e-10  5  RNA polymerase 0.000  8  Cys-Gly 0.000 0.000
   ypfG   ecocyc   kegg   phen   121  atoS  ecocyc   kegg  14.6 0.0  89  cyclopropane phosphatidylglycerol (dihexadec-9,10-cyclo-anoyl, n-C16:0 cyclo)  cyclopropane phosphatidylglycerol (dihexadec-9,10-cyclo-anoyl, n-C16:0 cyclo)  6.460
  14  Arachidonic acid metabolism 0  6  RNA polymerase 0.000  4  Succinate 0.000 0.000
   ypfH   ecocyc   kegg   phen   182  ydfW  ecocyc   kegg  11.8 0.0 &nbsp &nbsp &nbsp
 &nbsp &nbsp &nbsp  4  RNA degradation 0.003 &nbsp &nbsp &nbsp
   ypfJ   ecocyc   kegg   phen   273  glxK  ecocyc   kegg  9.8 0.0  3  Glycolaldehyde  C00266  3.544
 &nbsp &nbsp &nbsp  5  Chlorocyclohexane and chlorobenzene degradation 0.000  10  Acetol 0.001 0.000
   ypfN   ecocyc   kegg   phen   142  yjeK  ecocyc   kegg  10.7 0.0  12  4-Hydroxybenzoate  C00156  3.886
  3  Pyruvate metabolism 0.0002  15  Chlorocyclohexane and chlorobenzene degradation 0.000  7  5-Methyltetrahydrofolate 0.000 0.000
   yphA   ecocyc   kegg   phen   97  yaiI  ecocyc   kegg  10.1 0.0  2  N2-Succinyl-L-ornithine  C03415  3.727
  2  Novobiocin biosynthesis 0  2  Biosynthesis of unsaturated fatty acids 0.006 &nbsp &nbsp &nbsp
   yphB   ecocyc   kegg   phen   417  glyS  ecocyc   kegg  21.0 0.0  6  Glycolaldehyde  C00266  5.187
  15  C5-Branched dibasic acid metabolism 4e-11  1  Aminoacyl-tRNA biosynthesis 0.004  28  4-Aminobenzoate 0.000 0.000
   yphC   ecocyc   kegg   phen   270  yihO  ecocyc   kegg  12.3 0.0 &nbsp &nbsp &nbsp
 &nbsp &nbsp &nbsp  6  Chlorocyclohexane and chlorobenzene degradation 0.000 &nbsp &nbsp &nbsp
   yphG   ecocyc   kegg   phen   346  ydfO  ecocyc   kegg  10.5 0.0  1  1,2-Diacyl-sn-glycerol (didodecanoyl, n-C12:0)  C00641  3.774
  1  Biosynthesis of secondary metabolites 0  3  Mismatch repair 0.000  10  Phenylacetic acid 0.000 0.000
   yphH   ecocyc   kegg   phen   389  yegX  ecocyc   kegg  14.7 0.0  6  D-Glucosamine 1-phosphate  C06156  3.662
  3  Purine metabolism 1e-05  1  Two-component system 0.008  18  D-Glycerate 2-phosphate 0.000 0.000
   ypjB   ecocyc   kegg   phen   114  uhpB  ecocyc   kegg  23.0 0.0  81  S-Adenosylmethioninamine  C01137  20.217
  12  Arachidonic acid metabolism 6e-15  10  RNA polymerase 0.000  4  silver 0.000 0.000
   ypjC   ecocyc   kegg   phen   262  pbl  ecocyc   kegg  10.6 0.0  5  D-Glucosamine 1-phosphate  C06156  4.582
 &nbsp &nbsp &nbsp &nbsp &nbsp &nbsp  8  glucosyl-O-acetyl-rhamanosyl-N-acetylglucosamyl-undecaprenyl diphosphate 0.000 0.000
   ypjD   ecocyc   kegg   phen   47  yeiR  ecocyc   kegg  23.8 0.0  9  Oxalureate  C00802  9.600
 &nbsp &nbsp &nbsp  3  Folate biosynthesis 0.000  1  octanoate (n-C8:0) 0.000 0.000
   ypjJ   ecocyc   kegg   phen   151  exo  ecocyc   kegg  9.0 0.0 &nbsp &nbsp &nbsp
 &nbsp &nbsp &nbsp  2  Bisphenol degradation 0.002 &nbsp &nbsp &nbsp
   ypjL   ecocyc   kegg   phen   186  yahC  ecocyc   kegg  10.9 0.0 &nbsp &nbsp &nbsp
 &nbsp &nbsp &nbsp  13  Chlorocyclohexane and chlorobenzene degradation 0.000 &nbsp &nbsp &nbsp
   yqaA   ecocyc   kegg   phen   96  yfiA  ecocyc   kegg  8.9 0.0  9  2-Succinyl-6-hydroxy-2,4-cyclohexadiene-1-carboxylate  C05817  3.666
  2  Ubiquinone and other terpenoid-quinone biosynthesis 0  6  Bisphenol degradation 0.001  8  Maltoheptaose 0.000 0.000
   yqaD   ecocyc   kegg   phen   108  zraS  ecocyc   kegg  14.0 0.0  42  S-Adenosylmethioninamine  C01137  10.938
  12  Fluorobenzoate degradation 4e-08  4  Two-component system 0.000  8  D-Lactate 0.000 0.000
   yqaE   ecocyc   kegg   phen   124  ybgH  ecocyc   kegg  12.0 0.0  14  4-Aminobenzoate  C00568  3.661
  10  Arginine and proline metabolism 3e-05  1  Oxidative phosphorylation 0.003  7  (-)-Ureidoglycolate 0.000 0.000
   yqcC   ecocyc   kegg   phen   60  yniA  ecocyc   kegg  12.1 0.0  3  cyclopropane phosphatidylethanolamine (dihexadec-9,10-cyclo-anoyl, n-C16:0 cyclo)  cyclopropane phosphatidylethanolamine (dihexadec-9,10-cyclo-anoyl, n-C16:0 cyclo)  4.327
  1  Pantothenate and CoA biosynthesis 0  1  Pyrimidine metabolism 0.000  2  Deoxyuridine 0.000 0.000
   yqeA   ecocyc   kegg   phen   175  yhfK  ecocyc   kegg  8.0 0.0 &nbsp &nbsp &nbsp
 &nbsp &nbsp &nbsp  7  Chlorocyclohexane and chlorobenzene degradation 0.000 &nbsp &nbsp &nbsp
   yqeB   ecocyc   kegg   phen   89  priB  ecocyc   kegg  8.2 0.0  2  1,2-didodecanoyl-sn-glycerol 3-phosphate  C00416  4.649
 &nbsp &nbsp &nbsp  4  Arachidonic acid metabolism 0.001  2  D-Glucose 6-phosphate 0.000 0.000
   yqeC   ecocyc   kegg   phen   141  ygiB  ecocyc   kegg  8.4 0.0  1  dTTP  C00459  3.628
 &nbsp &nbsp &nbsp &nbsp &nbsp &nbsp  7  Dimethyl sulfide 0.000 0.000
   yqeF   ecocyc   kegg   phen   100  msrA  ecocyc   kegg  13.5 0.0  12  gamma-butyrobetaine  C01181  9.441
  4  Limonene and pinene degradation 0  1  Ribosome 0.005  1  Acetaldehyde 0.003 0.000
   yqeG   ecocyc   kegg   phen   264  fldB  ecocyc   kegg  11.7 0.0  1  Phenylacetic acid  C07086  7.446
  6  Microbial metabolism in diverse environments 5e-08  16  Cysteine and methionine metabolism 0.000  38  Aerobactin 0.000 0.000
   yqeH   ecocyc   kegg   phen   200  nadR  ecocyc   kegg  11.4 0.0  10  Glycolaldehyde  C00266  3.975
  3  Biotin metabolism 7e-06 &nbsp &nbsp &nbsp  11  [4Fe-4S] iron-sulfur cluster 0.000 0.000
   yqeI   ecocyc   kegg   phen   156  dacA  ecocyc   kegg  17.8 0.0  26  Pyridoxamine  C00534  6.718
  3  Lysine degradation 1e-09  8  RNA polymerase 0.000  10  Fumarate 0.000 0.000
   yqeJ   ecocyc   kegg   phen   154  ybfO  ecocyc   kegg  15.9 0.0  21  2-Dehydro-3-deoxy-D-galactonate 6-phosphate  C01286  3.980
  14  Phosphotransferase system (PTS) 2e-05  1  Caprolactam degradation 0.009  3  D-Ribulose 5-phosphate 0.001 0.000
   yqeK   ecocyc   kegg   phen   243  wbbK  ecocyc   kegg  9.1 0.0 &nbsp &nbsp &nbsp
 &nbsp &nbsp &nbsp &nbsp &nbsp &nbsp &nbsp &nbsp &nbsp
   yqfA   ecocyc   kegg   phen   61  rfaY  ecocyc   kegg  7.0 0.0  2  Butanal  C01412  4.576
  3  Tyrosine metabolism 0  7  Chlorocyclohexane and chlorobenzene degradation 0.000  4  Oxidized thioredoxin 0.006 0.000
   yqfB   ecocyc   kegg   phen   76  ydiU  ecocyc   kegg  7.7 0.0 &nbsp &nbsp &nbsp
 &nbsp &nbsp &nbsp  1  Homologous recombination 0.006 &nbsp &nbsp &nbsp
   yqfE   ecocyc   kegg   phen   116  yjhG  ecocyc   kegg  6.7 0.0 &nbsp &nbsp &nbsp
 &nbsp &nbsp &nbsp  8  Chlorocyclohexane and chlorobenzene degradation 0.000 &nbsp &nbsp &nbsp
   yqgB   ecocyc   kegg   phen   246  yhcE  ecocyc   kegg  12.5 0.0  44  1-(2-Carboxyphenylamino)-1-deoxy-D-ribulose 5-phosphate  C01302  5.643
  15  Microbial metabolism in diverse environments 4e-08  2  Sphingolipid metabolism 0.004  12  Sulfate 0.008 1.000
   yqgC   ecocyc   kegg   phen   191  rfaZ  ecocyc   kegg  6.6 0.0 &nbsp &nbsp &nbsp
 &nbsp &nbsp &nbsp  1  Mismatch repair 0.005 &nbsp &nbsp &nbsp
   yqgE   ecocyc   kegg   phen   116  yggJ  ecocyc   kegg  11.8 0.0  15  N-Acetyl-L-glutamate  C00624  4.962
  10  Lysine degradation 8e-08  1  Ribosome 0.008  8  dehydroglycine 0.000 0.000
   yqhA   ecocyc   kegg   phen   175  ompG  ecocyc   kegg  7.8 0.0  4  6-phospho-D-glucono-1,5-lactone  C01236  3.953
 &nbsp &nbsp &nbsp  2  Chloroalkane and chloroalkene degradation 0.006  8  O-acetyl-rhamanosyl-N-acetylglucosamyl-undecaprenyl diphosphate 0.000 0.000
   yqhC   ecocyc   kegg   phen   207  yeeJ  ecocyc   kegg  9.8 0.0 &nbsp &nbsp &nbsp
 &nbsp &nbsp &nbsp  9  Chlorocyclohexane and chlorobenzene degradation 0.000 &nbsp &nbsp &nbsp
   yqhG   ecocyc   kegg   phen   99  yddA  ecocyc   kegg  7.7 0.0  1  Dimethyl sulfoxide  C11143  -3.644
 &nbsp &nbsp &nbsp  4  Other glycan degradation 0.002  10  Butanoyl-CoA 0.000 0.000
   yqiA   ecocyc   kegg   phen   109  znuC  ecocyc   kegg  6.5 0.0 &nbsp &nbsp &nbsp
 &nbsp &nbsp &nbsp  2  Nucleotide excision repair 0.001 &nbsp &nbsp &nbsp
   yqiB   ecocyc   kegg   phen   95  ybeA  ecocyc   kegg  6.8 0.0 &nbsp &nbsp &nbsp
 &nbsp &nbsp &nbsp  7  Arachidonic acid metabolism 0.001 &nbsp &nbsp &nbsp
   yqiC   ecocyc   kegg   phen   131  rzoR  ecocyc   kegg  10.7 0.0  6  1,2-Diacyl-sn-glycerol (dioctadec-11-enoyl, n-C18:1)  C00641  4.234
  3  Phenylalanine metabolism 1e-05  8  Biosynthesis of siderophore group nonribosomal peptides 0.000  10  2,3-Dihydro-2,3-dihydroxybenzoate 0.000 0.000
   yqiG   ecocyc   kegg   phen   124  ycdC  ecocyc   kegg  9.4 0.0  2  Acetate  C00033  -3.602
 &nbsp &nbsp &nbsp &nbsp &nbsp &nbsp  2  Succinic semialdehyde 0.000 0.000
   yqiH   ecocyc   kegg   phen   121  exo  ecocyc   kegg  7.0 0.0 &nbsp &nbsp &nbsp
 &nbsp &nbsp &nbsp  2  Galactose metabolism 0.000 &nbsp &nbsp &nbsp
   yqiI   ecocyc   kegg   phen   86  mdoB  ecocyc   kegg  8.7 0.0  5  Melibiose  C05402  3.693
  3  Bisphenol degradation 0.0001  3  Ribosome 0.003  3  D-Sorbitol 6-phosphate 0.000 0.000
   yqiJ   ecocyc   kegg   phen   81  mdaB  ecocyc   kegg  8.2 0.0  4  (S)-2-Aceto-2-hydroxybutanoate  C06006  4.598
 &nbsp &nbsp &nbsp  6  Valine, leucine and isoleucine biosynthesis 0.006 &nbsp &nbsp &nbsp
   yqiK   ecocyc   kegg   phen   130  yqjA  ecocyc   kegg  12.3 0.0  5  5-Phospho-beta-D-ribosylamine  C03090  3.561
 &nbsp &nbsp &nbsp  5  Arachidonic acid metabolism 0.001  4  GTP 0.001 0.000
   yqjA   ecocyc   kegg   phen   146  ygjV  ecocyc   kegg  14.9 0.0  40  Hydroxypyruvate  C00168  5.396
  8  Pyrimidine metabolism 0.0003  6  Ethylbenzene degradation 0.004  1  Ring 1,2-epoxyphenylacetyl-CoA 0.001 0.000
   yqjC   ecocyc   kegg   phen   176  torI  ecocyc   kegg  7.9 0.0 &nbsp &nbsp &nbsp
 &nbsp &nbsp &nbsp  1  Ribosome 0.003 &nbsp &nbsp &nbsp
   yqjD   ecocyc   kegg   phen   157  yjeK  ecocyc   kegg  11.0 0.0  1  CTP  C00063  3.548
 &nbsp &nbsp &nbsp  14  Pantothenate and CoA biosynthesis 0.000  19  Aerobactin 0.000 0.000
   yqjE   ecocyc   kegg   phen   30  ygjI  ecocyc   kegg  30.3 0.0  11  2-Acyl-sn-glycero-3-phosphoethanolamine (n-C16:1)  C05973  16.214
  3  Biosynthesis of secondary metabolites 0.0005  7  Protein export 0.001  1  Succinate 0.001 0.000
   yqjF   ecocyc   kegg   phen   168  pnp  ecocyc   kegg  12.6 0.0  4  O-Phospho-L-serine  C01005  7.583
  12  Glycine, serine and threonine metabolism 2e-07  10  Chlorocyclohexane and chlorobenzene degradation 0.000  17  Adenosine 5'-phosphosulfate 0.000 0.000
   yqjG   ecocyc   kegg   phen   139  yjeK  ecocyc   kegg  10.5 0.0  3  L-Arginine  C00062  -4.390
  1  Inositol phosphate metabolism 4e-09  12  Chlorocyclohexane and chlorobenzene degradation 0.000  15  4-Phospho-L-aspartate 0.000 0.000
   yqjH   ecocyc   kegg   phen   75  ygiE  ecocyc   kegg  8.7 0.0  3  4-Methyl-2-oxopentanoate  C00233  3.558
 &nbsp &nbsp &nbsp  7  Novobiocin biosynthesis 0.002  3  Co2+ 0.000 0.000
   yqjI   ecocyc   kegg   phen   213  yqjA  ecocyc   kegg  12.1 0.0  19  N-Acetyl-L-glutamate  C00624  6.500
  9  Lysine degradation 2e-07  3  Glycerolipid metabolism 0.002  15  bis-molybdopterin guanine dinucleotide 0.000 0.000
   yqjK   ecocyc   kegg   phen   158  btuB  ecocyc   kegg  12.3 0.0  8  crotonobetaine  C04114  4.406
  2  Caprolactam degradation 3e-05  3  ABC transporters 0.000  10  L-Arginine 0.000 0.000
   yraH   ecocyc   kegg   phen   97  pnuC  ecocyc   kegg  11.1 0.0 &nbsp &nbsp &nbsp
 &nbsp &nbsp &nbsp  10  Valine, leucine and isoleucine degradation 0.000 &nbsp &nbsp &nbsp
   yraJ   ecocyc   kegg   phen   26  yagK  ecocyc   kegg  24.3 0.0  36  N-Acetylneuraminate  C00270  13.495
  6  alpha-Linolenic acid metabolism 1e-09  3  Inositol phosphate metabolism 0.000  2  Glyceraldehyde 3-phosphate 0.000 0.000
   yraK   ecocyc   kegg   phen   132  ygiL  ecocyc   kegg  9.6 0.0  4  Tetradecanoyl-phosphate (n-C14:1)  Tetradecanoyl-phosphate (n-C14:1)  3.556
  2  Phenylalanine metabolism 3e-05  3  Other glycan degradation 0.003  8  Putrescine 0.002 1.000
   yraM   ecocyc   kegg   phen   119  dsbC  ecocyc   kegg  7.5 0.0 &nbsp &nbsp &nbsp
 &nbsp &nbsp &nbsp  9  Chlorocyclohexane and chlorobenzene degradation 0.000 &nbsp &nbsp &nbsp
   yraN   ecocyc   kegg   phen   211  ygfM  ecocyc   kegg  7.9 0.0  1  Bicarbonate  C00288  3.900
 &nbsp &nbsp &nbsp &nbsp &nbsp &nbsp  11  O-acetyl-rhamanosyl-N-acetylglucosamyl-undecaprenyl diphosphate 0.000 0.000
   yraP   ecocyc   kegg   phen   135  yjeK  ecocyc   kegg  11.4 0.0  1  gamma-hydroxybutyrate  C00989  3.475
 &nbsp &nbsp &nbsp  12  Chlorocyclohexane and chlorobenzene degradation 0.000  12  heptosyl-phospho-heptosyl-heptosyl-kdo2-lipidA 0.000 0.000
   yraQ   ecocyc   kegg   phen   117  ushA  ecocyc   kegg  9.0 0.0  5  2-Acyl-sn-glycero-3-phosphoethanolamine (n-C14:0)  C05973  3.457
 &nbsp &nbsp &nbsp  10  Ascorbate and aldarate metabolism 0.000  8  3-Dehydro-L-gulonate 0.000 0.000
   yraR   ecocyc   kegg   phen   120  xdhA  ecocyc   kegg  8.3 0.0 &nbsp &nbsp &nbsp
 &nbsp &nbsp &nbsp  7  Galactose metabolism 0.000 &nbsp &nbsp &nbsp
   yrbG   ecocyc   kegg   phen   178  yfjG  ecocyc   kegg  9.5 0.0 &nbsp &nbsp &nbsp
  1  Biosynthesis of secondary metabolites 0  6  Bacterial secretion system 0.002 &nbsp &nbsp &nbsp
   yrbL   ecocyc   kegg   phen   129  yhiN  ecocyc   kegg  7.6 0.0  3  sn-Glycero-3-phospho-1-inositol  C01225  3.680
 &nbsp &nbsp &nbsp  8  Nucleotide excision repair 0.001  25  5-Phospho-beta-D-ribosylamine 0.000 0.000
   yrdB   ecocyc   kegg   phen   154  gadX  ecocyc   kegg  8.1 0.0  5  Aminoacetone  C01888  3.831
 &nbsp &nbsp &nbsp  5  C5-Branched dibasic acid metabolism 0.005  4  L-Homocysteine 0.000 0.000
   yrdD   ecocyc   kegg   phen   93  bipA  ecocyc   kegg  11.8 0.0 &nbsp &nbsp &nbsp
 &nbsp &nbsp &nbsp  4  Bacterial secretion system 0.000 &nbsp &nbsp &nbsp
   yrhA   ecocyc   kegg   phen   565  dgoT  ecocyc   kegg  13.8 0.0  1  2-hydroxy-6-ketononatrienedioate  C12624  4.798
 &nbsp &nbsp &nbsp  5  Chlorocyclohexane and chlorobenzene degradation 0.000  49  bis-molybdenum cofactor 0.000 0.000
   yrhB   ecocyc   kegg   phen   143  yhgE  ecocyc   kegg  7.3 0.0  2  5-Formamido-1-(5-phospho-D-ribosyl)imidazole-4-carboxamide  C04734  3.590
 &nbsp &nbsp &nbsp  4  DNA replication 0.004  6  1,4-alpha-D-glucan 0.000 0.000
   ysaB   ecocyc   kegg   phen   91  yjgI  ecocyc   kegg  7.6 0.0 &nbsp &nbsp &nbsp
 &nbsp &nbsp &nbsp  6  Chlorocyclohexane and chlorobenzene degradation 0.000 &nbsp &nbsp &nbsp
   ysgA   ecocyc   kegg   phen   595  yigL  ecocyc   kegg  11.4 0.0 &nbsp &nbsp &nbsp
  1  Biosynthesis of secondary metabolites 0  5  Lipopolysaccharide biosynthesis 0.000 &nbsp &nbsp &nbsp
   ytfA   ecocyc   kegg   phen   104  argI  ecocyc   kegg  11.6 0.0  3  Uridine  C00299  4.079
  10  Aminobenzoate degradation 6e-07  1  Mismatch repair 0.001  3  Molybdate 0.000 0.000
   ytfB   ecocyc   kegg   phen   84  trkH  ecocyc   kegg  6.8 0.0  13  octanoate (n-C8:0)  C06423  7.384
  3  Microbial metabolism in diverse environments 0  3  Ascorbate and aldarate metabolism 0.000  4  D-Galactonate 0.000 0.000
   ytfF   ecocyc   kegg   phen   123  sra  ecocyc   kegg  6.4 0.0 &nbsp &nbsp &nbsp
 &nbsp &nbsp &nbsp  8  Ribosome 0.001 &nbsp &nbsp &nbsp
   ytfH   ecocyc   kegg   phen   118  fkpA  ecocyc   kegg  9.3 0.0  3  Hydroxypyruvate  C00168  3.816
 &nbsp &nbsp &nbsp  6  Bacterial secretion system 0.000  3  D-Tagaturonate 0.000 0.000
   ytfI   ecocyc   kegg   phen   78  frlC  ecocyc   kegg  7.3 0.0 &nbsp &nbsp &nbsp
 &nbsp &nbsp &nbsp  4  RNA degradation 0.002 &nbsp &nbsp &nbsp
   ytfJ   ecocyc   kegg   phen   136  gadX  ecocyc   kegg  7.3 0.0  4  Oxidized glutathione  C00127  5.355
  2  Arachidonic acid metabolism 1e-05  12  Chlorocyclohexane and chlorobenzene degradation 0.000  20  Aerobactin 0.000 0.000
   ytfK   ecocyc   kegg   phen   349  yoeF  ecocyc   kegg  10.7 0.0  4  sulfur dioxide  C09306  3.653
 &nbsp &nbsp &nbsp  8  Bacterial secretion system 0.000  45  Crotonoyl-CoA 0.000 0.000
   ytfL   ecocyc   kegg   phen   127  basS  ecocyc   kegg  15.7 0.0  28  Oxidized glutathione  C00127  8.322
  2  Glycerophospholipid metabolism 9e-05  11  RNA polymerase 0.000  11  Taurine 0.000 0.000
   ytfN   ecocyc   kegg   phen   116  hsrA  ecocyc   kegg  9.3 0.0  6  p-Cresol  C01468  4.547
  6  Toluene degradation 1e-09  3  Homologous recombination 0.000  7  Taurine 0.000 0.000
   ytfP   ecocyc   kegg   phen   126  yifB  ecocyc   kegg  11.8 0.0  27  dTDP-L-rhamnose  C03319  4.707
  11  Polyketide sugar unit biosynthesis 8e-09  5  Phosphonate and phosphinate metabolism 0.001  7  Aerobactin 0.000 0.000
   ytjB   ecocyc   kegg   phen   186  sbmC  ecocyc   kegg  12.9 0.0 &nbsp &nbsp &nbsp
 &nbsp &nbsp &nbsp  9  Chlorocyclohexane and chlorobenzene degradation 0.000 &nbsp &nbsp &nbsp
   yzcX   ecocyc   kegg   phen   110  garP  ecocyc   kegg  9.5 0.0  2  Iminoaspartate  C05840  3.889
  4  Riboflavin metabolism 4e-05  3  Biosynthesis of siderophore group nonribosomal peptides 0.000  19  2,3-Dihydro-2,3-dihydroxybenzoate 0.000 0.000
   yzfA   ecocyc   kegg   phen   101  tauB  ecocyc   kegg  8.9 0.0  20  Thiamin monophosphate  C01081  4.762
  9  Lipopolysaccharide biosynthesis 4e-09  11  Lysine degradation 0.002  27  2-dodecanoyl-sn-glycerol 3-phosphate 0.000 0.000
   yzgL   ecocyc   kegg   phen   255  tiaE  ecocyc   kegg  14.3 0.0  5  &nbsp &nbsp
 &nbsp &nbsp &nbsp  19  Chlorocyclohexane and chlorobenzene degradation 0.000  20  2-Octaprenyl-6-methoxyphenol 0.000 0.000
  
 
