# Supplementary material for: Genomewide landscape of gene–metabolome associations in Escherichia coli
Source: Mol Syst Biol. 2017 Jan 16;13(1):907. doi: 10.15252/msb.20167150 (PMC5293155; doi:10.15252/msb.20167150)
Supplement: Supplementary file 4 — Table EV3 [file MSB-13-907-s004.zip › Table_EV3_Legend.docx]

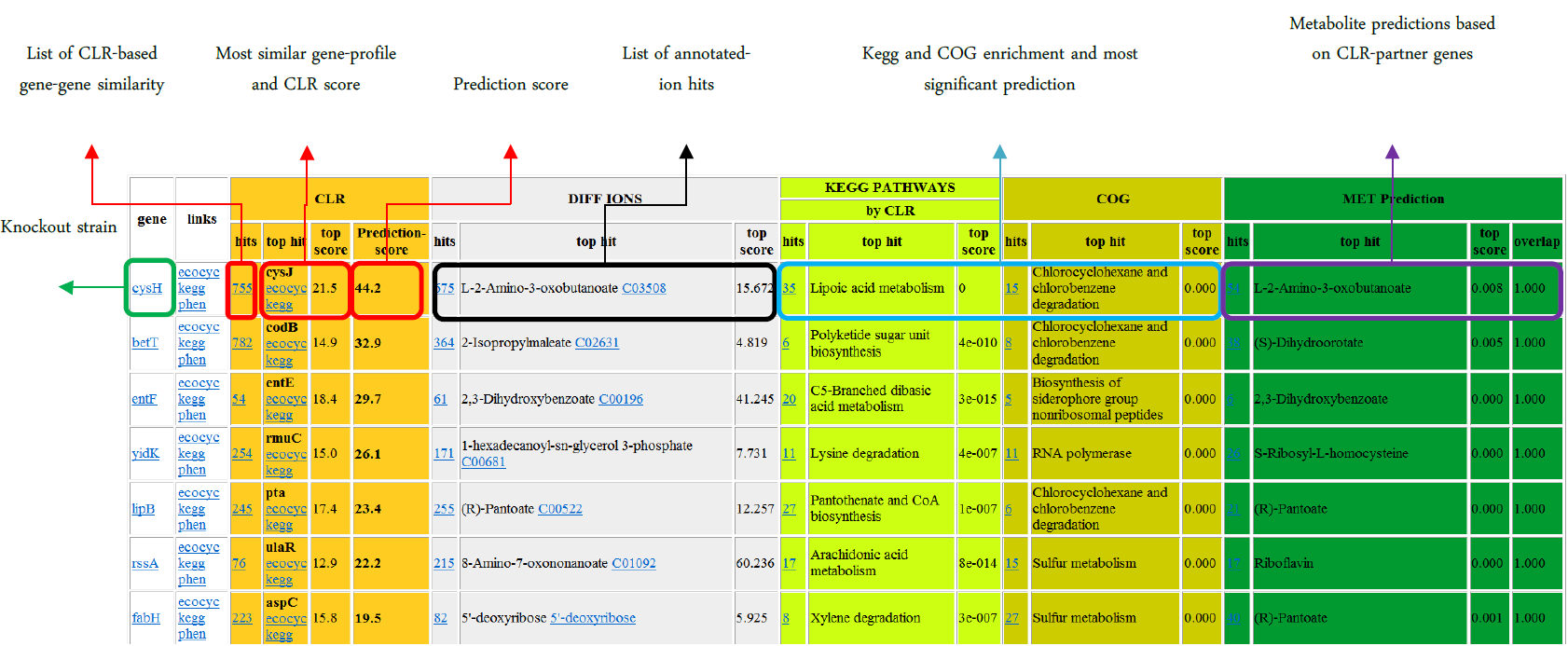


Each row corresponds to one gene deletion.

Orange columns report: number of gene deletions with significantly similar metabolome profiles; gene deletion with the highest similarity and corresponding similarity score; prediction score calculated as explained in the material and method section.

Grey columns report: number of individuated differential ions; annotated metabolite with the largest change and corresponding Z-score.

Light green columns report: Number of significantly enriched KEGG metabolic pathways among most similar gene deletions; most significant enriched pathway and corresponding significance by means of p-value.

Brown columns report: Number of significantly enriched Cluster of Orthologous Groups (COG) among most similar gene deletions; most significant enriched pathway and corresponding significance by means of p-value.

Dark green columns report: Number of significantly enriched metabolites among those directly linked (e.g. substrate or products) to most similar gene deletions; most significant overrepresented metabolite and whether the metabolite is also detected as significantly changed in our metabolome screen (i.e. 0 or 1).
