# Supplementary material for: Genomewide landscape of gene–metabolome associations in Escherichia coli
Source: Mol Syst Biol. 2017 Jan 16;13(1):907. doi: 10.15252/msb.20167150 (PMC5293155; doi:10.15252/msb.20167150)
Supplement: Supplementary file 4 — Table EV3 [file MSB-13-907-s004.zip › details/data_yaaU.html]

 
 
 yaaU 
  yaaU - details 
 
 
  CLR  
   Gene_matching CLR_index  yjeK 11.3
  yjdF 10.7
  holD 10.0
  hemX 9.6
  yjfJ 8.7
  fldB 8.4
  fhuD 8.3
  yhbS 8.2
  betT 8.1
  fhuC 8.1
  yjfK 7.6
  tnaA 7.4
  yneJ 7.4
  codB 7.3
  gadX 7.1
  ilvA 7.0
  hsdS 7.0
  panC 6.9
  yjgR 6.8
  lpp 6.7
  yieI 6.6
  yjhX 6.5
  yjeJ 6.4
  yjfL 6.4
  metL 6.3
  yijP 6.2
  ilvB 6.1
  yieF 6.1
  fdhD 5.8
  ruvB 5.8
  ynfG 5.8
  flgK 5.7
  ygeR 5.7
  mdlA 5.6
  yjiO 5.6
  cbrC 5.5
  gshA 5.5
  ymcA 5.4
  fpr 5.4
  ybdR 5.3
  tauB 5.2
  yjfY 5.2
  rhtA 5.2
  treC 5.2
  rlmB 5.1
  cysH 5.1
  rnb 5.1
  yidI 5.0
  yjfI 5.0
  gcl 4.9
  ade 4.9
  gspO 4.8
  ccmH 4.8
  yjfC 4.8
  yjbA 4.8
  srlB 4.8
  ygjM 4.7
  ilvE 4.7
  glgB 4.7
  yjdL 4.7
  mlrA 4.7
  ytfQ 4.7
  ushA 4.6
  yjeO 4.6
  yidA 4.6
  setA 4.6
  yaiI 4.5
  thiE 4.5
  atoA 4.5
  glmM 4.5
  metB 4.5
  ybaV 4.4
  rlpA 4.4
  dnaQ 4.4
  ulaC 4.4
  ygiH 4.3
  ccmF 4.3
  nohB 4.3
  yjeS 4.3
  mdtL 4.3
  ybcN 4.3
  ytfG 4.2
  yjeH 4.2
  ycjP 4.2
  deoC 4.2
  acpT 4.1
  etp 4.1
  mobA 4.1
  mfd 4.1
  yjhG 4.1
  cbpA 4.1
  lysC 4.1
  ilvM 4.1
  nagD 4.0
  fruK 4.0
  pdxJ 4.0
  pncB 4.0
  bcp 4.0
  rffC 4.0
  yghW 4.0
  panB 4.0
  yfcV 4.0
  yahK 4.0
  yigA 4.0
  araG 4.0
  hdeA 4.0
  ruvC 3.9
  hyuA 3.9
  cmtA 3.9
  nanE 3.9
  damX 3.9
  recT 3.9
  oxyR 3.9
  yidJ 3.8
  kefC 3.8
  yihM 3.8
  yadG 3.8
  tyrP 3.8
  rng 3.8
  ybeA 3.8
  ygjQ 3.8
  hofB 3.8
  yiaU 3.8
  pldA 3.8
  yhhW 3.8
  cchA 3.7
  cynX 3.7
  pfkA 3.7
  adiC 3.7
  gcvP 3.7
  metE 3.7
  aroG 3.7
  yhgF 3.7
  ygjV 3.7
  moaB 3.7
  kefA 3.7
  gudX 3.6
  cyaY 3.6
  hsrA 3.6
  ygcN 3.6
  abgB 3.6
  yggV 3.6
  yddL 3.6
  yjeT 3.6
  yhcO 3.5
  yraP 3.5
  thiC 3.5
  yfcP 3.5
  yqeG 3.5
  yigB 3.5
  tolQ 3.5
  ygcW 3.5
  yqjD 3.5
  rfaJ 3.5
  cld 3.5
  csgE 3.4
  asnA 3.4
  sbmC 3.4
  yhbE 3.4
  nhaA 3.4
  rffA 3.4
  ybdJ 3.4
  yiaA 3.4
  yhjB 3.4
  aroP 3.4
  ybgP 3.4
  csgF 3.4
  yggD 3.4
  glnH 3.4
  metH 3.3
  yfcD 3.3
  secB 3.3
  fkpA 3.3
  cysG 3.3
  frvA 3.3
  yjfM 3.3
  yjdI 3.3
  dcuR 3.3
  sodA 3.3
  yjjU 3.3
  yhgE 3.3
  yjbH 3.3
  secG 3.3
  aroH 3.3
  nanA 3.3
  ubiG 3.2
  ptsP 3.2
  yiiU 3.2
  nrdH 3.2
  yedP 3.2
  edd 3.2
  narZ 3.2
  ycbU 3.2
  bipA 3.2
  yicS 3.2
  ydaS 3.2
  sgcB 3.1
  yhaM 3.1
  yjfZ 3.1
  dadX 3.1
  yjeP 3.1
  yhfL 3.1
  yahC 3.1
  yafU 3.1
  yihN 3.1
  crl 3.1
  ygcL 3.1
  yigF 3.1
  ycjW 3.1
  yqeA 3.1
  yhhM 3.1
  glgP 3.1
  yghA 3.1
  trmE 3.1
  gss 3.1
  hyaF 3.1
  ulaB 3.0
  yceK 3.0
  yccC 3.0
  fucO 3.0
  yqeB 3.0
  exuT 3.0
  yfjI 3.0
  araJ 3.0
  yibL 3.0
  fliT 3.0
  mrr 3.0
  frlR 3.0
  aldB 3.0
  glcC 3.0
     Differential ions  
   id name formula mz mod AUC Z-score Z-score AUC Weighted   C00624  N-Acetyl-L-glutamate C7H11NO5 212.0537 .H/Na.H(+) 0.811 4.514 3.663
   C00624  N-Acetyl-L-glutamate C7H11NO5 212.0537 .Na(+) 0.811 4.514 3.663
   C00624  N-Acetyl-L-glutamate C7H11NO5 228.0226 .H/K.H(+) 0.697 4.498 3.137
   C03287  L-Glutamate 5-phosphate C5H10NO7P 228.0226 .H(+) 0.663 4.498 2.981
   C00526  Deoxyuridine C9H12N2O5 212.0537 -NH3.H(+) 0.654 4.514 2.951
   C00931  Porphobilinogen C10H14N2O4 496.9598 .(H2PO4K)2-H(+) 0.000 -3.518 -0.000
     KEGG pathway by CLR  
   Pathway_ion pvalue_ion qvalue_ion  Tryptophan metabolism 7e-09 0.0000
  Nitrotoluene degradation 0.002 0.0909
  Arginine and proline metabolism 0.007 0.2325
     COG enrichment  
   Pathway_MS pvalue_MS qvalue_MS  Pantothenate and CoA biosynthesis 2e-06 0.0002
  Valine, leucine and isoleucine biosynthesis 0.004 0.1348
  Thiamine metabolism 0.004 0.1135
  Homologous recombination 0.005 0.0870
  Cysteine and methionine metabolism 0.005 0.0722
  Protein export 0.006 0.0823
     Predicted metabolites from CLR  
   Predicted metabolites Pvalue Overlap with hits  dTDP-4-amino-4,6-dideoxy-D-galactose 0 0.0000
  4-Phospho-L-aspartate 0.0002 0.0000
  5-Methyltetrahydrofolate 0.0002 0.0000
  Aerobactin 0.0002 0.0000
  coprogen 0.0002 0.0000
  dihydrosirohydrochlorin 0.0002 0.0000
  Fe(III)hydroxamate 0.0002 0.0000
  Ferrichrome 0.0002 0.0000
  Uroporphyrinogen III 0.0002 0.0000
  5,6,7,8-Tetrahydrofolate 0.0005 0.0000
  2-Dehydro-3-deoxy-D-arabino-heptonate 7-phosphate 0.0009 0.0000
  branching glycogen 0.0009 0.0000
  3-Methyl-2-oxobutanoate 0.002 0.0000
  glycogen 0.002 0.0000
  L-ascorbate-6-phosphate 0.004 0.0000
  ferroxamine 0.004 0.0000
  L-Homoserine 0.004 0.0000
  5,10-Methylenetetrahydrofolate 0.004 0.0000
  L-Phenylalanine 0.004 0.0000
  dATP 0.007 0.0000
  dUTP 0.007 0.0000
  L-Homocysteine 0.007 0.0000
  L-Tyrosine 0.007 0.0000
  UTP 0.007 0.0000
  S-Adenosyl-L-homocysteine 0.008 0.0000
  dGTP 0.01 0.0000
  D-Erythrose 4-phosphate 0.01 0.0000
  D-Fructose 1,6-bisphosphate 0.01 0.0000
    
 
