# Supplementary material for: Genomewide landscape of gene–metabolome associations in Escherichia coli
Source: Mol Syst Biol. 2017 Jan 16;13(1):907. doi: 10.15252/msb.20167150 (PMC5293155; doi:10.15252/msb.20167150)
Supplement: Supplementary file 4 — Table EV3 [file MSB-13-907-s004.zip › details/data_yaaY.html]

 
 
 yaaY 
  yaaY - details 
 
 
  CLR  
   Gene_matching CLR_index  yrhA 9.4
  yhjC 9.2
  ycbG 9.2
  dgoT 9.0
  ybfN 8.7
  yidR 8.7
  emtA 8.6
  yobA 8.5
  aidB 8.3
  bioH 8.2
  hokC 7.8
  aroK 7.7
  mobB 7.6
  gpp 7.6
  yicJ 7.5
  amtB 7.4
  modE 7.4
  tfaS 7.3
  yjjM 7.3
  ygcW 7.3
  yjgF 7.3
  proY 7.2
  torS 7.2
  marC 7.1
  cspE 7.1
  yjfN 7.1
  yjfP 7.0
  yohG 7.0
  ybjG 6.9
  gntU 6.9
  trmA 6.9
  yiiF 6.9
  ybbP 6.9
  idnK 6.8
  yfcX 6.7
  ascG 6.6
  rpsO 6.6
  yddM 6.6
  ydhL 6.6
  yieP 6.6
  yicI 6.6
  yicM 6.6
  yfeS 6.6
  ulaG 6.5
  ivbL 6.5
  ybiM 6.5
  ymgH 6.5
  yhjX 6.5
  dhaR 6.4
  yfeH 6.4
  hchA 6.3
  ycdU 6.3
  yjgB 6.3
  rpsT 6.3
  yneF 6.2
  yiiM 6.2
  rzoD 6.2
  potG 6.1
  baeR 6.1
  yedW 6.1
  ygcS 6.0
  ydbJ 6.0
  bglJ 6.0
  cld 6.0
  ybfE 6.0
  ybfA 6.0
  mutY 6.0
  ubiG 6.0
  glxK 5.9
  ynfD 5.9
  ybbN 5.9
  aaeX 5.9
  tnaB 5.9
  yghO 5.9
  rtcA 5.8
  csiE 5.8
  nuoC 5.8
  ypfJ 5.7
  elaD 5.7
  yjiK 5.7
  ydhM 5.6
  wcaI 5.6
  yjiP 5.6
  ypeB 5.6
  yceG 5.6
  ynfC 5.5
  ybjS 5.5
  ydfW 5.5
  ycfS 5.5
  yibI 5.5
  ylbH 5.4
  tdcR 5.4
  hypE 5.4
  pqqL 5.4
  mcrC 5.4
  yibK 5.4
  yjiA 5.3
  pcnB 5.3
  setC 5.3
  ybfL 5.2
  leuB 5.2
  yihS 5.1
  bolA 5.1
  ychM 5.1
  yfdN 5.1
  yeeY 5.0
  yjhT 5.0
  yidX 5.0
  hybG 5.0
  gatZ 5.0
  fhiA 5.0
  glnD 5.0
  mcrB 4.9
  ycgJ 4.9
  yliB 4.9
  cspH 4.9
  yeeP 4.9
  yfdY 4.9
  abgT 4.9
  yedQ 4.8
  eutP 4.8
  wcaC 4.8
  etp 4.8
  yjiT 4.8
  recQ 4.7
  gldA 4.7
  narY 4.7
  kgtP 4.7
  dkgB 4.7
  epd 4.7
  hpt 4.7
  yciB 4.7
  abrB 4.6
  yhiM 4.6
  hipB 4.6
  yjdC 4.6
  aceK 4.6
  ycdH 4.6
  rpsU 4.6
  dcuC 4.6
  tufA 4.6
  treB 4.6
  ydfE 4.6
  hycA 4.6
  ygeO 4.5
  oxyR 4.5
  yrfD 4.5
  asr 4.5
  ydeQ 4.5
  ycbW 4.5
  hycB 4.5
  yncH 4.4
  ybdH 4.4
  ykfC 4.4
  gspG 4.4
  znuA 4.4
  tsr 4.4
  ycgZ 4.3
  barA 4.3
  intE 4.3
  glcF 4.3
  yebN 4.3
  ymfS 4.3
  cysB 4.2
  yegR 4.2
  ygdI 4.2
  fkpA 4.2
  yedM 4.2
  aphA 4.1
  yebA 4.1
  trpE 4.1
  yeeJ 4.1
  hdfR 4.1
  ymfP 4.0
  sucD 4.0
  dmsD 4.0
  ypfH 4.0
  yjiZ 4.0
  tfaD 4.0
  yedJ 4.0
  nikR 3.9
  ilvH 3.9
  malF 3.9
  yqhC 3.9
  ybiW 3.9
  ycdT 3.8
  hipA 3.8
  mhpD 3.8
  flgM 3.8
  ykgB 3.8
  yfjZ 3.8
  fliS 3.8
  yeeA 3.8
  yoeA 3.7
  yjdI 3.7
  gss 3.6
  hyfJ 3.6
  dnaG 3.6
  hycD 3.6
  moeA 3.6
  yajL 3.6
  norR 3.5
  lipB 3.5
  ytfK 3.5
  yfdE 3.5
  yidJ 3.5
  ydcE 3.5
  fliD 3.5
  yjeM 3.5
  yphC 3.4
  fic 3.4
  prmB 3.4
  rna 3.4
  yghA 3.4
  acrE 3.4
  prfC 3.3
  pinR 3.3
  djlC 3.3
  mhpB 3.3
  ulaE 3.3
  ycgY 3.3
  ybeA 3.3
  citA 3.3
  hisH 3.2
  napD 3.2
  folX 3.2
  yeeL 3.2
  kdpB 3.2
  prfB 3.2
  yaiF 3.2
  caiB 3.2
  guaD 3.2
  fimG 3.2
  ydcH 3.2
  serB 3.2
  yegX 3.1
  ansP 3.1
  yciI 3.1
  cvrA 3.1
  ykgE 3.1
  argK 3.1
  yejO 3.1
  ygcL 3.1
  yigL 3.0
  yhdU 3.0
  hcaR 3.0
  fliI 3.0
  hokE 3.0
  trmH 3.0
     Differential ions  
   id name formula mz mod AUC Z-score Z-score AUC Weighted   C01151  D-Ribose 1,5-bisphosphate C5H12O11P2 548.8884 .(H2PO4Na)2-H(+) 0.000 -3.895 -0.000
     KEGG pathway by CLR  
none  COG enrichment  
   Pathway_MS pvalue_MS qvalue_MS  Dioxin degradation 0.005 0.4487
     Predicted metabolites from CLR  
   Predicted metabolites Pvalue Overlap with hits  bis-molybdenum cofactor 5e-05 0.0000
  tungsten bispterin cofactor 5e-05 0.0000
  Acetol 0.0005 0.0000
  molybdenum cofactor 0.0005 0.0000
  Methylglyoxal 0.002 0.0000
  D-Gluconate 0.003 0.0000
  Guanine 0.003 0.0000
    
 
