# Supplementary material for: Genomewide landscape of gene–metabolome associations in Escherichia coli
Source: Mol Syst Biol. 2017 Jan 16;13(1):907. doi: 10.15252/msb.20167150 (PMC5293155; doi:10.15252/msb.20167150)
Supplement: Supplementary file 4 — Table EV3 [file MSB-13-907-s004.zip › details/data_yacL.html]

 
 
 yacL 
  yacL - details 
 
 
  CLR  
   Gene_matching CLR_index  ybjR 18.2
  idi 17.2
  uxuA 17.0
  ydhH 16.9
  yajC 16.2
  pyrB 16.0
  yjgD 15.7
  tap 15.2
  uvrA 15.1
  pyrE 15.0
  metN 14.9
  purD 14.6
  frvB 14.5
  uhpA 14.5
  yafY 14.0
  yghX 14.0
  purF 13.9
  apaH 13.4
  hyaE 12.9
  smpB 12.9
  napB 12.8
  xylA 12.7
  purR 12.6
  aroP 12.6
  yfaD 12.4
  paaB 12.4
  iap 12.3
  flgG 12.2
  ygeD 11.9
  fucA 11.7
  nadB 11.6
  norW 11.5
  fixX 11.5
  yghF 11.5
  gltA 11.3
  kefF 11.2
  oppF 11.0
  rbsB 10.8
  intD 10.8
  ygdD 10.6
  pyrF 10.6
  rnhB 10.5
  menA 10.5
  rluD 10.5
  arnT 10.4
  dcd 10.3
  argA 10.1
  yebR 10.1
  nudH 10.0
  solA 10.0
  yeiM 9.9
  bioF 9.8
  ybjM 9.8
  ygiS 9.7
  alpA 9.7
  purL 9.5
  recX 9.5
  fepE 9.5
  gadA 9.4
  yncM 9.3
  serA 9.2
  malG 9.2
  ppiD 9.1
  ydgC 9.1
  yidZ 9.0
  azoR 9.0
  ddlA 8.9
  treR 8.9
  yehW 8.9
  purM 8.8
  recB 8.8
  narW 8.8
  selA 8.8
  ygdQ 8.7
  cobT 8.5
  yfaL 8.5
  ymgE 8.4
  yeiH 8.4
  proC 8.3
  ygbJ 8.3
  yccM 8.1
  ybbY 8.0
  tldD 8.0
  ycdK 7.9
  purN 7.9
  narL 7.8
  yhiN 7.8
  wcaL 7.8
  srlR 7.8
  yaaX 7.6
  ycgR 7.6
  allC 7.5
  htrG 7.4
  ydcA 7.4
  oxc 7.3
  yeaL 7.3
  ptsI 7.3
  chpB 7.1
  ycjT 7.0
  idnR 6.9
  poxA 6.8
  aroA 6.8
  flgJ 6.8
  rnk 6.8
  agaS 6.6
  yafP 6.6
  betA 6.6
  ygaR 6.6
  yfbV 6.6
  sohB 6.5
  yegL 6.5
  ydaL 6.4
  norV 6.4
  csdA 6.4
  ymfL 6.4
  ybgH 6.4
  ycdX 6.3
  yqjA 6.3
  ydjM 6.3
  yggH 6.2
  lsrF 6.2
  yohL 6.2
  ydcR 6.2
  yliC 6.2
  rfe 6.2
  uxaC 6.2
  vsr 6.2
  lacY 6.1
  ptsG 6.1
  ulaF 6.0
  ybjL 5.9
  ybaP 5.9
  ptsH 5.8
  iscA 5.8
  sapC 5.8
  serC 5.8
  fiu 5.8
  ygeF 5.7
  sgbH 5.7
  hcaD 5.7
  allA 5.7
  yagA 5.7
  yehS 5.6
  ynaE 5.6
  mpaA 5.6
  ycfQ 5.6
  hns 5.5
  yaaH 5.5
  yiaF 5.5
  yfbL 5.4
  truD 5.4
  yeaS 5.4
  trpB 5.3
  yadE 5.3
  dicC 5.3
  nagB 5.3
  cyoC 5.3
  lplA 5.2
  ynbA 5.2
  yfdI 5.1
  ygbL 5.1
  fis 5.1
  ychP 5.1
  motB 5.1
  appY 5.0
  yecE 5.0
  yfdZ 5.0
  ltaE 5.0
  rhaR 4.9
  yibD 4.9
  yrbL 4.9
  yafQ 4.9
  pppA 4.9
  ydfD 4.9
  fucO 4.9
  fdhF 4.9
  ybfH 4.8
  ybfQ 4.8
  katE 4.8
  ymcB 4.8
  hisF 4.8
  nuoA 4.8
  uup 4.7
  ccmB 4.7
  ccmE 4.7
  ybdK 4.6
  gcvH 4.6
  ybbB 4.6
  ygiF 4.5
  yfhG 4.5
  paaA 4.5
  eutC 4.5
  dmsC 4.5
  yafV 4.5
  rfbX 4.4
  rcsC 4.4
  emrB 4.4
  csgG 4.4
  yfbN 4.4
  yafM 4.4
  yghJ 4.3
  yfjK 4.3
  kdgR 4.3
  btuC 4.3
  osmC 4.3
  rhlB 4.2
  yeeE 4.2
  bipA 4.2
  yphF 4.2
  ybhE 4.2
  rcsA 4.2
  ilvN 4.2
  yhbP 4.2
  rssB 4.2
  appA 4.1
  yqaE 4.1
  ycbK 4.1
  ykgJ 4.1
  yahL 4.1
  yjjZ 4.1
  yfjQ 4.0
  ybjX 4.0
  yehZ 4.0
  ynaI 4.0
  fadB 4.0
  feoA 4.0
  yaeJ 4.0
  ydiF 4.0
  ycaO 4.0
  nuoN 4.0
  tesA 4.0
  dinG 3.9
  mdoB 3.9
  fadA 3.9
  yehQ 3.9
  yfiD 3.8
  kdpB 3.8
  nrfD 3.8
  eutT 3.8
  ypdD 3.8
  yagW 3.8
  ycdN 3.8
  fdoG 3.8
  nuoK 3.8
  artI 3.8
  yedA 3.8
  yfcH 3.8
  yejH 3.8
  puuR 3.7
  mhpF 3.7
  yghU 3.7
  ypdC 3.6
  paaG 3.6
  fepC 3.6
  yfjL 3.6
  mfd 3.6
  ldcC 3.6
  ddpX 3.6
  ydgT 3.5
  ygcP 3.5
  cadC 3.5
  yfcC 3.5
  yahI 3.5
  ygaM 3.5
  rffE 3.4
  yhhN 3.4
  yddH 3.4
  ugpQ 3.4
  trpA 3.4
  talB 3.4
  cheW 3.4
  speF 3.4
  yhbY 3.4
  talA 3.4
  rpiB 3.4
  pncA 3.4
  hdhA 3.4
  cheY 3.3
  ybeL 3.3
  yebF 3.3
  mmuM 3.3
  rpe 3.3
  artP 3.3
  yfgC 3.3
  holE 3.3
  ybjC 3.3
  malZ 3.3
  ydgG 3.3
  dicB 3.2
  yciT 3.2
  yjhB 3.2
  yagQ 3.2
  lsrB 3.2
  potI 3.2
  yggF 3.2
  ydeU 3.2
  yhgA 3.1
  ychH 3.1
  yciS 3.1
  ycjP 3.1
  ydfQ 3.1
  yhaH 3.1
  yciC 3.1
  yfbP 3.1
  gpmI 3.1
  cpxR 3.1
  yccV 3.1
  yadS 3.1
  oppD 3.1
  yafL 3.1
  yqjF 3.1
  clpX 3.1
  yddE 3.1
  yhiL 3.0
  ybiN 3.0
  manX 3.0
  yiaM 3.0
  ugd 3.0
  yedV 3.0
  yieE 3.0
  ycbS 3.0
  hslV 3.0
  hflK 3.0
  yeaR 3.0
  yniA 3.0
  yoaG 3.0
  clpS 3.0
     Differential ions  
   id name formula mz mod AUC Z-score Z-score AUC Weighted   C01909  Dethiobiotin C10H18N2O3 216.1427 [+1].H(+) 0.871 8.805 7.667
   C16565  N-3-aminopropyl-1,5-diaminopentane C8H21N3 280.1381 .H2PO4Na.H(+) 0.799 8.289 6.619
   C01909  Dethiobiotin C10H18N2O3 237.1223 .H/Na.H(+) 0.828 7.901 6.545
   C01909  Dethiobiotin C10H18N2O3 237.1223 .Na(+) 0.828 7.901 6.545
   C05775  N1-(alpha-D-ribosyl)-5,6-dimethylbenzimidazole C14H18N2O4 280.1381 [+1].H(+) 0.752 8.289 6.236
   C01909  Dethiobiotin C10H18N2O3 215.1390 .H(+) 0.621 9.118 5.665
   C15699  gamma-glutamyl-putrescine C9H19O3N3 354.0842 .H2PO4K.H(+) 0.980 4.652 4.557
   C01909  Dethiobiotin C10H18N2O3 217.1473 [+2].H(+) 0.896 5.007 4.486
   C06156  D-Glucosamine 1-phosphate C6H14NO8P 180.0875 -HPO3.H(+) 0.993 4.310 4.279
   C05629  Phenylpropanoate C9H10O2 271.0372 .H2PO4Na.H(+) 0.730 5.794 4.230
   C00137  myo-Inositol C6H12O6 204.0564 [+1].Na(+) 0.751 4.904 3.685
   C15699  gamma-glutamyl-putrescine C9H19O3N3 219.1488 [+1].H(+) 0.675 5.412 3.655
   C00989  gamma-hydroxybutyrate C4H8O3 127.0380 .H/Na.H(+) 0.802 4.431 3.554
   C00159  D-Mannose C6H12O6 205.0581 [+2].Na(+) 0.895 3.961 3.546
   C04593  methylisocitrate C7H10O7 163.0601 -CO2.H(+) 0.836 4.202 3.513
   C00159  D-Mannose C6H12O6 203.0529 .H/Na.H(+) 0.768 4.515 3.470
   C00178  Thymine C5H6N2O2 344.9897 .(H2PO4)2NaH.H(+) 0.984 3.491 3.436
   C01909  Dethiobiotin C10H18N2O3 335.1077 .H2PO4Na.H(+) 0.718 4.742 3.403
   C00129  Isopentenyl diphosphate C5H12O7P2 464.9480 .(H2PO4)2NaH.H(+) 0.861 3.946 3.398
   C00235  Dimethylallyl diphosphate C5H12O7P2 464.9480 .(H2PO4)2NaH.H(+) 0.861 3.946 3.398
   C00137  myo-Inositol C6H12O6 323.0108 .HPO4Na2.H(+) 0.883 3.807 3.363
   C05775  N1-(alpha-D-ribosyl)-5,6-dimethylbenzimidazole C14H18N2O4 281.1411 [+2].H(+) 0.748 4.466 3.341
   C00407  L-Isoleucine C6H13NO2 133.1052 [+1].H(+) 0.747 4.444 3.321
   C00534  Pyridoxamine C8H12N2O2 169.0949 .H(+) 0.717 4.609 3.307
   C00437  N2-Acetyl-L-ornithine C7H14N2O3 311.0371 .H2PO4K.H(+) 0.695 4.759 3.306
   C11514  E-3-carboxy-2-pentenedioate 6-methyl ester C7H8O6 428.9521 .(H2PO4Na)2.H(+) 0.866 3.808 3.298
   C01097  D-Tagatose 6-phosphate C6H13O9P 163.0601 -H3PO4.H(+) 0.782 4.202 3.286
   C00079  L-Phenylalanine C9H11NO2 167.0902 [+1].H(+) 0.781 4.172 3.260
   C00031  D-Glucose C6H12O6 204.0564 [+1].Na(+) 0.661 4.904 3.239
   C00725  Lipoate C8H14O2S2 163.0601 -CO2.H(+) 0.765 4.202 3.214
   C00601  Phenylacetaldehyde C8H8O 103.0541 -H2O.H(+) 0.706 4.536 3.201
   C00270  N-Acetylneuraminate C11H19NO9 332.0963 .H/Na.H(+) 0.651 4.898 3.188
   C00270  N-Acetylneuraminate C11H19NO9 332.0963 .Na(+) 0.651 4.898 3.188
   C15998  L-methionine-R-sulfoxide C5H11NO3S 166.0509 .H(+) 0.712 4.466 3.178
   C00079  L-Phenylalanine C9H11NO2 188.0692 .H/Na.H(+) 0.626 5.012 3.137
   C00079  L-Phenylalanine C9H11NO2 188.0692 .Na(+) 0.626 5.012 3.137
   C00209  Oxalate C2H2O4 360.8509 .(H2PO4K)2-H(+) 0.841 3.711 3.121
   C00137  myo-Inositol C6H12O6 203.0529 .H/Na.H(+) 0.689 4.515 3.111
   C01909  Dethiobiotin C10H18N2O3 197.1281 -H2O.H(+) 0.790 3.933 3.105
   C00423  trans-Cinnamate C9H8O2 131.0502 -H2O.H(+) 0.761 4.052 3.085
   C01449  7-aminomethyl-7-deazaguanine C7H9N5O 181.0918 [+1].H(+) 0.883 3.484 3.077
   Glycerophosphoserine  Glycerophosphoserine C6H14NO8P 180.0875 -HPO3.H(+) 0.703 4.310 3.030
   C00855  D-Methionine C5H11NO2S 133.0323 -NH3.H(+) 0.751 4.025 3.024
   C01487  D-Allose C6H12O6 205.0581 [+2].Na(+) 0.762 3.961 3.020
   C01487  D-Allose C6H12O6 204.0564 [+1].Na(+) 0.613 4.904 3.006
   C00079  L-Phenylalanine C9H11NO2 166.0861 .H(+) 0.610 4.915 3.000
   C08362  Hexadecenoate (n-C16:1) C16H30O2 375.1932 .H2PO4Na.H(+) 0.642 4.640 2.979
   ferroxamine minus Fe(3)  ferroxamine minus Fe(3) C25H48N6O8 583.3222 .H/Na.H(+) 0.815 3.643 2.971
   C00179  Agmatine C5H14N4 251.0840 .H2PO4Na.H(+) 0.805 3.688 2.968
   C00095  D-Fructose C6H12O6 203.0529 .H/Na.H(+) 0.655 4.515 2.959
   C00534  Pyridoxamine C8H12N2O2 191.0788 .H/Na.H(+) 0.799 3.701 2.956
   C00534  Pyridoxamine C8H12N2O2 191.0788 .Na(+) 0.799 3.701 2.956
   C00855  D-Methionine C5H11NO2S 150.0588 .H(+) 0.769 3.840 2.951
   branching glycogen  branching glycogen C6H10O5 163.0601 .H(+) 0.700 4.202 2.941
   C00636  D-Mannose 1-phosphate C6H13O9P 163.0601 -H3PO4.H(+) 0.695 4.202 2.922
   C03406  N(omega)-(L-Arginino)succinate C10H18N4O6 562.9934 .(H2PO4K)2.H(+) 0.811 3.549 2.877
   C02225  2-Methylcitrate C7H10O7 163.0601 -CO2.H(+) 0.672 4.202 2.824
   C18096  Allulose 6-phosphate C6H13O9P 163.0601 -H3PO4.H(+) 0.671 4.202 2.821
   C15996  7-cyano-7-carbaguanine C7H5N5O 311.9879 .H2PO4K.H(+) 0.811 3.465 2.809
   C00275  D-Mannose 6-phosphate C6H13O9P 163.0601 -H3PO4.H(+) 0.658 4.202 2.765
   C00073  L-Methionine C5H11NO2S 133.0323 -NH3.H(+) 0.678 4.025 2.728
   C00352  D-Glucosamine 6-phosphate C6H14NO8P 180.0875 -HPO3.H(+) 0.627 4.310 2.702
   C04225  cis-2-Methylaconitate C7H8O6 428.9521 .(H2PO4Na)2.H(+) 0.706 3.808 2.688
   C00407  L-Isoleucine C6H13NO2 154.0841 .Na(+) 0.613 4.341 2.659
   C00407  L-Isoleucine C6H13NO2 154.0841 .H/Na.H(+) 0.613 4.341 2.659
   C00263  L-Homoserine C4H9NO3 142.0477 .H/Na.H(+) 0.715 3.711 2.652
   C00937  D-Lactaldehyde C3H6O2 98.0310 [+1].Na(+) 0.751 3.520 2.645
   C00109  2-Oxobutanoate C4H6O3 85.0284 -H2O.H(+) 0.661 3.939 2.604
   C00182  glycogen C6H10O5 163.0601 .H(+) 0.617 4.202 2.591
   C05235  Acetol C3H6O2 98.0310 [+1].Na(+) 0.735 3.520 2.588
   C02130  Acetyl-maltose C14H24O12 386.1308 [+1].H(+) 0.658 3.882 2.556
   C00163  Propionate (n-C3:0) C3H6O2 97.0277 .H/Na.H(+) 0.606 4.112 2.493
   C00577  D-Glyceraldehyde C3H6O3 360.8865 .(H2PO4K)2-H(+) 0.655 3.794 2.485
   C15930  L-Galactonate C6H12O7 333.0123 .H2PO4K.H(+) 0.621 3.922 2.437
   C00164  Acetoacetate C4H6O3 85.0284 -H2O.H(+) 0.608 3.939 2.394
   C00163  Propionate (n-C3:0) C3H6O2 98.0310 [+1].Na(+) 0.679 3.520 2.391
   C05629  Phenylpropanoate C9H10O2 293.0147 .HPO4Na2.H(+) 0.630 3.786 2.385
   C08362  Hexadecenoate (n-C16:1) C16H30O2 271.2288 +OH(-) 0.633 3.753 2.377
   C00073  L-Methionine C5H11NO2S 150.0588 .H(+) 0.615 3.840 2.362
   C00424  L-Lactaldehyde C3H6O2 98.0310 [+1].Na(+) 0.659 3.520 2.319
   C00624  N-Acetyl-L-glutamate C7H11NO5 191.0788 [+1].H(+) 0.612 3.701 2.265
   C00879  D-Galactarate C6H10O8 233.0354 .H/Na.H(+) 0.633 3.570 2.261
   C00879  D-Galactarate C6H10O8 233.0354 .Na(+) 0.633 3.570 2.261
   C00062  L-Arginine C6H14N4O2 175.1189 .H(+) 0.649 3.480 2.258
   C00327  L-Citrulline C6H13N3O3 312.0406 .H2PO4K.H(+) 0.602 3.565 2.145
   C00015  UDP C9H14N2O12P2 442.9669 .H/K.H(+) 0.607 3.531 2.144
   C00364  dTMP C10H15N2O8P 443.0363 .H2PO4Na.H(+) 0.594 5.091 0.000
   C05235  Acetol C3H6O2 97.0277 .H/Na.H(+) 0.594 4.112 0.000
   C00123  L-Leucine C6H13NO2 133.1052 [+1].H(+) 0.594 4.444 0.000
   C05519  L-Allo-threonine C4H9NO3 142.0477 .H/Na.H(+) 0.591 3.711 0.000
   C00137  myo-Inositol C6H12O6 205.0581 [+2].Na(+) 0.591 3.961 0.000
   C00232  Succinic semialdehyde C4H6O3 85.0284 -H2O.H(+) 0.589 3.939 0.000
   C00062  L-Arginine C6H14N4O2 197.1003 .Na(+) 0.584 4.266 0.000
   C00062  L-Arginine C6H14N4O2 197.1003 .H/Na.H(+) 0.584 4.266 0.000
   C00188  L-Threonine C4H9NO3 142.0477 .H/Na.H(+) 0.584 3.711 0.000
   C00124  D-Galactose C6H12O6 204.0564 [+1].Na(+) 0.578 4.904 0.000
   C00446  alpha-D-Galactose 1-phosphate C6H13O9P 163.0601 -H3PO4.H(+) 0.577 4.202 0.000
   C01672  1,5-Diaminopentane C5H14N2 86.0967 -NH3.H(+) 0.575 4.030 0.000
   C00880  D-Galactonate C6H12O7 333.0123 .H2PO4K.H(+) 0.574 3.922 0.000
   octadecenoate (n-C18:1)  octadecenoate (n-C18:1) C18H34O2 419.1968 .H2PO4K.H(+) 0.573 5.182 0.000
   C00186  L-Lactate C3H6O3 360.8865 .(H2PO4K)2-H(+) 0.573 3.794 0.000
   C00124  D-Galactose C6H12O6 203.0529 .H/Na.H(+) 0.572 4.515 0.000
   C00124  D-Galactose C6H12O6 205.0581 [+2].Na(+) 0.570 3.961 0.000
   C00031  D-Glucose C6H12O6 203.0529 .H/Na.H(+) 0.568 4.515 0.000
   C00670  sn-Glycero-3-phosphocholine C8H20NO6P 280.0942 .H/Na.H(+) 0.567 4.993 0.000
   C00670  sn-Glycero-3-phosphocholine C8H20NO6P 280.0942 .Na(+) 0.567 4.993 0.000
   C00134  Putrescine C4H12N2 72.0809 -NH3.H(+) 0.567 3.521 0.000
   C00159  D-Mannose C6H12O6 204.0564 [+1].Na(+) 0.566 4.904 0.000
   C00937  D-Lactaldehyde C3H6O2 97.0277 .H/Na.H(+) 0.565 4.112 0.000
   C06006  (S)-2-Aceto-2-hydroxybutanoate C6H10O4 289.0105 .HPO4Na2.H(+) 0.564 3.606 0.000
   C00256  D-Lactate C3H6O3 360.8865 .(H2PO4K)2-H(+) 0.563 3.794 0.000
   C00095  D-Fructose C6H12O6 323.0108 .HPO4Na2.H(+) 0.563 3.807 0.000
   C00047  L-Lysine C6H14N2O2 169.0949 .Na(+) 0.562 4.609 0.000
   C00047  L-Lysine C6H14N2O2 169.0949 .H/Na.H(+) 0.562 4.609 0.000
   C01909  Dethiobiotin C10H18N2O3 357.0776 .HPO4Na2.H(+) 0.561 8.775 0.000
   C15700  gamma-glutamyl-gamma-butyraldehyde C9H16O4N2 219.1293 [+2].H(+) 0.561 6.102 0.000
   C00514  D-Mannonate C6H12O7 333.0123 .H2PO4K.H(+) 0.558 3.922 0.000
   C00424  L-Lactaldehyde C3H6O2 97.0277 .H/Na.H(+) 0.557 4.112 0.000
   C00123  L-Leucine C6H13NO2 154.0841 .Na(+) 0.554 4.341 0.000
   C00123  L-Leucine C6H13NO2 154.0841 .H/Na.H(+) 0.554 4.341 0.000
   C00184  Dihydroxyacetone C3H6O3 360.8865 .(H2PO4K)2-H(+) 0.553 3.794 0.000
   C02730  o-Succinylbenzoate C11H10O5 225.0654 [+2].H(+) 0.553 4.788 0.000
   C00114  Choline C5H13NO 86.0967 -H2O.H(+) 0.552 4.030 0.000
   C00257  D-Gluconate C6H12O7 333.0123 .H2PO4K.H(+) 0.551 3.922 0.000
   C04114  crotonobetaine C7H13NO2 166.0861 .H/Na.H(+) 0.550 4.915 0.000
   C02962  D-Allose 6-phosphate C6H13O9P 163.0601 -H3PO4.H(+) 0.549 4.202 0.000
   C00092  D-Glucose 6-phosphate C6H13O9P 163.0601 -H3PO4.H(+) 0.548 4.202 0.000
   C00152  L-Asparagine C4H8N2O3 133.0606 .H(+) 0.545 3.527 0.000
   C00021  S-Adenosyl-L-homocysteine C14H20N6O5S 386.1308 [+1].H(+) 0.545 3.882 0.000
   C12624  2-hydroxy-6-ketononatrienedioate C9H8O6 333.0123 .H2PO4Na.H(+) 0.544 3.922 0.000
   C00770  L-Idonate C6H12O7 333.0123 .H2PO4K.H(+) 0.534 3.922 0.000
   C00152  L-Asparagine C4H8N2O3 155.0428 .Na(+) 0.533 3.948 0.000
   C00152  L-Asparagine C4H8N2O3 155.0428 .H/Na.H(+) 0.533 3.948 0.000
   C00031  D-Glucose C6H12O6 323.0108 .HPO4Na2.H(+) 0.532 3.807 0.000
   C01487  D-Allose C6H12O6 323.0108 .HPO4Na2.H(+) 0.532 3.807 0.000
   C01449  7-aminomethyl-7-deazaguanine C7H9N5O 163.0601 -NH3.H(+) 0.532 4.202 0.000
   C00078  L-Tryptophan C11H12N2O2 205.0969 .H(+) 0.532 3.471 0.000
   C03082  4-Phospho-L-aspartate C4H8NO7P 333.9692 .H2PO4Na.H(+) 0.529 3.577 0.000
   C01013  3-Hydroxypropanoate C3H6O3 360.8865 .(H2PO4K)2-H(+) 0.527 3.794 0.000
   C00031  D-Glucose C6H12O6 205.0581 [+2].Na(+) 0.526 3.961 0.000
   C00148  L-Proline C5H9NO2 72.0809 -CO2.H(+) 0.525 3.521 0.000
   C00047  L-Lysine C6H14N2O2 129.1014 -H2O.H(+) 0.525 3.821 0.000
   C00534  Pyridoxamine C8H12N2O2 311.0371 .HPO4Na2.H(+) 0.523 4.759 0.000
   C00407  L-Isoleucine C6H13NO2 132.1024 .H(+) 0.521 4.437 0.000
   C00062  L-Arginine C6H14N4O2 176.1217 [+1].H(+) 0.520 3.951 0.000
   C00817  D-Altronate C6H12O7 333.0123 .H2PO4K.H(+) 0.519 3.922 0.000
   C00095  D-Fructose C6H12O6 205.0581 [+2].Na(+) 0.518 3.961 0.000
   C00095  D-Fructose C6H12O6 204.0564 [+1].Na(+) 0.512 4.904 0.000
   C00966  2-Dehydropantoate C6H10O4 289.0105 .HPO4Na2.H(+) 0.512 3.606 0.000
   C06424  tetradecanoate (n-C14:0) C14H28O2 229.2152 .H(+) 0.510 7.346 0.000
   C00818  D-Glucarate C6H10O8 233.0354 .H/Na.H(+) 0.509 3.570 0.000
   C00818  D-Glucarate C6H10O8 233.0354 .Na(+) 0.509 3.570 0.000
   C01487  D-Allose C6H12O6 203.0529 .H/Na.H(+) 0.509 4.515 0.000
   C00103  D-Glucose 1-phosphate C6H13O9P 163.0601 -H3PO4.H(+) 0.508 4.202 0.000
   C00064  L-Glutamine C5H10N2O3 289.0105 .HPO4Na2.H(+) 0.508 3.606 0.000
   C16565  N-3-aminopropyl-1,5-diaminopentane C8H21N3 400.0994 .(H2PO4Na)2.H(+) 0.506 3.475 0.000
   C00124  D-Galactose C6H12O6 323.0108 .HPO4Na2.H(+) 0.501 3.807 0.000
   C00214  Thymidine C10H14N2O5 243.0990 .H(+) 0.500 4.456 0.000
   C00123  L-Leucine C6H13NO2 132.1024 .H(+) 0.499 4.437 0.000
   C00493  Shikimate C7H10O5 176.0655 [+1].H(+) 0.498 4.815 0.000
   C00407  L-Isoleucine C6H13NO2 372.0197 .(H2PO4Na)2.H(+) 0.498 3.524 0.000
   C00123  L-Leucine C6H13NO2 372.0197 .(H2PO4Na)2.H(+) 0.495 3.524 0.000
   C05629  Phenylpropanoate C9H10O2 287.0113 .H2PO4K.H(+) 0.494 3.522 0.000
   C00078  L-Tryptophan C11H12N2O2 188.0692 -NH3.H(+) 0.493 5.012 0.000
   C00159  D-Mannose C6H12O6 323.0108 .HPO4Na2.H(+) 0.482 3.807 0.000
   C04294  4-Methyl-5-(2-hydroxyethyl)-thiazole C6H9NOS 146.0566 [+2].H(+) 0.481 4.071 0.000
   C00842  dTDPglucose C16H26N2O16P2 738.9675 .HPO4K2.H(+) 0.479 3.477 0.000
   C00085  D-Fructose 6-phosphate C6H13O9P 163.0601 -H3PO4.H(+) 0.478 4.202 0.000
   C04236  3-Carboxy-4-methyl-2-oxopentanoate C7H10O5 176.0655 [+1].H(+) 0.473 4.815 0.000
   C02989  L-Methionine Sulfoxide C5H11NO3S 166.0509 .H(+) 0.470 4.466 0.000
   C02976  D-Fructose 1-phosphate C6H13O9P 163.0601 -H3PO4.H(+) 0.457 4.202 0.000
   C16519  2-succinyl-5-enolpyruvyl-6-hydroxy-3-cyclohexene-1-carboxylate C14H16O9 562.9934 .(H2PO4)2KH.H(+) 0.451 3.549 0.000
   C01449  7-aminomethyl-7-deazaguanine C7H9N5O 180.0875 .H(+) 0.447 4.310 0.000
   C00596  2-Oxopent-4-enoate C5H6O3 97.0277 -H2O.H(+) 0.426 4.112 0.000
   C05775  N1-(alpha-D-ribosyl)-5,6-dimethylbenzimidazole C14H18N2O4 279.1339 .H(+) 0.422 7.206 0.000
   C00931  Porphobilinogen C10H14N2O4 227.1064 .H(+) 0.000 5.729 0.000
   C00931  Porphobilinogen C10H14N2O4 249.0894 .H/Na.H(+) 0.000 4.335 0.000
   C00931  Porphobilinogen C10H14N2O4 249.0894 .Na(+) 0.000 4.335 0.000
   C01177  1D-myo-Inositol 1-phosphate C6H13O9P 163.0601 -H3PO4.H(+) 0.000 4.202 0.000
   C01304  2,5-Diamino-6-(ribosylamino)-4-(3H)-pyrimidinone 5'-phosphate C9H16N5O8P 354.0842 .H(+) 0.000 4.652 0.000
   C03340  2,3-Dihydrodipicolinate C7H7NO4 311.9879 .HPO4Na2.H(+) 0.000 3.465 0.000
   C04454  5-Amino-6-(5'-phosphoribitylamino)uracil C9H17N4O9P 357.0776 .H(+) 0.000 8.775 0.000
   C11436  2-phospho-4-(cytidine 5'-diphospho)-2-C-methyl-D-erythritol C14H26N3O17P3 873.9268 .(H2PO4K)2.H(+) 0.000 3.472 0.000
   Fe(III)hydoxamate, unloaded  Fe(III)hydoxamate, unloaded C9H21O6N3 270.1596 [+2].H(+) 0.000 3.879 0.000
     KEGG pathway by CLR  
   Pathway_ion pvalue_ion qvalue_ion  Lipoic acid metabolism 0 0.0000
  Fructose and mannose metabolism 5e-07 0.0000
  Aminoacyl-tRNA biosynthesis 6e-07 0.0000
  Streptomycin biosynthesis 6e-06 0.0001
  Propanoate metabolism 2e-05 0.0002
  Valine, leucine and isoleucine degradation 2e-05 0.0002
  Amino sugar and nucleotide sugar metabolism 8e-05 0.0005
  Lysine degradation 0.0002 0.0011
  Nicotinate and nicotinamide metabolism 0.0004 0.0019
  Phosphotransferase system (PTS) 0.0009 0.0041
  Phenylalanine metabolism 0.001 0.0053
  Starch and sucrose metabolism 0.001 0.0050
  Inositol phosphate metabolism 0.002 0.0060
  Chlorocyclohexane and chlorobenzene degradation 0.002 0.0062
  Pyruvate metabolism 0.002 0.0058
  Glycine, serine and threonine metabolism 0.003 0.0101
  ABC transporters 0.003 0.0096
  Biotin metabolism 0.004 0.0097
  Galactose metabolism 0.004 0.0118
  Lysine biosynthesis 0.006 0.0161
  Polyketide sugar unit biosynthesis 0.007 0.0158
  Ethylbenzene degradation 0.009 0.0201
     COG enrichment  
   Pathway_MS pvalue_MS qvalue_MS  Ethylbenzene degradation 0.0006 0.0203
  Geraniol degradation 0.0007 0.0150
  Pentose and glucuronate interconversions 0.002 0.0287
  Caprolactam degradation 0.002 0.0304
  Biosynthesis of secondary metabolites 0.003 0.0277
  Bacterial chemotaxis 0.005 0.0486
  Limonene and pinene degradation 0.006 0.0445
  Biosynthesis of unsaturated fatty acids 0.006 0.0396
  Fatty acid metabolism 0.007 0.0427
  Inositol phosphate metabolism 0.007 0.0432
  Lysine degradation 0.007 0.0398
  Tryptophan metabolism 0.007 0.0368
  Glycine, serine and threonine metabolism 0.01 0.0444
     Predicted metabolites from CLR  
   Predicted metabolites Pvalue Overlap with hits  3-Phosphohydroxypyruvate 0 0.0000
  2-(Formamido)-N1-(5-phospho-D-ribosyl)acetamidine 0 0.0000
  Nitrous oxide 0 0.0000
  Orotidine 5'-phosphate 0 0.0000
  5-Phospho-beta-D-ribosylamine 0 0.0000
  UDP-N-acetyl-D-glucosamine 0 0.0000
  L-Lactaldehyde 7e-05 1.0000
  Choline 0.0003 1.0000
  D-Glucosamine 6-phosphate 0.0006 1.0000
  Ethanolamine 0.0008 0.0000
  L-Fuculose 1-phosphate 0.0008 0.0000
  N2-Formyl-N1-(5-phospho-D-ribosyl)glycinamide 0.0008 0.0000
  N1-(5-Phospho-D-ribosyl)glycinamide 0.0008 0.0000
  Nicotinate 0.0008 0.0000
  (-)-Ureidoglycolate 0.0008 0.0000
  N-acetylmuramate 6-phosphate 0.0009 0.0000
  Ring 1,2-epoxyphenylacetyl-CoA 0.0009 0.0000
  L-Tryptophan 0.001 1.0000
  D-Fructose 6-phosphate 0.001 1.0000
  N-Acetyl-D-mannosamine 6-phosphate 0.002 0.0000
  3-Oxodecanoyl-CoA 0.003 0.0000
  3-Oxododecanoyl-CoA 0.003 0.0000
  3-Oxohexanoyl-CoA 0.003 0.0000
  3-Oxohexadecanoyl-CoA 0.003 0.0000
  3-Oxooctanoyl-CoA 0.003 0.0000
  3-Oxooctadecanoyl-CoA 0.003 0.0000
  3-Oxotetradecanoyl-CoA 0.003 0.0000
  3-Phospho-D-glycerate 0.003 0.0000
  Maltose 6'-phosphate 0.003 0.0000
  2(alpha-D-Mannosyl-6-phosphate)-D-glycerate 0.003 0.0000
  D-Mannitol 1-phosphate 0.003 0.0000
  Nitric oxide 0.003 0.0000
  Sucrose 6-phosphate 0.003 0.0000
  C'-(3-Indolyl)-glycerol 3-phosphate 0.007 0.0000
  Arbutin 6-phosphate 0.007 0.0000
  D-Fructose 1-phosphate 0.007 1.0000
  Galactitol 1-phosphate 0.007 0.0000
  L-alanine-D-glutamate-meso-2,6-diaminoheptanedioate 0.009 0.0000
  N-Acetyl-D-glucosamine 6-phosphate 0.009 0.0000
  D-Mannose 6-phosphate 0.009 1.0000
    
 
