# Supplementary material for: Genomewide landscape of gene–metabolome associations in Escherichia coli
Source: Mol Syst Biol. 2017 Jan 16;13(1):907. doi: 10.15252/msb.20167150 (PMC5293155; doi:10.15252/msb.20167150)
Supplement: Supplementary file 4 — Table EV3 [file MSB-13-907-s004.zip › details/data_yadL.html]

 
 
 yadL 
  yadL - details 
 
 
  CLR  
   Gene_matching CLR_index  yfeX 16.1
  yeiJ 15.8
  yohK 14.8
  yobD 14.3
  ynhG 13.9
  gltB 13.4
  yebZ 13.3
  pnuC 13.3
  yafT 13.2
  uhpB 13.0
  ypfI 12.9
  cusC 12.8
  envR 12.3
  yebG 12.3
  potH 12.3
  yadK 12.2
  ypjB 12.2
  yeaK 11.7
  hofQ 11.5
  mdtC 11.4
  leuO 11.2
  clpA 11.2
  cbl 11.1
  rsxA 10.9
  tatE 10.6
  yccR 10.6
  ybbO 10.5
  ynfL 10.4
  yafQ 10.3
  ykfI 10.3
  deoA 10.0
  gltJ 10.0
  ydiO 10.0
  phnF 9.9
  ydhD 9.8
  ygaH 9.7
  argR 9.7
  gltL 9.5
  sfsA 9.4
  kdpA 9.4
  wza 9.4
  citT 9.3
  ydhR 9.0
  ygaC 9.0
  aer 8.9
  yegE 8.9
  ydfA 8.8
  ycfT 8.6
  cyoD 8.6
  tfaE 8.6
  iscS 8.6
  ydgD 8.5
  yaiS 8.3
  fadB 8.2
  yraH 8.2
  nudG 8.0
  ycdS 8.0
  ldcC 7.9
  yehK 7.9
  yfdK 7.8
  ppdD 7.8
  ybfP 7.6
  rbsK 7.6
  fdhE 7.6
  ihfA 7.6
  insO 7.4
  rhaT 7.3
  ybfM 7.3
  thiD 7.2
  yahE 7.1
  htpX 7.1
  nrdG 7.0
  yliF 7.0
  yfjN 6.7
  eutI 6.6
  hyaB 6.6
  yfcT 6.6
  ygaW 6.6
  ygiV 6.5
  ygeL 6.5
  astE 6.4
  ycjG 6.3
  yidZ 6.2
  yeiE 6.1
  slp 6.0
  malK 6.0
  hsdR 6.0
  yqgA 6.0
  ybhK 5.9
  ymcB 5.9
  fnr 5.8
  yiaT 5.8
  dgoK 5.7
  ygbE 5.7
  ysdC 5.7
  potE 5.7
  yqcC 5.7
  ydiS 5.6
  sdhC 5.6
  nhaR 5.6
  clpX 5.6
  sixA 5.5
  sdhD 5.4
  fdrA 5.4
  ypdI 5.4
  yfjQ 5.4
  metJ 5.4
  ydfH 5.4
  nrdE 5.3
  yejK 5.2
  btuD 5.2
  argP 5.2
  yeaC 5.2
  sfmD 5.1
  ompF 5.1
  yecR 5.1
  yehM 5.0
  yebO 5.0
  ybiR 4.9
  yaaI 4.9
  yebY 4.9
  csgD 4.9
  ccmG 4.8
  rbsR 4.8
  yhhA 4.8
  frwD 4.8
  ycfX 4.7
  htrE 4.7
  melA 4.7
  yfjV 4.6
  ygfY 4.6
  yhaH 4.6
  potB 4.6
  rcsD 4.6
  deoD 4.6
  ycjW 4.6
  ydhA 4.6
  acnB 4.5
  aas 4.5
  yfjK 4.5
  kdpC 4.5
  cyoB 4.4
  ybdO 4.4
  pstA 4.4
  yfbF 4.4
  mipA 4.4
  ydbC 4.3
  yjiE 4.3
  melR 4.3
  dusC 4.3
  ycdM 4.3
  ybiA 4.2
  yniA 4.2
  mhpR 4.2
  ddpF 4.2
  fadL 4.2
  uxuR 4.2
  yfhJ 4.1
  yadI 4.1
  exbD 4.0
  ydcY 4.0
  torC 4.0
  lsrB 4.0
  yaaX 3.9
  wcaL 3.9
  cobC 3.9
  bcsE 3.9
  yqfA 3.9
  ilvC 3.8
  nohA 3.8
  yhhF 3.8
  yebE 3.8
  gudP 3.8
  mdtF 3.7
  mdoD 3.6
  glnP 3.6
  sapA 3.6
  ydiT 3.6
  garL 3.5
  yiaM 3.5
  mrp 3.5
  yfiQ 3.5
  hisP 3.5
  ydfU 3.5
  hslV 3.5
  fadA 3.5
  ygjE 3.5
  mak 3.5
  ygaV 3.5
  fliN 3.5
  ydhW 3.5
  ompA 3.4
  sbp 3.4
  cpxA 3.4
  ykgD 3.4
  ypdB 3.4
  yfjL 3.4
  pflC 3.4
  nlpI 3.3
  yhgA 3.3
  frc 3.3
  ygbA 3.3
  yjbI 3.3
  alsC 3.3
  mglB 3.2
  yoaF 3.2
  cusA 3.2
  ddpD 3.2
  gnsA 3.2
  yccV 3.2
  ydfR 3.2
  yqgB 3.2
  yncE 3.2
  yciF 3.2
  nagE 3.2
  ydjE 3.2
  ydaY 3.1
  yfhG 3.1
  ynfA 3.1
  ydgT 3.1
  yedE 3.1
  ahpF 3.1
  yfdG 3.1
  katE 3.0
  ydjN 3.0
  betB 3.0
  lldD 3.0
  yacC 3.0
  yfiH 3.0
     Differential ions  
   id name formula mz mod AUC Z-score Z-score AUC Weighted   octadecenoate (n-C18:1)  octadecenoate (n-C18:1) C18H34O2 305.2492 .H/Na.H(+) 0.713 4.002 2.851
   octadecenoate (n-C18:1)  octadecenoate (n-C18:1) C18H34O2 305.2492 .Na(+) 0.713 4.002 2.851
   C05973  2-Acyl-sn-glycero-3-phosphoethanolamine (n-C16:0) C21H44NO7P1 492.2489 .H/K.H(+) 0.673 4.053 2.726
   C08362  Hexadecenoate (n-C16:1) C16H30O2 255.2316 .H(+) 0.744 3.505 2.607
   octadecenoate (n-C18:1)  octadecenoate (n-C18:1) C18H34O2 283.2669 .H(+) 0.561 4.374 0.000
   C08362  Hexadecenoate (n-C16:1) C16H30O2 277.2190 .H/Na.H(+) 0.544 5.610 0.000
   C01530  octadecanoate (n-C18:0) C18H36O2 323.2386 .H/K.H(+) 0.536 4.861 0.000
     KEGG pathway by CLR  
   Pathway_ion pvalue_ion qvalue_ion  Arachidonic acid metabolism 0 0.0000
  Fatty acid biosynthesis 0.0004 0.0144
  Biosynthesis of unsaturated fatty acids 0.002 0.0542
     COG enrichment  
   Pathway_MS pvalue_MS qvalue_MS  Sphingolipid metabolism 0.004 0.3266
  Geraniol degradation 0.004 0.1700
     Predicted metabolites from CLR  
   Predicted metabolites Pvalue Overlap with hits  3-Oxodecanoyl-CoA 0.0009 0.0000
  3-Oxododecanoyl-CoA 0.0009 0.0000
  3-Oxohexanoyl-CoA 0.0009 0.0000
  3-Oxohexadecanoyl-CoA 0.0009 0.0000
  3-Oxooctanoyl-CoA 0.0009 0.0000
  3-Oxooctadecanoyl-CoA 0.0009 0.0000
  3-Oxotetradecanoyl-CoA 0.0009 0.0000
  silver 0.0009 0.0000
  Cu+ 0.002 0.0000
  Acetoacetyl-CoA 0.007 0.0000
  Uracil 0.009 0.0000
  Deoxyuridine 0.01 0.0000
    
 
