# Supplementary material for: Genomewide landscape of gene–metabolome associations in Escherichia coli
Source: Mol Syst Biol. 2017 Jan 16;13(1):907. doi: 10.15252/msb.20167150 (PMC5293155; doi:10.15252/msb.20167150)
Supplement: Supplementary file 4 — Table EV3 [file MSB-13-907-s004.zip › details/data_yaeF.html]

 
 
 yaeF 
  yaeF - details 
 
 
  CLR  
   Gene_matching CLR_index  yhiK 12.9
  yhfU 11.5
  rbbA 11.2
  yjeI 10.9
  dgoD 10.1
  pspA 10.1
  ykgL 9.8
  emrD 9.5
  fecB 9.3
  yjiV 9.2
  yaeI 9.2
  dgoA 9.1
  cspI 9.1
  srlA 8.8
  yjgK 8.8
  fecD 8.5
  yhiQ 8.1
  gntR 8.0
  yafD 7.9
  yjbO 7.8
  tdcG 7.7
  yghS 7.5
  yhiO 7.4
  yjgJ 7.3
  yihV 7.3
  yiiT 7.2
  yjhH 7.0
  mhpE 6.9
  yhfK 6.8
  wzzE 6.8
  rho 6.8
  yhjQ 6.7
  hslR 6.6
  yhjH 6.6
  bcsA 6.5
  yghE 6.5
  rimI 6.4
  sthA 6.4
  yigI 6.3
  ogrK 6.2
  yjcQ 6.1
  yghQ 6.0
  ampC 6.0
  lysU 6.0
  ybbK 5.9
  rplI 5.9
  acrF 5.8
  nemA 5.8
  chpA 5.8
  ypjJ 5.7
  ygjO 5.6
  ykgK 5.5
  kdgT 5.5
  ygeM 5.5
  ytfT 5.5
  yjgN 5.3
  yqjC 5.3
  smpA 5.3
  gspI 5.3
  yeiG 5.3
  yjcB 5.3
  yjfO 5.2
  yggL 5.2
  bcsF 5.2
  yejF 5.1
  yigE 5.1
  rfaE 5.1
  ytfI 5.0
  yjgM 5.0
  yhdJ 5.0
  prmA 4.9
  ppiA 4.9
  intA 4.9
  yahJ 4.9
  trkD 4.9
  nrfB 4.9
  ccmA 4.9
  agaA 4.9
  fadE 4.8
  gspL 4.8
  ulaA 4.8
  ygjP 4.8
  ygfB 4.8
  yjiD 4.8
  yibQ 4.8
  hslO 4.8
  yjhA 4.8
  gph 4.7
  emrE 4.7
  yjiY 4.7
  tatC 4.7
  nmpC 4.7
  emrA 4.7
  yfgG 4.6
  zapA 4.6
  pspB 4.6
  ygiQ 4.6
  rhoL 4.6
  yihX 4.5
  tufB 4.5
  fimI 4.5
  xdhC 4.5
  yicN 4.4
  ugpC 4.4
  thiS 4.4
  wzb 4.4
  chbB 4.3
  yiaB 4.3
  glyS 4.3
  ygeW 4.3
  exo 4.3
  rpsF 4.3
  frlB 4.3
  yoeB 4.2
  ykgI 4.2
  yffI 4.1
  ampG 4.1
  marB 4.1
  yieM 4.1
  yadD 4.0
  ptsA 4.0
  yjiL 4.0
  yhjY 4.0
  tatB 4.0
  pspD 4.0
  emrY 3.9
  bcr 3.9
  yhdX 3.9
  yagV 3.9
  ygcM 3.9
  ucpA 3.9
  pheM 3.9
  wzc 3.9
  yddA 3.8
  rpmJ 3.8
  rhtB 3.8
  gntX 3.8
  folP 3.8
  ynfH 3.8
  tynA 3.8
  ligT 3.8
  nanT 3.8
  yhbX 3.8
  rsmC 3.8
  alx 3.8
  glpK 3.7
  arsB 3.7
  clcA 3.7
  surE 3.7
  rluA 3.7
  ydhX 3.7
  thiF 3.7
  yjjA 3.7
  frlC 3.7
  cspB 3.6
  yiaN 3.6
  torI 3.6
  yigG 3.6
  ytfR 3.6
  citX 3.6
  sgbU 3.6
  ygfQ 3.6
  yjjN 3.6
  glcE 3.6
  nadR 3.5
  yjbN 3.5
  yrfA 3.5
  cspA 3.5
  ygjG 3.5
  greB 3.5
  yabI 3.5
  csiR 3.4
  yedO 3.4
  tatA 3.4
  yghJ 3.4
  yafX 3.4
  yhfY 3.4
  ypdJ 3.4
  yghU 3.3
  dcrB 3.3
  metL 3.3
  ynfN 3.3
  rpmE 3.3
  argB 3.3
  yhjK 3.3
  kdgK 3.2
  yfgD 3.2
  dgoR 3.2
  btuE 3.2
  hdeB 3.2
  sbmA 3.2
  yiiE 3.2
  ydjA 3.1
  rusA 3.1
  rffT 3.1
  ibpB 3.1
  thrL 3.1
  yjgW 3.0
  yjbF 3.0
  adiA 3.0
  actP 3.0
  yncK 3.0
  phoH 3.0
  yecG 3.0
  yjjP 3.0
  yidQ 3.0
  ecnB 3.0
  yrhC 3.0
  yraR 3.0
  ampD 3.0
  sanA 3.0
     Differential ions  
   id name formula mz mod AUC Z-score Z-score AUC Weighted   C03089  5-Methylthio-D-ribose C6H12O4S 452.9228 .(H2PO4K)2.H(+) 0.675 3.861 2.607
     KEGG pathway by CLR  
   Pathway_ion pvalue_ion qvalue_ion  Arginine and proline metabolism 0 0.0000
     COG enrichment  
   Pathway_MS pvalue_MS qvalue_MS  Protein export 0.0003 0.0269
  Ribosome 0.0007 0.0316
  Bacterial secretion system 0.0007 0.0231
  Lysine biosynthesis 0.001 0.0354
  Arachidonic acid metabolism 0.003 0.0585
  Aminoacyl-tRNA biosynthesis 0.005 0.0793
     Predicted metabolites from CLR  
   Predicted metabolites Pvalue Overlap with hits  2-Dehydro-3-deoxy-D-gluconate 0.0002 0.0000
  N-Acetyl-D-glucosamine(anhydrous)N-Acetylmuramic acid 0.0002 0.0000
  N-Acetyl-D-glucosamine(anhydrous)N-Acetylmuramyl-tripeptide 0.0002 0.0000
  N-Acetyl-D-glucosamine(anhydrous)N-Acetylmuramyl-tetrapeptide 0.0002 0.0000
  Citrate 0.0009 0.0000
  dehydroglycine 0.001 0.0000
  Fe3+ 0.001 0.0000
  L-Homoserine 0.001 0.0000
  4-Methyl-5-(2-phosphoethyl)-thiazole 0.002 0.0000
  1-deoxy-D-xylulose 5-phosphate 0.003 0.0000
  Inorganic triphosphate 0.003 0.0000
  Glycolate 0.005 0.0000
  D-Galactose 0.008 0.0000
    
 
