# Supplementary material for: Genomewide landscape of gene–metabolome associations in Escherichia coli
Source: Mol Syst Biol. 2017 Jan 16;13(1):907. doi: 10.15252/msb.20167150 (PMC5293155; doi:10.15252/msb.20167150)
Supplement: Supplementary file 4 — Table EV3 [file MSB-13-907-s004.zip › details/data_yaeI.html]

 
 
 yaeI 
  yaeI - details 
 
 
  CLR  
   Gene_matching CLR_index  yaeF 9.2
  yhfU 7.6
  cspI 7.5
  mhpE 6.9
  tdcG 6.8
  yghS 6.8
  gntR 6.5
  yieM 6.4
  yhiK 6.1
  ygiQ 6.0
  cdaR 6.0
  yjeI 5.9
  argB 5.8
  sthA 5.7
  rbbA 5.7
  dgoA 5.7
  yjgJ 5.6
  dgoD 5.6
  bcsF 5.6
  ydhX 5.5
  pspA 5.5
  yjiV 5.5
  ybbK 5.4
  ytfI 5.3
  tufB 5.3
  fecD 5.3
  srlA 5.2
  yjiY 5.2
  yabI 5.2
  yafD 5.1
  xdhC 5.1
  rhtC 5.1
  ulaA 5.0
  yggL 5.0
  zapA 4.9
  intA 4.9
  yhiQ 4.9
  rplI 4.9
  yiiE 4.9
  ytfT 4.8
  surE 4.8
  ykgL 4.8
  rhtB 4.8
  gntX 4.8
  yjgK 4.7
  fecB 4.7
  ygfQ 4.7
  yhjQ 4.6
  rsmC 4.6
  hslR 4.6
  yjgM 4.6
  yjcB 4.6
  sufS 4.5
  intF 4.5
  thiS 4.5
  yhjY 4.4
  kdgT 4.4
  tatC 4.4
  yoeB 4.3
  rho 4.3
  folP 4.3
  yjbO 4.3
  greB 4.2
  yhcN 4.2
  glcE 4.2
  ygeW 4.2
  yjcQ 4.1
  yhdX 4.1
  emrD 4.1
  ogrK 4.1
  ampG 4.1
  ykiB 4.0
  yjiD 4.0
  yjcS 4.0
  nrfB 4.0
  hybF 4.0
  caiD 4.0
  yjfO 4.0
  cpxP 4.0
  ecnB 4.0
  rimM 4.0
  ykgK 4.0
  yjiQ 4.0
  yeiG 3.9
  hslO 3.9
  yihX 3.9
  yigE 3.9
  yacG 3.8
  yghY 3.8
  yhiO 3.8
  ynfH 3.8
  gspI 3.8
  yigI 3.8
  rpsF 3.8
  iclR 3.7
  yhbX 3.7
  yjhA 3.7
  yicN 3.7
  emrA 3.6
  yrhC 3.6
  yghQ 3.6
  exo 3.6
  yejE 3.6
  yagV 3.6
  tehA 3.6
  yhdJ 3.6
  yjjN 3.6
  yjgW 3.6
  yghE 3.5
  yjbF 3.5
  yhfK 3.5
  yecG 3.5
  clcA 3.5
  yeaW 3.5
  ampC 3.5
  ugpE 3.4
  chpA 3.4
  manZ 3.4
  nmpC 3.4
  yddA 3.4
  acrF 3.4
  wzzE 3.3
  ppiA 3.3
  glpK 3.3
  ygfB 3.3
  ilvL 3.3
  emrY 3.3
  recG 3.3
  yjhD 3.3
  hdeB 3.3
  ygjG 3.3
  ybjR 3.3
  ygeM 3.3
  yfgD 3.3
  ccmA 3.3
  rhlE 3.3
  agaA 3.3
  nemA 3.2
  alx 3.2
  yjgN 3.2
  yedO 3.2
  rluA 3.2
  metL 3.2
  srlD 3.2
  prmA 3.2
  ygjP 3.2
  ydcI 3.2
  yjhH 3.2
  sbmA 3.2
  yiiT 3.2
  ileS 3.2
  codA 3.2
  yicG 3.2
  bcsA 3.1
  uspA 3.1
  ygiB 3.1
  yfiF 3.1
  rhoL 3.1
  yhjH 3.1
  zur 3.1
  yjiM 3.1
  psiF 3.1
  menD 3.0
  yfhR 3.0
  ptsA 3.0
  sgcE 3.0
  yfhQ 3.0
  ygjO 3.0
  arsB 3.0
  ligT 3.0
     Differential ions  
   id name formula mz mod AUC Z-score Z-score AUC Weighted   C03657  1,4-Dihydroxy-2-naphthoate C11H8O4 442.9528 .(H2PO4Na)2-H(+) 0.946 3.524 3.334
   C00644  D-Mannitol 1-phosphate C6H15O9P 502.9704 .(H2PO4Na)2.H(+) 0.727 4.253 3.090
   C06311  Galactitol 1-phosphate C6H15O9P 502.9704 .(H2PO4Na)2.H(+) 0.649 4.253 2.759
   C05973  2-Acyl-sn-glycero-3-phosphoethanolamine (n-C18:0) C23H48NO7P1 520.2633 .H/K.H(+) 0.709 3.533 2.506
   C00288  Bicarbonate CH2O3 82.9754 .H/Na-H(+) 0.623 3.736 2.327
   C00253  Nicotinate C6H5NO2 357.9552 .(H2PO4)2KH.H(+) 0.667 3.487 2.326
   C00365  dUMP C9H13N2O8P 442.9528 .H2PO4K-H(+) 0.633 3.524 2.232
   C01096  D-Sorbitol 6-phosphate C6H15O9P 502.9704 .(H2PO4Na)2.H(+) 0.569 4.253 0.000
   C00818  D-Glucarate C6H10O8 442.9528 .(H2PO4)2KH-H(+) 0.566 3.524 0.000
   C16519  2-succinyl-5-enolpyruvyl-6-hydroxy-3-cyclohexene-1-carboxylate C14H16O9 502.9704 .HPO4K2.H(+) 0.561 4.253 0.000
   Tetradecanoyl-phosphate (n-C14:0)  Tetradecanoyl-phosphate (n-C14:0) C14H29O5P 429.1521 .H2PO4Na.H(+) 0.515 4.646 0.000
   C00879  D-Galactarate C6H10O8 442.9528 .(H2PO4)2KH-H(+) 0.501 3.524 0.000
   L-Prolinylglycine  L-Prolinylglycine C7H12N2O3 442.9528 .(H2PO4K)2-H(+) 0.497 3.524 0.000
     KEGG pathway by CLR  
none  COG enrichment  
   Pathway_MS pvalue_MS qvalue_MS  Lysine biosynthesis 0.0007 0.0625
  Bisphenol degradation 0.002 0.0950
     Predicted metabolites from CLR  
   Predicted metabolites Pvalue Overlap with hits  Fe3+ 0.0004 0.0000
  L-Homoserine 0.0004 0.0000
  D-Sorbitol 6-phosphate 0.0004 1.0000
  Citrate 0.004 0.0000
    
 
