# Supplementary material for: Genomewide landscape of gene–metabolome associations in Escherichia coli
Source: Mol Syst Biol. 2017 Jan 16;13(1):907. doi: 10.15252/msb.20167150 (PMC5293155; doi:10.15252/msb.20167150)
Supplement: Supplementary file 4 — Table EV3 [file MSB-13-907-s004.zip › details/data_yafC.html]

 
 
 yafC 
  yafC - details 
 
 
  CLR  
   Gene_matching CLR_index  zntR 16.4
  ybdO 16.0
  fruR 15.1
  cyoD 14.0
  trpR 11.8
  cyoA 11.7
  galR 10.6
  perR 10.2
  narX 9.7
  galS 9.4
  soxR 9.4
  tdcA 9.3
  btuR 9.0
  nagC 8.9
  yfcP 8.7
  prpC 8.6
  fucR 8.6
  uidR 8.1
  pdhR 8.1
  soxS 8.1
  argR 7.9
  chbR 7.8
  xapR 7.6
  ydbH 7.5
  rbsR 7.4
  yaiI 7.3
  hupB 7.2
  rcsC 7.2
  cytR 7.1
  cheY 6.9
  sdiA 6.7
  cyoB 6.7
  mhpR 6.6
  dacC 6.5
  citB 6.2
  rstB 6.0
  phoP 6.0
  mipA 6.0
  ihfA 6.0
  ebgR 6.0
  frdA 5.9
  ycdX 5.8
  clpP 5.8
  fnr 5.7
  narL 5.7
  betI 5.6
  cheA 5.5
  metR 5.5
  cyoC 5.5
  rpiB 5.5
  djlB 5.4
  glcB 5.3
  purR 5.3
  helD 5.2
  leuO 5.2
  appY 5.2
  ybeF 5.1
  ygjV 4.9
  yeaY 4.9
  hrpA 4.8
  prpR 4.8
  ygaR 4.7
  uxuR 4.7
  wza 4.6
  yfbM 4.6
  clpA 4.5
  yniB 4.5
  ynjH 4.4
  srlR 4.4
  stpA 4.4
  ydfD 4.3
  ygcW 4.3
  lrp 4.3
  pepN 4.3
  yejG 4.2
  dps 4.2
  melR 4.2
  lysR 4.1
  ybeA 4.1
  mdh 4.1
  ycjT 4.0
  yehM 4.0
  yqiK 4.0
  cadC 4.0
  tfaR 4.0
  csgF 4.0
  fiu 3.9
  ydaY 3.9
  yfdK 3.9
  cirA 3.9
  yfaQ 3.9
  dacA 3.8
  yfdX 3.8
  nanR 3.7
  lhr 3.7
  ytjC 3.7
  fdhD 3.6
  nuoK 3.6
  yoaG 3.5
  rhaR 3.5
  rhlB 3.5
  malI 3.5
  yeaT 3.4
  gapC 3.4
  treR 3.4
  potH 3.4
  ygbE 3.4
  uhpA 3.3
  nuoN 3.3
  fdrA 3.3
  gpp 3.2
  nhaR 3.2
  ydiH 3.2
  sdhD 3.2
  pppA 3.1
  aceB 3.1
  eutT 3.1
  envR 3.1
  ybgH 3.1
  sucA 3.0
     Differential ions  
   id name formula mz mod AUC Z-score Z-score AUC Weighted   cyclopropane phosphatidylethanolamine (dihexadec-9,10-cyclo-anoyl, n-C16:0 cyclo)  cyclopropane phosphatidylethanolamine (dihexadec-9,10-cyclo-anoyl, n-C16:0 cyclo) C39H74N1O8P1 716.5212 .H(+) 0.951 4.380 4.164
   C00350  phosphatidylethanolamine (dioctadec-11-enoyl, n-C18:1) C41H78N1O8P1 744.5543 .H(+) 0.798 5.001 3.991
   cyclopropane phosphatidylethanolamine (dihexadec-9,10-cyclo-anoyl, n-C16:0 cyclo)  cyclopropane phosphatidylethanolamine (dihexadec-9,10-cyclo-anoyl, n-C16:0 cyclo) C39H74N1O8P1 718.5338 [+2].H(+) 0.925 4.110 3.803
   C06187  Arbutin 6-phosphate C12H17O10P 351.0514 -H(+) 0.808 3.841 3.104
   C04778  N1-(5-Phospho-alpha-D-ribosyl)-5,6-dimethylbenzimidazole C14H19N2O7P 477.0539 .H2PO4Na-H(+) 0.735 4.151 3.053
   C00112  CDP C9H15N3O11P2 423.9899 .H/Na-H(+) 0.614 4.864 2.987
   C05382  Sedoheptulose 7-phosphate C7H15O10P 424.9738 .H2PO4K-H(+) 0.675 4.325 2.921
   C00575  cAMP C10H12N5O6P 328.0454 -H(+) 0.773 3.764 2.908
   C07836  D-Glycero-D-manno-heptose 7-phosphate C7H15O10P 424.9738 .H2PO4K-H(+) 0.667 4.325 2.885
   C00575  cAMP C10H12N5O6P 330.0516 [+2]-H(+) 0.718 3.626 2.602
   C05932  N2-Succinyl-L-glutamate 5-semialdehyde C9H13NO6 350.0314 .H2PO4Na-H(+) 0.654 3.968 2.596
   C00105  UMP C9H13N2O9P 305.0201 -H2O-H(+) 0.614 4.154 2.552
   C03415  N2-Succinyl-L-ornithine C9H16N2O5 351.0514 .H2PO4Na-H(+) 0.663 3.841 2.546
   C00575  cAMP C10H12N5O6P 448.0070 .H2PO4Na-H(+) 0.654 3.800 2.486
   C05931  N2-Succinyl-L-glutamate C9H13NO7 366.0212 .H2PO4Na-H(+) 0.663 3.723 2.467
   C15767  gamma-glutamyl-gamma aminobutyric acid C9H16O5N2 351.0514 .H2PO4Na-H(+) 0.609 3.841 2.338
   C00575  cAMP C10H12N5O6P 561.9629 .(H2PO4)2KH-H(+) 0.608 3.686 2.240
   C00719  Glycine betaine C5H11NO2 74.0968 -CO2.H(+) 0.603 3.646 2.200
   C00112  CDP C9H15N3O11P2 543.9430 .HPO4Na2-H(+) 0.599 3.570 0.000
   C00112  CDP C9H15N3O11P2 402.0111 -H(+) 0.584 3.705 0.000
   C00015  UDP C9H14N2O12P2 424.9738 .H/Na-H(+) 0.582 4.325 0.000
   C00350  phosphatidylethanolamine (dioctadec-11-enoyl, n-C18:1) C41H78N1O8P1 766.5372 .H/Na.H(+) 0.571 3.895 0.000
   C00350  phosphatidylethanolamine (dioctadec-11-enoyl, n-C18:1) C41H78N1O8P1 766.5372 .Na(+) 0.571 3.895 0.000
   C00054  Adenosine 3',5'-bisphosphate C10H15N5O10P2 448.0070 .H/Na-H(+) 0.567 3.800 0.000
   C04268  dTDP-4-amino-4,6-dideoxy-D-galactose C16H27N3O14P2 682.0187 .H2PO4K-H(+) 0.566 10.685 0.000
   C01037  7,8-Diaminononanoate C9H20N2O2 211.1437 .H/Na.H(+) 0.561 -4.696 -0.000
   C05512  Deoxyinosine C10H12N4O4 424.9738 .HPO4K2-H(+) 0.547 4.325 0.000
   C00286  dGTP C10H16N5O13P3 543.9430 .H/K-H(+) 0.530 3.570 0.000
   C00053  3'-Phosphoadenylyl sulfate C10H15N5O13P2S 543.9430 .H/K-H(+) 0.526 3.570 0.000
   C00942  3',5'-Cyclic GMP C10H12N5O7P 366.0212 .H/Na-H(+) 0.523 3.723 0.000
   C00055  CMP C9H14N3O8P 304.0335 -H2O-H(+) 0.520 4.349 0.000
   C00015  UDP C9H14N2O12P2 402.9931 -H(+) 0.517 3.565 0.000
   C07838  D-Glycero-D-manno-heptose 1-phosphate C7H15O10P 424.9738 .H2PO4K-H(+) 0.515 4.325 0.000
   C00455  NMN C11H15N2O8P 351.0514 +OH(-) 0.506 3.841 0.000
   C00361  dGDP C10H15N5O10P2 561.9629 .H2PO4K-H(+) 0.497 3.686 0.000
   C00054  Adenosine 3',5'-bisphosphate C10H15N5O10P2 561.9629 .H2PO4K-H(+) 0.496 3.686 0.000
   C05198  5'-Deoxyadenosine C10H13N5O3 423.9899 .HPO4K2-H(+) 0.494 4.864 0.000
   C00362  dGMP C10H14N5O7P 328.0454 -H2O-H(+) 0.489 3.764 0.000
   C00055  CMP C9H14N3O8P 561.9629 .(H2PO4Na)2-H(+) 0.488 3.686 0.000
   C00334  4-Aminobutanoate C4H9NO2 126.0554 .H/Na.H(+) 0.483 -3.519 -0.000
   C00361  dGDP C10H15N5O10P2 448.0070 .H/Na-H(+) 0.474 3.800 0.000
   C00183  L-Valine C5H11NO2 74.0968 -CO2.H(+) 0.471 3.646 0.000
   C00559  Deoxyadenosine C10H13N5O3 423.9899 .HPO4K2-H(+) 0.450 4.864 0.000
   C00575  cAMP C10H12N5O6P 350.0314 .H/Na-H(+) 0.409 3.968 0.000
   C00530  Hydroquinone C6H6O2 111.0444 .H(+) 0.321 -3.612 -0.000
   C01134  Pantetheine 4'-phosphate C11H23N2O7PS 477.0539 .H2PO4Na-H(+) 0.000 4.151 0.000
   C02737  phosphatidylserine (dihexadec-9-enoyl, n-C16:1) C38H70N1O10P1 754.4489 .H/Na.H(+) 0.000 -4.112 -0.000
   C05809  3-Octaprenyl-4-hydroxybenzoate C47H70O3 705.5253 .H/Na.H(+) 0.000 4.773 0.000
   C11436  2-phospho-4-(cytidine 5'-diphospho)-2-C-methyl-D-erythritol C14H26N3O17P3 624.0293 .H/Na.H(+) 0.000 -3.745 -0.000
   C11436  2-phospho-4-(cytidine 5'-diphospho)-2-C-methyl-D-erythritol C14H26N3O17P3 873.9268 .(H2PO4K)2.H(+) 0.000 -3.522 -0.000
   C00246  Butyrate (n-C4:0) C4H8O2 111.0444 .H/Na.H(+) 0.608 -3.612 -2.196
   C00479  Propanal C3H6O 81.0330 .H/Na.H(+) 0.701 -3.545 -2.484
   C00479  Propanal C3H6O 81.0330 .Na(+) 0.701 -3.545 -2.484
   C04556  4-Amino-2-methyl-5-phosphomethylpyrimidine C6H10N3O4P 258.0062 .H/K.H(+) 0.647 -3.934 -2.545
   C01909  Dethiobiotin C10H18N2O3 197.1281 -H2O.H(+) 0.790 -3.652 -2.883
     KEGG pathway by CLR  
   Pathway_ion pvalue_ion qvalue_ion  Pyrimidine metabolism 1e-07 0.0000
  Butanoate metabolism 2e-06 0.0001
  Chlorocyclohexane and chlorobenzene degradation 2e-05 0.0004
  Bisphenol degradation 0.0002 0.0051
  Aminobenzoate degradation 0.0009 0.0150
  Lysine degradation 0.002 0.0281
  Benzoate degradation 0.002 0.0241
  Porphyrin and chlorophyll metabolism 0.002 0.0211
  Fluorobenzoate degradation 0.003 0.0293
  Riboflavin metabolism 0.004 0.0321
  Dioxin degradation 0.004 0.0348
  Valine, leucine and isoleucine degradation 0.006 0.0402
  Purine metabolism 0.007 0.0411
  Alanine, aspartate and glutamate metabolism 0.009 0.0513
     COG enrichment  
   Pathway_MS pvalue_MS qvalue_MS  Oxidative phosphorylation 3e-07 0.0000
  Two-component system 0.0001 0.0064
  Citrate cycle (TCA cycle) 0.001 0.0357
  Toluene degradation 0.002 0.0541
  Peptidoglycan biosynthesis 0.004 0.0811
     Predicted metabolites from CLR  
   Predicted metabolites Pvalue Overlap with hits  L-Malate 1e-05 0.0000
  Oxaloacetate 0.0004 0.0000
  Glyoxylate 0.0007 0.0000
  2-Demethylmenaquinone 8 0.0008 0.0000
  2-Demethylmenaquinol 8 0.0009 0.0000
  Fumarate 0.002 0.0000
  Succinate 0.006 0.0000
    
 
