# Supplementary material for: Genomewide landscape of gene–metabolome associations in Escherichia coli
Source: Mol Syst Biol. 2017 Jan 16;13(1):907. doi: 10.15252/msb.20167150 (PMC5293155; doi:10.15252/msb.20167150)
Supplement: Supplementary file 4 — Table EV3 [file MSB-13-907-s004.zip › details/data_yafO.html]

 
 
 yafO 
  yafO - details 
 
 
  CLR  
   Gene_matching CLR_index  ygiL 12.6
  sdaA 11.8
  rcsB 9.9
  yejL 9.6
  arsC 9.3
  yegX 9.2
  truB 9.0
  nohB 9.0
  yliE 8.8
  ycjN 8.7
  lsrF 8.4
  poxB 8.1
  tyrP 8.1
  yfdV 7.6
  frc 7.4
  ymfB 7.3
  glvG 7.1
  yphH 6.8
  rseC 6.8
  ynbA 6.8
  rzoR 6.7
  ydfP 6.6
  ymcC 6.5
  treA 6.5
  garP 6.4
  eco 6.3
  ycdN 6.3
  deoC 6.2
  sseB 6.2
  ybhH 6.1
  gnsB 6.1
  yadK 6.0
  mcrA 6.0
  acnA 5.9
  ynjF 5.8
  yceI 5.8
  bfd 5.8
  ydcV 5.8
  ygaF 5.8
  mscS 5.8
  pldB 5.8
  mlrA 5.8
  yafT 5.7
  torT 5.7
  ygdE 5.7
  yfdH 5.7
  ygcP 5.7
  csdA 5.6
  ssuA 5.6
  aaeR 5.6
  ydcS 5.6
  ybgE 5.6
  yehZ 5.5
  ksgA 5.5
  ydaL 5.4
  araG 5.4
  tktB 5.4
  yecF 5.3
  ymjA 5.3
  ybdM 5.3
  ycjY 5.3
  gspA 5.2
  pdhR 5.2
  yjbG 5.2
  yeiW 5.0
  gltF 5.0
  ychN 5.0
  ytjC 5.0
  ybhA 5.0
  coaA 5.0
  yaiA 4.9
  gcvP 4.9
  mpaA 4.9
  nrdD 4.9
  ydgG 4.8
  mltB 4.8
  ycbS 4.8
  ycjM 4.8
  yfiC 4.8
  ymfQ 4.8
  frsA 4.7
  ybhS 4.7
  speD 4.7
  ompF 4.7
  tsx 4.6
  ada 4.6
  yfiQ 4.6
  ydbA 4.6
  yraK 4.6
  artP 4.5
  fiu 4.5
  appC 4.5
  glgB 4.5
  galK 4.5
  yijP 4.5
  yegL 4.4
  envZ 4.4
  agaV 4.4
  yphF 4.4
  prpR 4.4
  sspB 4.4
  ykfI 4.4
  ompN 4.4
  ydjM 4.4
  yeaL 4.3
  frvR 4.3
  nlpB 4.3
  uup 4.3
  eaeH 4.3
  ycaC 4.2
  ybcS 4.2
  prfH 4.2
  mhpT 4.1
  cpdB 4.1
  yqeB 4.1
  yfbL 4.1
  yheV 4.1
  ymfD 4.1
  yacH 4.0
  atoB 4.0
  yfhA 4.0
  yejA 4.0
  ycgK 4.0
  ymgC 4.0
  pepN 4.0
  yliI 4.0
  ydiH 3.9
  yfhJ 3.9
  ilvG 3.9
  ybjR 3.9
  ymfM 3.9
  yjeS 3.9
  cspB 3.9
  phoB 3.9
  rph 3.8
  yaeH 3.8
  frdB 3.8
  yzcX 3.8
  nuoN 3.8
  puuP 3.8
  nanR 3.8
  yeeI 3.8
  ldcA 3.8
  yodB 3.7
  yciG 3.7
  yhdH 3.7
  hrpA 3.7
  ydiM 3.7
  ygeD 3.6
  ynaE 3.6
  yfjY 3.6
  yliF 3.6
  srlD 3.5
  ytfG 3.5
  ybcH 3.5
  galT 3.4
  yidZ 3.4
  ydfO 3.4
  ybbS 3.4
  yidE 3.4
  yqaE 3.4
  yfaO 3.4
  mdtD 3.4
  folP 3.4
  ydhV 3.3
  yodC 3.3
  ybgH 3.3
  ygaZ 3.3
  yedZ 3.3
  cirA 3.3
  rlpA 3.3
  yejH 3.3
  ybcK 3.2
  ymcB 3.2
  yeaY 3.2
  envC 3.2
  paaG 3.2
  rsxG 3.2
  yhcE 3.2
  hyuA 3.2
  ccmG 3.2
  hflK 3.2
  yhcF 3.2
  yfhH 3.1
  eutG 3.1
  yfhG 3.1
  hycG 3.1
  uhpC 3.1
  ydjX 3.1
  ybbD 3.1
  ypdC 3.1
  rnt 3.0
  yeaC 3.0
  glvC 3.0
  ycaO 3.0
  ygaQ 3.0
  yncE 3.0
  malX 3.0
  lsrD 3.0
  yghB 3.0
  yehW 3.0
  ybdB 3.0
  yfeO 3.0
  dppD 3.0
     Differential ions  
   id name formula mz mod AUC Z-score Z-score AUC Weighted   C00129  Isopentenyl diphosphate C5H12O7P2 366.9702 .H2PO4Na.H(+) 0.903 4.152 3.751
   C00235  Dimethylallyl diphosphate C5H12O7P2 366.9702 .H2PO4Na.H(+) 0.903 4.152 3.751
   C04133  N-Acetyl-L-glutamyl 5-phosphate C7H12NO8P 509.9603 .(H2PO4Na)2.H(+) 0.778 4.538 3.529
   C05925  Dihydroneopterin monophosphate C9H14N5O7P 374.0261 .H/K.H(+) 0.930 3.753 3.490
   C05925  Dihydroneopterin monophosphate C9H14N5O7P 509.9603 .HPO4K2.H(+) 0.705 4.538 3.199
   C00575  cAMP C10H12N5O6P 352.0439 .H/Na.H(+) 0.748 4.200 3.142
   C00575  cAMP C10H12N5O6P 352.0439 .Na(+) 0.748 4.200 3.142
   C00942  3',5'-Cyclic GMP C10H12N5O7P 384.0101 .H/K.H(+) 0.726 4.291 3.115
   C05931  N2-Succinyl-L-glutamate C9H13NO7 384.0101 .H2PO4K.H(+) 0.719 4.291 3.083
   C01031  S-Formylglutathione C11H17N3O7S 509.9603 .HPO4K2.H(+) 0.634 4.538 2.877
   C00612  N1-Acetylspermidine C9H21N3O 330.1233 .HPO4Na2.H(+) 0.706 3.735 2.635
   C01029  N8-Acetylspermidine C9H21N3O 330.1233 .HPO4Na2.H(+) 0.706 3.735 2.635
   C00575  cAMP C10H12N5O6P 330.0629 .H(+) 0.633 3.887 2.461
   C05932  N2-Succinyl-L-glutamate 5-semialdehyde C9H13NO6 374.0261 .HPO4Na2.H(+) 0.638 3.753 2.395
   C00152  L-Asparagine C4H8N2O3 366.9702 .(H2PO4)2KH.H(+) 0.598 4.152 0.000
   C04133  N-Acetyl-L-glutamyl 5-phosphate C7H12NO8P 390.0042 .H2PO4Na.H(+) 0.582 3.815 0.000
   C01079  Protoporphyrinogen IX C34H40N4O4 607.2643 .H/K.H(+) 0.566 3.483 0.000
   C00178  Thymine C5H6N2O2 366.9702 .(H2PO4Na)2.H(+) 0.557 4.152 0.000
   C05932  N2-Succinyl-L-glutamate 5-semialdehyde C9H13NO6 352.0439 .H2PO4Na.H(+) 0.456 4.200 0.000
     KEGG pathway by CLR  
   Pathway_ion pvalue_ion qvalue_ion  Purine metabolism 3e-06 0.0003
     COG enrichment  
   Pathway_MS pvalue_MS qvalue_MS  Terpenoid backbone biosynthesis 0.008 0.8095
     Predicted metabolites from CLR  
   Predicted metabolites Pvalue Overlap with hits  2-dodecanoyl-sn-glycerol 3-phosphate 0 0.0000
  2-hexadec-9-enoyl-sn-glycerol 3-phosphate 0 0.0000
  2-hexadecanoyl-sn-glycerol 3-phosphate 0 0.0000
  2-octadec-11-enoyl-sn-glycerol 3-phosphate 0 0.0000
  2-octadecanoyl-sn-glycerol 3-phosphate 0 0.0000
  2-tetradec-7-enoyl-sn-glycerol 3-phosphate 0 0.0000
  2-tetradecanoyl-sn-glycerol 3-phosphate 0 0.0000
  alpha-D-Galactose 1-phosphate 9e-05 0.0000
  2-Acyl-sn-glycero-3-phosphoethanolamine (n-C12:0) 0.0003 0.0000
  2-Acyl-sn-glycero-3-phosphoethanolamine (n-C14:0) 0.0003 0.0000
  2-Acyl-sn-glycero-3-phosphoethanolamine (n-C14:1) 0.0003 0.0000
  2-Acyl-sn-glycero-3-phosphoethanolamine (n-C16:0) 0.0003 0.0000
  2-Acyl-sn-glycero-3-phosphoethanolamine (n-C16:1) 0.0003 0.0000
  2-Acyl-sn-glycero-3-phosphoethanolamine (n-C18:0) 0.0003 0.0000
  2-Acyl-sn-glycero-3-phosphoethanolamine (n-C18:1) 0.0003 0.0000
  2-Acyl-sn-glycero-3-phosphoglycerol (n-C12:0) 0.0003 0.0000
  2-Acyl-sn-glycero-3-phosphoglycerol (n-C14:0) 0.0003 0.0000
  2-Acyl-sn-glycero-3-phosphoglycerol (n-C14:1) 0.0003 0.0000
  2-Acyl-sn-glycero-3-phosphoglycerol (n-C16:0) 0.0003 0.0000
  2-Acyl-sn-glycero-3-phosphoglycerol (n-C16:1) 0.0003 0.0000
  2-Acyl-sn-glycero-3-phosphoglycerol (n-C18:0) 0.0003 0.0000
  2-Acyl-sn-glycero-3-phosphoglycerol (n-C18:1) 0.0003 0.0000
  L-alanine-D-glutamate 0.002 0.0000
  dUTP 0.003 0.0000
  Choline 0.004 0.0000
  Glycine betaine 0.004 0.0000
  Cys-Gly 0.006 0.0000
  dCTP 0.006 0.0000
  L-Prolinylglycine 0.006 0.0000
  L-alanine-D-glutamate-meso-2,6-diaminoheptanedioate 0.008 0.0000
    
 
