# Supplementary material for: Genomewide landscape of gene–metabolome associations in Escherichia coli
Source: Mol Syst Biol. 2017 Jan 16;13(1):907. doi: 10.15252/msb.20167150 (PMC5293155; doi:10.15252/msb.20167150)
Supplement: Supplementary file 4 — Table EV3 [file MSB-13-907-s004.zip › details/data_yafT.html]

 
 
 yafT 
  yafT - details 
 
 
  CLR  
   Gene_matching CLR_index  ypfI 14.1
  yfeX 13.5
  yebG 13.5
  yadL 13.2
  yohK 13.0
  yadK 12.3
  yccR 11.9
  yobD 11.7
  pnuC 11.6
  ynhG 11.5
  yeiJ 11.4
  envR 11.2
  ydhD 11.1
  ykfI 10.9
  uhpB 10.8
  gltJ 10.5
  mdtC 10.5
  ynfL 10.4
  tatE 10.3
  yebZ 10.0
  clpA 9.8
  wza 9.5
  gltL 9.4
  ydiO 9.2
  citT 8.9
  yafQ 8.8
  ybbO 8.8
  deoA 8.6
  ypjB 8.5
  yeaK 8.5
  iscS 8.3
  ybfM 8.2
  gltB 8.1
  potH 8.1
  leuO 8.0
  rhaT 7.8
  ydhR 7.7
  cyoD 7.5
  hofQ 7.5
  kdpA 7.5
  cbl 7.4
  yeiE 7.3
  ybhS 7.1
  rsxA 6.9
  yahE 6.8
  cusC 6.7
  argR 6.7
  yfhJ 6.7
  ymcB 6.7
  yegE 6.6
  fdhE 6.6
  nudG 6.6
  tfaE 6.4
  ydfA 6.3
  yaiS 6.2
  ygaC 6.2
  eutI 6.2
  ycfT 6.1
  sfsA 6.0
  ygaH 5.9
  nrdG 5.8
  yraH 5.7
  yafO 5.7
  kdpC 5.7
  aer 5.7
  nagE 5.6
  potE 5.4
  ygeL 5.3
  ompF 5.2
  btuD 5.2
  deoD 5.1
  yliF 5.1
  ysdC 5.0
  ihfA 5.0
  yehK 5.0
  ddpF 5.0
  yidZ 4.9
  sdhD 4.9
  phnF 4.9
  ldcC 4.9
  poxB 4.9
  ybhK 4.8
  fadB 4.8
  yfdK 4.8
  fnr 4.8
  rbsK 4.7
  yfcT 4.7
  ydgD 4.7
  phnI 4.7
  ycdS 4.6
  yfhG 4.5
  ygaW 4.5
  clpX 4.5
  ydfH 4.5
  thiD 4.5
  ydjN 4.5
  metJ 4.5
  hyaB 4.4
  hsdR 4.4
  ybiA 4.4
  ydiT 4.4
  yfjQ 4.3
  ygdE 4.3
  mipA 4.3
  yfjN 4.3
  ygbE 4.3
  ygiV 4.2
  ybiR 4.2
  yfjH 4.2
  yebO 4.1
  ydhW 4.1
  yecR 4.1
  ppdD 4.1
  htpX 4.0
  yfjK 4.0
  ygfY 3.9
  frsA 3.9
  wcaL 3.9
  ycjG 3.9
  ydbC 3.8
  mhpR 3.8
  nrdE 3.8
  ydfC 3.7
  yniA 3.7
  yfaO 3.7
  tyrA 3.7
  yeeI 3.7
  frwD 3.7
  fdrA 3.6
  yejL 3.6
  cobC 3.6
  yccJ 3.6
  ybbB 3.6
  lsrB 3.6
  uxuR 3.6
  ykgD 3.6
  bglF 3.6
  tdcA 3.6
  yqgA 3.5
  frc 3.5
  yfbP 3.5
  ilvC 3.5
  slp 3.5
  pstA 3.5
  bcsE 3.5
  ydgK 3.5
  cpxA 3.5
  ygcP 3.4
  hisI 3.4
  torC 3.4
  gnsA 3.4
  chaC 3.4
  yegL 3.4
  fadL 3.4
  ydeI 3.4
  ybeR 3.3
  dusC 3.3
  yjiE 3.3
  ybgE 3.2
  sdhC 3.2
  yehM 3.2
  lldD 3.2
  ycjW 3.2
  nhaR 3.2
  ydiS 3.2
  cybC 3.2
  cpdB 3.2
  ygjE 3.2
  ybdO 3.1
  yceI 3.1
  yqgB 3.1
  ydgT 3.1
  ygbA 3.1
  yeaC 3.1
  ydhA 3.1
  yqaE 3.0
  sfmD 3.0
     Differential ions  
   id name formula mz mod AUC Z-score Z-score AUC Weighted   C01530  octadecanoate (n-C18:0) C18H36O2 323.2386 .H/K.H(+) 0.536 4.359 0.000
   C05973  2-Acyl-sn-glycero-3-phosphoethanolamine (n-C18:1) C23H46NO7P1 480.3073 .H(+) 0.489 3.474 0.000
     KEGG pathway by CLR  
   Pathway_ion pvalue_ion qvalue_ion  Arachidonic acid metabolism 0 0.0000
  Sphingolipid metabolism 2e-05 0.0011
     COG enrichment  
   Pathway_MS pvalue_MS qvalue_MS  Toluene degradation 0.007 0.6463
  Lysine degradation 0.009 0.4460
     Predicted metabolites from CLR  
   Predicted metabolites Pvalue Overlap with hits  dCTP 0.0002 0.0000
  dCMP 0.001 0.0000
  dUTP 0.002 0.0000
  Deoxyuridine 0.003 0.0000
  2-Deoxy-D-ribose 1-phosphate 0.004 0.0000
  CTP 0.004 0.0000
  dADP 0.004 0.0000
  dGDP 0.004 0.0000
  dUDP 0.004 0.0000
  dCDP 0.005 0.0000
  CDP 0.007 0.0000
    
 
