# Supplementary material for: Genomewide landscape of gene–metabolome associations in Escherichia coli
Source: Mol Syst Biol. 2017 Jan 16;13(1):907. doi: 10.15252/msb.20167150 (PMC5293155; doi:10.15252/msb.20167150)
Supplement: Supplementary file 4 — Table EV3 [file MSB-13-907-s004.zip › details/data_yafU.html]

 
 
 yafU 
  yafU - details 
 
 
  CLR  
   Gene_matching CLR_index  holD 11.5
  ydiZ 9.2
  cysH 9.1
  uidA 8.4
  glmM 8.3
  yjeK 7.9
  pdxJ 7.8
  frlR 7.8
  nudB 7.8
  rsgA 7.8
  murP 7.7
  pdxA 7.2
  ygcN 7.0
  pnp 6.7
  ymgA 6.7
  yccZ 6.5
  ybgA 6.3
  yahC 6.2
  yfhL 6.1
  yzgL 6.1
  wcaB 5.9
  metE 5.8
  holE 5.7
  spr 5.7
  leuD 5.6
  yjeP 5.4
  ybdR 5.4
  gor 5.4
  ilvA 5.3
  ygcL 5.3
  puuD 5.3
  ygiE 5.1
  bcsC 5.1
  tgt 5.0
  yjcS 5.0
  tiaE 4.9
  ydaS 4.9
  fliY 4.8
  yqjD 4.7
  tatD 4.7
  ydhL 4.7
  flk 4.7
  ydhF 4.6
  yeaR 4.6
  cof 4.5
  sbmC 4.5
  aroH 4.5
  ilvE 4.5
  ygjN 4.5
  cysC 4.5
  chbC 4.5
  yjdF 4.5
  cchB 4.5
  yjbJ 4.4
  ysgA 4.3
  ypjL 4.3
  gadX 4.3
  moeB 4.3
  cysJ 4.3
  agaD 4.3
  efp 4.2
  yqjB 4.2
  ybaO 4.2
  yhbJ 4.1
  sfmC 4.1
  malF 4.1
  yhdV 4.1
  paaI 4.1
  ypdG 4.1
  cysN 4.1
  yjhG 4.1
  yhfY 4.1
  yfgC 4.1
  frvA 4.1
  mutL 4.1
  yqiJ 4.0
  yceD 4.0
  yfcY 4.0
  yddM 4.0
  yjjJ 4.0
  panC 4.0
  purC 4.0
  ccmH 3.9
  rnb 3.9
  yniC 3.9
  fhiA 3.9
  ygjR 3.8
  ygcS 3.8
  miaA 3.8
  yafN 3.8
  ycdQ 3.8
  rbsD 3.8
  cld 3.8
  ykgH 3.8
  crcB 3.8
  dcuA 3.7
  yghO 3.7
  moaB 3.7
  gspO 3.7
  cysD 3.7
  ybjS 3.7
  ygeR 3.6
  cpxP 3.6
  ilvB 3.6
  avtA 3.6
  ybfE 3.5
  glpC 3.5
  ydcH 3.5
  yegR 3.5
  yphC 3.5
  yeaG 3.5
  uspE 3.5
  trxA 3.4
  metL 3.4
  fecC 3.4
  ydiD 3.4
  prmB 3.4
  yahK 3.4
  phr 3.4
  yeaA 3.4
  yjcO 3.3
  ygeX 3.3
  yqjF 3.3
  yebT 3.3
  htrE 3.3
  hyfB 3.3
  ulaC 3.3
  nrdF 3.3
  asr 3.3
  gntY 3.2
  gspI 3.2
  norR 3.2
  ypfN 3.2
  ubiG 3.2
  yhhJ 3.2
  prpD 3.2
  ymdE 3.2
  ilvM 3.2
  hisQ 3.2
  purE 3.2
  prfB 3.2
  cysI 3.2
  pdxB 3.1
  leuC 3.1
  betT 3.1
  katE 3.1
  hycC 3.1
  yaaU 3.1
  speA 3.1
  yqfA 3.1
  alaS 3.1
  yeeN 3.1
  aroL 3.1
  yoeF 3.1
  mcrB 3.0
  purU 3.0
  metH 3.0
  yeeJ 3.0
  cls 3.0
  fhuF 3.0
  sgbE 3.0
  ylcE 3.0
  yjhF 3.0
     Differential ions  
   id name formula mz mod AUC Z-score Z-score AUC Weighted   C08362  Hexadecenoate (n-C16:1) C16H30O2 271.2288 +OH(-) 0.633 3.846 2.436
   C06006  (S)-2-Aceto-2-hydroxybutanoate C6H10O4 289.0105 .HPO4Na2.H(+) 0.564 -3.672 -0.000
   C00887  Nitrous oxide N2O 162.9558 .H2PO4Na-H(+) 0.557 3.716 0.000
   C00966  2-Dehydropantoate C6H10O4 289.0105 .HPO4Na2.H(+) 0.512 -3.672 -0.000
   C00064  L-Glutamine C5H10N2O3 289.0105 .HPO4Na2.H(+) 0.508 -3.672 -0.000
     KEGG pathway by CLR  
none  COG enrichment  
   Pathway_MS pvalue_MS qvalue_MS  Chlorocyclohexane and chlorobenzene degradation 0 0.0000
  Fluorobenzoate degradation 0 0.0000
  Valine, leucine and isoleucine biosynthesis 3e-07 0.0000
  Sulfur metabolism 3e-07 0.0000
  C5-Branched dibasic acid metabolism 2e-05 0.0002
  Vitamin B6 metabolism 0.0002 0.0023
  Selenoamino acid metabolism 0.0004 0.0041
  Pantothenate and CoA biosynthesis 0.0006 0.0049
  Purine metabolism 0.0006 0.0049
  Biosynthesis of secondary metabolites 0.002 0.0144
  Mismatch repair 0.003 0.0204
  alpha-Linolenic acid metabolism 0.005 0.0324
  Ethylbenzene degradation 0.005 0.0301
  One carbon pool by folate 0.005 0.0284
  DNA replication 0.005 0.0267
     Predicted metabolites from CLR  
   Predicted metabolites Pvalue Overlap with hits  5-amino-1-(5-phospho-D-ribosyl)imidazole-4-carboxylate 0 0.0000
  Adenosine 5'-phosphosulfate 0 0.0000
  Reduced riboflavin 4e-05 0.0000
  Riboflavin 4e-05 0.0000
  3-Carboxy-2-hydroxy-4-methylpentanoate 0.0002 0.0000
  3-Carboxy-3-hydroxy-4-methylpentanoate 0.0002 0.0000
  5-Methyltetrahydrofolate 0.0002 0.0000
  L-methionine-R-sulfoxide 0.0002 0.0000
  GDP 0.0002 0.0000
  2-Isopropylmaleate 0.0006 0.0000
  Pyridoxine 5'-phosphate 0.0006 0.0000
  O-Phospho-4-hydroxy-L-threonine 0.0006 0.0000
  3'-Phosphoadenylyl sulfate 0.0008 0.0000
  3-Methyl-2-oxobutanoate 0.001 0.0000
  Sulfite 0.002 0.0000
  CDP 0.002 0.0000
  L-Methionine 0.002 0.0000
  Decanoyl-CoA (n-C10:0CoA) 0.003 0.0000
  Dodecanoyl-CoA (n-C12:0CoA) 0.003 0.0000
  Octanoyl-CoA (n-C8:0CoA) 0.003 0.0000
  Palmitoyl-CoA (n-C16:0CoA) 0.003 0.0000
  Tetradecanoyl-CoA (n-C14:0CoA) 0.003 0.0000
  FMN 0.003 0.0000
  Reduced FMN 0.003 0.0000
  5,6,7,8-Tetrahydrofolate 0.003 0.0000
  Hydrogen sulfide 0.005 0.0000
  L-Homocysteine 0.005 0.0000
  1-deoxy-D-xylulose 5-phosphate 0.007 0.0000
  Hexanoyl-CoA (n-C6:0CoA) 0.007 0.0000
  dADP 0.01 0.0000
  dGDP 0.01 0.0000
  dUDP 0.01 0.0000
    
 
