# Supplementary material for: Genomewide landscape of gene–metabolome associations in Escherichia coli
Source: Mol Syst Biol. 2017 Jan 16;13(1):907. doi: 10.15252/msb.20167150 (PMC5293155; doi:10.15252/msb.20167150)
Supplement: Supplementary file 4 — Table EV3 [file MSB-13-907-s004.zip › details/data_yafY.html]

 
 
 yafY 
  yafY - details 
 
 
  CLR  
   Gene_matching CLR_index  yjgD 16.6
  fucA 15.4
  flgG 15.3
  idi 15.2
  frvB 14.8
  intD 14.8
  alpA 14.2
  yacL 14.0
  azoR 13.3
  metN 12.9
  tap 12.4
  yajC 12.3
  pyrB 12.0
  uxuA 11.8
  uvrA 11.2
  ydhH 11.1
  yfaD 11.0
  poxA 11.0
  paaB 11.0
  yghF 10.7
  pyrE 10.7
  purD 10.4
  xylA 10.4
  recX 10.4
  iap 10.3
  tldD 10.2
  aroP 10.1
  purR 9.7
  yghX 9.6
  uhpA 9.5
  arnT 9.3
  smpB 9.2
  dcd 9.1
  gltA 9.1
  purF 9.0
  fixX 9.0
  selA 9.0
  rbsB 8.8
  dinG 8.6
  apaH 8.6
  ygbJ 8.4
  oppF 8.3
  napB 8.2
  yncM 8.1
  rnhB 7.8
  ybjR 7.7
  nadB 7.6
  mutM 7.6
  treR 7.5
  pyrF 7.1
  aroA 7.0
  ybcL 6.5
  purL 6.4
  yegX 6.4
  hyaE 6.4
  yqjF 6.3
  yqeJ 6.3
  lacY 6.2
  kefF 6.2
  rem 6.2
  oxc 5.9
  menA 5.8
  yebR 5.8
  chpB 5.8
  norV 5.8
  yliC 5.7
  yegL 5.7
  yidZ 5.6
  bioF 5.6
  rluD 5.3
  proC 5.3
  fucO 5.3
  gnsB 5.3
  ydfO 5.3
  yfaL 5.2
  ybfO 5.2
  ycdR 5.2
  flgJ 5.0
  narW 5.0
  purM 4.9
  cobT 4.9
  yodB 4.9
  ppiD 4.9
  ymgE 4.9
  mioC 4.8
  serA 4.4
  ydaU 4.4
  yphH 4.4
  ddlA 4.4
  ypfH 4.4
  kdpB 4.3
  mpaA 4.3
  ybjM 4.3
  ygiS 4.3
  ydfW 4.3
  purN 4.3
  ybhP 4.2
  srlR 4.2
  fiu 4.2
  ulaF 4.1
  fis 4.1
  idnR 4.1
  ybbP 4.0
  katE 4.0
  fepE 3.9
  dam 3.7
  yfbT 3.7
  ymgG 3.7
  ligB 3.7
  yedZ 3.6
  yeiW 3.6
  yfiP 3.5
  sapC 3.5
  oppD 3.5
  yhcE 3.5
  uspF 3.4
  gidA 3.4
  nuoA 3.4
  ybeH 3.4
  yjeK 3.4
  hns 3.4
  yaiZ 3.4
  recB 3.3
  garP 3.3
  malG 3.3
  galP 3.3
  citF 3.3
  ycdN 3.3
  mutT 3.2
  betT 3.2
  yehW 3.2
  csdA 3.1
  eutC 3.1
  yhbP 3.1
  ycfQ 3.1
  rhaR 3.1
  yeaJ 3.1
  sufB 3.0
  trpA 3.0
  nuoN 3.0
  yibD 3.0
  panC 3.0
     Differential ions  
   id name formula mz mod AUC Z-score Z-score AUC Weighted   C01005  O-Phospho-L-serine C3H8NO6P 106.0499 -HPO3.H(+) 0.934 37.505 35.032
   C06735  Aminoacetaldehyde C2H5NO 60.0446 .H(+) 0.760 23.886 18.147
   C01909  Dethiobiotin C10H18N2O3 216.1427 [+1].H(+) 0.871 16.305 14.196
   C01909  Dethiobiotin C10H18N2O3 215.1390 .H(+) 0.621 16.454 10.223
   C01909  Dethiobiotin C10H18N2O3 237.1223 .H/Na.H(+) 0.828 11.734 9.721
   C01909  Dethiobiotin C10H18N2O3 237.1223 .Na(+) 0.828 11.734 9.721
   C16565  N-3-aminopropyl-1,5-diaminopentane C8H21N3 280.1381 .H2PO4Na.H(+) 0.799 11.765 9.395
   C00670  sn-Glycero-3-phosphocholine C8H20NO6P 258.1109 .H(+) 0.628 14.167 8.902
   C05775  N1-(alpha-D-ribosyl)-5,6-dimethylbenzimidazole C14H18N2O4 280.1381 [+1].H(+) 0.752 11.765 8.851
   C01909  Dethiobiotin C10H18N2O3 217.1473 [+2].H(+) 0.896 7.896 7.075
   C15998  L-methionine-R-sulfoxide C5H11NO3S 166.0509 .H(+) 0.712 9.709 6.909
   C01236  6-phospho-D-glucono-1,5-lactone C6H11O9P 280.9980 .H/Na.H(+) 0.912 7.357 6.710
   C15699  gamma-glutamyl-putrescine C9H19O3N3 219.1488 [+1].H(+) 0.675 9.900 6.686
   C05775  N1-(alpha-D-ribosyl)-5,6-dimethylbenzimidazole C14H18N2O4 281.1411 [+2].H(+) 0.748 8.464 6.332
   C00793  D-Cysteine C3H7NO2S 144.0080 .H/Na.H(+) 0.891 7.034 6.270
   C00062  L-Arginine C6H14N4O2 213.0755 .H/K.H(+) 0.620 10.076 6.245
   C00062  L-Arginine C6H14N4O2 213.0755 .K(+) 0.620 10.076 6.245
   C06424  tetradecanoate (n-C14:0) C14H28O2 371.1617 .HPO4Na2.H(+) 0.705 7.466 5.261
   C00445  5,10-Methenyltetrahydrofolate C20H22N7O6 457.1660 .H(+) 0.795 6.369 5.061
   C08362  Hexadecenoate (n-C16:1) C16H30O2 397.1716 .HPO4Na2.H(+) 0.649 7.702 4.997
   C08362  Hexadecenoate (n-C16:1) C16H30O2 375.1932 .H2PO4Na.H(+) 0.642 6.541 4.199
   C05973  2-Acyl-sn-glycero-3-phosphoethanolamine (n-C16:0) C21H44NO7P1 672.2375 .(H2PO4)2NaH.H(+) 0.726 5.784 4.198
   C00855  D-Methionine C5H11NO2S 150.0588 .H(+) 0.769 5.362 4.121
   C02130  Acetyl-maltose C14H24O12 386.1308 [+1].H(+) 0.658 5.868 3.863
   C15809  dehydroglycine C2H3NO2 313.9433 .(H2PO4Na)2.H(+) 0.679 5.622 3.816
   C01083  Trehalose C12H22O11 345.1336 [+2].H(+) 0.892 4.246 3.786
   C00534  Pyridoxamine C8H12N2O2 207.0518 .H/K.H(+) 0.861 4.383 3.774
   C00641  1,2-Diacyl-sn-glycerol (didodecanoyl, n-C12:0) C27H52O5 593.3263 .H2PO4K.H(+) 0.850 4.435 3.768
   C06156  D-Glucosamine 1-phosphate C6H14NO8P 262.0564 [+2].H(+) 0.999 3.757 3.753
   C00380  Cytosine C4H5N3O 285.9376 .HPO4K2.H(+) 0.779 4.736 3.691
   C00855  D-Methionine C5H11NO2S 133.0323 -NH3.H(+) 0.751 4.875 3.662
   C00079  L-Phenylalanine C9H11NO2 188.0692 .H/Na.H(+) 0.626 5.687 3.560
   C00079  L-Phenylalanine C9H11NO2 188.0692 .Na(+) 0.626 5.687 3.560
   C00242  Guanine C5H5N5O 134.0449 -H2O.H(+) 0.742 4.766 3.536
   C15767  gamma-glutamyl-gamma aminobutyric acid C9H16O5N2 257.1009 [+2].Na(+) 0.909 3.849 3.498
   C03406  N(omega)-(L-Arginino)succinate C10H18N4O6 313.1133 .H/Na.H(+) 0.874 3.980 3.479
   C00263  L-Homoserine C4H9NO3 142.0477 .H/Na.H(+) 0.715 4.762 3.403
   C01909  Dethiobiotin C10H18N2O3 197.1281 -H2O.H(+) 0.790 4.191 3.309
   C00073  L-Methionine C5H11NO2S 133.0323 -NH3.H(+) 0.678 4.875 3.304
   C00062  L-Arginine C6H14N4O2 175.1189 .H(+) 0.649 5.086 3.300
   C00073  L-Methionine C5H11NO2S 150.0588 .H(+) 0.615 5.362 3.299
   C02989  L-Methionine Sulfoxide C5H11NO3S 188.0386 .H/Na.H(+) 0.762 4.254 3.244
   C12621  3-hydroxycinnamic acid C9H8O3 121.0630 -CO2.H(+) 0.861 3.738 3.220
   C01888  Aminoacetone C3H7NO 56.0496 -H2O.H(+) 0.689 4.659 3.209
   C00137  myo-Inositol C6H12O6 323.0108 .HPO4Na2.H(+) 0.883 3.628 3.204
   C00719  Glycine betaine C5H11NO2 74.0968 -CO2.H(+) 0.603 5.294 3.195
   C00601  Phenylacetaldehyde C8H8O 121.0630 .H(+) 0.849 3.738 3.174
   C00270  N-Acetylneuraminate C11H19NO9 310.1172 .H(+) 0.782 4.055 3.170
   Glycerophosphoserine  Glycerophosphoserine C6H14NO8P 262.0564 [+2].H(+) 0.837 3.757 3.144
   Tetradecanoyl-phosphate (n-C14:0)  Tetradecanoyl-phosphate (n-C14:0) C14H29O5P 347.1414 .H/K.H(+) 0.712 4.395 3.130
   C00015  UDP C9H14N2O12P2 638.9113 .(H2PO4)2KH.H(+) 0.659 4.598 3.031
   C00208  Maltose C12H22O11 345.1336 [+2].H(+) 0.694 4.246 2.948
   C01097  D-Tagatose 6-phosphate C6H13O9P 500.9617 .(H2PO4Na)2.H(+) 0.769 3.824 2.942
   C00079  L-Phenylalanine C9H11NO2 167.0902 [+1].H(+) 0.781 3.686 2.881
   C00979  O-Acetyl-L-serine C5H9NO4 130.0498 -H2O.H(+) 0.808 3.482 2.814
   C00636  D-Mannose 1-phosphate C6H13O9P 500.9617 .(H2PO4Na)2.H(+) 0.732 3.824 2.799
   C00159  D-Mannose C6H12O6 203.0529 .H/Na.H(+) 0.768 3.562 2.737
   C00860  L-Histidinol C6H11N3O 262.0564 .H2PO4Na.H(+) 0.722 3.757 2.712
   C00109  2-Oxobutanoate C4H6O3 85.0284 -H2O.H(+) 0.661 4.058 2.682
   C00164  Acetoacetate C4H6O3 85.0284 -H2O.H(+) 0.608 4.058 2.467
   C00137  myo-Inositol C6H12O6 203.0529 .H/Na.H(+) 0.689 3.562 2.454
   C02962  D-Allose 6-phosphate C6H13O9P 500.9617 .(H2PO4Na)2.H(+) 0.630 3.824 2.408
   C00881  Deoxycytidine C9H13N3O4 228.1059 .H(+) 0.683 3.525 2.407
   2-Acyl-sn-glycero-3-phosphoglycerol (n-C16:0)  2-Acyl-sn-glycero-3-phosphoglycerol (n-C16:0) C22H45O9P1 507.2580 .H/Na.H(+) 0.636 3.779 2.403
   C01081  Thiamin monophosphate C12H17N4O4PS 616.9524 .(H2PO4K)2.H(+) 0.627 3.783 2.372
   C00275  D-Mannose 6-phosphate C6H13O9P 500.9617 .(H2PO4Na)2.H(+) 0.616 3.824 2.356
   C00095  D-Fructose C6H12O6 203.0529 .H/Na.H(+) 0.655 3.562 2.334
   C00079  L-Phenylalanine C9H11NO2 166.0861 .H(+) 0.610 3.786 2.311
   C11453  2-C-methyl-D-erythritol 2,4-cyclodiphosphate C5H12O9P2 496.9433 .(H2PO4)2NaH.H(+) 0.650 3.531 2.295
   C00163  Propionate (n-C3:0) C3H6O2 97.0277 .H/Na.H(+) 0.606 3.719 2.255
   C00243  Lactose C12H22O11 365.1070 .H/Na.H(+) 0.608 3.616 2.199
   2-Acyl-sn-glycero-3-phosphoglycerol (n-C18:0)  2-Acyl-sn-glycero-3-phosphoglycerol (n-C18:0) C24H49O9P1 551.2857 .H/K.H(+) 0.602 3.526 2.123
   C00364  dTMP C10H15N2O8P 323.0605 .H(+) 0.600 3.698 0.000
   C00052  UDPgalactose C15H24N2O17P2 838.9388 .(H2PO4K)2.H(+) 0.599 3.580 0.000
   C00062  L-Arginine C6H14N4O2 317.0584 .HPO4Na2.H(+) 0.596 7.048 0.000
   C00299  Uridine C9H12N2O6 246.0839 [+1].H(+) 0.596 4.871 0.000
   C06007  (R)-2,3-Dihydroxy-3-methylpentanoate C6H12O4 171.0611 .H/Na.H(+) 0.595 -4.150 -0.000
   C05235  Acetol C3H6O2 97.0277 .H/Na.H(+) 0.594 3.719 0.000
   C00054  Adenosine 3',5'-bisphosphate C10H15N5O10P2 465.9992 .H/K.H(+) 0.593 -3.565 -0.000
   C00054  Adenosine 3',5'-bisphosphate C10H15N5O10P2 450.0204 .H/Na.H(+) 0.592 -3.497 -0.000
   C05519  L-Allo-threonine C4H9NO3 142.0477 .H/Na.H(+) 0.591 4.762 0.000
   C00232  Succinic semialdehyde C4H6O3 85.0284 -H2O.H(+) 0.589 4.058 0.000
   C00243  Lactose C12H22O11 345.1336 [+2].H(+) 0.589 4.246 0.000
   C04666  D-erythro-1-(Imidazol-4-yl)glycerol 3-phosphate C6H11N2O6P 359.0022 .H2PO4Na.H(+) 0.588 3.892 0.000
   Hexadecanoyl-phosphate (n-C16:0)  Hexadecanoyl-phosphate (n-C16:0) C16H33O5P 457.1660 .H2PO4Na.H(+) 0.585 6.369 0.000
   C00062  L-Arginine C6H14N4O2 197.1003 .Na(+) 0.584 4.944 0.000
   C00062  L-Arginine C6H14N4O2 197.1003 .H/Na.H(+) 0.584 4.944 0.000
   C00188  L-Threonine C4H9NO3 142.0477 .H/Na.H(+) 0.584 4.762 0.000
   C00387  Guanosine C10H13N5O5 286.1061 [+2].H(+) 0.584 5.257 0.000
   C01888  Aminoacetone C3H7NO 74.0600 .H(+) 0.582 6.754 0.000
   C02291  L-Cystathionine C7H14N2O4S 359.0022 .H2PO4K.H(+) 0.582 3.892 0.000
   C00283  Hydrogen sulfide H2S 208.8846 .HPO4K2.H(+) 0.578 -4.472 -0.000
   C00361  dGDP C10H15N5O10P2 450.0204 .H/Na.H(+) 0.578 -3.497 -0.000
   C02976  D-Fructose 1-phosphate C6H13O9P 500.9617 .(H2PO4Na)2.H(+) 0.578 3.824 0.000
   C04442  2-Dehydro-3-deoxy-D-gluconate 6-phosphate C6H11O9P 280.9980 .H/Na.H(+) 0.576 7.357 0.000
   C00166  Phenylpyruvate C9H8O3 121.0630 -CO2.H(+) 0.574 3.738 0.000
   6-hydroxymethyl-dihydropterin pyrophosphate  6-hydroxymethyl-dihydropterin pyrophosphate C7H11N5O8P2 529.9139 .HPO4K2.H(+) 0.574 3.463 0.000
   octadecenoate (n-C18:1)  octadecenoate (n-C18:1) C18H34O2 419.1968 .H2PO4K.H(+) 0.573 5.616 0.000
   C00124  D-Galactose C6H12O6 203.0529 .H/Na.H(+) 0.572 3.562 0.000
   C00352  D-Glucosamine 6-phosphate C6H14NO8P 262.0564 [+2].H(+) 0.571 3.757 0.000
   C00740  D-Serine C3H7NO3 144.0080 .H/K.H(+) 0.571 7.034 0.000
   C00031  D-Glucose C6H12O6 203.0529 .H/Na.H(+) 0.568 3.562 0.000
   C00670  sn-Glycero-3-phosphocholine C8H20NO6P 280.0942 .H/Na.H(+) 0.567 6.058 0.000
   C00670  sn-Glycero-3-phosphocholine C8H20NO6P 280.0942 .Na(+) 0.567 6.058 0.000
   C00078  L-Tryptophan C11H12N2O2 206.1030 [+1].H(+) 0.567 7.162 0.000
   C01079  Protoporphyrinogen IX C34H40N4O4 607.2643 .H/K.H(+) 0.566 -3.826 -0.000
   C00937  D-Lactaldehyde C3H6O2 97.0277 .H/Na.H(+) 0.565 3.719 0.000
   C00065  L-Serine C3H7NO3 88.0387 -H2O.H(+) 0.565 6.080 0.000
   C00095  D-Fructose C6H12O6 323.0108 .HPO4Na2.H(+) 0.563 3.628 0.000
   C00097  L-Cysteine C3H7NO2S 144.0080 .H/Na.H(+) 0.562 7.034 0.000
   C01909  Dethiobiotin C10H18N2O3 357.0776 .HPO4Na2.H(+) 0.561 12.453 0.000
   C15700  gamma-glutamyl-gamma-butyraldehyde C9H16O4N2 219.1293 [+2].H(+) 0.561 12.524 0.000
   tetradecenoate (n-C14:1)  tetradecenoate (n-C14:1) C14H26O2 369.1368 .HPO4Na2.H(+) 0.559 4.808 0.000
   C00064  L-Glutamine C5H10N2O3 130.0498 -NH3.H(+) 0.558 3.482 0.000
   C00424  L-Lactaldehyde C3H6O2 97.0277 .H/Na.H(+) 0.557 3.719 0.000
   C00740  D-Serine C3H7NO3 88.0387 -H2O.H(+) 0.557 6.080 0.000
   C01250  N-Acetyl-L-glutamate 5-semialdehyde C7H11NO4 175.0833 [+1].H(+) 0.555 4.212 0.000
   C02730  o-Succinylbenzoate C11H10O5 225.0654 [+2].H(+) 0.553 4.248 0.000
   C00487  D-Carnitine C7H15NO3 144.1004 -H2O.H(+) 0.550 3.908 0.000
   C04114  crotonobetaine C7H13NO2 166.0861 .H/Na.H(+) 0.550 3.786 0.000
   C02225  2-Methylcitrate C7H10O7 207.0518 .H(+) 0.549 4.383 0.000
   C00092  D-Glucose 6-phosphate C6H13O9P 500.9617 .(H2PO4Na)2.H(+) 0.545 3.824 0.000
   C00021  S-Adenosyl-L-homocysteine C14H20N6O5S 386.1308 [+1].H(+) 0.545 5.868 0.000
   C01286  2-Dehydro-3-deoxy-D-galactonate 6-phosphate C6H11O9P 280.9980 .H/Na.H(+) 0.544 7.357 0.000
   C00103  D-Glucose 1-phosphate C6H13O9P 500.9617 .(H2PO4Na)2.H(+) 0.544 3.824 0.000
   C00078  L-Tryptophan C11H12N2O2 243.0554 .H/K.H(+) 0.539 4.599 0.000
   C04114  crotonobetaine C7H13NO2 144.1004 .H(+) 0.539 3.908 0.000
   C00208  Maltose C12H22O11 365.1070 .H/Na.H(+) 0.538 3.616 0.000
   C00065  L-Serine C3H7NO3 106.0499 .H(+) 0.537 37.505 0.000
   C00575  cAMP C10H12N5O6P 450.0204 .H2PO4Na.H(+) 0.537 -3.497 -0.000
   C00031  D-Glucose C6H12O6 323.0108 .HPO4Na2.H(+) 0.532 3.628 0.000
   C01487  D-Allose C6H12O6 323.0108 .HPO4Na2.H(+) 0.532 3.628 0.000
   C00078  L-Tryptophan C11H12N2O2 205.0969 .H(+) 0.532 7.269 0.000
   Octadecanoyl-phosphate (n-C18:0)  Octadecanoyl-phosphate (n-C18:0) C18H37O5P 539.1279 .HPO4K2.H(+) 0.531 4.487 0.000
   C00085  D-Fructose 6-phosphate C6H13O9P 500.9617 .(H2PO4Na)2.H(+) 0.530 3.824 0.000
   C00446  alpha-D-Galactose 1-phosphate C6H13O9P 500.9617 .(H2PO4Na)2.H(+) 0.529 3.824 0.000
   C00147  Adenine C5H5N5 369.9664 .(H2PO4)2KH.H(+) 0.528 -4.323 -0.000
   C04593  methylisocitrate C7H10O7 207.0518 .H(+) 0.527 4.383 0.000
   C00007  O2 O2 206.8865 .HPO4K2.H(+) 0.526 -7.073 -0.000
   C00047  L-Lysine C6H14N2O2 129.1014 -H2O.H(+) 0.525 6.084 0.000
   C00055  CMP C9H14N3O8P 465.9992 .HPO4Na2.H(+) 0.525 -3.565 -0.000
   C00740  D-Serine C3H7NO3 106.0499 .H(+) 0.523 37.505 0.000
   C00407  L-Isoleucine C6H13NO2 132.1024 .H(+) 0.521 3.876 0.000
   C00062  L-Arginine C6H14N4O2 176.1217 [+1].H(+) 0.520 5.019 0.000
   C03415  N2-Succinyl-L-ornithine C9H16N2O5 257.1009 [+2].Na(+) 0.519 3.849 0.000
   C05512  Deoxyinosine C10H12N4O4 373.0488 .H2PO4Na.H(+) 0.512 6.323 0.000
   C06424  tetradecanoate (n-C14:0) C14H28O2 229.2152 .H(+) 0.510 10.124 0.000
   C00062  L-Arginine C6H14N4O2 177.1227 [+2].H(+) 0.509 4.068 0.000
   C01487  D-Allose C6H12O6 203.0529 .H/Na.H(+) 0.509 3.562 0.000
   C00049  L-Aspartate C4H7NO4 134.0449 .H(+) 0.505 4.766 0.000
   C00124  D-Galactose C6H12O6 323.0108 .HPO4Na2.H(+) 0.501 3.628 0.000
   C00214  Thymidine C10H14N2O5 243.0990 .H(+) 0.500 7.417 0.000
   C00019  S-Adenosyl-L-methionine C15H23N6O5S 382.1433 -H2O.H(+) 0.500 3.782 0.000
   C00123  L-Leucine C6H13NO2 132.1024 .H(+) 0.499 3.876 0.000
   C01083  Trehalose C12H22O11 365.1070 .H/Na.H(+) 0.498 3.616 0.000
   C00078  L-Tryptophan C11H12N2O2 188.0692 -NH3.H(+) 0.493 5.687 0.000
   C00318  L-Carnitine C7H15NO3 144.1004 -H2O.H(+) 0.493 3.908 0.000
   tetradecenoate (n-C14:1)  tetradecenoate (n-C14:1) C14H26O2 265.1570 .H/K.H(+) 0.489 3.506 0.000
   tetradecenoate (n-C14:1)  tetradecenoate (n-C14:1) C14H26O2 265.1570 .K(+) 0.489 3.506 0.000
   C00114  Choline C5H13NO 104.1064 .H(+) 0.487 -7.993 -0.000
   C15998  L-methionine-R-sulfoxide C5H11NO3S 188.0386 .H/Na.H(+) 0.482 4.254 0.000
   C00159  D-Mannose C6H12O6 323.0108 .HPO4Na2.H(+) 0.482 3.628 0.000
   C04294  4-Methyl-5-(2-hydroxyethyl)-thiazole C6H9NOS 146.0566 [+2].H(+) 0.481 3.579 0.000
   C05402  Melibiose C12H22O11 365.1070 .H/Na.H(+) 0.481 3.616 0.000
   C00364  dTMP C10H15N2O8P 496.9433 .HPO4K2.H(+) 0.479 3.531 0.000
   C00065  L-Serine C3H7NO3 144.0080 .H/K.H(+) 0.479 7.034 0.000
   C00361  dGDP C10H15N5O10P2 465.9992 .H/K.H(+) 0.478 -3.565 -0.000
   C05402  Melibiose C12H22O11 345.1336 [+2].H(+) 0.478 4.246 0.000
   C00596  2-Oxopent-4-enoate C5H6O3 116.0372 [+1].H(+) 0.477 4.791 0.000
   C05775  N1-(alpha-D-ribosyl)-5,6-dimethylbenzimidazole C14H18N2O4 399.0909 .H2PO4Na.H(+) 0.477 4.144 0.000
   C00957  Mercaptopyruvate C3H4O3S 294.8835 .HPO4K2.H(+) 0.473 -4.524 -0.000
   C00183  L-Valine C5H11NO2 74.0968 -CO2.H(+) 0.471 5.294 0.000
   C02989  L-Methionine Sulfoxide C5H11NO3S 166.0509 .H(+) 0.470 9.709 0.000
   C00979  O-Acetyl-L-serine C5H9NO4 148.0605 .H(+) 0.464 3.908 0.000
   C00641  1,2-Diacyl-sn-glycerol (dihexadec-9-enoyl, n-C16:1) C35H64O5 701.4083 .H2PO4K.H(+) 0.451 5.037 0.000
   C00029  UDPglucose C15H24N2O17P2 838.9388 .(H2PO4K)2.H(+) 0.440 3.580 0.000
   C00725  Lipoate C8H14O2S2 207.0518 .H(+) 0.439 4.383 0.000
   C03287  L-Glutamate 5-phosphate C5H10NO7P 369.9664 .HPO4Na2.H(+) 0.439 -4.323 -0.000
   C03733  UDP-D-galacto-1,4-furanose C15H24N2O17P2 838.9388 .(H2PO4K)2.H(+) 0.439 3.580 0.000
   C00596  2-Oxopent-4-enoate C5H6O3 97.0277 -H2O.H(+) 0.426 3.719 0.000
   C01672  1,5-Diaminopentane C5H14N2 104.1261 [+1].H(+) 0.426 -4.811 -0.000
   C05775  N1-(alpha-D-ribosyl)-5,6-dimethylbenzimidazole C14H18N2O4 279.1339 .H(+) 0.422 13.784 0.000
   C18096  Allulose 6-phosphate C6H13O9P 500.9617 .(H2PO4Na)2.H(+) 0.418 3.824 0.000
   3-Aminoacrylate  3-Aminoacrylate C3H5NO2 88.0387 .H(+) 0.333 6.080 0.000
   6-hydroxymethyl dihydropterin  6-hydroxymethyl dihydropterin C7H9N5O2 369.9664 .HPO4K2.H(+) 0.000 -4.323 -0.000
   C00217  D-Glutamate C5H9NO4 130.0498 -H2O.H(+) 0.000 3.482 0.000
   C00217  D-Glutamate C5H9NO4 148.0605 .H(+) 0.000 3.908 0.000
   C00931  Porphobilinogen C10H14N2O4 227.1064 .H(+) 0.000 9.337 0.000
   C00931  Porphobilinogen C10H14N2O4 228.1059 [+1].H(+) 0.000 3.525 0.000
   C01177  1D-myo-Inositol 1-phosphate C6H13O9P 500.9617 .(H2PO4Na)2.H(+) 0.000 3.824 0.000
   C04332  6,7-Dimethyl-8-(1-D-ribityl)lumazine C13H18N4O6 327.1241 .H(+) 0.000 5.026 0.000
   C04454  5-Amino-6-(5'-phosphoribitylamino)uracil C9H17N4O9P 357.0776 .H(+) 0.000 12.453 0.000
   Fe(III)hydoxamate, unloaded  Fe(III)hydoxamate, unloaded C9H21O6N3 270.1596 [+2].H(+) 0.000 7.778 0.000
   C00575  cAMP C10H12N5O6P 465.9992 .H2PO4K.H(+) 0.612 -3.565 -2.182
   C00362  dGMP C10H14N5O7P 386.0301 .H/K.H(+) 0.628 -3.802 -2.387
   C00475  Cytidine C9H13N3O5 386.0301 .HPO4Na2.H(+) 0.633 -3.802 -2.408
   C02876  Propanoyl phosphate C3H7O5P 157.0156 [+2].H(+) 0.667 -3.793 -2.530
   C01181  gamma-butyrobetaine C7H15NO2 386.0301 .(H2PO4Na)2.H(+) 0.701 -3.802 -2.664
   C05932  N2-Succinyl-L-glutamate 5-semialdehyde C9H13NO6 465.9992 .(H2PO4)2KH.H(+) 0.748 -3.565 -2.667
   C14179  sulfoacetate C2H4O5S 122.9755 -H2O.H(+) 0.640 -4.234 -2.709
   C00522  (R)-Pantoate C6H12O4 171.0611 .H/Na.H(+) 0.686 -4.150 -2.848
   C05932  N2-Succinyl-L-glutamate 5-semialdehyde C9H13NO6 450.0204 .(H2PO4)2NaH.H(+) 0.828 -3.497 -2.897
   C11145  methanesulfonate CH4O3S 238.9349 .HPO4Na2.H(+) 0.734 -4.170 -3.060
   C00108  Anthranilate C7H7NO2 176.0081 .H/K.H(+) 0.817 -3.893 -3.179
   C00568  4-Aminobenzoate C7H7NO2 176.0081 .H/K.H(+) 0.820 -3.893 -3.192
   C06056  4-Hydroxy-L-threonine C4H9NO4 369.9664 .(H2PO4)2KH.H(+) 0.802 -4.323 -3.467
   C00114  Choline C5H13NO 105.1101 [+1].H(+) 0.641 -5.725 -3.671
   C00704  Superoxide anion O2 206.8865 .HPO4K2.H(+) 0.636 -7.073 -4.495
     KEGG pathway by CLR  
   Pathway_ion pvalue_ion qvalue_ion  One carbon pool by folate 0 0.0000
  Lipoic acid metabolism 0 0.0000
  Aminoacyl-tRNA biosynthesis 1e-08 0.0000
  Microbial metabolism in diverse environments 4e-08 0.0000
  ABC transporters 6e-08 0.0000
  Biosynthesis of secondary metabolites 4e-07 0.0000
  Glycine, serine and threonine metabolism 1e-06 0.0000
  D-Glutamine and D-glutamate metabolism 1e-06 0.0000
  C5-Branched dibasic acid metabolism 2e-06 0.0000
  Arachidonic acid metabolism 2e-06 0.0000
  Ethylbenzene degradation 4e-06 0.0000
  Cysteine and methionine metabolism 1e-05 0.0001
  Arginine and proline metabolism 2e-05 0.0001
  Propanoate metabolism 0.0003 0.0010
  Bacterial chemotaxis 0.0005 0.0018
  Valine, leucine and isoleucine biosynthesis 0.001 0.0034
  Valine, leucine and isoleucine degradation 0.002 0.0052
  Naphthalene degradation 0.002 0.0060
  Nitrogen metabolism 0.003 0.0085
  Sulfur metabolism 0.003 0.0081
  Ubiquinone and other terpenoid-quinone biosynthesis 0.004 0.0096
  Phenylalanine metabolism 0.005 0.0116
  Alanine, aspartate and glutamate metabolism 0.005 0.0124
  Chlorocyclohexane and chlorobenzene degradation 0.01 0.0219
     COG enrichment  
   Pathway_MS pvalue_MS qvalue_MS  Aminoacyl-tRNA biosynthesis 0.002 0.1312
  Nucleotide excision repair 0.002 0.0656
  DNA replication 0.004 0.0993
  D-Alanine metabolism 0.004 0.0827
  Terpenoid backbone biosynthesis 0.004 0.0662
     Predicted metabolites from CLR  
   Predicted metabolites Pvalue Overlap with hits  2-(Formamido)-N1-(5-phospho-D-ribosyl)acetamidine 0 0.0000
  Orotidine 5'-phosphate 0 0.0000
  5-Phospho-beta-D-ribosylamine 0 0.0000
  N2-Formyl-N1-(5-phospho-D-ribosyl)glycinamide 4e-05 0.0000
  N1-(5-Phospho-D-ribosyl)glycinamide 4e-05 0.0000
  L-Lactaldehyde 0.0002 1.0000
  L-alanine-D-glutamate-meso-2,6-diaminoheptanedioate 0.0003 0.0000
  Choline 0.002 1.0000
  L-alanine-D-glutamate-meso-2,6-diaminoheptanedioate-D-alanine 0.003 0.0000
  L-Tryptophan 0.004 1.0000
  Citrate 0.007 0.0000
  Hydrogen peroxide 0.009 0.0000
  5-Phospho-alpha-D-ribose 1-diphosphate 0.009 0.0000
    
 
