# Supplementary material for: Genomewide landscape of gene–metabolome associations in Escherichia coli
Source: Mol Syst Biol. 2017 Jan 16;13(1):907. doi: 10.15252/msb.20167150 (PMC5293155; doi:10.15252/msb.20167150)
Supplement: Supplementary file 4 — Table EV3 [file MSB-13-907-s004.zip › details/data_yafZ.html]

 
 
 yafZ 
  yafZ - details 
 
 
  CLR  
   Gene_matching CLR_index  yagB 15.2
  ykfB 13.0
  yahG 11.7
  yiaU 11.0
  coaE 10.6
  yehI 10.1
  pepE 9.5
  crp 9.4
  yjeK 9.4
  yddL 9.2
  gspO 9.0
  hcaT 8.9
  flgK 8.6
  yehA 8.1
  yagU 8.0
  yagZ 8.0
  pck 7.7
  hemX 7.7
  ybdJ 7.5
  ygeR 7.5
  ycbU 7.4
  pfkA 7.4
  yneG 7.3
  ynbE 7.3
  gadX 7.2
  yfcD 7.1
  yeaT 7.1
  fldB 7.1
  yneK 7.0
  fkpA 6.9
  ygeL 6.9
  ybcN 6.7
  yjfL 6.6
  fhuC 6.6
  yahO 6.5
  pgm 6.5
  osmE 6.4
  mrcB 6.4
  pflA 6.3
  yeiM 6.3
  nlpE 6.2
  yjfK 6.2
  yaiO 6.0
  flhA 6.0
  yfcV 6.0
  stpA 5.9
  hflC 5.9
  ushA 5.9
  dnaJ 5.8
  yqeG 5.7
  treA 5.7
  pepB 5.7
  melR 5.7
  yjdF 5.7
  yfiM 5.7
  ilvB 5.7
  narZ 5.7
  yohL 5.6
  aroP 5.6
  ykfA 5.6
  yifK 5.6
  panC 5.5
  betI 5.5
  yhbS 5.5
  ilvY 5.4
  ydgK 5.4
  secB 5.3
  yhhW 5.3
  rhtA 5.3
  pal 5.3
  evgS 5.2
  yacH 5.2
  gcd 5.2
  cof 5.2
  ydfZ 5.1
  nadA 5.0
  agaS 5.0
  ymfR 5.0
  etp 5.0
  yfiR 4.9
  yjfJ 4.9
  yfbH 4.9
  yafW 4.8
  flgF 4.8
  hsdS 4.8
  ygeF 4.8
  ynfG 4.8
  ilvA 4.8
  yedP 4.8
  yeiT 4.7
  htpX 4.7
  fadD 4.7
  cysH 4.7
  tnaA 4.6
  betT 4.6
  holD 4.6
  treC 4.5
  yfaQ 4.5
  flgE 4.5
  flhB 4.5
  pflC 4.4
  rpoN 4.4
  yiiF 4.4
  dnaQ 4.4
  asnC 4.4
  yccV 4.3
  bipA 4.3
  uxuB 4.3
  metL 4.3
  codB 4.2
  cmtA 4.2
  rarD 4.2
  yhcO 4.1
  yhbE 4.1
  ydiY 4.1
  ydaF 4.1
  ccmF 4.0
  yfcI 4.0
  yjiO 4.0
  yfjL 4.0
  yddG 4.0
  ccmH 4.0
  ygcN 3.9
  yhgA 3.9
  ssnA 3.9
  glf 3.9
  tam 3.8
  yigI 3.8
  yahC 3.8
  ymcA 3.8
  pdhR 3.8
  yecA 3.8
  ilvM 3.8
  yagW 3.8
  thiC 3.8
  yaiI 3.8
  ybfF 3.7
  cysZ 3.7
  zitB 3.7
  ycdP 3.7
  ychQ 3.7
  fucP 3.7
  ada 3.6
  yaaX 3.6
  ybgS 3.6
  ybbN 3.6
  yagP 3.6
  srlE 3.6
  ybjX 3.6
  nac 3.6
  ydhA 3.5
  yfdO 3.5
  yfiD 3.5
  yjhX 3.5
  adiY 3.5
  ybiT 3.5
  yieP 3.5
  nanA 3.5
  macB 3.5
  tsr 3.5
  trkD 3.5
  flgA 3.4
  eutL 3.4
  feaR 3.4
  yedK 3.4
  pdxJ 3.3
  ybcQ 3.3
  dmsB 3.3
  ychA 3.3
  cchA 3.3
  aceB 3.3
  yfgB 3.3
  yneJ 3.3
  yfdE 3.2
  gldA 3.2
  ycdM 3.2
  ggt 3.2
  panB 3.2
  yhaM 3.2
  cyoB 3.2
  sgcB 3.1
  sfmC 3.1
  wbbH 3.1
  yeeW 3.1
  osmC 3.1
  intR 3.1
  ybgC 3.1
  creB 3.1
  gspJ 3.1
  srlB 3.1
  rnb 3.0
  yciT 3.0
  zntA 3.0
  ptsP 3.0
  asnA 3.0
  fixB 3.0
  ybgA 3.0
  kefA 3.0
  csgD 3.0
  yhjC 3.0
  fdnH 3.0
     Differential ions  
   id name formula mz mod AUC Z-score Z-score AUC Weighted   C00242  Guanine C5H5N5O 152.0459 [+2]-H(+) 0.641 5.709 3.660
   C08362  Hexadecenoate (n-C16:1) C16H30O2 271.2288 +OH(-) 0.633 5.342 3.384
   C07086  Phenylacetic acid C8H8O2 376.9817 .(H2PO4Na)2.H(+) 0.790 3.842 3.037
   1,6-anhydrous-N-Acetylmuramate  1,6-anhydrous-N-Acetylmuramate C11H17NO7 275.1011 [+1]-H(+) 0.767 3.953 3.033
   C00212  Adenosine C10H13N5O4 286.1039 [+2]+OH(-) 0.652 4.088 2.666
   C00330  Deoxyguanosine C10H13N5O4 286.1039 [+2]+OH(-) 0.642 4.088 2.623
   C05973  2-Acyl-sn-glycero-3-phosphoethanolamine (n-C16:0) C21H44NO7P1 455.3024 [+1].H(+) 0.670 3.878 2.598
   C03175  Shikimate 5-phosphate C7H11O8P 524.8640 .(H2PO4K)2-H(+) 0.554 3.740 0.000
   C01551  Allantoin C4H6N4O3 376.9817 .(H2PO4)2NaH.H(+) 0.537 3.842 0.000
   C00460  dUTP C9H15N2O14P3 738.8357 .(H2PO4K)2-H(+) 0.532 4.231 0.000
   C00119  5-Phospho-alpha-D-ribose 1-diphosphate C5H13O14P3 524.8640 .H2PO4K-H(+) 0.526 3.740 0.000
   C04732  4-(1-D-Ribitylamino)-5-aminouracil C9H16N4O6 275.1011 -H(+) 0.000 3.953 0.000
     KEGG pathway by CLR  
   Pathway_ion pvalue_ion qvalue_ion  Phosphonate and phosphinate metabolism 5e-05 0.0049
  Riboflavin metabolism 7e-05 0.0037
  Methane metabolism 0.0006 0.0207
     COG enrichment  
   Pathway_MS pvalue_MS qvalue_MS  Pantothenate and CoA biosynthesis 0.0002 0.0141
  Flagellar assembly 0.002 0.0908
  Cyanoamino acid metabolism 0.003 0.0786
  Arachidonic acid metabolism 0.003 0.0632
  Fructose and mannose metabolism 0.009 0.1667
  C5-Branched dibasic acid metabolism 0.01 0.1465
  DNA replication 0.01 0.1256
     Predicted metabolites from CLR  
   Predicted metabolites Pvalue Overlap with hits  2-Oxobutanoate 2e-05 0.0000
  Cadmium 0.0002 0.0000
  L-Tryptophan 0.0004 0.0000
  Co2+ 0.0006 0.0000
  4-Amino-2-methyl-5-phosphomethylpyrimidine 0.001 0.0000
  L-Homoserine 0.001 0.0000
  L-Phenylalanine 0.001 0.0000
  D-Sorbitol 6-phosphate 0.001 0.0000
  L-Tyrosine 0.002 0.0000
  Zinc 0.002 0.0000
  Formate 0.003 0.0000
  Propanoyl-CoA 0.004 0.0000
  Dimethyl sulfide 0.006 0.0000
  Dimethyl sulfoxide 0.006 0.0000
  nickel 0.006 0.0000
  trimethylamine 0.006 0.0000
  Trimethylamine N-oxide 0.006 0.0000
    
 
