# Supplementary material for: Genomewide landscape of gene–metabolome associations in Escherichia coli
Source: Mol Syst Biol. 2017 Jan 16;13(1):907. doi: 10.15252/msb.20167150 (PMC5293155; doi:10.15252/msb.20167150)
Supplement: Supplementary file 4 — Table EV3 [file MSB-13-907-s004.zip › details/data_yagE.html]

 
 
 yagE 
  yagE - details 
 
 
  CLR  
   Gene_matching CLR_index  yegJ 7.6
  ydfO 6.9
  ynjC 6.9
  nadR 6.4
  pbl 6.4
  ymfN 6.4
  ppdA 6.3
  ycfJ 6.2
  yqeK 6.1
  yeaH 6.0
  yfcQ 6.0
  hinT 6.0
  ecpD 5.9
  mviM 5.8
  ynbC 5.8
  glcG 5.8
  sufD 5.7
  ydeP 5.6
  ompG 5.4
  ycaQ 5.4
  relE 5.3
  narU 5.3
  yjhS 5.3
  ydeO 5.3
  yobF 5.3
  ycjD 5.3
  yeeO 5.2
  ydeH 5.2
  yjiJ 5.1
  crcA 5.1
  nlpA 5.1
  yaiT 5.1
  yfbT 5.1
  ybiR 5.0
  yfcS 5.0
  yjbB 5.0
  yagT 5.0
  ybaJ 5.0
  hokD 5.0
  yfeW 4.9
  iaaA 4.9
  ydeR 4.9
  ydhZ 4.9
  ynjB 4.9
  phnH 4.8
  yeeT 4.8
  yibF 4.8
  yhhI 4.8
  mdtI 4.7
  yahN 4.7
  nudD 4.7
  yfdC 4.6
  yehU 4.6
  yohO 4.6
  kil 4.6
  lsrG 4.5
  ygaY 4.5
  rsxC 4.5
  yecT 4.5
  rhsD 4.5
  otsA 4.5
  ychE 4.5
  eamA 4.4
  gmhB 4.4
  wbbK 4.4
  ypjC 4.4
  gadE 4.4
  clcB 4.4
  mltC 4.4
  nikC 4.3
  yeiA 4.3
  yfdP 4.3
  phnL 4.3
  yfcU 4.3
  mioC 4.3
  hokA 4.3
  yfbE 4.3
  ygfI 4.3
  wbbI 4.2
  yfjD 4.2
  ybbC 4.2
  hybA 4.2
  yehT 4.2
  sgbE 4.2
  ydeV 4.2
  sfcA 4.2
  clpB 4.2
  ygcU 4.2
  yeaD 4.1
  mutS 4.1
  yedY 4.1
  yeaN 4.1
  yggS 4.1
  uspF 4.1
  ycdW 4.1
  ygfJ 4.1
  ydiA 4.1
  yoaD 4.1
  ylcG 4.1
  ydeN 4.1
  rhsE 4.0
  potB 4.0
  abgA 4.0
  yadM 4.0
  dppB 4.0
  tbpA 3.9
  yagF 3.9
  xerC 3.9
  yegH 3.9
  yphG 3.9
  ydiK 3.9
  cueO 3.9
  yncG 3.9
  rhsA 3.9
  ydhB 3.9
  yjjB 3.9
  slyA 3.9
  yfcM 3.9
  ygfS 3.8
  manY 3.8
  tag 3.8
  flhD 3.8
  glpR 3.8
  yqhA 3.8
  sufB 3.7
  dnaG 3.7
  ygeK 3.7
  yjaA 3.7
  yiiL 3.7
  mdtA 3.7
  ycdC 3.7
  caiA 3.7
  yfiL 3.7
  mdtB 3.7
  ybgI 3.7
  agaW 3.7
  frlD 3.7
  yfeR 3.6
  yecD 3.6
  yfjZ 3.6
  yfeN 3.6
  aqpZ 3.6
  yebU 3.6
  thrL 3.6
  hyfI 3.6
  yfbJ 3.6
  gidA 3.6
  yagX 3.6
  yegI 3.6
  ydiI 3.5
  yegK 3.5
  gabT 3.5
  yeiW 3.5
  fucU 3.5
  yfdL 3.5
  rtcB 3.5
  yadH 3.5
  pfkB 3.5
  mngR 3.5
  yeiP 3.5
  ydiQ 3.5
  yphB 3.5
  pbpC 3.5
  acrA 3.5
  trmH 3.4
  gspD 3.4
  yidF 3.4
  yegD 3.4
  smtA 3.4
  yhdZ 3.4
  yhfT 3.4
  ydjY 3.4
  rfaZ 3.4
  yfeA 3.3
  astC 3.3
  mutM 3.3
  cyoE 3.3
  malP 3.3
  yagI 3.3
  nikR 3.3
  yeaP 3.3
  menB 3.3
  yodB 3.3
  ydjH 3.3
  yfhK 3.3
  bglH 3.3
  yehL 3.2
  ybhQ 3.2
  tdcE 3.2
  yfdS 3.2
  yecM 3.2
  sseA 3.2
  ydhO 3.2
  ppdB 3.2
  yigZ 3.2
  ygeQ 3.2
  yfgJ 3.2
  ygcR 3.2
  ybfP 3.1
  mutT 3.1
  yhjG 3.1
  yhcC 3.1
  trmC 3.1
  ybeH 3.1
  modA 3.1
  dam 3.1
  pabC 3.1
  phnE 3.1
  lrhA 3.1
  yqaC 3.1
  yajI 3.1
  ydfJ 3.1
  setB 3.1
  hisB 3.1
  yfjJ 3.0
  yegP 3.0
  wbbL 3.0
  recN 3.0
  ygcK 3.0
  yhdA 3.0
  pioO 3.0
  pinH 3.0
  ygcQ 3.0
  fixA 3.0
  apaG 3.0
  garL 3.0
  yqgC 3.0
  fucK 3.0
  gudD 3.0
  kptA 3.0
  rem 3.0
  yebW 3.0
  yodD 3.0
     Differential ions  
   id name formula mz mod AUC Z-score Z-score AUC Weighted   C00387  Guanosine C10H13N5O5 286.1061 [+2].H(+) 0.584 3.923 0.000
   C00416  1,2-dihexadecanoyl-sn-glycerol 3-phosphate C35H69O8P1 649.4762 .H(+) 0.509 -3.486 -0.000
     KEGG pathway by CLR  
none  COG enrichment  
none  Predicted metabolites from CLR  
   Predicted metabolites Pvalue Overlap with hits  glucosyl-O-acetyl-rhamanosyl-N-acetylglucosamyl-undecaprenyl diphosphate 0 0.0000
  5-Dehydro-4-deoxy-D-glucarate 0.0001 0.0000
  [4Fe-4S] iron-sulfur cluster 0.001 0.0000
  SufBCD with bound [4Fe-4S] cluster 0.001 0.0000
  [2Fe-1S] desulfurated iron-sulfur cluster 0.002 0.0000
  [2Fe-2S] iron-sulfur cluster 0.002 0.0000
  UDP 0.002 0.0000
  SufBCD with two bound [2Fe-2S] clusters 0.002 0.0000
  UDPglucose 0.002 0.0000
    
 
