# Supplementary material for: Genomewide landscape of gene–metabolome associations in Escherichia coli
Source: Mol Syst Biol. 2017 Jan 16;13(1):907. doi: 10.15252/msb.20167150 (PMC5293155; doi:10.15252/msb.20167150)
Supplement: Supplementary file 4 — Table EV3 [file MSB-13-907-s004.zip › details/data_yagF.html]

 
 
 yagF 
  yagF - details 
 
 
  CLR  
   Gene_matching CLR_index  ydfJ 7.6
  yncG 7.6
  yfjW 7.0
  ybiR 7.0
  yncC 7.0
  xerC 6.9
  dam 6.7
  ynbC 6.6
  ybaJ 6.5
  yeaH 6.4
  rem 6.4
  mutM 6.3
  ymfN 6.2
  relE 5.7
  yfcS 5.6
  ydhZ 5.6
  yegJ 5.6
  yoaD 5.5
  gidB 5.5
  pioO 5.5
  yfdC 5.5
  ycfJ 5.4
  yehD 5.3
  ydhB 5.3
  ybhQ 5.3
  yecT 5.2
  yfcQ 5.2
  abgA 5.1
  nudD 5.0
  yggU 5.0
  ybcL 4.9
  ydeH 4.9
  yebU 4.9
  rhsB 4.9
  yagT 4.9
  pfkB 4.9
  yegD 4.9
  ygaY 4.8
  ydiK 4.8
  agaW 4.7
  sseA 4.6
  otsA 4.6
  kil 4.6
  ydaG 4.5
  ydcI 4.5
  sufD 4.5
  yadH 4.4
  eamA 4.4
  ykiA 4.4
  yfhK 4.3
  artJ 4.3
  hokD 4.3
  wbbL 4.3
  lsrG 4.2
  ycjR 4.2
  yfdP 4.2
  crcA 4.2
  mdtI 4.2
  wbbK 4.2
  yfdS 4.1
  yjiW 4.1
  yfbE 4.1
  yaiT 4.1
  yfcU 4.1
  ydfO 4.1
  yhdY 4.1
  ycjZ 4.1
  recD 4.0
  yoaB 4.0
  ycdW 4.0
  ycfT 4.0
  ydeO 4.0
  yagE 3.9
  yqgB 3.9
  phnO 3.9
  ybjK 3.9
  mviM 3.9
  mppA 3.9
  yhdZ 3.8
  yehT 3.8
  ydeM 3.8
  yjhS 3.7
  ydiQ 3.7
  cueO 3.7
  gatR 3.7
  ydjH 3.7
  ompG 3.7
  ydeV 3.6
  rfaB 3.6
  yehU 3.6
  yeeT 3.6
  ydeN 3.6
  ydjY 3.6
  ynhG 3.6
  ybeH 3.6
  ydiI 3.6
  yohO 3.6
  gspD 3.6
  sgcX 3.5
  ybbC 3.5
  yiiL 3.5
  yeiP 3.5
  wbbI 3.5
  mutS 3.5
  sfcA 3.5
  yecN 3.5
  yagH 3.5
  ycdC 3.5
  yegK 3.5
  yeeO 3.5
  yeiA 3.5
  acrA 3.5
  yfcM 3.4
  recF 3.4
  uspF 3.4
  clcB 3.4
  yceF 3.4
  dcp 3.4
  ymdF 3.4
  yfiL 3.4
  yehR 3.4
  hinT 3.3
  yoaC 3.3
  fixA 3.3
  yfdL 3.3
  ycaQ 3.3
  yibF 3.3
  cutC 3.3
  iaaA 3.2
  ycgG 3.2
  yeaJ 3.2
  yegP 3.2
  vacJ 3.2
  ypfI 3.2
  bglH 3.2
  rfaZ 3.2
  yeaQ 3.2
  thiD 3.2
  dacD 3.2
  ygfI 3.2
  yjaA 3.2
  yfjS 3.2
  hycA 3.2
  ychE 3.1
  gidA 3.1
  yfgJ 3.1
  ygfS 3.1
  yhdA 3.1
  mdtA 3.1
  yjbB 3.1
  pinH 3.1
  hisM 3.1
  ynjC 3.1
  yeaD 3.1
  gabT 3.1
  yqhA 3.1
  marR 3.1
  mltC 3.1
  yhfT 3.1
  phnH 3.1
  exoX 3.1
  yaiU 3.1
  ydiA 3.1
  ygcU 3.0
  yeaP 3.0
  ybgI 3.0
  yhaC 3.0
  ylaC 3.0
  yfeA 3.0
  ygaM 3.0
  araC 3.0
  ampE 3.0
     Differential ions  
   id name formula mz mod AUC Z-score Z-score AUC Weighted   C00534  Pyridoxamine C8H12N2O2 207.0518 .H/K.H(+) 0.861 3.731 3.212
   C01097  D-Tagatose 6-phosphate C6H13O9P 500.9617 .(H2PO4Na)2.H(+) 0.769 4.078 3.137
   C00636  D-Mannose 1-phosphate C6H13O9P 500.9617 .(H2PO4Na)2.H(+) 0.732 4.078 2.985
   C02962  D-Allose 6-phosphate C6H13O9P 500.9617 .(H2PO4Na)2.H(+) 0.630 4.078 2.568
   C00275  D-Mannose 6-phosphate C6H13O9P 500.9617 .(H2PO4Na)2.H(+) 0.616 4.078 2.513
   C00445  5,10-Methenyltetrahydrofolate C20H22N7O6 495.1278 .H/K.H(+) 0.688 3.459 2.380
   C02976  D-Fructose 1-phosphate C6H13O9P 500.9617 .(H2PO4Na)2.H(+) 0.578 4.078 0.000
   C02225  2-Methylcitrate C7H10O7 207.0518 .H(+) 0.549 3.731 0.000
   C00092  D-Glucose 6-phosphate C6H13O9P 500.9617 .(H2PO4Na)2.H(+) 0.545 4.078 0.000
   C00103  D-Glucose 1-phosphate C6H13O9P 500.9617 .(H2PO4Na)2.H(+) 0.544 4.078 0.000
   C00085  D-Fructose 6-phosphate C6H13O9P 500.9617 .(H2PO4Na)2.H(+) 0.530 4.078 0.000
   C00446  alpha-D-Galactose 1-phosphate C6H13O9P 500.9617 .(H2PO4Na)2.H(+) 0.529 4.078 0.000
   C04593  methylisocitrate C7H10O7 207.0518 .H(+) 0.527 3.731 0.000
   C00725  Lipoate C8H14O2S2 207.0518 .H(+) 0.439 3.731 0.000
   C18096  Allulose 6-phosphate C6H13O9P 500.9617 .(H2PO4Na)2.H(+) 0.418 4.078 0.000
   C01177  1D-myo-Inositol 1-phosphate C6H13O9P 500.9617 .(H2PO4Na)2.H(+) 0.000 4.078 0.000
     KEGG pathway by CLR  
   Pathway_ion pvalue_ion qvalue_ion  Lipoic acid metabolism 0 0.0000
  Lysine biosynthesis 1e-06 0.0001
  Propanoate metabolism 8e-05 0.0027
  Pyruvate metabolism 0.002 0.0472
  Vitamin B6 metabolism 0.003 0.0528
     COG enrichment  
   Pathway_MS pvalue_MS qvalue_MS  Mismatch repair 0.004 0.3861
     Predicted metabolites from CLR  
   Predicted metabolites Pvalue Overlap with hits  glucosyl-O-acetyl-rhamanosyl-N-acetylglucosamyl-undecaprenyl diphosphate 0 0.0000
  UDP 6e-06 0.0000
  UDPglucose 6e-06 0.0000
  L-Arginine 0.001 0.0000
    
 
