# Supplementary material for: Genomewide landscape of gene–metabolome associations in Escherichia coli
Source: Mol Syst Biol. 2017 Jan 16;13(1):907. doi: 10.15252/msb.20167150 (PMC5293155; doi:10.15252/msb.20167150)
Supplement: Supplementary file 4 — Table EV3 [file MSB-13-907-s004.zip › details/data_yagX.html]

 
 
 yagX 
  yagX - details 
 
 
  CLR  
   Gene_matching CLR_index  nadR 10.6
  ylcG 9.1
  ydfO 9.0
  thrL 9.0
  rtcB 8.9
  glyS 8.4
  yphB 8.1
  pbl 7.6
  mutS 7.3
  ydhB 7.2
  yebS 7.2
  narU 7.2
  yaiL 7.0
  yhhI 6.9
  yeaN 6.8
  ypjC 6.7
  yidF 6.6
  xerD 6.6
  gutQ 6.4
  sseA 6.3
  ygcQ 6.3
  ubiH 6.2
  ydiQ 6.1
  ygeQ 6.1
  recN 6.1
  yqaC 6.0
  envC 6.0
  ygcR 6.0
  yfjD 5.9
  ypjA 5.9
  clcB 5.9
  ybfD 5.9
  yecD 5.8
  modA 5.8
  ynjC 5.8
  hinT 5.8
  yabP 5.7
  yigZ 5.6
  yfbT 5.6
  ygeK 5.6
  ydeO 5.6
  yccK 5.6
  recO 5.5
  ompG 5.5
  yeiP 5.4
  ynjB 5.4
  yphG 5.4
  yhgG 5.4
  ygdB 5.3
  ygeI 5.3
  yhfZ 5.3
  yfbJ 5.3
  yehL 5.2
  trmC 5.2
  yebB 5.2
  yciX 5.2
  yhdP 5.1
  gspD 5.1
  mdtA 5.1
  phnE 5.1
  rspB 5.1
  ung 5.1
  ygbF 5.1
  yebW 5.0
  rhsE 5.0
  malT 5.0
  yeeO 5.0
  gudD 4.9
  mltC 4.9
  sufB 4.9
  yfbE 4.9
  yecT 4.9
  metI 4.8
  yggS 4.8
  yodB 4.8
  yhhH 4.8
  yehT 4.7
  yhbO 4.7
  rhsA 4.7
  yfeA 4.6
  yfeW 4.6
  yfaZ 4.6
  kptA 4.6
  mutH 4.6
  udk 4.5
  nudD 4.5
  hyfI 4.5
  hemE 4.5
  ygcU 4.5
  ydeP 4.4
  yfeR 4.4
  ydcC 4.4
  flhD 4.3
  yqeH 4.3
  ydjY 4.3
  dinG 4.2
  gatR 4.2
  ybfO 4.2
  yedY 4.2
  ycdR 4.2
  slyA 4.2
  yohO 4.2
  yfgH 4.2
  yeaP 4.1
  pfkB 4.1
  otsA 4.1
  clpB 4.1
  rhsC 4.1
  ytfA 4.1
  yoaD 4.1
  ydhO 4.1
  ygcE 4.1
  yfdL 4.0
  deaD 4.0
  yegP 4.0
  yehU 4.0
  bdm 4.0
  ydeN 4.0
  yfcM 4.0
  glpR 4.0
  thrC 4.0
  yegX 3.9
  yfgJ 3.9
  fliR 3.9
  hybA 3.9
  yjbB 3.9
  mviM 3.9
  yfiP 3.9
  fixA 3.9
  eamA 3.9
  ppdB 3.9
  yebU 3.9
  yegH 3.9
  ycfJ 3.9
  pbpC 3.9
  ygaY 3.8
  lrhA 3.8
  yoaB 3.8
  fkpB 3.8
  yehR 3.8
  ydeH 3.8
  yqgB 3.8
  ygaT 3.8
  cyoE 3.8
  gltS 3.8
  yeaD 3.8
  yeaH 3.8
  hokA 3.7
  yecM 3.7
  tolC 3.7
  ybbC 3.7
  yhdZ 3.7
  yqeJ 3.7
  yhfT 3.7
  ycjD 3.7
  rpoS 3.7
  ygcK 3.6
  yecN 3.6
  marA 3.6
  yoaC 3.6
  yfiL 3.6
  rhsD 3.6
  yfhB 3.6
  rbbA 3.6
  yebV 3.6
  ypdH 3.6
  sfcA 3.6
  yagE 3.6
  mdoH 3.6
  pbpG 3.6
  fliJ 3.5
  ybgI 3.5
  ecpD 3.5
  yfeN 3.5
  yhdA 3.5
  uspF 3.5
  yidL 3.5
  frlD 3.5
  yobF 3.5
  yqeK 3.5
  yqhA 3.4
  yfcA 3.4
  ycjZ 3.4
  fbaB 3.4
  yibF 3.4
  proQ 3.4
  crcA 3.3
  dppB 3.3
  mioC 3.3
  yqiG 3.3
  ydgJ 3.3
  gadE 3.3
  ydjQ 3.3
  hokD 3.3
  phnL 3.3
  tag 3.3
  yfcQ 3.3
  smtA 3.3
  fabR 3.3
  sgcX 3.3
  ygcG 3.3
  yfaV 3.3
  prpB 3.3
  alsA 3.3
  ybeH 3.2
  ypdA 3.2
  malP 3.2
  wbbK 3.2
  bglH 3.2
  gnsB 3.2
  setB 3.2
  ybaJ 3.2
  ynjI 3.2
  ygfI 3.2
  yjgW 3.2
  gidA 3.2
  nikB 3.2
  sseB 3.1
  dsbB 3.1
  phnM 3.1
  ycaM 3.1
  yeiW 3.1
  yahN 3.1
  yjaA 3.1
  ygfS 3.1
  yfhK 3.1
  puuP 3.0
  lysU 3.0
  yegJ 3.0
  sufD 3.0
  ygeN 3.0
  marR 3.0
  yjgZ 3.0
  ydeV 3.0
  ybgF 3.0
  yncC 3.0
  yeeD 3.0
     Differential ions  
   id name formula mz mod AUC Z-score Z-score AUC Weighted   C06156  D-Glucosamine 1-phosphate C6H14NO8P 261.0535 [+1].H(+) 0.908 6.869 6.240
   C00352  D-Glucosamine 6-phosphate C6H14NO8P 261.0535 [+1].H(+) 0.720 6.869 4.948
   C04114  crotonobetaine C7H13NO2 100.1124 -CO2.H(+) 0.663 5.046 3.347
   C00299  Uridine C9H12N2O6 246.0839 [+1].H(+) 0.596 4.090 0.000
   C00059  Sulfate H2O4S 272.8685 .HPO4K2.H(+) 0.556 3.744 0.000
   Tetradecanoyl-phosphate (n-C14:0)  Tetradecanoyl-phosphate (n-C14:0) C14H29O5P 429.1521 .H2PO4Na.H(+) 0.515 3.879 0.000
   Glycerophosphoserine  Glycerophosphoserine C6H14NO8P 261.0535 [+1].H(+) 0.488 6.869 0.000
     KEGG pathway by CLR  
   Pathway_ion pvalue_ion qvalue_ion  Caprolactam degradation 0.0002 0.0206
  Biosynthesis of secondary metabolites 0.0009 0.0479
     COG enrichment  
   Pathway_MS pvalue_MS qvalue_MS  Aminoacyl-tRNA biosynthesis 0.007 0.7190
     Predicted metabolites from CLR  
   Predicted metabolites Pvalue Overlap with hits  [4Fe-4S] iron-sulfur cluster 0.0006 0.0000
  SufBCD with bound [4Fe-4S] cluster 0.0006 0.0000
  [2Fe-1S] desulfurated iron-sulfur cluster 0.0009 0.0000
  [2Fe-2S] iron-sulfur cluster 0.0009 0.0000
  D-Fructose 1,6-bisphosphate 0.0009 0.0000
  SufBCD with two bound [2Fe-2S] clusters 0.001 0.0000
  UDPglucose 0.001 0.0000
  GDP 0.01 0.0000
  UDP 0.01 0.0000
    
 
