# Supplementary material for: Genomewide landscape of gene–metabolome associations in Escherichia coli
Source: Mol Syst Biol. 2017 Jan 16;13(1):907. doi: 10.15252/msb.20167150 (PMC5293155; doi:10.15252/msb.20167150)
Supplement: Supplementary file 4 — Table EV3 [file MSB-13-907-s004.zip › details/data_yahC.html]

 
 
 yahC 
  yahC - details 
 
 
  CLR  
   Gene_matching CLR_index  yjeK 14.0
  metH 12.4
  metE 12.2
  ptsP 11.4
  holD 11.2
  cysH 11.1
  ypjL 10.9
  ilvB 10.8
  panC 10.7
  murP 10.6
  ygcN 10.5
  ybgA 10.5
  pdxJ 10.4
  yfaW 9.8
  nanA 9.8
  metB 9.7
  prpD 9.7
  ilvA 9.7
  mrcB 9.6
  rsgA 9.5
  rpoN 9.5
  ydiZ 9.5
  glmM 9.5
  yahK 9.4
  yjfJ 9.3
  ygeX 9.3
  chbC 9.1
  rnb 9.0
  coaE 8.9
  yfcZ 8.8
  pgm 8.7
  gadX 8.7
  cchB 8.7
  cof 8.7
  argH 8.5
  yjdF 8.5
  puuD 8.5
  uidA 8.3
  mak 8.2
  speG 8.2
  betT 8.2
  gspO 8.1
  metL 8.1
  yceD 8.1
  yfjR 8.1
  ppk 8.0
  cchA 8.0
  ygjR 7.9
  ndk 7.9
  pfkA 7.8
  purC 7.8
  rbfA 7.7
  rng 7.7
  yhbJ 7.7
  dnaQ 7.6
  efp 7.6
  nudB 7.5
  pgi 7.4
  yjeO 7.4
  lysC 7.4
  cysN 7.4
  ypfN 7.4
  pdxA 7.2
  yfaQ 7.2
  ymcA 7.2
  purK 7.2
  yjeP 7.1
  purH 7.1
  ydhF 7.1
  leuL 7.1
  yfgB 7.1
  ypdG 7.1
  yneG 7.1
  miaA 7.0
  yhhW 7.0
  ggt 6.9
  ykfA 6.9
  ilvM 6.9
  ilvE 6.9
  ygeR 6.8
  yeiQ 6.8
  feaR 6.8
  ydaS 6.8
  potA 6.8
  thiQ 6.8
  rffC 6.7
  asnA 6.7
  ydcF 6.7
  ydcY 6.7
  yigF 6.6
  yccZ 6.6
  exuT 6.6
  dacB 6.6
  ompR 6.5
  crcB 6.5
  aroH 6.5
  yqjB 6.4
  srlB 6.4
  ygcL 6.4
  moaB 6.4
  yieI 6.4
  panD 6.4
  yzgL 6.4
  yifK 6.3
  sfmC 6.3
  crp 6.3
  cysJ 6.2
  fadD 6.2
  rpiA 6.2
  ybdR 6.2
  yafU 6.2
  tpx 6.2
  cmtA 6.2
  hyaF 6.1
  yfhL 6.1
  ycdQ 6.1
  ysgA 6.1
  yfcD 6.1
  panB 6.1
  malI 6.1
  yibL 6.0
  fruK 6.0
  flgK 6.0
  yjeH 6.0
  yedP 6.0
  leuD 6.0
  spr 6.0
  gor 6.0
  mdtK 5.9
  zntA 5.8
  fhuF 5.8
  ydcJ 5.8
  yhdV 5.8
  envZ 5.8
  ybjD 5.8
  yeeS 5.8
  bcp 5.7
  uspE 5.7
  ascF 5.7
  yebT 5.7
  yciH 5.7
  yjhF 5.6
  cysC 5.6
  yajO 5.6
  dsrB 5.6
  sbmC 5.5
  htrE 5.5
  hsdS 5.5
  yjcH 5.5
  hybO 5.5
  rbsD 5.5
  yhbS 5.5
  moeB 5.5
  cysI 5.5
  yjfK 5.4
  yojI 5.4
  pepB 5.4
  cysG 5.4
  rfaQ 5.4
  proX 5.4
  ruvB 5.4
  yfcR 5.4
  nagC 5.4
  cbpA 5.3
  frvA 5.3
  ebgR 5.3
  ycfH 5.3
  yddL 5.3
  ygaU 5.2
  hemX 5.2
  kefC 5.1
  mutL 5.1
  yahO 5.1
  ybgC 5.0
  yqjD 5.0
  fldB 5.0
  tktA 5.0
  yfhM 5.0
  yfcV 5.0
  pal 5.0
  edd 5.0
  sdaC 4.9
  stpA 4.9
  ygfO 4.9
  flk 4.9
  nohA 4.9
  yrdB 4.9
  pepE 4.9
  yhbE 4.8
  ybcN 4.8
  yjfL 4.8
  treC 4.8
  gcl 4.8
  yagS 4.8
  secG 4.8
  dadX 4.7
  cysD 4.7
  yeaR 4.7
  hflC 4.7
  bcsC 4.7
  hofB 4.7
  ccmH 4.7
  aer 4.7
  blc 4.7
  ycjW 4.7
  trxA 4.7
  rihA 4.7
  mrcA 4.7
  yedK 4.7
  ulaB 4.6
  yjhP 4.6
  ydjG 4.6
  ulaC 4.6
  bioD 4.6
  entB 4.6
  paaY 4.6
  yehA 4.6
  wcaB 4.6
  dmsB 4.6
  ygfG 4.6
  yjbH 4.6
  yaiO 4.6
  yniC 4.6
  soxS 4.6
  djlC 4.6
  mgtA 4.6
  yjiH 4.5
  purE 4.5
  cpxP 4.5
  etp 4.5
  yhjR 4.5
  ycgH 4.5
  rep 4.5
  cyaY 4.5
  metC 4.5
  yjfM 4.5
  nlpE 4.5
  tnaA 4.5
  ilvY 4.5
  fhuB 4.4
  yhdN 4.4
  uraA 4.4
  yfcP 4.4
  ackA 4.4
  nlpI 4.4
  aroG 4.4
  yrbC 4.3
  pepQ 4.3
  mcrB 4.3
  yfiM 4.3
  eutL 4.3
  tiaE 4.3
  melR 4.2
  alaS 4.2
  ychF 4.2
  marR 4.2
  citG 4.2
  hdeA 4.2
  frlR 4.2
  agaS 4.2
  yedE 4.2
  ynbE 4.2
  wrbA 4.2
  fhuC 4.1
  ygiQ 4.1
  dgsA 4.1
  yqeG 4.1
  topB 4.1
  cysZ 4.1
  pdhR 4.1
  yicC 4.1
  yphD 4.1
  rbsK 4.1
  ygbN 4.1
  speA 4.0
  rfaH 4.0
  wbbH 4.0
  ygjN 4.0
  ubiG 4.0
  emrK 4.0
  yjdL 4.0
  ylcE 4.0
  prfB 4.0
  ynfM 4.0
  tolQ 3.9
  tig 3.9
  maoC 3.9
  rpmF 3.9
  sgcB 3.9
  envY 3.9
  yjcO 3.9
  holE 3.9
  speC 3.9
  sufC 3.9
  nhaA 3.9
  yhhM 3.8
  codB 3.8
  yjhC 3.8
  torY 3.8
  narZ 3.8
  macA 3.8
  yeeP 3.8
  flgE 3.8
  pdxB 3.8
  yafZ 3.8
  yagN 3.8
  yagB 3.8
  yjiO 3.8
  fes 3.8
  gshA 3.7
  fdoI 3.7
  pheP 3.7
  fucP 3.7
  yjjJ 3.7
  iadA 3.7
  yacH 3.7
  rfaL 3.7
  ybeQ 3.7
  fhlA 3.7
  yjgH 3.7
  yecC 3.7
  yqfA 3.7
  mbhA 3.7
  ydiY 3.7
  ydjQ 3.7
  rfaY 3.7
  yibG 3.6
  flgC 3.6
  yhjB 3.6
  yhcO 3.6
  adiC 3.6
  yjcS 3.6
  yjbJ 3.6
  yraP 3.6
  ygaW 3.6
  avtA 3.5
  yjfC 3.5
  astD 3.5
  tatD 3.5
  ybaX 3.5
  tolA 3.5
  ycgJ 3.5
  yieH 3.5
  yaaW 3.5
  yeiT 3.5
  ydhJ 3.5
  yciO 3.5
  yjhG 3.5
  torT 3.5
  norR 3.5
  pitB 3.4
  argI 3.4
  ydeS 3.4
  ygaD 3.4
  trmU 3.4
  fhuD 3.4
  yagZ 3.4
  glpC 3.4
  yodA 3.4
  yiaG 3.4
  cmk 3.4
  yohM 3.4
  yfaU 3.3
  yeaA 3.3
  yadN 3.3
  fliQ 3.3
  recE 3.3
  rffA 3.3
  ubiH 3.3
  dksA 3.3
  yeaG 3.3
  yecI 3.3
  ykfB 3.3
  ydiJ 3.3
  sucB 3.3
  yfcY 3.3
  yfiE 3.3
  ydhC 3.3
  udp 3.3
  yghW 3.3
  tfaD 3.3
  yeeW 3.3
  hyfB 3.2
  amiA 3.2
  dnaK 3.2
  yccC 3.2
  rfaJ 3.2
  kefA 3.2
  cysE 3.2
  sbcB 3.2
  deoR 3.2
  ybhT 3.2
  lysS 3.2
  yiaK 3.2
  yfiB 3.2
  mrr 3.2
  yfgC 3.2
  ytfJ 3.2
  yjaB 3.2
  yiaW 3.2
  nuoN 3.2
  hcaT 3.1
  ydfZ 3.1
  rlmB 3.1
  yfbM 3.1
  nrdH 3.1
  yfcO 3.1
  yhiJ 3.1
  yaaU 3.1
  prlC 3.1
  lipA 3.1
  aroP 3.1
  yffH 3.1
  rtn 3.1
  brnQ 3.1
  prpE 3.1
  kbl 3.1
  thiE 3.0
  rhtA 3.0
  yfjI 3.0
  rof 3.0
  yhgA 3.0
  dinF 3.0
  malF 3.0
  nrdF 3.0
  xdhB 3.0
  ygiH 3.0
  sufA 3.0
  yhaM 3.0
  recC 3.0
  rpsU 3.0
  hemF 3.0
     Differential ions  
   id name formula mz mod AUC Z-score Z-score AUC Weighted   Glycerophosphoserine  Glycerophosphoserine C6H14NO8P 276.0488 +OH(-) 0.676 3.903 2.638
   C06156  D-Glucosamine 1-phosphate C6H14NO8P 276.0488 +OH(-) 0.630 3.903 2.458
   C00352  D-Glucosamine 6-phosphate C6H14NO8P 276.0488 +OH(-) 0.588 3.903 0.000
   C00119  5-Phospho-alpha-D-ribose 1-diphosphate C5H13O14P3 606.8796 .(H2PO4)2NaH-H(+) 0.567 -3.718 -0.000
   C00536  Inorganic triphosphate H5O10P3 294.8523 .H/K-H(+) 0.558 3.549 0.000
   C00182  glycogen C6H10O5 143.0339 -H2O-H(+) 0.444 -3.668 -0.000
   branching glycogen  branching glycogen C6H10O5 143.0339 -H2O-H(+) 0.422 -3.668 -0.000
   C05817  2-Succinyl-6-hydroxy-2,4-cyclohexadiene-1-carboxylate C11H12O6 359.0166 .H2PO4Na-H(+) 0.375 3.488 0.000
   C01228  Guanosine 3',5'-bis(diphosphate) C10H17N5O17P4 775.8241 .HPO4K2-H(+) 0.790 -3.547 -2.800
   C16238  lipoyl-AMP C18H26N5O8PS2 572.0458 .H/K-H(+) 0.954 -4.361 -4.160
     KEGG pathway by CLR  
   Pathway_ion pvalue_ion qvalue_ion  Nicotinate and nicotinamide metabolism 0 0.0000
     COG enrichment  
   Pathway_MS pvalue_MS qvalue_MS  Chlorocyclohexane and chlorobenzene degradation 0 0.0000
  Fluorobenzoate degradation 0 0.0000
  Sulfur metabolism 7e-08 0.0000
  Pantothenate and CoA biosynthesis 0.0003 0.0034
  Biosynthesis of secondary metabolites 0.0005 0.0057
  Selenoamino acid metabolism 0.001 0.0105
  Pentose phosphate pathway 0.002 0.0133
  Valine, leucine and isoleucine biosynthesis 0.002 0.0185
  Purine metabolism 0.004 0.0255
  Mismatch repair 0.004 0.0265
  Fructose and mannose metabolism 0.004 0.0246
  Vitamin B6 metabolism 0.008 0.0418
  Cysteine and methionine metabolism 0.008 0.0402
     Predicted metabolites from CLR  
   Predicted metabolites Pvalue Overlap with hits  2-Octaprenyl-6-methoxyphenol 0 0.0000
  5-amino-1-(5-phospho-D-ribosyl)imidazole-4-carboxylate 0 0.0000
  5-phosphoribosyl-5-carboxyaminoimidazole 0 0.0000
  Adenosine 5'-phosphosulfate 0 0.0000
  Aerobactin 0 0.0000
  coprogen 0 0.0000
  Dephospho-CoA 0 0.0000
  dTDP-4-amino-4,6-dideoxy-D-galactose 0 0.0000
  Fe(III)hydroxamate 0 0.0000
  Ferrichrome 0 0.0000
  heptosyl-phospho-heptosyl-heptosyl-kdo2-lipidA 0 0.0000
  ferroxamine 0.0002 0.0000
  Hydrogen sulfide 0.0005 0.0000
  CDP 0.001 0.0000
  3-Methyl-2-oxobutanoate 0.001 0.0000
  Reduced riboflavin 0.001 0.0000
  Riboflavin 0.001 0.0000
  4-Phospho-L-aspartate 0.002 0.0000
  5-Methyltetrahydrofolate 0.002 0.0000
  beta-Alanine 0.002 0.0000
  L-Cystathionine 0.002 0.0000
  dihydrosirohydrochlorin 0.002 0.0000
  L-methionine-R-sulfoxide 0.002 0.0000
  Uroporphyrinogen III 0.002 0.0000
  L-Phenylalanine 0.003 0.0000
  GDP 0.004 0.0000
  dCDP 0.004 0.0000
  [4Fe-4S] iron-sulfur cluster 0.006 0.0000
  dATP 0.006 0.0000
  L-Homocysteine 0.006 0.0000
  L-Cysteine 0.007 0.0000
  2-Dehydro-3-deoxy-D-arabino-heptonate 7-phosphate 0.007 0.0000
  2-Methyl-4-amino-5-hydroxymethylpyrimidine diphosphate 0.007 0.0000
  Pyridoxine 5'-phosphate 0.007 0.0000
  O-Phospho-4-hydroxy-L-threonine 0.007 0.0000
  5,6,7,8-Tetrahydrofolate 0.01 0.0000
  [2Fe-2S] iron-sulfur cluster 0.01 0.0000
  D-Erythrose 4-phosphate 0.01 0.0000
    
 
