# Supplementary material for: Genomewide landscape of gene–metabolome associations in Escherichia coli
Source: Mol Syst Biol. 2017 Jan 16;13(1):907. doi: 10.15252/msb.20167150 (PMC5293155; doi:10.15252/msb.20167150)
Supplement: Supplementary file 4 — Table EV3 [file MSB-13-907-s004.zip › details/data_yahJ.html]

 
 
 yahJ 
  yahJ - details 
 
 
  CLR  
   Gene_matching CLR_index  emrE 17.1
  rho 16.3
  ogrK 15.9
  dkgA 14.6
  cspB 13.1
  lysU 11.6
  rsmC 11.5
  rpmE 11.4
  rimI 11.2
  marB 10.9
  ampG 10.8
  sieB 10.6
  fecB 10.6
  ppiA 10.4
  yhjH 10.2
  nmpC 10.2
  ygcM 10.1
  smpA 10.0
  msrA 10.0
  fecD 10.0
  yfgD 10.0
  cpdA 9.8
  livJ 9.5
  truB 9.5
  puuA 9.5
  nhoA 9.4
  tatC 9.1
  prmA 9.0
  yggL 9.0
  elbB 9.0
  tehB 8.9
  pqiB 8.8
  acrF 8.7
  tdcD 8.4
  hybF 8.4
  amiB 8.4
  panF 8.4
  yhfU 8.2
  nfrB 8.2
  hslR 8.1
  rusA 8.0
  ygaQ 7.9
  emrD 7.9
  ynfN 7.9
  yhaB 7.9
  dtd 7.8
  ssuD 7.7
  arsC 7.7
  yjgK 7.7
  yeaV 7.7
  manZ 7.6
  yjgJ 7.5
  pspD 7.5
  rfaE 7.4
  ugpC 7.4
  ybaA 7.3
  uspA 7.3
  yjhE 7.3
  ynfO 7.2
  fsr 7.2
  ybiN 7.2
  yhiK 7.2
  pcm 7.1
  rluA 7.0
  ptsA 7.0
  tufB 7.0
  wcaK 7.0
  pspA 7.0
  malM 7.0
  ydgJ 7.0
  yihV 7.0
  rzoR 6.9
  yhiO 6.9
  ynjD 6.8
  yrbA 6.8
  yjiY 6.7
  ymfT 6.7
  plsX 6.7
  ydjK 6.6
  gph 6.5
  rhoL 6.5
  ymcD 6.4
  rpmJ 6.4
  rtcB 6.4
  intF 6.4
  xdhC 6.3
  pspC 6.3
  rbbA 6.3
  yddA 6.3
  yegW 6.2
  htgA 6.2
  cspI 6.1
  rplI 6.1
  dmsA 6.1
  uhpC 6.1
  yiiT 6.1
  yhdL 6.1
  yoeB 6.0
  chpA 6.0
  yghE 6.0
  ykgC 6.0
  slyD 6.0
  rpsF 6.0
  yjcQ 5.8
  sbmA 5.8
  frmB 5.7
  ydiM 5.7
  yhdX 5.7
  yicG 5.7
  ccmA 5.7
  yrfA 5.6
  alx 5.6
  ybgF 5.6
  csiR 5.6
  yfeU 5.6
  yghQ 5.5
  rob 5.5
  yraK 5.5
  ygjP 5.4
  yheV 5.4
  ybiO 5.4
  ydfO 5.4
  amiC 5.4
  yhiQ 5.3
  nanT 5.3
  pheM 5.3
  ypjJ 5.2
  ygfK 5.2
  ygaX 5.2
  dhaH 5.2
  yegX 5.2
  rzpR 5.2
  yjcB 5.2
  frlA 5.1
  thiS 5.1
  yqeF 5.1
  bcsF 5.0
  garP 5.0
  yqiC 5.0
  yjbG 5.0
  ugpE 4.9
  yobH 4.9
  nupG 4.9
  yaeF 4.9
  ydiQ 4.9
  yjgM 4.9
  selB 4.9
  folP 4.9
  cspC 4.9
  borD 4.9
  yrhC 4.8
  ecnB 4.8
  ycbL 4.8
  rseC 4.8
  glpK 4.8
  glcB 4.8
  yphB 4.8
  yecG 4.8
  yjbF 4.8
  ydhX 4.7
  mmuP 4.7
  lar 4.7
  ygfT 4.7
  yjeI 4.7
  ynjB 4.6
  yjgW 4.6
  ampD 4.6
  intA 4.6
  cheY 4.6
  torD 4.6
  ynfK 4.6
  yjjA 4.6
  surE 4.5
  yfcE 4.5
  torI 4.5
  yeaN 4.5
  uidC 4.5
  puuP 4.5
  yebQ 4.5
  greB 4.5
  hokB 4.5
  yeaW 4.5
  ydcX 4.4
  tolB 4.4
  zapA 4.4
  ybhF 4.4
  eptA 4.4
  yedZ 4.4
  yphH 4.4
  paaD 4.3
  ssuC 4.3
  mngA 4.3
  rseB 4.3
  yjiD 4.3
  gntR 4.3
  ygjO 4.2
  tolR 4.2
  ycgF 4.2
  ybcC 4.2
  yhiL 4.2
  yebW 4.1
  eco 4.1
  rspA 4.1
  paaH 4.1
  ubiH 4.1
  yagV 4.1
  ytfT 4.1
  ycdN 4.1
  ynbA 4.0
  yahM 4.0
  thiF 4.0
  malX 4.0
  trpL 4.0
  rpmG 4.0
  ampC 4.0
  frdB 3.9
  sseB 3.9
  shiA 3.9
  uup 3.9
  yicR 3.9
  ycbQ 3.9
  cvpA 3.9
  yncK 3.9
  yeeO 3.9
  thrL 3.8
  dhaK 3.8
  yggR 3.8
  gnsB 3.8
  yhjY 3.8
  yphF 3.8
  ligT 3.8
  tauA 3.8
  fsaB 3.7
  yniD 3.7
  yeiG 3.7
  lysS 3.7
  ygfB 3.7
  ydhB 3.6
  glyS 3.6
  yncJ 3.6
  yccS 3.6
  lsrF 3.6
  ymgC 3.6
  ybbM 3.5
  yghS 3.5
  bglF 3.5
  yjbO 3.5
  gntX 3.5
  citF 3.5
  rhlE 3.5
  yjiV 3.5
  yojL 3.5
  nuoN 3.4
  surA 3.4
  yggJ 3.4
  ung 3.4
  yciW 3.4
  yehL 3.4
  ygeW 3.4
  ytjC 3.4
  rcsC 3.4
  ylcG 3.4
  sufS 3.4
  ydhO 3.4
  ybbY 3.4
  tnaC 3.4
  yeiS 3.3
  dgoD 3.3
  ykgL 3.3
  cstA 3.3
  ybfH 3.3
  ybiV 3.3
  wzzE 3.3
  htrC 3.3
  hslO 3.3
  yfjO 3.3
  ydhV 3.2
  cusF 3.2
  yieM 3.2
  yhdJ 3.2
  ycdR 3.2
  hokA 3.2
  pbpG 3.2
  yciA 3.2
  sugE 3.2
  nrfE 3.2
  fiu 3.2
  ycdG 3.2
  tonB 3.1
  ydaT 3.1
  ugpB 3.1
  iclR 3.1
  narQ 3.1
  yfhM 3.1
  sgcR 3.1
  nusB 3.1
  yjjZ 3.0
  ymdF 3.0
  nadR 3.0
  hisL 3.0
  yfhH 3.0
  marA 3.0
  ymgG 3.0
  ymfA 3.0
  pps 3.0
  tktB 3.0
  bisC 3.0
     Differential ions  
   id name formula mz mod AUC Z-score Z-score AUC Weighted   C00624  N-Acetyl-L-glutamate C7H11NO5 212.0537 .H/Na.H(+) 0.811 6.805 5.523
   C00624  N-Acetyl-L-glutamate C7H11NO5 212.0537 .Na(+) 0.811 6.805 5.523
   C01302  1-(2-Carboxyphenylamino)-1-deoxy-D-ribulose 5-phosphate C12H16NO9P 485.9974 .H2PO4K.H(+) 0.908 5.285 4.800
   C04302  N-(5-Phospho-D-ribosyl)anthranilate C12H16NO9P 485.9974 .H2PO4K.H(+) 0.850 5.285 4.489
   C00526  Deoxyuridine C9H12N2O5 212.0537 -NH3.H(+) 0.654 6.805 4.449
   C00153  Nicotinamide C6H6N2O 124.0600 [+1].H(+) 0.782 5.099 3.986
   C00546  Methylglyoxal C3H4O2 208.9510 .H2PO4K.H(+) 0.683 5.814 3.973
   C00469  Ethanol C2H6O 85.0068 .H/K.H(+) 0.782 5.032 3.935
   C00088  Nitrite HNO2 85.9628 .H/K.H(+) 0.666 5.771 3.846
   C00497  D-Malate C4H6O5 117.0183 -H2O.H(+) 0.811 4.434 3.594
   C00052  UDPgalactose C15H24N2O17P2 589.0434 .H/Na.H(+) 0.650 4.905 3.189
   C00580  Dimethyl sulfide C2H6S 85.0068 .H/Na.H(+) 0.633 5.032 3.184
   C01179  3-(4-Hydroxyphenyl)pyruvate C9H8O4 163.0385 -H2O.H(+) 0.755 4.174 3.149
   C00624  N-Acetyl-L-glutamate C7H11NO5 228.0226 .H/K.H(+) 0.697 4.503 3.140
   C14179  sulfoacetate C2H4O5S 140.9849 .H(+) 0.689 4.547 3.134
   C11145  methanesulfonate CH4O3S 216.9495 .H2PO4Na.H(+) 0.642 4.839 3.106
   C03287  L-Glutamate 5-phosphate C5H10NO7P 228.0226 .H(+) 0.663 4.503 2.984
   C00246  Butyrate (n-C4:0) C4H8O2 89.0596 .H(+) 0.704 4.039 2.842
   C00059  Sulfate H2O4S 234.9084 .H2PO4K.H(+) 0.689 4.103 2.826
   C00575  cAMP C10H12N5O6P 472.0007 .HPO4Na2.H(+) 0.735 3.654 2.687
   C00575  cAMP C10H12N5O6P 352.0439 .H/Na.H(+) 0.748 3.541 2.649
   C00575  cAMP C10H12N5O6P 352.0439 .Na(+) 0.748 3.541 2.649
   C00429  5,6-dihydrouracil C4H6N2O2 235.0104 .H2PO4Na.H(+) 0.664 3.802 2.523
   C12623  2,3-dihydroxicinnamic acid C9H8O4 163.0385 -H2O.H(+) 0.604 4.174 2.523
   C02876  Propanoyl phosphate C3H7O5P 157.0156 [+2].H(+) 0.667 3.661 2.442
   C00575  cAMP C10H12N5O6P 330.0629 .H(+) 0.633 3.782 2.395
   C00979  O-Acetyl-L-serine C5H9NO4 387.9785 .(H2PO4Na)2.H(+) 0.608 3.667 2.230
   C11145  methanesulfonate CH4O3S 118.9717 .H/Na.H(+) 0.599 5.926 0.000
   C00029  UDPglucose C15H24N2O17P2 589.0434 .H/Na.H(+) 0.583 4.905 0.000
   C00122  Fumarate C4H4O4 117.0183 .H(+) 0.577 4.434 0.000
   C01672  1,5-Diaminopentane C5H14N2 86.0967 -NH3.H(+) 0.575 -3.526 -0.000
   C05925  Dihydroneopterin monophosphate C9H14N5O7P 472.0007 .H2PO4K.H(+) 0.571 3.654 0.000
   C00134  Putrescine C4H12N2 72.0809 -NH3.H(+) 0.567 -4.181 -0.000
   C00993  D-Alanyl-D-alanine C6H12N2O3 144.0646 -NH3.H(+) 0.565 4.904 0.000
   C00114  Choline C5H13NO 86.0967 -H2O.H(+) 0.552 -3.526 -0.000
   C04114  crotonobetaine C7H13NO2 166.0861 .H/Na.H(+) 0.550 -4.068 -0.000
   C00149  L-Malate C4H6O5 117.0183 -H2O.H(+) 0.541 4.434 0.000
   C00048  Glyoxylate C2H2O3 194.9634 .H2PO4Na.H(+) 0.535 7.887 0.000
   C00148  L-Proline C5H9NO2 72.0809 -CO2.H(+) 0.525 -4.181 -0.000
   C00048  Glyoxylate C2H2O3 216.9495 .HPO4Na2.H(+) 0.517 4.839 0.000
   Tetradecanoyl-phosphate (n-C14:0)  Tetradecanoyl-phosphate (n-C14:0) C14H29O5P 429.1521 .H2PO4Na.H(+) 0.515 -3.580 -0.000
   C05932  N2-Succinyl-L-glutamate 5-semialdehyde C9H13NO6 472.0007 .(H2PO4Na)2.H(+) 0.515 3.654 0.000
   C00320  Thiosulfate H2O3S2 234.9084 .H2PO4Na.H(+) 0.504 4.103 0.000
   C00049  L-Aspartate C4H7NO4 117.0183 -NH3.H(+) 0.496 4.434 0.000
   C05629  Phenylpropanoate C9H10O2 287.0113 .H2PO4K.H(+) 0.494 -3.585 -0.000
   C00979  O-Acetyl-L-serine C5H9NO4 148.0605 .H(+) 0.464 4.445 0.000
   C00887  Nitrous oxide N2O 164.9695 .H2PO4Na.H(+) 0.460 6.873 0.000
   C05932  N2-Succinyl-L-glutamate 5-semialdehyde C9H13NO6 352.0439 .H2PO4Na.H(+) 0.456 3.541 0.000
   C00576  Betaine aldehyde C5H11NO 103.0968 [+1].H(+) 0.447 5.267 0.000
   C03733  UDP-D-galacto-1,4-furanose C15H24N2O17P2 589.0434 .H/Na.H(+) 0.447 4.905 0.000
   C03090  5-Phospho-beta-D-ribosylamine C5H12NO7P 447.9857 .(H2PO4)2NaH.H(+) 0.426 4.170 0.000
   C00217  D-Glutamate C5H9NO4 148.0605 .H(+) 0.000 4.445 0.000
   C00217  D-Glutamate C5H9NO4 387.9785 .(H2PO4Na)2.H(+) 0.000 3.667 0.000
   C02737  phosphatidylserine (dihexadec-9-enoyl, n-C16:1) C38H70N1O10P1 770.4381 .H/K.H(+) 0.000 -3.654 -0.000
   C03340  2,3-Dihydrodipicolinate C7H7NO4 387.9785 .(H2PO4)2NaH.H(+) 0.000 3.667 0.000
   C00015  UDP C9H14N2O12P2 578.8901 .HPO4K2.H(+) 0.639 -3.487 -2.229
   C00079  L-Phenylalanine C9H11NO2 166.0861 .H(+) 0.610 -4.068 -2.483
   C00957  Mercaptopyruvate C3H4O3S 142.9805 .H/Na.H(+) 0.672 -3.751 -2.520
   C03296  N2-Succinyl-L-arginine C10H18N4O5 277.1418 [+2].H(+) 0.761 -3.706 -2.819
   C03657  1,4-Dihydroxy-2-naphthoate C11H8O4 243.0012 .H/K.H(+) 0.785 -3.609 -2.834
   C00079  L-Phenylalanine C9H11NO2 167.0902 [+1].H(+) 0.781 -4.235 -3.309
   C00887  Nitrous oxide N2O 316.8757 .(H2PO4K)2.H(+) 0.819 -4.462 -3.655
     KEGG pathway by CLR  
   Pathway_ion pvalue_ion qvalue_ion  Arginine and proline metabolism 1e-08 0.0000
  Butanoate metabolism 1e-07 0.0000
  Tyrosine metabolism 2e-06 0.0001
  Benzoate degradation 5e-06 0.0001
  Alanine, aspartate and glutamate metabolism 4e-05 0.0005
  Microbial metabolism in diverse environments 4e-05 0.0005
  Nitrotoluene degradation 6e-05 0.0006
  Phenylalanine metabolism 8e-05 0.0007
  D-Glutamine and D-glutamate metabolism 0.0003 0.0019
  Two-component system 0.0003 0.0018
  Nitrogen metabolism 0.0005 0.0027
  Cysteine and methionine metabolism 0.0006 0.0031
  Taurine and hypotaurine metabolism 0.001 0.0054
  C5-Branched dibasic acid metabolism 0.001 0.0068
  Aminoacyl-tRNA biosynthesis 0.001 0.0064
  Citrate cycle (TCA cycle) 0.002 0.0084
  Lysine biosynthesis 0.002 0.0096
  Nicotinate and nicotinamide metabolism 0.003 0.0123
  Bacterial chemotaxis 0.006 0.0201
  Glutathione metabolism 0.006 0.0213
  Methane metabolism 0.006 0.0204
  Phenylalanine, tyrosine and tryptophan biosynthesis 0.007 0.0232
  Oxidative phosphorylation 0.008 0.0248
     COG enrichment  
   Pathway_MS pvalue_MS qvalue_MS  Ribosome 0.0005 0.0464
  Aminoacyl-tRNA biosynthesis 0.001 0.0610
  Bisphenol degradation 0.007 0.2197
  Glycerolipid metabolism 0.009 0.2217
     Predicted metabolites from CLR  
   Predicted metabolites Pvalue Overlap with hits  S-Formylglutathione 0 0.0000
  L-Lysine-tRNA (Lys) 0 0.0000
  tRNA(Lys) 0 0.0000
  Glycerol 2-phosphate 2e-05 0.0000
  sn-Glycero-3-phosphocholine 7e-05 0.0000
  sn-Glycero-3-phospho-1-inositol 7e-05 0.0000
  Glycerophosphoserine 7e-05 0.0000
  butanesulfonate 0.0005 0.0000
  Isethionic acid 0.0005 0.0000
  Dihydroxyacetone 0.0009 0.0000
  N-Acetyl-D-glucosamine(anhydrous)N-Acetylmuramic acid 0.0009 0.0000
  N-Acetyl-D-glucosamine(anhydrous)N-Acetylmuramyl-tripeptide 0.0009 0.0000
  N-Acetyl-D-glucosamine(anhydrous)N-Acetylmuramyl-tetrapeptide 0.0009 0.0000
  ethanesulfonate 0.0009 0.0000
  L-Methionine Sulfoxide 0.0009 0.0000
  methanesulfonate 0.0009 1.0000
  sulfoacetate 0.0009 1.0000
  Deoxycytidine 0.002 0.0000
  Glycerol 0.002 0.0000
  Deoxyadenosine 0.004 0.0000
  Deoxyguanosine 0.004 0.0000
  dehydroglycine 0.004 0.0000
  Deoxyinosine 0.004 0.0000
  Fe3+ 0.004 0.0000
  Guanosine 0.004 0.0000
  Citrate 0.005 0.0000
  4-Methyl-5-(2-phosphoethyl)-thiazole 0.007 0.0000
  Adenosine 0.007 0.0000
  Thymidine 0.007 0.0000
  Deoxyuridine 0.01 1.0000
  1-deoxy-D-xylulose 5-phosphate 0.01 0.0000
  Inosine 0.01 0.0000
  Inorganic triphosphate 0.01 0.0000
    
 
