# Supplementary material for: Genomewide landscape of gene–metabolome associations in Escherichia coli
Source: Mol Syst Biol. 2017 Jan 16;13(1):907. doi: 10.15252/msb.20167150 (PMC5293155; doi:10.15252/msb.20167150)
Supplement: Supplementary file 4 — Table EV3 [file MSB-13-907-s004.zip › details/data_yahK.html]

 
 
 yahK 
  yahK - details 
 
 
  CLR  
   Gene_matching CLR_index  yjeK 9.8
  yahC 9.4
  ilvA 9.4
  metE 9.1
  cysH 8.7
  ilvB 8.5
  holD 8.4
  panC 8.3
  gadX 8.2
  glmM 8.1
  pdxJ 7.9
  ypdG 7.8
  ygcL 7.7
  ptsP 7.5
  yhhW 7.4
  metB 7.3
  ygeR 7.3
  yajO 7.3
  yjdF 7.3
  cmtA 7.1
  ymcA 6.9
  cof 6.9
  nanA 6.6
  hemX 6.5
  betT 6.5
  ompR 6.5
  ilvE 6.4
  gspO 6.4
  srlB 6.3
  crcB 6.3
  lysC 6.3
  etp 6.2
  thiQ 6.2
  ygcN 6.1
  metH 6.0
  flgK 6.0
  crp 6.0
  rnb 5.9
  rsgA 5.9
  yfjR 5.9
  nhaA 5.9
  pdxA 5.9
  exuT 5.9
  ilvM 5.8
  ybgA 5.7
  rpsO 5.7
  yieI 5.6
  ascF 5.6
  metL 5.5
  yfcV 5.4
  lpp 5.4
  yneG 5.4
  asnA 5.4
  bioH 5.3
  yaaW 5.3
  fhuC 5.3
  ysgA 5.3
  proX 5.2
  entB 5.2
  ygeX 5.2
  argH 5.2
  rffC 5.2
  ubiG 5.2
  dnaQ 5.2
  dedA 5.2
  ygfO 5.1
  fldB 5.1
  aroH 5.1
  treC 5.1
  yjfL 5.1
  cchA 5.0
  mcrB 5.0
  speG 5.0
  nlpE 5.0
  murP 5.0
  ydgH 5.0
  emrK 4.9
  rpsU 4.9
  spr 4.9
  agaB 4.9
  ccmH 4.9
  chbC 4.8
  yhbJ 4.8
  fucP 4.8
  yibL 4.8
  yehA 4.8
  sfmC 4.8
  fruK 4.8
  yegX 4.8
  mgtA 4.7
  edd 4.7
  purC 4.7
  rbsD 4.7
  metQ 4.7
  rep 4.7
  sbmC 4.7
  yihN 4.7
  yhhM 4.7
  yjeO 4.7
  potA 4.7
  pepQ 4.6
  hsdS 4.6
  yfaQ 4.6
  yhbE 4.6
  yjfJ 4.6
  ypjL 4.6
  cysG 4.5
  uidA 4.5
  coaE 4.5
  kefA 4.5
  mrcA 4.5
  alaS 4.5
  rfaL 4.4
  rpoN 4.4
  panB 4.4
  yzgL 4.4
  uraA 4.4
  yfaW 4.4
  ykfB 4.4
  oxyR 4.4
  purK 4.4
  yhjC 4.4
  eda 4.4
  cbpA 4.4
  cyaY 4.4
  hokC 4.3
  yjfK 4.3
  prfC 4.3
  yeeP 4.3
  yfcD 4.3
  prpD 4.3
  cchB 4.2
  tnaA 4.2
  hdeA 4.2
  cysZ 4.2
  yhcA 4.2
  rbsK 4.2
  ybgC 4.2
  yddL 4.2
  kbaY 4.2
  kbl 4.2
  ydaS 4.2
  ydfZ 4.2
  yfcZ 4.2
  yhbS 4.2
  bcp 4.2
  cysN 4.2
  malI 4.1
  ygjR 4.1
  ygaU 4.1
  glpC 4.1
  yedE 4.1
  yadN 4.1
  ydfO 4.1
  yfhL 4.1
  yhdN 4.1
  ynfM 4.1
  yjeH 4.0
  ycgJ 4.0
  nlpI 4.0
  abrB 4.0
  yfcR 4.0
  yjhG 4.0
  glcB 4.0
  ynbE 4.0
  yaaU 4.0
  yfeS 4.0
  metC 4.0
  pfkA 3.9
  hcaT 3.9
  yfhM 3.9
  pgm 3.9
  ydcF 3.9
  lsrC 3.9
  kefC 3.9
  yohM 3.9
  ycbG 3.9
  proW 3.9
  yhjR 3.9
  yadB 3.9
  agaS 3.9
  yagU 3.9
  sgcB 3.9
  yicC 3.9
  xdhB 3.9
  flk 3.9
  yifK 3.9
  cysJ 3.9
  ybfN 3.8
  purH 3.8
  dadX 3.8
  kbaZ 3.8
  sdaC 3.8
  rbfA 3.8
  fliS 3.8
  ybbN 3.8
  yqeG 3.8
  yphD 3.8
  rpsT 3.8
  flgE 3.8
  crl 3.8
  dsrB 3.8
  yhdV 3.8
  bioD 3.7
  ybcN 3.7
  exbB 3.7
  ydiZ 3.7
  ybaX 3.7
  leuL 3.7
  prfB 3.7
  yebV 3.7
  mdtK 3.7
  ybjD 3.7
  speA 3.7
  cpxP 3.6
  ydeS 3.6
  wcaB 3.6
  rrmJ 3.6
  yjeP 3.6
  tpx 3.6
  dmsB 3.6
  yrbC 3.6
  hybO 3.6
  sodA 3.6
  pitB 3.6
  yebT 3.6
  yedK 3.6
  dinJ 3.6
  ycdT 3.6
  ushA 3.6
  rfaJ 3.6
  yibG 3.6
  yhcO 3.6
  nudB 3.6
  nrfG 3.6
  fadD 3.6
  acpT 3.6
  bcsC 3.6
  purE 3.6
  galF 3.6
  dcuC 3.6
  gshA 3.6
  glnD 3.5
  ychF 3.5
  ygjM 3.5
  frvA 3.5
  rng 3.5
  trg 3.5
  malF 3.5
  yciH 3.5
  ydaF 3.5
  ppx 3.5
  ulaE 3.5
  yheN 3.4
  yjiH 3.4
  yagZ 3.4
  dkgA 3.4
  djlC 3.4
  hyfB 3.4
  ytfQ 3.4
  ygcW 3.4
  yiaW 3.4
  yigB 3.4
  yrdB 3.4
  ilvY 3.4
  yafU 3.4
  paaY 3.4
  yjiO 3.4
  yjbJ 3.4
  yojI 3.3
  ynjA 3.3
  hyaF 3.3
  cysC 3.3
  yedP 3.3
  pnp 3.3
  ulaC 3.3
  yjgH 3.3
  yhaM 3.3
  speB 3.3
  ruvB 3.3
  torY 3.3
  ycdU 3.3
  yfjI 3.3
  avtA 3.3
  tiaE 3.3
  yjhR 3.3
  alsE 3.3
  gcl 3.3
  fes 3.3
  pepE 3.2
  yfiE 3.2
  ybdR 3.2
  narZ 3.2
  yibK 3.2
  ypfN 3.2
  zitB 3.2
  yaaA 3.2
  yhhS 3.2
  sixA 3.2
  yigF 3.2
  hofB 3.2
  yfiB 3.2
  secG 3.2
  hflC 3.2
  yheL 3.2
  ascG 3.2
  yaiI 3.2
  yjhF 3.2
  fimI 3.2
  mdlA 3.2
  yfbT 3.2
  yjhP 3.2
  yjfC 3.2
  rfbD 3.2
  yajG 3.2
  yphE 3.2
  yraP 3.2
  yneK 3.2
  mrcB 3.2
  yfcY 3.1
  yacH 3.1
  rfaQ 3.1
  cysB 3.1
  puuD 3.1
  ggt 3.1
  yagS 3.1
  rihA 3.1
  ylcE 3.1
  yidR 3.1
  trxA 3.1
  rfaH 3.1
  yfiR 3.1
  yieH 3.1
  wcaC 3.1
  cysU 3.1
  fimC 3.1
  baeR 3.1
  rumB 3.1
  rof 3.1
  rpmF 3.0
  amiA 3.0
  yicS 3.0
  yfiM 3.0
  rfaF 3.0
  ydcH 3.0
  yneJ 3.0
  spy 3.0
  dicB 3.0
  cdaR 3.0
  lysA 3.0
     Differential ions  
   id name formula mz mod AUC Z-score Z-score AUC Weighted   C03406  N(omega)-(L-Arginino)succinate C10H18N4O6 291.1196 [+2]-H(+) 0.790 5.628 4.448
   C00802  Oxalureate C3H4N2O4 168.9633 .H/K-H(+) 0.934 4.476 4.180
   C04494  Guanosine 3'-diphosphate 5'-triphosphate C10H18N5O20P5 703.8900 .H/Na-H(+) 0.790 3.667 2.899
   C00647  Pyridoxamine 5'-phosphate C8H13N2O5P 382.9811 .H2PO4K-H(+) 0.720 3.556 2.561
   C03415  N2-Succinyl-L-ornithine C9H16N2O5 249.1108 +OH(-) 0.706 3.500 2.470
   C00681  1-tetradecanoyl-sn-glycerol 3-phosphate C17H35O7P1 382.2150 [+1]-H(+) 0.599 -4.199 -0.000
   C00188  L-Threonine C4H9NO3 140.0345 .H/Na-H(+) 0.560 -5.746 -0.000
   C00680  meso-2,6-Diaminoheptanedioate C7H14N2O4 208.1035 [+1]+OH(-) 0.554 -3.467 -0.000
   C15767  gamma-glutamyl-gamma aminobutyric acid C9H16O5N2 249.1108 +OH(-) 0.544 3.500 0.000
   C03974  2-tetradecanoyl-sn-glycerol 3-phosphate C17H35O7P1 382.2150 [+1]-H(+) 0.537 -4.199 -0.000
   Glycerophosphoserine  Glycerophosphoserine C6H14NO8P 393.9680 .H2PO4K-H(+) 0.522 -4.578 -0.000
   C00263  L-Homoserine C4H9NO3 140.0345 .H/Na-H(+) 0.496 -5.746 -0.000
   C00253  Nicotinate C6H5NO2 140.0345 +OH(-) 0.467 -5.746 -0.000
   C00666  LL-2,6-Diaminoheptanedioate C7H14N2O4 208.1035 [+1]+OH(-) 0.000 -3.467 -0.000
   C00931  Porphobilinogen C10H14N2O4 225.0846 -H(+) 0.000 -3.720 -0.000
   C01131  L-Rhamnulose 1-phosphate C6H13O8P 243.0200 -H(+) 0.000 -4.414 -0.000
   C01268  5-Amino-6-(5'-phosphoribosylamino)uracil C9H15N4O9P 526.9519 .HPO4K2-H(+) 0.000 -4.564 -0.000
   C11436  2-phospho-4-(cytidine 5'-diphospho)-2-C-methyl-D-erythritol C14H26N3O17P3 735.9582 .H2PO4K-H(+) 0.000 -5.333 -0.000
   C04462  N-Succinyl-2-L-amino-6-oxoheptanedioate C11H15NO8 208.1035 -HPO3-H(+) 0.633 -3.467 -2.195
   C00279  D-Erythrose 4-phosphate C4H9O7P 201.0028 [+2]-H(+) 0.676 -3.658 -2.474
   C01099  L-Fuculose 1-phosphate C6H13O8P 243.0200 -H(+) 0.607 -4.414 -2.681
   C00352  D-Glucosamine 6-phosphate C6H14NO8P 393.9680 .H2PO4K-H(+) 0.643 -4.578 -2.946
   C00140  N-Acetyl-D-glucosamine C8H15NO6 393.9680 .HPO4K2-H(+) 0.712 -4.578 -3.258
   C00645  N-Acetyl-D-mannosamine C8H15NO6 393.9680 .HPO4K2-H(+) 0.743 -4.578 -3.402
   C02730  o-Succinylbenzoate C11H10O5 243.0200 .H/Na-H(+) 0.803 -4.414 -3.544
   C16154  uridine 5''-diphospho-{beta}-4-deoxy-4-formamido-L-arabinose C15H23N3O16P2 735.9582 .HPO4K2-H(+) 0.840 -5.333 -4.481
   C06156  D-Glucosamine 1-phosphate C6H14NO8P 393.9680 .H2PO4K-H(+) 0.979 -4.578 -4.484
   C05519  L-Allo-threonine C4H9NO3 140.0345 .H/Na-H(+) 0.833 -5.746 -4.786
     KEGG pathway by CLR  
   Pathway_ion pvalue_ion qvalue_ion  Fructose and mannose metabolism 5e-06 0.0005
  Benzoate degradation 5e-06 0.0002
  Aminobenzoate degradation 0.001 0.0380
  Bisphenol degradation 0.002 0.0409
  Microbial metabolism in diverse environments 0.003 0.0513
  Glycolysis / Gluconeogenesis 0.003 0.0487
  Nicotinate and nicotinamide metabolism 0.005 0.0704
  Porphyrin and chlorophyll metabolism 0.007 0.0817
  Amino sugar and nucleotide sugar metabolism 0.008 0.0875
  Phosphotransferase system (PTS) 0.01 0.0933
     COG enrichment  
   Pathway_MS pvalue_MS qvalue_MS  Chlorocyclohexane and chlorobenzene degradation 0 0.0000
  Fluorobenzoate degradation 0 0.0000
  Pantothenate and CoA biosynthesis 6e-05 0.0013
  Sulfur metabolism 6e-05 0.0010
  Purine metabolism 0.001 0.0166
  Selenoamino acid metabolism 0.002 0.0244
  Biosynthesis of secondary metabolites 0.004 0.0351
  Valine, leucine and isoleucine biosynthesis 0.004 0.0321
  Phosphotransferase system (PTS) 0.005 0.0324
  Galactose metabolism 0.006 0.0359
  Lipopolysaccharide biosynthesis 0.006 0.0370
  Ribosome 0.006 0.0342
  Pentose phosphate pathway 0.007 0.0369
  Arachidonic acid metabolism 0.008 0.0390
  Cysteine and methionine metabolism 0.009 0.0403
  Lysine biosynthesis 0.01 0.0387
     Predicted metabolites from CLR  
   Predicted metabolites Pvalue Overlap with hits  5-amino-1-(5-phospho-D-ribosyl)imidazole-4-carboxylate 0 0.0000
  5-phosphoribosyl-5-carboxyaminoimidazole 0 0.0000
  3-Methyl-2-oxobutanoate 0.0003 0.0000
  D-Tagatose 1,6-biphosphate 0.0003 0.0000
  2-Dehydro-3-deoxy-D-gluconate 6-phosphate 0.0007 0.0000
  4-Phospho-L-aspartate 0.0007 0.0000
  5-Methyltetrahydrofolate 0.0007 0.0000
  Adenosine 5'-phosphosulfate 0.0007 0.0000
  L-Cystathionine 0.0007 0.0000
  dihydrosirohydrochlorin 0.0007 0.0000
  Uroporphyrinogen III 0.0007 0.0000
  Hydrogen sulfide 0.002 0.0000
  L-Homocysteine 0.002 0.0000
  5,6,7,8-Tetrahydrofolate 0.003 0.0000
  2,5-diketo-D-gluconate 0.003 0.0000
  ADP-L-glycero-D-manno-heptose 0.003 0.0000
  Pyridoxine 5'-phosphate 0.003 0.0000
  O-Phospho-4-hydroxy-L-threonine 0.003 0.0000
  3'-Phosphoadenylyl sulfate 0.006 0.0000
  L-Cysteine 0.008 0.0000
  Adenosine 3',5'-bisphosphate 0.009 0.0000
    
 
