# Supplementary material for: Genomewide landscape of gene–metabolome associations in Escherichia coli
Source: Mol Syst Biol. 2017 Jan 16;13(1):907. doi: 10.15252/msb.20167150 (PMC5293155; doi:10.15252/msb.20167150)
Supplement: Supplementary file 4 — Table EV3 [file MSB-13-907-s004.zip › details/data_yahL.html]

 
 
 yahL 
  yahL - details 
 
 
  CLR  
   Gene_matching CLR_index  rnt 17.6
  uhpA 17.3
  tap 15.0
  yfjK 15.0
  clpX 12.3
  yeiE 12.0
  rpe 11.7
  sohB 11.5
  rpiB 11.4
  yidZ 11.3
  phoP 11.2
  ynfL 10.6
  glgC 10.4
  ytjC 10.2
  tesA 9.6
  rcsB 9.5
  fiu 9.4
  gltA 9.2
  yfhG 8.9
  gpmI 8.8
  helD 8.8
  ymcB 8.7
  nuoN 8.7
  nuoA 8.5
  ydfX 8.5
  yehQ 8.4
  treR 8.1
  purR 8.0
  zraS 8.0
  rhaR 7.9
  rssB 7.7
  narL 7.7
  glpX 7.6
  mpaA 7.3
  rhlB 7.3
  lysR 7.2
  ydgT 7.1
  lsrF 7.0
  ydeU 7.0
  appC 7.0
  ymfL 6.9
  nlpB 6.8
  yehS 6.7
  idnR 6.6
  pepN 6.6
  cyoC 6.5
  hupB 6.5
  yeeE 6.3
  ybjR 6.3
  yfdV 6.1
  cyoD 6.0
  oppA 6.0
  cusB 6.0
  ydcP 6.0
  dbpA 5.9
  yceH 5.8
  ydeI 5.7
  cadC 5.7
  ybgE 5.7
  prpR 5.7
  nuoB 5.7
  ygdD 5.6
  yebR 5.5
  cpxR 5.5
  poxB 5.4
  pflB 5.4
  metJ 5.4
  ompF 5.4
  hepA 5.4
  wza 5.4
  ygcP 5.3
  yajC 5.2
  yecE 5.2
  yfdI 5.2
  ydhW 5.1
  yehW 5.1
  srlR 5.1
  mltA 5.0
  ypjF 5.0
  yfbV 4.9
  ydgC 4.9
  ompN 4.9
  tktB 4.9
  nuoM 4.8
  degP 4.8
  dacC 4.8
  zraR 4.7
  fucR 4.7
  uxuR 4.6
  rcsC 4.6
  btuR 4.6
  yqaD 4.6
  ygeD 4.6
  ygbJ 4.6
  eutB 4.6
  yedV 4.6
  uxuA 4.6
  csdA 4.5
  yfcT 4.5
  ygaM 4.5
  dacA 4.5
  ycfR 4.4
  evgA 4.4
  ybjC 4.4
  yfhA 4.3
  uhpB 4.3
  cheY 4.3
  ydhR 4.3
  mhpR 4.2
  cusC 4.2
  mipA 4.2
  ycdN 4.1
  cusF 4.1
  yfeX 4.1
  yacL 4.1
  cirA 4.0
  yoaG 4.0
  yegL 4.0
  mdh 4.0
  slp 3.9
  yeaL 3.9
  ybjM 3.9
  ybaP 3.9
  oppD 3.8
  purD 3.8
  yecF 3.8
  ynaE 3.8
  yfjQ 3.7
  pgpB 3.7
  yfjH 3.7
  pgm 3.7
  cybC 3.7
  ygbE 3.7
  yegX 3.7
  yfeU 3.7
  bglG 3.7
  ldcA 3.7
  dcuS 3.6
  norV 3.6
  yfcJ 3.6
  talB 3.6
  clpA 3.5
  yghF 3.5
  eutT 3.5
  nuoK 3.5
  ykgD 3.5
  rffH 3.4
  ydiT 3.4
  talA 3.4
  cpxA 3.4
  yejK 3.4
  mhpF 3.4
  fadL 3.4
  pstB 3.4
  galR 3.4
  ygdR 3.4
  yjiE 3.3
  gnsB 3.3
  ynjF 3.3
  glgS 3.3
  mutM 3.3
  yciC 3.2
  metR 3.2
  yghX 3.2
  phoB 3.1
  yagA 3.1
  frdA 3.1
  ybfB 3.1
  yqeI 3.1
  paaG 3.1
  narX 3.1
  ybbY 3.0
  uup 3.0
  ymfM 3.0
  yphF 3.0
  ynbA 3.0
  ymfO 3.0
  frsA 3.0
     Differential ions  
   id name formula mz mod AUC Z-score Z-score AUC Weighted   C00350  phosphatidylethanolamine (ditetradecanoyl, n-C14:0) C33H66N1O8P1 870.3786 .(H2PO4)2KH.H(+) 0.954 4.630 4.417
   C00062  L-Arginine C6H14N4O2 175.1189 .H(+) 0.649 5.792 3.758
   C07086  Phenylacetic acid C8H8O2 354.9967 .(H2PO4)2NaH.H(+) 0.760 4.907 3.729
   C00568  4-Aminobenzoate C7H7NO2 138.0532 .H(+) 0.943 3.861 3.639
   C01165  L-Glutamate 5-semialdehyde C5H9NO3 154.0508 .H/Na.H(+) 0.854 4.023 3.435
   C00793  D-Cysteine C3H7NO2S 144.0080 .H/Na.H(+) 0.891 3.477 3.099
   C00577  D-Glyceraldehyde C3H6O3 91.0382 .H(+) 0.731 3.698 2.703
   C00163  Propionate (n-C3:0) C3H6O2 97.0277 .H/Na.H(+) 0.606 4.264 2.585
   C00212  Adenosine C10H13N5O4 286.1039 [+2]+OH(-) 0.652 3.957 2.580
   C00330  Deoxyguanosine C10H13N5O4 286.1039 [+2]+OH(-) 0.642 3.957 2.538
   C00082  L-Tyrosine C9H11NO3 138.0900 -CO2.H(+) 0.619 4.086 2.531
   C08362  Hexadecenoate (n-C16:1) C16H30O2 397.1716 .HPO4Na2.H(+) 0.649 3.854 2.500
   branching glycogen  branching glycogen C6H10O5 145.0492 -H2O.H(+) 0.678 3.516 2.383
   C00109  2-Oxobutanoate C4H6O3 85.0284 -H2O.H(+) 0.661 3.584 2.369
   C00497  D-Malate C4H6O5 91.0382 -CO2.H(+) 0.629 3.698 2.326
   C00149  L-Malate C4H6O5 91.0382 -CO2.H(+) 0.606 3.698 2.239
   C00164  Acetoacetate C4H6O3 85.0284 -H2O.H(+) 0.608 3.584 2.179
   C00062  L-Arginine C6H14N4O2 317.0584 .HPO4Na2.H(+) 0.596 5.075 0.000
   C00182  glycogen C6H10O5 145.0492 -H2O.H(+) 0.596 3.516 0.000
   C00148  L-Proline C5H9NO2 138.0532 .Na(+) 0.595 3.861 0.000
   C00148  L-Proline C5H9NO2 138.0532 .H/Na.H(+) 0.595 3.861 0.000
   C00256  D-Lactate C3H6O3 91.0382 .H(+) 0.595 3.698 0.000
   C05235  Acetol C3H6O2 97.0277 .H/Na.H(+) 0.594 4.264 0.000
   C00134  Putrescine C4H12N2 111.0891 .Na(+) 0.591 -3.638 -0.000
   C00134  Putrescine C4H12N2 111.0891 .H/Na.H(+) 0.591 -3.638 -0.000
   C00135  L-Histidine C6H9N3O2 156.0772 .H(+) 0.590 3.545 0.000
   C00232  Succinic semialdehyde C4H6O3 85.0284 -H2O.H(+) 0.589 3.584 0.000
   C04294  4-Methyl-5-(2-hydroxyethyl)-thiazole C6H9NOS 145.0492 [+1].H(+) 0.587 3.516 0.000
   C00062  L-Arginine C6H14N4O2 197.1003 .Na(+) 0.584 4.088 0.000
   C00062  L-Arginine C6H14N4O2 197.1003 .H/Na.H(+) 0.584 4.088 0.000
   C00184  Dihydroxyacetone C3H6O3 91.0382 .H(+) 0.575 3.698 0.000
   C00204  2-Dehydro-3-deoxy-D-gluconate C6H10O6 314.9952 .H2PO4K.H(+) 0.574 -4.447 -0.000
   C00627  Pyridoxine 5'-phosphate C8H12NO6P 206.0603 -CO2.H(+) 0.572 3.931 0.000
   C00740  D-Serine C3H7NO3 144.0080 .H/K.H(+) 0.571 3.477 0.000
   C00937  D-Lactaldehyde C3H6O2 97.0277 .H/Na.H(+) 0.565 4.264 0.000
   C00097  L-Cysteine C3H7NO2S 144.0080 .H/Na.H(+) 0.562 3.477 0.000
   C00108  Anthranilate C7H7NO2 138.0532 .H(+) 0.560 3.861 0.000
   C00424  L-Lactaldehyde C3H6O2 97.0277 .H/Na.H(+) 0.557 4.264 0.000
   C01250  N-Acetyl-L-glutamate 5-semialdehyde C7H11NO4 175.0833 [+1].H(+) 0.555 4.177 0.000
   C00062  L-Arginine C6H14N4O2 176.1217 [+1].H(+) 0.520 5.351 0.000
   C00062  L-Arginine C6H14N4O2 177.1227 [+2].H(+) 0.509 4.724 0.000
   C00114  Choline C5H13NO 104.1064 .H(+) 0.487 -3.824 -0.000
   C00065  L-Serine C3H7NO3 144.0080 .H/K.H(+) 0.479 3.477 0.000
   C00186  L-Lactate C3H6O3 91.0382 .H(+) 0.444 3.698 0.000
   C00596  2-Oxopent-4-enoate C5H6O3 97.0277 -H2O.H(+) 0.426 4.264 0.000
   C01672  1,5-Diaminopentane C5H14N2 104.1261 [+1].H(+) 0.426 -3.468 -0.000
   C01013  3-Hydroxypropanoate C3H6O3 91.0382 .H(+) 0.380 3.698 0.000
   C00430  5-Amino-4-oxopentanoate C5H9NO3 154.0508 .H/Na.H(+) 0.000 4.023 0.000
   C03741  L-Glutamate 1-semialdehyde C5H9NO3 154.0508 .H/Na.H(+) 0.000 4.023 0.000
   C06424  tetradecanoate (n-C14:0) C14H28O2 371.1617 .HPO4Na2.H(+) 0.705 -3.512 -2.475
   C01216  2-Dehydro-3-deoxy-D-galactonate C6H10O6 314.9952 .H2PO4K.H(+) 0.705 -4.447 -3.135
     KEGG pathway by CLR  
   Pathway_ion pvalue_ion qvalue_ion  Arachidonic acid metabolism 0 0.0000
  Pyruvate metabolism 3e-07 0.0000
  Propanoate metabolism 2e-06 0.0000
  Nicotinate and nicotinamide metabolism 6e-06 0.0001
  Aminobenzoate degradation 4e-05 0.0004
  Chlorocyclohexane and chlorobenzene degradation 8e-05 0.0008
  Arginine and proline metabolism 0.0001 0.0013
  Fructose and mannose metabolism 0.0002 0.0012
  Glycerolipid metabolism 0.0002 0.0017
  Butanoate metabolism 0.0006 0.0040
  Microbial metabolism in diverse environments 0.0009 0.0054
  Aminoacyl-tRNA biosynthesis 0.001 0.0071
  Biosynthesis of secondary metabolites 0.002 0.0096
  beta-Alanine metabolism 0.008 0.0375
  Glyoxylate and dicarboxylate metabolism 0.008 0.0351
  Novobiocin biosynthesis 0.009 0.0367
  Thiamine metabolism 0.009 0.0347
     COG enrichment  
   Pathway_MS pvalue_MS qvalue_MS  Two-component system 2e-09 0.0000
  Oxidative phosphorylation 7e-07 0.0000
  Pentose phosphate pathway 1e-05 0.0004
  Streptomycin biosynthesis 0.005 0.0959
     Predicted metabolites from CLR  
   Predicted metabolites Pvalue Overlap with hits  silver 2e-06 0.0000
  Cu+ 8e-06 0.0000
  Sedoheptulose 7-phosphate 2e-05 0.0000
  D-Glycerate 2-phosphate 5e-05 0.0000
  D-Erythrose 4-phosphate 0.0001 0.0000
  3-Phospho-D-glycerate 0.0002 0.0000
  L-alanine-D-glutamate-meso-2,6-diaminoheptanedioate-D-alanine 0.0002 0.0000
  L-alanine-D-glutamate-meso-2,6-diaminoheptanedioate 0.0003 0.0000
  2-Demethylmenaquinone 8 0.0009 0.0000
  D-Glucose 1-phosphate 0.0009 0.0000
  2-Demethylmenaquinol 8 0.001 0.0000
  D-Ribulose 5-phosphate 0.002 0.0000
  D-Fructose 6-phosphate 0.002 0.0000
  D-Xylulose 5-phosphate 0.003 0.0000
  alpha-D-Ribose 5-phosphate 0.005 0.0000
  Glyceraldehyde 3-phosphate 0.008 0.0000
    
 
