# Supplementary material for: Genomewide landscape of gene–metabolome associations in Escherichia coli
Source: Mol Syst Biol. 2017 Jan 16;13(1):907. doi: 10.15252/msb.20167150 (PMC5293155; doi:10.15252/msb.20167150)
Supplement: Supplementary file 4 — Table EV3 [file MSB-13-907-s004.zip › details/data_yaiF.html]

 
 
 yaiF 
  yaiF - details 
 
 
  CLR  
   Gene_matching CLR_index  ysgA 6.7
  ydcH 6.6
  bioH 6.4
  ybfN 6.2
  ycgJ 6.2
  yeeJ 6.0
  yeeP 5.9
  ynfD 5.8
  ivbL 5.8
  ypfJ 5.7
  ygcL 5.6
  mcrB 5.6
  dkgB 5.6
  yneF 5.4
  asr 5.3
  ybjS 5.2
  ybjG 5.2
  dmsD 5.2
  rpsO 5.2
  dhaR 5.1
  rpsT 5.1
  yigL 5.1
  leuB 5.1
  ycdH 5.1
  ubiG 5.0
  yrhA 5.0
  ydhL 5.0
  pnp 5.0
  yhjX 5.0
  yjiK 5.0
  yegR 5.0
  yfeH 4.9
  yhjC 4.9
  yddM 4.9
  ycdU 4.9
  csiE 4.9
  yibK 4.9
  ykgB 4.9
  cld 4.9
  yjhT 4.8
  recQ 4.8
  yebA 4.8
  yihO 4.8
  ypjM 4.8
  tnaB 4.8
  yebN 4.8
  prfB 4.7
  ygcW 4.7
  yncH 4.7
  ybiM 4.7
  glnD 4.7
  rzoD 4.7
  ycdT 4.6
  glcF 4.6
  bglJ 4.5
  aidB 4.5
  prmB 4.5
  potG 4.5
  etp 4.5
  emtA 4.5
  ynfC 4.4
  yghO 4.4
  ybfE 4.4
  hokC 4.4
  ascG 4.4
  ykfC 4.3
  ygcI 4.3
  yohG 4.3
  yphC 4.3
  proY 4.3
  gntU 4.3
  elaD 4.3
  abrB 4.3
  trxA 4.3
  yfeS 4.2
  fhiA 4.2
  yjdC 4.2
  nuoC 4.2
  ymgH 4.2
  yedW 4.2
  abgT 4.2
  yliB 4.2
  arpB 4.1
  ygcS 4.1
  ycbW 4.1
  marC 4.1
  yjjM 4.0
  oxyR 4.0
  yciB 4.0
  ygdI 4.0
  rbsD 4.0
  bolA 4.0
  gadX 4.0
  hycC 4.0
  ygaU 3.9
  yoeA 3.9
  yqhC 3.9
  yfdN 3.9
  gpp 3.9
  hipB 3.9
  ycbG 3.9
  baeR 3.8
  mobB 3.8
  znuA 3.8
  ulaG 3.8
  ykgE 3.8
  yjfP 3.8
  yjiA 3.8
  dgoR 3.8
  torS 3.8
  aroK 3.8
  tdcR 3.8
  tfaD 3.8
  chbC 3.7
  yidR 3.7
  malF 3.7
  pcnB 3.7
  rpsU 3.7
  ypdG 3.7
  hdfR 3.7
  ygbN 3.7
  ycgH 3.7
  yjiT 3.7
  ymdE 3.7
  yobA 3.6
  yjhG 3.6
  yjbJ 3.6
  araC 3.6
  gspO 3.6
  hokE 3.6
  fhlA 3.6
  ydbJ 3.6
  ymfS 3.6
  dgoT 3.6
  cysB 3.6
  cysH 3.6
  yejO 3.6
  tfaS 3.5
  yghK 3.5
  ymfP 3.5
  yicM 3.5
  djlC 3.5
  yhiM 3.5
  metE 3.5
  mcrC 3.5
  flk 3.5
  wcaI 3.4
  ilvA 3.4
  ydhM 3.4
  yphD 3.4
  yedM 3.4
  wcaC 3.4
  yjeK 3.4
  glxK 3.4
  yiiM 3.3
  ylbH 3.3
  gldA 3.3
  dcuC 3.3
  yjgF 3.2
  yeeY 3.2
  ypeB 3.2
  ypfH 3.2
  yjiP 3.2
  apt 3.2
  mbhA 3.2
  yaaY 3.2
  mntH 3.2
  rihA 3.2
  trmU 3.2
  yecC 3.2
  prfC 3.2
  ybbN 3.1
  yceG 3.1
  yedQ 3.1
  trmA 3.1
  yicJ 3.1
  yeeL 3.1
  rtcA 3.1
  glpC 3.0
  yhcA 3.0
  yjfN 3.0
  norR 3.0
  frlR 3.0
  cmtA 3.0
  fucP 3.0
  lipB 3.0
  ybbP 3.0
  citG 3.0
  ybjR 3.0
     Differential ions  
none  KEGG pathway by CLR  
none  COG enrichment  
   Pathway_MS pvalue_MS qvalue_MS  Chlorocyclohexane and chlorobenzene degradation 0 0.0000
  Fluorobenzoate degradation 0 0.0000
  RNA degradation 0.003 0.1004
  Ribosome 0.004 0.1013
  Lipoic acid metabolism 0.007 0.1438
     Predicted metabolites from CLR  
none 
 
