# Supplementary material for: Genomewide landscape of gene–metabolome associations in Escherichia coli
Source: Mol Syst Biol. 2017 Jan 16;13(1):907. doi: 10.15252/msb.20167150 (PMC5293155; doi:10.15252/msb.20167150)
Supplement: Supplementary file 4 — Table EV3 [file MSB-13-907-s004.zip › details/data_yaiI.html]

 
 
 yaiI 
  yaiI - details 
 
 
  CLR  
   Gene_matching CLR_index  ygjV 16.0
  ydbH 15.7
  citB 14.7
  csgF 14.4
  yfcP 14.2
  djlB 13.9
  ybeA 13.6
  ygiH 12.4
  yfbM 12.3
  tfaR 11.9
  rlpA 11.7
  fdhD 11.2
  sspB 11.0
  narX 10.6
  yqiK 10.4
  zntR 10.4
  sfmA 10.3
  ybdN 10.3
  yjeJ 10.2
  yphA 10.1
  ygcW 9.9
  ynjH 9.9
  cyoB 9.9
  ydfZ 9.6
  ssuE 9.6
  yqjA 9.5
  sbmA 9.2
  ychA 8.9
  yfiE 8.7
  aaeR 8.4
  yiaA 8.4
  yqjI 8.1
  hemX 8.1
  yfaQ 8.1
  thiE 8.0
  yidA 7.8
  gadX 7.7
  gpp 7.7
  mdtL 7.5
  ygdK 7.5
  yhhP 7.5
  yoaA 7.4
  pps 7.4
  fpr 7.4
  ybdO 7.3
  dacC 7.3
  yafC 7.3
  ybeF 7.3
  yidB 7.3
  yiaU 7.2
  gadA 7.1
  ycbG 7.0
  ycgY 7.0
  yagJ 7.0
  basR 6.8
  yigB 6.7
  ycdY 6.7
  nohB 6.7
  pfkA 6.6
  ybjH 6.6
  ybiU 6.6
  yhjX 6.6
  tauB 6.6
  helD 6.5
  frmR 6.4
  yehM 6.4
  hsrA 6.3
  ycdX 6.3
  mobA 6.2
  yieN 6.2
  ydeE 6.2
  ycbU 6.2
  clpP 6.1
  ibpA 6.1
  crp 6.1
  yfcD 6.0
  ydaY 6.0
  yfhM 6.0
  dhaR 6.0
  moaA 6.0
  yfaU 5.9
  creC 5.9
  mlrA 5.9
  ylbF 5.9
  ypdG 5.8
  glnE 5.7
  nrfA 5.7
  soxS 5.7
  ydiV 5.7
  yjdI 5.7
  yfcR 5.6
  ydfP 5.6
  visC 5.6
  yihN 5.6
  folX 5.6
  oxyR 5.5
  yneG 5.5
  yfeH 5.5
  uidR 5.5
  yajL 5.5
  maa 5.5
  yghA 5.4
  iclR 5.4
  sstT 5.4
  tar 5.4
  mltB 5.4
  yeaT 5.4
  tnaA 5.4
  rpiR 5.3
  yagB 5.3
  tdh 5.3
  ylbA 5.2
  tyrP 5.2
  ybeM 5.2
  proY 5.2
  ybeT 5.2
  ybjP 5.1
  lipB 5.1
  glxK 5.1
  dksA 5.1
  ycfN 5.1
  uraA 5.0
  ydiH 5.0
  ybiM 5.0
  nlpE 5.0
  yjaH 5.0
  yicS 5.0
  hcaT 5.0
  srmB 4.9
  yjbA 4.9
  yliD 4.9
  prlC 4.9
  ycgM 4.9
  yieI 4.9
  yddL 4.9
  bcsZ 4.9
  ibpB 4.8
  ivbL 4.8
  yijF 4.8
  inaA 4.8
  yidJ 4.8
  yigA 4.7
  yicI 4.7
  mcrA 4.7
  metF 4.7
  exbB 4.7
  ycaJ 4.6
  ydeS 4.6
  yfaA 4.6
  yedJ 4.6
  araG 4.6
  ygjM 4.6
  yaaU 4.5
  yahB 4.5
  cusR 4.4
  pal 4.4
  rpsO 4.4
  torD 4.4
  cchB 4.3
  fldB 4.3
  torT 4.3
  ybcS 4.3
  yobA 4.3
  ydhR 4.3
  metC 4.3
  dcuR 4.3
  yidI 4.3
  sdaB 4.3
  setC 4.3
  ycgK 4.2
  yigF 4.2
  gshB 4.2
  minC 4.2
  lsrC 4.2
  ykfB 4.1
  yddH 4.1
  nanA 4.1
  cobC 4.1
  potA 4.1
  dinJ 4.1
  yadB 4.0
  nhaA 4.0
  fkpA 4.0
  trpC 4.0
  ybcN 4.0
  yifE 4.0
  nadA 4.0
  ygeR 4.0
  yhjC 4.0
  betT 4.0
  aldB 4.0
  insO 4.0
  yegS 3.9
  ycaR 3.9
  ygbN 3.9
  yqiB 3.9
  aroK 3.9
  ygfX 3.8
  ychH 3.8
  ycjT 3.8
  cspH 3.8
  bcsG 3.8
  fadL 3.8
  proW 3.8
  renD 3.8
  ompR 3.8
  yfjF 3.8
  yafZ 3.8
  gntT 3.7
  ydjI 3.7
  pinQ 3.7
  yfjH 3.7
  ybgP 3.7
  fruK 3.7
  yfdE 3.7
  phnP 3.7
  yafW 3.7
  yedK 3.7
  yehI 3.7
  xdhB 3.7
  yieH 3.6
  rpsT 3.6
  hokC 3.6
  aceK 3.6
  yjaG 3.6
  ascG 3.6
  hscC 3.6
  pldB 3.6
  yffI 3.6
  yfjY 3.6
  yrhA 3.6
  yebY 3.5
  wcaD 3.5
  yjeK 3.5
  yhhW 3.5
  yebK 3.5
  hchA 3.5
  ileS 3.5
  yhaH 3.5
  yjdF 3.5
  yedL 3.5
  flgK 3.5
  ycbW 3.5
  flgM 3.5
  yjhR 3.5
  yieE 3.4
  ynbE 3.4
  yfgF 3.4
  cyaY 3.4
  tnaB 3.4
  yjhF 3.4
  crcB 3.4
  nfrB 3.4
  yafK 3.4
  yjgR 3.4
  creB 3.4
  narY 3.4
  tldD 3.3
  yaaW 3.3
  deoC 3.3
  fhuC 3.3
  ssnA 3.3
  ycjW 3.3
  ynfM 3.3
  macB 3.3
  yccV 3.3
  yfbH 3.3
  yfjI 3.3
  bioH 3.3
  ydiU 3.3
  hdeA 3.3
  yceD 3.2
  cirA 3.2
  yidX 3.2
  yihG 3.2
  trkD 3.2
  ssuA 3.2
  wcaI 3.2
  etp 3.2
  yeaY 3.2
  yahK 3.2
  ygbK 3.2
  ilvA 3.2
  yaaX 3.2
  mak 3.1
  ypaA 3.1
  ymcA 3.1
  ompL 3.1
  yfcI 3.1
  cspE 3.1
  yfcF 3.1
  gspO 3.1
  glvB 3.1
  pflC 3.1
  yddG 3.1
  yjfK 3.1
  coaE 3.1
  ptsP 3.1
  perR 3.1
  yieF 3.1
  thiQ 3.1
  yeeI 3.1
  slyX 3.1
  exuR 3.1
  yfaP 3.0
  ompA 3.0
  rzoD 3.0
  yqfB 3.0
  srlE 3.0
  pitB 3.0
  ygjQ 3.0
  gidB 3.0
  yjfL 3.0
  yfiR 3.0
  dsbA 3.0
  zitB 3.0
  yhaI 3.0
  modE 3.0
  yajO 3.0
  ymgB 3.0
     Differential ions  
   id name formula mz mod AUC Z-score Z-score AUC Weighted   C00575  cAMP C10H12N5O6P 329.0479 [+1]-H(+) 0.802 5.159 4.139
   C00942  3',5'-Cyclic GMP C10H12N5O7P 345.0429 [+1]-H(+) 0.819 4.799 3.929
   C05925  Dihydroneopterin monophosphate C9H14N5O7P 605.9214 .(H2PO4K)2-H(+) 0.878 4.391 3.856
   C00575  cAMP C10H12N5O6P 328.0454 -H(+) 0.773 4.909 3.793
   C00612  N1-Acetylspermidine C9H21N3O 328.1004 .HPO4Na2-H(+) 0.954 3.715 3.544
   C01029  N8-Acetylspermidine C9H21N3O 328.1004 .HPO4Na2-H(+) 0.954 3.715 3.544
   C00942  3',5'-Cyclic GMP C10H12N5O7P 344.0409 -H(+) 0.734 4.663 3.424
   C18239  cyclic pyranopterin monophosphate C10H14N5O8P 344.0409 -H2O-H(+) 0.722 4.663 3.369
   C04778  N1-(5-Phospho-alpha-D-ribosyl)-5,6-dimethylbenzimidazole C14H19N2O7P 477.0539 .H2PO4Na-H(+) 0.735 4.487 3.300
   C00575  cAMP C10H12N5O6P 448.0070 .H2PO4Na-H(+) 0.654 4.978 3.257
   C00575  cAMP C10H12N5O6P 330.0516 [+2]-H(+) 0.718 4.497 3.228
   C05931  N2-Succinyl-L-glutamate C9H13NO7 479.9710 .(H2PO4)2KH-H(+) 0.794 4.034 3.202
   C00942  3',5'-Cyclic GMP C10H12N5O7P 381.9982 .H/K-H(+) 0.861 3.704 3.190
   C00942  3',5'-Cyclic GMP C10H12N5O7P 479.9710 .H2PO4K-H(+) 0.756 4.034 3.051
   C04204  2,3-dihydroxybenzoylserine C10H11NO6 479.9710 .(H2PO4Na)2-H(+) 0.751 4.034 3.030
   C00105  UMP C9H13N2O9P 305.0201 -H2O-H(+) 0.614 4.900 3.010
   C05931  N2-Succinyl-L-glutamate C9H13NO7 366.0212 .H2PO4Na-H(+) 0.663 4.116 2.728
   C05925  Dihydroneopterin monophosphate C9H14N5O7P 567.9630 .(H2PO4)2KH-H(+) 0.760 3.582 2.722
   C05931  N2-Succinyl-L-glutamate C9H13NO7 381.9982 .H2PO4K-H(+) 0.713 3.704 2.643
   C05932  N2-Succinyl-L-glutamate 5-semialdehyde C9H13NO6 350.0314 .H2PO4Na-H(+) 0.654 3.819 2.499
   C04204  2,3-dihydroxybenzoylserine C10H11NO6 381.9982 .HPO4Na2-H(+) 0.663 3.704 2.458
   C00054  Adenosine 3',5'-bisphosphate C10H15N5O10P2 567.9630 .HPO4Na2-H(+) 0.666 3.582 2.387
   C00224  Adenosine 5'-phosphosulfate C10H14N5O10PS 567.9630 .HPO4Na2-H(+) 0.655 3.582 2.347
   C01302  1-(2-Carboxyphenylamino)-1-deoxy-D-ribulose 5-phosphate C12H16NO9P 366.0479 +OH(-) 0.647 3.541 2.290
   C04302  N-(5-Phospho-D-ribosyl)anthranilate C12H16NO9P 366.0479 +OH(-) 0.637 3.541 2.257
   C00112  CDP C9H15N3O11P2 423.9899 .H/Na-H(+) 0.614 3.647 2.239
   C00044  GTP C10H16N5O14P3 559.9477 .H/K-H(+) 0.597 3.454 0.000
   C00144  GMP C10H14N5O8P 344.0409 -H2O-H(+) 0.593 4.663 0.000
   C00054  Adenosine 3',5'-bisphosphate C10H15N5O10P2 426.0236 -H(+) 0.591 3.635 0.000
   C00112  CDP C9H15N3O11P2 402.0111 -H(+) 0.584 3.521 0.000
   C00575  cAMP C10H12N5O6P 567.9630 .(H2PO4Na)2-H(+) 0.578 3.582 0.000
   C00054  Adenosine 3',5'-bisphosphate C10H15N5O10P2 448.0070 .H/Na-H(+) 0.567 4.978 0.000
   C00105  UMP C9H13N2O9P 324.0362 [+1]-H(+) 0.565 4.402 0.000
   C03451  (R)-S-Lactoylglutathione C13H21N3O8S 498.0495 .H2PO4Na-H(+) 0.558 3.613 0.000
   C00361  dGDP C10H15N5O10P2 567.9630 .HPO4Na2-H(+) 0.534 3.582 0.000
   C00942  3',5'-Cyclic GMP C10H12N5O7P 366.0212 .H/Na-H(+) 0.523 4.116 0.000
   C00055  CMP C9H14N3O8P 304.0335 -H2O-H(+) 0.520 4.567 0.000
   C04462  N-Succinyl-2-L-amino-6-oxoheptanedioate C11H15NO8 559.9477 .(H2PO4K)2-H(+) 0.515 3.454 0.000
   C04114  crotonobetaine C7H13NO2 381.9982 .(H2PO4Na)2-H(+) 0.514 3.704 0.000
   C00361  dGDP C10H15N5O10P2 426.0236 -H(+) 0.510 3.635 0.000
   C00051  Reduced glutathione C10H17N3O6S 479.9710 .HPO4K2-H(+) 0.502 4.034 0.000
   C05198  5'-Deoxyadenosine C10H13N5O3 423.9899 .HPO4K2-H(+) 0.494 3.647 0.000
   C00362  dGMP C10H14N5O7P 328.0454 -H2O-H(+) 0.489 4.909 0.000
   C00035  GDP C10H15N5O11P2 479.9710 .H/K-H(+) 0.488 4.034 0.000
   C00361  dGDP C10H15N5O10P2 448.0070 .H/Na-H(+) 0.474 4.978 0.000
   C00559  Deoxyadenosine C10H13N5O3 423.9899 .HPO4K2-H(+) 0.450 3.647 0.000
   C00575  cAMP C10H12N5O6P 350.0314 .H/Na-H(+) 0.409 3.819 0.000
   C00931  Porphobilinogen C10H14N2O4 345.0429 .H2PO4Na-H(+) 0.000 4.799 0.000
   C01134  Pantetheine 4'-phosphate C11H23N2O7PS 477.0539 .H2PO4Na-H(+) 0.000 4.487 0.000
     KEGG pathway by CLR  
   Pathway_ion pvalue_ion qvalue_ion  Purine metabolism 2e-12 0.0000
  Pyrimidine metabolism 6e-06 0.0003
  Oxidative phosphorylation 0.0003 0.0082
  Sulfur metabolism 0.0004 0.0086
  Bisphenol degradation 0.002 0.0338
  Pantothenate and CoA biosynthesis 0.003 0.0503
  Methane metabolism 0.007 0.1028
     COG enrichment  
none  Predicted metabolites from CLR  
   Predicted metabolites Pvalue Overlap with hits  Thiamin monophosphate 0.0002 0.0000
  D-Fructose 1,6-bisphosphate 0.0005 0.0000
  Thiamin 0.0006 0.0000
  L-Tryptophan 0.001 0.0000
  butanesulfonate 0.005 0.0000
  dATP 0.005 0.0000
  dUTP 0.005 0.0000
  Hydrogen sulfide 0.005 0.0000
  Isethionic acid 0.005 0.0000
  L-Tyrosine 0.005 0.0000
  UTP 0.005 0.0000
  L-Threonine 0.007 0.0000
  dGTP 0.007 0.0000
  CTP 0.01 0.0000
  dCTP 0.01 0.0000
    
 
