# Supplementary material for: Genomewide landscape of gene–metabolome associations in Escherichia coli
Source: Mol Syst Biol. 2017 Jan 16;13(1):907. doi: 10.15252/msb.20167150 (PMC5293155; doi:10.15252/msb.20167150)
Supplement: Supplementary file 4 — Table EV3 [file MSB-13-907-s004.zip › details/data_yaiU.html]

 
 
 yaiU 
  yaiU - details 
 
 
  CLR  
   Gene_matching CLR_index  ymdF 8.6
  yahH 7.5
  yfbT 7.3
  ydfO 6.9
  ycdZ 6.8
  ycfP 6.7
  dhaK 6.6
  yceF 6.6
  yecT 6.5
  yebU 6.4
  yfbE 6.3
  zapA 6.3
  yliA 6.1
  ymfE 6.1
  ydaG 6.0
  ycdG 5.9
  ydcM 5.9
  mltD 5.8
  yccS 5.7
  nudD 5.6
  fepA 5.6
  ydbD 5.6
  rzpR 5.6
  hokD 5.6
  yfeT 5.5
  yebW 5.5
  ybbM 5.5
  ydeN 5.5
  yaiZ 5.4
  ybfG 5.4
  abgA 5.4
  yebV 5.2
  ymdC 5.2
  ydbL 5.2
  lar 5.1
  ybfH 5.1
  ynjC 5.1
  pfkB 5.1
  ybgO 5.0
  ymfT 5.0
  yfhK 4.9
  yjiQ 4.9
  dhaL 4.8
  kdpE 4.8
  ydaQ 4.8
  hisA 4.8
  nmpC 4.8
  mscL 4.8
  ymcD 4.7
  ydhB 4.7
  ykiB 4.7
  yphG 4.7
  mdtA 4.6
  eutA 4.6
  ymfI 4.6
  ycbQ 4.6
  ydhO 4.6
  ycgN 4.5
  yjhE 4.5
  fbaB 4.5
  yfcM 4.5
  ybiO 4.5
  htgA 4.5
  ydjY 4.5
  fsaA 4.5
  ligT 4.4
  sfcA 4.4
  ydbA 4.4
  ycjR 4.4
  bdm 4.4
  yccX 4.4
  ycfQ 4.4
  yjhV 4.4
  wzc 4.3
  yggM 4.3
  gatR 4.3
  yfeA 4.3
  yfiL 4.3
  otsA 4.3
  yajQ 4.3
  fimZ 4.3
  ymbA 4.3
  yegH 4.3
  uidC 4.2
  nadR 4.2
  yfaZ 4.2
  ybgQ 4.1
  glcB 4.1
  ycbF 4.1
  sseB 4.1
  ybjT 4.1
  yegX 4.1
  ynjB 4.1
  yahM 4.1
  eamA 4.1
  ylaC 4.0
  ymfO 4.0
  flgH 4.0
  yoaC 4.0
  ydcO 4.0
  sieB 4.0
  yeiW 3.9
  yfhQ 3.9
  ybdF 3.9
  paaH 3.9
  ynfH 3.9
  yohN 3.9
  ycaM 3.9
  tonB 3.8
  uspF 3.8
  yncA 3.8
  ymfA 3.8
  citF 3.8
  yehT 3.8
  ybcJ 3.8
  dmsA 3.7
  lsrG 3.7
  yphB 3.7
  marA 3.7
  yfjO 3.7
  rzpD 3.7
  yeaJ 3.7
  yedS 3.7
  ydeO 3.6
  yqeC 3.6
  yhhI 3.6
  yeaP 3.6
  yjhH 3.6
  ybiX 3.6
  betT 3.6
  yihW 3.6
  ydaM 3.6
  rhoL 3.5
  yfbJ 3.5
  yfgJ 3.5
  ybbB 3.5
  ybeB 3.5
  ypaA 3.5
  yeaN 3.5
  yncC 3.5
  dppB 3.5
  ydcI 3.4
  yecM 3.4
  trmC 3.4
  mgsA 3.4
  ygfB 3.4
  pbl 3.4
  wbbK 3.4
  ydiQ 3.4
  yoaB 3.3
  rimI 3.3
  ybcV 3.3
  gltI 3.3
  yajI 3.3
  ynbC 3.3
  yehU 3.3
  yajR 3.3
  dhaH 3.3
  yfhB 3.2
  yfcQ 3.2
  sufB 3.2
  dos 3.2
  ydhZ 3.2
  ycjD 3.2
  mhpT 3.1
  thrL 3.1
  pheL 3.1
  yohH 3.1
  yodB 3.1
  yeiI 3.1
  yphH 3.1
  yciE 3.1
  cysH 3.1
  ybiN 3.1
  ycdL 3.1
  ycgF 3.1
  yecD 3.1
  yagF 3.1
  rluB 3.0
  yohC 3.0
  essQ 3.0
  yceP 3.0
  yejF 3.0
  ydgJ 3.0
  hokA 3.0
  yfdL 3.0
     Differential ions  
none  KEGG pathway by CLR  
none  COG enrichment  
   Pathway_MS pvalue_MS qvalue_MS  Phosphonate and phosphinate metabolism 0.002 0.2497
     Predicted metabolites from CLR  
none 
 
