# Supplementary material for: Genomewide landscape of gene–metabolome associations in Escherichia coli
Source: Mol Syst Biol. 2017 Jan 16;13(1):907. doi: 10.15252/msb.20167150 (PMC5293155; doi:10.15252/msb.20167150)
Supplement: Supplementary file 4 — Table EV3 [file MSB-13-907-s004.zip › details/data_yajD.html]

 
 
 yajD 
  yajD - details 
 
 
  CLR  
   Gene_matching CLR_index  yagY 15.9
  radA 15.3
  ycaP 14.6
  ybiA 13.5
  ybdD 12.8
  nuoJ 12.0
  ydcU 11.0
  glcB 10.2
  sbp 10.1
  hslU 9.5
  thiP 9.5
  yaiE 9.4
  ybfB 9.3
  hisH 8.5
  ydcN 8.4
  ybeU 8.2
  ybjH 8.0
  ybdM 8.0
  mrp 7.8
  yajB 7.8
  pphB 7.6
  cheY 7.5
  yneG 7.5
  yqcD 7.5
  ycgV 7.5
  yeiM 7.4
  xdhB 7.2
  ynfE 6.9
  pykF 6.9
  ynaI 6.9
  kdgR 6.8
  ybhH 6.8
  tig 6.6
  yafJ 6.5
  eutL 6.1
  ycfD 6.1
  ggt 6.0
  hybD 5.9
  yhaH 5.9
  yneH 5.9
  ycdB 5.9
  ybcH 5.8
  eutA 5.7
  clpP 5.7
  ycbK 5.7
  ydhW 5.6
  nuoN 5.6
  ybjN 5.5
  yahE 5.5
  mazG 5.5
  clpS 5.3
  ygeY 5.3
  ybgP 5.1
  nuoA 5.0
  ygaX 5.0
  ypaA 5.0
  livK 5.0
  yagJ 4.9
  ybbB 4.8
  ycfK 4.8
  nlpD 4.7
  ygeH 4.7
  ybjO 4.7
  mscL 4.7
  ybjC 4.7
  ybiI 4.7
  yfdM 4.6
  torZ 4.6
  cheA 4.6
  ydjQ 4.6
  sodC 4.6
  ynbB 4.6
  elaB 4.5
  appB 4.4
  ycbZ 4.4
  sprT 4.3
  atoC 4.3
  yaiW 4.3
  yggM 4.2
  nuoF 4.2
  prpR 4.1
  pstA 4.1
  hycH 4.0
  hyfR 4.0
  yeaI 4.0
  bfr 4.0
  potE 4.0
  rhaT 4.0
  norV 3.9
  rstB 3.9
  yfjG 3.9
  nuoL 3.9
  pgpA 3.9
  cusF 3.8
  erfK 3.8
  sohB 3.7
  yddH 3.7
  menF 3.7
  gnd 3.7
  ycgL 3.7
  hyaD 3.7
  ykgJ 3.6
  yhbU 3.6
  clpX 3.6
  ompA 3.6
  eutG 3.5
  tesA 3.5
  yggT 3.4
  adhE 3.3
  ycgX 3.3
  ppdC 3.3
  yiaY 3.3
  yfdQ 3.2
  yfaP 3.2
  ubiE 3.2
  cydB 3.2
  ydhS 3.2
  yjeK 3.2
  clpA 3.2
  gpmI 3.1
  ydhC 3.1
  garD 3.1
  cybC 3.1
  uhpB 3.1
  xylA 3.1
  gadX 3.1
  yjdF 3.0
  glxR 3.0
  phoP 3.0
  yciS 3.0
  yeeU 3.0
     Differential ions  
   id name formula mz mod AUC Z-score Z-score AUC Weighted   C05973  2-Acyl-sn-glycero-3-phosphoethanolamine (n-C18:0) C23H48NO7P1 504.3154 .H/Na.H(+) 0.850 4.541 3.859
   C11453  2-C-methyl-D-erythritol 2,4-cyclodiphosphate C5H12O9P2 276.9897 -H(+) 0.886 4.074 3.611
   C00725  Lipoate C8H14O2S2 209.0552 [+2].H(+) 0.773 3.882 3.001
   C00417  cis-Aconitate C6H6O6 210.9633 .H/K-H(+) 0.747 3.927 2.935
   C04593  methylisocitrate C7H10O7 209.0552 [+2].H(+) 0.752 3.882 2.920
   C05973  2-Acyl-sn-glycero-3-phosphoethanolamine (n-C16:1) C21H42NO7P1 490.2320 .H/K.H(+) 0.709 3.943 2.795
   C05973  2-Acyl-sn-glycero-3-phosphoethanolamine (n-C16:1) C21H42NO7P1 490.2320 .K(+) 0.709 3.943 2.795
   C02225  2-Methylcitrate C7H10O7 209.0552 [+2].H(+) 0.714 3.882 2.773
   C00186  L-Lactate C3H6O3 328.9404 .(H2PO4Na)2-H(+) 0.649 4.197 2.724
   C01013  3-Hydroxypropanoate C3H6O3 328.9404 .(H2PO4Na)2-H(+) 0.647 4.197 2.714
   C01013  3-Hydroxypropanoate C3H6O3 208.9827 .H2PO4Na-H(+) 0.626 4.226 2.643
   C00184  Dihydroxyacetone C3H6O3 208.9827 .H2PO4Na-H(+) 0.621 4.226 2.624
   C00186  L-Lactate C3H6O3 208.9827 .H2PO4Na-H(+) 0.619 4.226 2.618
   C00262  Hypoxanthine C5H4N4O 120.0198 -NH3.H(+) 0.634 4.087 2.592
   C00042  Succinate C4H6O4 252.9454 .H2PO4K-H(+) 0.608 4.172 2.536
   C00170  5-Methylthioadenosine C11H15N5O3S 298.0963 .H(+) 0.616 3.940 2.428
   C00101  5,6,7,8-Tetrahydrofolate C19H23N7O6 664.1169 .(H2PO4)2NaH.H(+) 0.611 3.484 2.128
   C00446  alpha-D-Galactose 1-phosphate C6H13O9P 163.0601 -H3PO4.H(+) 0.577 -3.860 -0.000
   1,6-anhydrous-N-Acetylmuramate  1,6-anhydrous-N-Acetylmuramate C11H17NO7 298.0963 .H/Na.H(+) 0.574 3.940 0.000
   C02341  trans-Aconitate C6H6O6 210.9633 .H/K-H(+) 0.565 3.927 0.000
   C01672  1,5-Diaminopentane C5H14N2 105.1317 [+2].H(+) 0.561 3.871 0.000
   C00256  D-Lactate C3H6O3 208.9827 .H2PO4Na-H(+) 0.553 4.226 0.000
   C01530  octadecanoate (n-C18:0) C18H36O2 285.2795 .H(+) 0.549 4.655 0.000
   C07086  Phenylacetic acid C8H8O2 276.9897 .HPO4Na2-H(+) 0.549 4.074 0.000
   C02962  D-Allose 6-phosphate C6H13O9P 163.0601 -H3PO4.H(+) 0.549 -3.860 -0.000
   C00092  D-Glucose 6-phosphate C6H13O9P 163.0601 -H3PO4.H(+) 0.548 -3.860 -0.000
   C00130  IMP C10H13N4O8P 522.9553 .HPO4K2.H(+) 0.545 -3.485 -0.000
   C00184  Dihydroxyacetone C3H6O3 328.9404 .(H2PO4Na)2-H(+) 0.536 4.197 0.000
   C01449  7-aminomethyl-7-deazaguanine C7H9N5O 163.0601 -NH3.H(+) 0.532 -3.860 -0.000
   C05973  2-Acyl-sn-glycero-3-phosphoethanolamine (n-C16:1) C21H42NO7P1 453.2770 [+1].H(+) 0.531 7.665 0.000
   C00033  Acetate C2H4O2 200.9529 .HPO4Na2-H(+) 0.516 -3.546 -0.000
   C00103  D-Glucose 1-phosphate C6H13O9P 163.0601 -H3PO4.H(+) 0.508 -3.860 -0.000
   C00249  Hexadecanoate (n-C16:0) C16H32O2 257.2482 .H(+) 0.493 4.165 0.000
   C05973  2-Acyl-sn-glycero-3-phosphoethanolamine (n-C18:1) C23H46NO7P1 481.3114 [+1].H(+) 0.489 3.755 0.000
   C05973  2-Acyl-sn-glycero-3-phosphoethanolamine (n-C18:1) C23H46NO7P1 480.3073 .H(+) 0.489 4.427 0.000
   C00085  D-Fructose 6-phosphate C6H13O9P 163.0601 -H3PO4.H(+) 0.478 -3.860 -0.000
   C05973  2-Acyl-sn-glycero-3-phosphoethanolamine (n-C18:1) C23H46NO7P1 622.2547 .HPO4Na2.H(+) 0.464 4.283 0.000
   C02976  D-Fructose 1-phosphate C6H13O9P 163.0601 -H3PO4.H(+) 0.457 -3.860 -0.000
   C00577  D-Glyceraldehyde C3H6O3 208.9827 .H2PO4Na-H(+) 0.456 4.226 0.000
   C05512  Deoxyinosine C10H12N4O4 270.0919 [+1]+OH(-) 0.453 4.640 0.000
   C00641  1,2-Diacyl-sn-glycerol (dihexadec-9-enoyl, n-C16:1) C35H64O5 701.4083 .H2PO4K.H(+) 0.451 3.816 0.000
   C00337  (S)-Dihydroorotate C5H6N2O4 276.9897 .H2PO4Na-H(+) 0.447 4.074 0.000
   C00256  D-Lactate C3H6O3 328.9404 .(H2PO4Na)2-H(+) 0.443 4.197 0.000
   C00577  D-Glyceraldehyde C3H6O3 328.9404 .(H2PO4Na)2-H(+) 0.413 4.197 0.000
   C01551  Allantoin C4H6N4O3 276.9897 .H2PO4Na-H(+) 0.350 4.074 0.000
   C05512  Deoxyinosine C10H12N4O4 269.0883 +OH(-) 0.320 5.706 0.000
   C01177  1D-myo-Inositol 1-phosphate C6H13O9P 163.0601 -H3PO4.H(+) 0.000 -3.860 -0.000
   C02737  phosphatidylserine (ditetradec-7-enoyl, n-C14:1) C34H62N1O10P1 700.4066 [+2].Na(+) 0.000 3.714 0.000
   C00266  Glycolaldehyde C2H4O2 200.9529 .HPO4Na2-H(+) 0.634 -3.546 -2.248
   C00182  glycogen C6H10O5 163.0601 .H(+) 0.617 -3.860 -2.380
   C00275  D-Mannose 6-phosphate C6H13O9P 163.0601 -H3PO4.H(+) 0.658 -3.860 -2.539
   C18096  Allulose 6-phosphate C6H13O9P 163.0601 -H3PO4.H(+) 0.671 -3.860 -2.591
   C02225  2-Methylcitrate C7H10O7 163.0601 -CO2.H(+) 0.672 -3.860 -2.594
   C00636  D-Mannose 1-phosphate C6H13O9P 163.0601 -H3PO4.H(+) 0.695 -3.860 -2.684
   branching glycogen  branching glycogen C6H10O5 163.0601 .H(+) 0.700 -3.860 -2.701
   C00725  Lipoate C8H14O2S2 163.0601 -CO2.H(+) 0.765 -3.860 -2.952
   C01097  D-Tagatose 6-phosphate C6H13O9P 163.0601 -H3PO4.H(+) 0.782 -3.860 -3.018
   C04593  methylisocitrate C7H10O7 163.0601 -CO2.H(+) 0.836 -3.860 -3.227
     KEGG pathway by CLR  
   Pathway_ion pvalue_ion qvalue_ion  Lipoic acid metabolism 0 0.0000
  Fructose and mannose metabolism 3e-10 0.0000
  Amino sugar and nucleotide sugar metabolism 3e-07 0.0000
  Starch and sucrose metabolism 5e-07 0.0000
  Phosphotransferase system (PTS) 9e-06 0.0001
  Galactose metabolism 1e-05 0.0001
  Streptomycin biosynthesis 2e-05 0.0002
  Glycolysis / Gluconeogenesis 5e-05 0.0005
  Aminobenzoate degradation 0.0001 0.0009
  Inositol phosphate metabolism 0.0002 0.0012
  Polyketide sugar unit biosynthesis 0.0002 0.0013
  Fatty acid biosynthesis 0.0008 0.0048
  Fatty acid metabolism 0.001 0.0081
  Pentose phosphate pathway 0.002 0.0120
  Biosynthesis of unsaturated fatty acids 0.004 0.0194
  Pyruvate metabolism 0.008 0.0365
  Biosynthesis of secondary metabolites 0.008 0.0366
     COG enrichment  
   Pathway_MS pvalue_MS qvalue_MS  Oxidative phosphorylation 6e-06 0.0006
  Chloroalkane and chloroalkene degradation 4e-05 0.0017
  Naphthalene degradation 4e-05 0.0011
  Glycolysis / Gluconeogenesis 0.0005 0.0116
  D-Glutamine and D-glutamate metabolism 0.001 0.0203
  Arachidonic acid metabolism 0.001 0.0169
  Tyrosine metabolism 0.002 0.0243
  Glycerophospholipid metabolism 0.005 0.0549
  Ubiquinone and other terpenoid-quinone biosynthesis 0.009 0.0849
     Predicted metabolites from CLR  
   Predicted metabolites Pvalue Overlap with hits  2-Demethylmenaquinol 8 1e-05 0.0000
  2-Demethylmenaquinone 8 9e-05 0.0000
    
 
