# Supplementary material for: Genomewide landscape of gene–metabolome associations in Escherichia coli
Source: Mol Syst Biol. 2017 Jan 16;13(1):907. doi: 10.15252/msb.20167150 (PMC5293155; doi:10.15252/msb.20167150)
Supplement: Supplementary file 4 — Table EV3 [file MSB-13-907-s004.zip › details/data_yajO.html]

 
 
 yajO 
  yajO - details 
 
 
  CLR  
   Gene_matching CLR_index  ypdG 8.2
  ygcL 8.1
  yjeK 7.8
  cysH 7.3
  yahK 7.3
  betT 7.3
  ysgA 7.1
  panC 7.0
  gadX 7.0
  yadB 6.9
  glmM 6.7
  ybgA 6.7
  metE 6.3
  lysC 6.3
  pdxJ 6.3
  holD 6.3
  ygeR 6.3
  ilvB 6.2
  gspO 6.2
  metB 6.1
  agaB 5.9
  lsrC 5.8
  kbaY 5.8
  yfjR 5.8
  ilvA 5.8
  rfaF 5.7
  rnb 5.6
  yahC 5.6
  crp 5.6
  dedA 5.6
  mcrB 5.5
  ompR 5.5
  ymcA 5.5
  yohM 5.5
  yjhG 5.4
  yebT 5.4
  metL 5.4
  sbmC 5.4
  etp 5.3
  rbsD 5.2
  hsdS 5.2
  crcB 5.2
  rep 5.2
  cls 5.2
  nrfG 5.2
  yjeO 5.2
  rfaD 5.2
  yfcD 5.2
  ycgJ 5.1
  ynfM 5.0
  glpG 5.0
  fixC 5.0
  rsgA 5.0
  ptsP 5.0
  flgK 4.9
  hofB 4.9
  fruK 4.9
  abrB 4.9
  yhhM 4.8
  yihN 4.8
  cyaY 4.8
  ychF 4.8
  speA 4.8
  yjeH 4.8
  grxC 4.8
  ypfN 4.8
  yedK 4.7
  nlpI 4.7
  ydhP 4.7
  gspI 4.7
  yfiE 4.6
  rfaG 4.6
  yjfJ 4.6
  yeeP 4.6
  exbB 4.6
  spr 4.6
  ygeX 4.6
  ilvM 4.6
  rpsO 4.5
  yajG 4.5
  yhjR 4.5
  sfmC 4.5
  cof 4.4
  ycaL 4.4
  yhdN 4.4
  potA 4.4
  rfaJ 4.4
  yceD 4.4
  proX 4.4
  fucP 4.4
  mdtJ 4.4
  yojI 4.4
  spy 4.3
  rrmJ 4.3
  ydcH 4.3
  ybaO 4.3
  treC 4.3
  yjbJ 4.3
  pitB 4.3
  yieI 4.3
  yhhW 4.3
  cmtA 4.3
  damX 4.3
  aroH 4.3
  rof 4.3
  yjdF 4.2
  dnaQ 4.2
  blc 4.2
  frvA 4.2
  rpsT 4.2
  yhfY 4.2
  srlB 4.2
  exuT 4.2
  hybO 4.2
  yjhP 4.2
  yibL 4.2
  pfkA 4.2
  nudB 4.2
  thiQ 4.2
  alaS 4.2
  purC 4.2
  mdlA 4.1
  ccmH 4.1
  dinJ 4.1
  metH 4.1
  yjfL 4.1
  chbC 4.1
  yeeW 4.1
  yfjI 4.1
  dsrB 4.1
  yjiR 4.1
  ilvE 4.1
  idnT 4.1
  yhcA 4.1
  ydfO 4.1
  yahB 4.1
  glpC 4.1
  iscR 4.1
  yhcO 4.1
  ubiG 4.1
  yrbC 4.0
  rffG 4.0
  glyA 4.0
  ygcN 4.0
  yhbE 4.0
  malI 4.0
  yibG 4.0
  paaY 4.0
  yzgL 4.0
  fes 4.0
  ulaE 4.0
  uraA 3.9
  baeR 3.9
  fhuC 3.9
  fldB 3.9
  dcuC 3.9
  csiE 3.9
  dadA 3.9
  yheM 3.9
  torY 3.9
  pyrD 3.8
  ybcM 3.8
  fimI 3.8
  yfiF 3.8
  pdxA 3.8
  ecnA 3.8
  ybaX 3.8
  xdhB 3.8
  yifE 3.8
  ulaC 3.8
  edd 3.8
  yedP 3.8
  yegX 3.8
  alsE 3.7
  oppB 3.7
  bcsC 3.7
  astB 3.7
  yfeS 3.7
  ygjN 3.7
  yfhM 3.7
  yhhJ 3.7
  proW 3.7
  dipZ 3.7
  yhjC 3.7
  lpp 3.7
  ygfO 3.7
  yhbS 3.7
  emrK 3.7
  rlmB 3.7
  djlC 3.7
  yaaA 3.7
  trkG 3.7
  tig 3.7
  nanA 3.6
  flgC 3.6
  rffC 3.6
  ykfA 3.6
  yfiR 3.6
  cysB 3.6
  ydiZ 3.6
  panB 3.6
  yedN 3.6
  yicC 3.6
  ypdJ 3.6
  prmB 3.6
  yddL 3.6
  yfiB 3.6
  yccW 3.6
  ascF 3.6
  yeeR 3.6
  coaE 3.6
  mdtK 3.6
  metQ 3.6
  yqfA 3.6
  dicB 3.6
  cysU 3.5
  speB 3.5
  moaB 3.5
  dedD 3.5
  yciB 3.5
  mrcA 3.5
  oxyR 3.5
  ydeS 3.5
  xylB 3.5
  yjfK 3.5
  fliQ 3.5
  yjhF 3.5
  yjaB 3.5
  flgI 3.5
  yaaW 3.5
  rpmF 3.5
  yneG 3.5
  ppx 3.5
  yniC 3.5
  frlR 3.5
  fruB 3.5
  yjdO 3.5
  kbaZ 3.5
  ruvB 3.5
  prfC 3.4
  ybeD 3.4
  hemX 3.4
  yifK 3.4
  glcB 3.4
  cysN 3.4
  bioH 3.4
  topB 3.4
  syd 3.4
  emrB 3.4
  ydaS 3.4
  malF 3.4
  sodA 3.4
  hokC 3.4
  yfhL 3.4
  yehA 3.4
  ybfN 3.3
  uidA 3.3
  pgpA 3.3
  yfcV 3.3
  dacB 3.3
  ydgH 3.3
  yodA 3.3
  alr 3.3
  citG 3.3
  yigF 3.3
  rbsK 3.3
  queA 3.3
  ybgC 3.3
  yeaE 3.3
  yfcY 3.3
  tiaE 3.3
  ygcW 3.3
  entB 3.3
  speG 3.3
  yfcO 3.3
  yjeP 3.3
  ygdL 3.3
  bcp 3.3
  cysZ 3.2
  cchB 3.2
  trg 3.2
  ycdU 3.2
  phr 3.2
  tnaB 3.2
  rpoN 3.2
  fruA 3.2
  argH 3.2
  rfaL 3.2
  yidR 3.2
  ydhJ 3.2
  envZ 3.2
  ilvD 3.2
  hsdM 3.2
  ppiB 3.2
  yiiD 3.2
  arpA 3.2
  ycdT 3.2
  yrhA 3.1
  yjjM 3.1
  kefC 3.1
  ompL 3.1
  cvrA 3.1
  yjcO 3.1
  yghW 3.1
  fre 3.1
  cysI 3.1
  yfbM 3.1
  yhgE 3.1
  sdaC 3.1
  cedA 3.1
  ytfJ 3.1
  yfaQ 3.1
  flgE 3.1
  fadD 3.1
  lysA 3.1
  rihC 3.0
  trxA 3.0
  yagS 3.0
  yqeG 3.0
  ninE 3.0
  ascG 3.0
  cysP 3.0
  nuoN 3.0
  mgtA 3.0
  nlpE 3.0
  yaiI 3.0
     Differential ions  
   id name formula mz mod AUC Z-score Z-score AUC Weighted   C18239  cyclic pyranopterin monophosphate C10H14N5O8P 362.0530 -H(+) 0.816 3.688 3.010
   C00345  6-Phospho-D-gluconate C6H13O10P 394.9722 .H2PO4Na-H(+) 0.691 3.797 2.625
   C00224  Adenosine 5'-phosphosulfate C10H14N5O10PS 665.9260 .(H2PO4Na)2-H(+) 0.614 3.645 2.236
   C01216  2-Dehydro-3-deoxy-D-galactonate C6H10O6 394.9722 .(H2PO4)2NaH-H(+) 0.595 3.797 0.000
   C11143  Dimethyl sulfoxide C2H6OS 294.9457 .(H2PO4)2NaH-H(+) 0.564 -3.521 -0.000
   C00144  GMP C10H14N5O8P 362.0530 -H(+) 0.543 3.688 0.000
   C00204  2-Dehydro-3-deoxy-D-gluconate C6H10O6 394.9722 .(H2PO4)2NaH-H(+) 0.538 3.797 0.000
   C01419  Cys-Gly C5H10N2O3S 394.9722 .(H2PO4)2NaH-H(+) 0.485 3.797 0.000
   C01936  Maltohexaose C36H62O31 989.3097 -H(+) 0.476 4.145 0.000
   C00718  1,4-alpha-D-glucan C36H62O31 989.3097 -H(+) 0.462 4.145 0.000
   C00942  3',5'-Cyclic GMP C10H12N5O7P 362.0530 +OH(-) 0.439 3.688 0.000
   C00036  Oxaloacetate C4H4O5 152.9754 .H/Na-H(+) 0.660 -3.519 -2.322
     KEGG pathway by CLR  
   Pathway_ion pvalue_ion qvalue_ion  Arachidonic acid metabolism 6e-07 0.0001
  Sulfur relay system 4e-05 0.0021
  Folate biosynthesis 0.0002 0.0071
  Sphingolipid metabolism 0.0003 0.0074
  Purine metabolism 0.0007 0.0130
  Starch and sucrose metabolism 0.004 0.0628
     COG enrichment  
   Pathway_MS pvalue_MS qvalue_MS  Chlorocyclohexane and chlorobenzene degradation 0 0.0000
  Fluorobenzoate degradation 0 0.0000
  Pantothenate and CoA biosynthesis 3e-05 0.0008
  Lipopolysaccharide biosynthesis 0.0006 0.0118
  Fructose and mannose metabolism 0.002 0.0321
  Phosphotransferase system (PTS) 0.003 0.0346
  Valine, leucine and isoleucine biosynthesis 0.003 0.0311
  Sulfur metabolism 0.003 0.0304
  Lysine biosynthesis 0.007 0.0550
  Selenoamino acid metabolism 0.009 0.0606
     Predicted metabolites from CLR  
   Predicted metabolites Pvalue Overlap with hits  (S)-3-Methyl-2-oxopentanoate 0 0.0000
  Dephospho-CoA 0 0.0000
  3-Methyl-2-oxobutanoate 0.0004 0.0000
  D-Fructose 1-phosphate 0.0004 0.0000
  D-Tagatose 1,6-biphosphate 0.0004 0.0000
  4-Phospho-L-aspartate 0.0009 0.0000
  5-Methyltetrahydrofolate 0.0009 0.0000
  ADP-L-glycero-D-manno-heptose 0.003 0.0000
  Pyridoxine 5'-phosphate 0.003 0.0000
  O-Phospho-4-hydroxy-L-threonine 0.003 0.0000
  5,6,7,8-Tetrahydrofolate 0.003 0.0000
  1-deoxy-D-xylulose 5-phosphate 0.004 0.0000
  3'-Phosphoadenylyl sulfate 0.007 0.0000
  L-Idonate 0.007 0.0000
  Reduced riboflavin 0.007 0.0000
  Riboflavin 0.007 0.0000
  Adenosine 3',5'-bisphosphate 0.01 0.0000
    
 
