# Supplementary material for: Genomewide landscape of gene–metabolome associations in Escherichia coli
Source: Mol Syst Biol. 2017 Jan 16;13(1):907. doi: 10.15252/msb.20167150 (PMC5293155; doi:10.15252/msb.20167150)
Supplement: Supplementary file 4 — Table EV3 [file MSB-13-907-s004.zip › details/data_ybaP.html]

 
 
 ybaP 
  ybaP - details 
 
 
  CLR  
   Gene_matching CLR_index  ybjR 15.5
  ybdD 11.3
  zraS 10.5
  fiu 10.2
  ydeU 9.1
  ytjC 9.1
  gpmI 8.7
  nlpB 8.6
  tap 8.6
  appC 8.3
  rnt 8.2
  tktB 8.1
  nuoN 8.1
  cpxR 8.1
  mpaA 7.9
  cheY 7.7
  yddW 7.7
  uhpA 7.6
  rcsB 7.3
  rpiB 7.3
  ydhW 7.0
  ymcB 7.0
  pepN 6.9
  ymfD 6.9
  lsrF 6.8
  rssB 6.7
  ynbA 6.5
  ygdD 6.4
  poxB 6.4
  yfdV 6.4
  yfeU 6.4
  ygaR 6.3
  yagW 6.3
  yfbV 6.2
  yidZ 6.2
  frdD 6.1
  gltA 6.1
  ygeD 6.0
  yacL 5.9
  ydaL 5.7
  phoP 5.7
  rstB 5.7
  nuoA 5.7
  cusF 5.6
  yfjK 5.5
  ydiM 5.5
  degP 5.4
  yeaL 5.4
  ydcR 5.4
  eutT 5.4
  purR 5.2
  yfhA 5.2
  ompN 5.1
  uup 5.1
  cyoC 5.1
  nuoB 5.1
  norW 5.1
  cirA 5.1
  idnR 5.0
  frdB 5.0
  ygeF 5.0
  yegL 4.9
  ydeI 4.9
  frdC 4.9
  ydgC 4.8
  pgm 4.8
  ygaM 4.8
  evgS 4.8
  yecE 4.8
  yqeI 4.8
  ydcN 4.7
  sohB 4.7
  oppA 4.6
  ydiH 4.6
  frdA 4.6
  yjbG 4.6
  ybhE 4.6
  yeiE 4.6
  hupB 4.5
  yagP 4.5
  nuoK 4.5
  ptsG 4.5
  yfdR 4.5
  ybfQ 4.3
  ybgE 4.3
  phoB 4.3
  talA 4.3
  yagQ 4.3
  rbsB 4.3
  yfeD 4.3
  allC 4.2
  kdpD 4.2
  yghX 4.2
  ygcP 4.2
  appY 4.2
  yfhG 4.2
  narQ 4.2
  yedA 4.2
  fimD 4.2
  ycjT 4.1
  csgG 4.1
  yoaG 4.1
  yagU 4.1
  ydcA 4.0
  srlR 4.0
  talB 4.0
  tesA 4.0
  prpR 4.0
  yahL 3.9
  rzoR 3.9
  ycjY 3.8
  norV 3.8
  nadB 3.8
  rsxG 3.8
  yfdK 3.8
  rcsC 3.8
  ycbL 3.8
  lamB 3.8
  treR 3.8
  ydgK 3.7
  yaiS 3.7
  malG 3.7
  narL 3.7
  artP 3.7
  ycbK 3.7
  ycbS 3.7
  yaiY 3.6
  yphF 3.6
  ybcK 3.6
  clpP 3.6
  ycgL 3.6
  ydfZ 3.5
  ackA 3.5
  cadC 3.5
  gnsB 3.4
  ydfA 3.4
  clpX 3.4
  purF 3.4
  ybiV 3.4
  ycdN 3.4
  speE 3.4
  rhaR 3.3
  hepA 3.3
  sgbH 3.3
  ymfL 3.3
  feaR 3.3
  tsx 3.3
  ymfR 3.2
  yeaS 3.2
  yegX 3.2
  ccmB 3.2
  rstA 3.2
  lrp 3.2
  ydgJ 3.2
  slp 3.2
  acnA 3.2
  yecF 3.2
  ypjF 3.1
  mhpF 3.1
  cyoD 3.1
  yedV 3.1
  yfcC 3.1
  yejH 3.1
  ybfB 3.1
  mltA 3.1
  yecS 3.0
  lysR 3.0
  ydjM 3.0
  frc 3.0
     Differential ions  
   id name formula mz mod AUC Z-score Z-score AUC Weighted   C02341  trans-Aconitate C6H6O6 129.0199 -CO2-H(+) 0.996 3.927 3.912
   C11453  2-C-methyl-D-erythritol 2,4-cyclodiphosphate C5H12O9P2 276.9897 -H(+) 0.886 4.335 3.843
   C00417  cis-Aconitate C6H6O6 129.0199 -CO2-H(+) 0.955 3.927 3.751
   C06332  N-Acetylanthranilate C9H9NO3 180.0684 .H(+) 0.900 4.016 3.614
   C06156  D-Glucosamine 1-phosphate C6H14NO8P 180.0875 -HPO3.H(+) 0.993 3.606 3.580
   C06393  2,3-diaminopropionate C3H8N2O2 376.9333 .(H2PO4K)2.H(+) 0.988 3.594 3.550
   C04575  2,3-Dioxo-L-gulonate C6H8O7 147.0302 -CO2-H(+) 0.807 3.981 3.213
   C02780  2,5-diketo-D-gluconate C6H8O7 147.0302 -CO2-H(+) 0.771 3.981 3.071
   C06006  (S)-2-Aceto-2-hydroxybutanoate C6H10O4 380.9713 .(H2PO4)2KH.H(+) 0.686 4.201 2.882
   C00311  Isocitrate C6H8O7 147.0302 -CO2-H(+) 0.672 3.981 2.677
   C00966  2-Dehydropantoate C6H10O4 380.9713 .(H2PO4)2KH.H(+) 0.636 4.201 2.672
   C00129  Isopentenyl diphosphate C5H12O7P2 480.9220 .(H2PO4)2KH.H(+) 0.706 3.728 2.631
   C00235  Dimethylallyl diphosphate C5H12O7P2 480.9220 .(H2PO4)2KH.H(+) 0.706 3.728 2.631
   C05629  Phenylpropanoate C9H10O2 271.0372 .H2PO4Na.H(+) 0.730 3.566 2.604
   C01181  gamma-butyrobetaine C7H15NO2 146.1167 .H(+) 0.667 3.836 2.558
   Glycerophosphoserine  Glycerophosphoserine C6H14NO8P 180.0875 -HPO3.H(+) 0.703 3.606 2.535
   C00679  5-Dehydro-4-deoxy-D-glucarate C6H8O7 147.0302 -CO2-H(+) 0.602 3.981 2.395
   C00262  Hypoxanthine C5H4N4O 276.9736 .HPO4Na2-H(+) 0.638 3.719 2.373
   C00352  D-Glucosamine 6-phosphate C6H14NO8P 180.0875 -HPO3.H(+) 0.627 3.606 2.260
   C01099  L-Fuculose 1-phosphate C6H13O8P 380.9713 .H2PO4K.H(+) 0.576 4.201 0.000
   C00204  2-Dehydro-3-deoxy-D-gluconate C6H10O6 314.9952 .H2PO4K.H(+) 0.574 -3.455 -0.000
   C07086  Phenylacetic acid C8H8O2 276.9897 .HPO4Na2-H(+) 0.549 4.335 0.000
   C00158  Citrate C6H8O7 147.0302 -CO2-H(+) 0.548 3.981 0.000
   C00979  O-Acetyl-L-serine C5H9NO4 129.0199 -NH3-H(+) 0.528 3.927 0.000
   C01449  7-aminomethyl-7-deazaguanine C7H9N5O 180.0875 .H(+) 0.447 3.606 0.000
   C00337  (S)-Dihydroorotate C5H6N2O4 276.9897 .H2PO4Na-H(+) 0.447 4.335 0.000
   C16186  L-ascorbate-6-phosphate C6H9O9P 276.9736 .H/Na-H(+) 0.409 3.719 0.000
   C01551  Allantoin C4H6N4O3 276.9897 .H2PO4Na-H(+) 0.350 4.335 0.000
   C00217  D-Glutamate C5H9NO4 129.0199 -NH3-H(+) 0.000 3.927 0.000
   C01131  L-Rhamnulose 1-phosphate C6H13O8P 380.9713 .H2PO4K.H(+) 0.000 4.201 0.000
   C01216  2-Dehydro-3-deoxy-D-galactonate C6H10O6 314.9952 .H2PO4K.H(+) 0.705 -3.455 -2.435
     KEGG pathway by CLR  
   Pathway_ion pvalue_ion qvalue_ion  C5-Branched dibasic acid metabolism 2e-15 0.0000
  Ascorbate and aldarate metabolism 2e-05 0.0009
  Streptomycin biosynthesis 2e-05 0.0006
  Phosphotransferase system (PTS) 3e-05 0.0008
  Valine, leucine and isoleucine biosynthesis 7e-05 0.0014
  Amino sugar and nucleotide sugar metabolism 0.0004 0.0060
  Pentose and glucuronate interconversions 0.0008 0.0109
  Galactose metabolism 0.007 0.0840
     COG enrichment  
   Pathway_MS pvalue_MS qvalue_MS  Two-component system 6e-10 0.0000
  Oxidative phosphorylation 3e-09 0.0000
  Toluene degradation 2e-05 0.0006
  Citrate cycle (TCA cycle) 0.0001 0.0023
  Pentose phosphate pathway 0.0001 0.0018
  Microbial metabolism in diverse environments 0.002 0.0253
  Butanoate metabolism 0.004 0.0520
     Predicted metabolites from CLR  
   Predicted metabolites Pvalue Overlap with hits  Nitrous oxide 0 0.0000
  2-Demethylmenaquinone 8 8e-06 0.0000
  2-Demethylmenaquinol 8 1e-05 0.0000
  Sedoheptulose 7-phosphate 1e-05 0.0000
  D-Glycerate 2-phosphate 3e-05 0.0000
  D-Erythrose 4-phosphate 6e-05 0.0000
  3-Phospho-D-glycerate 0.0001 0.0000
  Nitric oxide 0.0001 0.0000
  Fumarate 0.0001 0.0000
  alpha-D-Ribose 5-phosphate 0.0002 0.0000
  D-Fructose 6-phosphate 0.001 0.0000
  Succinate 0.002 0.0000
  D-Glucose 6-phosphate 0.002 0.0000
  N-Acetyl-D-glucosamine 6-phosphate 0.003 0.0000
  Glyceraldehyde 3-phosphate 0.005 0.0000
  Citrate 0.006 1.0000
    
 
