# Supplementary material for: Genomewide landscape of gene–metabolome associations in Escherichia coli
Source: Mol Syst Biol. 2017 Jan 16;13(1):907. doi: 10.15252/msb.20167150 (PMC5293155; doi:10.15252/msb.20167150)
Supplement: Supplementary file 4 — Table EV3 [file MSB-13-907-s004.zip › details/data_ybbC.html]

 
 
 ybbC 
  ybbC - details 
 
 
  CLR  
   Gene_matching CLR_index  mviM 8.0
  wbbK 6.9
  lsrG 6.6
  ydhB 6.6
  ydeP 6.3
  yeeT 6.3
  yoaD 6.3
  ydeH 6.3
  ydeO 6.2
  hokD 6.2
  sseA 6.2
  kil 6.2
  hinT 6.1
  rsxC 6.1
  ybeH 6.0
  glcG 5.8
  yegH 5.7
  thrL 5.7
  yedY 5.5
  ycfJ 5.5
  wbbL 5.4
  yfbT 5.4
  ycjZ 5.4
  yeaP 5.4
  rem 5.4
  ylcG 5.3
  yfeR 5.2
  yhdZ 5.2
  yeiU 5.2
  ydhZ 5.1
  ynjC 5.1
  wbbI 5.1
  ychE 5.0
  bglH 5.0
  yegJ 5.0
  ynbC 5.0
  yeaH 5.0
  msyB 4.9
  ydiI 4.9
  yfcS 4.9
  cueO 4.9
  relE 4.9
  yfdP 4.9
  yqeK 4.9
  ymfN 4.8
  yecT 4.8
  yeiA 4.8
  ompG 4.8
  ybaJ 4.7
  narU 4.7
  crcA 4.7
  yehD 4.7
  yqgC 4.6
  yfaZ 4.6
  yhdA 4.6
  pbl 4.6
  yhhI 4.5
  pfkB 4.5
  nadR 4.5
  gidB 4.5
  rhsE 4.5
  yfbU 4.5
  gmhB 4.5
  ygfJ 4.5
  rspB 4.4
  ybgI 4.4
  ydeV 4.4
  alsA 4.4
  yphG 4.4
  nikE 4.4
  rhsA 4.4
  flhD 4.4
  yncG 4.3
  ymdF 4.3
  ydiK 4.3
  astC 4.3
  trmC 4.3
  yagH 4.3
  yeaN 4.3
  yqhA 4.3
  yebU 4.3
  gspD 4.2
  mutS 4.2
  yagE 4.2
  yhgG 4.2
  ydfO 4.2
  yhaC 4.2
  yadM 4.2
  ydeR 4.2
  fucK 4.2
  sufD 4.2
  yidF 4.2
  yadH 4.2
  ppdB 4.1
  yfcU 4.1
  ybiR 4.1
  yhfT 4.1
  dos 4.1
  yohO 4.1
  ynjB 4.1
  ecpD 4.1
  ycaQ 4.1
  yncC 4.1
  ykiA 4.0
  rhaS 4.0
  yehT 4.0
  xerC 4.0
  yfhK 4.0
  eamA 4.0
  deaD 4.0
  yfbE 4.0
  yfcM 4.0
  yfbJ 3.9
  yraN 3.9
  ydiQ 3.9
  mioC 3.9
  yehU 3.9
  slyA 3.9
  smtA 3.9
  yeiP 3.9
  yedS 3.8
  sfcA 3.8
  yfeW 3.8
  yeaD 3.8
  mltC 3.8
  agaW 3.8
  yfcQ 3.8
  yqeJ 3.8
  yggS 3.7
  gidA 3.7
  yagX 3.7
  ypjC 3.7
  yegK 3.7
  clcB 3.7
  yeeV 3.7
  lrhA 3.7
  mngR 3.7
  nudD 3.7
  fliR 3.7
  fliJ 3.6
  uspF 3.6
  yfdS 3.6
  yegD 3.6
  cutC 3.6
  yaiT 3.6
  yfhB 3.6
  yfiL 3.6
  fimG 3.6
  yagF 3.5
  nikA 3.5
  yliA 3.5
  livK 3.5
  iaaA 3.5
  yagT 3.5
  ygfS 3.5
  tdcC 3.5
  ygaY 3.5
  ycjD 3.5
  fbaB 3.5
  ygbF 3.5
  puuB 3.5
  yfhR 3.5
  ybfD 3.5
  yoaB 3.5
  pioO 3.4
  yfjD 3.4
  ygeP 3.4
  mdtI 3.4
  yjaA 3.4
  mdtB 3.4
  yobF 3.4
  malP 3.4
  tag 3.4
  ydjY 3.4
  yegP 3.4
  gadE 3.4
  ygcR 3.4
  ypdH 3.4
  ygcK 3.3
  yfgJ 3.3
  rhsD 3.3
  ybhQ 3.3
  yibF 3.3
  yhbO 3.3
  abgA 3.3
  mdtA 3.3
  malT 3.3
  ycdW 3.3
  yfeN 3.2
  ydiA 3.2
  yebV 3.2
  ydhO 3.2
  araH 3.2
  yeeO 3.2
  yidL 3.2
  manY 3.2
  ydeN 3.2
  hisB 3.2
  yejF 3.2
  ycdG 3.1
  yhjG 3.1
  dnaG 3.1
  caiA 3.1
  yoaC 3.1
  clpB 3.1
  glpR 3.0
  yjiZ 3.0
  ygfM 3.0
  epd 3.0
  dam 3.0
  ygeK 3.0
  pbpC 3.0
     Differential ions  
   id name formula mz mod AUC Z-score Z-score AUC Weighted   C01083  Trehalose C12H22O11 345.1336 [+2].H(+) 0.892 5.749 5.127
   C00208  Maltose C12H22O11 345.1336 [+2].H(+) 0.694 5.749 3.992
   C00243  Lactose C12H22O11 345.1336 [+2].H(+) 0.589 5.749 0.000
   C05402  Melibiose C12H22O11 345.1336 [+2].H(+) 0.478 5.749 0.000
   C04114  crotonobetaine C7H13NO2 100.1124 -CO2.H(+) 0.663 -4.067 -2.698
     KEGG pathway by CLR  
   Pathway_ion pvalue_ion qvalue_ion  C5-Branched dibasic acid metabolism 1e-09 0.0000
  Ascorbate and aldarate metabolism 3e-08 0.0000
  Galactose metabolism 9e-08 0.0000
  Starch and sucrose metabolism 1e-07 0.0000
  Phosphotransferase system (PTS) 2e-06 0.0000
  Pentose and glucuronate interconversions 4e-06 0.0001
  ABC transporters 6e-06 0.0001
  Bacterial chemotaxis 0.001 0.0142
  Valine, leucine and isoleucine biosynthesis 0.002 0.0180
  Porphyrin and chlorophyll metabolism 0.004 0.0403
     COG enrichment  
none  Predicted metabolites from CLR  
   Predicted metabolites Pvalue Overlap with hits  glucosyl-O-acetyl-rhamanosyl-N-acetylglucosamyl-undecaprenyl diphosphate 0 0.0000
  D-Fructose 1,6-bisphosphate 0.0006 0.0000
  nickel 0.001 0.0000
  Uracil 0.004 0.0000
  UDP 0.007 0.0000
    
 
