# Supplementary material for: Genomewide landscape of gene–metabolome associations in Escherichia coli
Source: Mol Syst Biol. 2017 Jan 16;13(1):907. doi: 10.15252/msb.20167150 (PMC5293155; doi:10.15252/msb.20167150)
Supplement: Supplementary file 4 — Table EV3 [file MSB-13-907-s004.zip › details/data_ybbM.html]

 
 
 ybbM 
  ybbM - details 
 
 
  CLR  
   Gene_matching CLR_index  ybiN 10.8
  ymfT 9.7
  ybiO 9.5
  nmpC 9.5
  ybfH 8.9
  rzpR 8.3
  yahM 8.2
  yccS 7.7
  ydfO 7.6
  lar 7.6
  ybcJ 7.2
  dhaH 7.2
  ycdG 6.8
  ydbL 6.8
  ycaI 6.6
  ymdF 6.6
  fimZ 6.6
  dhaK 6.5
  cadA 6.3
  kdpE 6.3
  ymfE 6.2
  icdC 6.0
  ymfA 5.9
  abgA 5.9
  puuA 5.9
  ymcD 5.7
  ycfQ 5.7
  yliA 5.6
  ybjI 5.6
  hlyE 5.5
  yaiU 5.5
  yaiZ 5.5
  yncJ 5.4
  paaD 5.3
  ybjT 5.3
  dppB 5.2
  yegX 5.1
  yccU 5.0
  ydaQ 4.9
  yeaV 4.9
  yggM 4.8
  rob 4.8
  ydjK 4.8
  ydiQ 4.8
  cbpM 4.8
  yecT 4.7
  yraK 4.7
  flgH 4.7
  rzpD 4.7
  plsX 4.7
  ypeA 4.7
  ymfI 4.7
  yajR 4.6
  yeeO 4.6
  yncK 4.5
  nudD 4.5
  ydcM 4.5
  ydgJ 4.5
  emrE 4.5
  yeaN 4.4
  yebW 4.4
  ydcO 4.4
  yahH 4.4
  ycgL 4.4
  yciW 4.4
  eamA 4.4
  fiu 4.4
  yjiY 4.4
  yebU 4.4
  ygfQ 4.4
  marB 4.3
  ydhB 4.3
  pppA 4.3
  ybaN 4.3
  ydcX 4.3
  ydhY 4.2
  ynjB 4.2
  yfcM 4.2
  yfbF 4.2
  ygaQ 4.2
  ydbD 4.1
  ynfK 4.1
  ymjA 4.1
  fsaA 4.1
  ykgH 4.1
  yegH 4.1
  ygaZ 4.0
  sieB 4.0
  ybgQ 4.0
  ykgC 4.0
  yceK 4.0
  yejH 4.0
  garP 4.0
  nadR 3.9
  ycdL 3.9
  ynfN 3.9
  ycjR 3.9
  yrhC 3.9
  malX 3.9
  yphG 3.9
  yfjO 3.8
  glvC 3.8
  zapA 3.8
  yebV 3.8
  yeaX 3.8
  ymbA 3.8
  ycbV 3.8
  yjhE 3.8
  yggC 3.8
  rtcB 3.8
  frlD 3.8
  nuoN 3.8
  yccX 3.7
  yphB 3.7
  yafE 3.7
  ycdZ 3.7
  mdtA 3.7
  yfaO 3.7
  chpB 3.7
  tonB 3.7
  yejA 3.6
  ydhO 3.6
  ylaC 3.6
  cusS 3.6
  yjeK 3.6
  dkgA 3.6
  tag 3.6
  yceP 3.6
  yajQ 3.6
  galR 3.6
  yfbT 3.6
  yahJ 3.5
  ynjC 3.5
  yhdX 3.5
  yciK 3.5
  ybgO 3.5
  tehB 3.5
  yfbE 3.5
  yegW 3.5
  glcB 3.5
  ynfH 3.4
  yehL 3.4
  ymfQ 3.4
  ydjY 3.4
  ycdN 3.4
  sseB 3.4
  yphH 3.4
  ycgG 3.4
  ydjH 3.4
  livM 3.4
  essQ 3.4
  ybbO 3.4
  ymfO 3.4
  yceF 3.4
  mhpT 3.4
  ydfV 3.3
  yehZ 3.3
  frsA 3.3
  yncE 3.2
  yoeB 3.2
  yebQ 3.2
  ydeN 3.2
  ydcV 3.2
  ynjD 3.2
  ymgG 3.2
  yfeA 3.2
  htgA 3.2
  yphF 3.2
  ycjM 3.1
  yehU 3.1
  livH 3.1
  msrA 3.1
  ykiB 3.1
  ycbL 3.1
  hokA 3.1
  ydeO 3.1
  yggN 3.1
  yncA 3.1
  ydaG 3.1
  yfjD 3.1
  ycbF 3.1
  ymfD 3.1
  ybgH 3.1
  fepA 3.1
  ydhZ 3.0
  ycaM 3.0
  yieM 3.0
  rhoL 3.0
  yhfT 3.0
  garK 3.0
     Differential ions  
none  KEGG pathway by CLR  
none  COG enrichment  
   Pathway_MS pvalue_MS qvalue_MS  Glycerolipid metabolism 0.002 0.1595
  Phosphonate and phosphinate metabolism 0.003 0.1302
     Predicted metabolites from CLR  
none 
 
