# Supplementary material for: Genomewide landscape of gene–metabolome associations in Escherichia coli
Source: Mol Syst Biol. 2017 Jan 16;13(1):907. doi: 10.15252/msb.20167150 (PMC5293155; doi:10.15252/msb.20167150)
Supplement: Supplementary file 4 — Table EV3 [file MSB-13-907-s004.zip › details/data_ybbN.html]

 
 
 ybbN 
  ybbN - details 
 
 
  CLR  
   Gene_matching CLR_index  kdpB 11.4
  etp 11.4
  yhjC 11.3
  bioH 11.2
  rpsO 10.9
  ycdT 10.7
  rpsT 10.0
  torS 9.9
  ycbG 9.9
  yfeS 9.5
  tsr 9.4
  cspE 9.1
  yieP 9.0
  mobB 8.7
  yciB 8.7
  ybfN 8.6
  ygcW 8.6
  dgoT 8.5
  yrhA 8.3
  marC 8.3
  yicM 8.3
  ascG 8.3
  emtA 8.2
  yiiF 8.2
  yjgB 8.1
  ubiG 8.1
  yceG 8.1
  amtB 8.0
  rtcA 8.0
  gpp 7.8
  ybbP 7.8
  mcrB 7.8
  gldA 7.7
  cspH 7.7
  ylbH 7.7
  yfdE 7.7
  ybiW 7.7
  glf 7.6
  ybjG 7.6
  kgtP 7.6
  gatZ 7.5
  ycdU 7.5
  yjjM 7.5
  trmA 7.5
  ybfA 7.3
  guaD 7.3
  malF 7.2
  mcrC 7.1
  ycgJ 7.0
  yfeH 7.0
  yjiA 6.9
  fkpA 6.8
  aphA 6.8
  ydbJ 6.8
  yjfN 6.8
  tfaS 6.7
  ulaE 6.6
  ybfF 6.6
  yeeP 6.6
  yjgF 6.6
  yedW 6.5
  hemX 6.5
  ilvA 6.4
  yhjX 6.3
  yiiM 6.3
  gntU 6.3
  aceK 6.3
  yhiM 6.2
  tdcR 6.2
  yrfD 6.2
  hokC 6.2
  intE 6.1
  baeR 6.1
  ypeB 6.1
  ycfS 6.1
  abrB 6.0
  yidR 6.0
  tufA 6.0
  fhuC 6.0
  aidB 6.0
  citA 6.0
  yqjF 6.0
  yjiT 6.0
  yncH 6.0
  ybfE 6.0
  yicI 5.9
  yaaY 5.9
  ycbU 5.9
  gspJ 5.9
  pitB 5.9
  ydeQ 5.9
  elaD 5.9
  yhhW 5.9
  lldP 5.8
  yfdN 5.8
  yibI 5.8
  yhbY 5.7
  ydcE 5.7
  pcnB 5.7
  cbrC 5.6
  aaeX 5.6
  ulaG 5.5
  rpsU 5.5
  dcuC 5.5
  modE 5.5
  prfB 5.4
  potG 5.4
  gspO 5.4
  slt 5.4
  mutY 5.4
  yfdY 5.4
  tnaB 5.3
  ivbL 5.3
  yddM 5.3
  nadA 5.3
  fdoH 5.3
  yjeK 5.3
  ypfH 5.3
  yddG 5.3
  fldB 5.3
  yhiP 5.2
  yneF 5.2
  fdnH 5.2
  gadX 5.2
  flgK 5.2
  yjiK 5.2
  ygcL 5.2
  folX 5.2
  udp 5.1
  yobA 5.1
  ynfG 5.1
  hchA 5.1
  hybG 5.1
  glcB 5.1
  narZ 5.1
  nikR 5.1
  ydhL 5.1
  ydhM 5.1
  ynjA 5.1
  yciI 5.0
  ycdH 5.0
  hcaT 5.0
  lipB 5.0
  acrB 5.0
  yiaU 5.0
  pnp 5.0
  ybiM 5.0
  norR 5.0
  yicJ 5.0
  fucP 5.0
  yjfK 5.0
  oxyR 4.9
  tfaD 4.9
  hokE 4.9
  rbsD 4.9
  thiC 4.9
  serB 4.9
  ysgA 4.9
  ydfW 4.8
  narH 4.8
  setC 4.8
  yjeM 4.8
  pflA 4.8
  ade 4.8
  yghK 4.8
  yphC 4.8
  cld 4.7
  metC 4.7
  blr 4.7
  ymfP 4.7
  ygdI 4.7
  ushA 4.7
  yddL 4.7
  yjdF 4.7
  proY 4.7
  yidH 4.7
  ykgE 4.7
  cbpA 4.6
  yneK 4.6
  tnaA 4.6
  yehD 4.6
  ymgH 4.6
  agaS 4.6
  ychM 4.6
  hipA 4.6
  flhB 4.6
  yohG 4.6
  hcaR 4.6
  argO 4.6
  ygeR 4.5
  kbaY 4.5
  zitB 4.5
  yahO 4.5
  aroK 4.5
  panC 4.5
  ygcS 4.5
  holD 4.5
  ytfK 4.5
  wbbH 4.5
  glxK 4.4
  bipA 4.4
  yjfP 4.4
  yfcV 4.4
  glcF 4.4
  ilvM 4.4
  asnC 4.4
  yghO 4.4
  yfdO 4.4
  rtn 4.4
  betT 4.4
  cysB 4.4
  flhA 4.4
  wcaI 4.3
  ybjS 4.3
  ykfB 4.3
  lysC 4.3
  ybgS 4.3
  hypE 4.3
  pqqL 4.3
  ilvB 4.3
  cheA 4.3
  yedM 4.3
  djlC 4.3
  fic 4.3
  yqeG 4.2
  uxaB 4.2
  glnD 4.2
  yibK 4.2
  flhE 4.2
  yqhH 4.2
  fhiA 4.2
  yfiR 4.2
  ascF 4.2
  asnA 4.2
  bglJ 4.2
  yigL 4.2
  fecE 4.2
  yjfZ 4.1
  ynfC 4.1
  mhpD 4.1
  yjhX 4.1
  csiE 4.1
  ytfJ 4.1
  yeeY 4.1
  yjfL 4.1
  hipB 4.1
  purH 4.1
  ytfH 4.1
  gspG 4.1
  rhtA 4.1
  ynfD 4.1
  ynbE 4.0
  dmsC 4.0
  yjiO 4.0
  ycdS 4.0
  ybfC 4.0
  ygeO 4.0
  prmB 4.0
  cysH 4.0
  macB 4.0
  yeeJ 4.0
  speB 4.0
  mdlB 4.0
  yebN 4.0
  trxA 4.0
  ptsP 4.0
  ydfE 3.9
  yhbS 3.9
  fixB 3.9
  lpxM 3.9
  yihO 3.9
  nikB 3.9
  ybfL 3.9
  rpmF 3.9
  yhbE 3.9
  flgF 3.9
  gudX 3.9
  ydiY 3.9
  avtA 3.8
  yeeA 3.8
  narY 3.8
  yjiP 3.8
  asr 3.8
  ycjU 3.8
  ytfQ 3.8
  dkgB 3.8
  nhaA 3.8
  yeaT 3.8
  metE 3.8
  ygcI 3.8
  yieG 3.8
  yahK 3.8
  dnaQ 3.8
  ygjM 3.8
  ilvY 3.8
  yiaL 3.7
  yedJ 3.7
  yicS 3.7
  ccmH 3.7
  yejO 3.7
  rof 3.7
  ycbW 3.7
  sfmH 3.7
  yghA 3.7
  yjhT 3.7
  ybeA 3.7
  kbaZ 3.7
  narG 3.7
  nuoC 3.7
  osmC 3.7
  fdnG 3.6
  hdfR 3.6
  yegR 3.6
  yrbG 3.6
  mobA 3.6
  amiA 3.6
  rstA 3.6
  yahG 3.6
  yafZ 3.6
  ykgB 3.6
  nanA 3.6
  hcr 3.6
  yeiT 3.6
  leuB 3.6
  ymfS 3.6
  agaB 3.6
  codB 3.6
  ypjM 3.6
  yegX 3.6
  secB 3.6
  kefA 3.6
  ydfZ 3.5
  yajL 3.5
  flgM 3.5
  glnQ 3.5
  cvrA 3.5
  yidX 3.5
  yicC 3.5
  rumA 3.5
  cmtA 3.5
  dmsB 3.5
  yhaO 3.5
  sdhB 3.5
  rarD 3.5
  ydcH 3.5
  glnB 3.5
  tatB 3.5
  yohL 3.4
  fliS 3.4
  ppc 3.4
  ilvD 3.4
  fumA 3.4
  ilvE 3.4
  uraA 3.4
  yhfX 3.4
  cyaY 3.4
  rzoD 3.4
  fhuD 3.4
  recQ 3.4
  nlpE 3.4
  thrB 3.4
  yhcO 3.4
  rna 3.4
  yoeA 3.4
  yidI 3.4
  dhaR 3.4
  yohD 3.4
  dmsD 3.4
  ycgZ 3.4
  yraN 3.4
  htgA 3.3
  bolA 3.3
  yhaM 3.3
  tktB 3.3
  yqhC 3.3
  ygfM 3.3
  yneG 3.3
  cysZ 3.3
  ycgR 3.3
  ydfO 3.3
  ykfC 3.3
  gshA 3.3
  yieI 3.3
  agaI 3.3
  yfjP 3.3
  tfaQ 3.3
  yfcX 3.3
  barA 3.3
  dnaJ 3.3
  gspH 3.3
  edd 3.3
  yieL 3.3
  prfC 3.2
  yagB 3.2
  nudD 3.2
  crp 3.2
  yhaL 3.2
  srlB 3.2
  yhcA 3.2
  yedQ 3.2
  sucD 3.2
  rluD 3.2
  yidJ 3.2
  yfbE 3.2
  yhjR 3.2
  fruK 3.2
  ypfJ 3.2
  yciU 3.2
  nrdH 3.2
  entB 3.2
  metR 3.2
  dppA 3.2
  sfcA 3.2
  hsdS 3.2
  cchA 3.2
  znuA 3.1
  yaiF 3.1
  yadN 3.1
  wcaC 3.1
  idnK 3.1
  betI 3.1
  yeeZ 3.1
  dadX 3.1
  yjeO 3.1
  fruB 3.1
  thiG 3.1
  nuoN 3.1
  csgD 3.1
  caiC 3.1
  rumB 3.1
  yciT 3.1
  ychQ 3.1
  napH 3.1
  deoB 3.1
  ynjC 3.0
  mgtA 3.0
  ycgH 3.0
  yecT 3.0
  hisH 3.0
  osmE 3.0
  yzgL 3.0
  ymdF 3.0
  secG 3.0
  rfaJ 3.0
  gmd 3.0
  yebA 3.0
  ybdH 3.0
     Differential ions  
   id name formula mz mod AUC Z-score Z-score AUC Weighted   d-biotin d-sulfoxide  d-biotin d-sulfoxide C10H16N2O4S 261.0815 [+2]-H(+) 0.988 3.478 3.435
   C00120  Biotin C10H16N2O3S 261.0815 +OH(-) 0.904 3.478 3.143
   C02341  trans-Aconitate C6H6O6 412.9263 .(H2PO4Na)2-H(+) 0.540 -3.516 -0.000
   C00033  Acetate C2H4O2 292.9218 .(H2PO4)2KH-H(+) 0.530 -3.812 -0.000
   C00299  Uridine C9H12N2O6 261.0815 +OH(-) 0.431 3.478 0.000
   C00266  Glycolaldehyde C2H4O2 292.9218 .(H2PO4)2KH-H(+) 0.626 -3.812 -2.385
   C00417  cis-Aconitate C6H6O6 412.9263 .(H2PO4Na)2-H(+) 0.854 -3.516 -3.002
   C11453  2-C-methyl-D-erythritol 2,4-cyclodiphosphate C5H12O9P2 412.9263 .H2PO4K-H(+) 0.883 -3.516 -3.105
     KEGG pathway by CLR  
   Pathway_ion pvalue_ion qvalue_ion  Lysine degradation 9e-05 0.0098
     COG enrichment  
   Pathway_MS pvalue_MS qvalue_MS  Chlorocyclohexane and chlorobenzene degradation 0 0.0000
  Fluorobenzoate degradation 0 0.0000
  Valine, leucine and isoleucine biosynthesis 0.0005 0.0130
  Bacterial secretion system 0.002 0.0330
  Protein export 0.005 0.0749
     Predicted metabolites from CLR  
   Predicted metabolites Pvalue Overlap with hits  (S)-3-Methyl-2-oxopentanoate 0 0.0000
  bis-molybdopterin guanine dinucleotide 0 0.0000
  tungsten bispterin cofactor guanine dinucleotide 0 0.0000
  molybdopterin guanine dinucleotide 0 0.0000
  3-Methyl-2-oxobutanoate 0.0003 0.0000
  D-Tagatose 1,6-biphosphate 0.0003 0.0000
  5-Amino-1-(5-Phospho-D-ribosyl)imidazole-4-carboxamide 0.0008 0.0000
  Aerobactin 0.0008 0.0000
  bis-molybdenum cofactor 0.0008 0.0000
  tungsten bispterin cofactor 0.0008 0.0000
  coprogen 0.0008 0.0000
  Fe(III)hydroxamate 0.0008 0.0000
  Ferrichrome 0.0008 0.0000
  6-Phospho-D-gluconate 0.003 0.0000
  bis-molybdopterin mono-guanine dinucleotide 0.003 0.0000
  tungsten bispterin cofactor mono-guanine dinucleotide 0.003 0.0000
  GDP-D-mannose 0.003 0.0000
  L-Lactate 0.006 0.0000
  Uracil 0.006 0.0000
  Acetol 0.007 0.0000
  D-Fructose 1-phosphate 0.007 0.0000
  molybdenum cofactor 0.007 0.0000
  2-Oxobutanoate 0.009 0.0000
  Dimethyl sulfide 0.009 0.0000
  Dimethyl sulfoxide 0.009 0.0000
  nickel 0.009 0.0000
  trimethylamine 0.009 0.0000
  Trimethylamine N-oxide 0.009 0.0000
  L-Tryptophan 0.009 0.0000
    
 
