# Supplementary material for: Genomewide landscape of gene–metabolome associations in Escherichia coli
Source: Mol Syst Biol. 2017 Jan 16;13(1):907. doi: 10.15252/msb.20167150 (PMC5293155; doi:10.15252/msb.20167150)
Supplement: Supplementary file 4 — Table EV3 [file MSB-13-907-s004.zip › details/data_ybcL.html]

 
 
 ybcL 
  ybcL - details 
 
 
  CLR  
   Gene_matching CLR_index  rem 9.8
  ydfO 9.3
  yphH 8.4
  yegX 8.0
  yebV 7.9
  mutM 7.9
  yfbT 7.6
  gidA 6.8
  yodB 6.7
  yafY 6.5
  nudD 6.3
  ybeH 6.3
  sseB 5.8
  uspF 5.8
  apaG 5.8
  yecT 5.8
  yfdL 5.7
  yeaD 5.7
  yfbE 5.6
  yjeK 5.6
  ycdR 5.6
  yfiP 5.4
  ydiK 5.4
  ycjM 5.4
  yeaJ 5.3
  yphG 5.3
  yfgI 5.3
  azoR 5.2
  hisM 5.2
  ydhZ 5.2
  dam 5.2
  hinT 5.2
  dinG 5.2
  pioO 5.2
  gnsB 5.1
  cueO 5.1
  yebW 5.1
  sfcA 5.0
  yedZ 5.0
  yagF 4.9
  ynjI 4.9
  yeiW 4.9
  fiu 4.9
  ydhO 4.8
  yfcM 4.8
  yliH 4.8
  yoaC 4.8
  yeiA 4.8
  yfhK 4.7
  ycdW 4.7
  mioC 4.7
  yegD 4.6
  yebU 4.6
  pfkB 4.6
  yidZ 4.6
  wbbI 4.5
  ynjC 4.5
  xerC 4.5
  ydjY 4.5
  cysH 4.5
  marA 4.4
  mutT 4.4
  ymfA 4.4
  ycdC 4.4
  ycjZ 4.4
  yegH 4.4
  intD 4.3
  ydhB 4.3
  lrhA 4.3
  betT 4.2
  yoaD 4.2
  ymdF 4.2
  ybiR 4.2
  hisA 4.2
  ydiQ 4.2
  yeaH 4.2
  yqeJ 4.1
  panC 4.1
  udk 4.1
  mdtA 4.1
  ydfJ 4.1
  yohN 4.1
  yfdP 4.1
  yncC 4.1
  ycfJ 4.1
  eamA 4.0
  ynjE 4.0
  yfcU 4.0
  essQ 4.0
  ybfH 4.0
  ydeN 4.0
  yfhB 4.0
  yfdC 4.0
  ydiA 3.9
  yahN 3.9
  otsA 3.9
  hokD 3.9
  ydjH 3.9
  yfcE 3.8
  gatR 3.8
  yehL 3.8
  yfeW 3.8
  ydiF 3.8
  trmC 3.8
  ydiI 3.8
  yecM 3.8
  yfiL 3.8
  garK 3.8
  phnM 3.7
  sufB 3.7
  fbaB 3.7
  uhpA 3.7
  ykgN 3.7
  ygcR 3.7
  sufD 3.7
  ycfQ 3.7
  rspB 3.7
  yfeA 3.7
  nmpC 3.7
  holD 3.6
  yfgJ 3.6
  rtcB 3.6
  yccE 3.6
  glpR 3.6
  yeiP 3.6
  rhlB 3.6
  yodD 3.6
  yecD 3.5
  ydgJ 3.5
  asnB 3.5
  yahH 3.5
  yqiG 3.5
  yehU 3.5
  yjbB 3.5
  pbpG 3.5
  mutS 3.4
  glmM 3.4
  mdtB 3.4
  yegK 3.4
  wbbK 3.4
  yfjS 3.4
  tktB 3.4
  slyA 3.4
  yohH 3.4
  yfaZ 3.4
  tap 3.3
  yeaN 3.3
  mscL 3.3
  yjhS 3.3
  yohC 3.3
  yhbO 3.3
  ykiA 3.3
  yhaC 3.3
  yoaB 3.3
  ypdA 3.3
  clcB 3.3
  ychE 3.3
  lsrG 3.2
  yegI 3.2
  ybhQ 3.2
  yahM 3.2
  ycdL 3.2
  yqgC 3.2
  yfaL 3.2
  ymgG 3.2
  setB 3.2
  ydeH 3.2
  yjiE 3.1
  tdcC 3.1
  yciC 3.1
  yfcS 3.1
  yneE 3.1
  pdxJ 3.1
  rsxC 3.1
  ptsP 3.1
  ynjB 3.1
  wbbL 3.1
  yphB 3.1
  yjfJ 3.0
  citF 3.0
  tolC 3.0
  ypdH 3.0
  yaiT 3.0
  yehT 3.0
  yeeT 3.0
  yebB 3.0
  rfaZ 3.0
  ygcL 3.0
  yfgH 3.0
  yfjO 3.0
  fucA 3.0
     Differential ions  
   id name formula mz mod AUC Z-score Z-score AUC Weighted   C00534  Pyridoxamine C8H12N2O2 342.9775 .HPO4K2.H(+) 0.979 5.051 4.946
   C04593  methylisocitrate C7H10O7 342.9775 .H2PO4K.H(+) 0.895 5.051 4.521
   C00534  Pyridoxamine C8H12N2O2 207.0518 .H/K.H(+) 0.861 4.798 4.131
   C00725  Lipoate C8H14O2S2 342.9775 .H2PO4K.H(+) 0.748 5.051 3.777
   C06156  D-Glucosamine 1-phosphate C6H14NO8P 298.0067 .H/K.H(+) 0.967 3.746 3.624
   C02225  2-Methylcitrate C7H10O7 342.9775 .H2PO4K.H(+) 0.626 5.051 3.159
   C00670  sn-Glycero-3-phosphocholine C8H20NO6P 258.1109 .H(+) 0.628 4.725 2.969
   C00062  L-Arginine C6H14N4O2 213.0755 .H/K.H(+) 0.620 4.367 2.707
   C00062  L-Arginine C6H14N4O2 213.0755 .K(+) 0.620 4.367 2.707
   C00937  D-Lactaldehyde C3H6O2 98.0310 [+1].Na(+) 0.751 3.556 2.672
   C05235  Acetol C3H6O2 98.0310 [+1].Na(+) 0.735 3.556 2.615
   C00163  Propionate (n-C3:0) C3H6O2 98.0310 [+1].Na(+) 0.679 3.556 2.416
   C00424  L-Lactaldehyde C3H6O2 98.0310 [+1].Na(+) 0.659 3.556 2.343
   C01602  Ornithine C5H12N2O2 253.0484 .H2PO4Na.H(+) 0.634 3.629 2.301
   C00719  Glycine betaine C5H11NO2 118.0861 .H(+) 0.640 3.460 2.214
   C00183  L-Valine C5H11NO2 118.0861 .H(+) 0.635 3.460 2.196
   C00054  Adenosine 3',5'-bisphosphate C10H15N5O10P2 465.9992 .H/K.H(+) 0.593 -3.524 -0.000
   C04666  D-erythro-1-(Imidazol-4-yl)glycerol 3-phosphate C6H11N2O6P 359.0022 .H2PO4Na.H(+) 0.588 5.325 0.000
   C02291  L-Cystathionine C7H14N2O4S 359.0022 .H2PO4K.H(+) 0.582 5.325 0.000
   Glycerophosphoserine  Glycerophosphoserine C6H14NO8P 298.0067 .H/K.H(+) 0.571 3.746 0.000
   C02225  2-Methylcitrate C7H10O7 207.0518 .H(+) 0.549 4.798 0.000
   C04593  methylisocitrate C7H10O7 207.0518 .H(+) 0.527 4.798 0.000
   C00074  Phosphoenolpyruvate C3H5O6P 304.9335 .H2PO4K.H(+) 0.527 -4.144 -0.000
   C00055  CMP C9H14N3O8P 465.9992 .HPO4Na2.H(+) 0.525 -3.524 -0.000
   C00352  D-Glucosamine 6-phosphate C6H14NO8P 298.0067 .H/K.H(+) 0.517 3.746 0.000
   C00361  dGDP C10H15N5O10P2 465.9992 .H/K.H(+) 0.478 -3.524 -0.000
   C00725  Lipoate C8H14O2S2 207.0518 .H(+) 0.439 4.798 0.000
   C01468  p-Cresol C7H8O 342.9775 .(H2PO4)2KH.H(+) 0.430 5.051 0.000
   C00575  cAMP C10H12N5O6P 465.9992 .H2PO4K.H(+) 0.612 -3.524 -2.156
   C05932  N2-Succinyl-L-glutamate 5-semialdehyde C9H13NO6 465.9992 .(H2PO4)2KH.H(+) 0.748 -3.524 -2.636
   C03263  Coproporphyrinogen III C36H44N4O8 663.3308 [+2].H(+) 0.684 -4.105 -2.808
   C00612  N1-Acetylspermidine C9H21N3O 330.1233 .HPO4Na2.H(+) 0.706 -4.021 -2.838
   C01029  N8-Acetylspermidine C9H21N3O 330.1233 .HPO4Na2.H(+) 0.706 -4.021 -2.838
   C00344  Phosphatidylglycerol (ditetradec-7-enoyl, n-C14:1) C34H63O10P1 685.3927 .H/Na.H(+) 0.809 -3.519 -2.847
   C03232  3-Phosphohydroxypyruvate C3H5O7P 304.9335 .H2PO4Na.H(+) 0.714 -4.144 -2.960
     KEGG pathway by CLR  
   Pathway_ion pvalue_ion qvalue_ion  Lipoic acid metabolism 0 0.0000
  Propanoate metabolism 7e-06 0.0003
  Lysine biosynthesis 9e-06 0.0003
  Lysine degradation 0.0006 0.0141
  Sulfur metabolism 0.0007 0.0120
  ABC transporters 0.002 0.0273
  Valine, leucine and isoleucine degradation 0.003 0.0363
  Pyruvate metabolism 0.005 0.0587
  Pantothenate and CoA biosynthesis 0.006 0.0578
  C5-Branched dibasic acid metabolism 0.007 0.0573
  Aminoacyl-tRNA biosynthesis 0.007 0.0521
  Valine, leucine and isoleucine biosynthesis 0.007 0.0523
  Vitamin B6 metabolism 0.007 0.0483
  Nicotinate and nicotinamide metabolism 0.01 0.0581
     COG enrichment  
   Pathway_MS pvalue_MS qvalue_MS  Mismatch repair 0.006 0.5739
     Predicted metabolites from CLR  
   Predicted metabolites Pvalue Overlap with hits  glucosyl-O-acetyl-rhamanosyl-N-acetylglucosamyl-undecaprenyl diphosphate 0 0.0000
  [4Fe-4S] iron-sulfur cluster 0.0006 0.0000
  SufBCD with bound [4Fe-4S] cluster 0.0006 0.0000
  [2Fe-1S] desulfurated iron-sulfur cluster 0.001 0.0000
  [2Fe-2S] iron-sulfur cluster 0.001 0.0000
  D-Fructose 1,6-bisphosphate 0.001 0.0000
  UDP 0.001 0.0000
  SufBCD with two bound [2Fe-2S] clusters 0.001 0.0000
  UDPglucose 0.001 0.0000
  (R)-Glycerate 0.003 0.0000
  CMP 0.005 1.0000
    
 
