# Supplementary material for: Genomewide landscape of gene–metabolome associations in Escherichia coli
Source: Mol Syst Biol. 2017 Jan 16;13(1):907. doi: 10.15252/msb.20167150 (PMC5293155; doi:10.15252/msb.20167150)
Supplement: Supplementary file 4 — Table EV3 [file MSB-13-907-s004.zip › details/data_ybcN.html]

 
 
 ybcN 
  ybcN - details 
 
 
  CLR  
   Gene_matching CLR_index  gspO 11.4
  yjeK 11.2
  pfkA 10.2
  yehA 9.8
  yfcD 9.6
  pepE 9.2
  coaE 8.9
  ybdJ 8.7
  yneG 8.6
  yiaU 8.4
  yagU 8.3
  yddL 8.2
  flgK 8.2
  yjdF 8.2
  yifK 7.9
  flgE 7.8
  yhbS 7.7
  yneK 7.6
  treC 7.4
  pal 7.2
  eutL 7.2
  mrcB 7.1
  hemX 7.0
  hcaT 7.0
  yafZ 6.7
  yfgL 6.7
  gadX 6.6
  yeeW 6.5
  yfaQ 6.5
  ydaF 6.5
  yfiM 6.5
  pck 6.4
  evgS 6.4
  ydiV 6.4
  ykfA 6.4
  ydiY 6.3
  yfcP 6.3
  yahO 6.2
  ykfB 6.1
  pgm 6.1
  ybjD 6.1
  cysH 6.0
  ynbE 6.0
  ygeR 5.9
  holD 5.9
  osmE 5.9
  hflC 5.7
  yfbH 5.7
  fldB 5.7
  yedK 5.6
  ggt 5.5
  crp 5.4
  betT 5.4
  yagZ 5.4
  yaiO 5.4
  panC 5.4
  frc 5.4
  rnb 5.4
  rpoN 5.3
  tnaA 5.3
  pdxJ 5.2
  ydhC 5.2
  yjfL 5.2
  rhtA 5.1
  ybeR 5.1
  yraQ 5.0
  iadA 4.9
  yjeO 4.9
  ccmH 4.9
  yjfK 4.9
  yjfJ 4.9
  yahC 4.8
  ilvB 4.8
  ulaC 4.7
  ydgK 4.7
  etp 4.7
  yeaT 4.7
  nlpE 4.7
  pgi 4.6
  hycI 4.6
  ynfG 4.6
  fruK 4.5
  hsdS 4.5
  ptsP 4.5
  yagB 4.5
  ycfH 4.4
  ybiU 4.4
  metL 4.4
  yegQ 4.4
  ygcN 4.3
  ymfR 4.3
  glmM 4.3
  ybdZ 4.3
  yqjD 4.3
  yaaU 4.3
  ulaB 4.3
  ylbE 4.3
  srlB 4.2
  ycbU 4.2
  secB 4.2
  yecA 4.2
  yfaW 4.1
  yraO 4.1
  ybdR 4.1
  ompR 4.1
  cof 4.1
  tig 4.1
  yaiI 4.0
  ysgA 4.0
  yedP 4.0
  glgP 4.0
  metB 4.0
  yahG 4.0
  zntA 4.0
  ybcH 4.0
  dadX 3.9
  ybiT 3.9
  melR 3.8
  yoaE 3.8
  fliH 3.8
  yceD 3.8
  yfcV 3.8
  ilvA 3.7
  ushA 3.7
  yhbE 3.7
  yahK 3.7
  rep 3.7
  pepB 3.7
  ycdQ 3.7
  hflX 3.7
  ydfZ 3.6
  ymcA 3.6
  yfaT 3.6
  cusR 3.6
  treA 3.6
  ruvB 3.6
  dnaQ 3.6
  ygeL 3.6
  yfhM 3.6
  yliI 3.6
  pflC 3.6
  yeaB 3.6
  ygcL 3.6
  ypdG 3.5
  phoQ 3.5
  yfeD 3.5
  narZ 3.5
  yfaP 3.5
  ybiC 3.4
  ldcA 3.4
  prlC 3.4
  gcd 3.4
  mrcA 3.4
  yajB 3.4
  ccmF 3.4
  ybbD 3.4
  yjfY 3.4
  rbsD 3.4
  yjeH 3.4
  aroP 3.3
  aceB 3.3
  cheB 3.3
  ybcQ 3.3
  ybcO 3.3
  pflA 3.3
  ilvY 3.3
  yieI 3.2
  hcaC 3.2
  fhuC 3.2
  yfaU 3.2
  yfeO 3.2
  yfgC 3.2
  ybaM 3.1
  mgtA 3.1
  yjfC 3.1
  yiaK 3.1
  hydN 3.1
  ybcI 3.1
  yjhD 3.1
  yggV 3.1
  gmd 3.1
  sgcB 3.0
  ygeX 3.0
  nac 3.0
  yhbW 3.0
  yfcA 3.0
  ycfD 3.0
  ilvM 3.0
  ygbT 3.0
  nrfC 3.0
  yraP 3.0
  ybeT 3.0
  ychF 3.0
  creC 3.0
     Differential ions  
   id name formula mz mod AUC Z-score Z-score AUC Weighted   C05198  5'-Deoxyadenosine C10H13N5O3 426.0048 .HPO4K2.H(+) 0.554 3.905 0.000
   C00112  CDP C9H15N3O11P2 426.0048 .H/Na.H(+) 0.551 3.905 0.000
   C00204  2-Dehydro-3-deoxy-D-gluconate C6H10O6 299.0245 .H2PO4Na.H(+) 0.540 -3.612 -0.000
   C00559  Deoxyadenosine C10H13N5O3 426.0048 .HPO4K2.H(+) 0.519 3.905 0.000
   C04462  N-Succinyl-2-L-amino-6-oxoheptanedioate C11H15NO8 426.0048 .H2PO4K.H(+) 0.506 3.905 0.000
   C01216  2-Dehydro-3-deoxy-D-galactonate C6H10O6 299.0245 .H2PO4Na.H(+) 0.495 -3.612 -0.000
   C00345  6-Phospho-D-gluconate C6H13O10P 299.0245 .H/Na.H(+) 0.723 -3.612 -2.612
     KEGG pathway by CLR  
   Pathway_ion pvalue_ion qvalue_ion  Ubiquinone and other terpenoid-quinone biosynthesis 0.001 0.1258
  Histidine metabolism 0.002 0.0894
  Porphyrin and chlorophyll metabolism 0.003 0.0858
  Naphthalene degradation 0.003 0.0875
  Biosynthesis of secondary metabolites 0.006 0.1136
     COG enrichment  
   Pathway_MS pvalue_MS qvalue_MS  Chlorocyclohexane and chlorobenzene degradation 0 0.0000
  Fluorobenzoate degradation 0 0.0000
  Pantothenate and CoA biosynthesis 0.0001 0.0035
  Fructose and mannose metabolism 0.0003 0.0059
  Arachidonic acid metabolism 0.003 0.0425
  Ascorbate and aldarate metabolism 0.004 0.0604
  Starch and sucrose metabolism 0.005 0.0576
  D-Alanine metabolism 0.008 0.0770
  C5-Branched dibasic acid metabolism 0.009 0.0799
  DNA replication 0.009 0.0719
  Pentose phosphate pathway 0.009 0.0676
     Predicted metabolites from CLR  
   Predicted metabolites Pvalue Overlap with hits  2-Oxobutanoate 0.0004 0.0000
  Phenylpropanoate 0.0005 0.0000
  L-ascorbate-6-phosphate 0.001 0.0000
  L-Homoserine 0.001 0.0000
  Undecaprenyl-diphospho-N-acetylmuramoyl-(N-acetylglucosamine)-L-ala-D-glu-meso-2,6-diaminopimeloyl-D-ala-D-ala 0.001 0.0000
  L-Cysteine 0.002 0.0000
  D-Fructose 1,6-bisphosphate 0.003 0.0000
  Undecaprenyl diphosphate 0.004 0.0000
  D-Glucose 6-phosphate 0.004 0.0000
  nickel 0.005 0.0000
  L-Tryptophan 0.005 0.0000
  D-Alanine 0.009 0.0000
    
 
