# Supplementary material for: Genomewide landscape of gene–metabolome associations in Escherichia coli
Source: Mol Syst Biol. 2017 Jan 16;13(1):907. doi: 10.15252/msb.20167150 (PMC5293155; doi:10.15252/msb.20167150)
Supplement: Supplementary file 4 — Table EV3 [file MSB-13-907-s004.zip › details/data_ybdD.html]

 
 
 ybdD 
  ybdD - details 
 
 
  CLR  
   Gene_matching CLR_index  radA 13.5
  yajD 12.8
  yaiE 11.5
  ybaP 11.3
  nuoJ 11.1
  ycaP 11.1
  ycdB 10.0
  hslU 9.5
  cheY 9.0
  ygaX 8.4
  ybdM 8.3
  glcB 8.1
  yedA 7.9
  yagY 7.7
  ydjG 7.6
  yqeI 7.4
  ynaI 7.3
  ycbZ 7.1
  yeiM 7.1
  tig 7.0
  ydeU 6.9
  yneG 6.9
  rcsB 6.8
  thiP 6.7
  norV 6.7
  ytjC 6.6
  ybjR 6.6
  atoS 6.6
  ycbC 6.5
  ybeT 6.4
  ydhS 6.4
  ynjF 6.3
  ydaT 6.3
  degP 6.3
  ybeL 6.2
  xdhB 6.2
  hisH 6.2
  eutG 6.1
  phoQ 6.1
  nuoA 6.1
  yfdV 6.0
  ybcH 5.8
  hybD 5.8
  cusF 5.8
  nuoN 5.8
  pphB 5.7
  ybdN 5.7
  yggM 5.7
  ypfG 5.6
  ydcU 5.6
  ycfD 5.6
  yddW 5.5
  yagP 5.5
  yeaB 5.5
  ybiS 5.5
  kdpD 5.4
  ycbK 5.3
  bfr 5.3
  bioA 5.3
  ybiI 5.3
  envZ 5.3
  ybjC 5.3
  yfeU 5.2
  eutL 5.1
  zraS 5.1
  cusR 5.0
  torZ 5.0
  pykF 5.0
  lsrF 5.0
  rstB 4.8
  nuoF 4.8
  yeeS 4.8
  ycgL 4.7
  yecE 4.7
  zwf 4.6
  yhcD 4.6
  yceA 4.6
  atoC 4.5
  ynbA 4.5
  rssB 4.5
  secG 4.4
  yajB 4.4
  ydhC 4.4
  elaB 4.4
  yoaH 4.4
  yafJ 4.4
  phoR 4.3
  yqcD 4.3
  ppdC 4.3
  ydfA 4.3
  feaR 4.3
  gpmI 4.2
  yceD 4.2
  yebE 4.2
  ypaA 4.2
  ybgL 4.2
  yddH 4.1
  yfiN 4.1
  nuoL 4.0
  yjbG 4.0
  ycfK 4.0
  ynfE 4.0
  nlpB 4.0
  dld 4.0
  ycbY 4.0
  ptsG 3.9
  sbp 3.9
  yncI 3.9
  yodC 3.9
  ybfB 3.9
  ybeU 3.8
  alkA 3.8
  ydiR 3.8
  phoB 3.8
  yqaD 3.8
  ackA 3.7
  rnt 3.7
  allC 3.7
  yneH 3.7
  yecH 3.7
  yciS 3.7
  yqeB 3.7
  ydiE 3.7
  galU 3.7
  xylA 3.7
  ygaP 3.6
  ccmB 3.6
  adhE 3.6
  ydfZ 3.6
  yhaH 3.6
  mpaA 3.5
  mrp 3.5
  garD 3.5
  frmB 3.5
  ypjF 3.4
  hyfR 3.4
  eutA 3.4
  ydjQ 3.4
  chbG 3.4
  nlpD 3.4
  ybiA 3.4
  ggt 3.3
  nrdD 3.3
  yfgL 3.3
  yagQ 3.3
  ydhW 3.3
  dctA 3.3
  artP 3.3
  ydhT 3.3
  atoB 3.3
  cfa 3.3
  ycbS 3.3
  yliG 3.3
  galM 3.3
  yfjQ 3.2
  coaE 3.2
  rpiB 3.2
  nagC 3.2
  pgm 3.1
  yoaE 3.1
  yjdJ 3.1
  gltA 3.1
  pstB 3.1
  yagW 3.1
  priB 3.0
  hflX 3.0
  ybhH 3.0
  evgS 3.0
  ydjM 3.0
  tpiA 3.0
  mhpF 3.0
     Differential ions  
   id name formula mz mod AUC Z-score Z-score AUC Weighted   C05973  2-Acyl-sn-glycero-3-phosphoethanolamine (n-C18:0) C23H48NO7P1 504.3154 .H/Na.H(+) 0.850 5.643 4.796
   C06156  D-Glucosamine 1-phosphate C6H14NO8P 180.0875 -HPO3.H(+) 0.993 3.607 3.581
   C06393  2,3-diaminopropionate C3H8N2O2 376.9333 .(H2PO4K)2.H(+) 0.988 3.612 3.568
   C11514  E-3-carboxy-2-pentenedioate 6-methyl ester C7H8O6 428.9521 .(H2PO4Na)2.H(+) 0.866 4.084 3.538
   C00178  Thymine C5H6N2O2 358.9423 .(H2PO4)2KH-H(+) 0.810 3.663 2.968
   C04225  cis-2-Methylaconitate C7H8O6 428.9521 .(H2PO4Na)2.H(+) 0.706 4.084 2.883
   C00129  Isopentenyl diphosphate C5H12O7P2 480.9220 .(H2PO4)2KH.H(+) 0.706 3.841 2.711
   C00235  Dimethylallyl diphosphate C5H12O7P2 480.9220 .(H2PO4)2KH.H(+) 0.706 3.841 2.711
   C04823  (S)-2-[5-Amino-1-(5-phospho-D-ribosyl)imidazole-4-carboxamido]succinate C13H19N4O12P 453.0710 -H(+) 0.731 3.678 2.689
   C00627  Pyridoxine 5'-phosphate C8H12NO6P 272.0388 .H/Na.H(+) 0.668 3.956 2.643
   C04677  5-Amino-1-(5-Phospho-D-ribosyl)imidazole-4-carboxamide C9H15N4O8P 457.0148 .H2PO4Na-H(+) 0.701 3.620 2.537
   Glycerophosphoserine  Glycerophosphoserine C6H14NO8P 180.0875 -HPO3.H(+) 0.703 3.607 2.535
   C02983  N-Methyltryptophan C12H14N2O2 457.0148 .(H2PO4Na)2-H(+) 0.692 3.620 2.506
   C06424  tetradecanoate (n-C14:0) C14H28O2 401.0960 .HPO4K2-H(+) 0.639 3.767 2.406
   C01181  gamma-butyrobetaine C7H15NO2 146.1167 .H(+) 0.667 3.604 2.404
   C03274  Glycerophosphoglycerol C6H15O8P 480.9645 .(H2PO4)2KH.H(+) 0.680 3.476 2.363
   C01337  XDP C10H14N4O12P2 616.9060 .HPO4K2-H(+) 0.655 3.498 2.291
   C00459  dTTP C10H17N2O14P3 616.9060 .H2PO4K-H(+) 0.650 3.498 2.275
   C00352  D-Glucosamine 6-phosphate C6H14NO8P 180.0875 -HPO3.H(+) 0.627 3.607 2.260
   C05402  Melibiose C12H22O11 359.1199 +OH(-) 0.607 3.459 2.101
   C00243  Lactose C12H22O11 359.1199 +OH(-) 0.561 3.459 0.000
   C01083  Trehalose C12H22O11 359.1199 +OH(-) 0.561 3.459 0.000
   C00204  2-Dehydro-3-deoxy-D-gluconate C6H10O6 299.0245 .H2PO4Na.H(+) 0.540 -4.199 -0.000
   ferroxamine minus Fe(3)  ferroxamine minus Fe(3) C25H48N6O8 561.3595 .H(+) 0.532 5.413 0.000
   C00438  N-Carbamoyl-L-aspartate C5H8N2O5 158.0105 -NH3-H(+) 0.497 3.841 0.000
   C01216  2-Dehydro-3-deoxy-D-galactonate C6H10O6 299.0245 .H2PO4Na.H(+) 0.495 -4.199 -0.000
   C00208  Maltose C12H22O11 359.1199 +OH(-) 0.448 3.459 0.000
   C01449  7-aminomethyl-7-deazaguanine C7H9N5O 180.0875 .H(+) 0.447 3.607 0.000
   C00132  Methanol CH4O1 172.9566 .HPO4Na2-H(+) 0.405 3.688 0.000
   C00249  Hexadecanoate (n-C16:0) C16H32O2 257.2389 [+2]-H(+) 0.667 -3.813 -2.545
   C00345  6-Phospho-D-gluconate C6H13O10P 299.0245 .H/Na.H(+) 0.723 -4.199 -3.037
     KEGG pathway by CLR  
   Pathway_ion pvalue_ion qvalue_ion  Phosphotransferase system (PTS) 2e-07 0.0000
  Amino sugar and nucleotide sugar metabolism 8e-06 0.0004
  Streptomycin biosynthesis 3e-05 0.0011
  Selenoamino acid metabolism 0.0002 0.0041
  Galactose metabolism 0.0004 0.0085
  Biosynthesis of secondary metabolites 0.0005 0.0075
  Terpenoid backbone biosynthesis 0.002 0.0239
  Alanine, aspartate and glutamate metabolism 0.002 0.0269
     COG enrichment  
   Pathway_MS pvalue_MS qvalue_MS  Glycolysis / Gluconeogenesis 5e-07 0.0000
  Two-component system 9e-06 0.0003
  Pyruvate metabolism 0.0002 0.0045
  Microbial metabolism in diverse environments 0.0003 0.0049
  Xylene degradation 0.0008 0.0126
  Taurine and hypotaurine metabolism 0.002 0.0203
  Dioxin degradation 0.002 0.0174
  D-Glutamine and D-glutamate metabolism 0.002 0.0189
  Inositol phosphate metabolism 0.002 0.0168
  Arachidonic acid metabolism 0.002 0.0152
  Benzoate degradation 0.002 0.0140
  Oxidative phosphorylation 0.002 0.0137
  Chloroalkane and chloroalkene degradation 0.006 0.0339
  Naphthalene degradation 0.006 0.0315
  Terpenoid backbone biosynthesis 0.006 0.0294
     Predicted metabolites from CLR  
   Predicted metabolites Pvalue Overlap with hits  D-Glycerate 2-phosphate 3e-05 0.0000
  3-Phospho-D-glycerate 0.0001 0.0000
  2-Demethylmenaquinone 8 0.0003 0.0000
  2-Demethylmenaquinol 8 0.0004 0.0000
  D-Lactate 0.0009 0.0000
  UTP 0.0009 0.0000
  D-Glucose 6-phosphate 0.002 0.0000
  N-Acetyl-D-glucosamine 6-phosphate 0.003 0.0000
  D-Glucose 1-phosphate 0.006 0.0000
    
 
