# Supplementary material for: Genomewide landscape of gene–metabolome associations in Escherichia coli
Source: Mol Syst Biol. 2017 Jan 16;13(1):907. doi: 10.15252/msb.20167150 (PMC5293155; doi:10.15252/msb.20167150)
Supplement: Supplementary file 4 — Table EV3 [file MSB-13-907-s004.zip › details/data_ybdH.html]

 
 
 ybdH 
  ybdH - details 
 
 
  CLR  
   Gene_matching CLR_index  yedJ 11.5
  glxK 8.4
  yjfP 7.7
  modE 7.6
  wcaI 7.4
  amtB 7.4
  yjiK 7.3
  yfeH 7.3
  hchA 7.2
  aceK 7.1
  yobA 7.1
  rzoD 7.0
  barA 6.8
  hcaR 6.8
  dhaR 6.7
  pqqL 6.7
  yrhA 6.7
  yhjC 6.5
  cld 6.5
  ybiM 6.4
  aroK 6.4
  yidR 6.4
  narY 6.3
  ycbG 6.3
  gpp 6.3
  oxyR 6.2
  yhbY 6.0
  ycbW 6.0
  yhjX 5.9
  yicJ 5.9
  ygcW 5.8
  ybfN 5.7
  yicI 5.7
  yfdY 5.7
  setC 5.7
  hokC 5.7
  yohG 5.6
  ydcE 5.6
  yihS 5.5
  malF 5.5
  ivbL 5.5
  ymgH 5.5
  ascG 5.5
  ynfC 5.5
  ypfJ 5.5
  ydfW 5.5
  trmA 5.4
  abrB 5.4
  ybfA 5.4
  ykgG 5.4
  aaeX 5.4
  ygcS 5.3
  rpsO 5.3
  dgoT 5.3
  yfjP 5.2
  proY 5.2
  ycdU 5.2
  bglJ 5.1
  gspH 5.1
  baeR 5.0
  ydhL 4.9
  yedM 4.8
  ydbJ 4.8
  gatZ 4.8
  mutY 4.7
  gldA 4.7
  rpsT 4.7
  yidX 4.6
  bioH 4.5
  eutP 4.5
  rtcA 4.5
  mobB 4.5
  hyfJ 4.5
  yaaY 4.4
  ybjG 4.4
  aidB 4.4
  ybbP 4.4
  marC 4.3
  ubiG 4.3
  yneF 4.3
  yieP 4.2
  ruvC 4.2
  rpsU 4.1
  ylbH 4.1
  ydhM 4.0
  mcrB 4.0
  tfaS 4.0
  idnK 4.0
  ybjS 4.0
  fic 4.0
  wcaE 3.9
  gntU 3.9
  ltaE 3.9
  fdhD 3.9
  djlC 3.9
  caiC 3.9
  yghO 3.9
  fkpA 3.8
  ybfE 3.8
  ybiW 3.8
  entF 3.8
  melB 3.8
  ytfK 3.8
  yiiM 3.7
  gspG 3.7
  ulaE 3.7
  yeeA 3.7
  yddM 3.7
  yhiM 3.7
  ulaG 3.7
  yeeY 3.7
  yedQ 3.6
  kdsC 3.6
  leuB 3.6
  ybeA 3.6
  potG 3.6
  trpE 3.6
  nuoC 3.6
  emtA 3.6
  torS 3.5
  csiE 3.5
  yicM 3.4
  lipB 3.4
  gss 3.4
  yjiT 3.4
  ydeQ 3.4
  glcB 3.4
  ycgJ 3.4
  yiiF 3.4
  yheO 3.4
  ynfD 3.4
  ygjQ 3.4
  ycdH 3.4
  hipB 3.3
  gor 3.3
  yghA 3.3
  fhiA 3.3
  yfeS 3.3
  hipA 3.3
  yjiP 3.3
  ychM 3.2
  ypfH 3.2
  yjdI 3.2
  yceG 3.2
  yqjI 3.2
  etp 3.2
  ybdL 3.2
  yjjM 3.2
  ydbK 3.1
  dmsD 3.1
  putA 3.1
  ypeB 3.1
  yedX 3.1
  ygdI 3.1
  fliO 3.1
  yicS 3.0
  wcaC 3.0
  ybaT 3.0
  yeeP 3.0
  yebN 3.0
  appA 3.0
  tnaB 3.0
  ybbN 3.0
  ilvH 3.0
     Differential ions  
   id name formula mz mod AUC Z-score Z-score AUC Weighted   C00631  D-Glycerate 2-phosphate C3H7O7P 184.9849 -H(+) 0.901 4.899 4.415
   C02341  trans-Aconitate C6H6O6 129.0199 -CO2-H(+) 0.996 3.625 3.611
   C01279  4-Amino-5-hydroxymethyl-2-methylpyrimidine C6H9N3O 138.0662 -H(+) 0.816 4.336 3.537
   C04778  N1-(5-Phospho-alpha-D-ribosyl)-5,6-dimethylbenzimidazole C14H19N2O7P 477.0539 .H2PO4Na-H(+) 0.735 4.719 3.470
   C00417  cis-Aconitate C6H6O6 129.0199 -CO2-H(+) 0.955 3.625 3.462
   C00197  3-Phospho-D-glycerate C3H7O7P 184.9849 -H(+) 0.697 4.899 3.414
   C00054  Adenosine 3',5'-bisphosphate C10H15N5O10P2 643.9583 .(H2PO4)2NaH-H(+) 0.633 5.112 3.238
   C03539  S-Ribosyl-L-homocysteine C9H17NO6S 439.9659 .HPO4K2-H(+) 0.769 4.159 3.200
   C00224  Adenosine 5'-phosphosulfate C10H14N5O10PS 643.9583 .(H2PO4)2NaH-H(+) 0.622 5.112 3.180
   C00942  3',5'-Cyclic GMP C10H12N5O7P 381.9982 .H/K-H(+) 0.861 3.645 3.138
   C00361  dGDP C10H15N5O10P2 643.9583 .(H2PO4)2NaH-H(+) 0.612 5.112 3.127
   C04556  4-Amino-2-methyl-5-phosphomethylpyrimidine C6H10N3O4P 138.0662 -HPO3-H(+) 0.696 4.336 3.018
   C00288  Bicarbonate CH2O3 196.9264 .H2PO4K-H(+) 0.755 3.528 2.663
   C00112  CDP C9H15N3O11P2 439.9659 .H/K-H(+) 0.638 4.159 2.654
   C01037  7,8-Diaminononanoate C9H20N2O2 206.1629 [+1]+OH(-) 0.747 3.510 2.623
   C05931  N2-Succinyl-L-glutamate C9H13NO7 381.9982 .H2PO4K-H(+) 0.713 3.645 2.600
   C00053  3'-Phosphoadenylyl sulfate C10H15N5O13P2S 641.9138 .H2PO4K-H(+) 0.691 3.698 2.554
   C00227  Acetyl phosphate C2H5O5P 312.8687 .HPO4K2-H(+) 0.659 3.860 2.544
   C04204  2,3-dihydroxybenzoylserine C10H11NO6 381.9982 .HPO4Na2-H(+) 0.663 3.645 2.418
   C14179  sulfoacetate C2H4O5S 312.8687 .HPO4K2-H(+) 0.622 3.860 2.399
   C00112  CDP C9H15N3O11P2 641.9138 .(H2PO4Na)2-H(+) 0.648 3.698 2.395
   C07838  D-Glycero-D-manno-heptose 1-phosphate C7H15O10P 522.9407 .(H2PO4)2KH-H(+) 0.616 3.779 2.329
   C01346  dUDP C9H14N2O11P2 522.9407 .H2PO4K-H(+) 0.609 3.779 2.301
   C00407  L-Isoleucine C6H13NO2 134.1071 [+2].H(+) 0.612 3.685 2.253
   C00044  GTP C10H16N5O14P3 559.9477 .H/K-H(+) 0.597 6.348 0.000
   C00330  Deoxyguanosine C10H13N5O4 439.9659 .HPO4K2-H(+) 0.595 4.159 0.000
   C00054  Adenosine 3',5'-bisphosphate C10H15N5O10P2 426.0236 -H(+) 0.591 4.506 0.000
   C05382  Sedoheptulose 7-phosphate C7H15O10P 522.9407 .(H2PO4)2KH-H(+) 0.584 3.779 0.000
   C00212  Adenosine C10H13N5O4 439.9659 .HPO4K2-H(+) 0.582 4.159 0.000
   C00227  Acetyl phosphate C2H5O5P 176.9355 .H/K-H(+) 0.556 4.342 0.000
   C00320  Thiosulfate H2O3S2 254.8785 .HPO4Na2-H(+) 0.552 3.483 0.000
   C05512  Deoxyinosine C10H12N4O4 522.9407 .(H2PO4K)2-H(+) 0.537 3.779 0.000
   C00979  O-Acetyl-L-serine C5H9NO4 129.0199 -NH3-H(+) 0.528 3.625 0.000
   C00015  UDP C9H14N2O12P2 522.9407 .H2PO4Na-H(+) 0.524 3.779 0.000
   C00047  L-Lysine C6H14N2O2 147.1045 [+2]-H(+) 0.516 3.518 0.000
   C04462  N-Succinyl-2-L-amino-6-oxoheptanedioate C11H15NO8 559.9477 .(H2PO4K)2-H(+) 0.515 6.348 0.000
   C04114  crotonobetaine C7H13NO2 381.9982 .(H2PO4Na)2-H(+) 0.514 3.645 0.000
   C00361  dGDP C10H15N5O10P2 426.0236 -H(+) 0.510 4.506 0.000
   C00148  L-Proline C5H9NO2 249.9880 .H2PO4K-H(+) 0.508 4.146 0.000
   C00286  dGTP C10H16N5O13P3 641.9138 .H2PO4K-H(+) 0.478 3.698 0.000
   C00074  Phosphoenolpyruvate C3H5O6P 184.9849 +OH(-) 0.476 4.899 0.000
   C00079  L-Phenylalanine C9H11NO2 186.0541 .H/Na-H(+) 0.472 4.743 0.000
   C00123  L-Leucine C6H13NO2 134.1071 [+2].H(+) 0.464 3.685 0.000
   C07836  D-Glycero-D-manno-heptose 7-phosphate C7H15O10P 522.9407 .(H2PO4)2KH-H(+) 0.451 3.779 0.000
   C00217  D-Glutamate C5H9NO4 129.0199 -NH3-H(+) 0.000 3.625 0.000
   C01134  Pantetheine 4'-phosphate C11H23N2O7PS 477.0539 .H2PO4Na-H(+) 0.000 4.719 0.000
   C11434  2-C-methyl-D-erythritol 4-phosphate C5H13O7P 252.9869 .H/K-H(+) 0.000 -4.401 -0.000
     KEGG pathway by CLR  
   Pathway_ion pvalue_ion qvalue_ion  Oxidative phosphorylation 6e-06 0.0005
  C5-Branched dibasic acid metabolism 9e-06 0.0004
  Microbial metabolism in diverse environments 1e-05 0.0003
  Aminobenzoate degradation 2e-05 0.0004
  Toluene degradation 3e-05 0.0005
  Methane metabolism 5e-05 0.0006
  Purine metabolism 0.0002 0.0027
  Glycolysis / Gluconeogenesis 0.0003 0.0031
  Sulfur metabolism 0.0009 0.0085
  Thiamine metabolism 0.002 0.0179
  Dioxin degradation 0.003 0.0196
  Bisphenol degradation 0.005 0.0285
  Glycerolipid metabolism 0.005 0.0264
  Chlorocyclohexane and chlorobenzene degradation 0.007 0.0395
  Glyoxylate and dicarboxylate metabolism 0.008 0.0410
  Pantothenate and CoA biosynthesis 0.008 0.0386
  Valine, leucine and isoleucine biosynthesis 0.01 0.0440
     COG enrichment  
   Pathway_MS pvalue_MS qvalue_MS  Inositol phosphate metabolism 0.002 0.1808
  Ribosome 0.002 0.1172
  Lipoic acid metabolism 0.005 0.1756
     Predicted metabolites from CLR  
   Predicted metabolites Pvalue Overlap with hits  D-Gluconate 0.0007 0.0000
  Reduced glutathione 0.01 0.0000
    
 
