# Supplementary material for: Genomewide landscape of gene–metabolome associations in Escherichia coli
Source: Mol Syst Biol. 2017 Jan 16;13(1):907. doi: 10.15252/msb.20167150 (PMC5293155; doi:10.15252/msb.20167150)
Supplement: Supplementary file 4 — Table EV3 [file MSB-13-907-s004.zip › details/data_ybdN.html]

 
 
 ybdN 
  ybdN - details 
 
 
  CLR  
   Gene_matching CLR_index  grxA 19.4
  uvrB 17.6
  ybjH 17.3
  purT 16.5
  djlB 14.6
  lipA 13.7
  rffE 13.1
  ycfD 11.5
  ybeA 11.5
  yaiI 10.3
  citB 10.0
  cusR 9.9
  rffM 9.6
  ybeT 8.5
  amiA 8.2
  yfcP 8.0
  renD 7.7
  ybfM 7.4
  ydbH 6.8
  csgB 6.5
  helD 6.4
  ylbA 6.4
  dgkA 5.7
  ybdD 5.7
  ygcW 5.6
  ygjV 5.4
  atoS 5.4
  trxC 5.4
  ycdX 5.3
  fdhD 5.3
  rlpA 5.2
  ybfQ 4.4
  yceI 4.3
  csgF 4.3
  ycdB 4.3
  gpp 4.1
  phoQ 4.1
  ydeU 4.1
  yebE 4.1
  ynjH 4.0
  ybdM 3.9
  ygdK 3.8
  yghA 3.8
  yicI 3.8
  yceA 3.7
  yiaU 3.7
  ydhT 3.7
  narX 3.7
  yiaA 3.7
  ycbC 3.6
  srmB 3.6
  sspB 3.5
  yqiK 3.5
  ybgL 3.5
  gadA 3.4
  pstB 3.4
  idnD 3.4
  lon 3.4
  clpP 3.3
  ybiS 3.3
  torR 3.3
  yecH 3.2
  glxK 3.2
  ychJ 3.1
  yoaE 3.1
  yebY 3.1
  yqjA 3.1
  oxyR 3.1
  fur 3.0
  aslA 3.0
  ydfT 3.0
     Differential ions  
   id name formula mz mod AUC Z-score Z-score AUC Weighted   C02730  o-Succinylbenzoate C11H10O5 239.0593 +OH(-) 0.858 11.966 10.267
   C00078  L-Tryptophan C11H12N2O2 239.0593 +Cl(-) 0.625 11.966 7.482
   C02107  D-tartrate C4H6O6 149.0097 -H(+) 0.680 9.568 6.503
   C00921  Dihydropteroate C14H14N6O3 313.1029 -H(+) 0.731 8.865 6.480
   C00898  L-tartrate C4H6O6 149.0097 -H(+) 0.642 9.568 6.140
   C04204  2,3-dihydroxybenzoylserine C10H11NO6 241.0548 [+1]-H(+) 0.799 7.435 5.937
   C04204  2,3-dihydroxybenzoylserine C10H11NO6 240.0508 -H(+) 0.908 6.346 5.764
   C11457  3-(3-hydroxy-phenyl)propionate C9H10O3 165.0541 -H(+) 0.916 6.136 5.623
   C02995  Maltose 6'-phosphate C12H23O14P 594.9634 .HPO4K2-H(+) 0.666 8.071 5.375
   C02591  Sucrose 6-phosphate C12H23O14P 594.9634 .HPO4K2-H(+) 0.638 8.071 5.153
   C00942  3',5'-Cyclic GMP C10H12N5O7P 345.0429 [+1]-H(+) 0.819 5.907 4.837
   C00885  Isochorismate C10H10O6 225.0304 -H(+) 0.717 6.692 4.797
   C00942  3',5'-Cyclic GMP C10H12N5O7P 344.0409 -H(+) 0.734 6.215 4.564
   C18239  cyclic pyranopterin monophosphate C10H14N5O8P 344.0409 -H2O-H(+) 0.722 6.215 4.490
   C00254  Prephenate C10H10O6 225.0304 -H(+) 0.655 6.692 4.384
   C00864  (R)-Pantothenate C9H17NO5 240.0827 .H/Na-H(+) 0.762 5.698 4.344
   C00423  trans-Cinnamate C9H8O2 165.0541 +OH(-) 0.669 6.136 4.107
   C00522  (R)-Pantoate C6H12O4 165.0725 +OH(-) 0.844 4.660 3.934
   cis-3-(3-carboxyethyl)-3,5-cyclohexadiene-1,2-diol  cis-3-(3-carboxyethyl)-3,5-cyclohexadiene-1,2-diol C9H12O4 165.0541 -H2O-H(+) 0.640 6.136 3.929
   C00079  L-Phenylalanine C9H11NO2 165.0725 [+1]-H(+) 0.770 4.660 3.589
   C00680  meso-2,6-Diaminoheptanedioate C7H14N2O4 325.0264 .H2PO4K-H(+) 0.883 3.715 3.281
   C00575  cAMP C10H12N5O6P 328.0454 -H(+) 0.773 4.145 3.203
   C05931  N2-Succinyl-L-glutamate C9H13NO7 366.0212 .H2PO4Na-H(+) 0.663 4.789 3.174
   C00575  cAMP C10H12N5O6P 329.0479 [+1]-H(+) 0.802 3.918 3.143
   C04778  N1-(5-Phospho-alpha-D-ribosyl)-5,6-dimethylbenzimidazole C14H19N2O7P 477.0539 .H2PO4Na-H(+) 0.735 4.204 3.092
   C00296  Quinate C7H12O6 430.9687 .(H2PO4Na)2-H(+) 0.875 3.515 3.074
   C00055  CMP C9H14N3O8P 242.0786 -HPO3-H(+) 0.639 4.011 2.561
   C00079  L-Phenylalanine C9H11NO2 166.0780 [+2]-H(+) 0.663 3.737 2.476
   C00105  UMP C9H13N2O9P 305.0201 -H2O-H(+) 0.614 4.026 2.473
   C00522  (R)-Pantoate C6H12O4 166.0780 [+1]+OH(-) 0.644 3.737 2.406
   C00387  Guanosine C10H13N5O5 500.0247 .(H2PO4)2NaH-H(+) 0.616 3.768 2.321
   C07838  D-Glycero-D-manno-heptose 1-phosphate C7H15O10P 430.9687 .HPO4Na2-H(+) 0.604 3.515 2.125
   C04479  2-Hydroxy-6-oxonona-2,4-diene-1,9-dioate C9H10O6 430.9687 .(H2PO4)2NaH-H(+) 0.600 3.515 2.110
   C06423  octanoate (n-C8:0) C8H16O2 165.0896 .H/Na-H(+) 0.594 5.059 0.000
   C00144  GMP C10H14N5O8P 344.0409 -H2O-H(+) 0.593 6.215 0.000
   C01118  O-Succinyl-L-homoserine C8H13NO6 240.0508 .H/Na-H(+) 0.587 6.346 0.000
   C00116  Glycerol C3H8O3 73.0303 -H2O-H(+) 0.584 -4.657 -0.000
   C00062  L-Arginine C6H14N4O2 444.9690 .(H2PO4K)2-H(+) 0.575 3.599 0.000
   C00957  Mercaptopyruvate C3H4O3S 140.9655 .H/Na-H(+) 0.574 -4.263 -0.000
   C03393  4-Phospho-D-erythronate C4H9O8P 117.0195 -H3PO4-H(+) 0.566 -3.458 -0.000
   C03451  (R)-S-Lactoylglutathione C13H21N3O8S 498.0495 .H2PO4Na-H(+) 0.558 4.154 0.000
   C00475  Cytidine C9H13N3O5 242.0786 -H(+) 0.555 4.011 0.000
   C00047  L-Lysine C6H14N2O2 165.1174 [+2]+OH(-) 0.546 4.366 0.000
   C05235  Acetol C3H6O2 73.0303 -H(+) 0.542 -4.657 -0.000
   C01181  gamma-butyrobetaine C7H15NO2 166.0780 .H/Na-H(+) 0.536 3.737 0.000
   C00689  alpha,alpha'-Trehalose 6-phosphate C12H23O14P 594.9634 .HPO4K2-H(+) 0.532 8.071 0.000
   C00262  Hypoxanthine C5H4N4O 117.0195 -H2O-H(+) 0.530 -3.458 -0.000
   C00134  Putrescine C4H12N2 223.0285 .H2PO4K-H(+) 0.529 12.399 0.000
   C00424  L-Lactaldehyde C3H6O2 73.0303 -H(+) 0.525 -4.657 -0.000
   C00942  3',5'-Cyclic GMP C10H12N5O7P 366.0212 .H/Na-H(+) 0.523 4.789 0.000
   C00036  Oxaloacetate C4H4O5 149.0097 +OH(-) 0.520 9.568 0.000
   C07836  D-Glycero-D-manno-heptose 7-phosphate C7H15O10P 430.9687 .HPO4Na2-H(+) 0.520 3.515 0.000
   C00147  Adenine C5H5N5 117.0195 -NH3-H(+) 0.517 -3.458 -0.000
   C06007  (R)-2,3-Dihydroxy-3-methylpentanoate C6H12O4 166.0780 [+1]+OH(-) 0.506 3.737 0.000
   C00251  chorismate C10H10O6 225.0304 -H(+) 0.490 6.692 0.000
   C00362  dGMP C10H14N5O7P 328.0454 -H2O-H(+) 0.489 4.145 0.000
   C00445  5,10-Methenyltetrahydrofolate C20H22N7O6 673.0845 .(H2PO4)2NaH-H(+) 0.481 4.218 0.000
   C01936  Maltohexaose C36H62O31 989.3097 -H(+) 0.476 4.250 0.000
   C00718  1,4-alpha-D-glucan C36H62O31 989.3097 -H(+) 0.462 4.250 0.000
   C00647  Pyridoxamine 5'-phosphate C8H13N2O5P 480.9661 .(H2PO4)2KH-H(+) 0.441 3.596 0.000
   C05382  Sedoheptulose 7-phosphate C7H15O10P 430.9687 .HPO4Na2-H(+) 0.411 3.515 0.000
   C05817  2-Succinyl-6-hydroxy-2,4-cyclohexadiene-1-carboxylate C11H12O6 239.0593 -H(+) 0.409 11.966 0.000
   C06007  (R)-2,3-Dihydroxy-3-methylpentanoate C6H12O4 165.0725 +OH(-) 0.401 4.660 0.000
   C01571  Decanoate (n-C10:0) C10H20O2 313.0786 .HPO4Na2-H(+) 0.371 15.616 0.000
   C00666  LL-2,6-Diaminoheptanedioate C7H14N2O4 325.0264 .H2PO4K-H(+) 0.000 3.715 0.000
   C00931  Porphobilinogen C10H14N2O4 345.0429 .H2PO4Na-H(+) 0.000 5.907 0.000
   C01134  Pantetheine 4'-phosphate C11H23N2O7PS 477.0539 .H2PO4Na-H(+) 0.000 4.204 0.000
   C03794  N6-(1,2-Dicarboxyethyl)-AMP C14H18N5O11P 500.0247 .H/K-H(+) 0.000 3.768 0.000
   C05809  3-Octaprenyl-4-hydroxybenzoate C47H70O3 719.4862 .H/K-H(+) 0.000 3.632 0.000
   C00416  1,2-didodecanoyl-sn-glycerol 3-phosphate C27H53O8P1 557.3196 .H/Na-H(+) 0.605 -3.492 -2.113
   C00042  Succinate C4H6O4 117.0195 -H(+) 0.638 -3.458 -2.207
   C06056  4-Hydroxy-L-threonine C4H9NO4 117.0195 -NH3-H(+) 0.663 -3.458 -2.294
   C03393  4-Phospho-D-erythronate C4H9O8P 236.9743 .H/Na-H(+) 0.600 -4.205 -2.524
   C00042  Succinate C4H6O4 139.0024 .H/Na-H(+) 0.674 -3.778 -2.548
   C00163  Propionate (n-C3:0) C3H6O2 73.0303 -H(+) 0.601 -4.657 -2.800
   C00042  Succinate C4H6O4 236.9743 .H2PO4Na-H(+) 0.670 -4.205 -2.816
   C00042  Succinate C4H6O4 73.0303 -CO2-H(+) 0.716 -4.657 -3.334
   C00937  D-Lactaldehyde C3H6O2 73.0303 -H(+) 0.827 -4.657 -3.853
     KEGG pathway by CLR  
   Pathway_ion pvalue_ion qvalue_ion  Microbial metabolism in diverse environments 4e-05 0.0029
  Chlorocyclohexane and chlorobenzene degradation 0.0002 0.0076
  Pyruvate metabolism 0.0002 0.0051
  Glyoxylate and dicarboxylate metabolism 0.0002 0.0047
  Propanoate metabolism 0.0004 0.0066
  Phenylalanine metabolism 0.0007 0.0087
  Purine metabolism 0.0007 0.0083
  Pyrimidine metabolism 0.001 0.0134
  Ascorbate and aldarate metabolism 0.002 0.0135
  Glycerolipid metabolism 0.002 0.0123
  Biotin metabolism 0.002 0.0176
  Pentose and glucuronate interconversions 0.004 0.0233
  Oxidative phosphorylation 0.004 0.0221
  Folate biosynthesis 0.004 0.0205
     COG enrichment  
   Pathway_MS pvalue_MS qvalue_MS  Sphingolipid metabolism 0.0004 0.0349
  Glycerolipid metabolism 0.001 0.0475
  Lipoic acid metabolism 0.001 0.0345
  Taurine and hypotaurine metabolism 0.005 0.1247
  Nucleotide excision repair 0.007 0.1379
  Two-component system 0.009 0.1498
     Predicted metabolites from CLR  
   Predicted metabolites Pvalue Overlap with hits  UDP 0.0002 0.0000
  dADP 0.0003 0.0000
  dGDP 0.0003 0.0000
  dUDP 0.0003 0.0000
  3'-Phosphoadenylyl sulfate 0.0003 0.0000
  dCDP 0.0005 0.0000
  Adenosine 3',5'-bisphosphate 0.0005 0.0000
  CDP 0.0007 0.0000
  Sulfite 0.003 0.0000
  GDP 0.003 0.0000
  L-Methionine 0.003 0.0000
    
 
