# Supplementary material for: Genomewide landscape of gene–metabolome associations in Escherichia coli
Source: Mol Syst Biol. 2017 Jan 16;13(1):907. doi: 10.15252/msb.20167150 (PMC5293155; doi:10.15252/msb.20167150)
Supplement: Supplementary file 4 — Table EV3 [file MSB-13-907-s004.zip › details/data_ybdO.html]

 
 
 ybdO 
  ybdO - details 
 
 
  CLR  
   Gene_matching CLR_index  cyoD 17.4
  zntR 16.4
  yafC 16.0
  argR 14.4
  leuO 13.5
  narX 12.8
  ihfA 12.7
  clpA 12.4
  fnr 12.1
  tdcA 11.3
  rbsR 10.8
  envR 10.6
  wza 10.4
  uidR 10.2
  mipA 9.9
  yobD 9.8
  fdrA 9.7
  yebG 9.5
  potH 9.4
  ydbH 9.0
  citB 8.8
  sdhD 8.6
  yohK 8.2
  yiaT 7.8
  clpP 7.8
  trpR 7.8
  perR 7.8
  cbl 7.7
  cyoB 7.7
  ybeF 7.5
  yfcP 7.5
  yaiI 7.3
  cyoA 7.2
  helD 7.1
  btuR 7.0
  hrpA 6.9
  yeiE 6.8
  cyoC 6.7
  dacC 6.6
  slp 6.5
  fucR 6.4
  ebgR 6.4
  ompA 6.4
  djlB 6.2
  nanR 6.1
  prpC 6.0
  uhpB 6.0
  phoP 6.0
  yeaK 6.0
  dacA 5.5
  hupB 5.4
  cpxR 5.3
  soxS 5.3
  phnF 5.3
  yebZ 5.3
  chbR 5.3
  galR 5.2
  ynjH 5.2
  hofQ 5.0
  sucA 5.0
  ygjV 5.0
  lrp 5.0
  cusC 4.9
  mltA 4.9
  yehM 4.9
  ygcW 4.9
  soxR 4.8
  ybeA 4.8
  wcaA 4.8
  bglG 4.7
  yeiJ 4.7
  tsx 4.7
  ypdI 4.7
  hepA 4.6
  ypfI 4.6
  cpxA 4.6
  fimD 4.6
  pnuC 4.6
  lhr 4.5
  yahE 4.5
  envY 4.5
  ydhR 4.5
  yebY 4.5
  yadL 4.4
  clpX 4.4
  yjiE 4.4
  rlpA 4.4
  ypjB 4.3
  ybjH 4.3
  yaiS 4.3
  rhaR 4.2
  ybgS 4.2
  ymcB 4.1
  yfeX 4.1
  yfdK 4.1
  uxuR 4.1
  cusA 4.1
  tfaR 4.1
  yciN 4.0
  csgF 4.0
  bcsE 4.0
  poxB 4.0
  ynfL 3.9
  yfbM 3.9
  gapC 3.9
  yafQ 3.9
  deoR 3.9
  cirA 3.9
  treR 3.8
  zraR 3.8
  ompF 3.8
  yphA 3.7
  ydiV 3.7
  yccJ 3.7
  ygbE 3.7
  ybiU 3.7
  ydgD 3.7
  ycdS 3.6
  frdA 3.6
  metR 3.5
  yidZ 3.5
  mdtC 3.5
  fdhD 3.5
  ycaJ 3.5
  ssuE 3.5
  tyrR 3.4
  fadR 3.4
  fimB 3.4
  mhpR 3.3
  gpp 3.3
  ydhD 3.3
  cadC 3.3
  rpiB 3.2
  yhaH 3.2
  yfcT 3.2
  sdhC 3.2
  moaA 3.2
  yafT 3.1
  yehK 3.1
  renD 3.1
  pepN 3.1
  yegE 3.1
  yniB 3.1
  frdD 3.1
  cusR 3.1
  nhaR 3.1
  hha 3.0
  ygaC 3.0
  ybfM 3.0
  ydaY 3.0
  ybbO 3.0
     Differential ions  
   id name formula mz mod AUC Z-score Z-score AUC Weighted   C01092  8-Amino-7-oxononanoate C9H17NO3 210.1094 .H/Na.H(+) 0.853 5.586 4.765
   cyclopropane phosphatidylethanolamine (dihexadec-9,10-cyclo-anoyl, n-C16:0 cyclo)  cyclopropane phosphatidylethanolamine (dihexadec-9,10-cyclo-anoyl, n-C16:0 cyclo) C39H74N1O8P1 718.5338 [+2].H(+) 0.925 4.951 4.581
   cyclopropane phosphatidylethanolamine (dihexadec-9,10-cyclo-anoyl, n-C16:0 cyclo)  cyclopropane phosphatidylethanolamine (dihexadec-9,10-cyclo-anoyl, n-C16:0 cyclo) C39H74N1O8P1 716.5212 .H(+) 0.951 4.714 4.482
   C00310  D-Xylulose C5H10O5 369.0017 .(H2PO4)2NaH.H(+) 0.892 4.499 4.012
   C00350  phosphatidylethanolamine (dioctadec-11-enoyl, n-C18:1) C41H78N1O8P1 744.5543 .H(+) 0.798 4.704 3.753
   C04778  N1-(5-Phospho-alpha-D-ribosyl)-5,6-dimethylbenzimidazole C14H19N2O7P 477.0539 .H2PO4Na-H(+) 0.735 4.388 3.227
   C00350  phosphatidylethanolamine (dihexadecanoyl, n-C16:0) C37H74N1O8P1 692.5148 .H(+) 0.785 4.077 3.201
   C05730  Glutathionylspermidine C17H34N6O5S 435.2396 .H(+) 0.782 4.093 3.199
   cyclopropane phosphatidylethanolamine (dihexadec-9,10-cyclo-anoyl, n-C16:0 cyclo)  cyclopropane phosphatidylethanolamine (dihexadec-9,10-cyclo-anoyl, n-C16:0 cyclo) C39H74N1O8P1 738.5036 .Na(+) 0.836 3.719 3.108
   cyclopropane phosphatidylethanolamine (dihexadec-9,10-cyclo-anoyl, n-C16:0 cyclo)  cyclopropane phosphatidylethanolamine (dihexadec-9,10-cyclo-anoyl, n-C16:0 cyclo) C39H74N1O8P1 738.5036 .H/Na.H(+) 0.836 3.719 3.108
   C00575  cAMP C10H12N5O6P 329.0479 [+1]-H(+) 0.802 3.829 3.072
   C03539  S-Ribosyl-L-homocysteine C9H17NO6S 439.9659 .HPO4K2-H(+) 0.769 3.985 3.066
   C05925  Dihydroneopterin monophosphate C9H14N5O7P 567.9630 .(H2PO4)2KH-H(+) 0.760 3.938 2.993
   C00112  CDP C9H15N3O11P2 423.9899 .H/Na-H(+) 0.614 4.838 2.971
   C00942  3',5'-Cyclic GMP C10H12N5O7P 345.0429 [+1]-H(+) 0.819 3.611 2.957
   C00575  cAMP C10H12N5O6P 328.0454 -H(+) 0.773 3.821 2.952
   C00979  O-Acetyl-L-serine C5H9NO4 104.0705 -CO2.H(+) 0.644 4.564 2.938
   C04494  Guanosine 3'-diphosphate 5'-triphosphate C10H18N5O20P5 681.9045 -H(+) 0.845 3.472 2.935
   C00254  Prephenate C10H10O6 369.0017 .HPO4Na2.H(+) 0.642 4.499 2.887
   C04494  Guanosine 3'-diphosphate 5'-triphosphate C10H18N5O20P5 703.8900 .H/Na-H(+) 0.790 3.595 2.842
   C05382  Sedoheptulose 7-phosphate C7H15O10P 424.9738 .H2PO4K-H(+) 0.675 4.192 2.831
   C00885  Isochorismate C10H10O6 369.0017 .HPO4Na2.H(+) 0.626 4.499 2.815
   C05931  N2-Succinyl-L-glutamate C9H13NO7 366.0212 .H2PO4Na-H(+) 0.663 4.228 2.802
   C07836  D-Glycero-D-manno-heptose 7-phosphate C7H15O10P 424.9738 .H2PO4K-H(+) 0.667 4.192 2.796
   octadecenoate (n-C18:1)  octadecenoate (n-C18:1) C18H34O2 403.2288 .H2PO4Na.H(+) 0.702 3.956 2.777
   C00942  3',5'-Cyclic GMP C10H12N5O7P 344.0409 -H(+) 0.734 3.681 2.703
   C05932  N2-Succinyl-L-glutamate 5-semialdehyde C9H13NO6 350.0314 .H2PO4Na-H(+) 0.654 4.107 2.687
   C00105  UMP C9H13N2O9P 305.0201 -H2O-H(+) 0.614 4.330 2.660
   C18239  cyclic pyranopterin monophosphate C10H14N5O8P 344.0409 -H2O-H(+) 0.722 3.681 2.660
   C00054  Adenosine 3',5'-bisphosphate C10H15N5O10P2 567.9630 .HPO4Na2-H(+) 0.666 3.938 2.624
   C00224  Adenosine 5'-phosphosulfate C10H14N5O10PS 567.9630 .HPO4Na2-H(+) 0.655 3.938 2.580
   C00112  CDP C9H15N3O11P2 439.9659 .H/K-H(+) 0.638 3.985 2.543
   C00119  5-Phospho-alpha-D-ribose 1-diphosphate C5H13O14P3 526.9023 .H2PO4K.H(+) 0.663 3.589 2.379
   C00206  dADP C10H15N5O9P2 681.9045 .(H2PO4K)2-H(+) 0.684 3.472 2.375
   C00575  cAMP C10H12N5O6P 448.0070 .H2PO4Na-H(+) 0.654 3.600 2.355
   C05198  5'-Deoxyadenosine C10H13N5O3 521.9619 .(H2PO4K)2-H(+) 0.601 3.798 2.284
   C00112  CDP C9H15N3O11P2 543.9430 .HPO4Na2-H(+) 0.599 4.031 0.000
   C00330  Deoxyguanosine C10H13N5O4 439.9659 .HPO4K2-H(+) 0.595 3.985 0.000
   C00144  GMP C10H14N5O8P 344.0409 -H2O-H(+) 0.593 3.681 0.000
   C00054  Adenosine 3',5'-bisphosphate C10H15N5O10P2 426.0236 -H(+) 0.591 5.002 0.000
   C00112  CDP C9H15N3O11P2 402.0111 -H(+) 0.584 4.845 0.000
   C00015  UDP C9H14N2O12P2 424.9738 .H/Na-H(+) 0.582 4.192 0.000
   C00212  Adenosine C10H13N5O4 439.9659 .HPO4K2-H(+) 0.582 3.985 0.000
   C00575  cAMP C10H12N5O6P 567.9630 .(H2PO4Na)2-H(+) 0.578 3.938 0.000
   C00350  phosphatidylethanolamine (dioctadec-11-enoyl, n-C18:1) C41H78N1O8P1 766.5372 .H/Na.H(+) 0.571 3.468 0.000
   C00350  phosphatidylethanolamine (dioctadec-11-enoyl, n-C18:1) C41H78N1O8P1 766.5372 .Na(+) 0.571 3.468 0.000
   C00054  Adenosine 3',5'-bisphosphate C10H15N5O10P2 448.0070 .H/Na-H(+) 0.567 3.600 0.000
   C00350  phosphatidylethanolamine (dihexadec-9enoyl, n-C16:1) C37H70N1O8P1 688.4884 .H(+) 0.566 4.396 0.000
   C01079  Protoporphyrinogen IX C34H40N4O4 607.2643 .H/K.H(+) 0.566 5.242 0.000
   C00042  Succinate C4H6O4 141.0162 .Na(+) 0.562 4.573 0.000
   C00042  Succinate C4H6O4 141.0162 .H/Na.H(+) 0.562 4.573 0.000
   C00559  Deoxyadenosine C10H13N5O3 521.9619 .(H2PO4K)2-H(+) 0.561 3.798 0.000
   C00312  L-Xylulose C5H10O5 369.0017 .(H2PO4)2NaH.H(+) 0.561 4.499 0.000
   C00334  4-Aminobutanoate C4H9NO2 104.0705 .H(+) 0.557 4.564 0.000
   C00112  CDP C9H15N3O11P2 521.9619 .H2PO4Na-H(+) 0.554 3.798 0.000
   C05512  Deoxyinosine C10H12N4O4 424.9738 .HPO4K2-H(+) 0.547 4.192 0.000
   C00251  chorismate C10H10O6 369.0017 .HPO4Na2.H(+) 0.546 4.499 0.000
   C00134  Putrescine C4H12N2 90.1102 [+1].H(+) 0.545 4.842 0.000
   C00361  dGDP C10H15N5O10P2 567.9630 .HPO4Na2-H(+) 0.534 3.938 0.000
   C00705  dCDP C9H15N3O10P2 521.9619 .H2PO4K-H(+) 0.534 3.798 0.000
   C00286  dGTP C10H16N5O13P3 543.9430 .H/K-H(+) 0.530 4.031 0.000
   C00053  3'-Phosphoadenylyl sulfate C10H15N5O13P2S 543.9430 .H/K-H(+) 0.526 4.031 0.000
   C00121  D-Ribose C5H10O5 369.0017 .(H2PO4)2NaH.H(+) 0.524 4.499 0.000
   C00942  3',5'-Cyclic GMP C10H12N5O7P 366.0212 .H/Na-H(+) 0.523 4.228 0.000
   C00055  CMP C9H14N3O8P 304.0335 -H2O-H(+) 0.520 4.679 0.000
   C00015  UDP C9H14N2O12P2 402.9931 -H(+) 0.517 4.038 0.000
   C07838  D-Glycero-D-manno-heptose 1-phosphate C7H15O10P 424.9738 .H2PO4K-H(+) 0.515 4.192 0.000
   C00361  dGDP C10H15N5O10P2 426.0236 -H(+) 0.510 5.002 0.000
   C00181  D-Xylose C5H10O5 369.0017 .(H2PO4)2NaH.H(+) 0.506 4.499 0.000
   C00101  5,6,7,8-Tetrahydrofolate C19H23N7O6 662.0977 .(H2PO4)2NaH-H(+) 0.505 3.963 0.000
   C00259  L-Arabinose C5H10O5 369.0017 .(H2PO4)2NaH.H(+) 0.501 4.499 0.000
   C01508  L-Lyxose C5H10O5 369.0017 .(H2PO4)2NaH.H(+) 0.498 4.499 0.000
   C05198  5'-Deoxyadenosine C10H13N5O3 423.9899 .HPO4K2-H(+) 0.494 4.838 0.000
   C00362  dGMP C10H14N5O7P 328.0454 -H2O-H(+) 0.489 3.821 0.000
   C00445  5,10-Methenyltetrahydrofolate C20H22N7O6 673.0845 .(H2PO4)2NaH-H(+) 0.481 6.654 0.000
   C00361  dGDP C10H15N5O10P2 448.0070 .H/Na-H(+) 0.474 3.600 0.000
   C11145  methanesulfonate CH4O3S 76.9706 -H2O-H(+) 0.469 -4.271 -0.000
   cyclopropane phosphatidylethanolamine (dihexadec-9,10-cyclo-anoyl, n-C16:0 cyclo)  cyclopropane phosphatidylethanolamine (dihexadec-9,10-cyclo-anoyl, n-C16:0 cyclo) C39H74N1O8P1 717.5246 [+1].H(+) 0.456 3.538 0.000
   C00559  Deoxyadenosine C10H13N5O3 423.9899 .HPO4K2-H(+) 0.450 4.838 0.000
   C00575  cAMP C10H12N5O6P 350.0314 .H/Na-H(+) 0.409 4.107 0.000
   C00931  Porphobilinogen C10H14N2O4 345.0429 .H2PO4Na-H(+) 0.000 3.611 0.000
   C01134  Pantetheine 4'-phosphate C11H23N2O7PS 477.0539 .H2PO4Na-H(+) 0.000 4.388 0.000
   (2R,4S)-2-methyl-2,3,3,4-tetrahydroxytetrahydrofuran  (2R,4S)-2-methyl-2,3,3,4-tetrahydroxytetrahydrofuran C5H10O5 369.0017 .(H2PO4)2NaH.H(+) 0.000 4.499 0.000
   C00217  D-Glutamate C5H9NO4 104.0705 -CO2.H(+) 0.000 4.564 0.000
   C00508  L-Ribulose C5H10O5 369.0017 .(H2PO4)2NaH.H(+) 0.000 4.499 0.000
   C05809  3-Octaprenyl-4-hydroxybenzoate C47H70O3 705.5253 .H/Na.H(+) 0.000 8.235 0.000
     KEGG pathway by CLR  
   Pathway_ion pvalue_ion qvalue_ion  Purine metabolism 3e-08 0.0000
  Pyrimidine metabolism 4e-07 0.0000
  Oxidative phosphorylation 8e-05 0.0016
  D-Glutamine and D-glutamate metabolism 8e-05 0.0013
  Alanine, aspartate and glutamate metabolism 0.0001 0.0017
  Sulfur metabolism 0.0001 0.0016
  Chlorocyclohexane and chlorobenzene degradation 0.0002 0.0020
  Arginine and proline metabolism 0.0004 0.0035
  Fatty acid metabolism 0.0008 0.0061
  Sulfur relay system 0.0008 0.0056
  Glycerophospholipid metabolism 0.0008 0.0054
  Butanoate metabolism 0.0009 0.0052
  Valine, leucine and isoleucine degradation 0.001 0.0075
  Bisphenol degradation 0.002 0.0086
  Glutathione metabolism 0.002 0.0098
  C5-Branched dibasic acid metabolism 0.005 0.0207
  Nitrogen metabolism 0.005 0.0229
  Propanoate metabolism 0.006 0.0242
     COG enrichment  
   Pathway_MS pvalue_MS qvalue_MS  Oxidative phosphorylation 2e-06 0.0002
  Toluene degradation 1e-05 0.0005
  Two-component system 0.0001 0.0035
  Citrate cycle (TCA cycle) 0.0004 0.0104
  Peptidoglycan biosynthesis 0.008 0.1580
  Riboflavin metabolism 0.009 0.1468
     Predicted metabolites from CLR  
   Predicted metabolites Pvalue Overlap with hits  silver 9e-06 0.0000
  Fumarate 2e-05 0.0000
  Cu+ 2e-05 0.0000
  Succinate 0.0002 1.0000
    
 
