# Supplementary material for: Genomewide landscape of gene–metabolome associations in Escherichia coli
Source: Mol Syst Biol. 2017 Jan 16;13(1):907. doi: 10.15252/msb.20167150 (PMC5293155; doi:10.15252/msb.20167150)
Supplement: Supplementary file 4 — Table EV3 [file MSB-13-907-s004.zip › details/data_ybdR.html]

 
 
 ybdR 
  ybdR - details 
 
 
  CLR  
   Gene_matching CLR_index  holD 13.2
  yjeK 12.0
  cysH 10.3
  garD 9.2
  glmM 8.4
  aslB 8.3
  yhaI 8.3
  fdoI 8.2
  codB 8.2
  yjdF 7.7
  pdxJ 7.6
  yccC 7.6
  metH 7.4
  fdoG 7.2
  metE 7.1
  nrdF 7.0
  aroH 7.0
  uidA 6.9
  metL 6.8
  nupC 6.6
  ccmH 6.6
  yggD 6.6
  ygeR 6.5
  spr 6.5
  ulaB 6.4
  gspO 6.4
  rnb 6.3
  ilvA 6.3
  ilvB 6.3
  yqjD 6.3
  flgK 6.2
  yjfJ 6.2
  yahC 6.2
  coaE 6.1
  panC 6.1
  ybeQ 6.0
  yjeO 6.0
  yfgC 5.8
  ksgA 5.8
  pdxA 5.7
  ptsG 5.7
  gadX 5.7
  ulaC 5.7
  betT 5.7
  ygcN 5.6
  sfmC 5.6
  ycjP 5.6
  yagP 5.6
  ygjN 5.5
  yhbS 5.5
  vsr 5.4
  yafU 5.4
  fadD 5.3
  yfcV 5.3
  yaaU 5.3
  ymcA 5.3
  ccmF 5.2
  yhhT 5.2
  ygjQ 5.2
  yraP 5.1
  ybiT 5.1
  uspE 5.1
  yjhG 5.1
  yggF 5.1
  yjfY 5.1
  aroP 5.0
  ydjG 5.0
  rsgA 5.0
  narZ 5.0
  yghW 4.9
  osmC 4.9
  flgC 4.9
  cof 4.9
  ygcL 4.9
  yqjG 4.9
  ydiZ 4.9
  yeiQ 4.9
  gshA 4.9
  nudH 4.8
  yjeP 4.8
  fhuC 4.8
  sufA 4.8
  yjfK 4.8
  ycaL 4.7
  yggV 4.7
  araG 4.6
  treC 4.6
  xylE 4.6
  xapB 4.6
  ppx 4.6
  feaR 4.5
  rbsD 4.5
  nagD 4.5
  rep 4.5
  puuD 4.4
  tolQ 4.4
  fhuD 4.4
  etp 4.4
  yohL 4.4
  yjcZ 4.4
  ydcW 4.3
  yedK 4.3
  ysgA 4.3
  yqeB 4.3
  potA 4.3
  yeaB 4.3
  mdtK 4.2
  yceD 4.2
  ptsP 4.2
  ilvE 4.2
  ycfH 4.2
  yccF 4.1
  yjeH 4.1
  fhuF 4.1
  ruvB 4.1
  ybcN 4.1
  mutL 4.1
  pgm 4.1
  ynfG 4.0
  hofB 4.0
  yjiO 4.0
  hsdM 3.9
  ytfG 3.9
  ydhF 3.9
  hycG 3.9
  ydhC 3.9
  ymgA 3.8
  ybgA 3.8
  evgS 3.8
  fruK 3.8
  ygjJ 3.8
  yhbW 3.8
  ygeA 3.8
  ycdQ 3.8
  frlR 3.8
  yliI 3.7
  aroG 3.7
  yhbE 3.7
  acrD 3.7
  rpoN 3.7
  yqiJ 3.7
  ychF 3.7
  nanA 3.7
  cchA 3.7
  ygiE 3.7
  cpxP 3.7
  ybdZ 3.7
  rlmB 3.7
  yieI 3.6
  ygeX 3.6
  yedP 3.6
  lysS 3.6
  yraI 3.6
  proV 3.6
  rluC 3.6
  kbl 3.6
  murP 3.6
  tiaE 3.5
  sbmC 3.5
  fixB 3.5
  yaiO 3.4
  hslJ 3.4
  mdlA 3.4
  flhB 3.4
  ydhJ 3.4
  rsuA 3.4
  srlB 3.4
  rhtA 3.4
  frc 3.4
  agaD 3.4
  ybaV 3.4
  artP 3.4
  slt 3.4
  glnP 3.3
  tpx 3.3
  dnaQ 3.3
  cysN 3.3
  ydaS 3.3
  sodA 3.3
  nrdH 3.3
  gcl 3.3
  yraK 3.3
  tam 3.3
  yqeG 3.3
  malG 3.2
  amiA 3.2
  yahK 3.2
  yehA 3.2
  cynX 3.2
  allD 3.2
  yccZ 3.2
  cysJ 3.2
  secB 3.2
  ykfA 3.2
  putP 3.2
  yhhW 3.2
  argH 3.2
  narW 3.2
  yeiM 3.2
  chbC 3.2
  nudB 3.1
  rffC 3.1
  apaH 3.1
  ydjL 3.1
  deoC 3.1
  cysC 3.1
  yfcO 3.1
  yzgL 3.1
  yiaK 3.1
  galF 3.1
  yhgA 3.1
  asnA 3.0
  metB 3.0
  purC 3.0
  rng 3.0
  lysC 3.0
  fldB 3.0
  yjbH 3.0
  mcrB 3.0
  fliG 3.0
  fcl 3.0
  gmd 3.0
  crcB 3.0
  rfaY 3.0
  fliC 3.0
  agaS 3.0
     Differential ions  
   id name formula mz mod AUC Z-score Z-score AUC Weighted   C01755  Thiocyanate CHNS 79.9585 .H/Na-H(+) 0.761 3.499 2.662
     KEGG pathway by CLR  
   Pathway_ion pvalue_ion qvalue_ion  Cysteine and methionine metabolism 0 0.0000
     COG enrichment  
   Pathway_MS pvalue_MS qvalue_MS  Chlorocyclohexane and chlorobenzene degradation 0 0.0000
  Fluorobenzoate degradation 0 0.0000
  Sulfur metabolism 5e-05 0.0013
  Amino sugar and nucleotide sugar metabolism 0.0004 0.0083
  Purine metabolism 0.0006 0.0097
  Phosphotransferase system (PTS) 0.0008 0.0098
  Ascorbate and aldarate metabolism 0.0009 0.0101
  Selenoamino acid metabolism 0.002 0.0175
  Pantothenate and CoA biosynthesis 0.002 0.0209
  Cysteine and methionine metabolism 0.004 0.0329
  Vitamin B6 metabolism 0.009 0.0649
  Mismatch repair 0.01 0.0634
     Predicted metabolites from CLR  
   Predicted metabolites Pvalue Overlap with hits  GDP-4-dehydro-6-deoxy-D-mannose 0 0.0000
  ferroxamine 0.0002 0.0000
  4-Phospho-L-aspartate 0.0003 0.0000
  5-Methyltetrahydrofolate 0.0003 0.0000
  Adenosine 5'-phosphosulfate 0.0003 0.0000
  Aerobactin 0.0003 0.0000
  coprogen 0.0003 0.0000
  Fe(III)hydroxamate 0.0003 0.0000
  Ferrichrome 0.0003 0.0000
  ITP 0.0003 0.0000
  2-Dehydro-3-deoxy-D-arabino-heptonate 7-phosphate 0.001 0.0000
  Pyridoxine 5'-phosphate 0.001 0.0000
  O-Phospho-4-hydroxy-L-threonine 0.001 0.0000
  Reduced riboflavin 0.003 0.0000
  Riboflavin 0.003 0.0000
  L-ascorbate-6-phosphate 0.005 0.0000
  L-Homoserine 0.005 0.0000
  L-Phenylalanine 0.005 0.0000
  GDP 0.005 0.0000
  Adenosine 0.008 0.0000
  dATP 0.008 0.0000
  L-Homocysteine 0.008 0.0000
  Thymidine 0.008 0.0000
    
 
