# Supplementary material for: Genomewide landscape of gene–metabolome associations in Escherichia coli
Source: Mol Syst Biol. 2017 Jan 16;13(1):907. doi: 10.15252/msb.20167150 (PMC5293155; doi:10.15252/msb.20167150)
Supplement: Supplementary file 4 — Table EV3 [file MSB-13-907-s004.zip › details/data_ybeH.html]

 
 
 ybeH 
  ybeH - details 
 
 
  CLR  
   Gene_matching CLR_index  rem 8.0
  yeeO 7.5
  hinT 7.5
  ydfO 7.5
  ydeP 6.9
  ylcG 6.7
  rtcB 6.7
  yfcS 6.6
  mutS 6.6
  ycfJ 6.3
  thrL 6.3
  hokD 6.3
  ybcL 6.3
  yphB 6.2
  yeaN 6.2
  ydeO 6.2
  lrhA 6.2
  ybbC 6.0
  wbbK 6.0
  dam 5.8
  yfdC 5.8
  ynjC 5.8
  nadR 5.8
  mioC 5.8
  yfcQ 5.7
  uspF 5.7
  narU 5.6
  pbl 5.6
  yaiT 5.5
  yagI 5.5
  nudD 5.4
  frlD 5.4
  ydiK 5.4
  lsrG 5.3
  clcB 5.2
  yfbT 5.2
  yggS 5.2
  yeaH 5.1
  mdtB 5.0
  ydhB 5.0
  yeiW 5.0
  rspB 5.0
  dinG 5.0
  yhhI 5.0
  sseA 5.0
  sufB 4.9
  yebU 4.9
  yedZ 4.9
  yfeW 4.8
  yqeK 4.8
  fbaB 4.8
  ygcR 4.8
  ycdW 4.7
  ydeN 4.7
  yegX 4.7
  setB 4.7
  mviM 4.7
  ydiQ 4.7
  yeiP 4.5
  yfgH 4.5
  sseB 4.5
  yecT 4.5
  ypjC 4.5
  yeiA 4.4
  dppB 4.4
  yfcU 4.4
  ycdR 4.3
  ydhO 4.3
  ybiR 4.3
  ydeH 4.3
  yfjD 4.3
  xerD 4.3
  hokA 4.3
  ung 4.3
  caiA 4.3
  yoaC 4.2
  crcA 4.2
  gidA 4.2
  wbbI 4.2
  nmpC 4.2
  glpR 4.2
  alsA 4.2
  yfbE 4.1
  yodB 4.1
  yhbO 4.1
  yfeN 4.1
  yeaP 4.1
  ybhQ 4.0
  yfdP 4.0
  yoaD 4.0
  ynjB 4.0
  yecM 4.0
  yphG 4.0
  yjaA 4.0
  yeeT 4.0
  mutM 4.0
  yfiL 4.0
  ydiA 4.0
  yegH 4.0
  gatR 3.9
  yfbJ 3.9
  ycjZ 3.9
  ydgJ 3.9
  smtA 3.9
  yohO 3.9
  yncC 3.9
  nikA 3.9
  marA 3.9
  yqgC 3.8
  yebV 3.8
  ygeQ 3.8
  acrR 3.8
  yncG 3.8
  phnL 3.8
  hybE 3.8
  tag 3.7
  yfcM 3.7
  glyS 3.7
  ybiN 3.7
  gidB 3.7
  ycdG 3.7
  yjgZ 3.7
  yfeR 3.7
  yohN 3.6
  ompG 3.6
  ydjY 3.6
  sufD 3.6
  citF 3.6
  ompW 3.6
  pfkB 3.6
  kil 3.6
  modA 3.6
  yegJ 3.6
  deaD 3.6
  yagF 3.6
  ymfN 3.6
  aqpZ 3.6
  yehD 3.6
  asnB 3.5
  yhfZ 3.5
  yfdS 3.5
  araH 3.5
  ychE 3.5
  ydhZ 3.5
  yjiJ 3.5
  yehL 3.5
  ybgI 3.5
  rsxC 3.5
  yfhK 3.5
  cueO 3.5
  ymgG 3.4
  essQ 3.4
  yafY 3.4
  yeeV 3.4
  rzpR 3.4
  yeaD 3.4
  glvC 3.4
  yqaC 3.4
  ecpD 3.4
  yjjB 3.4
  astC 3.4
  yegD 3.4
  yfjS 3.3
  ydjH 3.3
  yphH 3.3
  fixA 3.3
  eamA 3.3
  rfaZ 3.3
  citC 3.3
  trmC 3.3
  lar 3.3
  yagX 3.2
  ygeP 3.2
  yqeJ 3.2
  manY 3.2
  ygfS 3.2
  ygeK 3.2
  yciX 3.2
  apaG 3.2
  sfmH 3.2
  ycjD 3.2
  yfdL 3.2
  exoX 3.1
  ppdB 3.1
  yfaV 3.1
  ybfO 3.1
  rhsB 3.1
  yebW 3.1
  yagT 3.1
  yagE 3.1
  yoaB 3.1
  yedY 3.1
  malP 3.1
  yqgB 3.1
  iaaA 3.1
  yahM 3.1
  yhfT 3.1
  ycdN 3.1
  yegI 3.1
  yqhA 3.0
  yodD 3.0
  ycaQ 3.0
  hybA 3.0
  rhoL 3.0
  kptA 3.0
  ygbF 3.0
  ygcQ 3.0
  rhsE 3.0
  yhdP 3.0
  ydiI 3.0
     Differential ions  
   id name formula mz mod AUC Z-score Z-score AUC Weighted   C00079  L-Phenylalanine C9H11NO2 168.0925 [+2].H(+) 0.716 3.601 2.580
   C00704  Superoxide anion O2 206.8865 .HPO4K2.H(+) 0.636 3.609 2.293
   C00299  Uridine C9H12N2O6 246.0839 [+1].H(+) 0.596 -4.326 -0.000
   C04114  crotonobetaine C7H13NO2 168.0925 [+2].Na(+) 0.590 3.601 0.000
   C00007  O2 O2 206.8865 .HPO4K2.H(+) 0.526 3.609 0.000
   C04332  6,7-Dimethyl-8-(1-D-ribityl)lumazine C13H18N4O6 327.1241 .H(+) 0.000 -4.765 -0.000
     KEGG pathway by CLR  
   Pathway_ion pvalue_ion qvalue_ion  Riboflavin metabolism 4e-05 0.0036
  Galactose metabolism 0.0004 0.0220
  ABC transporters 0.0006 0.0198
  Arginine and proline metabolism 0.001 0.0300
     COG enrichment  
   Pathway_MS pvalue_MS qvalue_MS  Base excision repair 0.004 0.4588
  Mismatch repair 0.008 0.4012
     Predicted metabolites from CLR  
   Predicted metabolites Pvalue Overlap with hits  glucosyl-O-acetyl-rhamanosyl-N-acetylglucosamyl-undecaprenyl diphosphate 0 0.0000
  L-Asparagine 0.0001 0.0000
  [4Fe-4S] iron-sulfur cluster 0.0005 0.0000
  SufBCD with bound [4Fe-4S] cluster 0.0005 0.0000
  [2Fe-1S] desulfurated iron-sulfur cluster 0.0008 0.0000
  [2Fe-2S] iron-sulfur cluster 0.0008 0.0000
  D-Fructose 1,6-bisphosphate 0.0008 0.0000
  SufBCD with two bound [2Fe-2S] clusters 0.001 0.0000
  Uracil 0.004 0.0000
  UDP 0.009 0.0000
  SufBCD scaffold complex 0.01 0.0000
  SufSE sulfur acceptor complex 0.01 0.0000
  SufSE with bound sulfur 0.01 0.0000
    
 
