# Supplementary material for: Genomewide landscape of gene–metabolome associations in Escherichia coli
Source: Mol Syst Biol. 2017 Jan 16;13(1):907. doi: 10.15252/msb.20167150 (PMC5293155; doi:10.15252/msb.20167150)
Supplement: Supplementary file 4 — Table EV3 [file MSB-13-907-s004.zip › details/data_ybeQ.html]

 
 
 ybeQ 
  ybeQ - details 
 
 
  CLR  
   Gene_matching CLR_index  holD 11.2
  yjeK 10.7
  cybB 8.7
  ygeR 7.6
  sfmC 7.6
  ubiH 7.1
  ydcI 7.1
  ycgG 7.0
  cysH 7.0
  yjeO 6.9
  coaE 6.8
  ybeB 6.8
  ccmH 6.7
  ptsG 6.5
  ymdC 6.5
  rng 6.4
  pepB 6.3
  yzgL 6.3
  metL 6.2
  cof 6.1
  rcsF 6.0
  ydhV 6.0
  ybdR 6.0
  ygdB 6.0
  yraK 5.9
  dacD 5.9
  ilvB 5.9
  ygaZ 5.8
  fepA 5.8
  fadD 5.8
  yjdF 5.8
  betT 5.7
  yggV 5.6
  ykgN 5.6
  ygiQ 5.5
  pdxJ 5.4
  uspE 5.4
  cpxP 5.3
  ygcN 5.3
  hofB 5.3
  ygjN 5.3
  ydiZ 5.2
  yagP 5.1
  aroH 5.0
  cchB 5.0
  yjeH 4.9
  ycfP 4.9
  ydiQ 4.9
  yigE 4.9
  tpx 4.8
  hsdS 4.8
  sbmC 4.8
  ybfG 4.8
  tatC 4.8
  yifK 4.7
  yciW 4.7
  yjcS 4.7
  yjfJ 4.7
  yjhG 4.7
  yohH 4.6
  ygcM 4.6
  gadX 4.5
  yeaB 4.5
  ybaW 4.5
  puuD 4.5
  yfaQ 4.4
  yhaB 4.4
  glmM 4.4
  potA 4.3
  ydaS 4.3
  eptA 4.3
  hsdM 4.3
  yqjD 4.2
  feaR 4.2
  envZ 4.2
  yggT 4.2
  aroG 4.2
  yqeB 4.1
  gspO 4.1
  mrcB 4.1
  ypdG 4.1
  glyS 4.1
  yccZ 4.1
  ygeI 4.0
  ydgJ 4.0
  yceK 4.0
  ydjG 4.0
  uidA 4.0
  yccC 4.0
  mngB 4.0
  ydhJ 4.0
  ygjR 3.9
  ypjA 3.9
  ymcD 3.9
  fruK 3.9
  yfcV 3.8
  yaiO 3.8
  cmtA 3.8
  dacB 3.8
  ybgO 3.8
  fhuC 3.8
  yqjG 3.7
  mdtK 3.7
  ggt 3.7
  lysS 3.7
  yahC 3.7
  tpr 3.7
  rnb 3.7
  ynjB 3.7
  mrr 3.7
  dipZ 3.6
  ycaL 3.6
  ydcC 3.6
  efp 3.6
  hyfR 3.6
  ygfG 3.6
  rsgA 3.6
  yraP 3.5
  ygcE 3.5
  yeaV 3.5
  prlC 3.5
  fdnH 3.5
  murP 3.5
  crcB 3.5
  glf 3.4
  rfaL 3.4
  yieI 3.4
  ykfA 3.4
  essQ 3.3
  ybeZ 3.3
  ybhT 3.3
  yjfK 3.3
  rpiA 3.3
  rlmB 3.3
  nohA 3.3
  yfaU 3.3
  yfeA 3.3
  ycdM 3.3
  yehI 3.2
  yfbH 3.2
  yfcD 3.2
  iadA 3.2
  yciH 3.2
  marR 3.2
  bcsF 3.1
  yhaI 3.1
  sugE 3.1
  ykfG 3.1
  paaD 3.1
  ybgA 3.1
  yffH 3.1
  yfcO 3.1
  yhhM 3.1
  yciA 3.1
  ilvI 3.1
  ilvA 3.0
  fsaA 3.0
  phnJ 3.0
  ybiX 3.0
  vacJ 3.0
  ycdQ 3.0
  yeiQ 3.0
  ilvM 3.0
  yhbS 3.0
  ilvY 3.0
  fdoI 3.0
  metE 3.0
  ycdZ 3.0
  narZ 3.0
  yfcE 3.0
     Differential ions  
   id name formula mz mod AUC Z-score Z-score AUC Weighted   C00534  Pyridoxamine C8H12N2O2 191.0788 .H/Na.H(+) 0.799 3.818 3.049
   C00534  Pyridoxamine C8H12N2O2 191.0788 .Na(+) 0.799 3.818 3.049
   C00624  N-Acetyl-L-glutamate C7H11NO5 191.0788 [+1].H(+) 0.612 3.818 2.337
   C16155  UDP-4-keto-pyranose C14H20N2O16P2 708.9200 .HPO4K2.H(+) 0.473 3.620 0.000
     KEGG pathway by CLR  
none  COG enrichment  
   Pathway_MS pvalue_MS qvalue_MS  C5-Branched dibasic acid metabolism 0.0004 0.0343
  Pantothenate and CoA biosynthesis 0.0007 0.0283
  Valine, leucine and isoleucine biosynthesis 0.0009 0.0252
  Peptidoglycan biosynthesis 0.001 0.0209
  Arachidonic acid metabolism 0.002 0.0335
  Aminoacyl-tRNA biosynthesis 0.003 0.0378
  Other glycan degradation 0.006 0.0697
     Predicted metabolites from CLR  
   Predicted metabolites Pvalue Overlap with hits  ITP 2e-05 0.0000
  2-Dehydro-3-deoxy-D-arabino-heptonate 7-phosphate 9e-05 0.0000
  2-Oxobutanoate 0.0001 0.0000
  (S)-2-Aceto-2-hydroxybutanoate 0.0002 0.0000
  (S)-2-Acetolactate 0.0002 0.0000
  Choline 0.001 0.0000
  D-Erythrose 4-phosphate 0.001 0.0000
  Glycine 0.005 0.0000
    
 
