# Supplementary material for: Genomewide landscape of gene–metabolome associations in Escherichia coli
Source: Mol Syst Biol. 2017 Jan 16;13(1):907. doi: 10.15252/msb.20167150 (PMC5293155; doi:10.15252/msb.20167150)
Supplement: Supplementary file 4 — Table EV3 [file MSB-13-907-s004.zip › details/data_ybeT.html]

 
 
 ybeT 
  ybeT - details 
 
 
  CLR  
   Gene_matching CLR_index  bioA 16.3
  cusR 16.0
  yeeS 15.3
  yebE 14.5
  atoS 14.2
  phoQ 13.9
  yecH 13.8
  glpD 13.3
  ydhT 13.1
  yciV 13.0
  recF 12.4
  yceA 12.0
  glpE 12.0
  endA 11.9
  pldA 11.8
  yoaE 11.8
  cfa 11.8
  ycdB 11.7
  ychJ 11.5
  tyrB 11.5
  ydeJ 11.5
  yfgL 11.4
  secG 11.4
  torR 11.4
  ycbC 11.0
  ybgL 11.0
  btuF 10.8
  yibA 10.7
  yncI 10.6
  aceE 10.5
  nudG 10.4
  yebY 10.3
  ygdK 10.1
  ycaD 9.9
  kdpD 9.9
  ycjS 9.7
  pstA 9.7
  galM 9.7
  osmB 9.6
  yfcZ 9.4
  ydgA 9.4
  idnD 9.2
  ycbJ 9.2
  yoaH 9.2
  lldR 9.2
  dsbC 9.2
  yobB 9.0
  hyaB 8.9
  proX 8.8
  yecR 8.7
  nfnB 8.7
  hyaA 8.5
  ybhD 8.5
  ybdN 8.5
  renD 8.4
  aslA 8.4
  fabF 8.2
  yciS 8.1
  chbG 8.0
  nrfD 8.0
  pepT 8.0
  sucC 7.9
  hflX 7.9
  ypfG 7.8
  lon 7.7
  fdhE 7.4
  gshB 7.4
  yajB 7.3
  ybcO 7.3
  xylR 7.3
  hyaD 7.3
  ybiS 7.1
  yicH 7.1
  ybhB 7.0
  xylB 6.8
  maoC 6.8
  nrfC 6.7
  crr 6.7
  syd 6.6
  ompT 6.6
  chbC 6.4
  ybdD 6.4
  sdhC 6.4
  umuD 6.4
  gspC 6.3
  fimA 6.3
  yedE 6.3
  ydiY 6.2
  hdhA 6.1
  sdhA 6.1
  ybcI 6.0
  ugpQ 6.0
  arcB 6.0
  yjcC 5.9
  hyuA 5.7
  glnG 5.7
  guaC 5.6
  ydcY 5.6
  ygaW 5.5
  ybjH 5.5
  ycfK 5.5
  ybgD 5.5
  yadB 5.5
  ydeU 5.5
  feaR 5.5
  nhaA 5.5
  frmR 5.4
  ydcK 5.4
  grxC 5.4
  ybjP 5.4
  yjeB 5.3
  ybeA 5.3
  tatE 5.3
  hybO 5.3
  ttdA 5.3
  yaiI 5.2
  ygjM 5.1
  fbp 5.1
  yehE 5.1
  metF 5.0
  ygaP 5.0
  yijF 5.0
  flgD 5.0
  fixC 4.8
  yedD 4.8
  yhiI 4.8
  ycbY 4.7
  ydiE 4.7
  yjbD 4.7
  malS 4.7
  ylbE 4.7
  thiE 4.6
  ygjH 4.6
  eutH 4.5
  yhbP 4.5
  yegQ 4.5
  gsk 4.4
  pps 4.4
  srmB 4.4
  yidK 4.4
  mog 4.4
  yciU 4.3
  yifN 4.3
  yicI 4.3
  yfjU 4.3
  yedR 4.3
  ydcQ 4.2
  ygiH 4.2
  ptsP 4.2
  emrB 4.1
  yiaI 4.1
  speE 4.1
  menA 4.1
  hemX 4.1
  rpsT 4.1
  yeiU 4.0
  rpoZ 4.0
  rcsD 4.0
  yfaW 4.0
  ybbS 4.0
  yeaY 3.9
  ybjN 3.9
  yqhD 3.9
  ygbL 3.9
  ynjF 3.9
  yfaT 3.9
  sfsB 3.9
  ygcW 3.9
  flgM 3.8
  speA 3.8
  pstB 3.8
  ycfN 3.8
  yiaW 3.8
  leuL 3.8
  nanE 3.8
  ynjH 3.8
  yaeP 3.7
  yiaU 3.7
  yciM 3.7
  yfhM 3.7
  yeiL 3.7
  ydeS 3.7
  yfeZ 3.6
  fdrA 3.6
  ybcM 3.6
  ascB 3.6
  mglB 3.6
  ydfA 3.6
  dcuC 3.5
  ynaJ 3.5
  ycbG 3.5
  bioD 3.5
  ypfE 3.5
  ydgK 3.5
  rffC 3.5
  citT 3.5
  yegX 3.5
  fdhD 3.5
  yjgI 3.5
  kbl 3.5
  rihC 3.5
  bglB 3.5
  yfeH 3.5
  degP 3.4
  yhjX 3.4
  ypjF 3.4
  pepA 3.4
  ilvA 3.4
  sfmA 3.4
  mtlD 3.4
  yqjB 3.4
  mdlA 3.3
  rfaQ 3.3
  cydB 3.3
  tar 3.3
  tsx 3.3
  fsr 3.3
  cusS 3.3
  agaC 3.3
  yaeB 3.3
  rraA 3.3
  ydcL 3.3
  ycfD 3.2
  alr 3.2
  yejK 3.2
  fadR 3.2
  potA 3.2
  yciF 3.2
  xylA 3.2
  kdsC 3.2
  yodB 3.1
  clpP 3.1
  hofC 3.1
  ysaB 3.1
  amtB 3.1
  ddpF 3.1
  ydhA 3.1
  ydgH 3.1
  zur 3.1
  flgE 3.1
  ydcJ 3.1
  hyfG 3.0
  narJ 3.0
  ybcN 3.0
  pstC 3.0
  gpp 3.0
     Differential ions  
   id name formula mz mod AUC Z-score Z-score AUC Weighted   cyclopropane phosphatidylglycerol (dihexadec-9,10-cyclo-anoyl, n-C16:0 cyclo)  cyclopropane phosphatidylglycerol (dihexadec-9,10-cyclo-anoyl, n-C16:0 cyclo) C40H75O10P1 746.4966 [+1]-H(+) 0.951 6.328 6.016
   cyclopropane phosphatidylglycerol (dihexadec-9,10-cyclo-anoyl, n-C16:0 cyclo)  cyclopropane phosphatidylglycerol (dihexadec-9,10-cyclo-anoyl, n-C16:0 cyclo) C40H75O10P1 745.4931 -H(+) 0.959 5.668 5.436
   C05973  2-Acyl-sn-glycero-3-phosphoethanolamine (n-C18:1) C23H46NO7P1 479.2903 [+1]-H(+) 0.630 7.593 4.783
   C07836  D-Glycero-D-manno-heptose 7-phosphate C7H15O10P 245.0432 -CO2-H(+) 0.934 5.009 4.678
   C00249  Hexadecanoate (n-C16:0) C16H32O2 257.2389 [+2]-H(+) 0.667 6.986 4.662
   C05973  2-Acyl-sn-glycero-3-phosphoethanolamine (n-C18:1) C23H46NO7P1 478.2895 -H(+) 0.610 7.633 4.657
   C00641  1,2-Diacyl-sn-glycerol (dioctadecanoyl, n-C18:0) C39H76O5 743.5397 .H2PO4Na-H(+) 0.938 4.930 4.626
   cyclopropane phosphatidylethanolamine (dihexadec-9,10-cyclo-anoyl, n-C16:0 cyclo)  cyclopropane phosphatidylethanolamine (dihexadec-9,10-cyclo-anoyl, n-C16:0 cyclo) C39H74N1O8P1 714.5026 -H(+) 0.993 4.331 4.300
   C03406  N(omega)-(L-Arginino)succinate C10H18N4O6 409.0704 .H2PO4Na-H(+) 0.950 4.202 3.991
   C05382  Sedoheptulose 7-phosphate C7H15O10P 245.0432 -CO2-H(+) 0.773 5.009 3.872
   octadecenoate (n-C18:1)  octadecenoate (n-C18:1) C18H34O2 401.2192 .H2PO4Na-H(+) 0.638 5.983 3.814
   C00344  Phosphatidylglycerol (dioctadec-11-enoyl, n-C18:1) C42H79O10P1 773.5238 -H(+) 0.683 5.534 3.778
   C03974  2-tetradecanoyl-sn-glycerol 3-phosphate C17H35O7P1 401.2192 [+2]+OH(-) 0.626 5.983 3.742
   C07838  D-Glycero-D-manno-heptose 1-phosphate C7H15O10P 245.0432 -CO2-H(+) 0.727 5.009 3.641
   cyclopropane phosphatidylethanolamine (dihexadec-9,10-cyclo-anoyl, n-C16:0 cyclo)  cyclopropane phosphatidylethanolamine (dihexadec-9,10-cyclo-anoyl, n-C16:0 cyclo) C39H74N1O8P1 715.5062 [+1]-H(+) 0.993 3.665 3.639
   C00575  cAMP C10H12N5O6P 329.0479 [+1]-H(+) 0.802 4.270 3.425
   C00641  1,2-Diacyl-sn-glycerol (dioctadec-11-enoyl, n-C18:1) C39H72O5 637.5334 +OH(-) 0.934 3.621 3.383
   C00612  N1-Acetylspermidine C9H21N3O 328.1004 .HPO4Na2-H(+) 0.954 3.519 3.357
   C01029  N8-Acetylspermidine C9H21N3O 328.1004 .HPO4Na2-H(+) 0.954 3.519 3.357
   C00350  phosphatidylethanolamine (dioctadec-11-enoyl, n-C18:1) C41H78N1O8P1 743.5397 [+1]-H(+) 0.664 4.930 3.272
   C03451  (R)-S-Lactoylglutathione C13H21N3O8S 378.0988 -H(+) 0.807 3.909 3.156
   C00350  phosphatidylethanolamine (dioctadec-11-enoyl, n-C18:1) C41H78N1O8P1 742.5309 -H(+) 0.706 4.469 3.154
   C03974  2-octadec-11-enoyl-sn-glycerol 3-phosphate C21H41O7P1 453.2753 +OH(-) 0.837 3.681 3.080
   C00575  cAMP C10H12N5O6P 328.0454 -H(+) 0.773 3.984 3.078
   C05818  2-Demethylmenaquinone 8 C50H70O2 837.4572 .H2PO4K-H(+) 0.627 4.667 2.925
   C00575  cAMP C10H12N5O6P 330.0516 [+2]-H(+) 0.718 4.031 2.893
   C00641  1,2-Diacyl-sn-glycerol (dioctadec-11-enoyl, n-C18:1) C39H72O5 837.4572 .(H2PO4)2NaH-H(+) 0.617 4.667 2.880
   C00350  phosphatidylethanolamine (dioctadec-11-enoyl, n-C18:1) C41H78N1O8P1 960.4842 .(H2PO4)2NaH-H(+) 0.693 3.960 2.745
   C06424  tetradecanoate (n-C14:0) C14H28O2 228.2032 [+1]-H(+) 0.610 4.385 2.674
   C05973  2-Acyl-sn-glycero-3-phosphoethanolamine (n-C16:0) C21H44NO7P1 453.2753 [+1]-H(+) 0.723 3.681 2.661
   C00942  3',5'-Cyclic GMP C10H12N5O7P 344.0409 -H(+) 0.734 3.531 2.593
   C18239  cyclic pyranopterin monophosphate C10H14N5O8P 344.0409 -H2O-H(+) 0.722 3.531 2.551
   2-Acyl-sn-glycero-3-phosphoglycerol (n-C18:1)  2-Acyl-sn-glycero-3-phosphoglycerol (n-C18:1) C24H47O9P1 509.2846 -H(+) 0.624 4.022 2.511
   C00350  phosphatidylethanolamine (dihexadecanoyl, n-C16:0) C37H74N1O8P1 690.5013 -H(+) 0.641 3.905 2.505
   C00350  phosphatidylethanolamine (dihexadec-9enoyl, n-C16:1) C37H70N1O8P1 686.4716 -H(+) 0.685 3.592 2.460
   C00681  1-octadec-11-enoyl-sn-glycerol 3-phosphate C21H41O7P1 453.2753 +OH(-) 0.650 3.681 2.392
   C00144  GMP C10H14N5O8P 344.0409 -H2O-H(+) 0.593 3.531 0.000
   C00053  3'-Phosphoadenylyl sulfate C10H15N5O13P2S 745.8898 .(H2PO4Na)2-H(+) 0.589 -3.684 -0.000
   C05973  2-Acyl-sn-glycero-3-phosphoethanolamine (n-C16:1) C21H42NO7P1 570.2196 .H2PO4Na-H(+) 0.585 5.512 0.000
   C03296  N2-Succinyl-L-arginine C10H18N4O5 409.0704 .H2PO4K-H(+) 0.574 4.202 0.000
   C00186  L-Lactate C3H6O3 360.8865 .(H2PO4K)2-H(+) 0.573 -4.684 -0.000
   C05973  2-Acyl-sn-glycero-3-phosphoethanolamine (n-C16:1) C21H42NO7P1 451.2614 [+1]-H(+) 0.571 6.859 0.000
   C08362  Hexadecenoate (n-C16:1) C16H30O2 253.2177 -H(+) 0.566 9.283 0.000
   C00256  D-Lactate C3H6O3 360.8865 .(H2PO4K)2-H(+) 0.563 -4.684 -0.000
   C04421  N-Succinyl-LL-2,6-diaminoheptanedioate C11H18N2O7 409.0704 .H2PO4Na-H(+) 0.557 4.202 0.000
   C05973  2-Acyl-sn-glycero-3-phosphoethanolamine (n-C16:0) C21H44NO7P1 452.2770 -H(+) 0.554 6.025 0.000
   C00184  Dihydroxyacetone C3H6O3 360.8865 .(H2PO4K)2-H(+) 0.553 -4.684 -0.000
   C08362  Hexadecenoate (n-C16:1) C16H30O2 254.2217 [+1]-H(+) 0.550 8.935 0.000
   C00681  1-tetradecanoyl-sn-glycerol 3-phosphate C17H35O7P1 401.2192 [+2]+OH(-) 0.549 5.983 0.000
   C01013  3-Hydroxypropanoate C3H6O3 360.8865 .(H2PO4K)2-H(+) 0.527 -4.684 -0.000
   octadecenoate (n-C18:1)  octadecenoate (n-C18:1) C18H34O2 281.2478 -H(+) 0.511 10.758 0.000
   C00344  Phosphatidylglycerol (dioctadec-11-enoyl, n-C18:1) C42H79O10P1 775.5345 [+2]-H(+) 0.510 5.905 0.000
   C00249  Hexadecanoate (n-C16:0) C16H32O2 256.2353 [+1]-H(+) 0.505 7.902 0.000
   C00249  Hexadecanoate (n-C16:0) C16H32O2 255.2327 -H(+) 0.498 8.305 0.000
   C00362  dGMP C10H14N5O7P 328.0454 -H2O-H(+) 0.489 3.984 0.000
   C03274  Glycerophosphoglycerol C6H15O8P 245.0432 -H(+) 0.488 5.009 0.000
   octadecenoate (n-C18:1)  octadecenoate (n-C18:1) C18H34O2 282.2521 [+1]-H(+) 0.458 10.207 0.000
   C01233  sn-Glycero-3-phosphoethanolamine C5H14NO6P 214.0496 -H(+) 0.443 5.448 0.000
   C05973  2-Acyl-sn-glycero-3-phosphoethanolamine (n-C16:1) C21H42NO7P1 450.2618 -H(+) 0.416 6.523 0.000
   C05809  3-Octaprenyl-4-hydroxybenzoate C47H70O3 719.4862 .H/K-H(+) 0.000 4.768 0.000
   C05810  2-Octaprenylphenol C46H70O 637.5334 -H(+) 0.000 3.621 0.000
   C00286  dGTP C10H16N5O13P3 745.8898 .(H2PO4Na)2-H(+) 0.715 -3.684 -2.636
   C05973  2-Acyl-sn-glycero-3-phosphoethanolamine (n-C16:0) C21H44NO7P1 672.2375 .(H2PO4)2NaH.H(+) 0.726 -3.646 -2.646
   C11453  2-C-methyl-D-erythritol 2,4-cyclodiphosphate C5H12O9P2 548.8469 .(H2PO4K)2-H(+) 0.630 -4.242 -2.671
   C00577  D-Glyceraldehyde C3H6O3 360.8865 .(H2PO4K)2-H(+) 0.655 -4.684 -3.068
     KEGG pathway by CLR  
   Pathway_ion pvalue_ion qvalue_ion  Fatty acid biosynthesis 1e-07 0.0000
  Purine metabolism 6e-06 0.0003
  Biosynthesis of unsaturated fatty acids 9e-05 0.0029
  Fatty acid metabolism 0.0001 0.0027
  Glycerophospholipid metabolism 0.002 0.0453
  Pyruvate metabolism 0.006 0.0953
  Ubiquinone and other terpenoid-quinone biosynthesis 0.007 0.0996
     COG enrichment  
   Pathway_MS pvalue_MS qvalue_MS  RNA polymerase 0 0.0000
  Nitrotoluene degradation 2e-05 0.0009
  Biotin metabolism 0.002 0.0738
  Glycolysis / Gluconeogenesis 0.003 0.0687
  Fatty acid biosynthesis 0.004 0.0758
  Sphingolipid metabolism 0.004 0.0632
  Protein export 0.008 0.1003
  Glycerophospholipid metabolism 0.01 0.1075
     Predicted metabolites from CLR  
   Predicted metabolites Pvalue Overlap with hits  7,8-Diaminononanoate 0 0.0000
  myo-Inositol 0 0.0000
  D-Xylulose 0 0.0000
  Decanoyl-ACP (n-C10:0ACP) 0.0002 0.0000
  Thiamin monophosphate 0.0002 0.0000
  Dodecanoyl-ACP (n-C12:0ACP) 0.0006 0.0000
  Octanoyl-ACP (n-C8:0ACP) 0.0006 0.0000
  Palmitoyl-ACP (n-C16:0ACP) 0.0006 0.0000
  cis-hexadec-9-enoyl-[acyl-carrier protein] (n-C16:1) 0.001 0.0000
  Myristoyl-ACP (n-C14:0ACP) 0.003 0.0000
  IMP 0.005 0.0000
  Inosine 0.007 0.0000
    
 
