# Supplementary material for: Genomewide landscape of gene–metabolome associations in Escherichia coli
Source: Mol Syst Biol. 2017 Jan 16;13(1):907. doi: 10.15252/msb.20167150 (PMC5293155; doi:10.15252/msb.20167150)
Supplement: Supplementary file 4 — Table EV3 [file MSB-13-907-s004.zip › details/data_ybeU.html]

 
 
 ybeU 
  ybeU - details 
 
 
  CLR  
   Gene_matching CLR_index  yqcD 15.7
  yehB 15.4
  pykF 13.5
  ydhC 13.4
  ybgT 13.3
  adhE 12.9
  ybiA 12.6
  frdC 12.5
  yagY 12.3
  ompA 12.2
  cheY 12.0
  ydjZ 12.0
  degQ 11.8
  ymfC 10.7
  ybaQ 10.6
  ydiB 10.1
  creB 9.5
  ydcP 9.4
  nuoN 9.4
  slp 9.2
  hycH 8.8
  ylbA 8.8
  narQ 8.6
  ydaV 8.5
  ycbT 8.3
  iscA 8.2
  yajD 8.2
  yfbR 7.9
  frdB 7.9
  ybfB 7.9
  cydB 7.7
  aceE 7.6
  yqcE 7.6
  yfeK 7.6
  fimD 7.6
  yfgM 7.6
  clpX 7.4
  ycaP 7.4
  ydiN 7.3
  ygdQ 7.0
  lldD 6.9
  hslJ 6.9
  talB 6.8
  degP 6.4
  yncD 6.3
  ackA 6.2
  moaA 6.1
  ybbB 6.0
  panE 6.0
  yahE 5.9
  fepA 5.9
  hslU 5.8
  eutA 5.8
  yjfF 5.7
  hisA 5.7
  dbpA 5.6
  ybgP 5.6
  glcB 5.4
  ydcU 5.4
  ybeM 5.3
  feaB 5.3
  yhfS 5.1
  pepD 5.0
  nuoJ 5.0
  ahpF 4.9
  mutL 4.9
  ybjC 4.9
  yaiP 4.8
  yeaC 4.8
  yneH 4.8
  fiu 4.7
  clpS 4.7
  yrbE 4.6
  argP 4.5
  yhhK 4.5
  cusA 4.4
  prc 4.3
  ygaX 4.3
  ybhC 4.3
  cysW 4.2
  pta 4.2
  ybjH 4.2
  ybjO 4.2
  yebQ 4.2
  ybgC 4.2
  kdgR 4.1
  radA 4.1
  ykfA 4.1
  ydfT 4.1
  ydhY 4.1
  pps 4.0
  lipB 4.0
  citC 4.0
  ynfE 4.0
  panB 4.0
  dcuR 4.0
  panC 4.0
  dcuS 3.9
  napA 3.8
  ybdD 3.8
  elbA 3.8
  yedZ 3.8
  sufA 3.8
  rnt 3.8
  ydeW 3.8
  ydjX 3.7
  ydfX 3.7
  cusF 3.7
  yfdR 3.6
  yfdQ 3.6
  metJ 3.6
  qseC 3.6
  yaiE 3.5
  rsxA 3.5
  rstA 3.4
  yeiJ 3.4
  yhaI 3.4
  yfcJ 3.4
  ygeF 3.4
  ycdB 3.3
  clpP 3.3
  ygiU 3.3
  modC 3.3
  ygjF 3.3
  phoB 3.3
  ygjH 3.3
  yiiU 3.3
  eutL 3.3
  ynaI 3.2
  pgm 3.2
  ybjL 3.1
  ddpC 3.1
  cadC 3.1
  fumC 3.1
  ytjC 3.1
  yohC 3.1
  nuoH 3.1
  panD 3.1
  hisH 3.0
  yqjE 3.0
  yfhD 3.0
  pstA 3.0
  yfeC 3.0
     Differential ions  
   id name formula mz mod AUC Z-score Z-score AUC Weighted   C00612  N1-Acetylspermidine C9H21N3O 188.1754 .H(+) 0.984 9.696 9.544
   C01029  N8-Acetylspermidine C9H21N3O 188.1754 .H(+) 0.984 9.696 9.544
   C00612  N1-Acetylspermidine C9H21N3O 210.1577 .Na(+) 0.912 9.040 8.242
   C00612  N1-Acetylspermidine C9H21N3O 210.1577 .H/Na.H(+) 0.912 9.040 8.242
   C01029  N8-Acetylspermidine C9H21N3O 210.1577 .Na(+) 0.912 9.040 8.242
   C01029  N8-Acetylspermidine C9H21N3O 210.1577 .H/Na.H(+) 0.912 9.040 8.242
   C05973  2-Acyl-sn-glycero-3-phosphoethanolamine (n-C16:1) C21H42NO7P1 490.2320 .H/K.H(+) 0.709 11.108 7.874
   C05973  2-Acyl-sn-glycero-3-phosphoethanolamine (n-C16:1) C21H42NO7P1 490.2320 .K(+) 0.709 11.108 7.874
   C00612  N1-Acetylspermidine C9H21N3O 171.1494 -NH3.H(+) 0.793 9.405 7.463
   C01029  N8-Acetylspermidine C9H21N3O 171.1494 -NH3.H(+) 0.793 9.405 7.463
   C00612  N1-Acetylspermidine C9H21N3O 190.1816 [+2].H(+) 0.866 8.123 7.035
   C01029  N8-Acetylspermidine C9H21N3O 190.1816 [+2].H(+) 0.866 8.123 7.035
   C05973  2-Acyl-sn-glycero-3-phosphoethanolamine (n-C16:1) C21H42NO7P1 452.2758 .H(+) 0.616 11.070 6.823
   C00612  N1-Acetylspermidine C9H21N3O 189.1781 [+1].H(+) 0.663 9.840 6.528
   C01029  N8-Acetylspermidine C9H21N3O 189.1781 [+1].H(+) 0.663 9.840 6.528
   C01216  2-Dehydro-3-deoxy-D-galactonate C6H10O6 199.0209 .H/Na-H(+) 0.984 6.522 6.418
   C00522  (R)-Pantoate C6H12O4 169.0477 .H/Na-H(+) 0.724 8.609 6.233
   C06007  (R)-2,3-Dihydroxy-3-methylpentanoate C6H12O4 169.0477 .H/Na-H(+) 0.701 8.609 6.033
   C05973  2-Acyl-sn-glycero-3-phosphoethanolamine (n-C16:1) C21H42NO7P1 474.2614 .H/Na.H(+) 0.611 7.366 4.498
   C05973  2-Acyl-sn-glycero-3-phosphoethanolamine (n-C16:1) C21H42NO7P1 474.2614 .Na(+) 0.611 7.366 4.498
   C00350  phosphatidylethanolamine (dihexadecanoyl, n-C16:0) C37H74N1O8P1 692.5148 .H(+) 0.785 5.616 4.409
   5'-deoxyribose  5'-deoxyribose C5H10O4 115.0401 -H2O-H(+) 0.999 4.383 4.378
   C05649  6,7-Dihydropteridine C6H6N4 115.0401 -H2O-H(+) 0.988 4.383 4.330
   C05973  2-Acyl-sn-glycero-3-phosphoethanolamine (n-C16:0) C21H44NO7P1 492.2489 .H/K.H(+) 0.673 6.237 4.195
   C00864  (R)-Pantothenate C9H17NO5 242.1012 .H/Na.H(+) 0.652 5.631 3.674
   C06054  2-Oxo-3-hydroxy-4-phosphobutanoate C4H7O8P 446.8820 .(H2PO4)2KH-H(+) 0.761 4.684 3.564
   C00141  3-Methyl-2-oxobutanoate C5H8O3 115.0401 -H(+) 0.774 4.383 3.393
   C00183  L-Valine C5H11NO2 100.0755 -H2O.H(+) 0.608 5.566 3.382
   C00719  Glycine betaine C5H11NO2 100.0755 -H2O.H(+) 0.602 5.566 3.352
   C00624  N-Acetyl-L-glutamate C7H11NO5 212.0537 .H/Na.H(+) 0.811 3.978 3.228
   C00624  N-Acetyl-L-glutamate C7H11NO5 212.0537 .Na(+) 0.811 3.978 3.228
   C05973  2-Acyl-sn-glycero-3-phosphoethanolamine (n-C16:0) C21H44NO7P1 455.3024 [+1].H(+) 0.670 4.646 3.112
   C02780  2,5-diketo-D-gluconate C6H8O7 228.9755 .H/K-H(+) 0.867 3.535 3.066
   C00522  (R)-Pantoate C6H12O4 185.0187 .H/K-H(+) 0.812 3.759 3.054
   C06257  1-deoxy-D-xylulose C5H10O4 115.0401 -H2O-H(+) 0.663 4.383 2.908
   C15809  dehydroglycine C2H3NO2 75.0260 [+1].H(+) 0.758 3.814 2.891
   C04272  (R)-2,3-Dihydroxy-3-methylbutanoate C5H10O4 115.0401 -H2O-H(+) 0.646 4.383 2.831
   C04575  2,3-Dioxo-L-gulonate C6H8O7 228.9755 .H/K-H(+) 0.756 3.535 2.674
   C12621  3-hydroxycinnamic acid C9H8O3 185.0187 .H/Na-H(+) 0.709 3.759 2.666
   C00624  N-Acetyl-L-glutamate C7H11NO5 228.0226 .H/K.H(+) 0.697 3.773 2.631
   C00526  Deoxyuridine C9H12N2O5 212.0537 -NH3.H(+) 0.654 3.978 2.601
   C06007  (R)-2,3-Dihydroxy-3-methylpentanoate C6H12O4 185.0187 .H/K-H(+) 0.675 3.759 2.539
   C03287  L-Glutamate 5-phosphate C5H10NO7P 228.0226 .H(+) 0.663 3.773 2.500
   C00052  UDPgalactose C15H24N2O17P2 589.0434 .H/Na.H(+) 0.650 3.801 2.472
   C01092  8-Amino-7-oxononanoate C9H17NO3 169.0860 -NH3-H(+) 0.680 3.549 2.415
   C00311  Isocitrate C6H8O7 228.9755 .H/K-H(+) 0.677 3.535 2.392
   C00294  Inosine C10H12N4O5 267.0740 -H(+) 0.597 5.232 0.000
   C00148  L-Proline C5H9NO2 138.0532 .Na(+) 0.595 -3.959 -0.000
   C00148  L-Proline C5H9NO2 138.0532 .H/Na.H(+) 0.595 -3.959 -0.000
   C00407  L-Isoleucine C6H13NO2 114.0910 -H2O.H(+) 0.592 3.792 0.000
   C00168  Hydroxypyruvate C3H4O4 222.9624 .H2PO4Na-H(+) 0.590 3.884 0.000
   C01146  2-Hydroxy-3-oxopropanoate C3H4O4 222.9624 .H2PO4Na-H(+) 0.584 3.884 0.000
   C00029  UDPglucose C15H24N2O17P2 589.0434 .H/Na.H(+) 0.583 3.801 0.000
   C00679  5-Dehydro-4-deoxy-D-glucarate C6H8O7 228.9755 .H/K-H(+) 0.574 3.535 0.000
   C00258  (R)-Glycerate C3H6O4 87.0096 -H2O-H(+) 0.573 7.543 0.000
   C01909  Dethiobiotin C10H18N2O3 171.1494 -CO2.H(+) 0.571 9.405 0.000
   C00166  Phenylpyruvate C9H8O3 185.0187 .H/Na-H(+) 0.564 3.759 0.000
   C00051  Reduced glutathione C10H17N3O6S 346.0477 .H/K.H(+) 0.563 3.640 0.000
   C00051  Reduced glutathione C10H17N3O6S 346.0477 .K(+) 0.563 3.640 0.000
   C00108  Anthranilate C7H7NO2 138.0532 .H(+) 0.560 -3.959 -0.000
   C04734  5-Formamido-1-(5-phospho-D-ribosyl)imidazole-4-carboxamide C10H15N4O9P 285.0821 -HPO3-H(+) 0.557 4.660 0.000
   C00022  Pyruvate C3H4O3 108.9912 .H/Na-H(+) 0.554 5.742 0.000
   C14899  3-keto-L-gulonate-6-phosphate C6H11O10P 446.8820 .HPO4K2-H(+) 0.553 4.684 0.000
   C00294  Inosine C10H12N4O5 285.0821 +OH(-) 0.549 4.660 0.000
   C08362  Hexadecenoate (n-C16:1) C16H30O2 277.2190 .H/Na.H(+) 0.544 6.363 0.000
   C00123  L-Leucine C6H13NO2 114.0910 -H2O.H(+) 0.536 3.792 0.000
   C00148  L-Proline C5H9NO2 154.0266 .H/K.H(+) 0.533 -4.380 -0.000
   C00148  L-Proline C5H9NO2 154.0266 .K(+) 0.533 -4.380 -0.000
   C05973  2-Acyl-sn-glycero-3-phosphoethanolamine (n-C16:1) C21H42NO7P1 453.2770 [+1].H(+) 0.531 8.022 0.000
   C00864  (R)-Pantothenate C9H17NO5 258.0733 .H/K.H(+) 0.526 5.876 0.000
   C05973  2-Acyl-sn-glycero-3-phosphoethanolamine (n-C16:1) C21H42NO7P1 594.2159 .HPO4Na2.H(+) 0.523 3.668 0.000
   C00022  Pyruvate C3H4O3 87.0096 -H(+) 0.516 7.543 0.000
   C00249  Hexadecanoate (n-C16:0) C16H32O2 258.2492 [+1].H(+) 0.510 3.587 0.000
   C00055  CMP C9H14N3O8P 346.0477 .H/Na.H(+) 0.497 3.640 0.000
   C00249  Hexadecanoate (n-C16:0) C16H32O2 257.2482 .H(+) 0.493 4.083 0.000
   C00158  Citrate C6H8O7 228.9755 .H/K-H(+) 0.491 3.535 0.000
   C05973  2-Acyl-sn-glycero-3-phosphoethanolamine (n-C18:1) C23H46NO7P1 480.3073 .H(+) 0.489 4.811 0.000
   C00993  D-Alanyl-D-alanine C6H12N2O3 117.1015 -CO2.H(+) 0.479 8.778 0.000
   C00222  Malonate semialdehyde C3H4O3 87.0096 -H(+) 0.467 7.543 0.000
   C00222  Malonate semialdehyde C3H4O3 108.9912 .H/Na-H(+) 0.464 5.742 0.000
   C03733  UDP-D-galacto-1,4-furanose C15H24N2O17P2 589.0434 .H/Na.H(+) 0.447 3.801 0.000
   C00204  2-Dehydro-3-deoxy-D-gluconate C6H10O6 199.0209 .H/Na-H(+) 0.403 6.522 0.000
   C00341  Geranyl diphosphate C10H20O7P2 331.0756 +OH(-) 0.000 3.866 0.000
   C04732  4-(1-D-Ribitylamino)-5-aminouracil C9H16N4O6 295.1178 [+2]+OH(-) 0.000 3.995 0.000
   C00082  L-Tyrosine C9H11NO3 138.0900 -CO2.H(+) 0.619 -4.460 -2.763
   C00568  4-Aminobenzoate C7H7NO2 138.0532 .H(+) 0.943 -3.959 -3.731
     KEGG pathway by CLR  
   Pathway_ion pvalue_ion qvalue_ion  Tyrosine metabolism 4e-08 0.0000
  Valine, leucine and isoleucine degradation 6e-06 0.0001
  D-Alanine metabolism 4e-05 0.0005
  Aminoacyl-tRNA biosynthesis 6e-05 0.0006
  Valine, leucine and isoleucine biosynthesis 7e-05 0.0007
  Biosynthesis of unsaturated fatty acids 0.0002 0.0016
  Arginine and proline metabolism 0.0002 0.0016
  Tryptophan metabolism 0.0003 0.0018
  Microbial metabolism in diverse environments 0.0005 0.0026
  Xylene degradation 0.0006 0.0031
  Fatty acid biosynthesis 0.0007 0.0032
  Fatty acid metabolism 0.001 0.0056
  Novobiocin biosynthesis 0.001 0.0052
  Thiamine metabolism 0.001 0.0049
  Dioxin degradation 0.002 0.0060
  Peptidoglycan biosynthesis 0.004 0.0138
  Phenylalanine, tyrosine and tryptophan biosynthesis 0.005 0.0153
  Caprolactam degradation 0.006 0.0171
  Chloroalkane and chloroalkene degradation 0.006 0.0174
  Folate biosynthesis 0.006 0.0165
  Pantothenate and CoA biosynthesis 0.008 0.0200
     COG enrichment  
   Pathway_MS pvalue_MS qvalue_MS  Pyruvate metabolism 6e-06 0.0005
  Two-component system 6e-05 0.0022
  Oxidative phosphorylation 0.0001 0.0029
  Histidine metabolism 0.0002 0.0040
  Microbial metabolism in diverse environments 0.0002 0.0033
  Pantothenate and CoA biosynthesis 0.0003 0.0039
  Glycolysis / Gluconeogenesis 0.0009 0.0083
  Taurine and hypotaurine metabolism 0.001 0.0082
  Biosynthesis of secondary metabolites 0.001 0.0079
  D-Glutamine and D-glutamate metabolism 0.001 0.0099
  beta-Alanine metabolism 0.002 0.0122
  Citrate cycle (TCA cycle) 0.002 0.0144
  Toluene degradation 0.004 0.0204
  Chloroalkane and chloroalkene degradation 0.004 0.0212
  Naphthalene degradation 0.004 0.0198
  Lipoic acid metabolism 0.004 0.0187
  Methane metabolism 0.006 0.0266
  Butanoate metabolism 0.009 0.0371
     Predicted metabolites from CLR  
   Predicted metabolites Pvalue Overlap with hits  2-Dehydropantoate 2e-05 0.0000
  beta-Alanine 2e-05 0.0000
  (R)-Pantoate 2e-05 1.0000
  5-[(5-phospho-1-deoxyribulos-1-ylamino)methylideneamino]-1-(5-phosphoribosyl)imidazole-4-carboxamide 2e-05 0.0000
  silver 8e-05 0.0000
  Acetyl phosphate 0.0002 0.0000
  Cu+ 0.0002 0.0000
  [4Fe-4S] iron-sulfur cluster 0.0006 0.0000
  [2Fe-2S] iron-sulfur cluster 0.001 0.0000
  Selenate 0.001 0.0000
  selenite 0.001 0.0000
  2-Demethylmenaquinone 8 0.001 0.0000
  2-Demethylmenaquinol 8 0.001 0.0000
  Molybdate 0.001 0.0000
  Sulfate 0.003 0.0000
  Fumarate 0.005 0.0000
  L-Malate 0.008 0.0000
    
 
