# Supplementary material for: Genomewide landscape of gene–metabolome associations in Escherichia coli
Source: Mol Syst Biol. 2017 Jan 16;13(1):907. doi: 10.15252/msb.20167150 (PMC5293155; doi:10.15252/msb.20167150)
Supplement: Supplementary file 4 — Table EV3 [file MSB-13-907-s004.zip › details/data_ybeX.html]

 
 
 ybeX 
  ybeX - details 
 
 
  CLR  
   Gene_matching CLR_index  lomR 55.9
  yaiX 43.4
  yccW 26.6
  ybaL 22.8
  pphB 21.9
  ybgK 21.4
  ybcD 21.4
  ybhO 17.2
  yaiC 15.5
  yaaJ 15.2
  ybhG 15.0
  mhpB 15.0
  ybiV 14.3
  sgrR 13.3
  yaeJ 12.9
  ybeD 12.8
  ycaD 12.8
  yliG 12.0
  ycaC 11.7
  ybaE 11.0
  thiP 10.7
  ynaA 10.7
  yoeE 10.3
  essD 10.3
  yebK 10.2
  ppdC 10.0
  mazG 9.3
  ybhI 9.2
  yecP 9.2
  ybeM 9.0
  ygjK 8.7
  ycaJ 8.5
  yafJ 8.5
  stfR 8.4
  afuB 8.3
  yecO 8.1
  ycbY 8.0
  acnB 7.9
  yajC 7.7
  ybaV 7.4
  yahF 7.3
  ubiF 7.3
  ybiS 7.2
  yliF 7.1
  zwf 7.1
  yliE 6.9
  yahA 6.9
  ydfT 6.5
  ybhS 6.3
  ybhA 5.9
  yedL 5.8
  ycaL 5.2
  yedF 4.3
  mprA 4.1
  dcuA 4.0
  yfaY 4.0
  yfbQ 3.8
  yfjU 3.8
  ybaX 3.7
  ydjJ 3.4
  ybiP 3.1
     Differential ions  
   id name formula mz mod AUC Z-score Z-score AUC Weighted   C01468  p-Cresol C7H8O 125.0609 +OH(-) 0.954 27.035 25.791
   C01585  Hexanoate (n-C6:0) C6H12O2 137.0599 .H/Na-H(+) 0.710 18.267 12.961
   C12622  cis-3-(3-carboxyethenyl)-3,5-cyclohexadiene-1,2-diol C9H10O4 137.0599 -CO2-H(+) 0.608 18.267 11.107
   C16488  fructoselysine C12H24N2O7 307.1519 -H(+) 0.755 14.295 10.786
   psicoselysine  psicoselysine C12H24N2O7 307.1519 -H(+) 0.633 14.295 9.051
   C01571  Decanoate (n-C10:0) C10H20O2 193.1196 .H/Na-H(+) 0.735 9.641 7.084
   C16488  fructoselysine C12H24N2O7 263.1597 -CO2-H(+) 0.659 10.706 7.052
   C00141  3-Methyl-2-oxobutanoate C5H8O3 137.0255 .H/Na-H(+) 0.808 7.004 5.659
   Octadecanoyl-phosphate (n-C18:1)  Octadecanoyl-phosphate (n-C18:1) C18H35O5P 383.2028 .H/Na-H(+) 0.659 8.317 5.478
   C01092  8-Amino-7-oxononanoate C9H17NO3 169.0860 -NH3-H(+) 0.680 7.931 5.397
   C00681  1-dodecanoyl-sn-glycerol 3-phosphate C15H31O7P1 355.1746 [+2]-H(+) 0.883 5.105 4.508
   C06840  butanesulfonate C4H10O3S 137.0255 -H(+) 0.635 7.004 4.447
   C00680  meso-2,6-Diaminoheptanedioate C7H14N2O4 207.1004 +OH(-) 0.702 6.252 4.387
   C00860  L-Histidinol C6H11N3O 140.0788 -H(+) 0.904 4.845 4.378
   C00681  1-tetradecanoyl-sn-glycerol 3-phosphate C17H35O7P1 399.2121 +OH(-) 0.870 4.911 4.270
   C03974  2-tetradecanoyl-sn-glycerol 3-phosphate C17H35O7P1 399.2121 +OH(-) 0.806 4.911 3.961
   C15767  gamma-glutamyl-gamma aminobutyric acid C9H16O5N2 213.0867 -H2O-H(+) 0.748 5.246 3.924
   C03415  N2-Succinyl-L-ornithine C9H16N2O5 213.0867 -H2O-H(+) 0.735 5.246 3.853
   C03974  2-dodecanoyl-sn-glycerol 3-phosphate C15H31O7P1 355.1746 [+2]-H(+) 0.730 5.105 3.729
   Hexadecanoyl-phosphate (n-C16:0)  Hexadecanoyl-phosphate (n-C16:0) C16H33O5P 357.1883 .H/Na-H(+) 0.614 5.961 3.657
   C01468  p-Cresol C7H8O 107.0501 -H(+) 0.883 4.006 3.537
   C02637  3-Dehydroshikimate C7H8O5 127.0394 -CO2-H(+) 0.792 4.334 3.431
   Octadecanoyl-phosphate (n-C18:0)  Octadecanoyl-phosphate (n-C18:0) C18H37O5P 364.2460 [+1]-H(+) 0.607 5.586 3.393
   C00530  Hydroquinone C6H6O2 127.0394 +OH(-) 0.751 4.334 3.256
   C01602  Ornithine C5H12N2O2 153.0656 .H/Na-H(+) 0.604 5.199 3.142
   C01279  4-Amino-5-hydroxymethyl-2-methylpyrimidine C6H9N3O 138.0662 -H(+) 0.816 3.805 3.104
   C00681  1-dodecanoyl-sn-glycerol 3-phosphate C15H31O7P1 353.1686 -H(+) 0.739 4.177 3.089
   C11457  3-(3-hydroxy-phenyl)propionate C9H10O3 183.0645 +OH(-) 0.726 4.185 3.039
   L-alanine-L-glutamate  L-alanine-L-glutamate C8H14N2O5 235.0970 +OH(-) 0.639 4.635 2.960
   C00246  Butyrate (n-C4:0) C4H8O2 69.0359 -H2O-H(+) 0.656 4.371 2.867
   C06006  (S)-2-Aceto-2-hydroxybutanoate C6H10O4 127.0394 -H2O-H(+) 0.649 4.334 2.811
   C03406  N(omega)-(L-Arginino)succinate C10H18N4O6 291.1196 [+2]-H(+) 0.790 3.551 2.807
   C01909  Dethiobiotin C10H18N2O3 195.1153 -H2O-H(+) 0.706 3.820 2.697
   C04556  4-Amino-2-methyl-5-phosphomethylpyrimidine C6H10N3O4P 138.0662 -HPO3-H(+) 0.696 3.805 2.649
   C00123  L-Leucine C6H13NO2 113.0607 -NH3-H(+) 0.653 4.050 2.645
   C00407  L-Isoleucine C6H13NO2 113.0607 -NH3-H(+) 0.643 4.050 2.605
   C00437  N2-Acetyl-L-ornithine C7H14N2O3 191.1055 +OH(-) 0.745 3.475 2.591
   psicoselysine  psicoselysine C12H24N2O7 289.1415 -H2O-H(+) 0.625 3.518 2.197
   C16488  fructoselysine C12H24N2O7 289.1415 -H2O-H(+) 0.594 3.518 0.000
   cis-3-(3-carboxyethyl)-3,5-cyclohexadiene-1,2-diol  cis-3-(3-carboxyethyl)-3,5-cyclohexadiene-1,2-diol C9H12O4 139.0756 -CO2-H(+) 0.593 14.694 0.000
   C00246  Butyrate (n-C4:0) C4H8O2 87.0460 -H(+) 0.549 4.628 0.000
   C00993  D-Alanyl-D-alanine C6H12N2O3 177.0902 +OH(-) 0.548 6.122 0.000
   C11457  3-(3-hydroxy-phenyl)propionate C9H10O3 121.0655 -CO2-H(+) 0.532 3.755 0.000
   ferroxamine minus Fe(3)  ferroxamine minus Fe(3) C25H48N6O8 561.3595 .H(+) 0.532 4.257 0.000
   C00047  L-Lysine C6H14N2O2 167.0783 .H/Na-H(+) 0.529 4.273 0.000
   C04044  3-(2,3-Dihydroxyphenyl)propanoate C9H10O4 137.0599 -CO2-H(+) 0.523 18.267 0.000
   C00989  gamma-hydroxybutyrate C4H8O3 104.0435 [+1]-H(+) 0.515 3.756 0.000
   C01602  Ornithine C5H12N2O2 149.0953 +OH(-) 0.503 11.079 0.000
   C03974  2-dodecanoyl-sn-glycerol 3-phosphate C15H31O7P1 353.1686 -H(+) 0.498 4.177 0.000
   cis-3-(3-carboxyethyl)-3,5-cyclohexadiene-1,2-diol  cis-3-(3-carboxyethyl)-3,5-cyclohexadiene-1,2-diol C9H12O4 183.0645 -H(+) 0.494 4.185 0.000
   psicoselysine  psicoselysine C12H24N2O7 263.1597 -CO2-H(+) 0.494 10.706 0.000
   L-alanine-D-glutamate  L-alanine-D-glutamate C8H14N2O5 235.0970 +OH(-) 0.478 4.635 0.000
   tetradecenoate (n-C14:1)  tetradecenoate (n-C14:1) C14H26O2 247.1636 .H/Na-H(+) 0.469 4.398 0.000
   C00989  gamma-hydroxybutyrate C4H8O3 103.0398 -H(+) 0.464 4.181 0.000
   C00288  Bicarbonate CH2O3 60.9942 -H(+) 0.447 7.789 0.000
   C00156  4-Hydroxybenzoate C7H6O3 137.0255 -H(+) 0.405 7.004 0.000
   C00534  Pyridoxamine C8H12N2O2 167.0783 -H(+) 0.405 4.273 0.000
   C00966  2-Dehydropantoate C6H10O4 127.0394 -H2O-H(+) 0.391 4.334 0.000
   C00601  Phenylacetaldehyde C8H8O 137.0599 +OH(-) 0.380 18.267 0.000
   C00666  LL-2,6-Diaminoheptanedioate C7H14N2O4 207.1004 +OH(-) 0.000 6.252 0.000
   C04732  4-(1-D-Ribitylamino)-5-aminouracil C9H16N4O6 295.1178 [+2]+OH(-) 0.000 4.281 0.000
     KEGG pathway by CLR  
   Pathway_ion pvalue_ion qvalue_ion  Arachidonic acid metabolism 0 0.0000
  Limonene and pinene degradation 3e-12 0.0000
  Biosynthesis of secondary metabolites 2e-10 0.0000
  Toluene degradation 2e-08 0.0000
  Xylene degradation 2e-06 0.0000
  alpha-Linolenic acid metabolism 7e-05 0.0011
  Microbial metabolism in diverse environments 0.0002 0.0020
  Butanoate metabolism 0.0002 0.0020
  Ethylbenzene degradation 0.002 0.0156
  Bisphenol degradation 0.004 0.0350
  Nitrotoluene degradation 0.009 0.0723
     COG enrichment  
   Pathway_MS pvalue_MS qvalue_MS  Protein export 0.005 0.5238
     Predicted metabolites from CLR  
   Predicted metabolites Pvalue Overlap with hits  D-Glucose 6-phosphate 0.001 0.0000
  L-Alanine 0.002 0.0000
    
 
