# Supplementary material for: Genomewide landscape of gene–metabolome associations in Escherichia coli
Source: Mol Syst Biol. 2017 Jan 16;13(1):907. doi: 10.15252/msb.20167150 (PMC5293155; doi:10.15252/msb.20167150)
Supplement: Supplementary file 4 — Table EV3 [file MSB-13-907-s004.zip › details/data_ybfA.html]

 
 
 ybfA 
  ybfA - details 
 
 
  CLR  
   Gene_matching CLR_index  ydfW 10.4
  gpp 10.0
  ypfH 9.6
  ybbP 7.7
  glxK 7.6
  ygcW 7.4
  yfeH 7.3
  ybbN 7.3
  wcaC 7.2
  ycbG 7.1
  oxyR 6.9
  dhaR 6.9
  kdpB 6.9
  ybiM 6.9
  yieP 6.8
  bglJ 6.7
  proY 6.6
  lipB 6.5
  rtcA 6.5
  yobA 6.5
  yrhA 6.4
  yciB 6.4
  yaaY 6.0
  yhjX 5.9
  ymgH 5.9
  cysB 5.9
  yicI 5.8
  ycdU 5.8
  ypfJ 5.8
  prfC 5.8
  ybfN 5.7
  yeeP 5.7
  rzoD 5.7
  bioH 5.7
  yhjC 5.7
  yebA 5.6
  hokC 5.6
  intD 5.6
  recX 5.6
  yjjM 5.5
  ydhM 5.5
  tldD 5.4
  ynfC 5.4
  yjfN 5.4
  amtB 5.4
  ybdH 5.4
  ynfD 5.3
  cld 5.2
  aidB 5.2
  rpsO 5.2
  ubiG 5.2
  csiE 5.2
  yohG 5.2
  dgoT 5.2
  ybjG 5.1
  yhbY 5.1
  yeeY 5.1
  baeR 5.1
  emtA 5.1
  alpA 5.0
  gntU 5.0
  torS 5.0
  yghO 5.0
  yidR 5.0
  ycbW 5.0
  rpsT 4.9
  dkgB 4.8
  yicJ 4.8
  ychM 4.8
  yjfP 4.8
  dcuC 4.8
  tfaS 4.8
  yneF 4.8
  ycdH 4.7
  ivbL 4.7
  poxA 4.6
  yceG 4.6
  intE 4.6
  ulaG 4.5
  truA 4.5
  trmA 4.5
  elaD 4.5
  djlC 4.5
  yjgF 4.4
  glnD 4.4
  rpsU 4.3
  aaeR 4.3
  hdfR 4.3
  nuoC 4.3
  ssuE 4.3
  azoR 4.2
  tnaB 4.2
  ypeB 4.2
  dmsD 4.2
  cspE 4.1
  yqjF 4.1
  gldA 4.1
  yfeS 4.1
  ybfE 4.1
  gcvH 4.0
  ycgJ 4.0
  marC 4.0
  yjiA 4.0
  ydbJ 4.0
  ybjS 4.0
  aroK 4.0
  pyrF 4.0
  glcB 3.9
  ascG 3.9
  yedJ 3.9
  eutP 3.9
  abrB 3.8
  fucA 3.8
  ycdT 3.7
  yncH 3.7
  yibK 3.7
  yedQ 3.7
  yedW 3.7
  leuB 3.7
  yliB 3.7
  apaH 3.6
  potG 3.6
  rffE 3.6
  yjgB 3.6
  yjiP 3.6
  hybG 3.5
  pyrE 3.5
  mobB 3.5
  ydcH 3.5
  ykgE 3.5
  ygcL 3.4
  hyfJ 3.4
  norR 3.4
  ddlA 3.4
  aphA 3.4
  yfjP 3.4
  speB 3.4
  fucO 3.4
  yiiF 3.3
  yddM 3.3
  modE 3.3
  prmB 3.3
  bolA 3.3
  usg 3.2
  mcrB 3.2
  hipB 3.2
  kgtP 3.2
  ulaE 3.2
  idnK 3.2
  yphC 3.2
  wcaI 3.1
  ycgH 3.1
  mcrC 3.1
  yncM 3.1
  yqhC 3.1
  narY 3.1
  yjiK 3.1
  recC 3.1
  yjdI 3.1
  yjhT 3.1
  ykfC 3.1
  aaeX 3.0
  yhiF 3.0
  yiiM 3.0
  mutY 3.0
  ygeP 3.0
     Differential ions  
   id name formula mz mod AUC Z-score Z-score AUC Weighted   C00064  L-Glutamine C5H10N2O3 147.0763 .H(+) 0.516 3.996 0.000
   C11434  2-C-methyl-D-erythritol 4-phosphate C5H13O7P 252.9869 .H/K-H(+) 0.000 3.793 0.000
     KEGG pathway by CLR  
   Pathway_ion pvalue_ion qvalue_ion  D-Glutamine and D-glutamate metabolism 2e-08 0.0000
  Pyrimidine metabolism 4e-06 0.0002
  Nitrogen metabolism 3e-05 0.0009
  Two-component system 0.0001 0.0028
  Alanine, aspartate and glutamate metabolism 0.0002 0.0033
  Aminoacyl-tRNA biosynthesis 0.0003 0.0043
  Histidine metabolism 0.0004 0.0052
  ABC transporters 0.001 0.0137
  Purine metabolism 0.001 0.0139
  Arginine and proline metabolism 0.002 0.0200
     COG enrichment  
   Pathway_MS pvalue_MS qvalue_MS  Riboflavin metabolism 0.0003 0.0321
  Ribosome 0.003 0.1266
  D-Alanine metabolism 0.006 0.1858
  Lipoic acid metabolism 0.006 0.1393
     Predicted metabolites from CLR  
   Predicted metabolites Pvalue Overlap with hits  Orotidine 5'-phosphate 0 0.0000
  L-Lactaldehyde 4e-05 0.0000
  Acetol 0.0001 0.0000
  Methylglyoxal 0.0003 0.0000
  D-Gluconate 0.0005 0.0000
  L-Malate 0.005 0.0000
  Putrescine 0.01 0.0000
    
 
