# Supplementary material for: Genomewide landscape of gene–metabolome associations in Escherichia coli
Source: Mol Syst Biol. 2017 Jan 16;13(1):907. doi: 10.15252/msb.20167150 (PMC5293155; doi:10.15252/msb.20167150)
Supplement: Supplementary file 4 — Table EV3 [file MSB-13-907-s004.zip › details/data_ybfD.html]

 
 
 ybfD 
  ybfD - details 
 
 
  CLR  
   Gene_matching CLR_index  nadR 9.2
  ygeI 8.4
  ygeQ 8.2
  ypjA 8.2
  yhhI 8.0
  pbl 7.8
  glyS 7.7
  mutS 7.6
  xerD 7.5
  ygbF 7.2
  yhdP 6.9
  thrL 6.9
  mutH 6.9
  ygeN 6.6
  rhsA 6.6
  yphB 6.6
  ygdB 6.5
  yeaN 6.4
  rhsC 6.4
  gidB 6.4
  modA 6.3
  yidF 6.2
  ygaY 6.2
  gspD 6.2
  deaD 6.2
  gadE 6.1
  ydcC 6.1
  rhsD 6.1
  gudD 6.1
  fucU 6.1
  ampH 6.0
  ygfI 6.0
  yqaC 5.9
  yagX 5.9
  sseA 5.8
  envC 5.8
  nlpA 5.7
  glpR 5.7
  ygcK 5.6
  ygcG 5.5
  ompG 5.5
  narU 5.4
  hyfI 5.4
  rtcB 5.4
  ygcU 5.3
  ygcR 5.3
  glcG 5.3
  slyA 5.3
  yqeH 5.3
  metI 5.2
  yedY 5.2
  ylcG 5.2
  yebS 5.2
  mltC 5.2
  ygcQ 5.0
  clcB 5.0
  ydhB 5.0
  yfaZ 5.0
  gutQ 5.0
  ypjC 5.0
  argK 5.0
  dcp 5.0
  fixA 4.9
  yfjS 4.9
  yeiP 4.8
  yfjD 4.8
  ycjD 4.7
  yccK 4.7
  ydjQ 4.7
  phnJ 4.7
  ygeK 4.7
  yohO 4.7
  rpoS 4.7
  yecC 4.6
  yfjZ 4.6
  yagL 4.6
  yfbJ 4.6
  recN 4.5
  nudD 4.5
  prpE 4.5
  yhdA 4.5
  yebU 4.5
  phnE 4.5
  rspB 4.5
  yidL 4.5
  hinT 4.5
  ppdB 4.5
  tolC 4.4
  yaiL 4.4
  ygcE 4.4
  vacJ 4.4
  pinH 4.4
  ybfO 4.4
  apt 4.3
  pbpC 4.3
  recO 4.3
  kptA 4.3
  yeeL 4.3
  dacD 4.3
  yhbO 4.2
  ynjI 4.2
  ygaT 4.2
  napD 4.2
  mdtI 4.2
  yehR 4.2
  yabP 4.2
  marR 4.2
  yehU 4.1
  yjiJ 4.1
  yhcC 4.1
  ycfJ 4.1
  yehT 4.1
  rsxC 4.1
  yhdZ 4.1
  rhsE 4.0
  phnO 4.0
  malT 4.0
  relE 4.0
  malP 4.0
  yhfZ 4.0
  sfmH 4.0
  ung 4.0
  fdoH 4.0
  hisQ 4.0
  yqiG 4.0
  yifL 3.9
  ubiH 3.9
  kduD 3.9
  yigZ 3.9
  yqeJ 3.9
  yhjG 3.9
  acrR 3.8
  ygfM 3.8
  yajL 3.8
  yecM 3.8
  ycaQ 3.8
  yobF 3.8
  fimG 3.8
  yibF 3.8
  kil 3.8
  wbbJ 3.8
  trmH 3.8
  gatB 3.8
  arpB 3.8
  aqpZ 3.8
  ydeR 3.7
  yggS 3.7
  hycB 3.7
  yagH 3.7
  gatR 3.7
  clpB 3.7
  yehL 3.7
  phnL 3.7
  ybbW 3.7
  yfbT 3.7
  yehD 3.6
  yoaC 3.6
  yeaH 3.6
  thiD 3.6
  yeaP 3.6
  ydeN 3.6
  ymfN 3.6
  yncG 3.6
  yegH 3.6
  yecN 3.6
  citC 3.6
  fkpB 3.6
  ydeH 3.5
  yfeR 3.5
  yecT 3.5
  nikB 3.5
  yliD 3.5
  flhD 3.5
  dinG 3.5
  fucK 3.5
  ydeO 3.5
  ybbC 3.5
  yjaA 3.5
  puuB 3.5
  hyfC 3.4
  mviM 3.4
  yfcQ 3.4
  yqgC 3.4
  sgbE 3.4
  ycdR 3.4
  yecD 3.4
  cvrA 3.4
  lit 3.4
  yraN 3.4
  sufB 3.4
  wbbI 3.4
  dsbB 3.4
  ydeV 3.4
  yodB 3.4
  bglH 3.3
  agaI 3.3
  yeeT 3.3
  ychE 3.3
  phnH 3.3
  gabT 3.3
  rpiA 3.3
  cspH 3.3
  ynjB 3.3
  ynjC 3.2
  kgtP 3.2
  caiB 3.2
  yajI 3.2
  nikE 3.2
  ydhZ 3.2
  menB 3.2
  lrhA 3.2
  yfeW 3.2
  yqeK 3.1
  ilvC 3.1
  yeiU 3.1
  udp 3.1
  fdnH 3.1
  livF 3.1
  rfaC 3.1
  yjiW 3.1
  fbaB 3.1
  alsA 3.1
  rfaB 3.0
  yfbU 3.0
  yebB 3.0
  yahN 3.0
  mngR 3.0
  ycjZ 3.0
  yegP 3.0
  dnaG 3.0
  fucI 3.0
  ydeP 3.0
  yfjJ 3.0
  hisB 3.0
  ykiA 3.0
  trmC 3.0
  ytfA 3.0
  yfdL 3.0
     Differential ions  
   id name formula mz mod AUC Z-score Z-score AUC Weighted   C04114  crotonobetaine C7H13NO2 100.1124 -CO2.H(+) 0.663 4.446 2.949
     KEGG pathway by CLR  
   Pathway_ion pvalue_ion qvalue_ion  Caprolactam degradation 0.0002 0.0206
  Biosynthesis of secondary metabolites 0.0009 0.0479
     COG enrichment  
none  Predicted metabolites from CLR  
   Predicted metabolites Pvalue Overlap with hits  L-fuculose 0 0.0000
  D-Ribulose 5-phosphate 0.0008 0.0000
  nickel 0.003 0.0000
    
 
