# Supplementary material for: Genomewide landscape of gene–metabolome associations in Escherichia coli
Source: Mol Syst Biol. 2017 Jan 16;13(1):907. doi: 10.15252/msb.20167150 (PMC5293155; doi:10.15252/msb.20167150)
Supplement: Supplementary file 4 — Table EV3 [file MSB-13-907-s004.zip › details/data_ybgA.html]

 
 
 ybgA 
  ybgA - details 
 
 
  CLR  
   Gene_matching CLR_index  yahC 10.5
  crcB 10.2
  yjeK 9.8
  cysH 9.6
  ygcN 8.9
  cchB 8.7
  holD 8.6
  glmM 8.4
  murP 8.1
  pdxJ 7.8
  ypdG 7.7
  metE 7.4
  gadX 7.2
  gspO 7.2
  cof 7.1
  rpoN 7.1
  rsgA 7.0
  panC 7.0
  ygeR 6.7
  ilvA 6.7
  ypjL 6.7
  ygcL 6.7
  yeiQ 6.7
  ilvB 6.7
  spr 6.7
  yajO 6.7
  yceD 6.5
  prpD 6.5
  citG 6.5
  rnb 6.3
  aroH 6.3
  yafU 6.3
  yfgB 6.3
  uidA 6.2
  coaE 6.1
  ygjR 6.1
  yhhW 6.0
  yebT 6.0
  yzgL 5.9
  betT 5.9
  cchA 5.9
  etp 5.8
  yahK 5.7
  yfhL 5.7
  yjdF 5.6
  ilvE 5.6
  pdxA 5.5
  yojI 5.5
  rof 5.4
  purC 5.4
  yfaQ 5.3
  ydaS 5.3
  puuD 5.3
  yaaA 5.2
  ycfH 5.2
  bcp 5.2
  metL 5.2
  mrcA 5.1
  bcsC 5.1
  metH 5.1
  yfiE 5.0
  yfjR 5.0
  yhbJ 5.0
  ycjW 5.0
  trmU 4.9
  mrcB 4.9
  yifK 4.8
  ascF 4.8
  yjfJ 4.8
  mak 4.8
  ydiZ 4.8
  trkG 4.8
  yqjB 4.8
  flk 4.7
  cmtA 4.7
  abrB 4.7
  ysgA 4.6
  yedP 4.6
  cysN 4.6
  yjeO 4.6
  mdtK 4.6
  yigF 4.6
  ymcA 4.5
  chbC 4.5
  nanA 4.4
  ycgJ 4.4
  flgK 4.4
  fliQ 4.4
  moaB 4.4
  cysJ 4.4
  rbsD 4.4
  yfcD 4.4
  purH 4.3
  yibL 4.3
  yfaW 4.3
  rbfA 4.3
  yjeP 4.3
  ptsP 4.3
  ubiG 4.3
  rfaL 4.2
  frlR 4.2
  holE 4.2
  nudB 4.2
  cysZ 4.2
  pgm 4.2
  miaA 4.2
  metB 4.2
  hyfB 4.2
  dadX 4.1
  prfB 4.1
  ygfO 4.1
  yqjF 4.1
  yfcV 4.1
  yfcY 4.1
  yhdV 4.1
  yehA 4.1
  mcrB 4.1
  metC 4.0
  ggt 4.0
  yphD 4.0
  yneG 3.9
  ccmH 3.9
  yfcP 3.9
  ygeX 3.9
  lysC 3.9
  cysI 3.9
  yfcO 3.9
  yajG 3.9
  ybdR 3.8
  yjgH 3.8
  yieH 3.8
  uspE 3.8
  ybgC 3.8
  sdaC 3.8
  yedK 3.8
  agaD 3.8
  dnaQ 3.8
  hyaF 3.8
  srlB 3.8
  yddL 3.7
  moeB 3.7
  fis 3.7
  malI 3.7
  yohM 3.7
  hisQ 3.7
  yecI 3.7
  yeeP 3.7
  thiQ 3.7
  ydhJ 3.7
  envZ 3.7
  rep 3.7
  yhcO 3.7
  ykfA 3.6
  yadN 3.6
  speG 3.6
  argH 3.6
  ymgA 3.6
  fruK 3.6
  ompR 3.6
  nupC 3.6
  yjeH 3.6
  frvA 3.6
  dacB 3.5
  pfkA 3.5
  rng 3.5
  ndk 3.5
  ybjD 3.5
  yjdL 3.5
  inaA 3.5
  yaaW 3.5
  cpxP 3.5
  fucP 3.5
  yeeS 3.5
  paaY 3.4
  yqfA 3.4
  tatD 3.4
  leuD 3.4
  exuT 3.4
  ppx 3.4
  mlrA 3.4
  ygiQ 3.4
  dsrB 3.4
  sfmC 3.4
  proX 3.4
  mngB 3.4
  ychF 3.4
  trxA 3.4
  yieI 3.3
  ninE 3.3
  pepB 3.3
  glnD 3.3
  yjfK 3.3
  galF 3.3
  rfaH 3.3
  edd 3.3
  exbB 3.3
  malF 3.3
  ycdQ 3.3
  hsdS 3.3
  yecC 3.3
  rffC 3.3
  cmk 3.3
  ypfN 3.3
  yjbJ 3.2
  asnA 3.2
  treC 3.2
  ygiC 3.2
  yraP 3.2
  yjhC 3.2
  rpsO 3.2
  yjfL 3.2
  pheA 3.2
  yhiJ 3.2
  metQ 3.2
  ygfG 3.2
  ivy 3.2
  yrbC 3.2
  yjhG 3.2
  pnp 3.2
  yaiO 3.2
  yhhM 3.2
  yhjR 3.2
  yjjJ 3.2
  eutL 3.2
  kbl 3.2
  yrdB 3.2
  ilvM 3.1
  yliI 3.1
  ycgK 3.1
  cbpA 3.1
  speA 3.1
  yjiH 3.1
  yacH 3.1
  mutL 3.1
  ybeQ 3.1
  yhjB 3.1
  hemX 3.1
  sbmC 3.1
  yhbE 3.0
  damX 3.0
  glcE 3.0
  yafZ 3.0
  uraA 3.0
  norR 3.0
  nrfG 3.0
  ybaV 3.0
  rihA 3.0
  hyfH 3.0
  bax 3.0
  crp 3.0
     Differential ions  
   id name formula mz mod AUC Z-score Z-score AUC Weighted   C16238  lipoyl-AMP C18H26N5O8PS2 572.0458 .H/K-H(+) 0.954 -3.544 -3.381
     KEGG pathway by CLR  
none  COG enrichment  
   Pathway_MS pvalue_MS qvalue_MS  Chlorocyclohexane and chlorobenzene degradation 0 0.0000
  Fluorobenzoate degradation 0 0.0000
  Sulfur metabolism 6e-06 0.0001
  Selenoamino acid metabolism 0.0004 0.0049
  Pantothenate and CoA biosynthesis 0.0005 0.0059
  Valine, leucine and isoleucine biosynthesis 0.0007 0.0072
  Biosynthesis of secondary metabolites 0.0008 0.0066
  Cysteine and methionine metabolism 0.001 0.0108
  Fructose and mannose metabolism 0.002 0.0109
  C5-Branched dibasic acid metabolism 0.002 0.0112
  DNA replication 0.002 0.0103
  Mismatch repair 0.002 0.0110
  Peptidoglycan biosynthesis 0.004 0.0207
  Arachidonic acid metabolism 0.004 0.0199
  Cyanoamino acid metabolism 0.005 0.0210
  Purine metabolism 0.005 0.0198
  Phosphotransferase system (PTS) 0.006 0.0235
  Pyrimidine metabolism 0.008 0.0289
     Predicted metabolites from CLR  
   Predicted metabolites Pvalue Overlap with hits  Dephospho-CoA 0 0.0000
  4-Phospho-L-aspartate 0.0003 0.0000
  5-Methyltetrahydrofolate 0.0003 0.0000
  L-Cystathionine 0.0003 0.0000
  CDP 0.0004 0.0000
  Hydrogen sulfide 0.0005 0.0000
  L-Homocysteine 0.0005 0.0000
  Pyridoxine 5'-phosphate 0.001 0.0000
  Phenylpyruvate 0.001 0.0000
  O-Phospho-4-hydroxy-L-threonine 0.001 0.0000
  Reduced riboflavin 0.002 0.0000
  Riboflavin 0.002 0.0000
  dCDP 0.003 0.0000
  Sulfite 0.004 0.0000
  Undecaprenyl-diphospho-N-acetylmuramoyl-(N-acetylglucosamine)-L-ala-D-glu-meso-2,6-diaminopimeloyl-D-ala-D-ala 0.005 0.0000
  GDP 0.005 0.0000
  L-Methionine 0.005 0.0000
  5,6,7,8-Tetrahydrofolate 0.006 0.0000
  dATP 0.008 0.0000
    
 
