# Supplementary material for: Genomewide landscape of gene–metabolome associations in Escherichia coli
Source: Mol Syst Biol. 2017 Jan 16;13(1):907. doi: 10.15252/msb.20167150 (PMC5293155; doi:10.15252/msb.20167150)
Supplement: Supplementary file 4 — Table EV3 [file MSB-13-907-s004.zip › details/data_ybgD.html]

 
 
 ybgD 
  ybgD - details 
 
 
  CLR  
   Gene_matching CLR_index  yidK 13.7
  rmuC 13.1
  yagM 12.7
  mtlD 12.5
  yjdL 12.5
  katG 12.2
  gcvR 12.1
  treF 12.0
  qor 11.3
  glpE 11.0
  yhbP 10.3
  yidG 10.1
  sucB 10.1
  rraA 9.9
  tktA 9.9
  ydfU 9.6
  ydfI 9.4
  kbl 9.0
  yeaG 9.0
  yjeB 8.9
  mprA 8.8
  caiT 8.8
  speF 8.7
  flxA 8.6
  cfa 8.5
  kdpD 8.3
  rbsA 8.2
  seqA 8.2
  yggJ 8.2
  rfaQ 7.7
  yghR 7.7
  yicR 7.5
  fbp 7.4
  ulaR 7.4
  ccmD 7.2
  malS 7.2
  glnH 7.1
  recT 7.1
  ytfL 6.9
  yciV 6.9
  pfs 6.6
  yihR 6.5
  rssA 6.4
  nfsA 6.4
  metF 6.4
  lsrD 6.3
  thrA 6.2
  mfd 6.2
  nrfD 6.2
  yqjI 6.1
  fdnG 6.0
  potD 6.0
  ygaC 6.0
  ydjF 5.9
  yobG 5.9
  pinQ 5.9
  basS 5.8
  yneK 5.7
  aslA 5.7
  yjaG 5.7
  cysD 5.6
  cmk 5.6
  tam 5.6
  yjgH 5.6
  cspA 5.5
  yodC 5.5
  ybeT 5.5
  yncN 5.4
  nrfA 5.4
  serA 5.3
  trxA 5.3
  mobA 5.3
  uxaB 5.3
  polA 5.2
  yqgE 5.2
  hyaB 5.1
  yhiI 5.0
  glvB 5.0
  ybaZ 4.9
  gspJ 4.9
  nfrA 4.9
  argB 4.9
  cysQ 4.8
  ydbC 4.8
  setA 4.8
  csgB 4.8
  pta 4.8
  yibQ 4.7
  nadC 4.7
  idnD 4.7
  fieF 4.7
  tfaQ 4.7
  yfgC 4.6
  pyrL 4.6
  moaC 4.6
  fdhF 4.4
  cysJ 4.4
  ydeJ 4.4
  ygeW 4.4
  yebK 4.4
  thiE 4.4
  bioA 4.3
  ytfQ 4.3
  menC 4.3
  nrdD 4.3
  hyaA 4.3
  cstA 4.3
  yhaK 4.2
  hemF 4.2
  araG 4.2
  feaR 4.2
  xdhD 4.1
  rph 4.1
  msrA 4.1
  yfgL 4.1
  ade 4.0
  rmf 4.0
  cusR 4.0
  ybcW 4.0
  ybbS 4.0
  btuF 4.0
  rhaT 3.9
  hycG 3.9
  rpmG 3.8
  tyrB 3.8
  rfbB 3.8
  lpxM 3.7
  otsB 3.7
  dsbC 3.7
  nrfC 3.7
  hdhA 3.7
  cysN 3.7
  phnG 3.6
  bglA 3.6
  yjfZ 3.6
  gshA 3.6
  aspC 3.6
  entC 3.5
  lldR 3.5
  fliC 3.5
  yddJ 3.5
  yqiK 3.5
  ycbY 3.5
  xylR 3.5
  yddG 3.5
  lpcA 3.4
  bglX 3.4
  coaA 3.4
  yjtD 3.4
  chbG 3.4
  ygbL 3.4
  glnD 3.3
  yfdO 3.3
  ycfN 3.3
  tus 3.3
  glnG 3.3
  serB 3.3
  cbrC 3.3
  yadG 3.2
  hyaE 3.2
  ydfV 3.2
  citD 3.2
  lipB 3.2
  ecnA 3.2
  yqjD 3.2
  sfsB 3.2
  ydaV 3.2
  proV 3.2
  upp 3.1
  ypdD 3.1
  ybiS 3.1
  ugpQ 3.1
  eutH 3.1
  yoaE 3.1
  fadH 3.1
  secG 3.1
  endA 3.1
  atoS 3.1
  ycbR 3.1
  ansA 3.0
  kdsC 3.0
  ychJ 3.0
  dcm 3.0
  yjjU 3.0
  argG 3.0
  nanE 3.0
  gspC 3.0
  yhhP 3.0
  allR 3.0
  cheB 3.0
  yjbO 3.0
     Differential ions  
   id name formula mz mod AUC Z-score Z-score AUC Weighted   C00052  UDPgalactose C15H24N2O17P2 587.0279 .H/Na-H(+) 0.760 4.520 3.434
   C00624  N-Acetyl-L-glutamate C7H11NO5 144.0662 -CO2-H(+) 0.744 4.591 3.415
   C03733  UDP-D-galacto-1,4-furanose C15H24N2O17P2 587.0279 .H/Na-H(+) 0.718 4.520 3.244
   C15996  7-cyano-7-carbaguanine C7H5N5O 309.9669 .H2PO4K-H(+) 0.735 4.307 3.164
   C15996  7-cyano-7-carbaguanine C7H5N5O 407.9480 .(H2PO4)2KH-H(+) 0.672 4.528 3.043
   C00979  O-Acetyl-L-serine C5H9NO4 168.0271 .H/Na-H(+) 0.688 4.383 3.014
   C03733  UDP-D-galacto-1,4-furanose C15H24N2O17P2 566.0473 [+1]-H(+) 0.667 4.443 2.963
   C00979  O-Acetyl-L-serine C5H9NO4 266.0090 .H2PO4Na-H(+) 0.647 4.562 2.952
   C00052  UDPgalactose C15H24N2O17P2 706.9838 .HPO4Na2-H(+) 0.684 4.246 2.905
   C03172  S-Methyl-L-methionine C6H13NO2S 282.0214 .H2PO4Na-H(+) 0.697 3.921 2.735
   C03733  UDP-D-galacto-1,4-furanose C15H24N2O17P2 706.9838 .HPO4Na2-H(+) 0.629 4.246 2.671
   C03082  4-Phospho-L-aspartate C4H8NO7P 429.9324 .(H2PO4)2NaH-H(+) 0.714 3.693 2.639
   C00979  O-Acetyl-L-serine C5H9NO4 146.0457 -H(+) 0.662 3.594 2.380
   C00029  UDPglucose C15H24N2O17P2 566.0473 [+1]-H(+) 0.599 4.443 0.000
   C03082  4-Phospho-L-aspartate C4H8NO7P 445.9015 .(H2PO4)2KH-H(+) 0.596 3.936 0.000
   C00029  UDPglucose C15H24N2O17P2 587.0279 .H/Na-H(+) 0.581 4.520 0.000
   C00029  UDPglucose C15H24N2O17P2 565.0470 -H(+) 0.579 3.653 0.000
   C00979  O-Acetyl-L-serine C5H9NO4 385.9678 .(H2PO4Na)2-H(+) 0.562 3.729 0.000
   C00860  L-Histidinol C6H11N3O 282.0214 .HPO4Na2-H(+) 0.561 3.921 0.000
   C05973  2-Acyl-sn-glycero-3-phosphoethanolamine (n-C16:0) C21H44NO7P1 452.2770 -H(+) 0.554 3.596 0.000
   C00624  N-Acetyl-L-glutamate C7H11NO5 188.0561 -H(+) 0.545 4.126 0.000
   C03733  UDP-D-galacto-1,4-furanose C15H24N2O17P2 565.0470 -H(+) 0.532 3.653 0.000
   C00015  UDP C9H14N2O12P2 385.9678 -NH3-H(+) 0.492 3.729 0.000
   C00624  N-Acetyl-L-glutamate C7H11NO5 210.0381 .H/Na-H(+) 0.490 4.190 0.000
   C00979  O-Acetyl-L-serine C5H9NO4 287.9913 .HPO4Na2-H(+) 0.459 4.366 0.000
   C00029  UDPglucose C15H24N2O17P2 706.9838 .HPO4Na2-H(+) 0.455 4.246 0.000
   C00052  UDPgalactose C15H24N2O17P2 566.0473 [+1]-H(+) 0.405 4.443 0.000
   C15996  7-cyano-7-carbaguanine C7H5N5O 445.9015 .(H2PO4K)2-H(+) 0.371 3.936 0.000
   C00052  UDPgalactose C15H24N2O17P2 565.0470 -H(+) 0.368 3.653 0.000
   C00217  D-Glutamate C5H9NO4 146.0457 -H(+) 0.000 3.594 0.000
   C00217  D-Glutamate C5H9NO4 168.0271 .H/Na-H(+) 0.000 4.383 0.000
   C00217  D-Glutamate C5H9NO4 266.0090 .H2PO4Na-H(+) 0.000 4.562 0.000
   C00217  D-Glutamate C5H9NO4 287.9913 .HPO4Na2-H(+) 0.000 4.366 0.000
   C00217  D-Glutamate C5H9NO4 385.9678 .(H2PO4Na)2-H(+) 0.000 3.729 0.000
   C03340  2,3-Dihydrodipicolinate C7H7NO4 287.9913 .H2PO4Na-H(+) 0.000 4.366 0.000
   C03340  2,3-Dihydrodipicolinate C7H7NO4 309.9669 .HPO4Na2-H(+) 0.000 4.307 0.000
   C03340  2,3-Dihydrodipicolinate C7H7NO4 385.9678 .(H2PO4)2NaH-H(+) 0.000 3.729 0.000
   C03340  2,3-Dihydrodipicolinate C7H7NO4 407.9480 .(H2PO4Na)2-H(+) 0.000 4.528 0.000
   C03972  2,3,4,5-Tetrahydrodipicolinate C7H9NO4 188.0561 +OH(-) 0.000 4.126 0.000
     KEGG pathway by CLR  
   Pathway_ion pvalue_ion qvalue_ion  Lysine degradation 9e-07 0.0001
  Arginine and proline metabolism 2e-06 0.0001
  D-Glutamine and D-glutamate metabolism 5e-06 0.0001
  C5-Branched dibasic acid metabolism 0.0003 0.0062
  Lysine biosynthesis 0.0005 0.0074
  Nitrogen metabolism 0.0008 0.0116
  Sulfur metabolism 0.0008 0.0099
  Amino sugar and nucleotide sugar metabolism 0.001 0.0120
  Galactose metabolism 0.002 0.0135
  Glycerolipid metabolism 0.004 0.0305
  Glutathione metabolism 0.005 0.0319
  Alanine, aspartate and glutamate metabolism 0.005 0.0332
  Cyanoamino acid metabolism 0.007 0.0426
  Aminoacyl-tRNA biosynthesis 0.008 0.0437
  Vitamin B6 metabolism 0.009 0.0449
     COG enrichment  
   Pathway_MS pvalue_MS qvalue_MS  Methane metabolism 2e-05 0.0006
  Nitrotoluene degradation 0.0002 0.0044
  Lipopolysaccharide biosynthesis 0.0004 0.0071
  Novobiocin biosynthesis 0.0005 0.0066
  Cysteine and methionine metabolism 0.002 0.0206
  Cyanoamino acid metabolism 0.002 0.0218
  Sphingolipid metabolism 0.003 0.0215
  Microbial metabolism in diverse environments 0.003 0.0205
  Sulfur metabolism 0.003 0.0211
  Nucleotide excision repair 0.004 0.0234
  Tyrosine metabolism 0.006 0.0330
  Selenoamino acid metabolism 0.007 0.0366
  Lipoic acid metabolism 0.007 0.0356
     Predicted metabolites from CLR  
   Predicted metabolites Pvalue Overlap with hits  myo-Inositol 0 0.0000
  Trehalose 0 0.0000
  L-Phenylalanine 0.0002 0.0000
  3-(4-Hydroxyphenyl)pyruvate 0.0002 0.0000
  Adenosine 5'-phosphosulfate 0.0002 0.0000
  Thiamin monophosphate 0.0002 0.0000
  L-Tyrosine 0.0004 0.0000
  L-Methionine Sulfoxide 0.0008 0.0000
  Phenylpyruvate 0.0008 0.0000
  D-Carnitine 0.004 0.0000
  Sedoheptulose 7-phosphate 0.004 0.0000
  GTP 0.004 0.0000
  fused thiol:disulfide interchange protein (oxidized) 0.006 0.0000
  fused thiol:disulfide interchange protein (reduced) 0.006 0.0000
  UMP 0.006 0.0000
  S-Adenosyl-L-homocysteine 0.008 0.0000
    
 
