# Supplementary material for: Genomewide landscape of gene–metabolome associations in Escherichia coli
Source: Mol Syst Biol. 2017 Jan 16;13(1):907. doi: 10.15252/msb.20167150 (PMC5293155; doi:10.15252/msb.20167150)
Supplement: Supplementary file 4 — Table EV3 [file MSB-13-907-s004.zip › details/data_ybgL.html]

 
 
 ybgL 
  ybgL - details 
 
 
  CLR  
   Gene_matching CLR_index  ycbC 15.5
  yecH 15.3
  syd 15.1
  dsbC 14.9
  yebE 13.7
  yncI 13.5
  ybcM 13.5
  yedR 13.5
  idnD 13.1
  narJ 13.1
  atoS 13.1
  tyrB 12.2
  ycdB 11.9
  yibA 11.4
  ybeT 11.0
  yoaE 10.8
  yadB 10.7
  gspF 10.2
  agaC 10.0
  yicH 9.6
  gspC 9.2
  bioA 9.1
  malS 9.0
  yeeW 8.8
  ydcD 8.8
  nrfD 8.8
  cusR 8.7
  yfeZ 8.7
  galM 8.5
  ydcL 8.5
  ydcQ 8.4
  lldR 8.4
  hyaD 8.4
  yecR 8.3
  yedD 7.9
  pepT 7.9
  secG 7.8
  lon 7.7
  yedN 7.7
  umuD 7.6
  nrfF 7.6
  btuF 7.5
  grxC 7.5
  yijE 7.4
  ychJ 7.2
  ycfK 7.2
  yedE 7.0
  nudG 7.0
  ycbJ 6.8
  endA 6.8
  cfa 6.7
  yceA 6.5
  sucC 6.5
  yoaH 6.4
  phoQ 6.4
  ycjS 6.3
  nagZ 6.2
  flgM 6.2
  sdhC 6.1
  rrmJ 6.0
  xylR 6.0
  uvrD 5.9
  yfgL 5.9
  flgI 5.9
  yjeB 5.9
  cspG 5.9
  torR 5.9
  recF 5.9
  mog 5.8
  yciV 5.8
  yfcZ 5.8
  rfaF 5.7
  allD 5.6
  yiaI 5.6
  guaC 5.6
  ycfD 5.5
  yodC 5.4
  yeeS 5.3
  yifN 5.2
  yfeD 5.1
  ycaD 5.1
  nfnB 5.1
  flgD 5.1
  yhbP 5.1
  glpD 5.0
  sdhA 5.0
  ydgA 4.9
  hha 4.9
  pldA 4.9
  nrfC 4.9
  pnuC 4.9
  ybcO 4.8
  yobB 4.8
  yraQ 4.8
  tar 4.8
  ydcY 4.8
  tatE 4.8
  narV 4.8
  ybjP 4.7
  yciS 4.7
  hyaB 4.7
  rffG 4.6
  gspE 4.5
  fixC 4.5
  moaE 4.5
  glgX 4.5
  kdpA 4.4
  ykgD 4.4
  ydcJ 4.3
  puuC 4.3
  rffH 4.3
  yejK 4.3
  ypfG 4.2
  yraM 4.2
  phoE 4.2
  fimA 4.2
  ybdD 4.2
  bglB 4.1
  aldA 4.1
  frmR 4.1
  ynbD 4.1
  ydhT 4.0
  arcA 3.9
  emrB 3.9
  aceE 3.9
  yehE 3.9
  cheB 3.8
  rnd 3.8
  ybaM 3.8
  ybcI 3.8
  clpS 3.8
  csgA 3.7
  ybiC 3.7
  fdhE 3.7
  glpE 3.7
  luxS 3.6
  dinB 3.6
  yeaM 3.6
  yqjB 3.6
  ompT 3.6
  exbD 3.6
  glnG 3.6
  pstA 3.5
  citT 3.5
  agaB 3.5
  ycbR 3.5
  ybhB 3.5
  ybdN 3.5
  yqcC 3.4
  yfdM 3.4
  metB 3.4
  yaeP 3.4
  nlpI 3.4
  carB 3.3
  hflX 3.3
  ygdH 3.3
  ydhU 3.3
  ydeU 3.3
  ycjF 3.3
  yfjM 3.3
  yebF 3.2
  creA 3.2
  sbp 3.2
  ybjE 3.2
  chbG 3.2
  yqeI 3.1
  yecF 3.1
  lpcA 3.1
  pphA 3.1
  rpoZ 3.1
  galU 3.1
  rfaH 3.1
  ygdR 3.1
  alr 3.1
  flgE 3.1
  ynfA 3.0
  degP 3.0
  bioD 3.0
  phnK 3.0
  ydcK 3.0
  arcB 3.0
  ydcW 3.0
  yaiC 3.0
  hyuA 3.0
     Differential ions  
   id name formula mz mod AUC Z-score Z-score AUC Weighted   C06056  4-Hydroxy-L-threonine C4H9NO4 134.0474 -H(+) 0.582 -3.526 -0.000
   C03508  L-2-Amino-3-oxobutanoate C4H7NO3 134.0474 +OH(-) 0.571 -3.526 -0.000
   C00441  L-Aspartate 4-semialdehyde C4H7NO3 134.0474 +OH(-) 0.537 -3.526 -0.000
   C00147  Adenine C5H5N5 134.0474 -H(+) 0.520 -3.526 -0.000
   C00144  GMP C10H14N5O8P 380.0630 +OH(-) 0.517 -3.696 -0.000
   C00249  Hexadecanoate (n-C16:0) C16H32O2 256.2353 [+1]-H(+) 0.505 3.591 0.000
   C01228  Guanosine 3',5'-bis(diphosphate) C10H17N5O17P4 625.9571 .H/Na.H(+) 0.502 -3.469 -0.000
   C00144  GMP C10H14N5O8P 282.0856 -HPO3-H(+) 0.494 -3.538 -0.000
   tetradecenoate (n-C14:1)  tetradecenoate (n-C14:1) C14H26O2 243.1958 +OH(-) 0.415 4.444 0.000
   C01304  2,5-Diamino-6-(ribosylamino)-4-(3H)-pyrimidinone 5'-phosphate C9H16N5O8P 625.9571 .(H2PO4K)2.H(+) 0.000 -3.469 -0.000
   C00387  Guanosine C10H13N5O5 282.0856 -H(+) 0.633 -3.538 -2.240
   C00362  dGMP C10H14N5O7P 364.0661 +OH(-) 0.798 -3.635 -2.903
   C18239  cyclic pyranopterin monophosphate C10H14N5O8P 282.0856 -HPO3-H(+) 0.830 -3.538 -2.938
   C18239  cyclic pyranopterin monophosphate C10H14N5O8P 380.0630 +OH(-) 0.825 -3.696 -3.048
     KEGG pathway by CLR  
   Pathway_ion pvalue_ion qvalue_ion  Purine metabolism 3e-06 0.0003
  Vitamin B6 metabolism 0.0001 0.0078
     COG enrichment  
   Pathway_MS pvalue_MS qvalue_MS  RNA polymerase 0 0.0000
  Bacterial secretion system 0.0004 0.0153
  Biotin metabolism 0.001 0.0301
  Polyketide sugar unit biosynthesis 0.002 0.0435
  Two-component system 0.003 0.0486
  Nitrotoluene degradation 0.003 0.0489
  Protein export 0.003 0.0419
  Streptomycin biosynthesis 0.005 0.0566
  D-Alanine metabolism 0.007 0.0651
  alpha-Linolenic acid metabolism 0.007 0.0586
  Citrate cycle (TCA cycle) 0.007 0.0572
  Toluene degradation 0.008 0.0546
     Predicted metabolites from CLR  
   Predicted metabolites Pvalue Overlap with hits  7,8-Diaminononanoate 0 0.0000
  molybdopterin 3e-05 0.0000
  dTDPglucose 0.0001 0.0000
  Thiosulfate 0.0005 0.0000
  Succinate 0.001 0.0000
  D-Glucose 1-phosphate 0.006 0.0000
    
 
