# Supplementary material for: Genomewide landscape of gene–metabolome associations in Escherichia coli
Source: Mol Syst Biol. 2017 Jan 16;13(1):907. doi: 10.15252/msb.20167150 (PMC5293155; doi:10.15252/msb.20167150)
Supplement: Supplementary file 4 — Table EV3 [file MSB-13-907-s004.zip › details/data_ybhP.html]

 
 
 ybhP 
  ybhP - details 
 
 
  CLR  
   Gene_matching CLR_index  folM 40.2
  dppA 25.1
  narX 14.4
  mqo 9.7
  yahO 9.7
  acnA 6.8
  glcB 6.4
  tap 5.1
  yjeK 4.8
  betT 4.5
  ydfO 4.4
  purR 4.2
  yafY 4.2
  uhpA 4.0
  panC 4.0
  rsgA 4.0
  ybjR 3.9
  rnb 3.9
  fiu 3.9
  holD 3.7
  cysH 3.5
  flgK 3.5
  ygcL 3.4
  yehL 3.4
  fldB 3.3
  yafE 3.2
  yfcM 3.2
  fhuC 3.1
  entB 3.1
  yeiW 3.1
  yfbT 3.1
  yphG 3.0
  hslV 3.0
  yegX 3.0
     Differential ions  
   id name formula mz mod AUC Z-score Z-score AUC Weighted   C01449  7-aminomethyl-7-deazaguanine C7H9N5O 218.0429 .H/K.H(+) 0.692 46.308 32.052
   C01449  7-aminomethyl-7-deazaguanine C7H9N5O 218.0429 .K(+) 0.692 46.308 32.052
   C01508  L-Lyxose C5H10O5 189.0161 .H/K.H(+) 0.751 37.150 27.910
   C00312  L-Xylulose C5H10O5 189.0161 .H/K.H(+) 0.672 37.150 24.977
   C00121  D-Ribose C5H10O5 189.0161 .H/K.H(+) 0.653 37.150 24.248
   C00310  D-Xylulose C5H10O5 189.0161 .H/K.H(+) 0.612 37.150 22.736
   C00259  L-Arabinose C5H10O5 189.0161 .H/K.H(+) 0.603 37.150 22.400
   C02637  3-Dehydroshikimate C7H8O5 346.9316 .HPO4K2.H(+) 0.757 29.281 22.159
   C06006  (S)-2-Aceto-2-hydroxybutanoate C6H10O4 169.0539 .H/Na.H(+) 0.794 15.714 12.484
   5'-deoxyribose  5'-deoxyribose C5H10O4 135.0663 .H(+) 0.917 13.530 12.404
   C00966  2-Dehydropantoate C6H10O4 169.0539 .H/Na.H(+) 0.788 15.714 12.379
   C05649  6,7-Dihydropteridine C6H6N4 135.0663 .H(+) 0.853 13.530 11.536
   C06257  1-deoxy-D-xylulose C5H10O4 135.0663 .H(+) 0.748 13.530 10.119
   C04272  (R)-2,3-Dihydroxy-3-methylbutanoate C5H10O4 135.0663 .H(+) 0.689 13.530 9.321
   C02989  L-Methionine Sulfoxide C5H11NO3S 188.0386 .H/Na.H(+) 0.762 11.688 8.912
   C00255  Riboflavin C17H20N4O6 377.1509 .H(+) 0.622 13.641 8.478
   C01216  2-Dehydro-3-deoxy-D-galactonate C6H10O6 450.9152 .(H2PO4K)2.H(+) 0.798 10.350 8.259
   C00120  Biotin C10H16N2O3S 283.0503 .H/K.H(+) 0.634 12.263 7.775
   C03296  N2-Succinyl-L-arginine C10H18N4O5 277.1418 [+2].H(+) 0.761 8.782 6.681
   C00533  Nitric oxide NO 248.9424 .(H2PO4)2NaH.H(+) 0.735 8.753 6.431
   C03892  Phosphatidylglycerophosphate (didodecanoyl, n-C12:0) C30H60O13P2 713.3135 .H/Na.H(+) 0.719 8.608 6.185
   C05649  6,7-Dihydropteridine C6H6N4 157.0454 .H/Na.H(+) 0.967 6.144 5.944
   C04225  cis-2-Methylaconitate C7H8O6 308.9929 .H2PO4Na.H(+) 0.861 6.887 5.932
   5'-deoxyribose  5'-deoxyribose C5H10O4 157.0454 .H/Na.H(+) 0.934 6.144 5.737
   5'-deoxyribose  5'-deoxyribose C5H10O4 157.0454 .Na(+) 0.934 6.144 5.737
   C04171  2,3-Dihydro-2,3-dihydroxybenzoate C7H8O4 157.0454 .H(+) 0.930 6.144 5.713
   C15998  L-methionine-R-sulfoxide C5H11NO3S 166.0509 .H(+) 0.712 7.910 5.629
   C11514  E-3-carboxy-2-pentenedioate 6-methyl ester C7H8O6 308.9929 .H2PO4Na.H(+) 0.701 6.887 4.825
   C06056  4-Hydroxy-L-threonine C4H9NO4 158.0408 .H/Na.H(+) 0.996 4.624 4.606
   C01079  Protoporphyrinogen IX C34H40N4O4 591.2989 .H/Na.H(+) 0.689 6.562 4.521
   C00270  N-Acetylneuraminate C11H19NO9 310.1172 .H(+) 0.782 5.506 4.305
   C00327  L-Citrulline C6H13N3O3 214.0588 .H/K.H(+) 0.777 5.382 4.179
   C00327  L-Citrulline C6H13N3O3 214.0588 .K(+) 0.777 5.382 4.179
   C01571  Decanoate (n-C10:0) C10H20O2 293.1215 .H2PO4Na.H(+) 0.701 5.889 4.128
   C01083  Trehalose C12H22O11 344.1355 [+1].H(+) 0.930 4.428 4.117
   C00719  Glycine betaine C5H11NO2 140.0686 .Na(+) 0.724 5.459 3.951
   C00719  Glycine betaine C5H11NO2 140.0686 .H/Na.H(+) 0.724 5.459 3.951
   C00493  Shikimate C7H10O5 175.0574 .H(+) 0.718 5.309 3.813
   C05775  N1-(alpha-D-ribosyl)-5,6-dimethylbenzimidazole C14H18N2O4 281.1411 [+2].H(+) 0.748 5.007 3.746
   C03974  2-octadec-11-enoyl-sn-glycerol 3-phosphate C21H41O7P1 453.2753 +OH(-) 0.837 4.422 3.701
   C16565  N-3-aminopropyl-1,5-diaminopentane C8H21N3 280.1381 .H2PO4Na.H(+) 0.799 4.631 3.698
   C00522  (R)-Pantoate C6H12O4 171.0611 .H/Na.H(+) 0.686 5.364 3.681
   C06332  N-Acetylanthranilate C9H9NO3 202.0482 .H/Na.H(+) 0.613 5.949 3.645
   C06332  N-Acetylanthranilate C9H9NO3 202.0482 .Na(+) 0.613 5.949 3.645
   C00209  Oxalate C2H2O4 308.9404 .(H2PO4)2NaH.H(+) 0.866 4.104 3.554
   octadecenoate (n-C18:1)  octadecenoate (n-C18:1) C18H34O2 403.2288 .H2PO4Na.H(+) 0.702 4.978 3.494
   C05775  N1-(alpha-D-ribosyl)-5,6-dimethylbenzimidazole C14H18N2O4 280.1381 [+1].H(+) 0.752 4.631 3.484
   C00246  Butyrate (n-C4:0) C4H8O2 209.0125 .H2PO4Na.H(+) 0.706 4.874 3.441
   C00416  1,2-didodecanoyl-sn-glycerol 3-phosphate C27H53O8P1 673.3055 .H2PO4K.H(+) 0.839 4.013 3.367
   C00534  Pyridoxamine C8H12N2O2 305.0221 .H2PO4K.H(+) 0.819 4.095 3.353
   C01909  Dethiobiotin C10H18N2O3 351.0712 .H2PO4K.H(+) 0.832 3.915 3.257
   C00624  N-Acetyl-L-glutamate C7H11NO5 214.0588 [+2].Na(+) 0.604 5.382 3.248
   C00135  L-Histidine C6H9N3O2 194.0315 .H/K.H(+) 0.709 4.578 3.248
   C04236  3-Carboxy-4-methyl-2-oxopentanoate C7H10O5 175.0574 .H(+) 0.608 5.309 3.226
   C05973  2-Acyl-sn-glycero-3-phosphoethanolamine (n-C16:0) C21H44NO7P1 453.2753 [+1]-H(+) 0.723 4.422 3.197
   C00183  L-Valine C5H11NO2 141.0710 [+1].Na(+) 0.622 4.933 3.069
   C01302  1-(2-Carboxyphenylamino)-1-deoxy-D-ribulose 5-phosphate C12H16NO9P 351.0712 [+1].H(+) 0.773 3.915 3.027
   C00019  S-Adenosyl-L-methionine C15H23N6O5S 401.1593 [+1].H(+) 0.815 3.701 3.017
   C00475  Cytidine C9H13N3O5 226.0841 -H2O.H(+) 0.695 4.293 2.985
   C00681  1-octadec-11-enoyl-sn-glycerol 3-phosphate C21H41O7P1 453.2753 +OH(-) 0.650 4.422 2.874
   C04302  N-(5-Phospho-D-ribosyl)anthranilate C12H16NO9P 351.0712 [+1].H(+) 0.731 3.915 2.863
   C00288  Bicarbonate CH2O3 198.9397 .H2PO4K.H(+) 0.703 4.006 2.818
   C00719  Glycine betaine C5H11NO2 118.0861 .H(+) 0.640 4.297 2.750
   C00183  L-Valine C5H11NO2 118.0861 .H(+) 0.635 4.297 2.727
   C08362  Hexadecenoate (n-C16:1) C16H30O2 397.1716 .HPO4Na2.H(+) 0.649 4.177 2.710
   C05402  Melibiose C12H22O11 344.1355 [+1].H(+) 0.602 4.428 2.664
   C00475  Cytidine C9H13N3O5 245.1009 [+1].H(+) 0.652 3.995 2.605
   C00179  Agmatine C5H14N4 305.0221 .HPO4K2.H(+) 0.627 4.095 2.568
   C01530  octadecanoate (n-C18:0) C18H36O2 283.2658 -H(+) 0.645 3.978 2.565
   C06424  tetradecanoate (n-C14:0) C14H28O2 365.1578 .H2PO4K.H(+) 0.696 3.656 2.545
   C05973  2-Acyl-sn-glycero-3-phosphoethanolamine (n-C18:1) C23H46NO7P1 478.2895 -H(+) 0.610 4.158 2.537
   C00188  L-Threonine C4H9NO3 158.0228 .H/K.H(+) 0.690 3.672 2.534
   C04556  4-Amino-2-methyl-5-phosphomethylpyrimidine C6H10N3O4P 258.0062 .H/K.H(+) 0.647 3.852 2.492
   C02107  D-tartrate C4H6O6 151.0278 .H(+) 0.692 3.542 2.452
   C00670  sn-Glycero-3-phosphocholine C8H20NO6P 258.1109 .H(+) 0.628 3.823 2.402
   C05519  L-Allo-threonine C4H9NO3 158.0228 .H/K.H(+) 0.645 3.672 2.367
   C00249  Hexadecanoate (n-C16:0) C16H32O2 257.2389 [+2]-H(+) 0.667 3.481 2.323
   2-Acyl-sn-glycero-3-phosphoglycerol (n-C18:1)  2-Acyl-sn-glycero-3-phosphoglycerol (n-C18:1) C24H47O9P1 509.2846 -H(+) 0.624 3.669 2.290
   C09306  sulfur dioxide O2S 238.8614 .HPO4K2.H(+) 0.613 3.641 2.231
   C00064  L-Glutamine C5H10N2O3 169.0539 .H/Na.H(+) 0.598 15.714 0.000
   C06007  (R)-2,3-Dihydroxy-3-methylpentanoate C6H12O4 171.0611 .H/Na.H(+) 0.595 5.364 0.000
   C00147  Adenine C5H5N5 158.0408 .H/Na.H(+) 0.591 4.624 0.000
   C00093  Glycerol 3-phosphate C3H9O6P 346.9011 .HPO4K2.H(+) 0.579 5.343 0.000
   C00243  Lactose C12H22O11 344.1355 [+1].H(+) 0.577 4.428 0.000
   C00135  L-Histidine C6H9N3O2 179.0611 [+1].Na(+) 0.577 -3.669 -0.000
   C04272  (R)-2,3-Dihydroxy-3-methylbutanoate C5H10O4 157.0454 .H/Na.H(+) 0.575 6.144 0.000
   C04272  (R)-2,3-Dihydroxy-3-methylbutanoate C5H10O4 157.0454 .Na(+) 0.575 6.144 0.000
   octadecenoate (n-C18:1)  octadecenoate (n-C18:1) C18H34O2 419.1968 .H2PO4K.H(+) 0.573 5.043 0.000
   C05973  2-Acyl-sn-glycero-3-phosphoethanolamine (n-C16:1) C21H42NO7P1 451.2614 [+1]-H(+) 0.571 3.672 0.000
   psicoselysine  psicoselysine C12H24N2O7 309.1679 .H(+) 0.571 11.314 0.000
   C00670  sn-Glycero-3-phosphocholine C8H20NO6P 280.0942 .H/Na.H(+) 0.567 8.482 0.000
   C00670  sn-Glycero-3-phosphocholine C8H20NO6P 280.0942 .Na(+) 0.567 8.482 0.000
   C06257  1-deoxy-D-xylulose C5H10O4 157.0454 .H/Na.H(+) 0.565 6.144 0.000
   C06257  1-deoxy-D-xylulose C5H10O4 157.0454 .Na(+) 0.565 6.144 0.000
   C00487  D-Carnitine C7H15NO3 162.1118 .H(+) 0.562 9.336 0.000
   lipoate (protein bound)  lipoate (protein bound) C8H13OS2 190.0506 .H(+) 0.561 4.778 0.000
   C01419  Cys-Gly C5H10N2O3S 450.9152 .(H2PO4K)2.H(+) 0.559 10.350 0.000
   C00345  6-Phospho-D-gluconate C6H13O10P 450.9152 .HPO4K2.H(+) 0.556 10.350 0.000
   C00288  Bicarbonate CH2O3 182.9626 .H2PO4Na.H(+) 0.552 6.970 0.000
   C02989  L-Methionine Sulfoxide C5H11NO3S 167.0559 [+1].H(+) 0.552 4.315 0.000
   C00208  Maltose C12H22O11 344.1355 [+1].H(+) 0.551 4.428 0.000
   C00047  L-Lysine C6H14N2O2 185.0689 .H/K.H(+) 0.550 6.213 0.000
   C00047  L-Lysine C6H14N2O2 185.0689 .K(+) 0.550 6.213 0.000
   C00047  L-Lysine C6H14N2O2 283.0503 .H2PO4K.H(+) 0.549 12.263 0.000
   C03393  4-Phospho-D-erythronate C4H9O8P 450.9152 .(H2PO4)2KH.H(+) 0.549 10.350 0.000
   C00719  Glycine betaine C5H11NO2 156.0421 .H/K.H(+) 0.546 9.312 0.000
   C00719  Glycine betaine C5H11NO2 156.0421 .K(+) 0.546 9.312 0.000
   C00318  L-Carnitine C7H15NO3 162.1118 .H(+) 0.544 9.336 0.000
   C00152  L-Asparagine C4H8N2O3 135.0663 [+2].H(+) 0.544 13.530 0.000
   C00199  D-Ribulose 5-phosphate C5H11O8P 502.8935 .(H2PO4K)2.H(+) 0.542 -3.657 -0.000
   C00263  L-Homoserine C4H9NO3 158.0228 .H/K.H(+) 0.540 3.672 0.000
   C03657  1,4-Dihydroxy-2-naphthoate C11H8O4 227.0344 .H/Na.H(+) 0.536 8.407 0.000
   C00181  D-Xylose C5H10O5 189.0161 .H/K.H(+) 0.532 37.150 0.000
   C00250  Pyridoxal C8H9NO3 190.0506 .H/Na.H(+) 0.531 4.778 0.000
   C00120  Biotin C10H16N2O3S 245.1009 .H(+) 0.527 3.995 0.000
   C16488  fructoselysine C12H24N2O7 309.1679 .H(+) 0.526 11.314 0.000
   C00204  2-Dehydro-3-deoxy-D-gluconate C6H10O6 450.9152 .(H2PO4K)2.H(+) 0.520 10.350 0.000
   C00183  L-Valine C5H11NO2 156.0421 .H/K.H(+) 0.519 9.312 0.000
   C00183  L-Valine C5H11NO2 156.0421 .K(+) 0.519 9.312 0.000
   C06424  tetradecanoate (n-C14:0) C14H28O2 251.1942 .H/Na.H(+) 0.518 25.660 0.000
   C06424  tetradecanoate (n-C14:0) C14H28O2 251.1942 .Na(+) 0.518 25.660 0.000
   C00183  L-Valine C5H11NO2 140.0686 .Na(+) 0.517 5.459 0.000
   C00183  L-Valine C5H11NO2 140.0686 .H/Na.H(+) 0.517 5.459 0.000
   C00064  L-Glutamine C5H10N2O3 147.0763 .H(+) 0.516 11.810 0.000
   C00019  S-Adenosyl-L-methionine C15H23N6O5S 400.1503 .H(+) 0.513 3.771 0.000
   C15998  L-methionine-R-sulfoxide C5H11NO3S 167.0559 [+1].H(+) 0.512 4.315 0.000
   C04114  crotonobetaine C7H13NO2 145.1052 [+1].H(+) 0.511 13.223 0.000
   octadecenoate (n-C18:1)  octadecenoate (n-C18:1) C18H34O2 281.2478 -H(+) 0.511 3.569 0.000
   C05629  Phenylpropanoate C9H10O2 390.9929 .(H2PO4Na)2.H(+) 0.506 -4.029 -0.000
   C00249  Hexadecanoate (n-C16:0) C16H32O2 256.2353 [+1]-H(+) 0.505 4.355 0.000
   C01092  8-Amino-7-oxononanoate C9H17NO3 226.0841 .H/K.H(+) 0.503 4.293 0.000
   C00231  D-Xylulose 5-phosphate C5H11O8P 502.8935 .(H2PO4K)2.H(+) 0.502 -3.657 -0.000
   C00019  S-Adenosyl-L-methionine C15H23N6O5S 382.1433 -H2O.H(+) 0.500 8.610 0.000
   C04294  4-Methyl-5-(2-hydroxyethyl)-thiazole C6H9NOS 144.0483 .H(+) 0.498 10.978 0.000
   C00249  Hexadecanoate (n-C16:0) C16H32O2 255.2327 -H(+) 0.498 4.221 0.000
   C01602  Ornithine C5H12N2O2 155.0820 .H/Na.H(+) 0.498 8.659 0.000
   C00155  L-Homocysteine C4H9NO2S 158.0228 .H/Na.H(+) 0.495 3.672 0.000
   tetradecenoate (n-C14:1)  tetradecenoate (n-C14:1) C14H26O2 265.1570 .H/K.H(+) 0.489 7.517 0.000
   tetradecenoate (n-C14:1)  tetradecenoate (n-C14:1) C14H26O2 265.1570 .K(+) 0.489 7.517 0.000
   C00526  Deoxyuridine C9H12N2O5 149.1134 -HPO3.H(+) 0.489 3.529 0.000
   C00114  Choline C5H13NO 104.1064 .H(+) 0.487 3.884 0.000
   C00719  Glycine betaine C5H11NO2 141.0710 [+1].Na(+) 0.485 4.933 0.000
   C02979  Glycerol 2-phosphate C3H9O6P 346.9011 .HPO4K2.H(+) 0.483 5.343 0.000
   C15998  L-methionine-R-sulfoxide C5H11NO3S 188.0386 .H/Na.H(+) 0.482 11.688 0.000
   C16519  2-succinyl-5-enolpyruvyl-6-hydroxy-3-cyclohexene-1-carboxylate C14H16O9 351.0712 .H/Na.H(+) 0.478 3.915 0.000
   C16519  2-succinyl-5-enolpyruvyl-6-hydroxy-3-cyclohexene-1-carboxylate C14H16O9 351.0712 .Na(+) 0.478 3.915 0.000
   C02989  L-Methionine Sulfoxide C5H11NO3S 166.0509 .H(+) 0.470 7.910 0.000
   C00320  Thiosulfate H2O3S2 354.8649 .(H2PO4Na)2.H(+) 0.467 -3.524 -0.000
   C00898  L-tartrate C4H6O6 151.0278 .H(+) 0.461 3.542 0.000
   C03451  (R)-S-Lactoylglutathione C13H21N3O8S 382.1166 [+2].H(+) 0.456 6.881 0.000
   (2R,4S)-2-methyl-2,3,3,4-tetrahydroxytetrahydrofuran  (2R,4S)-2-methyl-2,3,3,4-tetrahydroxytetrahydrofuran C5H10O5 189.0161 .H/K.H(+) 0.000 37.150 0.000
   C00508  L-Ribulose C5H10O5 189.0161 .H/K.H(+) 0.000 37.150 0.000
   C04332  6,7-Dimethyl-8-(1-D-ribityl)lumazine C13H18N4O6 327.1241 .H(+) 0.000 8.922 0.000
   C04732  4-(1-D-Ribitylamino)-5-aminouracil C9H16N4O6 179.1373 -H3PO4.H(+) 0.000 8.076 0.000
   Fe(III)hydoxamate, unloaded  Fe(III)hydoxamate, unloaded C9H21O6N3 270.1596 [+2].H(+) 0.000 18.131 0.000
   C00166  Phenylpyruvate C9H8O3 436.9172 .(H2PO4K)2.H(+) 0.625 -3.493 -2.182
   C12621  3-hydroxycinnamic acid C9H8O3 436.9172 .(H2PO4K)2.H(+) 0.637 -3.493 -2.226
   C00117  alpha-D-Ribose 5-phosphate C5H11O8P 502.8935 .(H2PO4K)2.H(+) 0.611 -3.657 -2.234
   C00204  2-Dehydro-3-deoxy-D-gluconate C6H10O6 179.0611 .H(+) 0.619 -3.669 -2.270
   C01216  2-Dehydro-3-deoxy-D-galactonate C6H10O6 179.0611 .H(+) 0.637 -3.669 -2.339
   C00442  alpha-D-Ribose 1-phosphate C5H11O8P 502.8935 .(H2PO4K)2.H(+) 0.661 -3.657 -2.417
   C01112  D-Arabinose 5-phosphate C5H11O8P 502.8935 .(H2PO4K)2.H(+) 0.675 -3.657 -2.470
   C00438  N-Carbamoyl-L-aspartate C5H8N2O5 179.0611 [+2].H(+) 0.690 -3.669 -2.531
   C01101  L-Ribulose 5-phosphate C5H11O8P 502.8935 .(H2PO4K)2.H(+) 0.763 -3.657 -2.790
   C00957  Mercaptopyruvate C3H4O3S 256.9313 .H2PO4K.H(+) 0.756 -3.780 -2.859
   C03291  L-Xylulose 5-phosphate C5H11O8P 502.8935 .(H2PO4K)2.H(+) 0.837 -3.657 -3.059
     KEGG pathway by CLR  
   Pathway_ion pvalue_ion qvalue_ion  Lysine degradation 9e-05 0.0078
  Valine, leucine and isoleucine biosynthesis 0.0002 0.0065
  Pantothenate and CoA biosynthesis 0.0004 0.0123
  C5-Branched dibasic acid metabolism 0.0006 0.0136
  Biosynthesis of secondary metabolites 0.0008 0.0109
  Fatty acid biosynthesis 0.0009 0.0113
  Pentose and glucuronate interconversions 0.002 0.0162
  Aminobenzoate degradation 0.002 0.0145
  Ascorbate and aldarate metabolism 0.003 0.0293
  Vitamin B6 metabolism 0.005 0.0355
  Biotin metabolism 0.009 0.0627
     COG enrichment  
   Pathway_MS pvalue_MS qvalue_MS  Bacterial chemotaxis 0.0007 0.0675
  Biosynthesis of siderophore group nonribosomal peptides 0.001 0.0557
  One carbon pool by folate 0.003 0.0876
  DNA replication 0.003 0.0657
  Folate biosynthesis 0.004 0.0794
  Pyruvate metabolism 0.004 0.0689
  Glyoxylate and dicarboxylate metabolism 0.005 0.0637
  Sulfur metabolism 0.006 0.0695
  beta-Alanine metabolism 0.008 0.0823
  Mismatch repair 0.008 0.0740
     Predicted metabolites from CLR  
   Predicted metabolites Pvalue Overlap with hits  CTP 0.0001 0.0000
  L-Malate 0.0007 0.0000
  GTP 0.003 0.0000
    
 
