# Supplementary material for: Genomewide landscape of gene–metabolome associations in Escherichia coli
Source: Mol Syst Biol. 2017 Jan 16;13(1):907. doi: 10.15252/msb.20167150 (PMC5293155; doi:10.15252/msb.20167150)
Supplement: Supplementary file 4 — Table EV3 [file MSB-13-907-s004.zip › details/data_ybhQ.html]

 
 
 ybhQ 
  ybhQ - details 
 
 
  CLR  
   Gene_matching CLR_index  yfjW 9.7
  xerC 8.2
  ydfJ 7.4
  yncG 7.2
  dam 6.8
  yhdY 6.8
  glcG 6.5
  ylaC 6.4
  ydaG 6.3
  gidB 6.0
  nadR 6.0
  ydiK 5.9
  yegJ 5.8
  yegK 5.7
  ynbC 5.6
  lsrG 5.6
  ycjR 5.6
  ydeO 5.5
  yeeT 5.5
  phnL 5.5
  ydcI 5.4
  yeaH 5.3
  yqhA 5.3
  yfcQ 5.3
  yagF 5.3
  abgA 5.3
  thrL 5.2
  ydfO 5.1
  sseA 5.1
  yggS 5.1
  ydeH 5.1
  yobF 5.1
  ydeP 5.1
  yhfT 5.1
  hokD 5.0
  ydeV 5.0
  yibF 5.0
  yfjS 4.9
  ygaM 4.9
  relE 4.9
  wbbI 4.9
  hinT 4.9
  ampE 4.9
  nikC 4.8
  crcA 4.8
  hisQ 4.8
  pbl 4.8
  rhsD 4.8
  lrhA 4.6
  mutM 4.6
  yjiJ 4.6
  yfeR 4.5
  wbbK 4.5
  ymfN 4.4
  yhgG 4.4
  ecpD 4.4
  ycfJ 4.4
  yggU 4.4
  yjaH 4.4
  yfbE 4.4
  mdtA 4.4
  mviM 4.4
  slyA 4.4
  yagH 4.4
  yphB 4.3
  flhD 4.3
  ychE 4.3
  ybjK 4.3
  ydhB 4.3
  mutS 4.3
  pfkB 4.3
  yeaN 4.2
  recO 4.2
  fixA 4.2
  ybgI 4.2
  ykiA 4.2
  ylcG 4.2
  puuB 4.2
  hybA 4.2
  yecT 4.1
  glpR 4.1
  yeaD 4.1
  rhsA 4.1
  iaaA 4.1
  yagI 4.1
  osmY 4.1
  ybeH 4.0
  deaD 4.0
  phnO 4.0
  ydiA 4.0
  yhhI 4.0
  mdtB 3.9
  recN 3.9
  kil 3.9
  cueO 3.9
  gadE 3.9
  yfcU 3.9
  ygdB 3.9
  fimG 3.9
  dcp 3.8
  mltC 3.8
  rsxC 3.8
  fucI 3.8
  sufD 3.8
  yegI 3.8
  yfdS 3.8
  ydjY 3.8
  fbaB 3.8
  yfbT 3.8
  ybiR 3.7
  sgbE 3.7
  glpT 3.7
  yjaA 3.7
  yfgI 3.7
  narU 3.7
  rhsE 3.7
  yidF 3.7
  ygcK 3.7
  gmhB 3.6
  tag 3.6
  udk 3.6
  yfeN 3.6
  yraN 3.6
  hyfI 3.6
  acrA 3.6
  yqeK 3.6
  ycjZ 3.6
  yfbU 3.6
  yifO 3.6
  yjbB 3.6
  yfcS 3.6
  yadM 3.6
  yfdC 3.5
  gidA 3.5
  vacJ 3.5
  kduD 3.5
  ygaY 3.5
  bglH 3.5
  yecH 3.5
  clcB 3.5
  yehD 3.4
  yebU 3.4
  ymdF 3.4
  ygfJ 3.4
  cheZ 3.4
  yhdA 3.4
  bacA 3.4
  ymbA 3.4
  ompG 3.4
  uspF 3.4
  ydiQ 3.4
  nlpA 3.4
  nudD 3.4
  gatR 3.4
  yhhH 3.4
  rem 3.3
  yfeW 3.3
  flhE 3.3
  yebV 3.3
  yjjB 3.3
  ygeQ 3.3
  yeaP 3.3
  ycjD 3.3
  yoaB 3.3
  mdoG 3.3
  ygcG 3.3
  ybbC 3.3
  yfdP 3.3
  yegH 3.2
  yagE 3.2
  eamA 3.2
  yaiT 3.2
  setB 3.2
  ydgD 3.2
  gatB 3.2
  ynjC 3.2
  ycbF 3.2
  ybcL 3.2
  ybfG 3.2
  yehU 3.2
  ygeK 3.2
  xerD 3.1
  fabR 3.1
  exoX 3.1
  yiiL 3.1
  tdcE 3.1
  ycgG 3.1
  ycaM 3.1
  ydhO 3.1
  yecC 3.1
  rhsB 3.1
  gudD 3.1
  ybiX 3.1
  ligT 3.1
  ygcR 3.1
  yfcM 3.1
  clpB 3.1
  rtcB 3.1
  yciX 3.1
  tdcC 3.1
  pbpC 3.1
  yphG 3.0
  gspD 3.0
  hisM 3.0
  yfdL 3.0
  yhdZ 3.0
  yqgC 3.0
  wbbL 3.0
  ydjH 3.0
  ppdB 3.0
  ydiI 3.0
     Differential ions  
   id name formula mz mod AUC Z-score Z-score AUC Weighted   C00445  5,10-Methenyltetrahydrofolate C20H22N7O6 495.1278 .H/K.H(+) 0.688 5.196 3.575
   C00681  1-dodecanoyl-sn-glycerol 3-phosphate C15H31O7P1 475.1399 .H2PO4Na.H(+) 0.557 4.767 0.000
   C03974  2-dodecanoyl-sn-glycerol 3-phosphate C15H31O7P1 475.1399 .H2PO4Na.H(+) 0.545 4.767 0.000
   C00026  2-Oxoglutarate C5H6O5 364.9638 .(H2PO4)2NaH.H(+) 0.538 3.676 0.000
   Hexadecanoyl-phosphate (n-C16:0)  Hexadecanoyl-phosphate (n-C16:0) C16H33O5P 511.1012 .HPO4K2.H(+) 0.523 4.684 0.000
     KEGG pathway by CLR  
   Pathway_ion pvalue_ion qvalue_ion  Lysine biosynthesis 0.0009 0.0917
     COG enrichment  
   Pathway_MS pvalue_MS qvalue_MS  Mismatch repair 0.008 0.8025
     Predicted metabolites from CLR  
   Predicted metabolites Pvalue Overlap with hits  glucosyl-O-acetyl-rhamanosyl-N-acetylglucosamyl-undecaprenyl diphosphate 0 0.0000
  L-Histidine 0.0001 0.0000
  D-Fructose 1,6-bisphosphate 0.0004 0.0000
  Undecaprenyl diphosphate 0.0006 0.0000
  Ornithine 0.001 0.0000
  L-Lysine 0.002 0.0000
  L-Arginine 0.003 0.0000
  GDP 0.005 0.0000
  UDP 0.005 0.0000
    
 
