# Supplementary material for: Genomewide landscape of gene–metabolome associations in Escherichia coli
Source: Mol Syst Biol. 2017 Jan 16;13(1):907. doi: 10.15252/msb.20167150 (PMC5293155; doi:10.15252/msb.20167150)
Supplement: Supplementary file 4 — Table EV3 [file MSB-13-907-s004.zip › details/data_ybiO.html]

 
 
 ybiO 
  ybiO - details 
 
 
  CLR  
   Gene_matching CLR_index  puuA 10.1
  ybiN 9.9
  ybbM 9.5
  ybjT 9.4
  dhaH 9.3
  ybfH 9.2
  nmpC 8.8
  ydaU 8.1
  ymfA 8.0
  yaiZ 7.8
  ydfO 7.5
  ycfQ 7.4
  yegX 7.3
  ybcV 7.2
  ygaQ 7.2
  emrE 7.2
  ychP 7.1
  yahM 7.1
  yccX 7.0
  rzpR 6.9
  lsrF 6.9
  yliA 6.8
  ybjR 6.8
  yfcC 6.6
  paaD 6.3
  yeaV 6.3
  lar 6.1
  yhiL 6.1
  fiu 6.1
  yghT 6.0
  icdC 6.0
  yccU 5.9
  pppA 5.9
  ydgG 5.8
  ycdL 5.7
  ymfE 5.6
  dhaK 5.5
  yccS 5.5
  rcsC 5.5
  ycgL 5.5
  yahH 5.4
  yraK 5.4
  ymfT 5.4
  ydaG 5.4
  yahJ 5.4
  yebW 5.4
  ybiV 5.3
  bglF 5.3
  sieB 5.3
  ykgH 5.3
  ybjI 5.3
  ycdG 5.3
  ydhB 5.2
  garP 5.2
  yafE 5.2
  ynfK 5.1
  ynbA 5.1
  ydjK 5.1
  ydgJ 5.1
  ymdF 5.1
  yncA 5.0
  ymcD 5.0
  rssB 5.0
  yfbL 5.0
  plsX 5.0
  dkgA 5.0
  ycjR 5.0
  fsaA 5.0
  ycdN 4.9
  yebQ 4.8
  ccmB 4.8
  ycgN 4.8
  citF 4.7
  dppB 4.7
  mdlB 4.6
  ykgC 4.6
  paaG 4.6
  ypeA 4.6
  yfcG 4.6
  yphF 4.5
  rstA 4.5
  manZ 4.5
  yaiU 4.5
  kdpE 4.5
  yebV 4.5
  ycbL 4.4
  yphH 4.4
  ydcX 4.4
  ydcO 4.4
  glcB 4.4
  amiC 4.4
  cheY 4.3
  ydbD 4.3
  nuoN 4.3
  hflK 4.3
  yceF 4.3
  ydhO 4.3
  nuoK 4.3
  ydiQ 4.3
  ymfI 4.2
  nlpB 4.2
  yjhE 4.2
  uup 4.2
  ygaR 4.2
  shiA 4.2
  ycgR 4.2
  yfbE 4.1
  betT 4.1
  yphB 4.1
  tap 4.1
  uhpA 4.1
  yjbG 4.1
  gnsB 4.0
  yegW 4.0
  yidZ 4.0
  frdB 4.0
  ybhE 4.0
  yagQ 4.0
  bcsF 4.0
  ycgF 4.0
  yfbT 4.0
  yoeE 4.0
  ynjD 4.0
  ybaA 4.0
  dtd 4.0
  ymbA 4.0
  ymgG 4.0
  dhaL 4.0
  yecT 3.9
  ogrK 3.9
  ycaI 3.9
  fdhF 3.9
  yncK 3.9
  php 3.9
  sseB 3.9
  rzpD 3.9
  yfeA 3.8
  ynaI 3.8
  ygfY 3.8
  yedZ 3.8
  ydcM 3.8
  yfcE 3.8
  cspB 3.8
  hlyE 3.8
  ybcJ 3.8
  yjeK 3.7
  tehB 3.7
  yieM 3.7
  ytjC 3.7
  yfhQ 3.7
  nadB 3.7
  ydcR 3.7
  ycbV 3.7
  ymjA 3.7
  ybhF 3.7
  wcaK 3.7
  vsr 3.7
  ybfG 3.7
  yncJ 3.7
  ycgG 3.7
  hokB 3.6
  yddW 3.6
  marA 3.6
  panC 3.6
  yejH 3.6
  rho 3.6
  eutT 3.6
  abgA 3.6
  ygcL 3.6
  oppD 3.5
  ybeB 3.5
  yfaO 3.5
  truD 3.5
  chpB 3.5
  frlD 3.5
  yeaW 3.5
  talA 3.4
  marB 3.4
  ymgF 3.4
  uhpC 3.4
  puuP 3.4
  yfcJ 3.4
  ydgT 3.4
  hslV 3.4
  tonB 3.4
  etp 3.4
  malM 3.4
  yeaN 3.4
  paaH 3.4
  htgA 3.4
  yciW 3.4
  yhjC 3.4
  ylaC 3.3
  ssuC 3.3
  yhdX 3.3
  flgH 3.3
  ymfO 3.3
  yqjA 3.3
  yfaL 3.3
  zapA 3.2
  ybcK 3.2
  focA 3.2
  ynjC 3.2
  lplA 3.2
  purR 3.2
  frsA 3.2
  dmsA 3.2
  ydeN 3.2
  yeeO 3.2
  yfhM 3.2
  hokD 3.2
  uspF 3.2
  gpmI 3.2
  ynfN 3.2
  nrdI 3.2
  ligB 3.2
  ygcR 3.1
  yjiY 3.1
  ubiG 3.1
  malX 3.1
  ybbO 3.1
  ybgF 3.1
  yggM 3.1
  hokA 3.1
  chbR 3.1
  cadA 3.1
  ybgH 3.1
  yeaL 3.1
  ymdC 3.1
  ydbL 3.1
  ymgC 3.1
  yohH 3.1
  yebU 3.1
  yfiC 3.1
  yeiH 3.0
  eamA 3.0
  ymjC 3.0
  ycdZ 3.0
  yodB 3.0
  rpsT 3.0
  hupB 3.0
  livJ 3.0
  ynfO 3.0
  ycaO 3.0
  yfjO 3.0
  yfcM 3.0
     Differential ions  
   id name formula mz mod AUC Z-score Z-score AUC Weighted   C01585  Hexanoate (n-C6:0) C6H12O2 139.0738 .H/Na.H(+) 0.877 3.741 3.280
   C00262  Hypoxanthine C5H4N4O 139.0508 [+2].H(+) 0.772 4.018 3.103
   C00135  L-Histidine C6H9N3O2 139.0508 -NH3.H(+) 0.568 4.018 0.000
     KEGG pathway by CLR  
   Pathway_ion pvalue_ion qvalue_ion  Caprolactam degradation 3e-07 0.0000
  Histidine metabolism 8e-05 0.0037
  Microbial metabolism in diverse environments 9e-05 0.0027
  Ethylbenzene degradation 0.0008 0.0181
  Bisphenol degradation 0.001 0.0230
  beta-Alanine metabolism 0.002 0.0343
  Aminoacyl-tRNA biosynthesis 0.002 0.0324
  Ascorbate and aldarate metabolism 0.006 0.0655
  ABC transporters 0.01 0.1037
     COG enrichment  
   Pathway_MS pvalue_MS qvalue_MS  Limonene and pinene degradation 0.002 0.2422
  Aminobenzoate degradation 0.003 0.1316
  Glycerolipid metabolism 0.004 0.1309
  Bisphenol degradation 0.004 0.1037
  Phosphonate and phosphinate metabolism 0.004 0.0830
     Predicted metabolites from CLR  
   Predicted metabolites Pvalue Overlap with hits  Dihydroxyacetone 4e-06 0.0000
  D-Glycerate 2-phosphate 6e-05 0.0000
  Dodecanoly-phosphate (n-C12:0) 6e-05 0.0000
  Hexadecanoyl-phosphate (n-C16:0) 6e-05 0.0000
  Hexadecanoyl-phosphate (n-C16:1) 6e-05 0.0000
  Octadecanoyl-phosphate (n-C18:0) 6e-05 0.0000
  Octadecanoyl-phosphate (n-C18:1) 6e-05 0.0000
  Tetradecanoyl-phosphate (n-C14:0) 6e-05 0.0000
  Tetradecanoyl-phosphate (n-C14:1) 6e-05 0.0000
  3-Phospho-D-glycerate 0.0002 0.0000
  Ring 1,2-epoxyphenylacetyl-CoA 0.001 0.0000
  D-Fructose 6-phosphate 0.002 0.0000
  Choline 0.003 0.0000
  D-Glucose 6-phosphate 0.004 0.0000
  D-Mannose 6-phosphate 0.006 0.0000
    
 
