# Supplementary material for: Genomewide landscape of gene–metabolome associations in Escherichia coli
Source: Mol Syst Biol. 2017 Jan 16;13(1):907. doi: 10.15252/msb.20167150 (PMC5293155; doi:10.15252/msb.20167150)
Supplement: Supplementary file 4 — Table EV3 [file MSB-13-907-s004.zip › details/data_ybiR.html]

 
 
 ybiR 
  ybiR - details 
 
 
  CLR  
   Gene_matching CLR_index  ynhG 11.7
  yfdC 9.9
  yeaJ 9.5
  mioC 8.6
  lrhA 8.5
  yoaC 7.2
  yedZ 7.2
  yagF 7.0
  wbbK 6.8
  fixA 6.7
  fliZ 6.6
  yeiW 6.6
  ycfJ 6.4
  yegD 6.4
  yegJ 6.4
  rem 6.4
  ybbO 6.2
  ydiK 6.1
  mutM 6.0
  ydgJ 5.8
  clcB 5.8
  tdk 5.8
  yeaH 5.8
  yhcE 5.8
  yfbT 5.8
  ycdW 5.7
  yohC 5.6
  ydfO 5.5
  yoaD 5.5
  yecM 5.5
  yiiL 5.4
  ompW 5.4
  yecT 5.4
  ynjC 5.4
  yegX 5.4
  hokA 5.3
  eutI 5.2
  yfeX 5.2
  yfcS 5.2
  glnP 5.2
  yodB 5.0
  yagT 5.0
  sufD 5.0
  yfjZ 5.0
  yagE 5.0
  nudD 5.0
  mdtI 4.9
  yadL 4.9
  gidA 4.9
  yqgB 4.9
  yohN 4.9
  dinG 4.8
  kil 4.7
  gnsB 4.6
  trmC 4.6
  pioO 4.6
  yjbB 4.6
  asnB 4.6
  potB 4.6
  rtcB 4.5
  aqpZ 4.5
  ydhZ 4.5
  yfdL 4.5
  rsxC 4.5
  sufB 4.5
  ecpD 4.5
  garL 4.4
  ybeH 4.3
  yfcJ 4.3
  phnA 4.3
  yfdP 4.3
  astC 4.3
  yeaN 4.3
  mviM 4.3
  yeaD 4.2
  ydiO 4.2
  yohK 4.2
  yhdW 4.2
  ybcL 4.2
  hokD 4.2
  glpR 4.2
  ychE 4.2
  yfcM 4.2
  yafT 4.2
  fadB 4.2
  yqgC 4.2
  ydeP 4.2
  iscS 4.1
  gabT 4.1
  hinT 4.1
  yadH 4.1
  ybbC 4.1
  yebV 4.1
  yebW 4.1
  modC 4.1
  alsA 4.1
  yobF 4.1
  yeiP 4.0
  yhaC 4.0
  yafQ 4.0
  yebU 4.0
  mutT 4.0
  ydhB 4.0
  dam 4.0
  hisM 4.0
  pbl 3.9
  envC 3.9
  yhdZ 3.9
  wbbL 3.9
  ydfJ 3.9
  yncC 3.9
  clpB 3.9
  yfaV 3.9
  ydjX 3.8
  pfkB 3.8
  sgcX 3.8
  citC 3.8
  clpX 3.8
  yegH 3.8
  yfeR 3.7
  ybhQ 3.7
  ybgI 3.7
  apaG 3.7
  yggS 3.7
  azoR 3.7
  ygcR 3.7
  yeiJ 3.7
  eamA 3.7
  nadR 3.7
  crcA 3.7
  slyA 3.7
  udk 3.7
  yfiP 3.6
  tdcB 3.6
  yihE 3.6
  yncG 3.6
  modA 3.6
  yfgJ 3.6
  mutS 3.6
  marA 3.6
  cmr 3.6
  yhcC 3.5
  yphB 3.5
  mppA 3.5
  ompG 3.5
  yjgZ 3.5
  ycjZ 3.5
  lsrG 3.5
  cueO 3.5
  wbbI 3.5
  pabC 3.4
  yphH 3.4
  ydeH 3.4
  mdtB 3.4
  ygfS 3.4
  smtA 3.4
  sseB 3.3
  relE 3.3
  flgL 3.3
  ynjI 3.3
  yedY 3.3
  yfgH 3.3
  uhpB 3.3
  yfbE 3.3
  phnH 3.3
  yfjD 3.3
  gltS 3.3
  ydeN 3.2
  ysdC 3.2
  manY 3.2
  lldD 3.2
  nikD 3.2
  ppdB 3.2
  setB 3.2
  yqeK 3.2
  hyfC 3.2
  yjiW 3.2
  yeeT 3.2
  frvR 3.1
  yjeK 3.1
  eutB 3.1
  poxA 3.1
  frlD 3.1
  dcp 3.1
  prpE 3.1
  intD 3.1
  ydjY 3.1
  gabD 3.1
  yeeO 3.1
  ygfJ 3.0
  yhhI 3.0
  fliO 3.0
  argT 3.0
  yfcQ 3.0
  uspF 3.0
  gatR 3.0
  hybE 3.0
  ydiQ 3.0
  yidL 3.0
  mltC 3.0
  fdhE 3.0
  thrL 3.0
  hisA 3.0
     Differential ions  
   id name formula mz mod AUC Z-score Z-score AUC Weighted   C00979  O-Acetyl-L-serine C5H9NO4 104.0705 -CO2.H(+) 0.644 5.556 3.576
   C01005  O-Phospho-L-serine C3H8NO6P 186.0162 .H(+) 0.795 3.512 2.790
   C15809  dehydroglycine C2H3NO2 313.9433 .(H2PO4Na)2.H(+) 0.679 3.853 2.616
   C00979  O-Acetyl-L-serine C5H9NO4 186.0162 .H/K.H(+) 0.683 3.512 2.397
   C00979  O-Acetyl-L-serine C5H9NO4 186.0162 .K(+) 0.683 3.512 2.397
   C01005  O-Phospho-L-serine C3H8NO6P 223.9722 .H/K.H(+) 0.633 3.715 2.352
   C01005  O-Phospho-L-serine C3H8NO6P 223.9722 .K(+) 0.633 3.715 2.352
   C00704  Superoxide anion O2 206.8865 .HPO4K2.H(+) 0.636 3.686 2.343
   C00283  Hydrogen sulfide H2S 208.8846 .HPO4K2.H(+) 0.578 3.665 0.000
   C00160  Glycolate C2H4O3 212.9526 .H2PO4K.H(+) 0.570 3.708 0.000
   C01079  Protoporphyrinogen IX C34H40N4O4 607.2643 .H/K.H(+) 0.566 -3.623 -0.000
   octadecenoate (n-C18:1)  octadecenoate (n-C18:1) C18H34O2 283.2669 .H(+) 0.561 3.667 0.000
   C00334  4-Aminobutanoate C4H9NO2 104.0705 .H(+) 0.557 5.556 0.000
   C00007  O2 O2 206.8865 .HPO4K2.H(+) 0.526 3.686 0.000
   3-Aminoacrylate  3-Aminoacrylate C3H5NO2 261.9275 .HPO4K2.H(+) 0.515 5.001 0.000
   C01326  Hydrogen cyanide CHN 261.9275 .(H2PO4)2KH.H(+) 0.481 5.001 0.000
   3-Aminoacrylate  3-Aminoacrylate C3H5NO2 223.9722 .H2PO4K.H(+) 0.477 3.715 0.000
   C00596  2-Oxopent-4-enoate C5H6O3 116.0372 [+1].H(+) 0.477 -3.477 -0.000
   C00217  D-Glutamate C5H9NO4 104.0705 -CO2.H(+) 0.000 5.556 0.000
   C00217  D-Glutamate C5H9NO4 186.0162 .H/K.H(+) 0.000 3.512 0.000
   C00217  D-Glutamate C5H9NO4 186.0162 .K(+) 0.000 3.512 0.000
   2-Acyl-sn-glycero-3-phosphoglycerol (n-C18:1)  2-Acyl-sn-glycero-3-phosphoglycerol (n-C18:1) C24H47O9P1 647.2341 .H2PO4K.H(+) 0.639 -5.986 -3.824
     KEGG pathway by CLR  
   Pathway_ion pvalue_ion qvalue_ion  D-Glutamine and D-glutamate metabolism 2e-05 0.0015
  Arginine and proline metabolism 0.0003 0.0128
  Alanine, aspartate and glutamate metabolism 0.0006 0.0161
  C5-Branched dibasic acid metabolism 0.001 0.0230
  Aminoacyl-tRNA biosynthesis 0.001 0.0184
  Nitrogen metabolism 0.002 0.0278
  Sulfur metabolism 0.002 0.0238
  Butanoate metabolism 0.002 0.0243
  Glycine, serine and threonine metabolism 0.003 0.0240
  Cysteine and methionine metabolism 0.003 0.0239
  Fatty acid biosynthesis 0.003 0.0257
  Dioxin degradation 0.006 0.0423
  Ethylbenzene degradation 0.006 0.0391
  Valine, leucine and isoleucine degradation 0.009 0.0494
  Biosynthesis of unsaturated fatty acids 0.01 0.0529
     COG enrichment  
   Pathway_MS pvalue_MS qvalue_MS  Phosphonate and phosphinate metabolism 0.003 0.2828
     Predicted metabolites from CLR  
   Predicted metabolites Pvalue Overlap with hits  glucosyl-O-acetyl-rhamanosyl-N-acetylglucosamyl-undecaprenyl diphosphate 0 0.0000
  [2Fe-1S] desulfurated iron-sulfur cluster 0.0001 0.0000
  tungstate 0.0002 0.0000
  Succinic semialdehyde 0.001 0.0000
  [4Fe-4S] iron-sulfur cluster 0.002 0.0000
  SufBCD with bound [4Fe-4S] cluster 0.002 0.0000
  [2Fe-2S] iron-sulfur cluster 0.003 0.0000
  Molybdate 0.004 0.0000
  SufBCD with two bound [2Fe-2S] clusters 0.004 0.0000
  Ornithine 0.008 0.0000
  Sulfate 0.008 0.0000
    
 
