# Supplementary material for: Genomewide landscape of gene–metabolome associations in Escherichia coli
Source: Mol Syst Biol. 2017 Jan 16;13(1):907. doi: 10.15252/msb.20167150 (PMC5293155; doi:10.15252/msb.20167150)
Supplement: Supplementary file 4 — Table EV3 [file MSB-13-907-s004.zip › details/data_ybiT.html]

 
 
 ybiT 
  ybiT - details 
 
 
  CLR  
   Gene_matching CLR_index  malG 17.7
  artP 12.6
  glpF 10.8
  deoC 9.9
  xylE 9.8
  acrD 9.5
  rfaY 9.4
  xapB 8.7
  fdoI 8.6
  yjeK 8.4
  frwC 7.6
  gltL 7.4
  ycfH 7.3
  frc 7.2
  pnuC 7.1
  rbsK 7.0
  mgtA 6.9
  kdpA 6.8
  flhB 6.8
  sbp 6.8
  rbn 6.7
  yjfJ 6.7
  hycG 6.4
  frwB 6.3
  nupC 6.3
  flgK 6.2
  ypfI 6.0
  btuB 5.9
  rhaT 5.8
  yjfK 5.7
  tauD 5.7
  yfcV 5.6
  yeaK 5.6
  asmA 5.5
  araE 5.5
  feaR 5.4
  ydgK 5.3
  srlB 5.2
  ygeR 5.2
  ybdR 5.1
  ileS 5.1
  coaA 5.1
  ddpB 5.1
  yehA 5.0
  exbD 5.0
  agaS 5.0
  gltK 5.0
  gshA 4.9
  thiQ 4.9
  hyaF 4.9
  tatE 4.9
  alsB 4.9
  metL 4.9
  glnP 4.8
  yqgA 4.8
  rluC 4.8
  glpB 4.8
  holD 4.8
  ydiT 4.7
  yjfC 4.7
  hcaT 4.7
  ushA 4.6
  osmE 4.6
  cpdB 4.6
  rep 4.6
  ydcV 4.6
  cheR 4.5
  pheP 4.5
  ilvM 4.4
  yeeW 4.4
  yjdF 4.4
  gspO 4.3
  yjfL 4.3
  osmC 4.3
  pdxJ 4.3
  ccmG 4.2
  hemX 4.2
  intB 4.2
  uxuB 4.2
  cysH 4.2
  htpX 4.2
  ptsG 4.2
  yebG 4.1
  kdpC 4.1
  yecF 4.1
  treC 4.1
  metB 4.0
  secB 4.0
  ccmH 4.0
  ydiS 4.0
  ygeL 4.0
  coaE 4.0
  glmM 3.9
  fldB 3.9
  ybcN 3.9
  fumA 3.8
  hsdS 3.8
  flgE 3.8
  chbA 3.8
  nagE 3.8
  rfe 3.8
  glnH 3.7
  yraP 3.7
  yhbS 3.7
  gltJ 3.7
  betT 3.6
  fdhF 3.6
  nlpI 3.6
  znuB 3.6
  yedK 3.6
  evgS 3.6
  ynfG 3.6
  yohK 3.6
  agaC 3.6
  hyuA 3.6
  abgB 3.6
  aroP 3.6
  yacH 3.6
  fkpA 3.5
  yliC 3.5
  yhgA 3.5
  citT 3.5
  gadX 3.5
  dicC 3.5
  fdhE 3.5
  yafZ 3.5
  ydgI 3.4
  ybhL 3.4
  metH 3.4
  ycaN 3.4
  caiT 3.4
  yqeG 3.4
  nrdE 3.4
  dnaQ 3.4
  proV 3.4
  fadD 3.4
  nrdF 3.4
  ybhH 3.3
  sixA 3.3
  phnI 3.3
  ycjP 3.2
  torC 3.2
  pdxA 3.2
  btuE 3.2
  yqjK 3.2
  yegE 3.2
  flgC 3.1
  rsxA 3.1
  gcd 3.1
  sulA 3.1
  fdoG 3.1
  yccR 3.1
  yciH 3.1
  nohB 3.1
  glgP 3.1
  ycdS 3.1
  yjeO 3.1
  fixB 3.1
  sgcB 3.1
  yddL 3.1
  nrdD 3.1
  tam 3.0
  codB 3.0
  tnaA 3.0
  yaiS 3.0
  glnQ 3.0
  caiD 3.0
  sapA 3.0
  yedP 3.0
  yfeX 3.0
  rpmG 3.0
  mcrA 3.0
  kefA 3.0
     Differential ions  
   id name formula mz mod AUC Z-score Z-score AUC Weighted   C00559  Deoxyadenosine C10H13N5O3 252.1082 .H(+) 0.687 5.826 4.001
   C00942  3',5'-Cyclic GMP C10H12N5O7P 517.9472 .HPO4K2-H(+) 0.743 3.729 2.770
   C03974  2-hexadec-9-enoyl-sn-glycerol 3-phosphate C19H37O7P1 425.2390 +OH(-) 0.638 3.750 2.394
   C00989  gamma-hydroxybutyrate C4H8O3 222.9988 .H2PO4Na-H(+) 0.613 3.522 2.158
   C00416  1,2-didodecanoyl-sn-glycerol 3-phosphate C27H53O8P1 557.3196 .H/Na-H(+) 0.605 3.499 2.117
   C00380  Cytosine C4H5N3O 113.0576 [+1].H(+) 0.592 -4.419 -0.000
   C00212  Adenosine C10H13N5O4 306.0618 .H/K.H(+) 0.583 -3.624 -0.000
   C00135  L-Histidine C6H9N3O2 154.0626 -H(+) 0.572 3.909 0.000
   C00475  Cytidine C9H13N3O5 266.0748 .H/Na.H(+) 0.562 -4.379 -0.000
   C00475  Cytidine C9H13N3O5 266.0748 .Na(+) 0.562 -4.379 -0.000
   C00047  L-Lysine C6H14N2O2 283.0503 .H2PO4K.H(+) 0.549 -4.479 -0.000
   C00315  Spermidine C7H19N3 282.0966 .H2PO4K.H(+) 0.523 -4.668 -0.000
   C05198  5'-Deoxyadenosine C10H13N5O3 252.1082 .H(+) 0.517 5.826 0.000
   C00330  Deoxyguanosine C10H13N5O4 306.0618 .H/K.H(+) 0.506 -3.624 -0.000
   C00681  1-hexadec-9-enoyl-sn-glycerol 3-phosphate C19H37O7P1 425.2390 +OH(-) 0.422 3.750 0.000
   C03393  4-Phospho-D-erythronate C4H9O8P 236.9743 .H/Na-H(+) 0.600 -3.686 -2.212
   C00042  Succinate C4H6O4 236.9743 .H2PO4Na-H(+) 0.670 -3.686 -2.469
   C00380  Cytosine C4H5N3O 112.0508 .H(+) 0.601 -4.184 -2.516
   C00475  Cytidine C9H13N3O5 282.0489 .H/K.H(+) 0.653 -3.989 -2.604
   C00475  Cytidine C9H13N3O5 282.0489 .K(+) 0.653 -3.989 -2.604
   C00120  Biotin C10H16N2O3S 283.0503 .H/K.H(+) 0.634 -4.479 -2.840
   C01181  gamma-butyrobetaine C7H15NO2 282.0489 .H2PO4K.H(+) 0.718 -3.989 -2.863
   C01181  gamma-butyrobetaine C7H15NO2 266.0748 .H2PO4Na.H(+) 0.701 -4.379 -3.069
   C00475  Cytidine C9H13N3O5 244.0940 .H(+) 0.720 -4.300 -3.096
     KEGG pathway by CLR  
   Pathway_ion pvalue_ion qvalue_ion  Arachidonic acid metabolism 4e-15 0.0000
  Lysine degradation 3e-07 0.0000
  Biotin metabolism 4e-05 0.0011
  ABC transporters 0.0005 0.0118
  Peptidoglycan biosynthesis 0.002 0.0360
  Oxidative phosphorylation 0.004 0.0458
  beta-Alanine metabolism 0.004 0.0405
  Aminoacyl-tRNA biosynthesis 0.004 0.0416
  Lysine biosynthesis 0.006 0.0550
  Butanoate metabolism 0.009 0.0699
     COG enrichment  
   Pathway_MS pvalue_MS qvalue_MS  Pyrimidine metabolism 2e-05 0.0019
  Pantothenate and CoA biosynthesis 0.0007 0.0304
  ABC transporters 0.001 0.0398
  Arachidonic acid metabolism 0.002 0.0424
  Protein export 0.003 0.0468
  Phosphotransferase system (PTS) 0.003 0.0401
  Vitamin B6 metabolism 0.004 0.0518
  DNA replication 0.006 0.0657
     Predicted metabolites from CLR  
   Predicted metabolites Pvalue Overlap with hits  Pyridoxine 5'-phosphate 0.0005 0.0000
  O-Phospho-4-hydroxy-L-threonine 0.0005 0.0000
  D-Carnitine 0.002 0.0000
  L-Phenylalanine 0.002 0.0000
  Adenosine 0.004 1.0000
  dATP 0.004 0.0000
  dUTP 0.004 0.0000
  Thymidine 0.004 0.0000
  L-Tyrosine 0.004 0.0000
  UTP 0.004 0.0000
  L-Cysteine 0.006 0.0000
  dGTP 0.006 0.0000
  1-deoxy-D-xylulose 5-phosphate 0.006 0.0000
  L-Carnitine 0.009 0.0000
  CTP 0.009 0.0000
  Cytidine 0.009 1.0000
  dADP 0.009 0.0000
  dCTP 0.009 0.0000
  dGDP 0.009 0.0000
  dUDP 0.009 0.0000
    
 
