# Supplementary material for: Genomewide landscape of gene–metabolome associations in Escherichia coli
Source: Mol Syst Biol. 2017 Jan 16;13(1):907. doi: 10.15252/msb.20167150 (PMC5293155; doi:10.15252/msb.20167150)
Supplement: Supplementary file 4 — Table EV3 [file MSB-13-907-s004.zip › details/data_ybiW.html]

 
 
 ybiW 
  ybiW - details 
 
 
  CLR  
   Gene_matching CLR_index  hcaR 11.7
  ydeQ 10.1
  ubiG 8.2
  amtB 8.1
  gatZ 8.0
  mobB 7.9
  yfjG 7.7
  hchA 7.7
  mutY 7.7
  ybbN 7.7
  ykgG 7.4
  yhjC 7.1
  fkpA 7.1
  argO 7.1
  pflA 7.0
  aaeX 6.8
  modE 6.7
  cynR 6.7
  ycbG 6.6
  bioH 6.6
  aceK 6.6
  ascG 6.5
  etp 6.5
  ydcE 6.3
  yrbG 6.3
  yfeS 6.3
  xapR 6.2
  pqqL 6.1
  dgoT 6.0
  yrhA 6.0
  yiiF 5.9
  yeeU 5.7
  cysK 5.7
  yidR 5.6
  sodC 5.4
  ybfN 5.4
  rpsO 5.4
  glnL 5.4
  elaD 5.3
  yieP 5.3
  ylbH 5.3
  yciI 5.3
  ycdU 5.3
  pgpA 5.2
  mcrB 5.2
  ynfG 5.1
  ygcW 5.0
  setC 5.0
  trmA 5.0
  yiiM 5.0
  baeR 4.9
  gldA 4.9
  ydhL 4.9
  marC 4.9
  tfaS 4.8
  mcrC 4.8
  yicM 4.8
  yncH 4.8
  fldB 4.8
  ybjO 4.8
  yegX 4.8
  flhA 4.8
  yggP 4.8
  yjiA 4.7
  gspJ 4.7
  ydbJ 4.7
  ytfK 4.7
  yjjM 4.7
  torS 4.6
  citA 4.6
  malF 4.6
  emtA 4.5
  ilvA 4.5
  ushA 4.5
  rpsT 4.5
  barA 4.5
  tdcR 4.4
  ygcL 4.4
  rtcA 4.4
  yiaY 4.4
  panC 4.4
  ybjG 4.3
  bipA 4.3
  ulaE 4.2
  cspE 4.2
  yjfP 4.2
  thiC 4.2
  yceG 4.2
  yedJ 4.2
  hcaT 4.2
  abrB 4.2
  yjfN 4.2
  yeeA 4.1
  wcaI 4.1
  yedW 4.1
  yddM 4.1
  yedM 4.1
  ycaN 4.1
  yihS 4.1
  entB 4.1
  gpp 4.0
  oxyR 4.0
  appB 4.0
  ivbL 4.0
  ycgJ 4.0
  yhiM 3.9
  yidX 3.9
  hemX 3.9
  yaaY 3.9
  ybbP 3.9
  yohL 3.9
  ybfE 3.9
  flgK 3.9
  yhiP 3.8
  ybdH 3.8
  yjeM 3.8
  gnsB 3.8
  yibI 3.8
  ychM 3.7
  eutP 3.7
  hokC 3.7
  rna 3.7
  ypeB 3.7
  yjgB 3.7
  aidB 3.7
  mhpR 3.6
  asnC 3.6
  yqhC 3.6
  yobA 3.6
  zupT 3.5
  ydfO 3.5
  yeeP 3.5
  yjiK 3.5
  yohM 3.5
  yigG 3.5
  intE 3.5
  yddG 3.5
  dhaR 3.5
  pspE 3.4
  glcB 3.4
  narY 3.4
  fecE 3.4
  ycdH 3.4
  tatB 3.4
  yphH 3.4
  gspG 3.4
  gspH 3.4
  aroC 3.4
  secB 3.4
  dedA 3.4
  gntU 3.3
  pcnB 3.3
  ypfJ 3.3
  ubiE 3.3
  gspO 3.3
  trkD 3.3
  yhjX 3.3
  yiaU 3.3
  yciB 3.3
  yjiT 3.3
  yrfD 3.3
  yeiW 3.3
  panB 3.2
  flgA 3.2
  macB 3.2
  yjeK 3.2
  aroE 3.2
  yigI 3.2
  ppiB 3.2
  argD 3.2
  ulaG 3.2
  yohC 3.2
  cbrC 3.2
  yfdN 3.2
  yjgF 3.2
  ygeH 3.2
  yeaR 3.2
  hisH 3.2
  yhbS 3.1
  ynfC 3.1
  yicI 3.1
  yoeA 3.1
  fnr 3.1
  lpxM 3.1
  hdfR 3.1
  ybfC 3.0
  yqhH 3.0
  bglJ 3.0
  ygcS 3.0
  yfiD 3.0
  rarD 3.0
  ybdL 3.0
  yeaJ 3.0
  ybhH 3.0
  ymfP 3.0
  betT 3.0
  ynjA 3.0
  ycbU 3.0
     Differential ions  
   id name formula mz mod AUC Z-score Z-score AUC Weighted   C00417  cis-Aconitate C6H6O6 210.9633 .H/K-H(+) 0.747 9.797 7.321
   C00680  meso-2,6-Diaminoheptanedioate C7H14N2O4 207.1004 +OH(-) 0.702 9.908 6.952
   C02637  3-Dehydroshikimate C7H8O5 344.9129 .HPO4K2-H(+) 0.675 9.227 6.228
   C01909  Dethiobiotin C10H18N2O3 133.1579 -HPO3-H(+) 0.913 6.674 6.092
   C00937  D-Lactaldehyde C3H6O2 91.0399 +OH(-) 0.799 7.482 5.975
   C00122  Fumarate C4H4O4 133.0147 +OH(-) 0.764 6.923 5.291
   C00186  L-Lactate C3H6O3 328.9404 .(H2PO4Na)2-H(+) 0.649 7.887 5.120
   C01013  3-Hydroxypropanoate C3H6O3 328.9404 .(H2PO4Na)2-H(+) 0.647 7.887 5.100
   C00424  L-Lactaldehyde C3H6O2 91.0399 +OH(-) 0.678 7.482 5.072
   C00497  D-Malate C4H6O5 134.0192 [+1]-H(+) 0.875 5.680 4.969
   C05235  Acetol C3H6O2 91.0399 +OH(-) 0.649 7.482 4.854
   C00937  D-Lactaldehyde C3H6O2 92.0432 [+1]+OH(-) 0.819 5.690 4.659
   C00242  Guanine C5H5N5O 133.0147 -NH3-H(+) 0.670 6.923 4.640
   C00116  Glycerol C3H8O3 91.0399 -H(+) 0.618 7.482 4.626
   C11453  2-C-methyl-D-erythritol 2,4-cyclodiphosphate C5H12O9P2 276.9897 -H(+) 0.886 5.212 4.620
   C01013  3-Hydroxypropanoate C3H6O3 208.9827 .H2PO4Na-H(+) 0.626 6.979 4.366
   C11514  E-3-carboxy-2-pentenedioate 6-methyl ester C7H8O6 187.0193 -H(+) 0.870 4.986 4.335
   C00184  Dihydroxyacetone C3H6O3 208.9827 .H2PO4Na-H(+) 0.621 6.979 4.335
   C00186  L-Lactate C3H6O3 208.9827 .H2PO4Na-H(+) 0.619 6.979 4.323
   C00122  Fumarate C4H4O4 134.0192 [+1]+OH(-) 0.761 5.680 4.321
   C00163  Propionate (n-C3:0) C3H6O2 92.0432 [+1]+OH(-) 0.749 5.690 4.260
   C00424  L-Lactaldehyde C3H6O2 92.0432 [+1]+OH(-) 0.729 5.690 4.151
   C00522  (R)-Pantoate C6H12O4 165.0725 +OH(-) 0.844 4.771 4.028
   C05235  Acetol C3H6O2 92.0432 [+1]+OH(-) 0.674 5.690 3.836
   C03912  1-Pyrroline-5-carboxylate C5H7NO2 134.0192 .H/Na-H(+) 0.668 5.680 3.793
   C00079  L-Phenylalanine C9H11NO2 165.0725 [+1]-H(+) 0.770 4.771 3.674
   C05629  Phenylpropanoate C9H10O2 187.0193 .H/K-H(+) 0.735 4.986 3.665
   C04225  cis-2-Methylaconitate C7H8O6 187.0193 -H(+) 0.689 4.986 3.435
   C01508  L-Lyxose C5H10O5 284.9763 .H2PO4K-H(+) 0.912 3.752 3.420
   C02917  (S)-Propane-1,2-diol C3H8O2 248.9274 .HPO4K2-H(+) 0.658 5.164 3.400
   C02631  2-Isopropylmaleate C7H10O4 330.9414 .HPO4K2-H(+) 0.929 3.627 3.370
   C02912  (R)-Propane-1,2-diol C3H8O2 248.9274 .HPO4K2-H(+) 0.641 5.164 3.313
   C00680  meso-2,6-Diaminoheptanedioate C7H14N2O4 190.0898 [+1]-H(+) 0.789 4.179 3.298
   C01013  3-Hydroxypropanoate C3H6O3 224.9541 .H2PO4K-H(+) 0.702 4.586 3.218
   C00312  L-Xylulose C5H10O5 284.9763 .H2PO4K-H(+) 0.856 3.752 3.210
   C00577  D-Glyceraldehyde C3H6O3 224.9541 .H2PO4K-H(+) 0.689 4.586 3.159
   C11472  D-Glycero-D-manno-heptose 1,7-bisphosphate C7H16O13P2 488.9455 .H2PO4Na-H(+) 0.810 3.839 3.111
   C06187  Arbutin 6-phosphate C12H17O10P 351.0514 -H(+) 0.808 3.729 3.014
   C00186  L-Lactate C3H6O3 224.9541 .H2PO4K-H(+) 0.651 4.586 2.983
   C00256  D-Lactate C3H6O3 224.9541 .H2PO4K-H(+) 0.641 4.586 2.938
   C01100  L-Histidinol phosphate C6H12N3O4P 355.9892 .H2PO4K-H(+) 0.773 3.721 2.875
   C00266  Glycolaldehyde C2H4O2 292.9218 .(H2PO4)2KH-H(+) 0.626 4.443 2.779
   C00184  Dihydroxyacetone C3H6O3 224.9541 .H2PO4K-H(+) 0.605 4.586 2.774
   C00438  N-Carbamoyl-L-aspartate C5H8N2O5 193.0491 +OH(-) 0.726 3.771 2.737
   C02679  Dodecanoate (n-C12:0) C12H24O2 199.1688 -H(+) 0.657 4.063 2.670
   L-alanine-D-glutamate  L-alanine-D-glutamate C8H14N2O5 488.9455 .(H2PO4K)2-H(+) 0.694 3.839 2.664
   C01585  Hexanoate (n-C6:0) C6H12O2 137.0599 .H/Na-H(+) 0.710 3.675 2.608
   C00576  Betaine aldehyde C5H11NO 102.0909 .H(+) 0.685 3.794 2.598
   C00181  D-Xylose C5H10O5 284.9763 .H2PO4K-H(+) 0.678 3.752 2.542
   C12621  3-hydroxycinnamic acid C9H8O3 434.9026 .(H2PO4K)2-H(+) 0.642 3.941 2.529
   C06423  octanoate (n-C8:0) C8H16O2 143.1065 -H(+) 0.659 3.797 2.502
   C00577  D-Glyceraldehyde C3H6O3 360.8865 .(H2PO4K)2-H(+) 0.655 3.798 2.488
   C03415  N2-Succinyl-L-ornithine C9H16N2O5 351.0514 .H2PO4Na-H(+) 0.663 3.729 2.472
   C00380  Cytosine C4H5N3O 92.0242 -H2O-H(+) 0.671 3.647 2.446
   C00719  Glycine betaine C5H11NO2 355.9892 .(H2PO4Na)2-H(+) 0.649 3.721 2.416
   C00310  D-Xylulose C5H10O5 284.9763 .H2PO4K-H(+) 0.642 3.752 2.410
   C00262  Hypoxanthine C5H4N4O 276.9736 .HPO4Na2-H(+) 0.638 3.765 2.402
   L-alanine-L-glutamate  L-alanine-L-glutamate C8H14N2O5 488.9455 .(H2PO4K)2-H(+) 0.612 3.839 2.349
   C06424  tetradecanoate (n-C14:0) C14H28O2 227.2001 -H(+) 0.654 3.565 2.331
   C01233  sn-Glycero-3-phosphoethanolamine C5H14NO6P 355.9892 .HPO4Na2-H(+) 0.625 3.721 2.324
   C00631  D-Glycerate 2-phosphate C3H7O7P 456.8445 .(H2PO4K)2-H(+) 0.661 3.490 2.308
   C00197  3-Phospho-D-glycerate C3H7O7P 456.8445 .(H2PO4K)2-H(+) 0.657 3.490 2.292
   C15767  gamma-glutamyl-gamma aminobutyric acid C9H16O5N2 351.0514 .H2PO4Na-H(+) 0.609 3.729 2.270
   C12622  cis-3-(3-carboxyethenyl)-3,5-cyclohexadiene-1,2-diol C9H10O4 137.0599 -CO2-H(+) 0.608 3.675 2.235
   Dodecanoly-phosphate (n-C12:0)  Dodecanoly-phosphate (n-C12:0) C12H25O5P1 317.0943 .H/K-H(+) 0.599 4.009 0.000
   C06423  octanoate (n-C8:0) C8H16O2 165.0896 .H/Na-H(+) 0.594 4.462 0.000
   octanoate (protein bound)  octanoate (protein bound) C8H14O 143.1065 +OH(-) 0.594 3.797 0.000
   C12623  2,3-dihydroxicinnamic acid C9H8O4 216.9902 .H/K-H(+) 0.588 4.055 0.000
   C00183  L-Valine C5H11NO2 355.9892 .(H2PO4Na)2-H(+) 0.587 3.721 0.000
   C00149  L-Malate C4H6O5 133.0147 -H(+) 0.584 6.923 0.000
   C00266  Glycolaldehyde C2H4O2 194.9448 .H2PO4K-H(+) 0.582 6.090 0.000
   C00031  D-Glucose C6H12O6 412.9658 .(H2PO4)2KH-H(+) 0.582 -3.485 -0.000
   C00385  Xanthine C5H4N4O2 133.0147 -H2O-H(+) 0.575 6.923 0.000
   C00186  L-Lactate C3H6O3 360.8865 .(H2PO4K)2-H(+) 0.573 3.798 0.000
   C00033  Acetate C2H4O2 194.9448 .H2PO4K-H(+) 0.571 6.090 0.000
   C01279  4-Amino-5-hydroxymethyl-2-methylpyrimidine C6H9N3O 371.9793 .(H2PO4)2KH-H(+) 0.570 5.574 0.000
   C00149  L-Malate C4H6O5 134.0192 [+1]-H(+) 0.567 5.680 0.000
   C02341  trans-Aconitate C6H6O6 210.9633 .H/K-H(+) 0.565 9.797 0.000
   C00256  D-Lactate C3H6O3 360.8865 .(H2PO4K)2-H(+) 0.563 3.798 0.000
   L-Prolinylglycine  L-Prolinylglycine C7H12N2O3 190.0898 [+1]+OH(-) 0.561 4.179 0.000
   C00097  L-Cysteine C3H7NO2S 138.0224 +OH(-) 0.559 5.197 0.000
   C00334  4-Aminobutanoate C4H9NO2 124.0402 .H/Na-H(+) 0.557 3.512 0.000
   C00447  Sedoheptulose 1,7-bisphosphate C7H16O13P2 488.9455 .H2PO4Na-H(+) 0.557 3.839 0.000
   C00227  Acetyl phosphate C2H5O5P 176.9355 .H/K-H(+) 0.556 4.730 0.000
   C00022  Pyruvate C3H4O3 108.9912 .H/Na-H(+) 0.554 5.452 0.000
   C00184  Dihydroxyacetone C3H6O3 360.8865 .(H2PO4K)2-H(+) 0.553 3.798 0.000
   C00256  D-Lactate C3H6O3 208.9827 .H2PO4Na-H(+) 0.553 6.979 0.000
   C00385  Xanthine C5H4N4O2 107.0362 -CO2-H(+) 0.550 -3.827 -0.000
   C00460  dUTP C9H15N2O14P3 488.9455 .H/Na-H(+) 0.549 3.839 0.000
   C07086  Phenylacetic acid C8H8O2 276.9897 .HPO4Na2-H(+) 0.549 5.212 0.000
   C00345  6-Phospho-D-gluconate C6H13O10P 448.8912 .HPO4K2-H(+) 0.547 5.037 0.000
   C00047  L-Lysine C6H14N2O2 165.1174 [+2]+OH(-) 0.546 5.549 0.000
   C00497  D-Malate C4H6O5 133.0147 -H(+) 0.545 6.923 0.000
   C00166  Phenylpyruvate C9H8O3 434.9026 .(H2PO4K)2-H(+) 0.544 3.941 0.000
   C01585  Hexanoate (n-C6:0) C6H12O2 115.0750 -H(+) 0.543 3.719 0.000
   C00259  L-Arabinose C5H10O5 284.9763 .H2PO4K-H(+) 0.540 3.752 0.000
   C00184  Dihydroxyacetone C3H6O3 328.9404 .(H2PO4Na)2-H(+) 0.536 7.887 0.000
   C11457  3-(3-hydroxy-phenyl)propionate C9H10O3 121.0655 -CO2-H(+) 0.532 20.201 0.000
   C00033  Acetate C2H4O2 292.9218 .(H2PO4)2KH-H(+) 0.530 4.443 0.000
   C00124  D-Galactose C6H12O6 412.9658 .(H2PO4)2KH-H(+) 0.530 -3.485 -0.000
   C01013  3-Hydroxypropanoate C3H6O3 360.8865 .(H2PO4K)2-H(+) 0.527 3.798 0.000
   C03287  L-Glutamate 5-phosphate C5H10NO7P 247.9936 .H/Na-H(+) 0.527 5.310 0.000
   C04044  3-(2,3-Dihydroxyphenyl)propanoate C9H10O4 137.0599 -CO2-H(+) 0.523 3.675 0.000
   C00116  Glycerol C3H8O3 92.0432 [+1]-H(+) 0.517 5.690 0.000
   C06187  Arbutin 6-phosphate C12H17O10P 369.0577 +OH(-) 0.515 4.542 0.000
   C00121  D-Ribose C5H10O5 284.9763 .H2PO4K-H(+) 0.512 3.752 0.000
   C00148  L-Proline C5H9NO2 114.0565 -H(+) 0.510 3.511 0.000
   C00186  L-Lactate C3H6O3 107.0362 +OH(-) 0.509 -3.827 -0.000
   C00455  NMN C11H15N2O8P 351.0514 +OH(-) 0.506 3.729 0.000
   C00064  L-Glutamine C5H10N2O3 163.0752 +OH(-) 0.484 3.930 0.000
   C01419  Cys-Gly C5H10N2O3S 448.8912 .(H2PO4K)2-H(+) 0.475 5.037 0.000
   tetradecenoate (n-C14:1)  tetradecenoate (n-C14:1) C14H26O2 247.1636 .H/Na-H(+) 0.469 3.986 0.000
   C00793  D-Cysteine C3H7NO2S 138.0224 +OH(-) 0.464 5.197 0.000
   C00989  gamma-hydroxybutyrate C4H8O3 103.0398 -H(+) 0.464 3.926 0.000
   C00222  Malonate semialdehyde C3H4O3 108.9912 .H/Na-H(+) 0.464 5.452 0.000
   C00163  Propionate (n-C3:0) C3H6O2 91.0399 +OH(-) 0.459 7.482 0.000
   C00577  D-Glyceraldehyde C3H6O3 208.9827 .H2PO4Na-H(+) 0.456 6.979 0.000
   C11145  methanesulfonate CH4O3S 94.9806 -H(+) 0.453 4.715 0.000
   C00253  Nicotinate C6H5NO2 122.0268 -H(+) 0.453 9.230 0.000
   C05512  Deoxyinosine C10H12N4O4 270.0919 [+1]+OH(-) 0.453 6.858 0.000
   C01419  Cys-Gly C5H10N2O3S 350.9192 .HPO4K2-H(+) 0.451 4.195 0.000
   C00337  (S)-Dihydroorotate C5H6N2O4 276.9897 .H2PO4Na-H(+) 0.447 5.212 0.000
   C00256  D-Lactate C3H6O3 328.9404 .(H2PO4Na)2-H(+) 0.443 7.887 0.000
   C01179  3-(4-Hydroxyphenyl)pyruvate C9H8O4 216.9902 .H/K-H(+) 0.427 4.055 0.000
   C00184  Dihydroxyacetone C3H6O3 107.0362 +OH(-) 0.427 -3.827 -0.000
   C00577  D-Glyceraldehyde C3H6O3 328.9404 .(H2PO4Na)2-H(+) 0.413 7.887 0.000
   C16186  L-ascorbate-6-phosphate C6H9O9P 276.9736 .H/Na-H(+) 0.409 3.765 0.000
   C06007  (R)-2,3-Dihydroxy-3-methylpentanoate C6H12O4 165.0725 +OH(-) 0.401 4.771 0.000
   C00601  Phenylacetaldehyde C8H8O 137.0599 +OH(-) 0.380 3.675 0.000
   C01551  Allantoin C4H6N4O3 276.9897 .H2PO4Na-H(+) 0.350 5.212 0.000
   C05512  Deoxyinosine C10H12N4O4 269.0883 +OH(-) 0.320 7.458 0.000
   (2R,4S)-2-methyl-2,3,3,4-tetrahydroxytetrahydrofuran  (2R,4S)-2-methyl-2,3,3,4-tetrahydroxytetrahydrofuran C5H10O5 284.9763 .H2PO4K-H(+) 0.000 3.752 0.000
   C00508  L-Ribulose C5H10O5 284.9763 .H2PO4K-H(+) 0.000 3.752 0.000
   C00666  LL-2,6-Diaminoheptanedioate C7H14N2O4 190.0898 [+1]-H(+) 0.000 4.179 0.000
   C00666  LL-2,6-Diaminoheptanedioate C7H14N2O4 207.1004 +OH(-) 0.000 9.908 0.000
   C01268  5-Amino-6-(5'-phosphoribosylamino)uracil C9H15N4O9P 624.9228 .(H2PO4K)2-H(+) 0.000 3.645 0.000
   C02737  phosphatidylserine (ditetradec-7-enoyl, n-C14:1) C34H62N1O10P1 696.3931 .H/Na-H(+) 0.000 3.731 0.000
   C00095  D-Fructose C6H12O6 412.9658 .(H2PO4)2KH-H(+) 0.633 -3.485 -2.207
   C00137  myo-Inositol C6H12O6 412.9658 .(H2PO4)2KH-H(+) 0.654 -3.485 -2.280
   C01487  D-Allose C6H12O6 412.9658 .(H2PO4)2KH-H(+) 0.692 -3.485 -2.414
   C00159  D-Mannose C6H12O6 412.9658 .(H2PO4)2KH-H(+) 0.705 -3.485 -2.457
   C00256  D-Lactate C3H6O3 107.0362 +OH(-) 0.666 -3.827 -2.547
   C00577  D-Glyceraldehyde C3H6O3 107.0362 +OH(-) 0.765 -3.827 -2.927
   C12622  cis-3-(3-carboxyethenyl)-3,5-cyclohexadiene-1,2-diol C9H10O4 301.0110 .H2PO4Na-H(+) 0.632 -4.649 -2.937
   C04044  3-(2,3-Dihydroxyphenyl)propanoate C9H10O4 301.0110 .H2PO4Na-H(+) 0.715 -4.649 -3.324
   C01013  3-Hydroxypropanoate C3H6O3 107.0362 +OH(-) 0.921 -3.827 -3.523
     KEGG pathway by CLR  
   Pathway_ion pvalue_ion qvalue_ion  Microbial metabolism in diverse environments 8e-10 0.0000
  Fatty acid biosynthesis 3e-05 0.0008
  Aminobenzoate degradation 0.0002 0.0031
  Oxidative phosphorylation 0.0002 0.0036
  Bisphenol degradation 0.0003 0.0039
  Toluene degradation 0.0004 0.0043
  Lysine degradation 0.0005 0.0052
  Caprolactam degradation 0.0007 0.0056
  Xylene degradation 0.001 0.0076
  Biosynthesis of secondary metabolites 0.001 0.0101
  Ethylbenzene degradation 0.002 0.0147
  Butanoate metabolism 0.003 0.0188
  Two-component system 0.004 0.0202
  Peptidoglycan biosynthesis 0.005 0.0262
  Naphthalene degradation 0.008 0.0396
     COG enrichment  
   Pathway_MS pvalue_MS qvalue_MS  Bacterial secretion system 6e-05 0.0059
  Protein export 0.004 0.2084
  Chloroalkane and chloroalkene degradation 0.008 0.2598
  Naphthalene degradation 0.008 0.1949
     Predicted metabolites from CLR  
   Predicted metabolites Pvalue Overlap with hits  2-Oxobutanoate 0.0002 0.0000
  S-Adenosyl-L-homocysteine 0.001 0.0000
  Propanoyl-CoA 0.003 0.0000
  S-Adenosyl-L-methionine 0.003 0.0000
    
 
